# Supplementary material for: Examining the Availability and Accessibility of Rehabilitation Services in a Rural District of South Africa: A Mixed-Methods Study
Source: Int J Environ Res Public Health. 2021 Apr 28;18(9):4692. doi: 10.3390/ijerph18094692 (PMC8125304; doi:10.3390/ijerph18094692)
Supplement: Supplementary file 1 [file ijerph-18-04692-s001.zip › Supplementary 1 In-depth Interview Guide.pdf]

Adults with Physical Disabilities

Demographic Information

1. Male or female
2. Age
3. Occupation
4. Highest educational qualification obtained
5. Social Grant status
6. Household size
7. Dependents
8. Other income earners in the house
9. Relationship status
10. Length of time in current district of residence

Background on disability

1. Type of disability
2. Anatomical area affected by disability
3. History and onset of disability
4. Use of any assistive devices
5. Adaptations to the home/workplace to facilitate independence
6. Extent of functional independence
7. How presence of disability has affected his/her life

Barriers and Facilitators: Access to rehabilitation services

1. Understanding of intention of rehabilitation service
2. Nearest rehabilitation provider
3. Nearest health facility for general health inquiries
4. Previous encounters with the rehabilitation services
5. How to get to and return from health facility for rehabilitation services
6. Procedure to be seen by rehabilitation provider
7. Interactions with rehabilitation providers
8. Intervals between rehabilitation appointments
9. What makes going to the health facility for rehabilitation easier?

10. What would a successful rehabilitation service enable you to do?

Overcoming Barriers

1. Challenges to engaging with rehabilitation services
2. What should change to improve ease of access to rehabilitation services?
