# Supplementary material for: Examining the Availability and Accessibility of Rehabilitation Services in a Rural District of South Africa: A Mixed-Methods Study
Source: Int J Environ Res Public Health. 2021 Apr 28;18(9):4692. doi: 10.3390/ijerph18094692 (PMC8125304; doi:10.3390/ijerph18094692)
Supplement: Supplementary file 1 [file ijerph-18-04692-s001.zip › Supplementary 2 National Health Commodities Catalogue for PHC facilities.pdf]

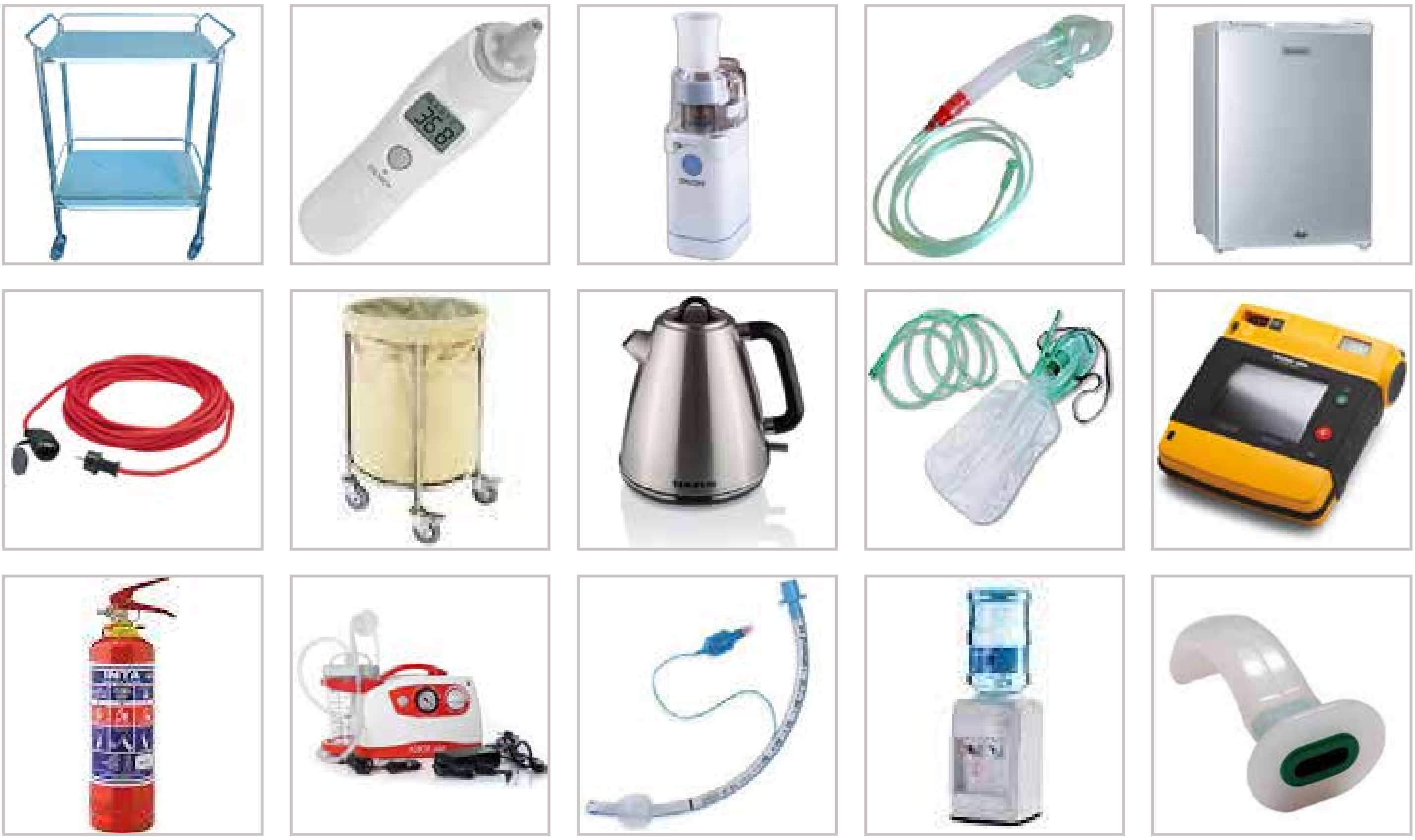

National Health Commodities Catalogue

for Primary Health Care Facilities

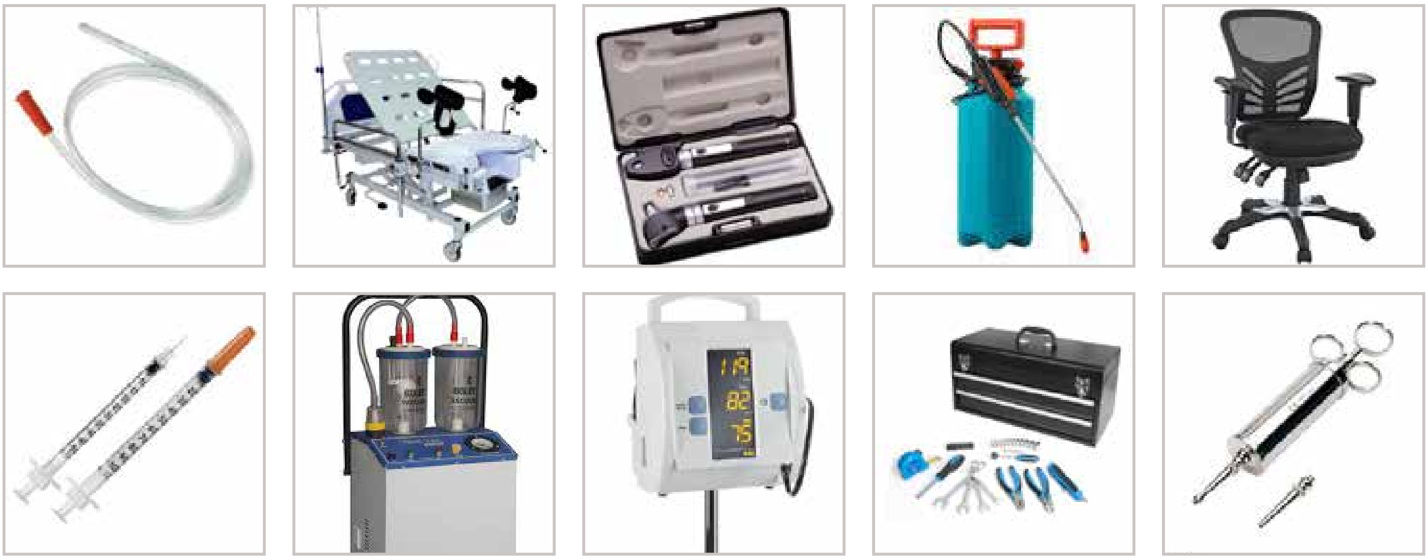

health

Department:
Health
REPUBLIC OF SOUTH AFRICA

2030 NDP

Disclaimer

i While every effort has been exercised to ensure that no copyright has been infringed by the use of curated images in this document, copyright of Internet images cannot always be established. Any such infringement is unintentional, and the image will be removed if the Department is contacted: idealclinic@health.gov.za.

ii No claim is made that the images are true representations of the exact items available on Transversal Contracts. Where such images have been made available, they have been included, to assist recognition of delivered products by end-users. Should the physical appearance of any item change after a subsequent Transversal Contract, the image will be updated in the next edition of this catalogue.

iii Any procurement of items in this catalogue will follow the prescripts of the Public Finance Management Act. Any brand-specific physical features portrayed in the images, are co-incidental, and are not an indication of any preference towards any brand or supplier.

iv The continuation of this project and production of this material were made possible by the support of the American People through the U.S. Agency for International Development (USAID) under the Cooperative Agreement No. AID-674-A-12-00016, awarded to BroadReach. The contents are the responsibility of the South African National Department of Health and do not necessarily reflect the views of USAID or the United States Government.

Design & layout: www.itldesign.co.za

National Health Commodities Catalogue

for Primary Health Care Facilities

acknowledgement

The objectives of PHC can be summed up as the promotion of wellness, prevention of disease, early detection of disease, and effective treatment at the appropriate level of care. The success of the PHC facility to contribute to an improvement in health outcomes is premised on the following three pillars:

- **People** who are motivated, capacitated, empowered, supported, and inspired to serve
- **Processes** that are rational, unambiguous, understandable and clearly enunciated in policies, protocols and guidelines
- **Platform** that includes infrastructure, equipment, medicines, and consumables

This document was produced through the implementation of accepted techniques of quality improvement and standardisation (Operation Phakisa). After an assessment of the existing challenges, the Ideal Clinic, was developed as an umbrella standard with a concurrent plan to achieve that umbrella standard. This includes the standardisation of the quality and minimum quantities of commodities (equipment, instruments, consumables and furniture) in PHC facilities.

The completion of this catalogue was led by Ms Jeanette Hunter and Mr Ramphelane Morewane. Dr Sibusiso Zuma (FH360) did a sterling job of developing this document to final draft stage. Dr Shaidah Asmall did the final review and relevant improvements.

The procurement of commodities has been universally identified as a major obstacle to achieving Ideal Clinic status. Poorly equipped facilities without the necessary consumables are not conducive to the delivery of quality health care. Thus, this Health Commodities Catalogue serves as a tool to assist PHC Facilities in achieving Ideal Clinic status, by specifying:

- The right equipment and consumables required for the specific service needs
- Minimum quality criteria and durability standards
- Adequate quantities required to permit efficient clinical activity

This catalogue is intended to be a dynamic document that will be reviewed and amended regularly to ensure that it meets changing demands within the health sector and to ensure consistent availability of essential equipment and consumables in PHC facilities.

My sincere appreciation to the following officials who were part of the National Specification and Catalogue Committee:

Dr S Abizu NWDOH	Mr F Gebrehemin ECDOH	Ms N Maphumulo KZNDOH	Ms N Ndlovu MPDOH
Dr D Abrahams GDOH	Dr T Gustav KZNDOH	Ms ME Mashao LPDOH	Dr N Ndwamanto LPDOH
Dr S Asmall	Mr R Hussey NDOH	Ms D Matenche National Treasury	Ms D Nkosi MPDOH
Mr S Bakhane NDOH	Mr S Khosa NDOH	Mr MJ Mathobisa FSDOH	Ms P Ntamane GDOH
Mr D Baloyi LPDOH	Mr B Khoza NDOH	Ms E Mere NWDOH	Ms A Railoun NDOH
Ms M Boikanyo NWDOH	Ms H Langa KZNDOH	Ms Z Mnukwana ECDOH	Mr S Rakubu Aurum
Ms M Bok NDOH	Mr R Mabope NDOH	Ms Y Mokgalagadi NDOH	Mr D Selby GDOH
Mr C Clarence KZNDOH	Ms V Mabunda LPDOH	Ms O Mokgatlhe NDOH	Ms O Simango LPDOH
Ms M Dichaba NDOH	Ms L Madikizela NDOH	Mr M Morabe NDOH	Ms D Tshabalala NDOH
Ms M Eckard NCDOH	Mr K Mahlako NDOH	Mr R Morewane NDOH	Dr S Zuma FHI360
Ms I da Gama GDOH	Ms M Makgotlhoe GDOH	Ms N Mpama NDOH	

Thank you to our partner organisations USAID, CDC and the European Union who supported the development of this catalogue.

Ms. M P Matsoso
Director-General: Health
14 May 2018

table of contents

LIST OF ABBREVIATIONS	6
LIST OF ACRONYMS	7
INTRODUCTION	10
EXAMPLE OF ITEM PAGE LAYOUT	13
EXAMPLE OF INSTRUMENTS SPECIFICATIONS PAGE LAYOUT	14
EXAMPLE OF CONSUMABLES ITEM PAGE LAYOUT	15
EXAMPLE OF TECHNICAL SPECIFICATIONS PAGE LAYOUT	16

A. Equipment 17

CLINICAL	17
Diagnostic	18
Essential	39
Specialised	46
Resuscitation	*46*
Point of Care Testing	*61*
Sterilisation	*63*
Oral Health	*67*
Rehabilitation	*88*
GENERAL	117
Building maintenance	118
Cleaning	123
Cold chain	140
Fire safety	144
Garden	147
Information and Communications Technology	166
Kitchen	167
Miscellaneous	172

EQUIPMENT

INSTRUMENTS

B. Instruments 181

Resuscitation	182
Surgical	187
Obstetric	200
Oral health	206
Rehabilitation	259

CONSUMABLES

C. Consumables 267

CLINICAL	267
Clinical stationery	268
Surgical	285
Point of Care Testing	337
Obstetric	346
Oral health	348
Rehabilitation	375
Sterilisation	411
Personal protection equipment	413
GENERAL	421
Building maintenance consumables	422
Cleaning	438
Garden	462
General stationery	463
Miscellaneous	466

D. Furniture & Appliances 469

Clinical 470

General 507

FURNITURE & APPLIANCES

E. Appendices 537

INTRODUCTION TO THE TECHNICAL SPECIFICATIONS 538

APPENDIX 1 :TECHNICAL SPECIFICATIONS 541

Clinical equipment 542

General equipment 631

Furniture and appliances 724

APPENDIX 2 : IDEAL CLINIC CHECKLISTS 797

Ideal clinic checklists 798

Ideal community health centre checklists 824

APPENDICES

INDEX 879

list of abbreviations

±	plus or minus
**b.p.m**	beats per minute
**Incl.**	Including or inclusive
**Units of Measure**	
**cm**	centimetre
**cm.H_2O**	centimetres of water
**l**	litre
**ml**	millilitre
**mm**	millimetre
**mm.Hg**	millimetres of mercury
**kPa**	kilopascal
**kg**	kilogram

list of acronyms

**AAMI**	Association for the Advancement of Medical Instrumentation
**ANSI**	American National Standards Institute
**ABS**	Acrylonitrile Butadiene Styrene: an opaque thermoplastic
**AED**	automated external defibrillator
**BHS**	British Hypertension Society
**BP**	British Pharmacopoeia: Set of standards for medicinal and associated products.
**CD**	compact disk
**CE**	Conformité Européene: A certification mark that indicates conformity with health, safety, and environmental protection standards for products sold within the European Economic Area
**CEE**	International standard from the International Electrotechnical Commission (IEC)
**CFC**	Chlorofluorocarbon: A family of refrigerants, now banned because of harmful environmental effects.
**CKS**	Co-ordinating specification: not yet a national standard.
**DIN**	Deutsches Institut für Normung. German national organization for standardization
**DIY**	Do it yourself
**DVD**	Digital video disk
**ECG**	Electrocardiograph (machine), electrocardiogram (recording)
**EN**	European Norm: European Standards maintained by CEN (European Committee for Standardization), CENELEC and ETSI (European Telecommunications Standards Institute)
**ESH**	European Society for Hypertension
**ET**	Endotracheal tube
**FDA**	Food and Drug Administration. American body regulating medicines and medical devices in the United states of America.
**F**	French: a common measure of diameter of hollow catheters. The F number is calculated as 3X outer diameter of the catheter (in mm), i.e. a 4mm catheter is 12 F. Higher number means thicker.
**g**	Gauge: a common measure of outer diameter of metal wire. Higher number means thinner.

**GDH**	Gauteng Department of Health
**GT**	Gauteng Provincial Tender
**Hb**	Haemoglobin
**beta-HCG**	Human Chorionic Gonadotrophin
**HEPA**	High Efficiency Particulate Arrestance Filter: 99.97% efficient on particles of 0.3microns in size
**HIV**	Human Immunodeficiency Virus
**ICT**	Information and Communications Technology
**IEC**	International Electrotechnical Commission: An international standards organisation that prepares and publishes International Standards for all electrical, electronic and related technologies
**IEEE**	Institute of Electrical and Electronics Engineers
**ISO**	International Standards Organisation
**KDU**	Knock Down Unit
**LAN**	Lime Ammonia Nitrogen fertiliser
**LCD**	Liquid Crystal Display
**LED**	Light Emitting Diode
**MAP**	Mean Arterial Pressure
**mdf**	Medium density fibreboard: Synthetic wood substitute.
**MOD**	Modified
**MOU**	Midwife Obstetric Unit
**MTBF**	Mean Time Before Failure
**NHANES**	US National Health and Nutrition Examination Survey
**NHLS**	National Health Laboratory Services
**OXIMETER**	Instrument for measuring capillary blood oxygen saturation.
**PAWPER**	Paediatric Advanced Weight Prediction in the Emergency Room

**PEEP**	Positive End-Expiratory Pressure
**PHC**	Primary Health Care
**PoCT**	Point of Care Testing
**POPI**	Protection of Personal Information Act
**POP-OFF**	Valve pops off at a defined pressure
**PQS**	World Health Organisation Performance, Quality and Safety process. Defines standards and pre-qualifies cold chain equipment, and publishes a catalogue.
**PTFE**	Polytetrafluoroethylene
**PVC**	Polyvinyl chloride plastic compound
**Rh**	Rhesus Factor
**SABS**	South African Bureau of Standards: A body that has been accredited by SANS to certify that a product meets a SANS specification.
**SANS**	South African National Standards: Publisher of South African standards.
**SCM**	Supply Chain Management
**SMS**	Short Messaging Service
**SOP**	Standing Operational Procedure
**SpO_2**	Peripheral capillary oxygen saturation
**SVS**	Stock Visibility System
**TV**	Television
**USB**	Universal Serial Bus
**VAT**	Value Added Tax
**WBPHCOT**	Ward-Based Primary Health Care Outreach Team
**WHO**	World Health Organisation

introduction

PURPOSE OF THIS DOCUMENT

Procurement of commodities has been universally identified as a major obstacle to achieving Ideal Clinic status.

Poorly equipped facilities are not conducive to delivering quality health care.

This document has been conceived as a tool to assist in achieving Ideal Clinic status, by specifying:

- the right equipment and consumables
- quality and durability standards
- adequate quantities to permit efficient clinical activity.

SCOPE

This document is applicable to Primary Health Care clinics and Community Health Centres.

HOW TO USE THIS DOCUMENT

An Operational Manager should have a working knowledge of the first section. In most cases, the OM will not need to be familiar with the Technical Specifications (Appendix 2), but should understand how the items contribute to positive scores in the various components of the Ideal Clinic Framework (Appendix 2).

HOW THE DOCUMENT IS ORGANIZED

The document has three divisions: the body, Appendix 1 and Appendix 2.

The body of the document contains details of each commodity. The commodities are organised into four groups:

- Equipment, Instruments, Consumables and Furniture and Appliances. Each group is subdivided into two or more subgroups. The divisions will be apparent by consulting the Tables of Content.

For ease of navigation, the pages of each group are colour-coded in the margins.

Equipment and Furniture and **Appliances** are treated similarly.

- Refer to sample on page 13
- Identifying data (catalogue number, standardized name, if available on transversal contracts, relevant contract number)
- Description, including an image, to allow the user to identify correct delivery

- Description of the use of the item
- What the user should expect the item to do/perform
- Accessories supplied as standard with the item
- Expected care of the item by the user
- Warranty information (if relevant)
- Cross reference to the page number of the corresponding Technical Specifications.

Instruments do not have a separate technical specification, as many instruments, e.g. forceps, share the same generic technical specification, and differ only in design. See sample on page 14

Consumables also do not have a separate technical specification, but where there are existing applicable standards, these are listed. There is no warranty information – ideally consumables should be consumed before warranties or expiry dates are exceeded, and faulty batches should be returned immediately. See sample on page 15.

Appendix 1 contains the Technical Specifications for commodities in the categories Equipment and Furniture and Appliances.

- Refer to example on page 16
- These are specifications used to procure items, and will be applicable to forthcoming Transversal Contracts. Provinces and Districts can use these specifications for off-contract procurement
- They are intended to ensure the quality of the commodity items
- Functional specifications describe in technical terms, what the item should be made of, how it should work, and in some cases, how it should look
- Applicable standards are recognized South African or international quality and performance standards, with which the item must comply
- Performance specifications describe the environment in which the item will operate, what outputs it should produce, and how long it should last
- A list of accessories that the supplier must supply with the item
- Warranty terms and conditions expected from the supplier
- Special instructions to suppliers, e.g. to propose optional extras when bidding or quoting
- Note to Supply Chain Management to harmonise the procurement process.

Appendix 2 contains the relevant components from the Ideal Clinic Framework, linking each commodity item to a specific component. Since there are obviously different requirements for clinics and Community Health Centres, there are separate component checklists for the two facility types.

GENERAL EXCLUSIONS APPLICABLE TO ORAL HEALTH AND REHABILITATION SERVICES

Oral Health

This catalogue is not applicable unless a facility is rendering, or preparing to render, Oral Health services.

The Equipment, Instruments and Consumables listed under Oral Health, are required to set up a new dental surgery. Some of the items listed, may not be suitable as replacements for older, existing equipment, because of compatibility issues. Replacements should be found on the relevant Treasury Term Contracts. A suitably qualified professional should be consulted before orders are finalised.

- For certain items, the final choice depends on environmental factors, such as availability of power and water. Alternatives are indicated, but a suitably qualified professional should be consulted before orders are finalised.
- The dental units referenced here, are supplied complete with handpieces. Handpieces are also listed separately for replacement purposes, but the choice has been limited to non-fibre-optic, stainless steel body types. Other types will be available on the term contract.
- In all cases, reference should be made to the Treasury Term Contract in force at the time.

Rehabilitation Services

This catalogue is not applicable unless a facility is rendering, or preparing to render, Rehabilitation Services.

Note that

- In addition to the clinical equipment list in the following pages, there are a number of items listed under Equipment General, in the Basic Toolkit. See checklist 213 in Appendix 2.
- Note that the Tools items should only be ordered if:
  - there is a regular Rehabilitation Service provided at the facility;
  - there is a dedicated space for the provision of such service - the space is required to provide a safe working environment for the professional staff concerned;
  - power tools must not be operated where there are no adequate work surfaces, lighting and power sockets;
  - there is secure, lockable storage for the tools when not in use; the tools are expensive and very portable.

example of
item page layout

TECHNICAL SPECIFICATIONS
page
X

Catalogue Number	Item Name	Available on Transversal Contract	Contract Number or Code
Number used in this catalogue for cross-reference purposes	Standardised name of the item	If **yes**, the reference number of the contract is provided, e.g. RT 252 of 2017; or **No**	If there is an active contract, the item number or code assigned in that contract, e.g. RT 252-01-05 ME is provided to facilitate procurement.

Description	A brief description of the item is provided here.	Image of the item appears here
Use	Specifies what the item is used for.	
Clinical or User Specifications	Describes the physical and/or performance characteristics of the item, as required by an end-user.	
Accessories or Related Consumables	Accessories/spares supplied as standard with the item.	
Care	Describes the reasonable care that the user is expected to take of the item.	
Maintenance	Describes specialised care to be performed by third parties. *Only applicable to some items.*	
Supplier Warranty	The period for which the supplier is responsible for durability and performance (excluding deliberate or accidental damage).	

example of
instruments specifications page layout

NO TECHNICAL SPECIFICATIONS NONE

Catalogue Number	Item Name	Available on Transversal Contract	Contract Number or Code
Number used in this catalogue for cross-reference purposes	Standardised name of the item	If **yes**, the reference number of the contract is provided, e.g. RT 252 of 2017; or **No**	If there is an active contract, the item number or code assigned in that contract, e.g. RT 252-01-05 ME is provided to facilitate procurement.

Functional Specifications	These specifications relate to the materials, design and construction of the item in relation to its intended use, e.g. the type of light source (LED), operating voltage (220V), radius of reach, mounting point and length of cable, for a wall-mounted examination light.
Applicable Standards	List of recognised national (e.g. SANS) or international standards (e.g. ISO or CE), with which the item must be certified to comply.
Performance Specifications	These specifications may relate to operating conditions (e.g. ambient temperature), measurable outputs (e.g. temperature reduction in degrees Celsius), or expected longevity (e.g. Mean Time Before Failure)
Accessories or Related Consumables	List of accessories that must be supplied with the item.<br>Optional items, if approved via the procurement process, will be given a separate item number (e.g. a clinical stethoscope with a paediatric head).
Warranties	This section specifies the required duration of the warranty period, and any specific inclusions or exclusions.
Instructions to Suppliers	There may be specific instructions to be given to suppliers during the procurement process, regarding optional equipment, delivery, installation, training, and maintenance.
Note to Procurement	Specific issues to be brought to the attention of Supply Chain Management (SCM) personnel, e.g. a preferred standardised Item Name to be used in future contracts. Also alerts SCM to the fact that certain items must be compatible with other items (serialised).

There is no cross-reference to Appendix 1, but these items may be cross-referenced in Appendix 2.

example of
consumables item page layout

NO TECHNICAL SPECIFICATIONS NONE

Catalogue Number	Item Name	Available on Transversal Contract	Contract Number or Code
Number used in this catalogue for cross-reference purposes	Standardised name of the item	If **yes**, the reference number of the contract is provided, e.g. RT 252 of 2017; or **No**	If there is an active contract, the item number or code assigned in that contract, e.g. RT 252-01-05 ME is provided to facilitate procurement.

Description	A brief description of the item is provided here.	Image of the item appears here
Use	Specifies what the item is used for.	
Applicable Standards	List of recognised national (e.g. SANS) or international standards (e.g. ISO or CE), with which the item must be certified to comply.	
Note to Procurement	Specific issues to be brought to the attention of Supply Chain Management (SCM) personnel, e.g. a preferred standardised Item Name to be used in future contracts. Also alerts SCM to the fact that certain items must be compatible with other items (serialised).	

There is no cross-reference to Appendix 1, but these items may be cross-referenced in Appendix 2.

example of
technical specifications page layout

Catalogue Number	Item Name	Available on Transversal Contract	Contract Number or Code
Number used in this catalogue for cross-reference purposes	Standardised name of the item	If **yes**, the reference number of the contract is provided, e.g. RT 252 of 2017; or **No**	If there is an active contract, the item number or code assigned in that contract, e.g. RT 252-01-05 ME is provided to facilitate procurement.

Functional Specifications	These specifications relate to the materials, design and construction of the item in relation to its intended use, e.g. the type of light source (LED), operating voltage (220V), radius of reach, mounting point and length of cable, for a wall-mounted examination light.
Applicable Standards	List of recognised national (e.g. SANS) or international standards (e.g. ISO or CE), with which the item must be certified to comply.
Performance Specifications	These specifications may relate to operating conditions (e.g. ambient temperature), measurable outputs (e.g. temperature reduction in degrees Celsius), or expected longevity (e.g. Mean Time Before Failure)
Accessories or Related Consumables	List of accessories that must be supplied with the item.<br>Optional items, if approved via the procurement process, will be given a separate item number (e.g. a clinical stethoscope with a paediatric head).
Warranties	This section specifies the required duration of the warranty period, and any specific inclusions or exclusions.
Instructions to Suppliers	There may be specific instructions to be given to suppliers during the procurement process, regarding optional equipment, delivery, installation, training, and maintenance.
Note to Procurement	Specific issues to be brought to the attention of Supply Chain Management (SCM) personnel, e.g. a preferred standardised Item Name to be used in future contracts. Also alerts SCM to the fact that certain items must be compatible with other items (serialised).

A

equipment clinical

DIAGNOSTIC 18

- Blood pressure measurement: aneroid type 18
- Blood pressure monitor: automated non-invasive 19
- Cardiotocograph 20
- Diagnostic set: portable 21
- Diagnostic set: wall mounted 22
- Diagnostic combination set: wall mounted 23
- Electrocardiograph: 12 channel 24
- Foetoscope 25
- Patella hammer 26
- Peak flow meter 27
- Pulse oximeter: stand-alone 28
- Scale: adult, with height board 29
- Scale: adult, without height board 30
- Scale: baby 31
- Stadiometer 32
- Stethoscope 33
- Syringe: ear 34
- Thermometer: digital, ear-type 35
- Thermometer: digital, pen type 36
- Torch: pupillary 37
- Tuning fork 38

ESSENTIAL 39

- Examination light: ceiling-mounted 39
- Examination light: headlamp 40
- Examination light: mobile, without battery 41
- Examination light: wall-mounted 42
- Tape: measuring 43
- Tape: MUAC 44
- Urine collection 45

SPECIALISED 46

Resuscitation 46

- Automated external defibrillator 46
- Cardiac arrest board 48
- Infant warmer: radiant 49
- Infant warmer: mattress 50
- Infant warmer: temperature probe 51
- Laryngoscope set 52
- Monitor: multi-function vital signs, including SpO_2 53
- Nebuliser 54
- Oxygen regulator with flow-meter 55
- Resuscitators: manual 56
- Suction machine: portable 57
- Suction machine: trolley type 58
- Tape: Broselow 59
- Tape: PAWPER and flipper chart 60

Point of Care Testing 61

- Glucometer 61
- Haemoglobinometer 62

Sterilisation 63

- Autoclave: tabletop 63
- Instrument steriliser: desktop 65

Oral Health 67

- Amalgam separator: 2 surgeries 67
- Amalgamator: digital 68
- Autoclave: cassette-type, desktop 69
- Compressor: 2-4 surgeries 70
- Cuspidor/Spittoon/Water Unit 71
- Delivery system: mobile, with hand pieces, non-fibre-optic 72
- Delivery system: fixed, with hand pieces, non-fibre-optic 73
- Dental chair: basic 74
- Dental chair: portable 75
- Dental curing light 76
- Dental handpieces: non-fibre-optic 77
- Dental light: LED 78
- Dental scaler: air 79
- Suction: dry, 2-3 surgeries 80
- Suction: dry, mobile 81
- Suction: wet, 2-4 surgeries 82
- Ultrasonic cleaner 83
- Water distillation 84
- X-ray Apron 85
- X-ray: Digital oral imaging plate with a computer 87

Rehabilitation 88

- Aluminium crutches 88
- Balance pad 89
- Basin: portable, large 90
- Bobath ball 91
- Bobath roller 92
- Construction play equipment 93
- Hot water bottle: standard 94
- Mat: gym 95
- Mirror: handheld 96
- Soccer ball 97
- Standing frame: prone upper standing frame 98
- Tape measure: retractable, soft 99
- Toys: multisensory 100
- Transfer board 101
- Walking frame 102
- Walking stick 104
- Wedges: set of 4 105
- Weight band: ankle and wrist 106
- Wheelchair 107
- Audiometer: portable 109
- Cards: therapy, speech 110
- HiPro box 111
- Noise makers 112
- OAE/AABR screener (portable) 113
- Otolight 114
- Otoscope: portable 115
- Tympanometer: portable, screening 116

Diagnostic

Blood pressure measurement: aneroid type

TECHNICAL SPECIFICATIONS page 542

Catalogue Number	Item Name	Available on Transversal Contract	Contract Number or Code
PHC-E-115	Sphygmomanometer: aneroid	No	

Description	A unit which measures blood pressure.	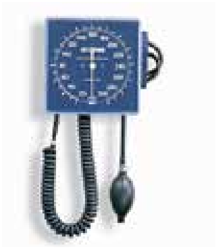
Use	Suitable for blood pressure measurement by a clinician, using the auscultatory method. This is the preferable type if the patient has a disturbance of heart rhythm, e.g. Atrial fibrillation.	
Clinical or User Specifications	The user inflates and deflates an upper-arm cuff, by squeezing a bulb and closing or releasing the exit valve.<br>Blood pressure is displayed on a large, clear, high-contrast analogue gauge, with a dial graduated in mm.Hg.<br>Not dependent on an external power source.<br>The unit can be mounted to a desktop, or to an adjacent wall or pole with special brackets.	
Accessories or Related Consumables	Large adult, adult and paediatric upper-arm cuffs.<br>Special mounting brackets must be ordered separately, if required.	
Care	Clinicians must be adequately trained on the correct identification of the Korotkoff sounds.<br>The cuffs (outer cover and inflatable bladder) can be washed. They must be replaced if worn or damaged, or if the Velcro is no longer adhering.	
Maintenance	The unit must be calibrated at least annually, by technical personnel of the department or the supplier.	
Supplier Warranty	2 years on cuffs and covers (including bladders, tubes, outer covers, inflation bulbs, air inlet valves, release valves, filters, and cuff-fastening mechanism – velcro or other).<br>Instrument itself guaranteed for 10 years, including an annual calibration guarantee.<br>Guarantee excludes deliberate or accidental damage.	

Blood pressure monitor: automated non-invasive

TECHNICAL SPECIFICATIONS page 544

Catalogue Number	Item Name	Available on Transversal Contract	Contract Number or Code
PHC-E-116	Blood pressure monitor: automated, non-invasive, desktop model	RT 2	42272501-00002

Description	An electronic unit which automatically measures and records systolic and diastolic blood pressure and pulse rate. 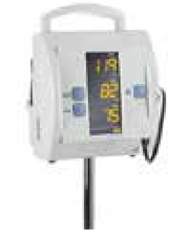
Use	Suitable for blood pressure screening in high patient-load areas, such as where vital signs are recorded. Note: not accurate if the patient has a disturbance of heart rhythm, e.g. Atrial fibrillation, therefore should not be selected for consultation rooms.
Clinical or User Specifications	The unit will automatically inflate and deflate an upper-arm cuff, once the start button is pressed.<br>The unit records and displays the blood pressure and pulse readings, which are also stored in memory until over-written.<br>The unit has a built-in battery, which charges from the mains in normal use, and can keep the unit operating for at least 8 hours, in the event of a power failure.<br>The unit can be mounted to a desktop, or to an adjacent wall or pole with special brackets.
Accessories/related consumables	Large adult, adult and paediatric upper-arm cuffs.<br>Special mounting brackets must be ordered separately, if required.
Care	If the unit does not work, but power is on, have it checked immediately.<br>The internal battery will need replacement, typically after 300 charge-discharge cycles.<br>The cuffs (outer cover and inflatable bladder) can be washed. They must be replaced if worn or damaged, or if the Velcro is no longer adhering.<br>The unit must be calibrated at least annually, preferably 6-monthly.
Maintenance	The unit must be calibrated at least annually, by technical personnel of the department or the supplier.<br>Internal battery must be replaced when prompted to do so by the battery indicator. Contact technical personnel of the department or the supplier.
Supplier Warranty	3 years on all parts, including cuffs and covers, but excluding batteries. Batteries guaranteed for at least 300 charge cycles, or 2 years. Guarantee excludes deliberate or accidental damage.

Cardiotocograph

TECHNICAL SPECIFICATIONS page 547

Catalogue Number	Item Name	Available on Transversal Contract	Contract Number or Code
PHC-E-265	Cardiotocograph	RT2-2016	42181701-00005

Description	An ultra-sound based instrument to detect foetal heart activity in pregnancy.	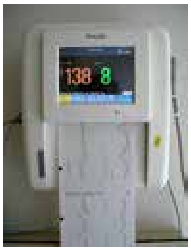
Use	For external monitoring of foetal heart rate, and external monitoring of uterine activity of pregnant patients.	
Clinical or User Specifications	Twins monitoring is required.<br>Must perform automatic self-check when switched on.<br>Visible flashing foetal heartberat indicator foetal heart rate.	
Accessories/related consumables	Ultrasound probes, all connections and power cables.	
Care	Standard cleaning and disinfection.	
Maintenance	The unit must be calibrated at least annually, by technical personnel of the department or the supplier.<br>This unit must be supplied with a 5 - year maintenance contract. Service procedures and service intervals as per Supplier's instructions.	
Supplier Warranty	2 years (excluding deliberate or accidental damage).	

Diagnostic set: portable

TECHNICAL SPECIFICATIONS page 550

Catalogue Number	Item Name	Available on Transversal Contract	Contract Number or Code
PHC-E-117	Diagnostic set: portable	RT 252	RT 252-01-050 ME

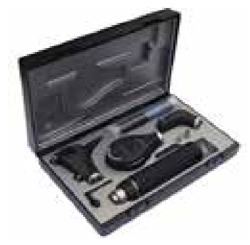

Description	Battery-powered portable set consisting of ophthalmoscope, otoscope and illuminated tongue blade depressor.
Use	Used for examining the eyes (particularly the fundi), the ears and nasal cavity, and the throat.
Clinical or User Specifications	Container with zip or other closure, housing:<br>A mirror-type ophthalmoscope, with standard focussing device and range of lenses;<br>An otoscope, with a fibre-optic light path;<br>Re-usable plastic otoscope specula, in 4 sizes;<br>Illuminated tongue blade depressor, with lamp and holding screw for disposable wooden tongue blades;<br>Knurled handle, housing two non-corrosive batteries; connector for the instruments, and rheostat for brightness adjustment.
Accessories or Related Consumables	1 spare globe ophthalmoscope.<br>1 spare globe otoscope.<br>1 set batteries – standard Size C 1,5V alkaline.
Care	Clean and disinfect with standard solutions.<br>Replace batteries if the light is dim.<br>Check batteries for leakage at least monthly.<br>If globe burns out, replace or have replaced by technical personnel.
Supplier Warranty	2 years.

Diagnostic set: wall mounted

TECHNICAL SPECIFICATIONS page 551

Catalogue Number	Item Name	Available on Transversal Contract	Contract Number or Code
PHC-E-118	Diagnostic set: wall-mounted	No	RT 252-01-05 ME

Description	A wall-mounted unit, combining a wall-mounted cradle, an otoscope, an ophthalmoscope, and the necessary transformer. 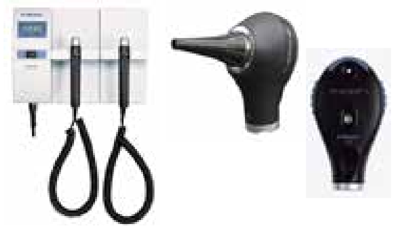
Use	The Ophthalmoscope is used to examine the fundus of the eye, to check for signs of acute disease (e.g. raised intracranial pressure) or chronic disease (e.g. cataracts or vascular changes associated with Diabetes Mellitus or Hypertension.<br>The Otoscope is used to examine the ear canal (wax or foreign bodies) and the middle ear (perforation of eardrum, or signs of fluid or infection in the middle ear).
Clinical or User Specifications	Wall-mounted – cannot be misplaced. Must be mounted on the wall behind the examination couch, towards the head end of the couch.<br>Powered by mains electricity – no batteries.<br>Instruments switch on automatically when lifted out of cradle.<br>Brightness of light is adjustable.<br>Ophthalmoscope has different lenses and filters to accommodate clinical needs.<br>Long cables permit examination irrespective of patient's position.
Accessories or Related Consumables	The Otoscope is supplied with four re-usable specula, from infant to adult sizes.
Care	Keep unit connected to mains.<br>Bulbs are long-life types, but may need replacement from time to time. Contact service personnel immediately.<br>Keep cables from twisting excessively. If cables fray, have them replaced.<br>If unit does not work, but the power is on, have unit checked immediately.<br>Keep a small container of disinfectant solution handy, to clean ear specula after each use.<br>Clean with standard cleaning and disinfectant solutions.
Supplier Warranty	7 years on instrument heads. Cables 2 years. Transformer 1 year (excluding accidental or deliberate damage, power disruptions/surges, lightning strike).

Diagnostic combination set: wall mounted

TECHNICAL SPECIFICATIONS page 553

Catalogue Number	Item Name	Available on Transversal Contract	Contract Number or Code
PHC-E-119	Diagnostic combination set: wall-mounted	No	

Description	A wall-mounted unit, combining a wall-mounted cradle, an otoscope, an ophthalmoscope, an aneroid-type sphygmomanometer, rotatable-head and the necessary transformer.	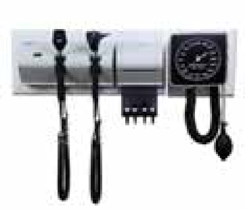
Use	The Ophthalmoscope is used to examine the fundus of the eye, to check for signs of acute disease (e.g. raised intracranial pressure) or chronic disease (e.g. cataracts or vascular changes associated with Diabetes Mellitus or Hypertension.<br>The Otoscope is used to examine the ear canal (wax or foreign bodies) and the middle ear (perforation of eardrum, or signs of fluid or infection in the middle ear).<br>The Sphygmomanometer is used to measure systolic and diastolic blood pressure.	
Clinical or User Specifications	Wall-mounted – cannot be misplaced. Must be mounted on the wall behind the examination couch, towards the head end of the couch.<br>Powered by mains electricity – no batteries.<br>Instruments switch on automatically when lifted out of cradle.<br>Brightness of light is adjustable.<br>Ophthalmoscope has different lenses and filters to accommodate clinical needs.<br>Long cables permit examination irrespective of patient's position.<br>Sphygmomanometer has a large display.	
Accessories/related consumables	The otoscope is supplied with four re-usable specula, from infant to adult sizes.<br>The sphygmomanometer, is supplied with large adult, adult, paediatric and infant cuffs (complete with bladders, cover and tubing and connectors).	
Care	Keep unit connected to mains.<br>Bulbs are long-life types, but may need replacement from time to time. Contact service personnel immediately.<br>Keep cables from twisting excessively. If cables fray, have them replaced.<br>If unit does not work, but the power is on, have unit checked immediately.<br>Keep a small container of disinfectant solution handy, to clean ear specula after each use.<br>Clean with standard cleaning and disinfectant solutions.	
Supplier Warranty	7 years on instrument heads. Cables 2 years. Transformer 1 year (excluding accidental or deliberate damage, power disruptions/surges, lightning strike).	

Electrocardiograph: 12 channel

TECHNICAL SPECIFICATIONS page 554

Catalogue Number	Item Name	Available on Transversal Contract	Contract Number or Code
PHC-E-165	Electrocardiograph (ECG): 12 channel	RT 2-2016	42295104-00001

Description	Trolley-mounted 12-channel ECG machine.	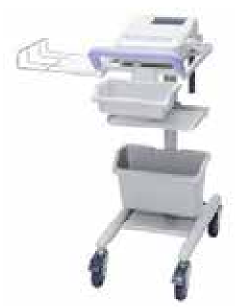
Use	To record the electrical activity of the heart.	
Clinical or User Specifications	Machine records ECG on A4 paper.<br>Three leads are displayed on the built-in screen.<br>Extensive self-test facilities.<br>Built-in measurement and diagnostic algorithms.<br>Can run for 2 hours on battery if there is no mains power.	
Accessories/related consumables	Trolley on four castors.<br>A starter-pack of consumables must be supplied with the unit.<br>ECG cable with adaptor clips.<br>Operator's manual.<br>ECG electrodes: disposable, self-adhesive, for resting ECG. PHC-C-212.	
Care	Keep charged at all times.<br>Run off mains power when possible.	
Supplier Warranty	2 years (excluding deliberate or accidental damage).<br>5-year maintenance contract.	

Foetoscope

NO TECHNICAL SPECIFICATIONS NONE

Catalogue Number	Item Name	Available on Transversal Contract	Contract Number or Code
PHC-E-266	Foetoscope	No	

Description	Pinard horn stethoscope, in plastic or aluminium.	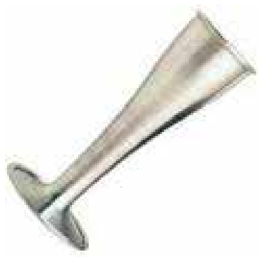
Use	To hear the foetal heartbeat.	
Clinical or User Specifications	No sharp edges.	
Accessories/related consumables		
Care	Standard cleaning and disinfection.	
Supplier Warranty		

Patella hammer

TECHNICAL SPECIFICATIONS page 557

Catalogue Number	Item Name	Available on Transversal Contract	Contract Number or Code
PHC-E-120	Patella hammer	RT 252	RT 252-01-163 ME

Description	Dejerine-pattern percussion hammer.	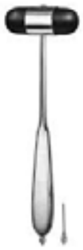
Use	Used for assessing presence and strength of tendon reflexes.	
Clinical or User Specifications	Metal mallet-style hammer head, with cylindrical solid rubber heads on both faces.<br>Metal handle screws into hammer head.<br>Point of handle houses a screw-in pointed tip, for the testing of cutaneous pinprick sensation. However, it's use is not recommended because of possibility of cross-contamination.<br>All metal components are non-rusting.	
Accessories/related consumables		
Care	Clean and disinfect with standard solutions.	
Supplier Warranty	2 years.	

Peak flow meter

TECHNICAL SPECIFICATIONS page 558

Catalogue Number	Item Name	Available on Transversal Contract	Contract Number or Code
PHC-E-267	Peak flow meter	No	RT 252-01-163 ME

Description	A tubular instrument with a mouthpiece and a scale on which the peak flow is read.	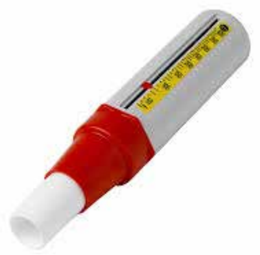
Use	Used to measure the peak expiratory flow (in l/min), which is a proxy for the narrowing of the airways in acute or chronic airways disease. The patient is encouraged to exhale through the instrument as forcefully as possible, and the position of a sliding pointer is read off a scale.	
Clinical or User Specifications	Disposable paper mouthpiece inserts.<br>Direct-reading scale on side of instrument.	
Accessories/related consumables	Disposable mouthpiece PHC-C-341.	
Care	Standard cleaning and disinfection.	
Supplier Warranty	1 year.	

Pulse oximeter: stand-alone

TECHNICAL SPECIFICATIONS page 559

Catalogue Number	Item Name	Available on Transversal Contract	Contract Number or Code
PHC-E-139	Pulse oximeter: stand-alone	RT 4	RT 4-05-055 ME

Description	Portable pulse oximeter.	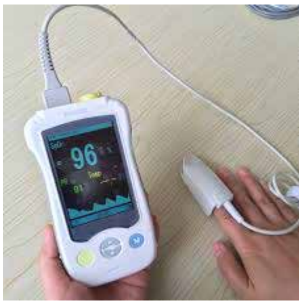
Use	The device measures the oxygenation of peripheral capillary blood, in the fingertip of an adult or the toe of a child or neonate.<br>The degree of oxygenation is useful in measuring the effectiveness of cardiopulmonary resuscitation, as well as the effectiveness of oxygen therapy in patients suffering from chronic lung disease.<br>This device can be used in facilities where the existing vital signs monitor lacks the pulse oximetry capability.	
Clinical or User Specifications	Portable hand-held battery-operated pulse oximeter.	
Accessories or Related Consumables	Adult and paediatric non-disposable finger-tip probes.	
Care	Standard cleaning and disinfecting.<br>Change batteries when prompted to do so by "low battery" indicator.	
Supplier Warranty	2 years (excluding deliberate or accidental damage).	

Scale: adult, with height board

TECHNICAL SPECIFICATIONS page 560

Catalogue Number	Item Name	Available on Transversal Contract	Contract Number or Code
PHC-E-122	Scale: adult, with height rod	RT 24	RT 24-08-005

Description	Adult scale with height rod.	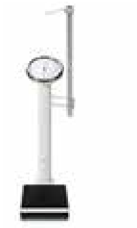
Use	To be used for height and weight screening.	
Clinical or User Specifications	Mechanical scale. Up to 150kg.<br>Incorporates height rod.	
Accessories or Related Consumables		
Care	Standard cleaning and disinfecting.	
Maintenance	Scale must be calibrated at least annually. Contact departmental technical personnel or supplier.	
Supplier Warranty	2 years (excluding deliberate or accidental damage).	

Scale: adult, without height board

TECHNICAL SPECIFICATIONS page 561

Catalogue Number	Item Name	Available on Transversal Contract	Contract Number or Code
PHC-E-123	Scale: adult, without height board	RT 24	RT 24-08-003

Description	Adult scale.	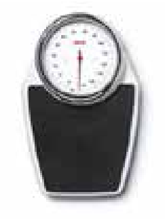
Use	To be used for weight screening.	
Clinical or User Specifications	Mechanical scale, up to 150kg.	
Accessories or Related Consumables		
Care	Standard cleaning and disinfecting.	
Maintenance	Scale must be calibrated at least annually. Contact departmental technical personnel or supplier.	
Supplier Warranty	2 years (excluding deliberate or accidental damage).	

Scale: baby

TECHNICAL SPECIFICATIONS page 562

Catalogue Number	Item Name	Available on Transversal Contract	Contract Number or Code
PHC-E-124	Baby scale	RT 24	RT 24-08-001

Description	Baby scale. 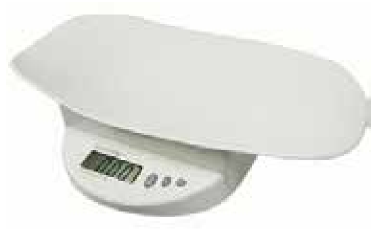
Use	To be used for weighing of babies in consulting rooms.
Clinical or User Specifications	Electronic, battery-operated scale, up to 20kg. Auto switch-off to conserve battery power.<br>Retains last reading.
Accessories or Related Consumables	Set of batteries.
Care	Standard cleaning and disinfecting.<br>Replace battery(ies) with same model cells, when prompted to do so by the battery indicator.<br>If corrosion is noted in the battery contacts, report the item to technical service personnel.
Maintenance	Scale must be calibrated at least annually. Contact departmental technical personnel or supplier.
Supplier Warranty	2 years (excluding deliberate or accidental damage).

Stadiometer

TECHNICAL SPECIFICATIONS page 563

Catalogue Number	Item Name	Available on Transversal Contract	Contract Number or Code
PHC-E-125	Stadiometer	RT 24	RT 24-08-009

Description	Stadiometer. 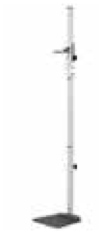
Use	To measure height of children and adults while standing.
Clinical or User Specifications	Wall-mounted height board, not attached to a weight scale.
Accessories or Related Consumables	
Care	Standard cleaning and disinfecting.
Supplier Warranty	2 years (excluding deliberate or accidental damage).

Stethoscope

TECHNICAL SPECIFICATIONS page 564

Catalogue Number	Item Name	Available on Transversal Contract	Contract Number or Code
PHC-E-126	Stethoscope: dual head	No	

Description	A single-tube, rotatable-head general-purpose stethoscope. 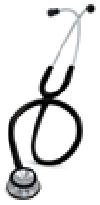
Use	For checking blood pressure, auscultation of the heart, lung fields, abdomen and arterial bruits.
Clinical or User Specifications	The single-tube design eliminates rubbing noises. The instrument has been chosen to selectively amplify sounds of clinical interest, while blocking out environmental noises. The bell or the diaphragm can be rotated into position, depending on the clinical use.<br>For optimal use, the auditory pathway between the patient's body and the clinician's ears, should be airtight.
Accessories or Related Consumables	Spare diaphragm.<br>Spare earpieces (soft and hard).
Care	The instrument should be handled with care.<br>The spring on the headpiece should not be over-extended.<br>Ensure that the earpieces are screwed on firmly, to prevent loss.<br>The instrument should be wiped down with an approved disinfectant regularly.
Supplier Warranty	5 years (excluding deliberate or accidental damage0.

Syringe: ear

TECHNICAL SPECIFICATIONS page 565

Catalogue Number	Item Name	Available on Transversal Contract	Contract Number or Code
PHC-E-127	Syringe:ear	No	

Description	Ear syringe.	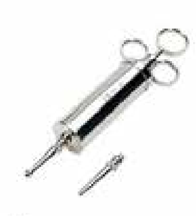
Use	Instrument to remove wax from ears by gentle flushing.	
Clinical or User Specifications	Ergonomic design for easy, single-handed operation.	
Accessories or Related Consumables	Silicone tips.<br>Re-usable silicone tips.	
Care	Standard cleaning and disinfection.	
Supplier Warranty	1 year (excluding deliberate or accidental damage).	

Thermometer: digital, ear-type

TECHNICAL SPECIFICATIONS page 566

Catalogue Number	Item Name	Available on Transversal Contract	Contract Number or Code
PHC-E-130	Thermometer: digital, ear-type	No	

Description	Ear-type thermometer.	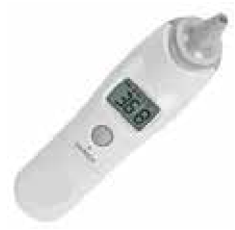
Use	For measuring body temperature.	
Clinical or User Specifications	Electronic, battery powered thermometer, for measuring body temperature at the external auditory meatus. Not suitable for infants – use pen-type thermometer for infants.<br>Batteries may be alkaline type or re-chargeable, depending on model procured.<br>Less affected by high ambient temperature and sweating than no-touch infra-red sensing types.	
Accessories or Related Consumables	Thermometer probe covers: disposable PHC-C-178.	
Care	Standard cleaning and disinfection.<br>Replace batteries with the same model cells when required, or keep batteries charged (depends on model procured).	
Supplier Warranty	2 years.	

Thermometer: digital, pen type

TECHNICAL SPECIFICATIONS page 567

Catalogue Number	Item Name	Available on Transversal Contract	Contract Number or Code
PHC-E-131	Thermometer: digital pen type	No	

Description	Pen-type thermometer.	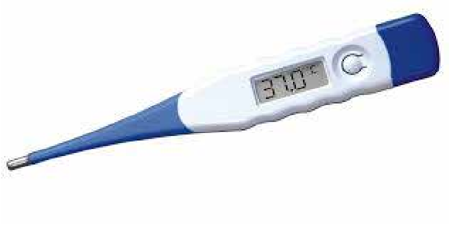
Use	For measuring body temperature.	
Clinical or User Specifications	Electronic device for measuring body temperature by direct contact with skin or mucous membranes.<br>Used for infants – the ear type will not fit in external auditory meatus.	
Accessories or Related Consumables	None.	
Care	Standard cleaning and disinfection.<br>Replace battery(ies) with same model button-type cells, when prompted to do so by the battery indicator.	
Supplier Warranty	2 years.	

Torch: pupillary

TECHNICAL SPECIFICATIONS page 568

Catalogue Number	Item Name	Available on Transversal Contract	Contract Number or Code
PHC-E-121	Torch: pupillary	No	

Description	A small durable battery-operated penlight, with a robust body and switch mechanism. 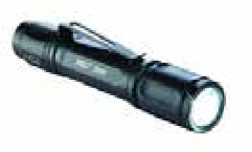
Use	The penlight is used primarily for examining pupillary reflexes and the oral cavity, but also for transilluminating masses.
Clinical or User Specifications	Non-slip body.<br>Positive on-off switch.<br>Single source of uniform white light.<br>Splash-proof.
Accessories/related consumables	Two sets of non-corrosive batteries.
Care	Check batteries from time-to-time. Even non-corrosive batteries can leak after long periods.<br>If bulb burns out, replace the unit.<br>Clean with standard cleaning/disinfecting solutions.
Supplier Warranty	Warranted free of manufacturing defects for a 6-month period.

Tuning fork

TECHNICAL SPECIFICATIONS page 569

Catalogue Number	Item Name	Available on Transversal Contract	Contract Number or Code
PHC-E-132	Tuning fork set	No	

Description	Set of two tuning forks.	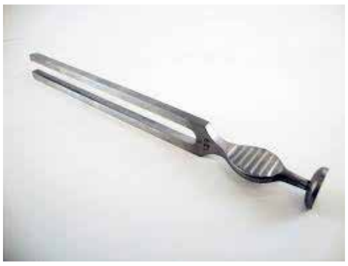
Use	Used to test hearing and vibration sense. Only one set is required in a PHC facility.	
Clinical or User Specifications	The 256Hz fork is generally used for the Rinne, Weber and Schwabach tests of hearing.<br>The 128hz fork is preferable for the assessment of loss of vibration sense, e.g. in suspected diabetic or alcohol-related peripheral neuropathy.	
Accessories or Related Consumables	None.	
Care	Standard cleaning and disinfection.	
Supplier Warranty	None.	

Essential

Examination light: ceiling-mounted

TECHNICAL SPECIFICATIONS page 570

Catalogue Number	Item Name	Available on Transversal Contract	Contract Number or Code
PHC-E-111	Examination light: clinical, ceiling-mounted, directional	RT 2	41111931-00002

Description	A ceiling-mounted examination light. 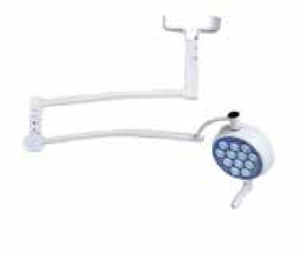
Use	The unit is used to illuminate parts of the patient, during clinical examination. For use where a wall-mounted unit cannot be used.
Clinical or User Specifications	The unit can be positioned according to clinical need, and will maintain the desired position.
Accessories or Related Consumables	None.
Care	Report immediately if unit malfunctions but power is available.<br>Standard cleaning and disinfection.
Supplier Warranty	5 years on all components.

Examination light: headlamp

TECHNICAL SPECIFICATIONS page 571

Catalogue Number	Item Name	Available on Transversal Contract	Contract Number or Code
PHC-E-098	Examination light: headlamp	No	

Description	A lightweight battery-operated light, worn on the forehead.	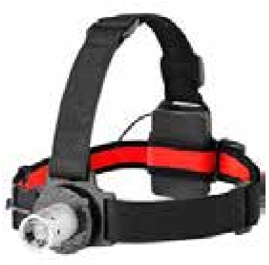
Use	Useful for hands-free illumination of an area of interest, during a clinical examination or simple procedure, or when mains power is unavailable.	
Clinical or User Specifications	Note: this is not a surgical headlamp – the light output is significantly lower.<br>The lightweight lamp is mounted on an adjustable headband. The lamp is adjustable for tilt.<br>Easy-access on-off switch on the lamp unit.<br>Easy-access focusing ring on the lamp, to narrow or widen the beam of light.<br>Belt-worn rechargeable Lithium-ion battery pack.	
Accessories or Related Consumables	Charger, charge cable.	
Care	Washable with standard detergents – do not immerse.<br>Keep charged when not in use.<br>Caution: This type of light maintains a constant light output until the battery level is drained to a certain point, then switches off suddenly to protect the battery. The light does not dim before switching off. Be aware of this possibility when item is being used as the sole source of light.	
Supplier Warranty	1 year (excluding deliberate or accidental damage).	

Examination light: mobile, without battery

TECHNICAL SPECIFICATIONS page 572

Catalogue Number	Item Name	Available on Transversal Contract	Contract Number or Code
PHC-E-154	Examination light: mobile, without battery	RT 2-2016	41111931-00004

Description	Examination light on castors without battery.	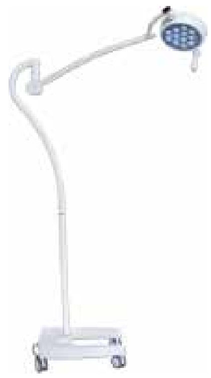
Use	Provides directional concentrated light for clinical examinations.	
Clinical or User Specifications	Bright LED light head.<br>Can be adjusted for optimal lighting position.<br>On a tilt-resistant base, with castors.<br>3meter power cord.	
Accessories or Related Consumables		
Care	Standard cleaning and disinfection.	
Supplier Warranty	5 years including LED light (excluding deliberate or accidental damage).	

Examination light: wall-mounted

TECHNICAL SPECIFICATIONS page 573

Catalogue Number	Item Name	Available on Transversal Contract	Contract Number or Code
PHC-E-110	Examination light: clinical, wall-mounted, directional	RT 2	1111931-00005

Description	A wall-mounted examination light.	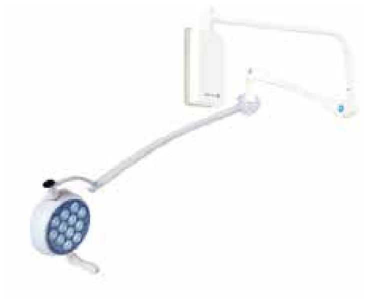
Use	The unit is used to illuminate parts of the patient, during clinical examination or procedures.	
Clinical or User Specifications	Wall-mounted – cannot be misplaced. Must be mounted on the wall behind the examination couch, towards the head end of the couch.<br>The unit can be positioned according to clinical need, and will maintain the desired position.	
Accessories or Related Consumables	None.	
Care	Report immediately if unit malfunctions but power is available.<br>Standard cleaning and disinfection.	
Supplier Warranty	5 years on all components.	

Tape: measuring

TECHNICAL SPECIFICATIONS page 574

Catalogue Number	Item Name	Available on Transversal Contract	Contract Number or Code
PHC-E-113	Tape measure: tailor type	No	

Description	Measuring tape.	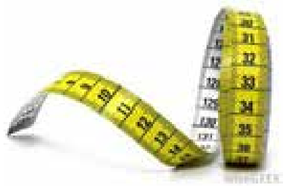
Use	Used for clinical measurements, e.g. waste-hip ratio.	
Clinical or User Specifications	Non-stretch flexible tape.<br>Durable material.<br>Non-fade, non-smudge markings.	
Accessories or Related Consumables	None.	
Care	Standard cleaning and disinfection.	
Supplier Warranty	None.	

Tape: MUAC

NO TECHNICAL SPECIFICATIONS NONE

Catalogue Number	Item Name	Available on Transversal Contract	Contract Number or Code
PHC-E-262	Tape: Mid-Upper Arm Circumference, Child	No	
PHC-E-263	Tape: Mid-Upper Arm Circumference, Adult	No	

Description	A non-stretch vinyl tape, marked in millimetres, for measuring Mid-Upper Arm Circumference.	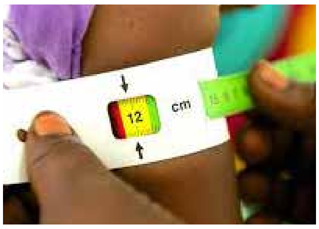
Use	The tape is used for rapid nutritional assessment in children between 6 and 60 months old, and in adults (primarily pregnant women). Mid-Upper Arm Circumference is a stable proxy measurement for nutritional status.<br>The tape is wrapped around the upper arm at the midpoint between the acromion and olecranon processes. The end of the tape is inserted through a slit in the tab-end, and adjusted for a close fit. The reading is taken from a window in the tab-end, at the printed alignment mark.	
Clinical or User Specifications	The child tape is colour-coded, showing the nutritional status as Good (green), marginal (yellow) or malnourished (red). The tape measures up to 265mm<br>The adult tape is not colour-coded, and measures up to 500mm.<br>Note that the tape should conform to the current WHO standard S0145620.	
Accessories or Related Consumables	None.	
Care	Standard cleaning and disinfection.	
Supplier Warranty		

Urine collection

TECHNICAL SPECIFICATIONS page 575 & 576

Catalogue Number	Item Name	Available on Transversal Contract	Contract Number or Code
PHC-E-151	Bedpan	No	
PHC-E-158	Urine jar or flask	No	

Description	Receptacles for urine collection.	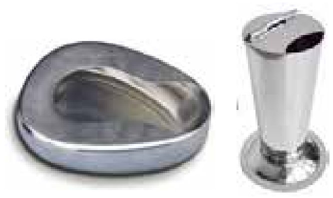
Use	Urine collection for testing purposes.	
Clinical or User Specifications	Standard bedpans are provided for use by females.<br>Urine jars or flasks are provided for use by men.	
Accessories or Related Consumables	None.	
Care	Standard cleaning and disinfection.<br>Autoclaveable.	
Supplier Warranty	2 years (excluding deliberate or accidental damage).	

Specialised

RESUSCITATION

Automated external defibrillator

TECHNICAL SPECIFICATIONS page 577

Catalogue Number	Item Name	Available on Transversal Contract	Contract Number or Code
PHC-E-133	Automated external defibrillator (AED)	RT 4	RT 4-05-072-A-ME

Description	A self-contained device which is used in the resuscitation of a patient who has suffered cardiac arrest. It prompts the user with visual and audible instructions.	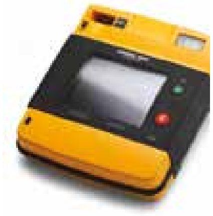
Use	In a suspected cardiac arrest, two electrodes are applied to the patient's chest. The unit determines whether there is any cardiac electrical activity, and whether to deliver an electric pulse to restart the heart. If unsuccessful, it tells the user to deliver another pulse (or pulses) at a higher energy charge. A display shows any detected cardiac electrical activity. It can be used as a temporary cardiac monitor, while stabilizing or transporting the patient, by means of a separate three-lead ECG cable.	
Clinical or User Specifications	There are separate adult and child electrodes. The unit detects the electrode size, and modifies the electrical pulse accordingly. The unit can deliver approximately 50 shocks at 150 Joules, or about 2 hours of monitoring time.	

Accessories or Related Consumables	One 3-lead ECG cable with pouch RT 4-05-073-A-ME.<br>One adult AED pacing pads set.<br>One paediatric AED pacing pads set.<br>Two rechargeable batteries RT 4-05-074-A-ME.<br>Battery charger.<br>One carrying case with shoulder strap for the case.<br>One operation manual.<br>One CD service manual.<br>One in-service video.<br>Replacement pads: adult PHC-C-174<br>Replacement pads: paediatric PHC-C-175
Care	The unit is very robust, but should be handled with care. Keep the unit clean.<br>The unit has a self-test feature, and should be tested preferably daily, but at least weekly. If the self-test fails, the supplier should be contacted immediately.<br>A second battery is provided, and the batteries should be swopped on a regular basis, to prolong battery life. The battery which is out of the unit must also be kept charged, in case of emergency need.<br>Order a replacement battery when indicated by the self-test.<br>The electrodes have an expiry date.<br>Order replacement electrodes before the expiry date.<br>Note that there is a 45-day lead time for delivery.
Supplier Warranty	The batteries have a 2-year guarantee, but will eventually fail, and need to be replaced.<br>The unit itself has an unconditional 2-year guarantee (excluding deliberate or accidental damage).<br>The pads have a 2-year guarantee shelf life.

Cardiac arrest board

TECHNICAL SPECIFICATIONS page 580

Catalogue Number	Item Name	Available on Transversal Contract	Contract Number or Code
PHC-E-134	Cardiac arrest board	No	

Description	Cardiac arrest board.	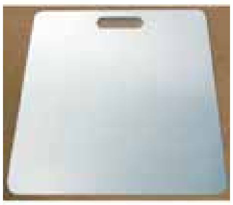
Use	A board which is slipped under the thorax of a collapsed patient, to provide a stable rigid platform against which to perform chest compressions during resuscitation.	
Clinical or User Specifications	The board is approximately 60cm by 40cm in size, can be made of rigid plastic or other rigid material, with a smooth finish to enable insertion under the chest of an unconscious patient. This is important to ensure the effectiveness of chest compression, if the patient happens to be on a soft surface such as a bed. Not required if patient is on a hard surface.<br>May have cut-outs to serve as hand grips.	
Accessories or Related Consumables		
Care	Keep on or near the emergency trolley (most emergency trolleys have storage hooks or shelves for this purpose).<br>Standard cleaning and disinfection.	
Supplier Warranty	2 years (excluding deliberate or accidental damage).	

Infant warmer: radiant

TECHNICAL SPECIFICATIONS page 581

Catalogue Number	Item Name	Available on Transversal Contract	Contract Number or Code
PHC-E-190	Infant warmer: radiant, mobile	RT2-2016	42191811-00001

Description	A unit comprising a nursing platform for neonates, with an overhead radiant warming system.	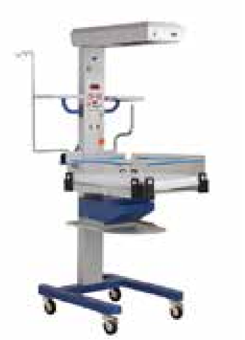
Use	The unit is used for the care and resuscitation of neonates in distress. It provides a thermo-neutral environment for the neonate, while facilitating open access (different from an incubator). Thermal stability is necessary, particularly for premature or Low Birth Weight neonates.	
Clinical or User Specifications	Microprocessor-controlled temperature maintenance. Fail-safe protection against over-heating.<br>Easy, one-touch controls.<br>Fixed distance from heater grid to mattress. Heat focused on area within the bassinet.<br>Bassinet can be tilted to Trendelenburg and Reverse-Trendelenburg positions.<br>Manual and automatic alarms, for over/under temperature, probe disconnection, power disconnection.<br>Temperature probe for precise monitoring.	
Accessories or Related Consumables	PHC-E-261 Infant warmer temperature probe.<br>PHC-E-260 Infant warmer replacement mattress.	
Care	This equipment must be purchased with a five-year maintenance plan. Service intervals as per supplier instructions. Supplier must provide on-site training as part of delivery and commissioning.	
Supplier Warranty	2 years (excluding deliberate or accidental damage).	

Infant warmer: mattress

NO TECHNICAL SPECIFICATIONS
NONE

Catalogue Number	Item Name	Available on Transversal Contract	Contract Number or Code
PHC-E-260	Infant warmer: replacement mattress	RT2-2016	42191814-00002

Description	Reusable temperature probe, used with radiant infant warmer.
Use	The probe is used for precision monitoring of the air temperature in the neonate's environment.
Clinical or User Specifications	Must monitor temperature with a precision of <0.2 °C, to maintain temperature in the thermo-neutral range.
Accessories or Related Consumables	
Care	Standard cleaning and disinfection.
Supplier Warranty	2 years (excluding deliberate or accidental damage).

Infant warmer: temperature probe

NO TECHNICAL SPECIFICATIONS NONE

Catalogue Number	Item Name	Available on Transversal Contract	Contract Number or Code
PHC-E-261	Infant warmer: temperature probe	RT2-2016	42191814-00001

Description	Reusable temperature probe, used with radiant infant warmer.
Use	The probe is used for precision monitoring of the air temperature in the neonate's environment.
Clinical or User Specifications	Must monitor temperature with a precision of <0.5 °C to maintain temperature in the thermo-neutral range.
Accessories or Related Consumables	
Care	Standard cleaning and disinfection.
Supplier Warranty	2 years (excluding deliberate or accidental damage).

Laryngoscope set

TECHNICAL SPECIFICATIONS page 583

Catalogue Number	Item Name	Available on Transversal Contract	Contract Number or Code
PHC-E-135	Laryngoscope set for PHC facilities	No	

Description	Detachable-blade hand-held fibre-optic laryngoscope set, for endotracheal intubation.	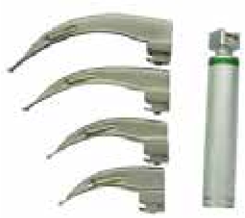
Use	This instrument set is used when it is necessary to insert an endotracheal tube, to secure a patient's airway.	
Clinical or User Specifications	Non-slip handle. Light source is built into the head of the handle. Bulb cannot unscrew and fall out during use. Fibre-optic light path built into each blade so that it cannot be removed or fall out. Blade is positively attached to handle in the closed position. Light turns on automatically when blade is extended into the operating position.<br>The set consists of: 1 adult handle; 1 paediatric handle (may be optional); paediatric blade sizes 0, 1, straight fibre optic (Miller pattern), and 1,2,3,4,5 curved fibre-optic(Macintosh pattern). batteries are housed in the handles.	
Accessories or Related Consumables	Spare bulb, spare set of batteries. Paediatric handle may be optional.	
Care	Normal care for stainless steel medical instruments.<br>Check instrument operation daily.<br>Remove and inspect batteries weekly.<br>Although batteries are non-corrosive, they may still leak after an extended period.<br>Replace bulb if instrument does not light up and batteries are good.	
Supplier Warranty	5-year guarantee on handles, blades, hook-on mechanism and switches. At least 2-year guarantee on light bulb (excluding deliberate or accidental damage).	

Monitor: multi-function vital signs, including SpO_2

TECHNICAL SPECIFICATIONS page 584

Catalogue Number	Item Name	Available on Transversal Contract	Contract Number or Code
PHC-E-136	Monitor: multi-function vital signs, including SpO_2	RT 4-2015	RT 4-05-030 ME

Description	This is an automated electronic device for monitoring vital signs in an emergency setting. The unit monitors pulse, blood pressure, SpO_2 and optionally temperature and the ECG. 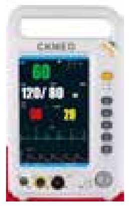
Use	The unit is typically used in a resuscitation situation, where continuous monitoring of vital signs is needed. It is usually mounted on the wall or on an emergency trolley.
Clinical or User Specifications	The unit operates automatically, once switched on and connected to the patient. Measurements are repeated at pre-set intervals – usually at a standard setting, which does not require changing under normal circumstances. Measurements are stored for later review.<br>The pulse can be indicated by an audible beep tone, so that the user is not constrained while performing resuscitation.<br>The unit has a built-in battery and charger, and can be detached from its normal mounting position, if required for continuous monitoring of a patient who is being transported.<br>The battery permits sustained monitoring in case of a power failure.<br>Supplier is responsible for delivery, commissioning (including mounting) and training.
Accessories or Related Consumables	Supplied with patient cables, sensors, power cable, cuffs (large adult, adult child, neonate, disposable self-adhesive monitoring pads.<br>ECG electrodes: disposable, self-adhesive, for resting ECG. PHC-C-212.
Care	Although the instrument specified has been designed for mobile use, it will be damaged by dropping or careless use. Do not unmount unless absolutely necessary.<br>The unit has a self-test routine on start-up – test daily. In case of failure, contact supplier immediately.<br>Clean with standard cleaning/anti-infective solutions. Cuffs and cuff covers are washable and disinfectable.<br>Check cables and air tubing for wear and damage. Order replacements if worn.<br>Keep plugged in at all times.
Maintenance	The unit must be calibrated at least annually, by technical personnel of the department or the supplier.<br>Internal battery must be replaced when prompted to do so by the battery indicator. Contact technical personnel of the department or the supplier.
Supplier Warranty	3 years on all parts (including cuffs and cuff covers) except battery (excluding deliberate or accidental damage).

Nebuliser

TECHNICAL SPECIFICATIONS page 587

Catalogue Number	Item Name	Available on Transversal Contract	Contract Number or Code
PHC-E-137	Nebuliser	No	

Description	Nebuliser.	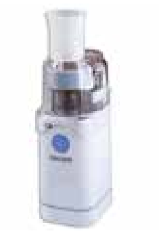
Use	An electrical device to generate a stream of aerosol mist for drug delivery for treatment of chronic or acute pulmonary disease.	
Clinical or User Specifications	The nebuliser is used to deliver moisturised air via a face mask. The nebulisation chamber converts medication into micron-sized particles, for delivery into the airways via the nebulised air stream.<br>Powered by 220V. mains electricity.	
Accessories or Related Consumables	Mask: oxygen (diluter type) adult PHC-C-108.<br>Mask: oxygen (diluter type) paediatric PHC-C-107.	
Care	Standard cleaning and disinfection.	
Supplier Warranty	2 years (excluding deliberate or accidental damage).	

Oxygen regulator with flow-meter

TECHNICAL SPECIFICATIONS page 588

Catalogue Number	Item Name	Available on Transversal Contract	Contract Number or Code
PHC-E-138	Oxygen regulator with flow-meter	No	

Description	Oxygen regulator with flow-meter.	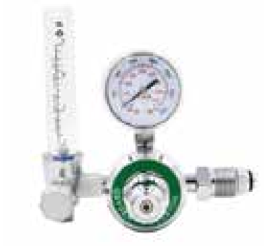
Use	This item controls the pressure at which oxygen is released from a cylinder, and also permits the flow rate of the released oxygen to be adjusted to clinical needs.<br>Connects directly to an oxygen cylinder, has connection point for oxygen mask or tubing.	
Clinical or User Specifications	The regulator has a standard bullnose fitting for connection to common sizes of oxygen cylinder.<br>The regulator has a pre-set delivery pressure.<br>An oxygen mask can be connected directly to the output of the flowmeter. Alternately, a length of oxygen tubing can be connected to the flowmeter, for a longer reach.<br>The flowmeter can be set to the required litres per minute.	
Accessories or Related Consumables	None.	
Care	Standard cleaning and disinfection.	
Supplier Warranty	5 years on needle valve, 10 years on valve body and flowmeter (excluding deliberate or accidental damage).	

Resuscitators: manual

TECHNICAL SPECIFICATIONS pages 589 to 592

Catalogue Number	Item Name	Available on Transversal Contract	Contract Number or Code
PHC-E-140	Resuscitator: infant, manual	RT 4	RT 4-05-026 ME
PHC-E-141	Resuscitator: child, manual	RT 4	RT 4-05-025 ME
PHC-E-142	Resuscitator: adult, manual	RT 4	RT 4-05-024 ME
PHC-E-143	PEEP valve	RT 4	RT 4-05-027 ME

Description	Set of manual resuscitators. 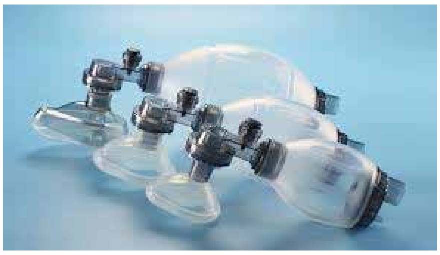
Use	For assisted ventilation of a collapsed patient.<br>The infant resuscitator is used to ventilate premature neonates and infants, <4kg.
Clinical or User Specifications	Can be operated with single hand. Self-re-expanding bag.<br>Inlet for supplementary oxygen.<br>Clear face masks. Can be used with PEEP valve, which has adjustable pressure.<br>Mask sizes 00,0 and 1 to be supplied with infant resuscitator.<br>Mask sizes 1, 2 and 3 to be supplied with child resuscitator.<br>Mask sizes 4 and 5 to be supplied with adult resuscitator.
Accessories or Related Consumables	Supplied in a soft carrying case made of durable, washable material with appropriate carrying straps.
Care	Standard cleaning and disinfecting.<br>PEEP valve is autoclaveable.
Supplier Warranty	2 years (excluding deliberate or accidental damage).

Suction machine: portable

TECHNICAL SPECIFICATIONS pages 593

Catalogue Number	Item Name	Available on Transversal Contract	Contract Number or Code
PHC-E-146	Suction machine: portable	RT 4-2015	RT 4-05-041 ME

Description	Suction machine: portable.	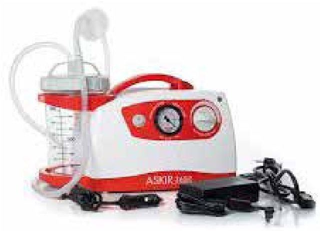
Use	To be used during resuscitation.<br>Suitable for PHC clinics.	
Clinical or User Specifications	Mobile suction device. Mains operated, with built-in battery and charger. 1 hour operation on full battery charge.<br>Castor-mounted. Two receiver jars (2l each).	
Accessories or Related Consumables	Supplied with re-usable, autoclaveable suction tube and cannula.<br>Filter: antibacterial. PHC-C-162.	
Care	Standard cleaning and disinfecting.<br>Suction tube and cannula must be cleaned and autoclaved after use.<br>Receiver jars and lids autoclaveable.<br>Replace hydrophobic bacterial filter after each use.	
Maintenance	Must be serviced annually by departmental or supplier technical personnel.	
Supplier Warranty	2 years (excluding deliberate or accidental damage).	

Suction machine: trolley type

TECHNICAL SPECIFICATIONS page 595

Catalogue Number	Item Name	Available on Transversal Contract	Contract Number or Code
PHC-E-144	Suction machine: tolley type	RT 2-2016	42171801-00002

Description	Suction, mobile.	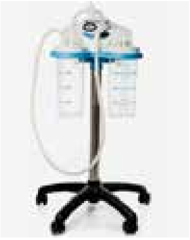
Use	To be used during resuscitation.<br>For use in CHCs	
Clinical or User Specifications	Suction device. Mains operated, with built-in re-chargeable battery and charger. 1 hour operation on full battery charge.<br>Mounted on a mobile stand with 4 castor wheels, two lockable. Two receiver jars (2l each).	
Accessories or Related Consumables	Supplied with re-usable, autoclaveable suction tube and cannula.<br>Filter: antibacterial. PHC-C-161.	
Care	Standard cleaning and disinfecting.<br>Receiver jars and lids autoclaveable.<br>Replace hydrophobic bacterial filter after each use.	
Maintenance	Must be serviced annually be departmental or supplier technical personnel.	
Supplier Warranty	2 years (excluding deliberate or accidental damage).	

Tape: Broselow

TECHNICAL SPECIFICATIONS pages 597

Catalogue Number	Item Name	Available on Transversal Contract	Contract Number or Code
PHC-E-128	Broselow tape	RT 4	RT 4-05-010 ME

Description	A printed tape, showing recommended dosages of resuscitation drugs and defibrillation energy for use in infants, where the weight is unknown.	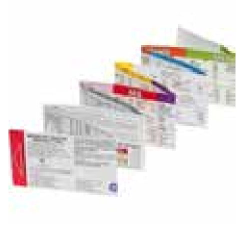
Use	Used in resuscitation settings, where there is no time to weigh the infant.	
Clinical or User Specifications	The tape is used to measure the length of the infant, and converts infant length into calculated weight, when there is no time to weigh the infant. The measured length on the tape will fall into a particular range. Each range is colour-coded, with subdivisions for larger or smaller infants within that length range.<br>Each colour – coded zone has recommended dosages of resuscitation drugs and defibrillation energy for use in infants of the calculated weight.<br>The use of the tape can be extended further by using pre-packed containers, matching each colour code, having all the equipment and drugs associated with that colour code (e.g. endotracheal tube size, drugs in correct mg/kg).	
Accessories or Related Consumables	Clear plastic storage pouch.	
Care	Standard cleaning and disinfection.<br>Note that most of these tapes are made of coated paper – Do not soak.	
Supplier Warranty	None.	

Tape: PAWPER and flipper chart

TECHNICAL SPECIFICATIONS page 598

Catalogue Number	Item Name	Available on Transversal Contract	Contract Number or Code
PHC-E-129	PAWPER Tape and chart	No	

Description	The Paediatric Advanced Weight Prediction in the Emergency Room (PAWPER) tape, is a printed tape, showing recommended dosages of resuscitation drugs and defibrillation energy for use in infants, where the weight is unknown.	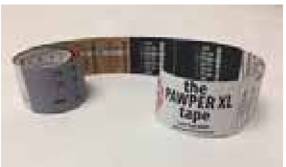
Use	Used in resuscitation settings, where there is no time to weigh the infant.	
Clinical or User Specifications	The tape is used to measure the length of the infant, and converts infant length into calculated weight, when there is no time to weigh the infant. The measured length on the tape will fall into a particular range. Each range is colour-coded, with subdivisions for larger or smaller infants within that length range.<br>Each colour – coded zone has recommended dosages of resuscitation drugs and defibrillation energy for use in infants of the calculated weight. These are show on the accompanying flipper chart.<br>The PAWPER tape formula for conversion of length to weight, has been shown in published studies to be more accurate than the Broselow Tape in the South African setting.	
Accessories or Related Consumables	Supplied as a set of four tapes and one flipper chart.	
Care	Standard cleaning and disinfection.<br>Note that most of these tapes are made of coated paper – Do not soak.	
Supplier Warranty	None.	

POINT OF CARE TESTING

TECHNICAL SPECIFICATIONS page 599

Glucometer

Catalogue Number	Item Name	Available on Transversal Contract	Contract Number or Code
PHC-E-149	Glucometer	No	

Description	Portable, handheld blood glucose meter.	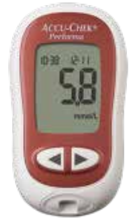
Use	For rapid measurement of glucose level from finger-prick sample.	
Clinical or User Specifications	Battery operated.<br>Measures blood glucose by reading the colour changes on the instrument's disposable test strip.<br>Records reading in memory.	
Accessories or Related Consumables	One pack of glucometer test strips PHC-C-181.<br>One pack of multipurpose lancets PHC-C-146.	
Care	Replace batteries if prompted to do so by the "battery low" indicator.<br>These types of devices have been shown to facilitate the transmission of blood-borne diseases in a multi-user environment. Follow manufacturer's cleaning instructions carefully.	
Supplier Warranty	2 years (excluding deliberate or accidental damage).	

Haemoglobinometer

TECHNICAL SPECIFICATIONS page 601

Catalogue Number	Item Name	Available on Transversal Contract	Contract Number or Code
PHC-E-152	Haemoglobinmeter	No	

Description	Electronic meter to measure blood haemoglobin level. 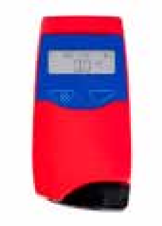
Use	For rapid measurement of haemoglobin level from finger-prick sample.
Clinical or User Specifications	Battery-powered handheld meter (rechargeable or replaceable batteries, depending on model procured).<br>Uses either a micro-cuvette or a test strip (depending on model procured).<br>Capillary blood specimen (using standard lancets).<br>Display reads haemoglobin level out directly.
Accessories or Related Consumables	Lancets, multipurpose PHC-C-146.<br>Hb meter test strips PHC-C-182.<br>Hb meter microcuvette PHC-C-183. (Depending on the model procured.)
Care	Replace/recharge batteries if prompted to do so by the "battery low" indicator.<br>These types of devices have been shown to facilitate the transmission of blood-borne diseases in a multi-user environment. Follow manufacturer's cleaning instructions carefully.<br>Biosafety waste disposal containers are mandatory.
Maintenance	This unit must be recalibrated at least annually. Contact departmental or supplier technical personnel.
Supplier Warranty	2-year shelf life.

STERILISATION

Autoclave: tabletop

TECHNICAL SPECIFICATIONS page 601

Catalogue Number	Item Name	Available on Transversal Contract	Contract Number or Code
PHC-E-157	Autoclave: tabletop	No	

Description	A medium-sized tabletop automatic autoclave. The autoclave operates on the following general cycle: while water is being heated into steam, air is sucked out of the sterilisation chamber. Steam is then introduced into the chamber under pressure (to keep the temperature above boiling point). The steam penetrates textiles and wrappings because of the alternating vacuum and pressure. As steam condenses, it forms moisture and gives off heat, which is very effective at heat transfer to the objects to be sterilised. The temperature and pressure are maintained for a pre-determined period. Thereafter, the steam is released, and there is a drying (and cooling) cycle. The cycles vary in time and temperature, depending on the load to be sterilised. A display panel shows temperature, pressure, type of cycle, stage of cycle, and any alarm conditions.	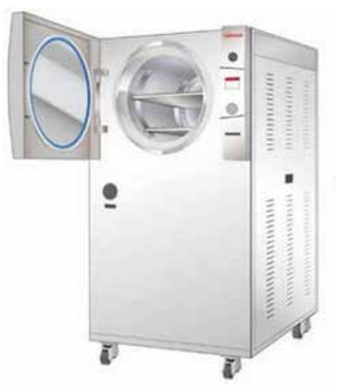
Use	This size of autoclave is suitable for a larger clinic, or a Community Health Centre with an MOU.<br>This autoclave can sterilise all types of loads. Although it has numerous safety features, care is still necessary because of the steam and very high temperatures generated.<br>Depending on the model selected, de-ionised water may be required – follow the manufacturer's instructions carefully.<br>The autoclave requires periodic testing for temperature and pressure and leakage. There are specific test protocols to carry these out, and the machine has pre-programmed test cycles. A prion cycle is available if required by the SOP<br>The tests should be carried out regularly as per SOP, to maintain confidence in the sterilisation process.<br>The following are general guidelines for use:<br>a. Clean the drain strainer before loading.<br>b. Do not load non-autoclaveable items. Place items so that the steam can get to them.<br>c. Do not place autoclaveable glass directly in contact with metal surfaces – use a heat-resistant plastic tray.<br>d. The autoclave uses water and power, irrespective of load. Therefore utilise the full space.<br>e. Clean and dry items before placing in the autoclave, especially if in a pack.<br>f. Arrange loads according to cycle type, i.e. do not mix liquids and instruments, as they require different cycles.	

	g. The autoclave has an interlock, to prevent the door from opening while it is still under pressure and the temperature is high. ONCE THE DOOR CAN BE OPENED, CAUTION IS STILL REQUIRED – THE AIR AND ITEMS INSIDE MAY STILL BE EXTREMELY HOT.<br>h. Wait at least 30 seconds before reaching, or looking into the autoclave, after opening the door.<br>i. Wait 5 minutes before removing the load.<br>j. Use personal protection when removing items from the autoclave.<br>The autoclave can store the data for the last 20 cycles. The data can be downloaded to a computer for review or filing. If the optional printer is purchased, the data can be printed directly.
Clinical or User Specifications	Pre-set programmes for the following:<br>Wrapped dry goods (Linen, Gowns) – 134°C<br>Wrapped or pouched hard goods (Instruments – solid and hollow) – 134°C<br>Unwrapped hard goods – 134°C<br>Plastics – 105°C.<br>Fluids or rubber goods –121°C<br>Flash cycle – 134°C<br>Porous items.<br>Alarms for all types of malfunction: temperature, pressure, water supply, interrupted cycle.
Accessories or Related Consumables	Spare door seal.<br>Perforated sterilising trays (at least three), long enough to house maternity instruments.<br>Pack of Bowie-Dick test packs. PHC-C-213<br>Helix test kit with pack of strips. PHC-C-214.
Care	Observe water quality requirements.<br>Ensure drainage after daily use.<br>**Testing**<br>This should be carried out at least weekly, but more frequently if the SOP requires it.<br>**Bowie-Dick test:** a test pack (and nothing else) is placed over the drain outlet, and the Bowie-Dick cycle is run on the autoclave. If the thermochromatic paper at the core of the pack does not change colour completely, the test has failed. Maintenance personnel should be summoned.<br>**Leak test:** run this cycle with an empty autoclave. The cycle develops the usual operating vacuum, and then monitors the vacuum over a period of 15 minutes. If there is a leak, the vacuum will decrease more rapidly than allowed. If this test fails, summon maintenance personnel, and change the door seal.<br>**Helix test:** uses the same cycle as the Bowie-dick, but a test strip is placed inside a deep, catheter-like receptacle. This tests steam penetration inside deep hollow instruments, and should be carried out if such instruments are used in the facility. If this test fails, summon maintenance personnel.
Maintenance	The autoclave is supplied with a full maintenance contract, for 5 years. The supplier will provide a schedule of preventative and planned maintenance.
Supplier Warranty	2 year guarantee (excluding deliberate or accidental damage)<br>Vacuum pump guaranteed for 5 years<br>Heating elements guaranteed for 10 years

Instrument steriliser: desktop

TECHNICAL SPECIFICATIONS page 605

Catalogue Number	Item Name	Available on Transversal Contract	Contract Number or Code
PHC-E-112	Instrument steriliser: desktop	No	

Description	Desktop instrument sterilising autoclave.<br>For smaller PHC facilities.	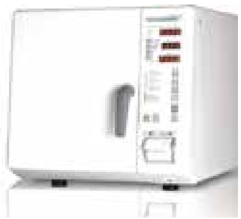
Use	Primary use is to sterilise dental instruments and other small instruments at PHC level.	
Clinical or User Specifications	22l capacity.<br>Can autoclave solid, porous, hollow, packed and unpacked items.<br>Prion cycle available.<br>Simple 2-step start.<br>Automatic stop at end of cycle.<br>Does not affect the temper of steel instruments.<br>Negative pressure drying cycle.<br>Self-test, checks water quality.	
Accessories or Related Consumables	Spare door seal.<br>Perforated sterilising trays (at least three), long enough to house maternity instruments.<br>Pack of Bowie-Dick test packs. PHC-C-213<br>Helix test kit with pack of strips. PHC-C-214.<br>Observe water quality requirements.<br>Ensure drainage after daily use.	

	**Testing**<br>This should be carried out at least weekly, but more frequently if the SOP requires it.<br>**Bowie-Dick test:** a test pack (and nothing else) is placed over the drain outlet, and the Bowie-Dick cycle is run on the autoclave. If the thermochromatic paper at the core of the pack does not change colour completely, the test has failed. Maintenance personnel should be summoned.<br>**Leak test:** run this cycle with an empty autoclave. The cycle develops the usual operating vacuum, and then monitors the vacuum over a period of 15 minutes. If there is a leak, the vacuum will decrease more rapidly than allowed. If this test fails, summon maintenance personnel, and change the door seal.<br>**Helix test:** uses the same cycle as the Bowie-dick, but a test strip is placed inside a deep, catheter-like receptacle. This tests steam penetration inside deep hollow instruments, and should be carried out if such instruments are used in the facility. If this test fails, summon maintenance personnel.<br>The autoclave is supplied with a full maintenance contract, for 5 years. The supplier will provide a schedule of preventative and planned maintenance.
Care	Report immediately if unit malfunctions but power is available.<br>Standard cleaning and disinfection.
Supplier Warranty	5 years complete service and maintenance contract.

ORAL HEALTH

- This is a compendium of equipment required to set up a new dental surgery. Some of the items listed, may not be suitable as replacements for older, existing equipment, because of compatibility issues. Replacements should be found on the Treasury term contract. A suitably qualified professional should be consulted before orders are finalised.
- For certain items, the final choice depends on environmental factors, such as availability of power and water. Alternatives are indicated, but a suitably qualified professional should be consulted before orders are finalised.
- The dental units referenced here, are supplied complete with handpieces. Handpieces are also listed separately for replacement purposes, but the choice has been limited to non-fibre-optic, stainless steel body types. Other types will be available on the term contract.
- In all cases, reference should be made to the Treasury term contract in force at the time.

Amalgam separator: 2 surgeries

TECHNICAL SPECIFICATIONS page 608

Catalogue Number	Item Name	Available on Transversal Contract	Contract Number or Code
PHC-E-164	Amalgam separator: 2 surgeries	In preparation	

Description	A fixed-installation amalgam separator.
Use	Extracts amalgam from waste water from the dental unit and cuspidor, to prevent mercury from contaminating the local water supply.
Clinical or User Specifications	Automatic switch on when waste water is available from treatment unit.<br>Can connect to more than one treatment unit.<br>Separated amalgam is available for recycling.
Accessories or Related Consumables	
Care	Standard cleaning and disinfection.
Maintenance	This type of unit is supplied with a maintenance contract. Service intervals as per manufacturer's instructions.
Supplier Warranty	2 years (excluding deliberate or accidental damage).

TECHNICAL SPECIFICATIONS page 609

Amalgamator: digital

Catalogue Number	Item Name	Available on Transversal Contract	Contract Number or Code
PHC-E-166	Amalgamator: digital	In preparation	

Description	An electronic unit for the preparation of amalgam.
Use	For thorough mixing of amalgam or glass ionomer prior to use.
Clinical or User Specifications	Note: this unit differs from the older analogue amalgamators.<br>Automatic timer.<br>Must accept mercury and glass ionomer capsules.
Accessories or Related Consumables	Replaceable capsule holder.
Care	Standard cleaning and disinfection.
Supplier Warranty	2 years (excluding deliberate or accidental damage).

Autoclave: cassette-type, desktop

TECHNICAL SPECIFICATIONS page 610

Catalogue Number	Item Name	Available on Transversal Contract	Contract Number or Code
PHC-E-167	Desktop Autoclave S-Type for Dental Instruments	In preparation	

Description	A S-type, cassette loading desktop autoclave.	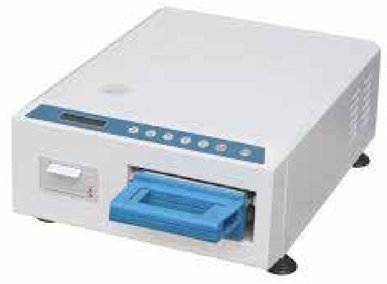
Use	For sterilising wrapped, unwrapped or hollow dental instruments. Note that this autoclave is almost exclusively for dental instruments, is transportable, and can be used by outreach teams. Larger autoclaves for shared use are to be found under Sterilisation.	
Clinical or User Specifications	Must be a cassette type loading autoclave.<br>Reservoir for distilled water.<br>Instruments are placed wrapped or unwrapped into the cassette, which is inserted into the steriliser.<br>Drying cycle for wrapped instruments.	
Accessories or Related Consumables	Bowie-Dick test pack PHC-C-213. Helix test pack PHC-C-214.<br>Cassette and cassette accessories.	
Care	Must use distilled water. Water distiller must be procured with this unit if not already available.<br>Bowie-Dick, Helix or equivalent tests to be performed as per SOP.	
Maintenance	This type of unit is supplied with a maintenance contract. Service intervals as per manufacturer's instructions.	
Supplier Warranty	2 years (excluding deliberate or accidental damage).	

Compressor: 2-4 surgeries

TECHNICAL SPECIFICATIONS page 611

Catalogue Number	Item Name	Available on Transversal Contract	Contract Number or Code
PHC-E-168	Compressor: 2-4 surgeries	In preparation	

Description	A fixed-installation, oil-free air compressor.<br>This is the preferred option even for a single surgery, as it permits future expansion.
Use	Mounted externally in a weather-proof enclosure. Serves 2-4 dental surgeries.
Clinical or User Specifications	Quiet operation (maximum 60 dB).<br>Oil-free, self-draining.<br>Air filtration system 10μ particles.<br>Storage capacity 35-75l.
Accessories or Related Consumables	
Care	Service intervals as per supplier's instructions.
Maintenance	This type of unit is supplied with a maintenance contract. Service intervals as per manufacturer's instructions.
Supplier Warranty	2 years (excluding deliberate or accidental damage).

Cuspidor/Spittoon/Water Unit

TECHNICAL SPECIFICATIONS page 612

Catalogue Number	Item Name	Available on Transversal Contract	Contract Number or Code
PHC-E-169	Cuspidor/Spittoon/Water Unit	In preparation	

Description	A chairside water and spittoon system.
Use	Unit supplies warm water for mouth rinsing, and provides a bowl into which water may be ejected. Attachment for saliva ejector.
Clinical or User Specifications	Warm water faucet, with receptacle holder.<br>Enamel bowl with particle trap.<br>Suction connector for saliva ejector.
Accessories or Related Consumables	Autoclaveable HVE cannulas to be supplied.
Care	Standard cleaning and disinfection.<br>Water quality should be checked according to SOP.
Maintenance	This type of unit is supplied with a maintenance contract. Service intervals as per manufacturer's instructions.
Supplier Warranty	5 years (excluding deliberate or accidental damage).

Delivery system: mobile, with hand pieces, non-fibre-optic

TECHNICAL SPECIFICATIONS page 613

Catalogue Number	Item Name	Available on Transversal Contract	Contract Number or Code
PHC-E-170	Delivery system: mobile, with hand pieces, non-fibre-optic	In preparation.	

Description	A self-contained unit, consisting of a cabinet on castors housing a compressor, cleanwater system, hand piece outlets, and hand pieces.
Use	Used to deliver services where there is no central compressor (if there is a central compressor, select delivery system: fixed). Not compatible with fibre-optic handpieces.
Clinical or User Specifications	Cabinet on castors.<br>Hand piece outlets (4 to 6 holes).<br>3 in 1 syringe.<br>Straight hand piece.<br>Contra angle hand piece.<br>High speed hand piece.<br>Air motor.<br>Compressor.<br>Cleanwater system.
Accessories or Related Consumables	Note that a curing light and scaler have not been specified for this unit, and must be ordered separately.
Care	Standard cleaning and disinfection.
Maintenance	This type of unit is supplied with a maintenance contract. Service intervals as per manufacturer's instructions.
Supplier Warranty	5 years (excluding deliberate or accidental damage).

Delivery system: fixed, with hand pieces, non-fibre-optic

TECHNICAL SPECIFICATIONS page 614

Catalogue Number	Item Name	Available on Transversal Contract	Contract Number or Code
PHC-E-171	Delivery system: fixed, with hand pieces, non-fibre-optic	In preparation	

Description	A Dental delivery system, complete with tubing, spittoon, scaler, clean water system and curing light.
Use	This is the unit of choice when there is a central compressor and suction system. Otherwise, choose the mobile delivery system. Not compatible with fibre-optic hand pieces.
Clinical or User Specifications	Hand piece outlets (4 to 6 holes) – self-activating.<br>3 in 1 syringe.<br>Control block must have 4-chair position controls (including reset button).<br>The unit must be connected to the cuspidor/ spittoon/ water unit.<br>Piezo-electric scaler and curing light included.
Accessories or Related Consumables	
Care	Standard cleaning and disinfection.
Maintenance	This type of unit is supplied with a maintenance contract. Service intervals as per manufacturer's instructions.
Supplier Warranty	5 years (excluding deliberate or accidental damage).

Dental chair: basic

TECHNICAL SPECIFICATIONS page 616

Catalogue Number	Item Name	Available on Transversal Contract	Contract Number or Code
PHC-E-172	Dental chair: basic	In preparation	

Description	Basic dental chair, suitable for a Primary Health Care facility.
Use	For positioning the patient for examinations and operative procedures.
Clinical or User Specifications	Fall range of movement in reclining, height, tilt (Trendelenburg), swivel.<br>Adjustable backrest.<br>Removable upholstery – easily cleanable.<br>Plastic shoe protector – replaceable.<br>Programmable.<br>Foot controls.
Accessories or Related Consumables	
Care	Standard cleaning and disinfection.
Maintenance	This type of unit is supplied with a maintenance contract. Service intervals as per manufacturer's instructions.
Supplier Warranty	5 years (excluding deliberate or accidental damage).

Dental chair: portable

TECHNICAL SPECIFICATIONS page 617

Catalogue Number	Item Name	Available on Transversal Contract	Contract Number or Code
PHC-E-173	Dental chair: portable	In preparation	

Description	A lightweight, portable dental chair, which folds into a backpack for transportation.
Use	For use by outreach teams.
Clinical or User Specifications	Maximum weight 12,5kg.<br>Height adjustable from 36cm to 60cm.<br>Must support 200kg without deflection.
Accessories or Related Consumables	Backpack. Compatible portable dental light, spit funnel and tray.
Care	Standard cleaning and disinfection.
Maintenance	This type of unit is supplied with a maintenance contract. Service intervals as per manufacturer's instructions.
Supplier Warranty	2 years (excluding deliberate or accidental damage).

Dental curing light

TECHNICAL SPECIFICATIONS page 618 & 618

Catalogue Number	Item Name	Available on Transversal Contract	Contract Number or Code
PHC-E-174	Dental curing light: corded	In preparation	
PHC-E-175	Dental curing light: cordless	In preparation	

Description	Handheld light source for curing resin based composites and glass ionomers used in dental restorations.
Use	The light probe is held against the composite once the cavity has been filled, to harden the resin. Different compounds and different shades require exposure to the light for different periods of time.
Clinical or User Specifications	Built-in selectable exposure timer.<br>In cordless models, audio-visual warning when battery charge is dropping below adequate levels to deliver stated irradiance. Auto-shutdown when power too low.
Accessories or Related Consumables	All necessary curing probes/tips.<br>Cordless model must be supplied with charger (complete with power cord).
Care	Standard cleaning and disinfection.<br>Care must be taken not to apply the light for longer than necessary – enough heat can be generated to cause intra-oral burns.
Supplier Warranty	2 years (excluding deliberate or accidental damage).

Dental handpieces: non-fibre-optic

TECHNICAL SPECIFICATIONS page 619

Catalogue Number	Item Name	Available on Transversal Contract	Contract Number or Code
PHC-E-176	Turbine: full range	In preparation	
PHC-E-177	Hand-piece motor: slow	In preparation	
PHC-E-178	LED straight handpiece: for micro motor	In preparation	
PHC-E-179	LED contra-angle handpiece: for micromotor	In preparation	
PHC-E-180	LED Prophylaxis handpiece: for micromotor	In preparation	

Description	Range of handpieces.
Use	Only required for replacement purposes, or in facilities where the items were not supplied. The new dental service units specified, are fully equipped with handpieces at time of delivery.
Clinical or User Specifications	Note that only non-fibre-optic types with stainless steel bodies are listed here. Other types (fibre-optic, titanium body) can be referenced from the term contract, if required for compatibility purposes.
Accessories or Related Consumables	
Care	Autoclaveable.
Supplier Warranty	2 years (excluding deliberate or accidental damage).

Dental light: LED

TECHNICAL SPECIFICATIONS page 622

Catalogue Number	Item Name	Available on Transversal Contract	Contract Number or Code
PHC-E-184	Dental light: LED	In preparation	

Description	Operator light for work field illumination.
Use	Mounted either on the dental chair, or on the ceiling.
Clinical or User Specifications	LED bulb.<br>Articulated arm can be adjusted in all three axes, to obtain optimal position.<br>Articulated arm will hold the desired position without slippage.<br>Touch sensor to switch on /off and adjust intensity.<br>Shatterproof shield.
Accessories or Related Consumables	All mounting hardware to be supplied by the supplier.<br>1 spare bulb.
Care	Optimal location to be determined in consultation with the supplier, the end-user, and provincial infrastructure personnel.<br>Standard cleaning and disinfection.
Maintenance	This type of unit is supplied with a maintenance contract. Service intervals as per manufacturer's instructions.
Supplier Warranty	5 years (excluding deliberate or accidental damage).

Dental scaler: air

TECHNICAL SPECIFICATIONS page 623

Catalogue Number	Item Name	Available on Transversal Contract	Contract Number or Code
PHC-E-185	Dental scaler: air	In preparation	

Description	Air-powered scaler for mounting in the dental delivery unit.
Use	For scaling of teeth. Needs to be ordered separately if the mobile dental service unit is selected (fixed unit is specified with a piezo scaler).
Clinical or User Specifications	Handpiece must be washable and autoclaveable.
Accessories or Related Consumables	Supplied with at least three scaling tips and wrench, and all other accessories.
Care	Standard cleaning and disinfection (autoclave).
Supplier Warranty	2 years (excluding deliberate or accidental damage).

Suction: dry, 2-3 surgeries

TECHNICAL SPECIFICATIONS page 624

Catalogue Number	Item Name	Available on Transversal Contract	Contract Number or Code
PHC-E-186	Suction: dry, 2-3 surgeries	In preparation	

Description	A fixed installation centralised dry aspiration plant for serving 2-4 surgeries.
Use	To provide suction to the various items of equipment that require it. This is the type generally used, when water scarcity prevails.
Clinical or User Specifications	Flow rate at least 820l/minute.
Accessories or Related Consumables	
Care	
Maintenance	This type of unit is supplied with a maintenance contract. Service intervals as per manufacturer's instructions.
Supplier Warranty	2 years (excluding deliberate or accidental damage).

Suction: dry, mobile

TECHNICAL SPECIFICATIONS page 625

Catalogue Number	Item Name	Available on Transversal Contract	Contract Number or Code
PHC-E-187	Suction: dry, mobile	In preparation	

Description	Mobile suction unit on castors.
Use	For use in a single surgery where the cost of a multi-surgery unit is not warranted. Suitable for use where water supply is restricted.
Clinical or User Specifications	Canister can hold at least 4l of aspirate. Must be emptied manually.
Accessories or Related Consumables	
Care	
Maintenance	This type of unit is supplied with a maintenance contract. Service intervals as per manufacturer's instructions.
Supplier Warranty	2 years (excluding deliberate or accidental damage).

Suction: wet, 2-4 surgeries

TECHNICAL SPECIFICATIONS page 626

Catalogue Number	Item Name	Available on Transversal Contract	Contract Number or Code
PHC-E-188	Suction: wet, 2-4 surgeries	In preparation	

Description	A fixed installation centralised wet aspiration plant for serving 2-4 surgeries.
Use	To provide suction to the various items of equipment that require it. This is the type generally used, when water availability is not a problem.
Clinical or User Specifications	Can serve more than one surgery.
Accessories or Related Consumables	
Care	
Maintenance	This type of unit is supplied with a maintenance contract. Service intervals as per manufacturer's instructions.
Supplier Warranty	2 years (excluding deliberate or accidental damage).

Ultrasonic cleaner

TECHNICAL SPECIFICATIONS page 627

Catalogue Number	Item Name	Available on Transversal Contract	Contract Number or Code
PHC-E-189	Ultrasonic cleaner	In preparation	

Description	Electronic ultrasonic instrument bath.
Use	Cleaning of dental instruments prior to sterilisation.
Clinical or User Specifications	Tank size minimum 4,5l.
Accessories or Related Consumables	Instrument basket. Optional extra basket, beaker, bur tray.
Care	Standard cleaning and disinfection.
Maintenance	This unit may require validation of cleaning efficiency at intervals stipulated by the manufacturer.
Supplier Warranty	1 year (excluding deliberate or accidental damage).

Water distillation

TECHNICAL SPECIFICATIONS page 628

Catalogue Number	Item Name	Available on Transversal Contract	Contract Number or Code
PHC-E-191	Water distillation unit	In preparation	

Description	A desktop water distillation unit.
Use	Produce a supply of distilled water for dental autoclave.
Clinical or User Specifications	Must be able to supply at least 4l of distilled water to supply different types of autoclaves.
Accessories or Related Consumables	
Care	Standard cleaning and disinfection.
Supplier Warranty	1 year (excluding deliberate or accidental damage)

X-ray Apron

NO TECHNICAL SPECIFICATIONS
NONE

Catalogue Number	Item Name	Available on Transversal Contract	Contract Number or Code
PHC-C-181	X-ray Apron: child	RT21-2015	RT21-04-015
PHC-C-182	X-ray Apron: adult	RT21-2015	RT21-04-018

Description	Radiation Protective Apron. 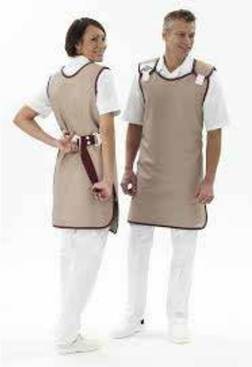
Use	Shielding of patients during dental x-ray procedures.
Clinical or User Specifications	Double sided aprons, 0.25mm lead equivalent.<br>Covering to be waterproof, stain-resistant.<br>Lead equivalent must be clearly labelled on the outside of each apron.<br>RT21-04-015: size Small.<br>RT21-04-018 size X-large.
Accessories or Related Consumables	None.
Care	
Supplier Warranty	1 year (excluding deliberate or accidental damage).

X-ray: wall-mounted

TECHNICAL SPECIFICATIONS page 629

Catalogue Number	Item Name	Available on Transversal Contract	Contract Number or Code
PHC-E-192	X-ray: wall-mounted	In preparation	

Description	Wall-mounted intra-oral X-ray unit.
Use	Capture analogue or digital images of oral anatomy. In a new surgery, analogue images are not an option (require photographic film, chemicals and processing equipment).
Clinical or User Specifications	Wall-mounted, usually behind the head end of the dental chair.<br>May require a long arm option to reach the patient while the latter is sitting in the dental chair.<br>Computerised timer. Selectable programmes with automatic setting of time and intensity.<br>Power supply built into unit.
Accessories or Related Consumables	Remote control – corded or cordless.<br>Note: If the unit on term contract does not include a digital oral imaging plate, the latter should be ordered at the same time as the X-ray unit.
Care	Standard cleaning and disinfection.
Maintenance	This type of unit is supplied with a maintenance contract. Service intervals as per manufacturer's instructions.
Supplier Warranty	10 years on arm (excluding deliberate or accidental damage).<br>5 years on tube.

X-ray: Digital oral imaging plate with a computer

TECHNICAL SPECIFICATIONS page 630

Catalogue Number	Item Name	Available on Transversal Contract	Contract Number or Code
PHC-E-193	X-ray: Digital oral imaging plate with a computer	In preparation	

Description	A system utilising radiation-sensitive plates to acquire digital X-ray images.
Use	Phosphor-coated plates of different sizes are inserted intra-orally instead of photographic film. The plates are placed in a processor post-exposure to the X-rays. The processor scans the plates, and produces a digital image, which can be viewed on a PC. One unit can be networked to computers in several surgeries.
Clinical or User Specifications	A selection of intra-oral plates, as specified in the term contract.<br>Single-use hygiene bags for each plate size.<br>Note: Compatible laptop/PC must be procured from existing term contracts.
Accessories or Related Consumables	Storage box or rack for the plates.<br>Hygiene bags 100 per plate size.<br>Image handling and dsiplay software.<br>All connectivity requirements between unit and PC(s).
Care	Observe aseptic precautions when transferring exposed plate from patient's mouth to processing unit (hygiene bag to be removed as per manufacturer's instructions).
Supplier Warranty	2 years (excluding deliberate or accidental damage).

REHABILITATION

Physical Therapy

Aluminium crutches

NO TECHNICAL SPECIFICATIONS
NONE

Catalogue Number	Item Name	Available on Transversal Contract	Contract Number or Code
PHC-E-212	Aluminium crutches: 1 pair adult	No	
PHC-E-213	Aluminium crutches: 1 pair paediatric	No	

Description	Aluminium adjustable elbow crutches in adult and paediatric sizes. 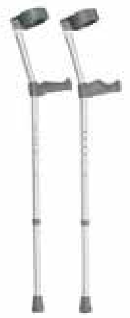
Use	For training patients before prescribing.
Clinical or User Specifications	Handle and cuff is a one- piece design with soft handle, plastic, moulded, vinyl coated. Adjustability in shaft utilizes double click mechanism.<br>Adult: Height from handle to ground (470mm-650mm) Extra length available. Height from handle to ground (470mm-650mm). Maximum body weight 120kg. Supplied as pair. Ferrules and cuffs available separately.<br>Paediatric and Youth: Single adjustable Paediatric crutches. Sold per pair. Available in 2 height options- Paediatric and youth.<br>Ferrules must have a thick base and a metal washer to help prevent splitting. Easy to attach and come in 22mm and 24mm.<br>Must meet SABS standards. Samples of crutches and ferrules must be issued in advance of award of tender to test durability of each in rough terrain.<br>Must comply with CSK 112-1973.
Accessories	None.
Care	Ensure that all moveable parts are working and secure before use.
Supplier Warranty	1 year (excluding deliberate or accidental damage).

Balance pad

NO TECHNICAL SPECIFICATIONS
NONE

Catalogue Number	Item Name	Available on Transversal Contract	Contract Number or Code
PHC-E-214	Balance pad	No	

Description	Pillow-shaped thick foam mat approximately 50cm x 41cm x 6cm used for assessing and treating balance reactions and postural control. 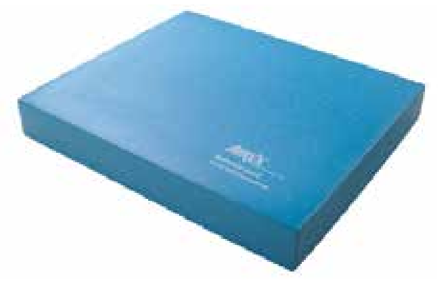
Use	To retrain balance in geriatrics, sports, orthopaedic, neuromusculoskeletal and neuro patients.
Clinical or User Specifications	Closed-cell foam pad. Impervious to water, easy to clean and long-lasting. Travelling teams could consider the Balance Air Cushion instead of the Balance pad.
Accessories	None.
Care	Remove shoes before exercising on the pad, as this may damage the pad surface. Do not place heavy objects or objects with sharp edges on top of the pad. Do not expose pad to high temperatures.
Supplier Warranty	2 years (excluding deliberate or accidental damage).<br>Expected service life 5 years.

Basin: portable, large

NO TECHNICAL SPECIFICATIONS
NONE

Catalogue Number	Item Name	Available on Transversal Contract	Contract Number or Code
PHC-E-215	Basin: portable, large	No	

Description	Enamel dish 50cm diameter.	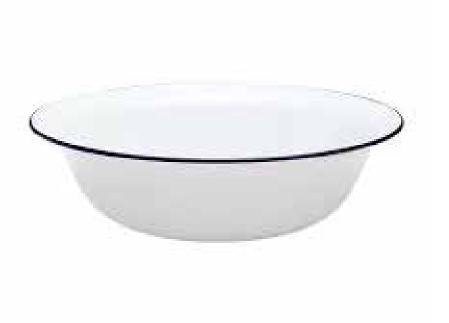
Use	Used in a variety of settings including after POP removal, for septic hands and for foot care.	
Clinical or User Specifications	Should be of sufficient depth to immerse a foot up to an ankle above the malleoli.	
Accessories	None.	
Care	should be autoclaveable.	
Supplier Warranty	N/A.	

Bobath ball

NO TECHNICAL SPECIFICATIONS NONE

Catalogue Number	Item Name	Available on Transversal Contract	Contract Number or Code
PHC-E-216	Bobath ball: 85cm	No	
PHC-E-217	Bobath ball: 65cm	No	
PHC-E-218	Bobath ball: 45cm	No	

Description	Large PVC inflatable ball. Pressure can be regulated by inflating or deflating the ball.	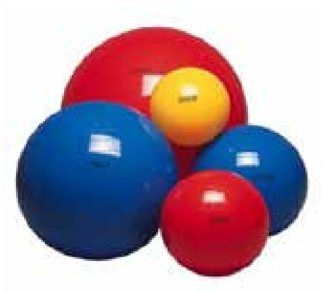
Use	For stimulation of afferent pathways to the brain, eliciting postural reactions, evaluating and training balance and co-ordination and rehabilitating core muscles in a variety of patients.	
Clinical or User Specifications	Dense PVC, extremely durable, easy to clean and antibacterial. Anti-burst.<br>Three sizes: 85cm diameter, 65cm diameter, 45cm diameter.<br>Maximum weight-bearing 150kg.	
Accessories	Hand pump and spare plugs.	
Care	Avoid solvents and bleach. Ball must be placed and stored on a surface without abrasive edges or puncturing may happen. Clients should be appropriately assessed and selected for this equipment. Adequate supervision and appropriate exercise prescription is required to prevent falls. Maximum weight must be respected.	
Supplier Warranty	1 year (excluding deliberate or accidental damage).	
Note to Procurement	Balls should be procured in sets of three sizes. One hand pump per set is sufficient, but balls should have their own spare plugs.<br>Expected service life 5 years.	

Bobath roller

NO TECHNICAL SPECIFICATIONS
NONE

Catalogue Number	Item Name	Available on Transversal Contract	Contract Number or Code
PHC-E-219	Bobath roller: 50cm	No	
PHC-E-220	Bobath roller: 30cm	No	

Description	Foam roller covered in tarpaulin/imitation leather.	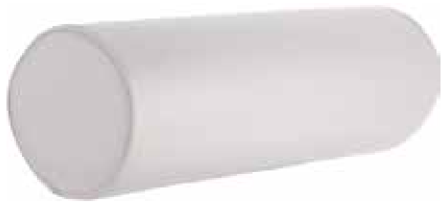
Use	For stimulating postural reactions and rehabilitating core muscles in a variety of patients.	
Clinical or User Specifications	Roller should be light but able to withstand 200kg. The core is made from very firm foam. The outer layer should be antibacterial plastic (100% polyester or PVC) which is easy to wipe and clean.<br>Large: length between 120 and 150cm, diameter 50cm.<br>Small: length between 90 and 120cm, diameter 30cm.<br>Maximum weight of roller 5kg.<br>Weight-bearing capacity 200kg.<br>The roller should be able to withstand weights and knocks usually sustained in a clinical and transportation setting without deformation.	
Accessories	None.	
Care	Do not place roller underneath heavy or sharp objects as this may damage the shape or covering. Clients should be appropriately assessed and selected for this equipment. Adequate supervision and appropriate exercise prescription is required to prevent falls. Maximum weight must be respected.	
Supplier Warranty	1 year (excluding deliberate or accidental damage).	
Note to Procurement	1 year warranty required.<br>Purchase in sets of 2 (large and small).<br>Expected service life 5 years.	

Construction play equipment

NO TECHNICAL SPECIFICATIONS NONE

Catalogue Number	Item Name	Available on Transversal Contract	Contract Number or Code
PHC-E-221	Construction play equipment	No	

Description	Large Lego set with minimum 100 building bricks of assorted sizes/shapes and colours, set of Duplo building bricks, minimum 30 pieces of assorted sizes/ shapes and colours; set of minimum 40 pieces painted wooden square, rectangle and triangular blocks, Puzzles ([3-6], [9-12], [20-24], [36-48] pieces made of wood or cardboard that is at least 1.5mm thick.
Use	Paint or varnish on wooden blocks must be non-toxic.
Clinical or User Specifications	Building bricks must be Lego/Duplo brand, as other makes are not as robust and corners tend to chip off and discolour and they lose their ability to grip each other.
Accessories	
Care	
Supplier Warranty	N//A.

Hot water bottle: standard

NO TECHNICAL SPECIFICATIONS
NONE

Catalogue Number	Item Name	Available on Transversal Contract	Contract Number or Code
PHC-E-222	Hot water bottle: standard	No	

Description	Standard hot water bottle and removable towelling cover.	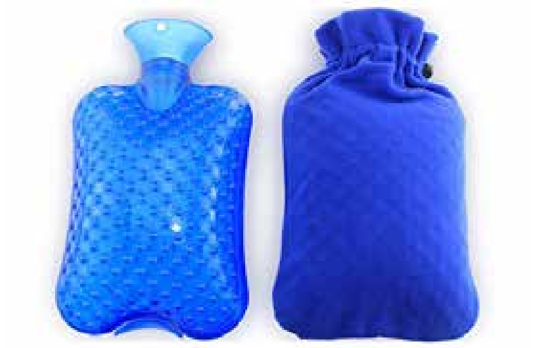
Use	For pain relief and muscle relaxation in patients with musculoskeletal, rheumatological and orthopaedic conditions.	
Clinical or User Specifications	Heat-resistant silicon rubber bottle, 1litre capacity.<br>Instead of hydro collator for hot-packs (more portable and durable). Also for working with ear moulds in audio patients.	
Accessories	Spare bung or sealing washer.	
Care	Beware of burns when preparing or applying hot water bottle. This product should not be purchased without a kettle being available at site.	
Supplier Warranty	1 year (excluding deliberate or accidental damage).	

Mat: gym

NO TECHNICAL SPECIFICATIONS NONE

Catalogue Number	Item Name	Available on Transversal Contract	Contract Number or Code
PHC-E-223	Mat: gym	No	

Description	Large foam rubber gym mat for exercises, as well as assessing and treating children. These mats can be easily rolled up and stored/ transported.	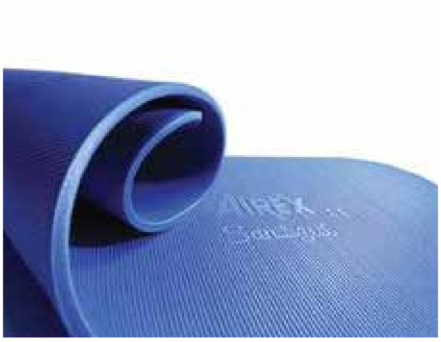
Use	For assessment and exercises, can be used for individual rehab or group therapy.	
Clinical or User Specifications	Size 180cm (L) x 100cm (W) x 1.5cm thick. Must be easy to roll up for transportation and storage. High-quality closed-cell memory foam, with a non-slip surface. Material should be easy to wipe and disinfect. Material should be durable and not prone to deformation.	
Accessories	None.	
Care	Do not place heavy objects or those with sharps edges on top of the mat, as this could damage the material. Do not expose to high temperatures.	
Supplier Warranty	2 years (excluding deliberate or accidental damage). Expected service life 5 years.	

Mirror: handheld

NO TECHNICAL SPECIFICATIONS
NONE

Catalogue Number	Item Name	Available on Transversal Contract	Contract Number or Code
PHC-E-224	Mirror: handheld, with cover	No	
PHC-E-225	Mirror: handheld, with handle	No	

Description	Small rectangular A4 sized mirror.<br>One model has a protective cover which also functions as an adjustable stand. Mirror can thus be tilted to different angles as needed.<br>The other model has a handle for easier holding, can also be hung vertically on a surface.
Use	For fitting hearing aids, learning to examine skin for pressure areas in neuro patients, providing oral rehabilitation and rehabilitating self- care.
Clinical or User Specifications	A protective cover which doubles as a stand is the most practical solution for travelling teams.<br>The model with a handle may be the preferred option for patient use.
Accessories	None.
Care	Standard cleaning and disinfection. Do not drop or scratch. Do not place heavy objects on top of mirror.
Supplier Warranty	6 months (excluding deliberate or accidental damage).

Soccer ball

NO TECHNICAL SPECIFICATIONS NONE

Catalogue Number	Item Name	Available on Transversal Contract	Contract Number or Code
PHC-E-226	Soccer ball	No	

Description	Plastic inflatable soccer ball.	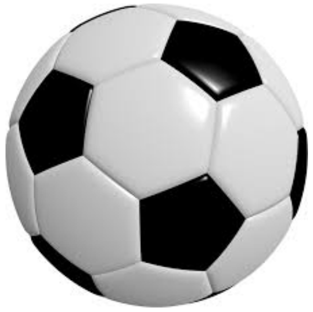
Use	For rehabilitating sports and musculoskeletal conditions, but also used in neuro rehab and in group work.	
Clinical or User Specifications	22cm diameter, the ball's weight must be in the range of 410 to 450 grams (14 to 16 ounces) and inflated to a pressure of between 0.6 and 1.1 standard atmospheres (8.8 and 16.2 psi) at sea level. The outer lining can be of synthetic leather, PVC or felt. The covering should be backed by multiple layers (minimum of 2) of polyester and the bladder should be of latex rubber or butyl. Silicone valve.	
Accessories	Pump and valve adapter.	
Care	Do not puncture or expose to high temperatures.	
Supplier Warranty	N/A.	

Standing frame: prone upper standing frame

NO TECHNICAL SPECIFICATIONS NONE

Catalogue Number	Item Name	Available on Transversal Contract	Contract Number or Code
PHC-E-227	Positioner: prone upper standing frame, small	RT233-2017	42192208-00527
PHC-E-228	Positioner: prone upper standing frame, medium	RT233-2017	42192208-00528
PHC-E-229	Positioner: prone upper standing frame, large	RT233-2017	42192208-00529

Description	Wooden modular standing frame, padded, in small, medium and large sizes.
Use	For assisting patients in standing and weight-bearing to improve postural control, encourage functional activities in standing posture and address/prevent joint contractures.
Clinical or User Specifications	Adjustable prone standing support.<br>Adjustable forward tilt to prone minimum 30 degrees.<br>Upright posture support: lower limb, pelvis and trunk.<br>Allow age appropriate abduction of hips.<br>Allow age appropriate hip and knee extension.<br>To provide knee support under patella, height adjustable and well padded for comfort<br>Foot support with adjustable forwards and backwards, abduction and lateral rotation<br>Lay tray / worktable: removable, adjustable height and forwards.<br>Sizes:<br>Extra Small: heel – head 65cm-100cm, hip width max 30cm<br>Small: heel to head 85-120cm. Hip width max 30cm<br>Medium: heel to head 200cm-135cm hip width max 35cm<br>Large: heel to head 135cm-160cm hip width max 40cm.
Accessories	
Care	
Supplier Warranty	1 year (excluding deliberate or accidental damage).

Tape measure: retractable, soft

NO TECHNICAL SPECIFICATIONS NONE

Catalogue Number	Item Name	Available on Transversal Contract	Contract Number or Code
PHC-E-230	Tape measure: retractable, soft		

Description	Flexible soft retractable measuring tape in a hard-plastic casing. 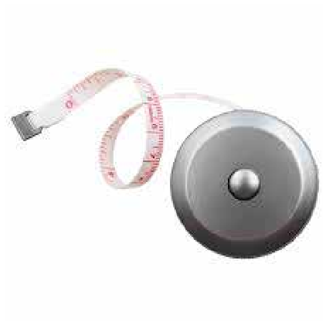
Use	For measuring swelling or muscle mass, as well as in manufacturing assistive devices and fitting them.
Clinical or User Specifications	Retraction mechanism should be durable and not prone to breaking.<br>Retracting 60 inch/1.5m non-stretch fiberglass tape in a durable plastic case for long life.<br>Non-stretch, non-fade.<br>The tape measure width is 0.3 inch/ 8mm, the retractable tape measure diameter is 2 inches. Large and clear markings in both Inches and cm for easier reading.
Accessories	None.
Care	Standard cleaning and disinfection. Do not overextend the tape as this will break the retraction mechanism.
Supplier Warranty	1 year (excluding deliberate or accidental damage).<br>Expected service life 5 years.

Toys: multisensory

NO TECHNICAL SPECIFICATIONS NONE

Catalogue Number	Item Name	Available on Transversal Contract	Contract Number or Code
PHC-E-231	Toys: multisensory	No	

Description	Collection of toys providing tactile, auditory and visual stimulation.
Use	For assessing, introducing new stimuli and treating patients with sensory integration issues.
Clinical or User Specifications	Colourful, robust 10-15cm plastic rattle; soft plastic squeaky toy with maximum dimension of 15cm; 15cm soft washable fabric ball; non-toxic modelling clay of 4 different colours, 100g of each colour; 11-13-inch plastic/vinyl baby doll with jointed hips and shoulders, sleep-eyes, removable set of clothing, 5 robust die-cast metal toy cars representing assorted actual vehicles (e.g. fire engine, lorry, sedan car, tractor, bakkie, delivery van, etc.), between 5 and 8cm long.<br>Items must be made of non-toxic materials, have no sharp edges or corners, no internal magnets.<br>Surfaces should be resistant to bacterial colonisation.<br>Toys must be washable and paint must endure alcohol or chlorine-based disinfection without peeling. Toys must not have small items that could come loose and be put into a child's mouth, e.g. loosely sewn buttons.
Accessories	Baby doll should come with toy feeding bottle.
Care	Clean and disinfect after each use.
Supplier Warranty	

Transfer board

NO TECHNICAL SPECIFICATIONS
NONE

Catalogue Number	Item Name	Available on Transversal Contract	Contract Number or Code
PHC-E-232	Transfer board	No	

Description	Varnished, curved wooden plank with hand holes cut into them.
Use	Enables patients to transfer between bed and wheelchair, by sliding along the board.
Clinical or User Specifications	The transfer board must have an oblong hole on each side Width: (750mm x 338mm) thickness (6mm-12mm maximum) the board must weigh 1.6-2kg and have a weight capacity 300kg.
Accessories	Strap for shoulder carry.<br>Carry bag.
Care	Standard cleaning and disinfection.
Supplier Warranty	N/A.<br>Expected service life 5 years.

Walking frame

NO TECHNICAL SPECIFICATIONS NONE

Catalogue Number	Item Name	Available on Transversal Contract	Contract Number or Code
PHC-E-233	Walking frame: adult	HM03-2015CW	2.1
PHC-E-234	Walking frame: paediatric	No	

Description	Aluminium four-legged height adjustable, foldable walking frame with rubber handles and durable rubber ferrules.	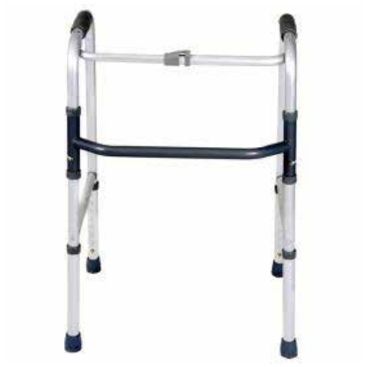
Use	For training patients before prescribing.	
Clinical or User Specifications	Walking aid, pulpit walker, folded metal frame, height adjustable.<br>Frame must fold into a flat position with legs together, When in the extended position the frame must be secure to prevent collapse while walking Folding/unfolding of the walker must be smooth and easy to operate. Frame may be riveted or bolted together.<br>Where components are joined, plastic spacers, fitting snugly to the tube, must ensure a rigid assembly.<br>Nuts must have nylon locking inserts. Bolts may not protrude beyond the nuts.<br>Rivets must have a washer on the clinched side to prevent them pulling loose.<br>There must be no sharp points. Tubes to be anodised aluminium of quality at least that of 6063T6.<br>Tubes must be at least 1,5 mm thick. The mechanism must be wobble-free, even in the fully extended position.<br>Height adjustable: 650-800 mm.<br>Height adjustment on all four legs by means of two spring-loaded pins and matching holes.<br>Spring loaded pins must be at least 8 mm in diameter. Holes must not be countersunk.<br>Pins must protrude at least 2 mm beyond the outside of the outer tube.<br>Height adjustment of the walker must be smooth and easy to operate.<br>Clearance between the sliding uprights must be as small as possible and must not exceed 0,6 mm.<br>The height adjustable legs must have an anti-rattle bush at the top of the inner tube.<br>The two front legs must be splayed forward.<br>Width at the base of the front end: +/- 450 mm (minimum).<br>The two back legs must be splayed backward.<br>Width at the base of the rear end: +/- 600 mm.	

	Durable rubber shoes must be fitted to each leg.<br>The shoes must be fitted so that they cannot be removed by pulling and twisting.<br>Shoe diameter: 44 mm where it makes contact with the floor. Shoe may taper to approximately 30 mm.<br>The hole in the shoe must be at least 35 mm deep.<br>The floor contact of the shoe must be concave with an anti-slip tread.<br>The end of the tube onto which the shoe is fitted must be plugged.<br>Hand grips must be of resilient and durable plastic this is resistant to perspiration and scuffing and which does not stain the hands.<br>The palm section of the hand grip may not include any pronounced lump.<br>Mass: less than 2 kg.<br>Items 2.1 and 2.2 will be considered as a series.<br>Should be compatible with small front castors to enable the prescriber to adjust it into a walker for patients who are unable to lift the walking frame safely between steps.
Accessories	Front castor wheels and stems specific to the walking frame.
Care	Ensure that all moveable parts are working and secure before use.
Supplier Warranty	1 year (excluding deliberate or accidental damage).<br>Expected service life 5 years.
Note to Procurement	Ensure that the frame is purchased together with fitting front castors.

Walking stick

NO TECHNICAL SPECIFICATIONS
NONE

Catalogue Number	Item Name	Available on Transversal Contract	Contract Number or Code
PHC-E-235	Walking stick: adult	HM03-2015CW	4.1
PHC-E-236	Walking stick: paediatric	No	

Description	Aluminium walking stick, adjustable. Adult and Paediatric sizes.	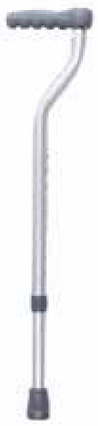
Use	For stock. There needs to be a minimum stock level set for assistive devices.	
Clinical or User Specifications	Walking stick, height adjustable: 650-900mm, low mass metal with crutch type T-handle. Adjustment by means of spring-loaded pins. Spring loaded pins must be hard aluminium, brass or mild steel nickel plated and adjustment must be easy and smooth. Durable plastic handgrip. With attached tip.	
Accessories	None.	
Care	Sand and grime may interfere with adjusting mechanism- ensure user keeps this as clean as possible.	
Supplier Warranty	1 year (excluding deliberate or accidental damage).	

Wedges: set of 4

NO TECHNICAL SPECIFICATIONS NONE

Catalogue Number	Item Name	Available on Transversal Contract	Contract Number or Code
PHC-E-237	Wedges: set of 4	No	

Description	Set of hard foam wedges covered in PVC or tarpaulin and of varying heights and lengths.
Use	For rehabilitating head control, postural control and scapular stability in neuro patients, as well as assisting in positioning neuro, musculoskeletal and orthopaedic patients.
Clinical or User Specifications	Core from very firm foam, Hygienic, (100% polyester) tarpaulin cover. Must be antibacterial plastic which is easy to wipe and clean. The wedges should be able to withstand weights and knocks usually sustained in a clinical and transportation setting without deformation. Load capacity 145kg.
Accessories	None.
Care	Do not place wedges underneath heavy or sharp objects as this may damage the shape or covering. Clients should be appropriately assessed and selected for this equipment. Adequate supervision and appropriate exercise prescription is required.
Supplier Warranty	2 years (excluding deliberate or accidental damage).<br>Expected service life 5 years.

Item	Length (cm)	Breadth (cm)	Height (cm)
Wedge 45 x 8	45	45	8
Wedge 45 x 15	45	45	15
Wedge 45 x 20	45	45	20
Sliding wedge 45	90	45	45

Weight band: ankle and wrist

NO TECHNICAL SPECIFICATIONS
NONE

Catalogue Number	Item Name	Available on Transversal Contract	Contract Number or Code
PHC-E-238	Weights: ankle and wrist, 200g to 2.5kg	No	

Description	Ankle and wrist bands with individual compartments to which metal weights are added, to adjust the total weight of the band. Band is secured onto wrist or ankle with d-ring and Velcro straps to accommodate different sized limbs.	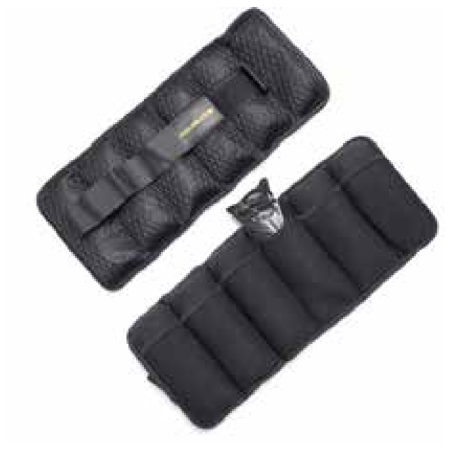
Use	Used for exercise rehabilitation, for strengthening muscles in orthopaedic and musculoskeletal conditions.	
Clinical or User Specifications	The outer layer should be antibacterial plastic which is easy to wipe and clean. The wedges should be able to withstand weights and knocks usually sustained in a clinical and transportation setting without deformation.<br>5 individual weight bags of 200g. total weight can be adjusted from 200g to 2,5kg.	
Accessories	None.	
Care	Clients should be appropriately assessed and selected for this equipment. Adequate supervision and appropriate exercise prescription is required to prevent injury.	
Supplier Warranty	2 years (excluding deliberate or accidental damage).<br>Expected service life 5 years.	

Wheelchair

NO TECHNICAL SPECIFICATIONS
NONE

Catalogue Number	Item Name	Available on Transversal Contract	Contract Number or Code
PHC-E-239	Wheelchair: Standard back height, 25cm x 30cm, no customisation, with basic tray	RT233	42192210-00000 plus 2192208-00505
PHC-E-240	Wheelchair: Standard back height, 30cm x 30cm, with customisation and basic tray	RT233	42192210-00001 plus 42192208-00015 plus 2192208-00507
PHC-E-241	Wheelchair: Standard back height, 30cm x 40cm, with customisation and basic tray	RT233	42192210-00003 plus 42192208-00015 plus 42192208-00011 plus 2192208-00507
PHC-E-242	Wheelchair: Short back height, 36cm x 40cm, with customisation and basic tray	RT233	42192210-00017 plus 42192208-00015 plus 42192208-00011 plus 2192208-00508
PHC-E-243	Wheelchair: Standard back height, 41cm wide, with customisation and basic tray	RT233	42192210-00006 plus 42192208-00015 plus 42192208-00011 plus 2192208-00509
PHC-E-244	Wheelchair: Standard back height, 46cm wide, with customisation and basic tray	RT233	42192210-00007 plus 42192208-00015 plus 42192208-00011 plus 2192208-00510
PHC-E-246	Wheelchair: Standard back height, 51cm wide, with customisation and basic tray	RT233	42192210-00008 plus 42192208-00015 plus 42192208-00013 plus 42192208-00511

Description	Basic folding-frame wheelchair, standard back height. Epoxy-coated finish. Customised for rugged terrain, with basic tray.	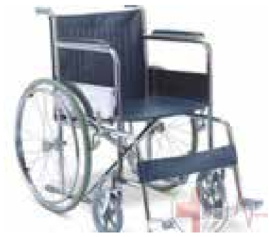
Use	This wheelchair in 7 seat sizes is intended to be kept at the facility for assessment and training of users. Based on the assessment, a customised version can be ordered for the patient from the RT233 Term Contract.	
Clinical or User Specifications	Chairs have been selected with customisation options for rugged terrain rear wheels and castors.<br>Comprehensive user specifications are available in RT233 -2017 – detailed technical specifications. Too extensive to repeat here.<br>Weight not to exceed 24kg with rubber castors.	
Accessories	Repair/tool kit:<br>4 rear wheel bearings<br>4 castor axle bearings<br>4 castor stem bearings<br>4 seat guides (if applicable to the model)<br>adjustment tool.	
Care	User must be trained in care, maintenance and cleaning. Repair and modification must be offered on site and as outreach.	
Supplier Warranty	Frame and cross-bars guaranteed for a minimum of 3 years during appropriate use.	

Speech Therapy/Audiology

Audiometer: portable

NO TECHNICAL SPECIFICATIONS NONE

Catalogue Number	Item Name	Available on Transversal Contract	Contract Number or Code
PHC-E-247	Audiometer: portable	RT274-2018ME	42182419-009331

Description	Laptop-based two-channel audiometer.
Use	For portable audiometric testing, where an acoustically insulated booth is not available. The audiometer uses noise-cancelling headphones to reproduce a quiet environment.
Clinical or User Specifications	Masked pure tone and warble tone air and bone conduction and speech testing. Masking sounds must include narrowband, white and speech noise.<br>Must test 125-20 000Hz from -10-120dB (100dB for frequencies above 12 000Hz) in increments of 5db.<br>Unit must either have internal storage for pre-recorded speech in South African accents/languages, or must have an external CD player, from which speech can be played. The audiometer must control the CD player directly.<br>Requires compatible laptop, with sufficient processing power and Random Access Memory, to perform the necessary processing.
Accessories	Must be supplied complete with noise-cancelling earphones, paired insert phones, bone conductor, response button, talk-forward, built-in or gooseneck microphone. Either built-in pre-recorded speech storage, or USB ports and an external CD player to play pre-recorded speech.<br>Manuals, software and carry case.<br>Power cord.<br>Charger, if applicable.
Care	
Supplier Warranty	2 years.<br>Expected service life 10 years.
Instructions to Suppliers	Personnel to be trained in use of the equipment. Software updates to be provided free of charge. Describe requirements for the compatible computer.
Note to Procurement	Compatible laptop computer to be sourced from available term contracts.

Cards: therapy, speech

NO TECHNICAL SPECIFICATIONS
NONE

Catalogue Number	Item Name	Available on Transversal Contract	Contract Number or Code
PHC-E-248	Vocabulary, picture – food	RT275-2016	85122108-00438
PHC-E-249	Vocabulary, picture – everyday objects	RT275-2016	85122108-00439
PHC-E-250	Vocabulary, picture – basic verbs	RT275-2016	85122108-00440
PHC-E-251	Vocabulary, picture – familiar verbs	RT275-2016	85122108-00441
PHC-E-252	Vocabulary, picture – categories	RT275-2016	85122108-00442
PHC-E-253	Vocabulary, picture – what`s wrong?	RT275-2016	85122108-00447

Description	Sets of high- quality photozgraph cards – themed equipment for language and sensory development.
Use	To treat language delays and communication pathologies.
Clinical or User Specifications	
Accessories	
Care	
Supplier Warranty	

NO TECHNICAL SPECIFICATIONS NONE

HiPro box

Catalogue Number	Item Name	Available on Transversal Contract	Contract Number or Code
PHC-E-254	HiPro box	RT274-2018ME	42182419-00330

Description	A box which permits the connection of programmable hearing aids to a computer, for adjustment of the hearing aid to the patient's needs.
Use	Interface connector for programming hearing aids.
Clinical or User Specifications	Uses open standard HiPro interface. Connects to a laptop which contains the programming software (via USB) and the hearing aid (via specialised connectors).<br>Mains powered.
Accessories	Power cable, USB-to-USB cable, interface cable to hearing aids. Modular software for use with the device.
Care	
Supplier Warranty	2 years.<br>Expected service life 10 years.
Instructions to Suppliers	Hardware installation assistance and training must be provided. Describe requirements for the compatible computer.

Noise makers

NO TECHNICAL SPECIFICATIONS NONE

Catalogue Number	Item Name	Available on Transversal Contract	Contract Number or Code
PHC-E-255	Noise makers	No	

Description	Set of three gadgets that produce sounds in specific frequency ranges.
Use	Assessment of hearing range in pre-verbal babies.
Clinical or User Specifications	1 sturdy plastic rattle with tiny balls inside that produces a sound of about 2000Hz when gently tilted, 1 sturdy plastic rattle with bigger balls inside that produces a sound of about 500Hz when gently tilted, 1 plastic or metal handle with three to five round brass bells each with a diameter of about 15-20mm.
Accessories	None.
Care	
Supplier Warranty	1 year.

OAE/AABR screener (portable)

NO TECHNICAL SPECIFICATIONS NONE

Catalogue Number	Item Name	Available on Transversal Contract	Contract Number or Code
PHC-E-256	OAE/AABR screener (portable)	RT274-2018ME	42182419-009349

Description	A hand-held portable unit capable of conducting screening DPOAEs and automated ABRs.
Use	
Clinical or User Specifications	Must consist of the hand-held screening unit, probe, system cables (USB to connect system to computer); ABR cable. Must store at least 50 tests and have a built-in normative database to reference pass/refer results.
Accessories	Charger unit and power cord, carry case, instruction manual, data management software CD.<br>Ear tip accessory box, including probe replacement filters and 50 ear tips of various sizes, including for babies.
Care	
Supplier Warranty	2 years.<br>Expected service life 10 years.
Note to Procurement	Supplier to indicate whether firmware updates may be necessary in the lifetime of the instrument; if so, manner of obtaining/installing the updates, at no additional cost.

Otolight

NO TECHNICAL SPECIFICATIONS NONE

Catalogue Number	Item Name	Available on Transversal Contract	Contract Number or Code
PHC-E-257	Otolight	RT274-2018ME	42143504-00010

Description	Otolight for ear mould impressions Complete with compatible batteries, hard casing and minimum of 2 tapered tips (hard transparent plastic tips).<br>Battery-powered.
Use	
Clinical or User Specifications	
Accessories	Supplied with a set of non-corrosive batteries.
Care	
Supplier Warranty	2 years.

Otoscope: portable

NO TECHNICAL SPECIFICATIONS NONE

Catalogue Number	Item Name	Available on Transversal Contract	Contract Number or Code
PHC-E-258	Otoscope: portable	RT274-2018ME	RT274-2018ME

Description	Battery-powered otoscope for ear examination.
Use	Examination and instrumentation of external auditory meatus and middle ear.
Clinical or User Specifications	Robust case with zip or other closure, housing otoscope head, handle, and specula.<br>The Otoscope to be supplied with 3 polypropylene specula in each size 2.5, 3, 4 and 5 mm – re-usable quality.<br>The Otoscope is to utilize fibre optic cool-light supply with no reflections. The Otoscope is to have a Wide-angled viewing lens for instrumentation whilst under magnification. The unit is to be completely sealed for Pneumatic otoscopy (i.e. not open operating model). Light LED (optimized for best tissue viewing).<br>Knurled handle, housing two non-corrosive batteries; connector for the instrument head, and rheostat for brightness adjustment.
Accessories	Specula.<br>Case.<br>Spare globe.<br>Non-corrosive batteries (1 set).
Care	
Supplier Warranty	Minimum 7-year warranty on head and handle.<br>Expected service life 15 years.
Note to Procurement	These specifications differ from RT274, and should be used in future procurements, particularly regarding warranty.

Tympanometer: portable, screening

NO TECHNICAL SPECIFICATIONS
NONE

Catalogue Number	Item Name	Available on Transversal Contract	Contract Number or Code
PHC-E-259	Tympanometer: portable, screening	RT274-2018ME	RT274-2018ME

Description	Portable screening tympanometer with high frequency and reflex testing options.
Use	For hearing screening.
Clinical or User Specifications	Must have standard 226Hz and 1 000Hz probe tones and must test reflexes at 250, 1000, 2000 and 4000Hz. Must include an internal printer, probe assembly, memory for at least 12 tests.
Accessories	Two rolls of paper.<br>Box of ear tips of at least six different sizes, with three tips of each size.<br>Carry case, instruction manual.<br>Replacement paper rolls.
Care	Avoid dropping and moisture.
Supplier Warranty	2 years.<br>Expected service life 10 years.
Note to Procurement	Consumables; paper to be compatible with the internal printer.

equipment
general

**BUILDING MAINTENANCE**	**118**
Basic tool kit	118
Extension cable: electrical, general purpose	120
Ladder: aluminium, folding	121
Multi-plug	122
**CLEANING**	**123**
Bin: pedal 4,5 l	123
Bin: pedal 20l	124
Bin: wall-mounted, refuse bag holding unit	125
Bin: sanitary towel	126
Bin: sharps	127
Broom: interior use	128
Buckets: colour coded	129
Dust pan and brush set	130
Mop: colour-coded	131
Mop: static head, complete	132
Paper towel dispenser: folded towels	133
Paper towel dispenser: roll	134
Soap dispenser	135
Spray bottle	136
Squeegee	137
Toilet roll dispenser	138
Trolley: janitor	139
**COLD CHAIN**	**140**
Cooler box	140
Thermometer: data logging	141
Thermometer: refrigerator	142
Thermometer: room	143
**FIRE SAFETY**	**144**
Fire extinguisher	144
Fire hose on reel	145
Smoke alarm	146
**GARDEN**	**147**
Bins: waste, heavy duty	147
Broom	148
Edge Trimmer/brush cutter: heavy duty	149
Extension cable: electrical, garden	150
Fork	151
Gardening set: 3-piece	152
Hedge clipper: shears, manual	153
Hoe	154
Hosepipe: garden	155
Lawn mower: Electric, heavy duty	156
Lawn mower: petrol, heavy duty	157
Pick mattock	158
Rake: leaf and grass, heavy duty	159
Rake: steel, heavy duty	160
Shovel: square mouth	161
Slasher	162
Spade	163
Watering can	164
Wheelbarrow	165
**INFORMATION AND COMMUNICATIONS TECHNOLOGY**	**166**
**KITCHEN**	**167**
Kettle: electric	167
Rack: dish	168
Rack: cutlery	169
Urn: electric	170
Watercooler: free-standing	171
**MISCELLANEOUS**	**172**
Bins: brazier	172
Clock: wall	173
Complaints and suggestions box	174
Gun safe	175
Notice board: wall-mounted	176
Pamphlet-holding boxes	177
Poster display boards	178
Whiteboard	179

Building maintenance

Basic tool kit

TECHNICAL SPECIFICATIONS page 631 to 666

Catalogue Number	Item Name	Available on Transversal Contract	Contract Number or Code
PHC-E-194	Allen key set	No	
PHC-E-195	Clamps, adjustable (pair)	No	
PHC-E-196	Drill, cordless	No	
PHC-E-197	Drill bit set	No	
PHC-E-078	File, flat, combined	No	
PHC-E-198	Foam cutter, electric	No	
PHC-E-199	Frying pan, electric, for splints	No	
PHC-E-080	Funnel, plastic, medium	No	
PHC-E-081	Hacksaw, all-metal	No	
PHC-E-082	Hacksaw blades, assorted	No	
PHC-E-072	Hammer, claw, approximately 450gm	No	
PHC-E-200	Hearing Protector	No	
PHC-E-201	Heat gun, electric	No	
PHC-E-202	Jigsaw, electric	No	
PHC-E-203	Jigsaw blades, set	No	
PHC-E-204	Measuring rule, folding	No	
PHC-E-077	Pliers set	No	
PHC-E-083	Pruning saw, medium	No	
PHC-E-084	Pruning shears, bypass, heavy duty	No	
PHC-E-205	Punch, revolving	No	
PHC-E-206	Punch, eyelet	No	

PHC-E-156	Riveter, hand	No	
PHC-E-086	Scraper, paint, medium	No	
PHC-E-073	Screwdriver set	No	
PHC-E-079	Sharpening stone, medium/fine combined	No	
PHC-E-074	Shifting spanner, 0-40mm	No	
PHC-E-075	Swivel-head spanner set	No	
PHC-E-207	Tape measure, industrial	No	
PHC-E-157	Toolbox	No	
PHC-E-208	Utility knife, replaceable blades	No	
PHC-E-085	Voltage tester, contactless	No	
PHC-E-076	Water pump pliers	No	
PHC-E-209	Workbench, folding, portable	No	

Description	Basic tool kit.	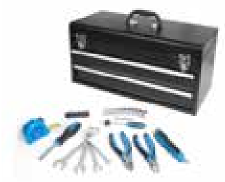
Use	For general maintenance and maintenance of garden tools.	
Clinical or User Specifications	This kit has the commonest tools required for non-specialised maintenance. Excludes plumbing and electrical, building and carpentry.	
Accessories or Related Consumables	None.	
Care	Clean tools after use to avoid corrosion.	
Supplier Warranty	1 year (excluding deliberate or accidental damage).	

Extension cable: electrical, general purpose

TECHNICAL SPECIFICATIONS page 635

Catalogue Number	Item Name	Available on Transversal Contract	Contract Number or Code
PHC-E-069	Extension cable: Electrical, general purpose 3m	No	
PHC-E-070	Extension cable: Electrical, general purpose 5m	No	
PHC-E-071	Extension cable: Electrical, general purpose 10m	No	
PHC-E-211	Extension cable: on reel, 20m	No	

Description	Extension cable, electrical. General purpose, various lengths available.<br>Reel-mounted cable is for use by outreach teams, as the cable is protected from damage in transit.	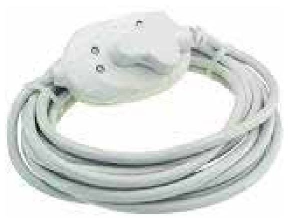
Use	Electrical extension for general use.	
Clinical or User Specifications	This extension has a normal 15A 3-pin plug on one end, and two 15A 3-pin connectors at the other.	
Accessories or Related Consumables	None.	
Care	Coil without twisting when not in use.<br>If accidentally cut during use, have it properly mended by maintenance personnel.<br>If extension becomes warm in use, burns, or gives off sparks, discontinue use immediately.	
Supplier Warranty	1 year (excluding deliberate or accidental damage).	

Ladder: aluminium, folding

TECHNICAL SPECIFICATIONS page 647

Catalogue Number	Item Name	Available on Transversal Contract	Contract Number or Code
PHC-E-068	Ladder: aluminium	No	

Description	Ladder: aluminium.	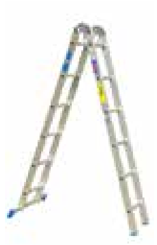
Use	For gardening and general maintenance work.	
Clinical or User Specifications	Heavy duty folding and extendable aluminium stepladder.	
Accessories or Related Consumables	None.	
Care	Clean after use to prevent metal corrosion.	
Supplier Warranty	1 year (excluding deliberate or accidental damage).	

Multi-plug

TECHNICAL SPECIFICATIONS page 649

Catalogue Number	Item Name	Available on Transversal Contract	Contract Number or Code
PHC-E-210	Multi-plug	No	

Description	Multi-plug adaptor.	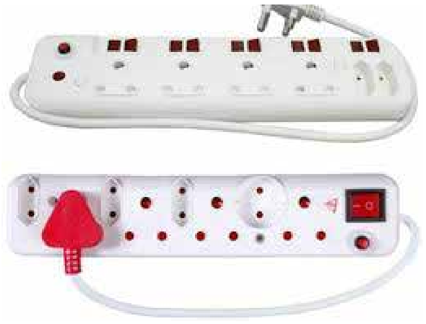
Use	For using machines (audiometers, screening tympanometers, electrotherapy, splinting pans) where plug points are not ideally placed (e.g. at schools, community halls, older clinics).	
Clinical or User Specifications		
Accessories or Related Consumables	None.	
Care		
Supplier Warranty	1 year (excluding deliberate or accidental damage).	

Cleaning

Bin: pedal 4,5 l

TECHNICAL SPECIFICATIONS page 763

Catalogue Number	Item Name	Available on Transversal Contract	Contract Number or Code
PHC-E-001	Bin: pedal, 4,5l	No	

Description	Light-duty stainless steel pedal bin.	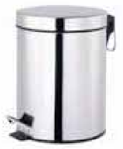
Use	Waste bin for use in low-use areas, where space is restricted.	
Clinical or User Specifications	All stainless steel construction.<br>Rubber bumpers inside lid to reduce noise.<br>Can be used with or without a bin liner, depending on the application.	
Accessories or Related Consumables		
Care	Standard cleaning and disinfection.	
Supplier Warranty	2 years on all components (excluding deliberate or accidental damage).	

Bin: pedal 20l

TECHNICAL SPECIFICATIONS page 668

Catalogue Number	Item Name	Available on Transversal Contract	Contract Number or Code
PHC-E-002	Bin: pedal, 20l	No	

Description	Heavy-duty stainless steel pedal bin.	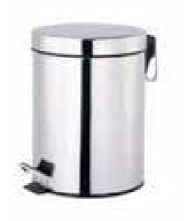
Use	Waste bin for use in high-use areas, where a domestic light-duty bin is not suitable.	
Clinical or User Specifications	All stainless steel construction.<br>Rubber bumpers inside lid to reduce noise.<br>Non-scratch, rubber-tipped feet.<br>Can be used with or without a bin liner, depending on the application.	
Accessories or Related Consumables		
Care	Standard cleaning and disinfection.	
Supplier Warranty	2 years on all components (excluding deliberate or accidental damage).	

Bin: wall-mounted, refuse bag holding unit

TECHNICAL SPECIFICATIONS page 669

Catalogue Number	Item Name	Available on Transversal Contract	Contract Number or Code
PHC-E-003	Bin: wall-mounted, refuse bag holding unit	No	

Description	Bin, wall-mounted, refuse bag holding unit.	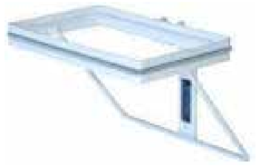
Use	Waste bin for use in high-use areas, where waste is deposited directly into a black plastic bag.	
Clinical or User Specifications	The unit is a wall-mounted horizontal frame, in which a waste bag is secured in the open-mouth position. The bag is held in place by an outer frame.	
Accessories or Related Consumables		
Care	Standard cleaning and disinfection.	
Supplier Warranty	2 years on all components (excluding deliberate or accidental damage).	

Bin: sanitary towel

TECHNICAL SPECIFICATIONS page 670

Catalogue Number	Item Name	Available on Transversal Contract	Contract Number or Code
PHC-E-004	Bin: sanitary towel	No	

Description	Bin: sanitary towel.	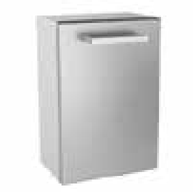
Use	For disposal of sanitary towels in facilities with septic tank sewerage disposal.	
Clinical or User Specifications		
Accessories or Related Consumables		
Care	Standard cleaning and disinfection.	
Supplier Warranty	1 year on all components (excluding deliberate or accidental damage).	

Bin: sharps

TECHNICAL SPECIFICATIONS page 671

Catalogue Number	Item Name	Available on Transversal Contract	Contract Number or Code
PHC-E-005	Bin: sharps	No	

Description	Bin: sharps.	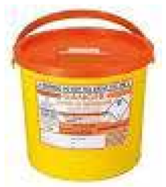
Use	For discarding syringes, needles, broken vials, cartridges, scalpel blades, lancets, and other contaminated sharp objects.	
Clinical or User Specifications	Robust, puncture-proof, tamper-proof, leak-proof.<br>One-way entry port, no spillage if knocked over.<br>Tight-fitting lid, non-opening once closed.<br>Bright yellow colour, red lid, labelled "Danger Contaminated Sharps".<br>International hazardous waste symbol.<br>Must be securely fixed to a wall, trolley or table top.	
Accessories or Related Consumables	Securing bracket and mounting hardware.	
Care	Seal once ¾ full.<br>Standard cleaning and disinfection. Hot water wash (90ºC).	
Supplier Warranty	1 year on all components (excluding deliberate or accidental damage).	

Broom: interior use

TECHNICAL SPECIFICATIONS page 672

Catalogue Number	Item Name	Available on Transversal Contract	Contract Number or Code
PHC-E-006	Broom: interior use	No	

Description	Household broom.	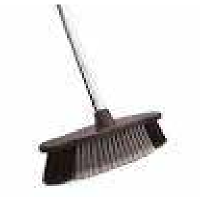
Use	For sweeping inside buildings. There is a different heavy-duty broom for external use.	
Clinical or User Specifications	Robust, non-rusting handle.<br>Replaceable broom head, attached with suitable metal bracket and screws.<br>Soft bristles, for interior use.<br>Protective plastic strip around broom head.<br>Hanging hole at end of handle.	
Accessories or Related Consumables		
Care	Standard cleaning and disinfection.	
Supplier Warranty	1 year on all components (excluding deliberate or accidental damage).	

Buckets: colour coded

TECHNICAL SPECIFICATIONS page 673

Catalogue Number	Item Name	Available on Transversal Contract	Contract Number or Code
PHC-E-007	Bucket: red	No	
PHC-E-008	Bucket: blue	No	
PHC-E-009	Bucket: green	No	

Description	Colour-coded bucket: Heavy duty.	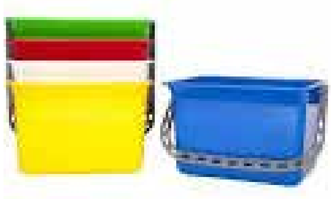
Use	For cleaning of floors, furniture and fittings.	
Clinical or User Specifications	Rectangular shape, to suit janitor trolley.<br>Used in combination with colour-coded mops and cloths, for cleaning.<br>**Red bucket**: toilet areas.<br>**Blue bucket**: clinical areas and non-clinical service areas.<br>**Green bucket**: bathroom and consulting room basins.	
Accessories or Related Consumables		
Care	Must be rinsed and dried after use.	
Supplier Warranty	1 year (excluding deliberate or accidental damage).	

Dust pan and brush set

TECHNICAL SPECIFICATIONS page 674

Catalogue Number	Item Name	Available on Transversal Contract	Contract Number or Code
PHC-E-010	Dust pan and brush set	No	

Description	Dust pan and brush.	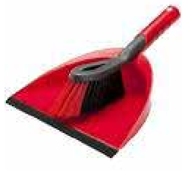
Use	For collecting dust and particles inside buildings.	
Clinical or User Specifications	Heavy-duty plastic dust pan, with hole in handle for hanging.<br>Heavy-duty soft-bristle, plastic brush with handle. Hole in handle for hanging.	
Accessories or Related Consumables		
Care	Standard cleaning and disinfection.	
Supplier Warranty	1 year on all components (excluding deliberate or accidental damage).	

Mop: colour-coded

TECHNICAL SPECIFICATIONS page 675

Catalogue Number	Item Name	Available on Transversal Contract	Contract Number or Code
PHC-E-015	Mop: red	No	
PHC-E-016	Mop: blue	No	
PHC-E-017	Mop: black	No	

Description	Colour-coded mops.	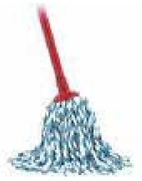
Use	For cleaning of floors.	
Clinical or User Specifications	Used in combination with colour-coded buckets and cloths, for cleaning.<br>**Red mop**: toilet areas.<br>**Blue mop**: clinical areas and non-clinical service areas.<br>**Black mop** (must be labelled): exterior areas.	
Accessories or Related Consumables		
Care	Must be rinsed and dried after use.	
Supplier Warranty	1 year (excluding deliberate or accidental damage).	

Mop: static head, complete

NO TECHNICAL SPECIFICATIONS
NONE

Catalogue Number	Item Name	Available on Transversal Contract	Contract Number or Code
PHC-E-018	Mop: static head, complete	No	

Description	Mop with broad head over which a fitted microfibre cloth is stretched.	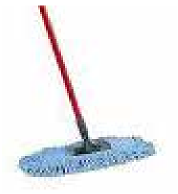
Use	For general cleaning of dry floors, dust removal on high fixtures.	
Clinical or User Specifications	Long-handled mop with a flat head. Microfibre cloth is stretched over the head. Cloth is exchanged when dirty.	
Accessories or Related Consumables	Microfibre sleeve for mop: static head. PHC-C-210.	
Care	Cloths are re-usable. Wash separately from other laundry.	
Supplier Warranty	1 year (excluding deliberate or accidental damage).	

Paper towel dispenser: folded towels

TECHNICAL SPECIFICATIONS page 677

Catalogue Number	Item Name	Available on Transversal Contract	Contract Number or Code
PHC-E-020	Paper towel dispenser: folded towels	RT 14-2016	47131710-00003

Description	A holder for packs of folded paper towels.	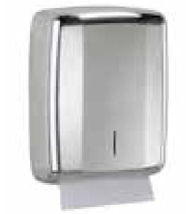
Use	For all areas where there is a hand washbasin.	
Clinical or User Specifications	Wall-mounted, polycarbonate dispenser for folded paper towels.<br>Must be fixed to the wall, in proximity to the washbasin.	
Accessories or Related Consumables	None.	
Care	Standard cleaning and disinfection.	
Supplier Warranty	2 years on all components (excluding accidental or deliberate damage).	

Paper towel dispenser: roll

TECHNICAL SPECIFICATIONS page 678

Catalogue Number	Item Name	Available on Transversal Contract	Contract Number or Code
PHC-E-021	Paper towel dispenser: roll	No	

Description	A holder for rolls of paper towel.	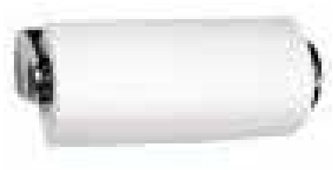
Use	For all areas where there is a hand washbasin.	
Clinical or User Specifications	Simple, heavy-duty stainless steel roll-holder, that will accept non-proprietary standard or large-diameter perforated paper rolls (readily purchasable).<br>Not suitable for industrial-size rolls.<br>Must be affixed to the wall, in proximity to the washbasin.	
Accessories or Related Consumables	None.	
Care	Standard cleaning and disinfection.	
Supplier Warranty	5 years on all components (excluding accidental or deliberate damage).	

Soap dispenser

TECHNICAL SPECIFICATIONS page 679

Catalogue Number	Item Name	Available on Transversal Contract	Contract Number or Code
PHC-E-014	Soap dispenser	No	

Description	A soap dispenser for washing hands.	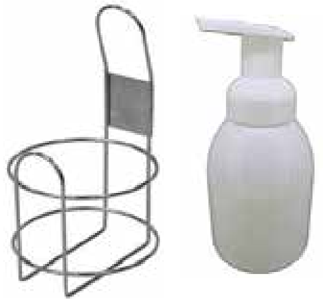
Use	For all areas where there is a hand washbasin.	
Clinical or User Specifications	Simple, heavy-duty stainless steel cage unit, that will accept non-proprietary, standard replaceable, plastic pump-action bottles.<br>Bottles are re-fillable with bulk-purchased liquid soap.<br>Bottles can be replaced when pump mechanism fails.<br>Must be affixed to the wall, in proximity to the washbasin.	
Accessories or Related Consumables	Two empty bottles, wall-mounting hardware.	
Care	Standard cleaning and disinfection.	
Supplier Warranty	5 years on metal components (excluding accidental or deliberate damage).	

Spray bottle

TECHNICAL SPECIFICATIONS page 680 to682

Catalogue Number	Item Name	Available on Transversal Contract	Contract Number or Code
PHC-E-022	Spray bottle: 750ml	No	
PHC-E-023	Spray bottle: 2l	No	
PHC-E-024	Spray bottle: 10l backpack	No	

Description	Spray bottles for surface application of cleaning liquids. 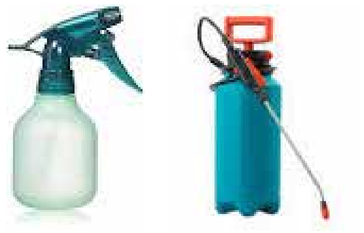
Use	To spray window cleaner, detergents, disinfectants, weed killer, insecticides.
Clinical or User Specifications	The small size has a simple trigger-action pump mechanism, and is suitable for light, intermittent work.<br>The medium size is for more sustained application – it is pressurised before use with a built-in pump.<br>The large unit is primarily for outdoor use. The unit is pressurised with a built-in pump, and the contents are then applied with a spray wand.
Accessories or Related Consumables	None.
Care	Standard cleaning and disinfection.
Supplier Warranty	1 year (excluding accidental or deliberate damage).

Squeegee

TECHNICAL SPECIFICATIONS page 683

Catalogue Number	Item Name	Available on Transversal Contract	Contract Number or Code
PHC-E-150	Squeegee: window	No	

Description	Squeegee: medium length handle.	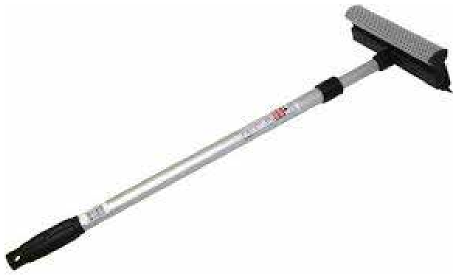
Use	For cleaning of windows.	
Clinical or User Specifications	Squeegee with medium-length handle, for cleaning and drying of windows.<br>Silicon rubber live edge. The squeegee is dragged across the window pane after washing with a cloth and rinsing, to avoid smearing.<br>Handle is sufficiently long enough to reach tops of window panes, but not long enough to twist during use.	
Accessories or Related Consumables		
Care	Must be rinsed and dried after use.	
Supplier Warranty	1 year (excluding deliberate or accidental damage).	

Toilet roll dispenser

TECHNICAL SPECIFICATIONS page 684

Catalogue Number	Item Name	Available on Transversal Contract	Contract Number or Code
PHC-E-025	Dispenser: toilet roll	RT 14-2016	47131710-00000

Description	Holder for rolls of toilet paper.	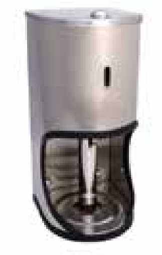
Use	For all areas where there is a toilet.	
Clinical or User Specifications	Wall-mounted stainless steel unit.<br>Capacity 2 rolls.<br>Must be affixed to the wall, in proximity to the toilet bowl.	
Accessories or Related Consumables	None.	
Care	Standard cleaning and disinfection.	
Supplier Warranty	5 years on all components (excluding accidental or deliberate damage).	

Trolley: janitor

TECHNICAL SPECIFICATIONS page 685

Catalogue Number	Item Name	Available on Transversal Contract	Contract Number or Code
PHC-E-030	Trolley: janitor	No	

Description	Janitor trolley.	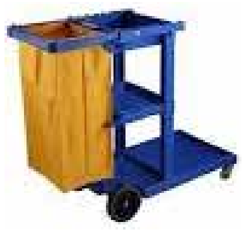
Use	Contains all requirements for efficient cleaning in a mobile unit.	
Clinical or User Specifications	The trolley holds a large refuse bag kept in the open position on the frame by a retainer. There is a sealing lid over the mouth of the bag, to prevent spillage and odours escaping.<br>Alongside the receptacle are three shelves. The top shelf is sunken, to accommodate spray bottles and other containers. The middle and lower shelves are enclosed by a rail on all sides, and house cloths and brushes.<br>The free edge of the top shelf has hooks or snap holders, into which mop handles can be secured when the unit is moving.<br>Space for three mops (or mop/broom combinations).<br>The floor of the trolley extends beyond the shelves, to provide a support for two heavy-duty buckets.<br>The trolley is castor-mounted for easy movement into and out of rooms.	
Accessories or Related Consumables	Colour-coded Mops, buckets, spray bottles to be ordered as separate items – see Table of Contents at the beginning of this section.<br>Cloths to be ordered under consumables.	
Care	Clean after use as with standard cleaning and disinfectant solutions.<br>Refuse bag disposable. Replace after use.	
Supplier Warranty	2 years on all components (excluding deliberate or accidental damage).	

Cold chain

Cooler box

TECHNICAL SPECIFICATIONS page 686

Catalogue Number	Item Name	Available on Transversal Contract	Contract Number or Code
PHC-E-035	Vaccine cooler box	No	

Description	Insulated container for vaccine transport.	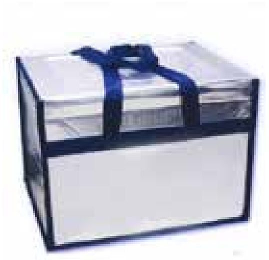
Use	For maintenance of the cold chain when vaccine is transported to the field.	
Clinical or User Specifications	The purpose of this item is to keep the temperature of transported vaccine within specified limits.<br>It is vital that the vaccine should not be frozen, as this will inactivate freeze-sensitive and lyophilised vaccines.<br>To compensate for the effects of opening and closing the box, a cold-life requirement of 24 hours has been selected, based on the assumption that the vaccines will be used within 12 hours of packing.<br>A foam pad is provided, to place over the cold packs, during an immunisation session. Placing the vaccine directly on an ice-pack is not recommended.	
Accessories or Related Consumables	Water packs for cooler box. PHC-C-203.	
Care	Clean after use as per manufacturer's instructions.<br>Fill water-packs with tap water and cool in refrigerator as per supplier's instructions. Do not freeze.<br>Do not replace water-packs with pre-filled types, as these may freeze below 0 degrees, and inactivate the vaccine.	
Supplier Warranty	1 year (excluding deliberate or accidental damage).	

Thermometer: data logging

TECHNICAL SPECIFICATIONS page 682

Catalogue Number	Item Name	Available on Transversal Contract	Contract Number or Code
PHC-E-042	Data logging thermometer	No	

Description	Thermometer which records temperature at pre-set intervals.	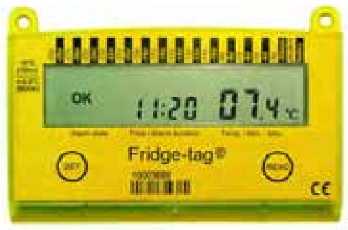
Use	For use with vaccine and medicine refrigerators.	
Clinical or User Specifications	The thermometer has a temperature probe which is placed inside the refrigerator – the connecting wire is very thin, and will not interfere with the door seal.<br>The body of the thermometer has a display, and will be mounted on or near the exterior of the refrigerator.<br>The instrument records temperatures at pre-set intervals, and stores the readings for a month – thereafter it overwrites the readings in memory with new readings.<br>Permits scrolling through readings for previous days.<br>A monthly report can be downloaded to a computer.<br>The instrument has an internal non-replaceable battery with a 2-year life-span.	
Accessories or Related Consumables	Temperature probe.	
Care	Standard cleaning and disinfection.<br>**THE INSTRUMENT MUST BE REPLACED EVERY TWO YEARS.**	
Supplier Warranty	2 years (excluding deliberate or accidental damage).	

Thermometer: refrigerator

TECHNICAL SPECIFICATIONS page 688

Catalogue Number	Item Name	Available on Transversal Contract	Contract Number or Code
PHC-E-153	Thermometer: refrigerator	No	

Description	Simple thermometer for use inside a standard refrigerator.	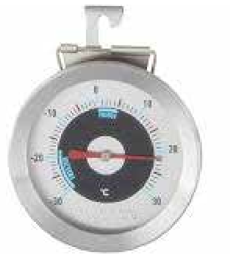
Use	To determine that the thermostat setting delivers the desired temperature range.	
Clinical or User Specifications	Indicates temperature inside the refrigerator at any given time. Does not record maximum and minimum temperatures.<br>Analogue-type. No batteries required.<br>Stainless steel.	
Accessories or Related Consumables		
Care	Do not drop.	
Supplier Warranty	2 years (excluding deliberate or accidental damage).	

Thermometer: room

TECHNICAL SPECIFICATIONS page 689

Catalogue Number	Item Name	Available on Transversal Contract	Contract Number or Code
PHC-E-114	Room thermometer	No	

Description	Electronic room thermometer.	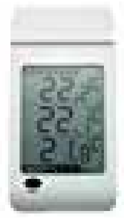
Use	Used to monitor room temperature in medicine storage locations.	
Clinical or User Specifications	The instrument records the minimum and maximum temperatures in a room, over the preceding period. The period is reset, once a daily reading has been noted. In the case of a week-end, the maximum and minimum recorded temperatures will have been recorded since the Friday.<br>Battery-operated, batteries need to be replaced at least annually.	
Accessories or Related Consumables	None.	
Care	Standard cleaning and disinfection.<br>Replace battery(ies) with same model button-type cells, when prompted to do so by the battery indicator.	
Supplier Warranty	1 year (excluding deliberate or accidental damage).	

Fire safety

Fire extinguisher

TECHNICAL SPECIFICATIONS page 690

Catalogue Number	Item Name	Available on Transversal Contract	Contract Number or Code
PHC-E-043	Fire extinguisher	No	

Description	Handheld firefighting equipment.	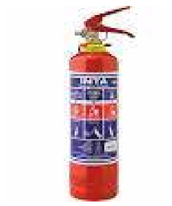
Use	For protection of patients, staff and state assets, against fire.	
Clinical or User Specifications	**Fire extinguisher**: there should be one Carbon dioxide extinguisher of 2,5kg for every 250sq. metres. Maximum distance of travel to reach the nearest extinguisher = 10m (Note, this is approximate, and will depend on the specific fire loading of the facility).	
Accessories or Related Consumables	Extinguishers supplied with mounting brackets and approved signage.	
Care	Standard cleaning and disinfection.	
Maintenance	After delivery, it is the legal responsibility of the building owner to arrange maintenance of fire extinguishers.<br>Extinguishers require annual or biannual service, depending on the model. Sticker showing date of last service and latest date for next service, to be affixed to each extinguisher.	
Supplier Warranty	N/A.	

Fire hose on reel

TECHNICAL SPECIFICATIONS page 691

Catalogue Number	Item Name	Available on Transversal Contract	Contract Number or Code
PHC-E-044	Fire hose on reel	No	

Description	High-pressure, double-wall non-kinking rubber hose, directly connected to mains supply with swivel connector. On reel.<br>Complete with nozzle.	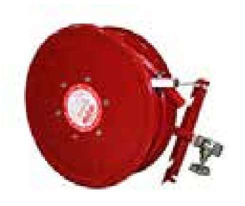
Use	For protection of patients, staff and state assets, against fire.	
Clinical or User Specifications	**Fire hose and reel**: 20mm x 30m hose with fittings. Maximum distance from any point on the same floor to the fire hose-reel = 30m. (Note, this is approximate, and will depend on the specific fire loading of the facility).	
Accessories or Related Consumables	Approved signage.	
Care	Standard cleaning and disinfection.	
Maintenance	Hose and reel require periodic pressure testing by a competent technician. Consult the local Fire Department.	
Supplier Warranty	N/A.	

Smoke alarm

TECHNICAL SPECIFICATIONS page 692

Catalogue Number	Item Name	Available on Transversal Contract	Contract Number or Code
PHC-E-045	Smoke alarm	No	

Description	Standard firefighting equipment.	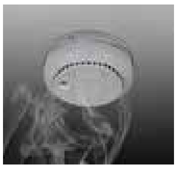
Use	For protection of patients, staff and state assets, against fire.	
Clinical or User Specifications	**Smoke alarm**: ceiling mounted, battery operated. Minimum 110 decibel loudness. Red LED to indicate that unit is operational. One per room that is completely enclosed when the door is shut.	
Accessories or Related Consumables	Supplied and installed complete with batteries.	
Care	Standard cleaning and disinfection.<br>Change battery in smoke alarm when the pilot light shows "Low Battery" state. Refer to departmental technical personnel.	
Supplier Warranty	1 year.	

Garden

Bins: waste, heavy duty

TECHNICAL SPECIFICATIONS page 693

Catalogue Number	Item Name	Available on Transversal Contract	Contract Number or Code
PHC-E-046	Bin: general waste, 240l black, with wheels	No	
PHC-E-047	Bin: healthcare risk waste, 140l red, with wheels	No	
PHC-E-048	Bin: general waste, 140l green, with wheels	No	
PHC-E-049	Bin: general waste, 90l black, no wheels	No	

Description	Bins for management of various categories of waste.	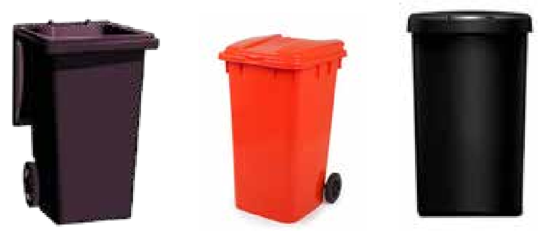
Use	Waste management, including garden waste.	
Clinical or User Specifications	All bins are made from durable plastic, with well-fitting, hinged lids. All except the 90l. bin have wheels to facilitate movement.<br>Black 240l bin is used for garden waste.<br>Red 140l bin is intended for medical waste.<br>Black 90l bin is intended for general waste.	
Accessories or Related Consumables		
Care	Standard cleaning and disinfection.	
Supplier Warranty	2 years.	

Broom

TECHNICAL SPECIFICATIONS page 694

Catalogue Number	Item Name	Available on Transversal Contract	Contract Number or Code
PHC-E-052	Broom: garden	No	

Description	Garden broom.	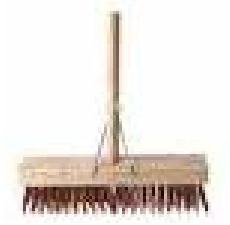
Use	For tidying external areas.	
Clinical or User Specifications	Heavy duty, large broom.<br>Wooden handle.<br>Stiff bristles.	
Accessories or Related Consumables	None.	
Care		
Supplier Warranty	None.	

Edge Trimmer/brush cutter: heavy duty

TECHNICAL SPECIFICATIONS page 695

Catalogue Number	Item Name	Available on Transversal Contract	Contract Number or Code
PHC-E-050	Edge trimmer/brush cutter: petrol, heavy duty	No	

Description	Edge trimmer/brush cutter: petrol, heavy duty.	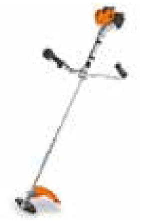
Use	Used to trim borders, clear pathways and weed beds, and trim grass in areas not suitable for lawnmowers.	
Clinical or User Specifications	Interchangeable heads: solid blade for heavier long grass and thin bushes, nylon line for general trimming and edging.	
Accessories or Related Consumables	Harness, safety glasses, hearing protectors, tool kit, manual.<br>Fuel mixing bottle.<br>100m recommended line.<br>Blade and line heads.	
Care	Clean after every use to prevent corrosion.<br>Clean air filter, and soak in petrol before each use.<br>Requires a mixture of petrol and 2-stroke oil, mixed in correct proportions as per supplier's instructions.<br>Have unit serviced during winter months every year.<br>Procure line as required.	
Supplier Warranty	2 years (excluding deliberate or accidental damage).	

Extension cable: electrical, garden

TECHNICAL SPECIFICATIONS page 696

Catalogue Number	Item Name	Available on Transversal Contract	Contract Number or Code
PHC-E-051	Extension cable: electrical, garden	No	

Description	Extension cable: electrical, garden.	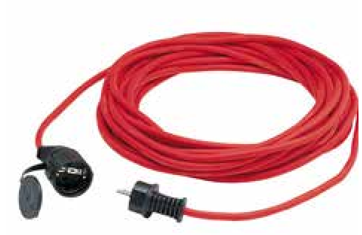
Use	Electrical extension for use with electric-powered lawn mower.	
Clinical or User Specifications	This extension has a normal 15A 3-pin plug on one end, and a Female Shuko connector on the other. The Shuko connector is safer than other types when used outdoors.	
Accessories or Related Consumables	None.	
Care	Coil without twisting when not in use.<br>If accidentally cut during use, have it properly mended.	
Supplier Warranty	1 year (excluding deliberate or accidental damage).	

Fork

TECHNICAL SPECIFICATIONS page 697

Catalogue Number	Item Name	Available on Transversal Contract	Contract Number or Code
PHC-E-053	Fork: garden, 4-pronged	No	

Description	Garden fork.	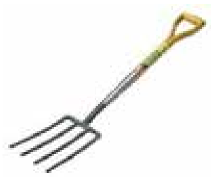
Use	For gardening.	
Clinical or User Specifications	Durable 4-prionged garden fork.	
Accessories or Related Consumables	None.	
Care	Clean after use to prevent metal corrosion.	
Supplier Warranty	1 year (excluding deliberate or accidental damage).	

Gardening set: 3-piece

TECHNICAL SPECIFICATIONS page 698

Catalogue Number	Item Name	Available on Transversal Contract	Contract Number or Code
PHC-E-059	Garden set: 3-piece	No	

Description	3-piece garden set.	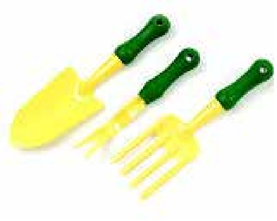
Use	For planting and tending to flower beds and pots.	
Clinical or User Specifications	Heavy duty hand trowel, hand fork and weed grabber.	
Accessories or Related Consumables	None.	
Care	Clean after use to prevent metal corrosion.	
Supplier Warranty	1 year (excluding deliberate or accidental damage).	

Hedge clipper: shears, manual

TECHNICAL SPECIFICATIONS page 699

Catalogue Number	Item Name	Available on Transversal Contract	Contract Number or Code
PHC-E-060	Hedge shears	No	

Description	Hedge shears. 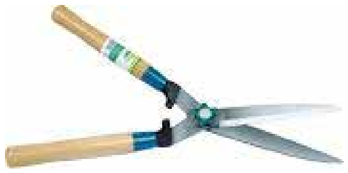
Use	For trimming bushes and hedges.
Clinical or User Specifications	Durable hedge-trimming shears.
Accessories or Related Consumables	None.
Care	Clean after use to prevent metal corrosion.
Supplier Warranty	1 year (excluding deliberate or accidental damage).

Hoe

TECHNICAL SPECIFICATIONS page 700

Catalogue Number	Item Name	Available on Transversal Contract	Contract Number or Code
PHC-E-061	Hoe	No	

Description	Hoe.	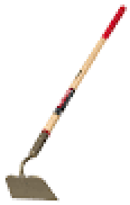
Use	For planting and weed removal.	
Clinical or User Specifications	Heavy duty hoe.	
Accessories or Related Consumables	None.	
Care	Clean after use to prevent metal corrosion.	
Supplier Warranty	1 year (excluding deliberate or accidental damage).	

Hosepipe: garden

TECHNICAL SPECIFICATIONS page 701

Catalogue Number	Item Name	Available on Transversal Contract	Contract Number or Code
PHC-E-062	Hosepipe: garden	No	

Description	Hosepipe: Garden, with tap fittings.	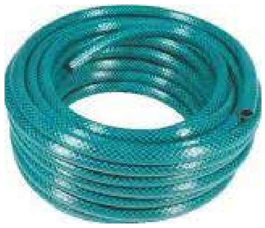
Use	Used to water plants and for external cleaning.	
Clinical or User Specifications	50m length, 20mm diameter reinforced plastic.	
Accessories or Related Consumables	Tap connector, quick-connector, multi-pattern spray head, wall-mounted hose holder, spare washer kit, hose joiner (for mending), two good quality worm-drive clamps.	
Care	Coil loosely on wall-mounted holder when not in use.	
Supplier Warranty	1 year (excluding deliberate or accidental damage).	

Lawn mower: Electric, heavy duty

TECHNICAL SPECIFICATIONS page 702

Catalogue Number	Item Name	Available on Transversal Contract	Contract Number or Code
PHC-E-063	Lawn mower: electric, heavy duty	No	

Description	Lawn mower with electric motor, suitable for smaller premises.	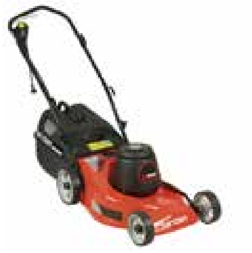
Use	For maintaining lawns, up to about 4000m^2.	
Clinical or User Specifications	The maximum allowable cable length is 40m, i.e. the cable supplied with the mower plus a 20m extension. If this combined length does not permit the lawnmower to reach all the borders of the lawn, a petrol-driven model should be selected.	
Accessories or Related Consumables	20m cable (replaceable if damaged), with a male Shuko plug at one end. This cable is designed to be used with an extension cable with a female Shuko plug. Shuko plugs have been specified, as they are safer and more durable for this type of use.	
Care	The machine must be cleaned after each use, to prevent rusting of metal components.<br>Keep cables from twisting excessively. Wrap the cable on the machine as instructed, after use.<br>If cables get frayed or cut, have them replaced. Otherwise there is a danger of electric shock.<br>Blades must be replaced at least once a year.	
Supplier Warranty	2 years (excluding wearable items (cables and blades) and deliberate or accidental damage).	

Lawn mower: petrol, heavy duty

TECHNICAL SPECIFICATIONS page 703

Catalogue Number	Item Name	Available on Transversal Contract	Contract Number or Code
PHC-E-064	Lawnmower: petrol, heavy duty	No	

Description	Lawnmower: petrol, heavy duty.	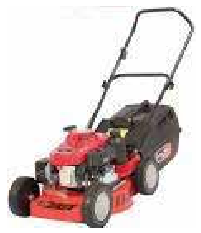
Use	Used to trim borders, clear pathways and weed beds, and trim grass in areas not suitable for lawnmowers.	
Clinical or User Specifications	This model is for use on larger premises (>4000 sq. metres), or where electric power is unreliable.<br>Can tolerate some unevenness of ground, but not large stones (use brush-cutter).	
Accessories or Related Consumables	Safety glasses, hearing protectors, tool kit, manual.<br>Spare air cleaner.<br>Spare set of blades.<br>Petrol "jerry can".	
Care	Clean after every use to prevent corrosion.<br>Clean air filter, and soak in petrol before each use. Swop with spare air cleaner during heavy use. Wash and dry after use.<br>Requires regular petrol.<br>Engine oil level should be checked before each use, topped up as required.<br>Oil must be changed as per supplier's instructions.<br>Have unit serviced during winter months every year.<br>Change blades when worn beyond limit specified by supplier.	
Supplier Warranty	2 years (excluding deliberate or accidental damage).	

Pick mattock

TECHNICAL SPECIFICATIONS page 704

Catalogue Number	Item Name	Available on Transversal Contract	Contract Number or Code
PHC-E-065	Pick mattock	No	

Description	Pick mattock.	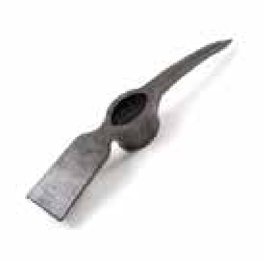
Use	For loosening ground, cutting and chopping.	
Clinical or User Specifications	Diamond point is used to break up ground, mattock blade breaks up ground and cuts edges of a hole.	
Accessories or Related Consumables	None.	
Care		
Supplier Warranty	1 year on head and handle (excluding deliberate and accidental damage).	

Rake: leaf and grass, heavy duty

TECHNICAL SPECIFICATIONS page 705

Catalogue Number	Item Name	Available on Transversal Contract	Contract Number or Code
PHC-E-054	Rake: leaf and grass, heavy duty	No	

Description	Leaf/grass rake.	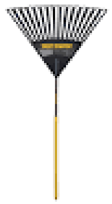
Use	For raking up leaves and lawn clippings.	
Clinical or User Specifications	Durable rake for gathering leaves or lawn clippings.	
Accessories or Related Consumables	None.	
Care	Do not use for other purposes – teeth are flexible and fragile.<br>Clean after use to prevent metal corrosion.	
Supplier Warranty	1 year (excluding deliberate or accidental damage).	

Rake: steel, heavy duty

TECHNICAL SPECIFICATIONS page 706

Catalogue Number	Item Name	Available on Transversal Contract	Contract Number or Code
PHC-E-055	Rake: steel, heavy duty	No	

Description	Garden rake.	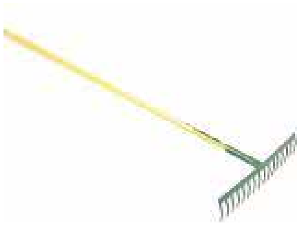
Use	For breaking up earth clods, removal of stones, and levelling of ground.	
Clinical or User Specifications	Durable garden rake.<br>All-metal construction.	
Accessories or Related Consumables	None.	
Care	Clean after use to prevent metal corrosion.	
Supplier Warranty	1 year (excluding deliberate or accidental damage).	

Shovel: square mouth

TECHNICAL SPECIFICATIONS page 707

Catalogue Number	Item Name	Available on Transversal Contract	Contract Number or Code
PHC-E-056	Shovel: square mouth	No	

Description	Garden shovel: square mouth.	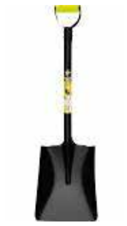
Use	For gardening.	
Clinical or User Specifications	Durable garden shovel.	
Accessories or Related Consumables	None.	
Care	Clean after use to prevent metal corrosion.	
Supplier Warranty	1 year (excluding deliberate or accidental damage).	

Slasher

TECHNICAL SPECIFICATIONS page 708

Catalogue Number	Item Name	Available on Transversal Contract	Contract Number or Code
PHC-E-057	Slasher: garden	No	

Description	Garden slasher.	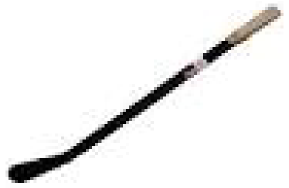
Use	For hacking long grass and weeds.	
Clinical or User Specifications	Tempered steel blade with ergonomic grip in wood or moulded nylon.	
Accessories or Related Consumables	None.	
Care	Clean after use to prevent metal corrosion.	
Supplier Warranty	1 year (excluding deliberate or accidental damage).	

Spade

TECHNICAL SPECIFICATIONS page 709

Catalogue Number	Item Name	Available on Transversal Contract	Contract Number or Code
PHC-E-058	Garden spade	No	

Description	Garden spade.	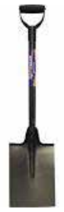
Use	For gardening.	
Clinical or User Specifications	Durable 4-pronged garden fork.	
Accessories or Related Consumables	None.	
Care	Clean after use to prevent metal corrosion.	
Supplier Warranty	1 year (excluding deliberate or accidental damage).	

Watering can

TECHNICAL SPECIFICATIONS page 710

Catalogue Number	Item Name	Available on Transversal Contract	Contract Number or Code
PHC-E-066	Watering can	No	

Description	Watering can.	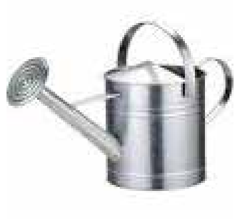
Use	Used to water plants.	
Clinical or User Specifications	Galvanised iron watering can, with detachable rose for sprinkling seedlings, or for applying diluted chemicals.	
Accessories or Related Consumables	Detachable rose.	
Care	Dry after every use to prevent corrosion.	
Supplier Warranty	1 year (excluding deliberate or accidental damage).	

Wheelbarrow

TECHNICAL SPECIFICATIONS page 711

Catalogue Number	Item Name	Available on Transversal Contract	Contract Number or Code
PHC-E-067	Wheel barrow	No	

Description	Wheel barrow.	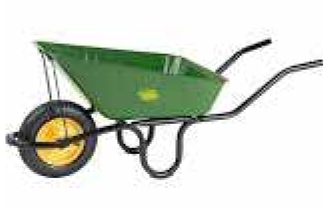
Use	For gardening and general maintenance work.	
Clinical or User Specifications	Heavy-duty wheelbarrow with solid rubber tyre.	
Accessories or Related Consumables	None.	
Care	Clean after use to prevent metal corrosion.	
Supplier Warranty	1 year (excluding deliberate or accidental damage).	

Information and Communications Technology

NO TECHNICAL SPECIFICATIONS NONE

Catalogue Number	Item Name	Available on Transversal Contract	Contract Number or Code
PHC-E-088	Personal computer	National/Provincial contracts	
PHC-E-089	Multifunction printer/scanner/copier/fax	Provincial contracts	
PHC-E-090	SMS results printer	Supplied by NHLS	
PHC-E-091	SVS scanner	Supplied as part of Pharmaceutical Supply Chain initiative	

These items are from external suppliers. Specifications and contract arrangements are unique to provinces and suppliers.

The items are listed here for completeness and cross-reference.

Kitchen

Kettle: electric

TECHNICAL SPECIFICATIONS page 712

Catalogue Number	Item Name	Available on Transversal Contract	Contract Number or Code
PHC-E-092	Kettle: electric	No	

Description	Electric kettle.	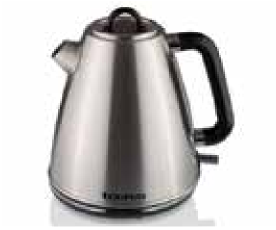
Use	For boiling small volumes of water.	
Clinical or User Specifications	Stainless steel body and lid. Auto switch-off.<br>Cordless type.<br>1,7l.<br>Rapid-boiling. 2000Watts.	
Accessories or Related Consumables	None.	
Care	De-scale 6-monthly to maintain efficiency – more often in hard water areas.	
Supplier Warranty	1 year (excluding deliberate or accidental damage).	

Rack: dish

TECHNICAL SPECIFICATIONS page 713

Catalogue Number	Item Name	Available on Transversal Contract	Contract Number or Code
PHC-E-093	Rack: dish	No	

Description	Dish drying rack.	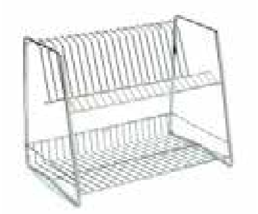
Use	For air-drying of washed crockery.	
Clinical or User Specifications	Stainless steel counter-top rack.	
Accessories or Related Consumables	None.	
Care	Standard cleaning and disinfection.	
Supplier Warranty	1 year (excluding deliberate or accidental damage).	

Rack: cutlery

TECHNICAL SPECIFICATIONS page 713

Catalogue Number	Item Name	Available on Transversal Contract	Contract Number or Code
PHC-E-094	Rack: cutlery	No	

Description	Cutlery drying rack.	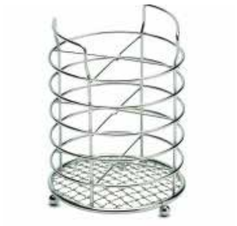
Use	For air-drying of washed cutlery.	
Clinical or User Specifications	Stainless steel counter-top rack.	
Accessories or Related Consumables	None.	
Care	Standard cleaning and disinfection.	
Supplier Warranty	2 years (excluding deliberate or accidental damage).	

Urn: electric

TECHNICAL SPECIFICATIONS page 714

Catalogue Number	Item Name	Available on Transversal Contract	Contract Number or Code
PHC-E-096	Urn: electric	No	

Description	Urn, electric.	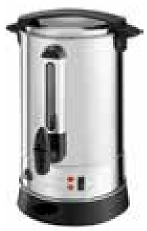
Use	For boiling water in larger volumes than possible with a kettle.	
Clinical or User Specifications	All stainless steel construction.<br>Thermostat to maintain water at desired temperature.<br>Metal spigot tap.<br>Has safety cut-out and anti boil-dry.	
Accessories or Related Consumables	None.	
Care	De-scale 6-monthly to maintain efficiency – more often in hard water areas.<br>Disconnect from mains electricity supply if empty.	
Supplier Warranty	2 years (excluding deliberate or accidental damage).	

Watercooler: free-standing

TECHNICAL SPECIFICATIONS page 715

Catalogue Number	Item Name	Available on Transversal Contract	Contract Number or Code
PHC-E-097	Watercooler: free-standing	No	

Description	Free-standing watercooler.	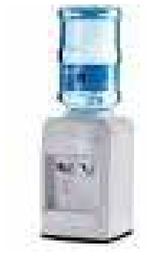
Use	To provide cool drinking water in waiting areas.	
Clinical or User Specifications	Free-standing floor-mounted unit.<br>The unit fits standard 15l bottles.<br>Dispenses cold water via a push-to-fill tap.<br>Drip tray to prevent spillage onto floor.<br>Disposable paper cups – no polystyrene.	
Accessories or Related Consumables	Disposable paper cups. PHC-C-164.	
Care	Replace water container when empty.	
Maintenance	Bottled watercoolers require sanitising every three months. Contact departmental technical personnel or supplier.	
Supplier Warranty	2 years (excluding deliberate or accidental damage).	

Miscellaneous

Bins: brazier

TECHNICAL SPECIFICATIONS page 716

Catalogue Number	Item Name	Available on Transversal Contract	Contract Number or Code
PHC-E-159	Brazier bin 105x135x75		
PHC-E-160	Brazier bin 105x190x75		
PHC-E-161	Brazier bin 140x210x130		
PHC-E-162	Brazier bin 140x280x130		

Description	Small open storage bins. 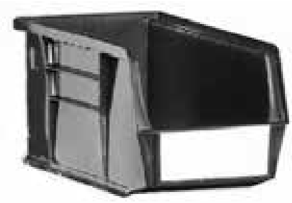
Use	For the storage of packages of medicines in an orderly sorted manner.
Clinical or User Specifications	Made of durable plastic.<br>Range of colours and also clear plastic.<br>Can be placed on shelves, or wall-mounted.<br>Front edge can be labelled.
Accessories or Related Consumables	
Care	Standard cleaning method.
Supplier Warranty	N/A

Clock: wall

TECHNICAL SPECIFICATIONS page 717

Catalogue Number	Item Name	Available on Transversal Contract	Contract Number or Code
PHC-E-099	Clock: wall	No	

Description	Wall clock, analogue.	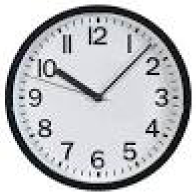
Use	To display time in consulting rooms and waiting areas.	
Clinical or User Specifications	250-300mm. diameter, round. Battery-operated.<br>Body of high-impact plastic, with clear dial cover.<br>Dial plate white with bold black Arabic numerals.<br>Hour and minute hands black, sweep second hand may be black or red.<br>Built-in screw-head retainer for wall-mounting.	
Accessories or Related Consumables	Wall-plug and screw, for mounting on wall.<br>Complete with battery.	
Care	Installation by supplier or departmental technical personnel.<br>Replace battery with cell of same size, when required.	
Supplier Warranty	1 year.	

Complaints and suggestions box

TECHNICAL SPECIFICATIONS page 718

Catalogue Number	Item Name	Available on Transversal Contract	Contract Number or Code
PHC-E-100	Complaints and suggestions box	No	

Description	Complaints and suggestions box.<br>Frosted white, all-acrylic plastic construction box with lid.	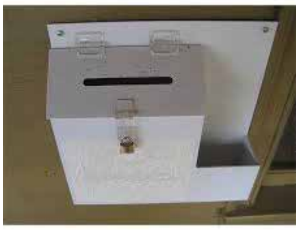
Use	Locked box to receive compliments, complaints or suggestions from patients.	
Clinical or User Specifications	White plastic backboard, with attached box with lockable lid, and open compartment for note paper. Lid has a slot, for insertion of written notes.<br>Lid is kept locked.<br>Unlocked weekly, to access contents for review.<br>The backboard is printed with labels describing the use of the box, in three official languages.<br>Text colour in Provincial colours.<br>Permanently mounted in a prominent location in the main waiting area.	
Accessories or Related Consumables	Brass combination-type lock.	
Care	To be mounted to the wall by supplier or departmental technical personnel.<br>Standard cleaning and disinfection.<br>Replenish notepaper and writing instruments as required.	
Supplier Warranty	1 year.	

Gun safe

TECHNICAL SPECIFICATIONS page 719

Catalogue Number	Item Name	Available on Transversal Contract	Contract Number or Code
PHC-E-101	Gun safe	No	

Description	Gun safe.	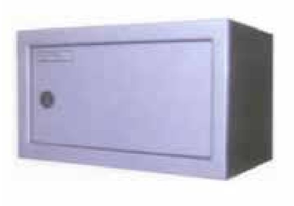
Use	For secure storage of handguns in the security office, prior to a patient's entry into the PHC facility.	
Clinical or User Specifications	Wall-mounted – must be mounted on a double (i.e. 18cm) solid masonry wall.<br>Quality and thickness of materials are laid down in the Fire Arm Control Act 60 of 2000, and its regulations. The safe must be certified to comply.<br>The safe has 2 locks, with different keys. After securing the firearm in the safe, one key is given to the firearm owner, the other is retained by the security officer on duty.	
Accessories or Related Consumables	2 sets of keys.	
Care	Installation by departmental technical personnel, in accordance with the regulations.<br>Spare keys must be securely stored, in accordance with key policy.	
Supplier Warranty	5 years on all components.	

Notice board: wall-mounted

TECHNICAL SPECIFICATIONS page 720

Catalogue Number	Item Name	Available on Transversal Contract	Contract Number or Code
PHC-E-102	Notice board: A3 size	No	
PHC-E-103	Notice board: A2 size		

Description	Notice board, wall-mounted.	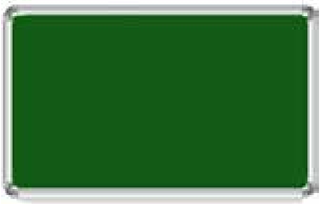
Use	For displaying ad-hoc information, for the benefit of the public or the staff.	
Clinical or User Specifications	Aluminium frame. Fibreboard backing. Faced with polyester, non-shiny fabric. Dark colour preferred for contrast with notices.<br>Notices affixed with chart pins.	
Accessories or Related Consumables	Wall-mounting hardware.<br>Box of multi-coloured plastic push pins.	
Care	Installation by supplier or departmental technical personnel.	
Supplier Warranty	2 years on all components.	

Pamphlet-holding boxes

TECHNICAL SPECIFICATIONS page 721

Catalogue Number	Item Name	Available on Transversal Contract	Contract Number or Code
PHC-E-104	Pamphlet-holding boxes	No	

Description	Pamphlet-holding boxes, wall-mounted.	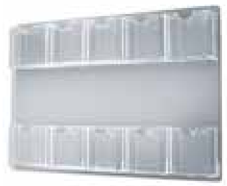
Use	For displaying and dispensing patient information leaflets in reception and waiting areas.	
Clinical or User Specifications	White backing board.<br>Individual clear acrylic pockets, to hold A4 size pamphlets or smaller.<br>2 horizontal rows of 4 pockets.	
Accessories or Related Consumables	Wall-mounting hardware.	
Care	Installation by supplier or departmental technical personnel.	
Supplier Warranty	2 years on all components.	

Poster display boards

TECHNICAL SPECIFICATIONS page 722

Catalogue Number	Item Name	Available on Transversal Contract	Contract Number or Code
PHC-E-105	Poster display boards: A1 size	No	
PHC-E-106	Poster display boards: A0 size	No	

Description	Poster display boards, wall-mounted.	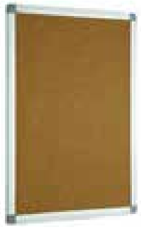
Use	For displaying patient information posters in reception and waiting areas, without damaging wall surfaces.	
Clinical or User Specifications	Aluminium frame. Fibreboard backing. Faced with polyester, non-shiny fabric. Dark colour preferred for contrast with notices.<br>Posters affixed with clips or push pins.	
Accessories or Related Consumables	Wall-mounting hardware.<br>Box of multi-coloured plastic push pins.	
Care	Installation by supplier or departmental technical personnel.	
Supplier Warranty	2 years on all components.	

Whiteboard

TECHNICAL SPECIFICATIONS page 723

Catalogue Number	Item Name	Available on Transversal Contract	Contract Number or Code
PHC-E-107	Whiteboard, 1200x1000	No	
PHC-E-108	Whiteboard, 1800x1200	No	
PHC-E-109	Whiteboard 3000x1200	No	

Description	Whiteboards, wall-mounted.	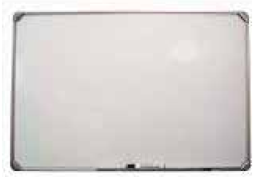
Use	For temporary recording of proceedings, notes, meeting minutes, discussion points and diagrams.	
Clinical or User Specifications	Aluminium frame.<br>Magnetic, white, epoxy-coated, sheet metal, writing surface.	
Accessories or Related Consumables	Wall-mounting hardware.<br>Whiteboard felt eraser.	
Care	Installation by supplier or departmental technical personnel.	
Supplier Warranty	2 years on all components.	

B
instruments

RESUSCITATION 182

- Magill forceps: adult 182
- Magill forceps: child 183
- Introducer (intubation stylet): adult 184
- Introducer (intubation stylet): child 185
- Scissors: rescue 186

SURGICAL 187

- Forceps: artery, curved 187
- Forceps: artery, straight 188
- Forceps: non-toothed 189
- Forceps: toothed 190
- Forceps: mosquito, curved 191
- Forceps: mosquito, straight 192
- Needle holder 193
- Scalpel handle: long, No. 3 194
- Scissors: dissecting 195
- Scissors: stitch 196
- Stitch tray: small 197
- Swab holder 198
- Syringe: dental 199

OBSTETRIC 200

- Basin 200
- Bowl: large 201
- Bowl: small 202
- Kidney dish 203
- Scissors: episiotomy 204
- Scissors: umbilical 205

ORAL HEALTH 206

- Amalgam carrier: plastic 206
- Amalgam plugger 208
- Ball burnisher 209
- Bur Block 210
- Bur brush 211
- Carver: Amalgam 212
- Carver: wax 213
- Dappen dish 214
- Dental Explorer 215
- Dental Probe: periodontal 216
- Elevator: root, apical 217
- Elevator: root, Cryers 218
- Elevator: root, Warwick-James 219
- Excavator: dental 220
- Flat plastic 221
- Forceps: tongue 222
- Forceps: tooth extracting, Lower Anteriors Adult 223
- Forceps: tooth extracting, Lower Bicuspids 224
- Forceps: tooth extracting, Lower Canines Adult 225
- Forceps: tooth extracting, Lower Molars 226
- Forceps: tooth extracting, Lower roots and Crowded Incisors 227
- Forceps: tooth extracting, Upper Anteriors and Canines 228
- Forceps: tooth extracting, Upper Bicuspids and Roots 229
- Forceps: tooth extracting, Upper Molars, Left 230
- Forceps: tooth extracting, Upper Molars, Right 231
- Forceps: tooth extracting, Upper Molars, Right 232
- Forceps: tooth extracting, Upper roots 233
- Forceps: tooth extracting, Upper roots 234
- Forceps: tooth extracting, Upper roots 235
- Forceps: tooth extracting, Upper roots, Small 237
- Forceps: tooth extracting, Lower molars, child 238
- Forceps: tooth extracting, Lower teeth and roots, child 239
- Forceps: tooth extracting, Upper Incisors and Canines, child 240
- Forceps: tooth extracting, Upper Molars, child 241
- Forceps: tooth extracting, Upper teeth and roots, Molars, child 242
- Forceps: wire ligature 243
- Gag (mouth prop) 244
- Holder: Bib 245
- Holder: cotton pellet 246
- Matrix retainer 247
- Needle holder 249
- Periodontal Hoe 250
- Rongeur: dental 251
- Scaler, Dental 252
- Scissors: ligature 253
- Slab: mixing, glass 254
- Spatula: Cement 255
- Syringe: dental, aspirating 256
- Trimmer: Gingival margin 257
- Tweezers: Cotton and Dressing 258

REHABILITATION 259

Physical therapy 259

- Scissors: standard 259
- Scissors: splinting 260

Audiology/speech therapy 261

- Cerumen management kit 261
- Curette: cerumen, plastic 262
- Curette: cerumen, lighted 263
- Ear loop: Bileau, small 264
- Forceps: crocodile 265
- Syringe: impression 266

Resuscitation

Magill forceps: adult

NO TECHNICAL SPECIFICATIONS NONE

Catalogue Number	Item Name	Available on Transversal Contract	Contract Number or Code
PHC-I-023	Magill forceps: adult	RT 252	RT 252-01-122 ME

Functional Specifications	Forceps, endotracheal, Magill`s.<br>Serrated, fenestrated jaws, screw joint.<br>Stainless steel with matte finish.<br>Length: 240mm.<br>Autoclaveable, Specification number: 1691.
Use	Used to assist in the insertion of an endotracheal tube in an adult patient. Allows the body of the ET tube to be gripped and manipulated , without interfering with the line of sight while using a laryngoscope.
Applicable Standards	ISO 13402:1995, ISO7151:1988, ISO 7153-1:2016, ISO900101 & ISO13485.
Performance Specifications	15-year service life.
Warranties	2 years.
Note to Procurement	

Magill forceps: child

NO TECHNICAL SPECIFICATIONS NONE

Catalogue Number	Item Name	Available on Transversal Contract	Contract Number or Code
PHC-I-024	Magill forceps: child	RT 252	RT 252-01-122 ME

Functional Specifications	Forceps, endotracheal, Magill`s.<br>Serrated, fenestrated jaws, screw joint.<br>Stainless steel with matte finish.<br>Length: 200mm.<br>Autoclaveable, Specification number: 1699.
Use	Used to assist in the insertion of an endotracheal tube in a paediatric patient. Allows the body of the ET tube to be gripped and manipulated , without interfering with the line of sight while using a laryngoscope.
Applicable Standards	ISO 13402:1995, ISO7151:1988, ISO 7153-1:2016, ISO900101 & ISO13485.
Performance Specifications	15-year service life.
Warranties	2 years.
Note to Procurement	

Introducer (intubation stylet): adult

NO TECHNICAL SPECIFICATIONS NONE

Catalogue Number	Item Name	Available on Transversal Contract	Contract Number or Code
PHC-I-025	Introducer (intubation stylet): adult	RT 13	RT 13-51-004

Functional Specifications	Size: Adult -To fit Endotracheal tube size 6.5-11.0mm. Length:340mm-365mm.<br>Manufactured from low friction atraumatic outer coating around a malleable aluminium core.<br>Soft atraumatic tip, U-bend or other mechanism at proximal end to limit depth.<br>Latex free. Clinically clean packaging. Individually packed in peel pouch.
Use	The stylet is used to modify the curve of the tip of an endotracheal tube, to facilitate insertion between the vocal cords.<br>The stylet is placed inside the ET tube, with the tip protruding.<br>The tube and enclosed stylet are then bent to the desired shape.<br>The stylet is withdrawn after the ET tube has been inserted.
Applicable Standards	
Performance Specifications	
Warranties	2-year shelf life.
Note to Procurement	

Introducer (intubation stylet): child

NO TECHNICAL SPECIFICATIONS
NONE

Catalogue Number	Item Name	Available on Transversal Contract	Contract Number or Code
PHC-I-026	Introducer (intubation stylet): child	RT 13	RT 13-51-002

Functional Specifications	Size: Paediatric -To fit Endotracheal tube size 4.0-4.5mm. Length: 275 mm.<br>Manufactured from low friction atraumatic outer coating around a malleable aluminium core.<br>Soft atraumatic tip, U-bend or other mechanism at proximal end to limit depth.<br>Latex free. Clinically clean packaging. Individually packed in peel pouch.
Use	The stylet is used to modify the curve of the tip of an endotracheal tube, to facilitate insertion between the vocal cords.<br>The stylet is placed inside the ET tube, with the tip protruding.<br>The tube and enclosed stylet are then bent to the desired shape.<br>The stylet is withdrawn after the ET tube has been inserted.
Applicable Standards	
Performance Specifications	
Warranties	2-year shelf life.
Note to Procurement	

Scissors: rescue

NO TECHNICAL SPECIFICATIONS NONE

Catalogue Number	Item Name	Available on Transversal Contract	Contract Number or Code
PHC-I-020	Scissors: rescue	RT 4	RT 4-05-051 ME

Functional Specifications	Heavy duty rescue scissors suitable for Emergency Medical Care and Rescue of patients in the pre-hospital environment.<br>The scissors must not be smaller or larger than 15cm long. The handle must be ergonomic to allow for a solid grip. The blades must be constructed from high quality 420 surgical grade stainless steel.<br>Autoclaveable.<br>Blades and handles to have non-stick surface.
Use	These scissors are used to rapidly remove clothing and strictures from patients, in emergency situations when there is no time for conventional undressing.
Applicable Standards	ISO 13402:1995, ISO 7741:1986, ISO 7153-1:2016, IS0900101 & IS013485.
Performance Specifications	The blades must be able to cut most materials including clothing and security belts.<br>10-year service life.
Warranties	2 years.
Note to Procurement	

Surgical

NO TECHNICAL SPECIFICATIONS
NONE

Forceps: artery, curved

Catalogue Number	Item Name	Available on Transversal Contract	Contract Number or Code
PHC-I-004	Forceps: artery, curved	RT 252	RT 252-01-085 ME

Functional Specifications	Forceps, artery, Kocher.<br>Curved, box joint.<br>Stainless steel.<br>Length: 200mm.<br>Autoclaveable.<br>Specification number: 6330.
Use	Medium-sized locking forceps, used to hold tissue in desired position, or to achieve haemostasis.<br>With curved jaws.<br>Ratcheting locks. Finger rings both shanks.
Applicable Standards	ISO 13402:1995, ISO 7151:1988, ISO 7153-1:2016, ISO900101 & ISO13485.
Performance Specifications	15-year service life.
Warranties	2 years.
Note to Procurement	

Forceps: artery, straight

NO TECHNICAL SPECIFICATIONS
NONE

Catalogue Number	Item Name	Available on Transversal Contract	Contract Number or Code
PHC-I-003	Forceps: artery, straight	RT 252	RT 252-01-084 ME

Functional Specifications	Forceps, artery, Kocher.<br>Straight, box joint.<br>Stainless steel.<br>Length: 200mm.<br>Autoclaveable.<br>Specification number: 4636.
Use	Medium-sized locking forceps, used to hold tissue in desired position, or to achieve haemostasis.<br>With straight jaws.<br>Ratcheting locks. Finger-rings both shanks.
Applicable Standards	ISO 13402:1995, ISO 7151:1988, ISO 7153-1:2016, ISO900101 & ISO13485.
Performance Specifications	15-year service life.
Warranties	2 years.
Note to Procurement	

Forceps: non-toothed

NO TECHNICAL SPECIFICATIONS NONE

Catalogue Number	Item Name	Available on Transversal Contract	Contract Number or Code
PHC-I-002	Forceps: non-toothed	RT 252	RT 252-01-113 ME

Functional Specifications	Forceps, dissecting/dressing.<br>Standard pattern.<br>Block joint.<br>Non-toothed serrated jaws.<br>Grip with transverse grooves.<br>Stainless steel.<br>Length: 180mm.<br>Autoclaveable.<br>Specification number: 1520.
Use	Medium-sized dissecting forceps for holding and bringing into approximation friable tissues.<br>Non-toothed.
Applicable Standards	ISO 13402:1995, ISO 7151:1988, ISO 7153-1:2016, ISO900101 & ISO13485.
Performance Specifications	15-year service life.
Warranties	2 years.
Note to Procurement	

Forceps: toothed

NO TECHNICAL SPECIFICATIONS
NONE

Catalogue Number	Item Name	Available on Transversal Contract	Contract Number or Code
PHC-I-001	Forceps: toothed	RT 252	RT 252-01-109 ME

Functional Specifications	Dissecting forceps, toothed. Bonney pattern.<br>Block joint.<br>Serrated jaws, 1 x 2 teeth.<br>Roughened handle.<br>Stainless steel with matte finish.<br>Length: 180mm.<br>Autoclaveable.<br>Specification number: 1511.
Use	Medium-sized dissecting forceps for holding and bringing into approximation friable tissues.<br>Interlocking teeth.
Applicable Standards	ISO 13402:1995, ISO 7151:1988, ISO 7153-1:2016, ISO900101 & ISO13485.
Performance Specifications	15-year service life.
Warranties	2 years.
Note to Procurement	

Forceps: mosquito, curved

NO TECHNICAL SPECIFICATIONS NONE

Catalogue Number	Item Name	Available on Transversal Contract	Contract Number or Code
PHC-I-006	Forceps: mosquito, curved	RT 252	RT 252-01-312 ME

Functional Specifications	Forceps, artery, Halsted-mosquito.<br>Curved, box joint.<br>Fine tip.<br>Stainless steel.<br>Length: 125mm.<br>Autoclaveable.
Use	Small locking forceps, to secure haemostasis in small-vessel bleeds.<br>With curved jaws.<br>Ratcheting locks.<br>Finger-rings both shanks.
Applicable Standards	ISO 13402:1995, ISO 7151:1988, ISO 7153-1:2016, ISO900101 & ISO13485.
Performance Specifications	15-year service life.
Warranties	2 years.
Note to Procurement	

Forceps: mosquito, straight

NO TECHNICAL SPECIFICATIONS NONE

Catalogue Number	Item Name	Available on Transversal Contract	Contract Number or Code
PHC-I-005	Forceps: mosquito, straight	RT 252	RT 252-01-079 ME

Functional Specifications	Forceps, artery, Halsted-mosquito.<br>Straight, box joint.<br>Stainless steel.<br>Length: 125mm.<br>Autoclaveable.<br>Specification number: 1489.
Use	Small locking forceps, to secure haemostasis in small-vessel bleeds.<br>With straight jaws.<br>Ratcheting lock.<br>Finger-rings both shanks.
Applicable Standards	ISO 13402:1995, ISO 7151:1988, ISO 7153-1:2016, ISO900101 & ISO13485.
Performance Specifications	15-year service life.
Warranties	2 years.
Note to Procurement	

Needle holder

NO TECHNICAL SPECIFICATIONS NONE

Catalogue Number	Item Name	Available on Transversal Contract	Contract Number or Code
PHC-I-008	Needle holder	RT 252	RT 252-01-173 ME

Functional Specifications	Needle holder, Mayo-Hegar.<br>Box joint.<br>Tungsten Carbide Jaw Inserts.<br>Stainless steel.<br>Length: 150mm.<br>Autoclaveable.<br>Specification number: 1568.
Use	Medium-sized universal-pattern needle holder. Ratcheting lock.<br>Tungsten carbide inserts in the jaws, for long life.<br>Finger-ring on both shanks.
Applicable Standards	ISO 13402:1995, ISO 7151:1988, ISO 7153-1:2016, ISO900101 & ISO13485.
Performance Specifications	15-year service life.
Warranties	2 years.
Note to Procurement	

Scalpel handle: long, No. 3

NO TECHNICAL SPECIFICATIONS NONE

Catalogue Number	Item Name	Available on Transversal Contract	Contract Number or Code
PHC-I-007	Scalpel handle: long, No.3	RT 252	RT 252-01-015 ME

Functional Specifications	Scalpel handle.<br>Type: Long, No: 3.<br>Scalpel Handle – must be compatible with blades number: RT 252-01-005ME, RT 252-01-006 ME, RT 252-01-007 ME AND RT 252-01-008 ME.<br>Autoclaveable.<br>Specification number: 1605.
Use	Used in conjunction with a matching blade, for incisions, excisions, and drainage.
Applicable Standards	ISO 13402:1995, ISO 7740:1985, ISO 7153-1:2016, ISO900101 & ISO13485.
Performance Specifications	15-year service life.
Warranties	2 years.
Note to Procurement	ITEMS NUMBERS: (RT 252-01-005 ME, RT 252-01-006 ME, RT 252-01-007 ME, RT 252-01-008 ME, RT 252-01-014 ME, RT 252-01-015 ME, RT 252-01-018 ME AND RT 252-01-019 ME) will be evaluated as series in terms of 16.4 paragraph in the Special Conditions of Contract.

NO TECHNICAL SPECIFICATIONS NONE

Scissors: dissecting

Catalogue Number	Item Name	Available on Transversal Contract	Contract Number or Code
PHC-I-027	Scissors: dissecting	RT252-2015	RT252-01-227ME

Functional Specifications	Dissecting scissors. McIndoe/Metzenbaum pattern. Stainless steel.<br>Screw joint, double straight shanks. Finger-ring shanks.<br>Both blades blunt.<br>Curved on flat.<br>Length 180mm.<br>Autoclaveable.<br>Specification number: 6162.
Use	For intra-oral dissection.
Applicable Standards	ISO 13402:1995, ISO 7741:1986, ISO 7153-1:2016, ISO900101 & ISO13485.
Performance Specifications	15- year service life.
Warranties	2 years (excluding deliberate or accidental damage).
Note to Procurement	

Scissors: stitch

NO TECHNICAL SPECIFICATIONS
NONE

Catalogue Number	Item Name	Available on Transversal Contract	Contract Number or Code
PHC-I-009	Scissors: stitch	RT 252	RT 252-01-240 ME

Functional Specifications	Scissors, suture, Heath.<br>Screw joint, double straight shanks.<br>Sharp/sharp.<br>Length: 150mm.<br>Autoclaveable.<br>Stainless steel, matte finish.<br>Specification number: 6162.
Use	Medium-sized handles, but small sharp blades, for insertion into suture before cutting.<br>The selected model has straight shanks, as opposed to the more common double-curvature pattern.<br>Finger-ring shanks.
Applicable Standards	ISO 13402:1995, ISO 7741:1986, ISO 7153-1:2016, ISO900101 & ISO13485.
Performance Specifications	10-year service life.
Warranties	2 years.
Note to Procurement	

Stitch tray: small

NO TECHNICAL SPECIFICATIONS NONE

Catalogue Number	Item Name	Available on Transversal Contract	Contract Number or Code
PHC-I-010	Stitch tray: small	No	

Functional Specifications	Perforated tray with lid, minimum dimensions 26X12X6cm.<br>Stainless steel.<br>Autoclaveable.
Use	Wire mesh- or perforated-sheet stainless steel tray with lid.<br>Used to assemble the various instruments into a pack, prior to sterilisation.
Applicable Standards	ISO 13402:1995, ISO 7153-1:2016, ISO900101 & ISO13485.
Performance Specifications	15-year service life.
Warranties	2 years.
Note to Procurement	

Swab holder

NO TECHNICAL SPECIFICATIONS NONE

Catalogue Number	Item Name	Available on Transversal Contract	Contract Number or Code
PHC-I-011	Swab holder	RT 252	RT 252-01-139 ME

Functional Specifications	Forceps, sponge/gauze pad holding.<br>Rampley.<br>Straight, box joint.<br>Serrated jaws with ratchet.<br>Stainless steel.<br>Length: 180mm.<br>Autoclaveable.<br>Specification number: 1542.
Use	Forceps with two looped jaws, designed to hold surgical sponges securely.<br>Ratcheting lock.<br>Finger-rings both shanks.
Applicable Standards	ISO 13402:1995, ISO7151:1988, ISO 7153-1:2016, ISO900101 & ISO13485.
Performance Specifications	15-year service life.
Warranties	2 years.
Note to Procurement	

NO TECHNICAL SPECIFICATIONS: NONE

Syringe: dental

Catalogue Number	Item Name	Available on Transversal Contract	Contract Number or Code
PHC-I-031	Syringe: dental	RT287-2015	RT287-04-066

Functional Specifications	Syringe, cartridge: Aspirating, 1.8 ml Carpule, SL 33 needle attachment with short cartridge pick, No SL 18 C, double claw plunge.<br>For infiltration of local anaesthetic in surgery and oral health.
Use	
Applicable Standards	ISO 9997:1999 (SANS standard is for a disposable type).
Performance Specifications	Autoclaveable.<br>Expected service life 15 years.
Warranties	2 years.
Note to Procurement	Aspirating model has been selected instead of T-bar type, as this is required for Oral Health.

Obstetric

Basin

NO TECHNICAL SPECIFICATIONS NONE

Catalogue Number	Item Name	Available on Transversal Contract	Contract Number or Code
PHC-I-021	Basin		

Functional Specifications	Basin.<br>Dimensions approximately 450daimx100mm high.<br>Stainless steel.<br>Autoclaveable.
Use	Used to hold obstetric instruments during sterilisation.<br>Once the pack is opened, filled with warm disinfectant solution for instrument and glove rinsing.
Applicable Standards	ISO 13402:1995, ISO 7153-1:2016, IS0900101 & IS013485.
Performance Specifications	15-year service life.
Warranties	2 years.
Note to Procurement	

Bowl: large

NO TECHNICAL SPECIFICATIONS
NONE

Catalogue Number	Item Name	Available on Transversal Contract	Contract Number or Code
PHC-I-022	Bowl: large	No	

Functional Specifications	Bowl, large.<br>Dimensions approximately 350daim x 60mm high.<br>Stainless steel.<br>Autoclaveable.
Use	Large Stainless steel bowl, used to hold instruments and/or disinfectant lotions during a procedure.
Applicable Standards	ISO 13402:1995, ISO 7153-1:2016, ISO900101 & ISO13485.
Performance Specifications	15-year service life.
Warranties	2 years.
Note to Procurement	

Bowl: small

NO TECHNICAL SPECIFICATIONS NONE

Catalogue Number	Item Name	Available on Transversal Contract	Contract Number or Code
PHC-I-012	Bowl: small	No	

Functional Specifications	Gallipot/lotion bowl.<br>Diameter approximately 200mm.<br>Stainless steel.<br>Autoclaveable.
Use	Uses include holding Skin disinfectant during preparation for a procedure, and as holder for sutures during the procedure.
Applicable standards	ISO 13402:1995, ISO 7153-1:2016, ISO900101 & ISO13485.
Performance Specifications	15-year service life.
Warranties	2 years.
Note to procurement	

NO TECHNICAL SPECIFICATIONS NONE

Kidney dish

Catalogue Number	Item Name	Available on Transversal Contract	Contract Number or Code
PHC-I-013	Kidney dish: large	No	
PHC-I-032	Kidney dish: small	No	

Functional Specifications	Kidney dish.<br>Dimensions approximately:<br>Large: approx 250x130x40mm<br>Small: approx 150x90x32mm.<br>Stainless steel.<br>Autoclaveable.
Use	For general use as a receptacle for fluids, tissues and instruments.
Applicable standards	ISO 13402:1995, ISO 7153-1:2016, IS0900101 & IS013485.
Performance Specifications	15-year service life.
Warranties	2 years.
Note to procurement	

Scissors: episiotomy

NO TECHNICAL SPECIFICATIONS
NONE

Catalogue Number	Item Name	Available on Transversal Contract	Contract Number or Code
PHC-I-019	Scissors: episiotomy	RT 252	RT 252-01-234 ME

Functional Specifications	Scissors, episiotomy, Braun-Stadler.<br>Stainless steel.<br>Angled to side.<br>Blunt/blunt blades.<br>Length: 150mm.<br>Autoclaveable.<br>Specification number: 5411.
Use	Medium-size scissors.<br>Strong blades, both blunt to avoid inadvertent damage to birth canal structures during an episiotomy.<br>Blades are angles to one side, to permit insertion into maximally-dilated cervix.
Applicable Standards	ISO 13402:1995, ISO7741:1986, ISO 7153-1:2016, ISO900101 & ISO13485.
Performance Specifications	10-year service life.
Warranties	2 years.
Note to Procurement	

Scissors: umbilical

NO TECHNICAL SPECIFICATIONS
NONE

Catalogue Number	Item Name	Available on Transversal Contract	Contract Number or Code
PHC-I-018	Scissors: umbilical	RT 252	RT 252-01-242 ME

Functional Specifications	Scissors, umbilical.<br>Screw joint.<br>Stainless steel.<br>Autoclavable.<br>Blunt/blunt blades.<br>Length: 120mm.<br>Specification number: 5412.
Use	Scissors with wide, flat, curved blades.<br>Used to cut the umbilical cord.<br>The inner curve of the blades holds the cord, so that it does not slip while being cut.
Applicable standards	ISO 13402:1995, ISO7741:1986, ISO 7153-1:2016, ISO900101 & ISO13485.
Performance Specifications	10-year service life.
Warranties	2 years.
Note to Procurement	

Oral health

Note: to avoid duplication, the following items are listed elsewhere in this Instruments section:

- Cotton wool holder (see Bowl: Small)
- Dental syringe and needles
- Kidney dish

Amalgam carrier: plastic

NO TECHNICAL SPECIFICATIONS
NONE

Catalogue Number	Item Name	Available on Transversal Contract	Contract Number or Code
PHC-I-033	Amalgam carrier: plastic, straight	RT287-2015	RT287-04-003
PHC-I-034	Amalgam carrier: plastic, right-angle	RT287-2015	RT287-04-002

Functional Specifications	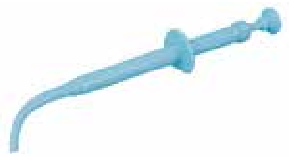
Applicable Standards	
Performance Specifications	Autoclaveable.<br>Expected service life 10 years.
Accessories	None.

Warranties	1 year (excluding deliberate or accidental damage).
Instructions to Suppliers	
Note to Procurement	

Amalgam plugger

NO TECHNICAL SPECIFICATIONS
NONE

Catalogue Number	Item Name	Available on Transversal Contract	Contract Number or Code
PHC-I-035	Amalgam plugger: No.151	RT287-2015	RT287-04-012
PHC-I-036	Amalgam plugger: No.153	RT287-2015	RT287-04-013

Functional Specifications	RT287-04-012: Plugger, amalgam, dental: Plastic Inst. No 151, serrated handle.<br>RT287-04-013: Plugger, amalgam, dental: Plastic Inst. No 153, serrated handle.
Applicable Standards	
Performance Specifications	Autoclaveable.<br>Expected service life 10 years.
Accessories	None.
Warranties	1 year (excluding deliberate or accidental damage).
Instructions to Suppliers	
Note to Procurement	

Ball burnisher

NO TECHNICAL SPECIFICATIONS
NONE

Catalogue Number	Item Name	Available on Transversal Contract	Contract Number or Code
PHC-I-037	Ball burnisher No. 153	RT287-2015	RT287-04-019
PHC-I-038	Ball burnisher No. 1/23	RT287-2015	RT287-04-020

Functional Specifications	Plugger, plastic filling, dental: Composite. Instrument, Zirconium Nitride Coated, No. Plastic 153 SM, silicone handle. Plugger, plastic filling, dental: Composite instrument, Zirconium Nitride Coated, No. Hollenbach 1/2 3, silicone handle. 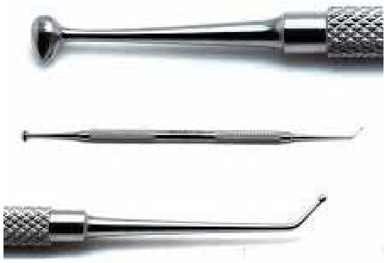
Applicable Standards	
Performance Specifications	Autoclaveable. Expected service life 10 years.
Accessories	None.
Warranties	1 year (excluding deliberate or accidental damage).
Instructions to Suppliers	
Note to Procurement	

Bur Block

NO TECHNICAL SPECIFICATIONS NONE

Catalogue Number	Item Name	Available on Transversal Contract	Contract Number or Code
PHC-I-039	Bur Block	No	

Functional Specifications	Plastic or resin block, with holes to hold approximately 12 Small, Medium and large burs.
Applicable Standards	
Performance Specifications	Autoclaveable.<br>Expected service life 10 years.
Accessories	None.
Warranties	1 year (excluding deliberate or accidental damage).
Instructions to Suppliers	
Note to Procurement	

Bur brush

NO TECHNICAL SPECIFICATIONS
NONE

Catalogue Number	Item Name	Available on Transversal Contract	Contract Number or Code
PHC-I-111	Bur brush	RT287-2015	RT287-07-001

Functional Specifications	Brush, Scratch, Dental: Bur Cleaning, Brass Wire Brush Pencil Type No. 3, Telescopic Handle .
Applicable Standards	
Performance Specifications	Autoclaveable.<br>Expected service life 10 years.
Accessories	None.
Warranties	1 year (excluding deliberate or accidental damage).
Instructions to Suppliers	
Note to Procurement	

Carver: Amalgam

NO TECHNICAL SPECIFICATIONS NONE

Catalogue Number	Item Name	Available on Transversal Contract	Contract Number or Code
PHC-I-040	Amalgam carver	RT287-2015	RT287-04-008

Functional Specifications	Carver, dental: Amalgam, Cleoid-Discoid, 92/89, serrated handle.	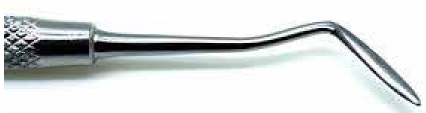
Applicable Standards		
Performance Specifications	Autoclaveable.<br>Expected service life 10 years.	
Accessories	None.	
Warranties	1 year (excluding deliberate or accidental damage).	
Instructions to Suppliers		
Note to Procurement		

Carver: wax

NO TECHNICAL SPECIFICATIONS
NONE

Catalogue Number	Item Name	Available on Transversal Contract	Contract Number or Code
PHC-I-041	Carver: wax	RT287-2015	RT287-09-030

Functional Specifications	Carver, Dental: Wax, Le Cron. 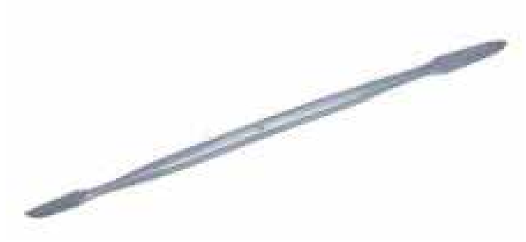
Applicable Standards	
Performance Specifications	Autoclaveable.<br>Expected service life 10 years.
Accessories	None.
Warranties	1 year (excluding deliberate or accidental damage).
Instructions to Suppliers	
Note to Procurement	

Dappen dish

NO TECHNICAL SPECIFICATIONS NONE

Catalogue Number	Item Name	Available on Transversal Contract	Contract Number or Code
PHC-I-042	Dappen dish	RT287-2015	RT287-07-009

Functional Specifications	Dish, Medicament, Dental: Dappen Type, 30 x 25mm, Clear, Autoclavable.
Applicable Standards	
Performance Specifications	Autoclaveable.
Accessories	None.
Warranties	1 year (excluding deliberate or accidental damage).
Instructions to Suppliers	
Note to Procurement	

Dental Explorer

Catalogue Number	Item Name	Available on Transversal Contract	Contract Number or Code
PHC-I-043	Dental Explorer: FT2/54	RT287-2015	RT287-01-001
PHC-I-044	Dental Explorer: FT 5/8	RT287-2015	RT287-01-002

Functional Specifications	RT287-01-001: Explorer, dental: No. FT2/54, Blacks Formula: 7-4-23 / 10-6-26, Serrated handle.<br>RT287-01-002: Explorer, dental: No. FT 5/8, Blacks Formula: 14-4 / 11-24, Serrated handle.	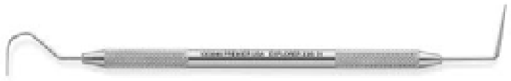
Applicable Standards		
Performance Specifications	Autoclaveable.<br>Expected service life 15 years.	
Accessories	None.	
Warranties	1 year (excluding deliberate or accidental damage).	
Instructions to Suppliers		
Note to Procurement		

Dental Probe: periodontal

NO TECHNICAL SPECIFICATIONS NONE

Catalogue Number	Item Name	Available on Transversal Contract	Contract Number or Code
PHC-I-045	Dental Probe: periodontal	RT287-2015	RT287-04-034

Functional Specifications	Probe, periodontal: Expro, Color-coded, 1-2-3-5-7-8-9-10mm interval markings, Serrated handle.	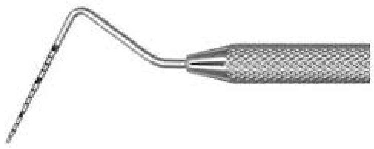
Applicable Standards		
Performance Specifications	Autoclaveable.<br>Expected service life 15 years.	
Accessories	None.	
Warranties	1 year (excluding deliberate or accidental damage).	
Instructions to Suppliers		
Note to Procurement		

Elevator: root, apical

NO TECHNICAL SPECIFICATIONS
NONE

Catalogue Number	Item Name	Available on Transversal Contract	Contract Number or Code
PHC-I-046	Elevator: root, apical, straight	RT287-2015	RT287-06-008
PHC-I-047	Elevator: root, apical, right	RT287-2015	RT287-06-007
PHC-I-048	Elevator: root, apical, left	RT287-2015	RT287-06-006

Functional Specifications	RT287-06-006: Elevator, Root: Apical Left, No. 27.<br>RT287-06-007: Elevator, Root: Apical Right, No. 28.<br>RT287-06-008: Elevator, Root: Apical Straight, No. 26.
Applicable Standards	
Performance Specifications	Autoclaveable.<br>Expected service life 15 years.
Accessories	None.
Warranties	1 year (excluding deliberate or accidental damage).
Instructions to Suppliers	
Note to Procurement	

Elevator: root, Cryers

NO TECHNICAL SPECIFICATIONS NONE

Catalogue Number	Item Name	Available on Transversal Contract	Contract Number or Code
PHC-I-049	Elevator: Cryers, right	RT287-2015	RT287-06-010
PHC-I-050	Elevator: Cryers, left	RT287-2015	RT287-06-009

Functional Specifications	Root elevator, Cryers pattern, available in Left and Right.	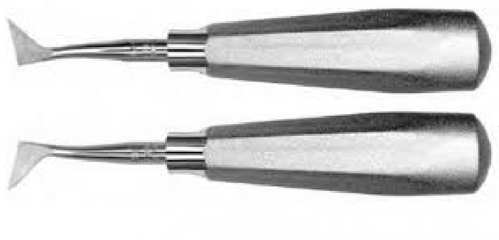
Applicable Standards		
Performance Specifications	Autoclaveable.<br>Expected service life 15 years.	
Accessories	None.	
Warranties	1 year (excluding deliberate or accidental damage).	
Instructions to Suppliers		
Note to Procurement		

Elevator: root, Warwick-James

NO TECHNICAL SPECIFICATIONS
NONE

Catalogue Number	Item Name	Available on Transversal Contract	Contract Number or Code
PHC-I-051	Elevator: root, Warwick-James, straight	RT287-2015	RT287-06-014
PHC-I-052	Elevator: root, Warwick-James, left	RT287-2015	RT287-06-012
PHC-I-113	Elevator: root, Warwick-James, right	RT287-2015	RT287-06-013

Functional Specifications	Root elevator, Warwick-James pattern, available in Straight, Left and Right. 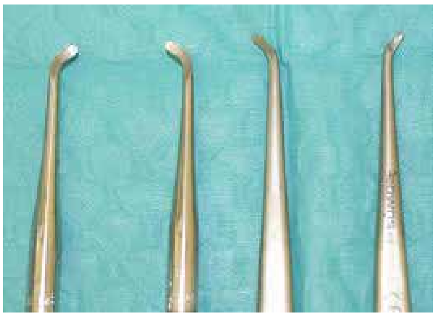
Applicable Standards	
Performance Specifications	Autoclaveable.<br>Expected service life 15 years.
Accessories	None.
Warranties	1 year (excluding deliberate or accidental damage).
Instructions to Suppliers	
Note to Procurement	

Excavator: dental

NO TECHNICAL SPECIFICATIONS NONE

Catalogue Number	Item Name	Available on Transversal Contract	Contract Number or Code
PHC-I-053	Excavator: 125/126	RT287-2015	RT287-04-022
PHC-I-054	Excavator: 129/130	RT287-2015	RT287-04-023
PHC-I-055	Excavator: 133/134	RT287-2015	RT287-04-024

Functional Specifications	RT287-04-022: No. 125/126, Blacks Formula: 25-90-9-8R / 25-10-9-8-L, serrated handle.<br>RT287-04-023: No. 129/130, Blacks Formula: 17-90-9-8-R / 17-10-9-8-L, serrated handle.<br>RT287-04-024: No. 133/134, Blacks Formula: 10-90-9-8-R / 10-10-9-8-L, serrated handle.	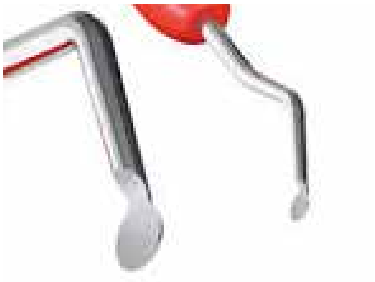
Applicable Standards		
Performance Specifications	Autoclaveable.<br>Expected service life 15 years.	
Accessories	None.	
Warranties	1 year (excluding deliberate or accidental damage).	
Instructions to Suppliers		
Note to Procurement		

Flat plastic

NO TECHNICAL SPECIFICATIONS NONE

Catalogue Number	Item Name	Available on Transversal Contract	Contract Number or Code
PHC-I-056	Flat plastic: No. 3	RT287-2015	RT287-04-037
PHC-I-057	Flat plastic: No. 2	RT287-2015	RT287-04-038

Functional Specifications	RT287-04-037: Plugger, plastic filling, dental: Thymozine Inst. No. 3, serrated handle.<br>RT287-04-038: Plugger, plastic filling, dental: Thymozine Inst. No. 2, serrated handle.	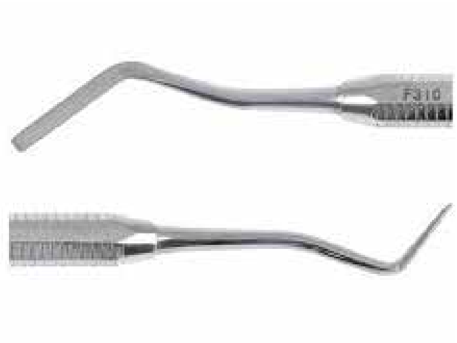
Applicable Standards		
Performance Specifications	Autoclaveable.<br>Expected service life 15 years.	
Accessories	None.	
Warranties	1 year (excluding deliberate or accidental damage).	
Instructions to Suppliers		
Note to Procurement		

Forceps: tongue

NO TECHNICAL SPECIFICATIONS
NONE

Catalogue Number	Item Name	Available on Transversal Contract	Contract Number or Code
PHC-I-058	Forceps: tongue	RT267-2015	RT267-06-043

Functional Specifications	Retractor, Oral: Tongue and Cheek Retractor<br>Copper; 150mm.	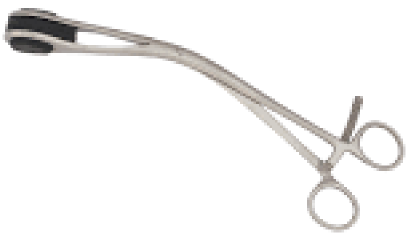
Applicable Standards		
Performance Specifications	Autoclaveable.<br>Expected service life 15 years.	
Accessories	None.	
Warranties	1 year (excluding deliberate or accidental damage).	
Instructions to Suppliers		
Note to Procurement		

Forceps: tooth extracting, Lower Anteriors Adult

NO TECHNICAL SPECIFICATIONS
NONE

Catalogue Number	Item Name	Available on Transversal Contract	Contract Number or Code
PHC-I-115	Forceps: tooth extracting Lower Canines Adult	No	

Functional Specifications	Forceps for extracting Adult Lower Anteriors.	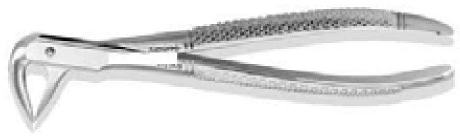
Applicable Standards		
Performance Specifications	Standard cleaning and disinfection.	
Accessories	None.	
Warranties	1 year (excluding deliberate or accidental damage).	
Instructions to Suppliers		
Note to Procurement		

Forceps: tooth extracting, Lower Bicuspids

NO TECHNICAL SPECIFICATIONS
NONE

Catalogue Number	Item Name	Available on Transversal Contract	Contract Number or Code
PHC-I-059	Forceps: tooth extracting, Lower Bicuspids	RT287-2015	RT287-06-019

Functional Specifications	Forceps: tooth extracting, Lower Bicuspids, No 13.	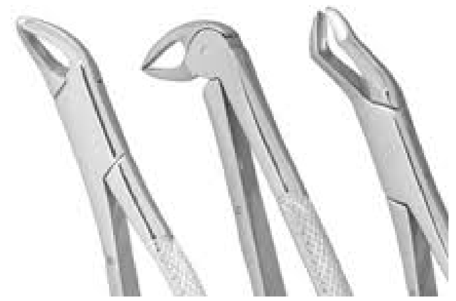
Applicable Standards		
Performance Specifications	Autoclaveable.<br>Expected service life 15 years.	
Accessories	None.	
Warranties	1 year (excluding deliberate or accidental damage).	
Instructions to Suppliers		
Note to Procurement		

Forceps: tooth extracting, Lower Canines Adult

NO TECHNICAL SPECIFICATIONS
NONE

Catalogue Number	Item Name	Available on Transversal Contract	Contract Number or Code
PHC-I-114	Forceps: tooth extracting Lower Canines Adult	No	

Functional Specifications	Forceps for extracting Adult Lower Canines	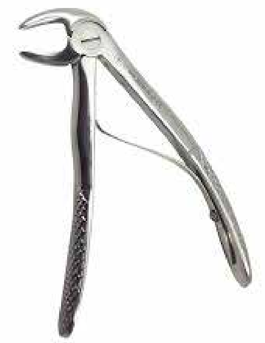
Applicable Standards		
Performance Specifications	Standard cleaning and disinfection.	
Accessories	None.	
Warranties	1 year (excluding deliberate or accidental damage)	
Instructions to Suppliers		
Note to Procurement		

Forceps: tooth extracting, Lower Molars

NO TECHNICAL SPECIFICATIONS
NONE

Catalogue Number	Item Name	Available on Transversal Contract	Contract Number or Code
PHC-I-060	Forceps: tooth extracting, Lower Molars	RT287-2015	RT287-06-021

Functional Specifications	Forceps: tooth extracting, Lower Molars, No 86 Narrow beak (Cow-horn).	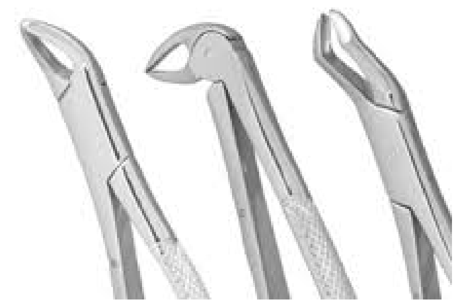
Applicable Standards		
Performance Specifications	Autoclaveable.<br>Expected service life 15 years.	
Accessories	None.	
Warranties	1 year (excluding deliberate or accidental damage).	
Instructions to Suppliers		
Note to Procurement		

NO TECHNICAL SPECIFICATIONS
NONE

Forceps: tooth extracting, Lower roots and Crowded Incisors

Catalogue Number	Item Name	Available on Transversal Contract	Contract Number or Code
PHC-I-061	Forceps: tooth extracting, Lower roots and Crowded Incisors	RT287-2015	RT287-06-022

Functional Specifications	Forceps: tooth extracting, Lower roots and Crowded Incisors.<br>No. 74N (cow-horn: narrow beak).	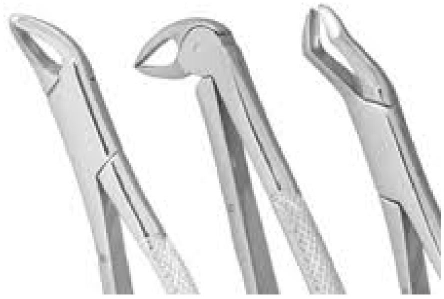
Applicable Standards		
Performance Specifications	Autoclaveable.<br>Expected service life 15 years.	
Accessories	None.	
Warranties	1 year (excluding deliberate or accidental damage).	
Instructions to Suppliers		
Note to Procurement		

Forceps: tooth extracting, Upper Anteriors and Canines

NO TECHNICAL SPECIFICATIONS
NONE

Catalogue Number	Item Name	Available on Transversal Contract	Contract Number or Code
PHC-I-062	Forceps: tooth extracting, Upper Anteriors and Canines	RT287-2015	RT287-06-024

Functional Specifications	Forceps: tooth extracting, Upper Anteriors and Canines.<br>No. 107.
Applicable Standards	
Performance Specifications	Autoclaveable.<br>Expected service life 15 years.
Accessories	None.
Warranties	1 year (excluding deliberate or accidental damage).
Instructions to Suppliers	
Note to Procurement	

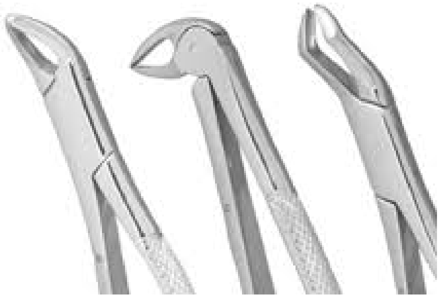

Forceps: tooth extracting, Upper Bicuspids and Roots

NO TECHNICAL SPECIFICATIONS NONE

Catalogue Number	Item Name	Available on Transversal Contract	Contract Number or Code
PHC-I-063	Forceps: tooth extracting, Upper Bicuspids and Roots	RT287-2015	RT287-06-026

Functional Specifications	Forceps: tooth extracting, Upper Bicuspids and Roots, No. 7.	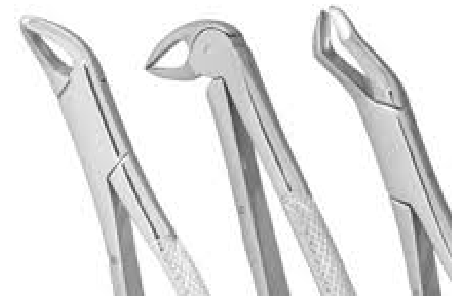
Applicable Standards		
Performance Specifications	Autoclaveable.<br>Expected service life 15 years.	
Accessories	None.	
Warranties	1 year (excluding deliberate or accidental damage).	
Instructions to Suppliers		
Note to Procurement		

Forceps: tooth extracting, Upper Molars, Left

NO TECHNICAL SPECIFICATIONS
NONE

Catalogue Number	Item Name	Available on Transversal Contract	Contract Number or Code
PHC-I-064	Forceps: tooth extracting, Upper Molars, Left	RT287-2015	RT287-06-027

Functional Specifications	Forceps: tooth extracting, Upper Molars, Left, No. 90N (Cow-horn). 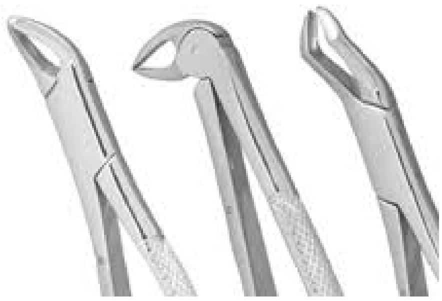
Applicable Standards	
Performance Specifications	Autoclaveable.<br>Expected service life 15 years.
Accessories	None.
Warranties	1 year (excluding deliberate or accidental damage).
Instructions to Suppliers	
Note to Procurement	

Forceps: tooth extracting, Upper Molars, Right

NO TECHNICAL SPECIFICATIONS
NONE

Catalogue Number	Item Name	Available on Transversal Contract	Contract Number or Code
PHC-I-065	Forceps: tooth extracting, Upper Molars, Right	RT287-2015	RT287-06-027

Functional Specifications	Forceps: tooth extracting, Upper Molars, Right. No. 17.	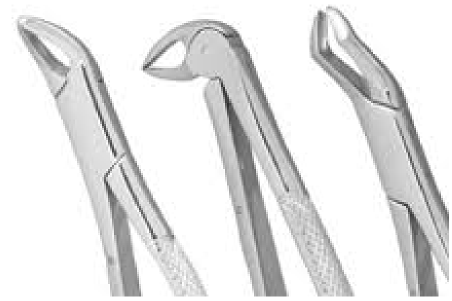
Applicable Standards		
Performance Specifications	Autoclaveable.<br>Expected service life 15 years.	
Accessories	None.	
Warranties	1 year (excluding deliberate or accidental damage).	
Instructions to Suppliers		
Note to Procurement		

Forceps: tooth extracting, Upper Molars, Right

NO TECHNICAL SPECIFICATIONS NONE

Catalogue Number	Item Name	Available on Transversal Contract	Contract Number or Code
PHC-I-066	Forceps: tooth extracting, Upper Molars, Right	RT287-2015	RT287-06-029

Functional Specifications	Forceps: tooth extracting, Upper Molars, Right.<br>No. 89 (cow-horn).	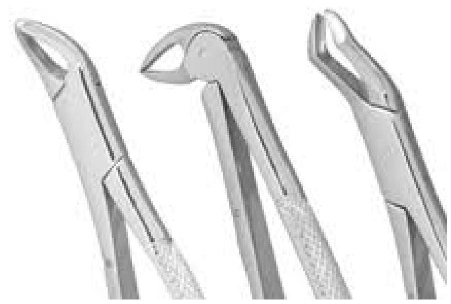
Applicable Standards		
Performance Specifications	Autoclaveable.<br>Expected service life 15 years.	
Accessories	None.	
Warranties	1 year (excluding deliberate or accidental damage).	
Instructions to Suppliers		
Note to Procurement		

Forceps: tooth extracting, Upper roots

NO TECHNICAL SPECIFICATIONS NONE

Catalogue Number	Item Name	Available on Transversal Contract	Contract Number or Code
PHC-I-067	Forceps: tooth extracting, Upper roots	RT287-2015	RT287-06-030

Functional Specifications	Forceps: tooth extracting, Upper roots, No. 29. 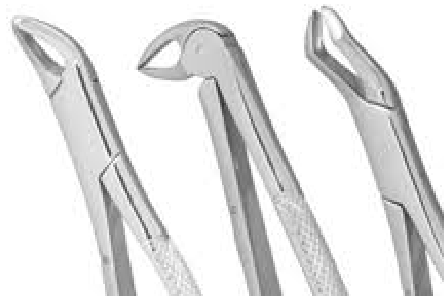
Applicable Standards	
Performance Specifications	Autoclaveable.<br>Expected service life 15 years.
Accessories	None.
Warranties	1 year (excluding deliberate or accidental damage).
Instructions to Suppliers	
Note to Procurement	

Forceps: tooth extracting, Upper roots

NO TECHNICAL SPECIFICATIONS NONE

Catalogue Number	Item Name	Available on Transversal Contract	Contract Number or Code
PHC-I-068	Forceps: tooth extracting, Upper roots	RT287-2015	RT287-06-031

Functional Specifications	Forceps: tooth extracting, Upper roots, No 29S.	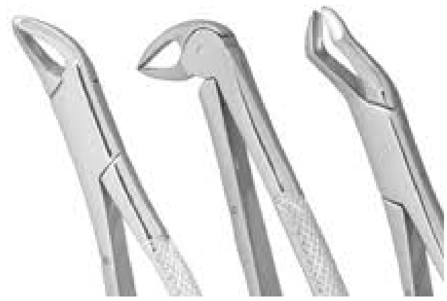
Applicable Standards		
Performance Specifications	Autoclaveable.<br>Expected service life 15 years.	
Accessories	None.	
Warranties	1 year (excluding deliberate or accidental damage).	
Instructions to Suppliers		
Note to Procurement		

Forceps: tooth extracting, Upper roots

NO TECHNICAL SPECIFICATIONS NONE

Catalogue Number	Item Name	Available on Transversal Contract	Contract Number or Code
PHC-I-069	Forceps: tooth extracting, Upper roots	RT287-2015	RT287-06-032

Functional Specifications	Forceps: tooth extracting, Upper roots, No 44 N.	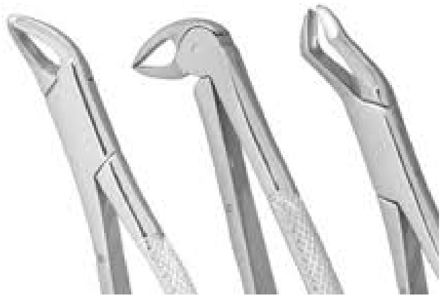
Applicable Standards		
Performance Specifications	Autoclaveable.<br>Expected service life 15 years.	
Accessories	None.	
Warranties	1 year (excluding deliberate or accidental damage).	
Instructions to Suppliers		
Note to Procurement		

Forceps: tooth extracting, Upper roots

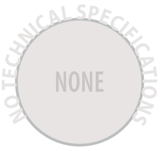

Catalogue Number	Item Name	Available on Transversal Contract	Contract Number or Code
PHC-I-070	Forceps: tooth extracting, Upper roots	RT287-2015	RT287-06-033

Functional Specifications	Forceps: tooth extracting, Upper roots, No. 76 (Read).	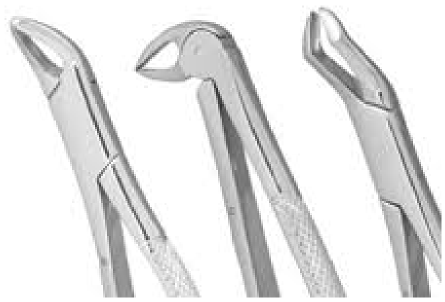
Applicable Standards		
Performance Specifications	Autoclaveable.<br>Expected service life 15 years.	
Accessories	None.	
Warranties	1 year (excluding deliberate or accidental damage).	
Instructions to Suppliers		
Note to Procurement		

Forceps: tooth extracting, Upper roots, Small

NO TECHNICAL SPECIFICATIONS NONE

Catalogue Number	Item Name	Available on Transversal Contract	Contract Number or Code
PHC-I-071	Forceps: tooth extracting, Upper roots, Small	RT287-2015	RT287-06-034

Functional Specifications	Forceps: tooth extracting, Upper roots, Small, No. 76 (Read). 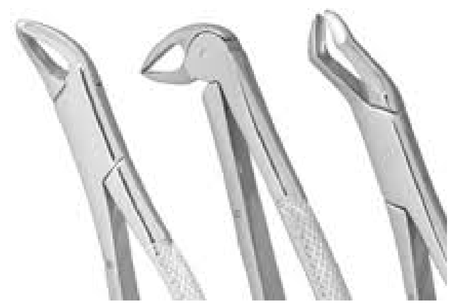
Applicable Standards	
Performance Specifications	Autoclaveable.<br>Expected service life 15 years.
Accessories	None.
Warranties	1 year (excluding deliberate or accidental damage).
Instructions to Suppliers	
Note to Procurement	

Forceps: tooth extracting, Lower molars, child

NO TECHNICAL SPECIFICATIONS
NONE

Catalogue Number	Item Name	Available on Transversal Contract	Contract Number or Code
PHC-I-072	Forceps: tooth extracting, Lower molars, child	RT287-2015	RT287-06-035

Functional Specifications	Forceps: tooth extracting, Lower molars, children pattern No. 161.	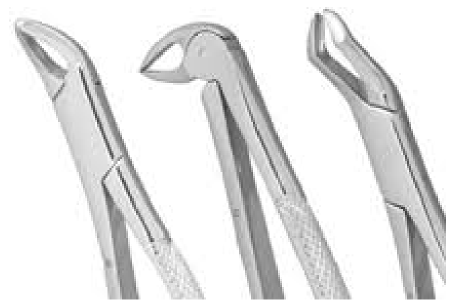
Applicable Standards		
Performance Specifications	Autoclaveable.<br>Expected service life 15 years.	
Accessories	None.	
Warranties	1 year (excluding deliberate or accidental damage).	
Instructions to Suppliers		
Note to Procurement		

Forceps: tooth extracting, Lower teeth and roots, child

NO TECHNICAL SPECIFICATIONS
NONE

Catalogue Number	Item Name	Available on Transversal Contract	Contract Number or Code
PHC-I-073	Forceps: tooth extracting, Lower teeth and roots, child	RT287-2015	RT287-06-036

Functional Specifications	Forceps: tooth extracting, Lower teeth and roots, children pattern No. 123.	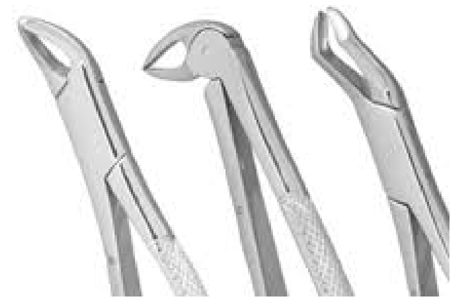
Applicable Standards		
Performance Specifications	Autoclaveable.<br>Expected service life 15 years.	
Accessories	None.	
Warranties	1 year (excluding deliberate or accidental damage).	
Instructions to Suppliers		
Note to Procurement		

Forceps: tooth extracting, Upper Incisors and Canines, child

NO TECHNICAL SPECIFICATIONS
NONE

Catalogue Number	Item Name	Available on Transversal Contract	Contract Number or Code
PHC-I-074	Forceps: tooth extracting, Upper Incisors and Canines, child	RT287-2015	RT287-06-037

Functional Specifications	Forceps: tooth extracting, Upper Incisors and Canines, children pattern No. 37.	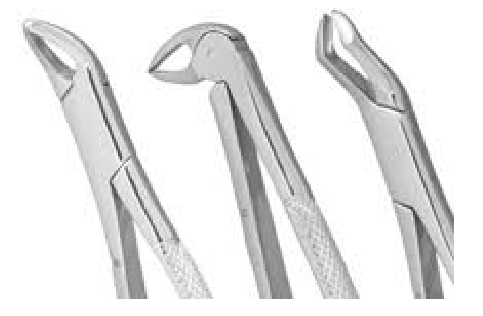
Applicable Standards		
Performance Specifications	Autoclaveable.<br>Expected service life 15 years.	
Accessories	None.	
Warranties	1 year (excluding deliberate or accidental damage).	
Instructions to Suppliers		
Note to Procurement		

Forceps: tooth extracting, Upper Molars, child

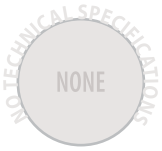

Catalogue Number	Item Name	Available on Transversal Contract	Contract Number or Code
PHC-I-075	Forceps: tooth extracting, Upper Molars, child	RT287-2015	RT287-06-038

Functional Specifications	Forceps: tooth extracting, Upper Molars, children pattern, No 39.	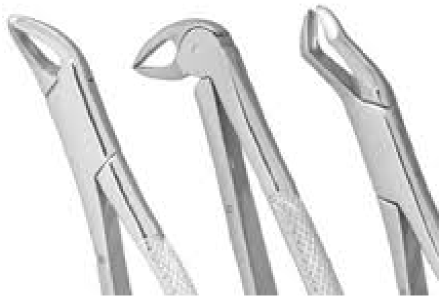
Applicable Standards		
Performance Specifications	Autoclaveable.<br>Expected service life 15 years.	
Accessories	None.	
Warranties	1 year (excluding deliberate or accidental damage).	
Instructions to Suppliers		
Note to Procurement		

Forceps: tooth extracting, Upper teeth and roots, Molars, child

NO TECHNICAL SPECIFICATIONS
NONE

Catalogue Number	Item Name	Available on Transversal Contract	Contract Number or Code
PHC-I-076	Forceps: tooth extracting, Upper teeth and roots, Molars, child	RT287-2015	RT287-06-039

Functional Specifications	Forceps: tooth extracting, Upper teeth and roots, Molars, children pattern, No. 138.	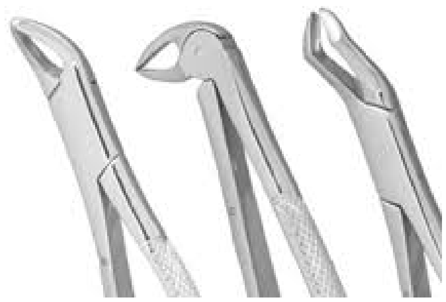
Applicable Standards		
Performance Specifications	Autoclaveable.<br>Expected service life 15 years.	
Accessories	None.	
Warranties	1 year (excluding deliberate or accidental damage).	
Instructions to Suppliers		
Note to Procurement		

Forceps: wire ligature

NO TECHNICAL SPECIFICATIONS NONE

Catalogue Number	Item Name	Available on Transversal Contract	Contract Number or Code
PHC-I-077	Forceps: wire ligature	RT287-2015	RT287-06-016

Functional Specifications	Pliers, Dental: Wire Ligature Twister, Surgical, 150mm, Tungsten Carbide Tips with Pyramidal Profile.	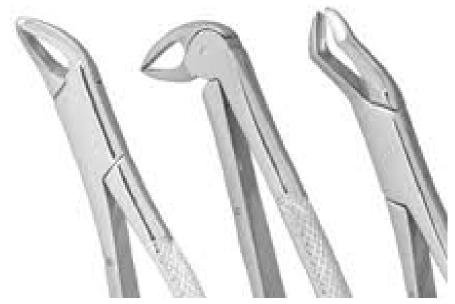
Applicable Standards		
Performance Specifications	Autoclaveable.<br>Expected service life 15 years.	
Accessories	None.	
Warranties	1 year (excluding deliberate or accidental damage).	
Instructions to Suppliers		
Note to Procurement		

Gag (mouth prop)

NO TECHNICAL SPECIFICATIONS NONE

Catalogue Number	Item Name	Available on Transversal Contract	Contract Number or Code
PHC-I-078	Gag (mouth prop): large	RT287-2015	RT287-06-050
PHC-I-079	Gag (mouth prop): medium	RT287-2015	RT287-06-051
PHC-I-080	Gag (mouth prop): small	RT287-2015	RT287-06-052

Functional Specifications	Mouth Prop, Dental: Rubber with Stainless Steel Insert.<br>In Large, medium and Small sizes.
Applicable Standards	
Performance Specifications	Autoclaveable.<br>Expected service life 15 years.
Accessories	None.
Warranties	1 year (excluding deliberate or accidental damage).
Instructions to Suppliers	
Note to Procurement	

Holder: Bib

NO TECHNICAL SPECIFICATIONS NONE

Catalogue Number	Item Name	Available on Transversal Contract	Contract Number or Code
PHC-I-081	Bib holder	RT287-2015	RT287-07-014

Functional Specifications	Holder, Napkin, Dental: Round Plastic or Chain Necklace with two Alligators Clips.
Applicable Standards	
Performance Specifications	Standard cleaning and disinfection.<br>Expected service life 5 years.
Accessories	None.
Warranties	1 year (excluding deliberate or accidental damage).
Instructions to Suppliers	
Note to Procurement	

Holder: cotton pellet

NO TECHNICAL SPECIFICATIONS NONE

Catalogue Number	Item Name	Available on Transversal Contract	Contract Number or Code
PHC-I-082	Cotton pellet holder	RT287-2015	RT287-07-010

Functional Specifications	Dispenser, Cotton Pellet, Dental: Chrome Plated Brass with Mesh Top, Spring Loaded Placement, Flip Cover, 42,5 x 42,5 x 48mm .
Applicable Standards	
Performance Specifications	Autoclaveable.<br>Expected service life 15 years.
Accessories	None.
Warranties	1 year (excluding deliberate or accidental damage).
Instructions to Suppliers	
Note to Procurement	

Matrix retainer

NO TECHNICAL SPECIFICATIONS NONE

Catalogue Number	Item Name	Available on Transversal Contract	Contract Number or Code
PHC-I-083	Matrix retainer: narrow	RT287-2015	RT287-04-041
PHC-I-084	Matrix retainer: wide	RT287-2015	RT287-04-042

Functional Specifications	RT287-04-041: Retainer, matrix, dental: Siqveland, narrow.<br>RT287-04-042: Retainer, matrix, dental: Siqveland, wide.	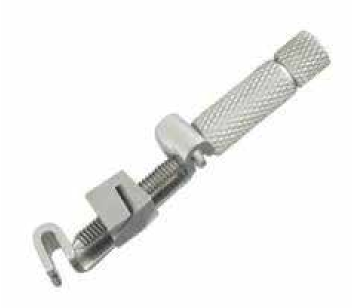
Applicable Standards		
Performance Specifications	Autoclaveable.<br>Expected service life 15 years.	
Accessories	None.	
Warranties	1 year (excluding deliberate or accidental damage).	
Instructions to Suppliers		
Note to Procurement		

Mouth Mirror

NO TECHNICAL SPECIFICATIONS NONE

Catalogue Number	Item Name	Available on Transversal Contract	Contract Number or Code
PHC-I-085	Mouth Mirror: Handle	RT287-2015	RT287-01-005
PHC-I-086	Mirror: head, to fit handle	RT287-2015	RT287-01-007

Functional Specifications	RT287-01-005: Serrated handle for mouth examining mirror. Metric thread.<br>RT287-01-007: Mirror for mouth examination. Metal with simple screw stem and metric thread, plane front-surface, size No. 4.	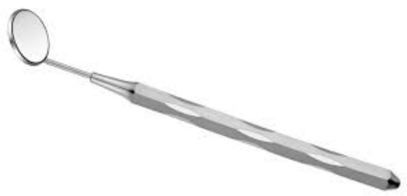
Applicable Standards		
Performance Specifications	Autoclaveable.<br>Expected service life 15 years.	
Accessories	None.	
Warranties	1 year (excluding deliberate or accidental damage).	
Instructions to Suppliers		
Note to Procurement		

Needle holder

NO TECHNICAL SPECIFICATIONS
NONE

Catalogue Number	Item Name	Available on Transversal Contract	Contract Number or Code
PHC-I-087	Needle holder	RT287-2015	RT287-06-040

Functional Specifications	Holder, Suture Needle: Dental, Crile-Wood, Tungsten Carbide Inserts with a Ground Pyramidal Profile, 150mm.
Applicable Standards	
Performance Specifications	Autoclaveable.<br>Expected service life 15 years.
Accessories	None.
Warranties	1 year (excluding deliberate or accidental damage).
Instructions to Suppliers	
Note to Procurement	

Periodontal Hoe

NO TECHNICAL SPECIFICATIONS
NONE

Catalogue Number	Item Name	Available on Transversal Contract	Contract Number or Code
PHC-I-117	Periodontal Hoe: SG 5F	RT287-2015	RT287-03-025

Functional Specifications	Hoe, Periodontal: Goldman Fox No. SG F 5, serrated handle.	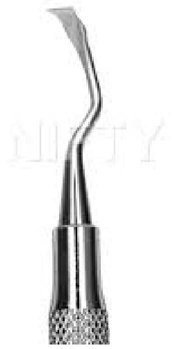
Applicable Standards		
Performance Specifications	Standard cleaning and disinfection.	
Accessories	None.	
Warranties	1 year (excluding deliberate or accidental damage).	
Instructions to Suppliers		
Note to Procurement		

Rongeur: dental

NO TECHNICAL SPECIFICATIONS
NONE

Catalogue Number	Item Name	Available on Transversal Contract	Contract Number or Code
PHC-I-088	Rongeur: dental No. 4	RT287-2015	RT287-06-017
PHC-I-089	Rongeur: dental No. 5S	RT287-2015	RT287-06-018

Functional Specifications	Rongeur: Dental, No. 4 and No5S.	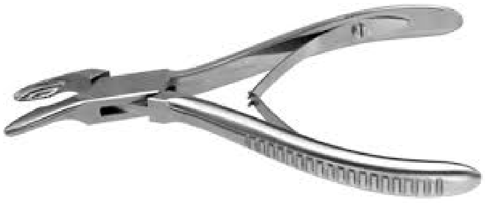
Applicable Standards		
Performance Specifications	Autoclaveable.<br>Expected service life 15 years.	
Accessories	None.	
Warranties	1 year (excluding deliberate or accidental damage).	
Instructions to Suppliers		
Note to Procurement		

Scaler, Dental

NO TECHNICAL SPECIFICATIONS NONE

Catalogue Number	Item Name	Available on Transversal Contract	Contract Number or Code
PHC-I-116	Scaler, Dental: H6/7	RT287-2015	RT287-03-029

Functional Specifications	Scaler, Dental: Hygienist No. SH 6/7, serrated handle.	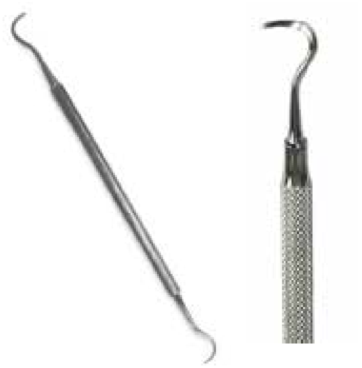
Applicable Standards		
Performance Specifications	Standard cleaning and disinfection.	
Accessories	None.	
Warranties	1 year (excluding deliberate or accidental damage).	
Instructions to Suppliers		
Note to Procurement		

Scissors: ligature

NO TECHNICAL SPECIFICATIONS
NONE

Catalogue Number	Item Name	Available on Transversal Contract	Contract Number or Code
PHC-I-093	Scissors: ligature	RT287-2015	RT287-06-047

Functional Specifications	Scissors, Medical Wire: Wire Cutting, Notched, Tungsten Inserts, 120mm.
Applicable Standards	
Performance Specifications	Autoclaveable.<br>Expected service life 15 years.
Accessories	None.
Warranties	1 year (excluding deliberate or accidental damage).
Instructions to Suppliers	
Note to Procurement	

Slab: mixing, glass

NO TECHNICAL SPECIFICATIONS
NONE

Catalogue Number	Item Name	Available on Transversal Contract	Contract Number or Code
PHC-I-090	Mixing slab, glass	RT287-2015	RT287-07-015

Functional Specifications	Mixing Slab, Dental: Glass, Large, 150 x 75 x 18-20mm.
Applicable Standards	
Performance Specifications	Autoclaveable.<br>Expected service life 15 years.
Accessories	None.
Warranties	1 year (excluding deliberate or accidental damage).
Instructions to Suppliers	
Note to Procurement	

Spatula: Cement

NO TECHNICAL SPECIFICATIONS NONE

Catalogue Number	Item Name	Available on Transversal Contract	Contract Number or Code
PHC-I-094	Spatula: Cement	RT287-2015	RT287-04-061

Functional Specifications	Spatula, dental: Cement, double ended, No. 8.
Applicable Standards	
Performance Specifications	Autoclaveable.<br>Expected service life 15 years.
Accessories	None.
Warranties	1 year (excluding deliberate or accidental damage).
Instructions to Suppliers	
Note to Procurement	

Syringe: dental, aspirating

NO TECHNICAL SPECIFICATIONS NONE

Catalogue Number	Item Name	Available on Transversal Contract	Contract Number or Code
PHC-I-095	Syringe: dental, aspirating	RT287-2015	RT287-04-066

Functional Specifications	Syringe, cartridge: Aspirating, 1.8 ml Carpule, SL 33 needle attachment with short cartridge pick, No. SL 18 C, double claw plunge.
Applicable Standards	
Performance Specifications	Autoclaveable.<br>Expected service life 15 years.
Accessories	None.
Warranties	1 year (excluding deliberate or accidental damage).
Instructions to Suppliers	
Note to Procurement	

Trimmer: Gingival margin

NO TECHNICAL SPECIFICATIONS NONE

Catalogue Number	Item Name	Available on Transversal Contract	Contract Number or Code
PHC-I-096	Trimmer: Gingival margin	RT287-2015	RT287-04-028
PHC-I-097	Trimmer: Gingival margin	RT287-2015	RT287-04-029

Functional Specifications	RT287-04-028: Trimmer, gingival margin: No. TC U1/U2, Blacks Formula: 10-80-8-12, serrated handle.<br>RT287-04-029: Trimmer, gingival margin: No. TC U3/U4, Blacks Formula: 10-95-8-12, serrated handle.	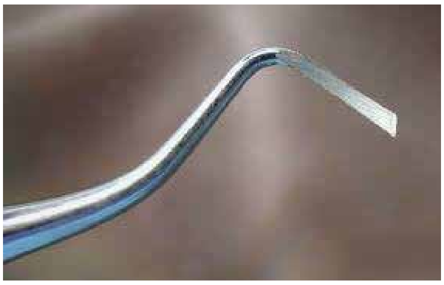
Applicable Standards		
Performance Specifications	Autoclaveable.<br>Expected service life 15 years.	
Accessories	None.	
Warranties	1 year (excluding deliberate or accidental damage).	
Instructions to Suppliers		
Note to Procurement		

Tweezers: Cotton and Dressing

NO TECHNICAL SPECIFICATIONS NONE

Catalogue Number	Item Name	Available on Transversal Contract	Contract Number or Code
PHC-I-098	Cotton and Dressing Tweezers	RT287-2015	RT287-04-030
PHC-I-099	Cotton and Dressing Tweezers	RT287-2015	RT287-04-031

Functional Specifications	RT287-04-030: Forceps, dressing: Dental, Approx. 1mm Serrated Blade, ± 150mm Length, serrated handle No DP 17.<br>RT287-04-031: Forceps, dressing: Dental Approx. 1mm Serrated Blade, ± 150mm Length, serrated handle with Lock No. DP 18L. 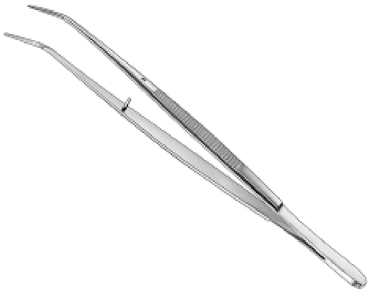
Applicable Standards	
Performance Specifications	Autoclaveable.<br>Expected service life 15 years.
Accessories	None.
Warranties	1 year (excluding deliberate or accidental damage).
Instructions to Suppliers	
Note to Procurement	

Rehabilitation

PHYSICAL THERAPY

NO TECHNICAL SPECIFICATIONS
NONE

Scissors: standard

Catalogue Number	Item Name	Available on Transversal Contract	Contract Number or Code
PHC-I-100	Scissors: standard, adult	No	
PHC-I-101	Scissors: standard, child	No	

Functional Specifications	Scissors, adult right handed. Hard plastic handles, durable.<br>Scissors, kids, left-and right- handed. Hard plastic handles, durable.
Applicable Standards	For cutting things such as strapping, material, Velcro, paper, etc.
Performance Specifications	Standard cleaning and disinfection.
Accessories	None.
Warranties	1 year (excluding deliberate or accidental damage).
Instructions to Suppliers	
Note to Procurement	

Scissors: splinting

NO TECHNICAL SPECIFICATIONS — NONE

Catalogue Number	Item Name	Available on Transversal Contract	Contract Number or Code
PHC-I-102	Scissors: splinting	No	

Functional Specifications	Heavy duty scissors. Slight curve to the blades. Must be able to cut warm thermoplastics cleanly.
Applicable Standards	Cutting and shaping thermoplastic splinting materials.
Performance Specifications	Standard cleaning and disinfection.
Accessories	None.
Warranties	1 year (excluding deliberate or accidental damage).
Instructions to Suppliers	
Note to Procurement	

AUDIOLOGY/SPEECH THERAPY

Cerumen management kit

NO TECHNICAL SPECIFICATIONS
NONE

Catalogue Number	Item Name	Available on Transversal Contract	Contract Number or Code
PHC-I-103	Ear irrigation sprayer: manual	RT274-2018ME	42182409-00032

Functional Specifications	Ear irrigation sprayer: 1 spray wash bottle (470ml) with 20 specialised ear irrigation tips and 1 irrigation basin.
Applicable Standards	
Performance Specifications	Standard cleaning and disinfection.
Accessories	Note that for complete cerumen management, the curettes, ear loop and crocodile forceps listed hereafter, are also required..
Warranties	1 year (excluding deliberate or accidental damage).
Instructions to Suppliers	
Note to Procurement	

Curette: cerumen, plastic

NO TECHNICAL SPECIFICATIONS
NONE

Catalogue Number	Item Name	Available on Transversal Contract	Contract Number or Code
PHC-I-105	Curette: cerumen, plastic, adult	RT274-2018ME	42291607-00003
PHC-I-106	Curette: cerumen, plastic, paediatric	RT274-2018ME	42291607-00004

Functional Specifications	Plastic cerumen curettes: adult. Pack of 10.<br>Plastic cerumen curettes: paediatric. Pack of 10.
Applicable Standards	
Performance Specifications	
Accessories	None.
Warranties	
Instructions to Suppliers	
Note to Procurement	

Curette: cerumen, lighted

NO TECHNICAL SPECIFICATIONS NONE

Catalogue Number	Item Name	Available on Transversal Contract	Contract Number or Code
PHC-I-107	Curette: cerumen, lighted, magnified	RT274-2018ME	RT274-2018ME

Functional Specifications	
Applicable Standards	
Performance Specifications	Standard cleaning and disinfection.
Accessories	Replacement light.<br>Replacement curettes.
Warranties	1 year (excluding deliberate or accidental damage).
Instructions to Suppliers	
Note to Procurement	

Ear loop: Bileau, small

NO TECHNICAL SPECIFICATIONS
NONE

Catalogue Number	Item Name	Available on Transversal Contract	Contract Number or Code
PHC-I-108	Ear loop: Bileau, small	RT274-2018ME	42182419-00570

Functional Specifications	
Applicable Standards	
Performance Specifications	Standard cleaning and disinfection.
Accessories	None.
Warranties	1 year (excluding deliberate or accidental damage).
Instructions to Suppliers	
Note to Procurement	

Forceps: crocodile

NO TECHNICAL SPECIFICATIONS NONE

Catalogue Number	Item Name	Available on Transversal Contract	Contract Number or Code
PHC-I-109	Forceps: crocodile	RT274-2018ME	42182419-00569

Functional Specifications	Stainless steel. Box joint. Serrated jaws. Finger rings both handles.
Applicable Standards	ISO 13402:1995, ISO 7151:1988, ISO 7153-1:2016, ISO900101 & ISO13485.
Performance Specifications	Standard cleaning and disinfection.<br>Expected service life 10 years
Accessories	None.
Warranties	5 years (excluding deliberate or accidental damage).
Instructions to Suppliers	
Note to Procurement	

Syringe: impression

NO TECHNICAL SPECIFICATIONS NONE

Catalogue Number	Item Name	Available on Transversal Contract	Contract Number or Code
PHC-I-110	Syringe: impression	RT274-2018ME	RT274-2018ME

Functional Specifications	Syringe for ear mould impression, 10-15cm in length Syringe suitable for making ear mould impressions, with double plunge impression syringe.
Applicable Standards	
Performance Specifications	Standard cleaning and disinfection.
Accessories	None.
Warranties	1 year (excluding deliberate or accidental damage).
Instructions to Suppliers	
Note to Procurement	

C

consumables
clinical

CLINICAL STATIONERY 268

- Appointment cards: general 268
- Maternity health record 269
- Patient information register 270
- Patient record for adults 271
- Patient record for children 272
- Referral forms: general 273
- Referral forms: WBPHCOT 274
- Register: assets 275
- Register: Daily reception headcount 276
- Register: Monthly tally summary 277
- Register : Patient safety incident 278
- Register: PHC comprehensive 279
- Register: Weekly tally notepad 280
- Road to Health Booklet for Boys 281
- Road to Health Booklet for Girls 282
- Sick note 283
- Temperature recording forms 284

SURGICAL 285

- AED replacement Pads 285
- Amniotomy hook 286
- Antibacterial filter: portable suction machine 287
- Antibacterial filter: trolley suction 288
- Bandage: crepe 289
- Blade: scalpel 290
- Blade: stitch-cutter 291
- Catheter: intrathoracic, silicon 292
- Catheter: suction 293
- Cotton wool: balls 1g (500s) 294
- Drainage system: chest, underwater, adult 295
- Dressing pack: disposable 296
- ECG electrodes: disposable, self-adhesive, for resting ECG 297
- Endotracheal tubes: cuffed 298
- Endotracheal tubes: uncuffed 299
- Eye patch: disposable 300
- Gauze: absorbent, Type 1, sterile, peel pack (5 swabs) 301
- Gauze: paraffin, 100X100 (box of 10) 302
- Gauze: swabs, plain 303
- Intravenous administration set 304
- Intravenous cannula 305
- Intravenous cannula: winged 306
- Lubricant gel, sterile 307
- Mask: laryngeal, adult 308
- Mask: oxygen, diluter type 309
- Mask: oxygen, re-breathing type 310
- Nasal Cannula: oxygen 311
- Nasogastric catheter 312
- Needles: hypodermic 313
- Needles: Hypodermic, Dental 314
- Padding: cast orthopaedic 315
- Plaster of Paris bandage 316
- Peak Flow Meter: mouth-piece, disposable 317
- Oropharyngeal airway (Guedel) 318
- Plaster: self-adhesive, roll 319
- Razor, disposable 320
- Sanitary towels: standard 321
- Sheath: incontinence 322
- Skin traction kit 323
- Stockinette: 100mm, roll 25m 324
- Stockinette: 150mm, roll 25m 325
- Suture materials 326
- Swab: 70% isopropyl alcohol 327
- Syringe: hypodermic 328
- Tampon: vaginal 329
- Tape: surgical. Adhesive, microporous, 25mm 330
- Tape: surgical. Adhesive, microporous, 50mm 331
- Thermal blanket 332
- Thermometer probe covers: disposable 333
- Tube: stomach washout 334
- Ultrasound gel 335
- Urinary catheters (Foley's) 336

POINT OF CARE TESTING 337

- Glucometer consumables 337
- Haemoglobinmeter consumables and accessories (older models) 338
- Haemoglobinmeter consumables (newer models) 339
- HIV test kit: rapid 340
- Lancets, Multipurpose 341
- Malaria Rapid Diagnostic test kit 342
- Rhesus test kit 344
- Urine dip sticks 345

OBSTETRIC 346

- Cord clamp: umbilical 346
- Sanitary towels: maternity 347

ORAL HEALTH 348

- Acid etch 348
- Amalgam capsule 349
- Anaesthetic: topical 350
- Apron: dental, plastic 351
- Articulating paper: thin, blue 352
- Bib: plastic 353
- Bonding agent: self-etch 354
- Brush: applicator 355
- Cement: zinc oxide and eugenol 356
- Composites 357
- Cotton wool pellets 358
- Dental floss: Waxed 359
- Dry socket alveolar paste 360
- Fissure sealants 361
- Fluoride trays 362
- Fluoride gel 363
- Glass ionomer 364
- Haemostat sponge 365
- Paste: prophylaxis 366
- Polishing kit 367
- Polishing strips 368
- Polyester strips (composite) 369
- Suture material: intra-oral 370
- Tip: dental suction (saliva ejector) 371
- Toothbrush 372
- Toothpaste: dental, fluoride 373
- Varnish: cavity liner 374

REHABILITATION 375

- Batteries: hearing aid 375
- Bicarbonate of soda: sachets 376
- Breadboard: one-handed 377
- Cable ties: nylon, 378
- Cardboard: A4, various colours 379
- Coloured pencils, pens, crayons 380
- Curette: cerumen, lighted 381
- Cushion: wheelchair 382
- Cushion covers: wheelchair 383
- Ear mould impression material and scoop 384
- Ear mould impression Otolight: replacement globe 385
- Ear mould impression Otolight: replacement tips 386
- Exercise band: latex 387
- Feeding cup 388
- Feeding spoon 389
- Ferrule: rubber 390
- Foam: EVA 391
- Foam: high-density, 36/20 392
- Foam: HD compressed 1300 393
- Masonite 394
- Oil: arnica 395
- Otostops 396
- Paper: print-out, tympanometer 397
- Pelvic straps 398
- Pressure garment material: elastonet 399
- Sewing kit 400
- Splinting material: thermoplastic sheets 401
- Splinting material: thermoplastic tape 402
- Towelling 403
- Varnish: wood 404
- Velcro: circle, self-adhesive 405
- Velcro: loop, 5cm width, 50m roll 406
- Washboard: one-handed 407
- Wheelchair spares kit 408
- Wheelchair gloves 409
- Wood 410

STERILISATION 411

- Bowie-Dick test packs 411
- Helix test kit and strips 412

PERSONAL PROTECTION EQUIPMENT 413

- Aprons: disposable 413
- Face shield 414
- Gloves: sterile 415
- Gloves: non-sterile 416
- Goggles 417
- Gown: disposable 418
- Particulate mask (N-95 respirator) 419
- Surgical mask 420

Clinical stationery

Appointment cards: general

NO TECHNICAL SPECIFICATIONS NONE

Catalogue Number	Item Name	Available on Transversal Contract	Contract Number or Code
PHC-C-016	Appointment Cards: General	No	

Description	Cards to register patient identifying data, and date and time of next appointment at the facility.
Use	Used for the implementation of the Patient Appointments system.
Applicable Standards	
Note to Procurement	

Maternity health record

NO TECHNICAL SPECIFICATIONS
NONE

Catalogue Number	Item Name	Available on Transversal Contract	Contract Number or Code
PHC-C-017	Maternity Health Record	No	

Description	
Use	Used to record the progress of a pregnancy and maternal health.
Applicable Standards	
Note to Procurement	

Patient information register

NO TECHNICAL SPECIFICATIONS
NONE

Catalogue Number	Item Name	Available on Transversal Contract	Contract Number or Code
PHC-C-022	Patient information register or tick sheet	No	

Description	
Use	
Applicable Standards	
Note to Procurement	

Patient record for adults

NO TECHNICAL SPECIFICATIONS
NONE

Catalogue Number	Item Name	Available on Transversal Contract	Contract Number or Code
PHC-C-020	Patient record for adults	No	

Description	Document to record episodes of care.
Use	Used as part of the longitudinal health record, as per ICSM.
Applicable Standards	
Note to Procurement	

Patient record for children

NO TECHNICAL SPECIFICATIONS
NONE

Catalogue Number	Item Name	Available on Transversal Contract	Contract Number or Code
PHC-C-021	Patient record for children	No	

Description	Document to record episodes of care.
Use	Used as part of the longitudinal health record, as per ICSM.
Applicable Standards	
Note to Procurement	

Referral forms: general

NO TECHNICAL SPECIFICATIONS NONE

Catalogue Number	Item Name	Available on Transversal Contract	Contract Number or Code
PHC-C-023	Referral forms: general	No	

Description	Pre-printed form for recording important information.
Use	Used when a patient is referred to the next level of care.
Applicable Standards	
Note to Procurement	

Referral forms: WBPHCOT

NO TECHNICAL SPECIFICATIONS NONE

Catalogue Number	Item Name	Available on Transversal Contract	Contract Number or Code
PHC-C-024	Referral forms: WBPHCOT	No	

Description	Pre-printed form for recording important information.
Use	Used when a patient is referred to the local WBPHCOT.
Applicable Standards	
Note to Procurement	

Register: assets

NO TECHNICAL SPECIFICATIONS NONE

Catalogue Number	Item Name	Available on Transversal Contract	Contract Number or Code
PHC-C-034	Register: Assets	No	

Description	Register of Assets of the PHC Facility.
Use	Keeping track of all non-consumable assets of the facility, including equipment, instruments, and furniture.
Applicable Standards	Format will depend on Provincial requirements.
Note to Procurement	

Register: Daily reception headcount

NO TECHNICAL SPECIFICATIONS NONE

Catalogue Number	Item Name	Available on Transversal Contract	Contract Number or Code
PHC-C-030	Register: Daily reception headcount	No	

Description	Register maintained at reception.
Use	Record of daily attendances.
Applicable Standards	
Note to Procurement	

Register: Monthly tally summary

NO TECHNICAL SPECIFICATIONS NONE

Catalogue Number	Item Name	Available on Transversal Contract	Contract Number or Code
PHC-C-031	Register: Monthly tally summary	No	

Description	Register of monthly summary statistics.
Use	Monthly summary of statistics reported to District.
Applicable Standards	
Note to Procurement	

Register : Patient safety incident

NO TECHNICAL SPECIFICATIONS
NONE

Catalogue Number	Item Name	Available on Transversal Contract	Contract Number or Code
PHC-C-033	Register : Patient Safety Incident	No	

Description	Register of Safety Incidents.
Use	For recording of Patient Safety Incidents, in accordance with the Policy.<br>Incidents are categorised as SAC1, SAC2, or SAC3. The register provides for recording details of the incident, as well as internal investigations and outcomes.<br>SAC1 incidents must be reported within 24 hours to the District or Provincial office.
Applicable Standards	Final Draft National Policy to manage Patient Safety Incidents in South Africa 18 Dec 2015.
Note to Procurement	

Register: PHC comprehensive

NO TECHNICAL SPECIFICATIONS
NONE

Catalogue Number	Item Name	Available on Transversal Contract	Contract Number or Code
PHC-C-029	Register: PHC comprehensive	No	

Description	Master record of PHC encounters.
Use	
Applicable Standards	
Note to Procurement	

Register: Weekly tally notepad

NO TECHNICAL SPECIFICATIONS
NONE

Catalogue Number	Item Name	Available on Transversal Contract	Contract Number or Code
PHC-C-032	Register: Weekly tally notepad	No	

Description	Sheets for summary of weekly statistics.
Use	Summary of weekly statistics, to be used for Monthly summary.
Applicable Standards	
Note to Procurement	

Road to Health Booklet for Boys

NO TECHNICAL SPECIFICATIONS
NONE

Catalogue Number	Item Name	Available on Transversal Contract	Contract Number or Code
PHC-C-018	Road to Health Booklet for Boys	No	

Description	Document and chart to record growth, milestones, development and immunisation.
Use	
Applicable Standards	
Note to Procurement	

Road to Health Booklet for Girls

NO TECHNICAL SPECIFICATIONS NONE

Catalogue Number	Item Name	Available on Transversal Contract	Contract Number or Code
PHC-C-019	Road to Health Booklet for Girls	No	

Description	Document and chart to record growth, milestones, development and immunisation.
Use	
Applicable Standards	
Note to Procurement	

Sick note

NO TECHNICAL SPECIFICATIONS NONE

Catalogue Number	Item Name	Available on Transversal Contract	Contract Number or Code
PHC-C-025	Sick note	No	

Description	Official sick note.
Use	Used to confirm absenteeism as result of illness or disability.
Applicable Standards	
Note to Procurement	

Temperature recording forms

NO TECHNICAL SPECIFICATIONS
NONE

Catalogue Number	Item Name	Available on Transversal Contract	Contract Number or Code
PHC-C-134	Recording form: room temperature	No	
PHC-C-135	Recording form: refrigerator temperature	No	

Description	Forms to log daily temperatures.
Use	Keep a log of temperature variations, where these may affect the stability of vaccines or medications.
Applicable Standards	
Note to Procurement	

Surgical

AED replacement Pads

NO TECHNICAL SPECIFICATIONS
NONE

Catalogue Number	Item Name	Available on Transversal Contract	Contract Number or Code
PHC-C-174	Replacement pads: Adult	RT 4	RT 4-05-075 ME
PHC-C-175	Replacement pads: Paediatric	RT 4	RT 4-05-076 ME

Description	Replacement pads for Automatic External Defibrillator.
Use	Foil-packed in sets of two. Adult and paediatric size. Discard after use.
Applicable Standards	
Note to Procurement	These items must be in series with RT 4-05-072-A-ME

Amniotomy hook

NO TECHNICAL SPECIFICATIONS
NONE

Catalogue Number	Item Name	Available on Transversal Contract	Contract Number or Code
PHC-C-171	Amniotomy hook, disposable, sterile	No	

Description	Smooth ABS plastic long-handled hook.
Use	For Artificial Rupture of Membranes, in non-progressing labour.
Applicable Standards	None available.
Note to Procurement	

Antibacterial filter: portable suction machine

NO TECHNICAL SPECIFICATIONS NONE

Catalogue Number	Item Name	Available on Transversal Contract	Contract Number or Code
PHC-C-162	Antibacterial filter: portable suction machine	No	

Description	Filter to prevent liquid being aspirated into the vacuum pump mechanism.
Use	Replace after each use.
Applicable Standards	
Note to Procurement	This item must be in series with RT 4-05-041 ME. Not currently on contract.

Antibacterial filter: trolley suction

NO TECHNICAL SPECIFICATIONS
NONE

Catalogue Number	Item Name	Available on Transversal Contract	Contract Number or Code
PHC-C-161	Antibacterial filter: trolley suction	RT 2-2016	42171803-00001

Description	Filter to prevent liquid being aspirated into the vacuum pump mechanism.
Use	Replace after each use.
Applicable Standards	
Note to Procurement	This item must be in series with 42171801-00001.

Bandage: crepe

NO TECHNICAL SPECIFICATIONS NONE

Catalogue Number	Item Name	Available on Transversal Contract	Contract Number or Code
PHC-C-147	Bandage: crepe	RT 42-2018	42311505-00031

Description	Woven cotton bandage. Roll.<br>100mm by 4,5m stretched length.
Use	Light compression over dressing.
Applicable Standards	SANS 945-1.
Note to Procurement	

Blade: scalpel

NO TECHNICAL SPECIFICATIONS
NONE

Catalogue Number	Item Name	Available on Transversal Contract	Contract Number or Code
PHC-C-144	Blade: scalpel, No 10, sterile	RT 252	RT 252-01-005 ME
PHC-C-333	Blade: scalpel, No. 11, sterile	RT252	RT252-01-006ME
PHC-C-334	Blade: scalpel, No. 12, sterile	RT252	RT252-01-007ME

Description	Full-bellied single-use scalpel blade. 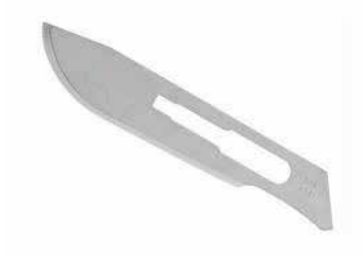
Use	Used with No 3 handle, for incisions and drainage procedures. Individually packed in sterile peel-pack. Supplied in packs/boxes.
Applicable Standards	Material: ISO 7153-1.<br>Dimensions: ISO 7740:1985.
Note to Procurement	Ensure compatibility with scalpel handle RT 252-01-015 ME.<br>For the No.12 blade, model 12D (sharpening bevel on both sides) is preferred for Oral health, therefore it should be the standard model procured.<br>Note that future requirement may be for single-use, disposable retractable-blade scalpel. Enquire before next procurement.

Blade: stitch-cutter

NO TECHNICAL SPECIFICATIONS
NONE

Catalogue Number	Item Name	Available on Transversal Contract	Contract Number or Code
PHC-C-145	Blade: stitch-cutter	RT 252	RT 252-01-028 ME

Description	Long-body stainless steel blade with curved cutting edge at one end.	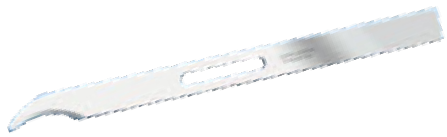
Use	For cutting stiches while maintaining aseptic technique. Does not need or fit a scalpel handle.	
Applicable Standards	Material: ISO 7153-1.	
Note to Procurement	Ensure stainless steel (some models are available in carbon steel).	

Catheter: intrathoracic, silicon

NO TECHNICAL SPECIFICATIONS
NONE

Catalogue Number	Item Name	Available on Transversal Contract	Contract Number or Code
PHC-C-364	Catheter: intrathoracic, silicon s 120	No	
PHC-C-365	Catheter: intrathoracic, silicon s 124	No	
PHC-C-366	Catheter: intrathoracic, silicon s 128	No	
PHC-C-367	Catheter: intrathoracic, silicon s 130	No	
PHC-C-368	Catheter: intrathoracic, silicon s 132	No	

Description	Sterile single-use catheters for intrathoracic insertion.
Use	Drainage of haemo-pneumothorax.
Applicable Standards	ISO 20697.
Note to Procurement	

Catheter: suction

NO TECHNICAL SPECIFICATIONS
NONE

Catalogue Number	Item Name	Available on Transversal Contract	Contract Number or Code
PHC-C-359	Catheter: suction, 6F	No	
PHC-C-360	Catheter: suction, 8F	No	
PHC-C-361	Catheter: suction, 10F	No	
PHC-C-362	Catheter: suction, 12F	No	
PHC-C-363	Catheter: suction, 14F	No	

Description	Sterile single use suction catheters.
Use	Suction of bodily fluids during resuscitation and procedures.
Applicable Standards	ISO 8836:2014.
Note to Procurement	

Cotton wool: balls 1g (500s)

NO TECHNICAL SPECIFICATIONS
NONE

Catalogue Number	Item Name	Available on Transversal Contract	Contract Number or Code
PHC-C-148	Cotton wool: balls 1g (500s)	RT 42-2018	42311505-00049

Description	Cotton wool, absorbent balls, type 2, non-sterile, autoclavable, 500g package Size: Medium 1g.
Use	Swabbing and cleaning.
Applicable Standards	Latest issue of SANS 149 Compliance certificate to be submitted with bid.
Note to Procurement	

Drainage system: chest, underwater, adult

NO TECHNICAL SPECIFICATIONS
NONE

Catalogue Number	Item Name	Available on Transversal Contract	Contract Number or Code
PHC-C-369	Drainage system: chest, underwater, adult	No	

Description	A device consisting of a clear drainage bottle, with a graduated scale to measure volume drained; a locking, non-leak cap, with an inlet and outlet. The bottle is partly-filled with water when in use. A replaceable silicon tube is provided to connect the system to an intrathoracic catheter. Sterilised, single use.
Use	For providing an underwater seal to an intrathoracic drain. The inlet is below the level of the water in the bottle, hence the system acts as a one-way valve for air or fluid in the intrapleural space.
Applicable Standards	
Note to Procurement	

Dressing pack: disposable

NO TECHNICAL SPECIFICATIONS
NONE

Catalogue Number	Item Name	Available on Transversal Contract	Contract Number or Code
PHC-C-149	Dressing pack: disposable	RT 42-2018	42311505-00307

Description	Dressing Pack: Surgical: Sterile Peel Packed.<br>Contents:<br>Gauze plain: ±100mm x 100mm; Ten<br>Gloves Non powdered, latex free, medium<br>Cleansing swabs 30mm 1 bundle of 5 balls/ or 5 cotton wool balls<br>Red plastic bag X 1<br>Disposable soft drape 90 x 90cm 2 sheets<br>Forceps x 2.
Use	Wound dressing.
Applicable Standards	SANS 446.
Note to Procurement	

ECG electrodes: disposable, self-adhesive, for resting ECG

NO TECHNICAL SPECIFICATIONS NONE

Catalogue Number	Item Name	Available on Transversal Contract	Contract Number or Code
PHC-C-212	ECG electrodes: disposable, self-adhesive, for resting ECG	No	

Description	Peel-and-stick, self-adhesive electrodes. Pre-loaded with conductive gel.
Use	Applied to patient’s chest and extremities, according to the chosen lead configuration. Each electrode has a built-in snap-on connector, to which the leads from the cardiograph are attached.
Applicable Standards	
Note to Procurement	Procured in boxes of 50 or 100. Not suitable for stress or exercise ECGs.

Endotracheal tubes: cuffed

NO TECHNICAL SPECIFICATIONS
NONE

Catalogue Number	Item Name	Available on Transversal Contract	Contract Number or Code
PHC-C-357	Endotracheal tube: cuffed size 3	RT 13	RT13-25-001
PHC-C-358	Endotracheal tube: cuffed size 4	RT 13	RT13-25-003
PHC-C-089	Endotracheal tube: cuffed size 5	RT 13	RT 13-25-005
PHC-C-090	Endotracheal tube: cuffed size 6	RT 13	RT 13-25- 007
PHC-C-091	Endotracheal tube: cuffed size 7	RT 13	RT 13-25- 009
PHC-C-092	Endotracheal tube: cuffed size 8	RT 13	RT 13-25-010

Description	Cuffed endotracheal tube, PVC reinforced with metal spiral. Atraumatic, non-kinking. 15mm outer diameter connector. Individual sterile peel pack.
Use	Securing of airway for ventilation purposes, usually in larger children or adults. Inflatable cuff ensures seal inside trachea.
Applicable Standards	ISO 5361:2016.
Note to Procurement	

Endotracheal tubes: uncuffed

NO TECHNICAL SPECIFICATIONS
NONE

Catalogue Number	Item Name	Available on Transversal Contract	Contract Number or Code
PHC-C-086	Endotracheal tube: uncuffed 2,0mm inner diameter	RT 13	RT13-22-001
PHC-C-354	Endotracheal tube: uncuffed 2,5mm inner diameter	RT 13	RT13-22-002
PHC-C-087	Endotracheal tube: uncuffed 3,0mm inner diameter	RT 13	RT 13-22-003
PHC-C-355	Endotracheal tube: uncuffed 3,5mm inner diameter	RT 13	RT13-22-004
PHC-C-088	Endotracheal tube: uncuffed 4,0mm inner diameter	RT 13	RT 13-22-005
PHC-C-356	Endotracheal tube: uncuffed 4,5mm inner diameter	RT 13	RT13-22-005

Description	Uncuffed endotracheal tube, PVC reinforced with metal spiral. Atraumatic, non-kinking. 15mm outer diameter connector. Individual sterile peel pack.
Use	Securing of airway for ventilation purposes, usually in paediatric patients.
Applicable Standards	ISO 5361:2016.
Note to Procurement	

Eye patch: disposable

NO TECHNICAL SPECIFICATIONS
NONE

Catalogue Number	Item Name	Available on Transversal Contract	Contract Number or Code
PHC-C-165	Eye patch: disposable	No	

Description	Disposable plastic eye patch, with adjustable elastic headband.
Use	For occlusion of eye during after injury or treatment.
Applicable Standards	
Note to Procurement	

Gauze: absorbent, Type 1, sterile, peel pack (5 swabs)

NO TECHNICAL SPECIFICATIONS
NONE

Catalogue Number	Item Name	Available on Transversal Contract	Contract Number or Code
PHC-C-150	Gauze: absorbent, Type 1, sterile, peel pack (5 swabs)	RT 42-2018	42311505-00070

Description	Swabs, gauze, absorbent, type 1, bleached, sterile X-Ray detectable, 12 ply, 1 x 5 swabs Size: 100mm x 100mm Sterile peel pack.
Use	Wound and burn cleaning and dressing.
Applicable Standards	SANS 446.
Note to Procurement	

Gauze: paraffin, 100X100 (box of 10)

NO TECHNICAL SPECIFICATIONS NONE

Catalogue Number	Item Name	Available on Transversal Contract	Contract Number or Code
PHC-C-152	Gauze: paraffin, 100X100 (box of 10)	RT 42-2018	42311505-00101

Description	Dressing, paraffin gauze, B.P., 10 dressings Size: 100mmx100mm Sterile individually peel packed Box.
Use	Burns and skin graft dressing.
Applicable Standards	British Pharmacopoeia standard for Paraffin Gauze Dressing (Tulle Gras). Proof must be submitted.<br>MCC product registration must be submitted.
Note to Procurement	

Gauze: swabs, plain

NO TECHNICAL SPECIFICATIONS
NONE

Catalogue Number	Item Name	Available on Transversal Contract	Contract Number or Code
PHC-C-151	Gauze: swabs plain, 100x100X8ply (pack of 100)	RT 42-2018	42311505-00063

Description	Swabs, gauze, absorbent, type 3, bleached, 8 ply, 100 swabs Size: 100mm x 100mm Non sterile.
Use	Wound cleaning.
Applicable Standards	SANS 446.
Note to Procurement	

Intravenous administration set

NO TECHNICAL SPECIFICATIONS
NONE

Catalogue Number	Item Name	Available on Transversal Contract	Contract Number or Code
PHC-C-095	Intravenous administration set 20 drops/ml	No	
PHC-C-096	Intravenous administration set 60 drops/ml	No	

Description	Set with pointed cannula for insertion into container of intravenous fluid, drip chamber (with regulated drop pipette), 1,8m clear flexible tubing terminating in Luer lock male adapter.<br>Flow-regulating clamp. Y-injection site. Sterile fluid path. Packed in sterile tamper-proof peel pack.<br>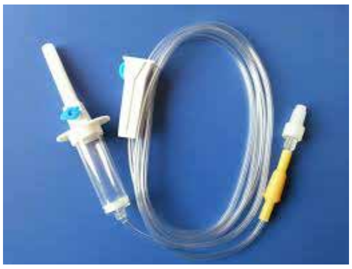
Use	For intravenous therapy with fluids alone, or fluids and medication.<br>Adult set: 20 drops per ml.<br>Paediatric set: 60 drops per ml.
Applicable Standards	SANS 1775-1:2011.
Note to Procurement	

Intravenous cannula

NO TECHNICAL SPECIFICATIONS
NONE

Catalogue Number	Item Name	Available on Transversal Contract	Contract Number or Code
PHC-C-097	Intravenous cannula: 18g green	No	
PHC-C-098	Intravenous cannula: 20g pink	No	
PHC-C-099	Intravenous cannula: 22g blue	No	
PHC-C-100	Intravenous cannula: 24g yellow	No	

Description	Intravenous cannula with stylet, in various sizes. Individual sterile peel pack.	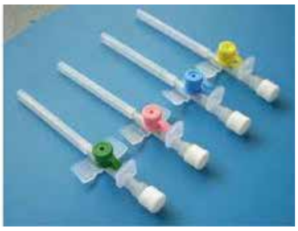
Use	To establish secure access to a vein, for intravenous infusion or therapy.	
Applicable Standards	SANS 327: 201.	
Note to Procurement		

Intravenous cannula: winged

NO TECHNICAL SPECIFICATIONS
NONE

Catalogue Number	Item Name	Available on Transversal Contract	Contract Number or Code
PHC-C-198	Intravenous cannula: winged	No	

Description	"Butterfly" cannula, with attached plastic wings for fixation. Female Luer-type port. Individually packed. Sterile. 22g.
Use	Primarily used for scalp vein access in very small children.
Applicable Standards	SANS 305 :2011.
Note to Procurement	

Lubricant gel, sterile

NO TECHNICAL SPECIFICATIONS NONE

Catalogue Number	Item Name	Available on Transversal Contract	Contract Number or Code
PHC-C-172	Lubricant gel, sterile	No	

Description	Aqueous gel in tubes or sterile peel packs.
Use	For examinations or instrument insertions, where lubrication is required.
Note to Procurement	

Mask: laryngeal, adult

NO TECHNICAL SPECIFICATIONS
NONE

Catalogue Number	Item Name	Available on Transversal Contract	Contract Number or Code
PHC-C-103	Laryngeal mask: adult (50-90kg) size 4.0	RT 13	RT 13-15-006
PHC-C-104	Laryngeal mask: adult (90+ kg) size 5.0	RT 13	RT 13-15-007
PHC-C-351	Laryngeal mask: size 2	RT 13	RT13-11-003
PHC-C-352	Laryngeal mask: size 2,5	RT 13	RT13-11-004
PHC-C-353	Laryngeal mask: size 3	RT 13	RT13-11-005

Description	Second generation supraglottic airway Soft, gel-like, non-inflatable cuff, anatomical design.<br>Two sizes: for smaller and larger adults.	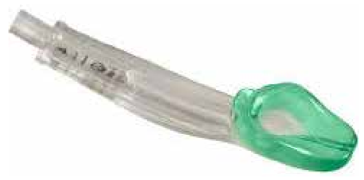
Use	Has been shown to be easier to use by first responders, does not traumatise the vocal cords. Works by sealing the supraglottic area. No inflation of cuff required.	
Applicable Standards	ISO 11712:2009.	
Note to Procurement		

Mask: oxygen, diluter type

NO TECHNICAL SPECIFICATIONS NONE

Catalogue Number	Item Name	Available on Transversal Contract	Contract Number or Code
PHC-C-107	Mask: oxygen (diluter type) Paediatric 24%	RT 13	RT 13-41-001
PHC-C-108	Mask: oxygen (diluter type) Adult 24%	RT 13	RT 13-42-001
PHC-C-109	Humidifier pre-filled water pack	RT 13	RT 13-48-002

Description	Oxygen delivery mask with venturi that regulates the delivered oxygen concentration.	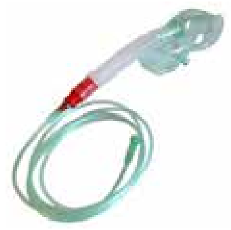
Use	For oxygen delivery to patient who is breathing spontaneously. A pre-filled water pack can be connected in line with the diluter masks, to moisten inspired air.	
Applicable Standards	ISO 11712:2009.	
Note to Procurement		

Mask: oxygen, re-breathing type

NO TECHNICAL SPECIFICATIONS NONE

Catalogue Number	Item Name	Available on Transversal Contract	Contract Number or Code
PHC-C-105	Mask: oxygen (re-breathing type) Paediatric	RT 13	RT 13-43-001
PHC-C-106	Mask: oxygen (re-breathing type) Adult	RT 13	RT 13-43-002

Description	Oxygen delivery mask with under-chin re-breathing bag.	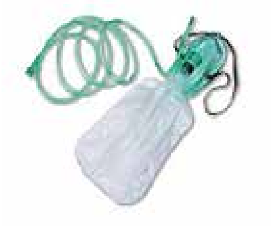
Use	For oxygen delivery to patient who is breathing spontaneously.	
Applicable Standards	ISO 11712:2009.	
Note to Procurement		

Nasal Cannula: oxygen

NO TECHNICAL SPECIFICATIONS
NONE

Catalogue Number	Item Name	Available on Transversal Contract	Contract Number or Code
PHC-C-110	Nasal cannula: oxygen, paediatric	RT 13	RT 13-45-004
PHC-C-111	Nasal cannula: oxygen, adult	RT 13	RT 13-43-005
PHC-C-112	Humidifier pre-filled water pack	RT 13	RT 13-48-002

Description	Oxygen delivery nasal cannula.	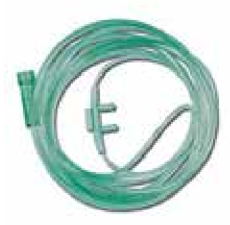
Use	For oxygen delivery to patient who is breathing spontaneously.<br>Over-the-ear style nasal cannula.<br>Retaining strap.<br>2 m. crush-proof tubing.<br>Can be used with pre-filled water pack to supply humidification during oxygen therapy.	
Applicable Standards	ISO/DIS 17256 (when it comes into force).	
Note to Procurement		

Nasogastric catheter

Catalogue Number	Item Name	Available on Transversal Contract	Contract Number or Code
PHC-C-130	Nasogastric tube: 600mm F 8	No	Nasogastric tube, 600mm FG 8
PHC-C-131	Nasogastric tube: 1000mm F 10	No	Nasogastric tube, 1000mm FG10
PHC-C-132	Nasogastric tube: 1000mm F 12	No	Nasogastric tube, 1000mm FG12
PHC-C-342	Nasogastric tube: 600mm F 5	No	

Description	Smooth PVC catheter, with round insertion end, funnel- shaped proximal end.<br>Sterile peel pack.	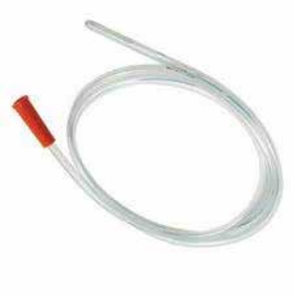
Use	For draining the gastric system in obstruction, or direct feeding when patient cannot swallow.<br>Verify correct placement in stomach prior to any feeding or lavage.	
Applicable Standards		
Note to Procurement		

Needles: hypodermic

NO TECHNICAL SPECIFICATIONS
NONE

Catalogue Number	Item Name	Available on Transversal Contract	Contract Number or Code
PHC-C-122	Needle: hypodermic 18g pink	No	
PHC-C-123	Needle: hypodermic 20g yellow	No	
PHC-C-124	Needle, hypodermic 21g green	No	
PHC-C-125	Needle: hypodermic 22g black	No	
PHC-C-126	Needle: hypodermic 23g blue	No	

Description	Needle, hypodermic, for administering parenteral therapy.	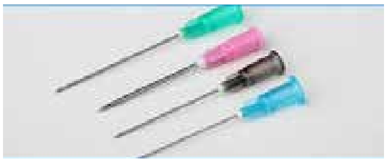
Use	Long-point needle with plastic Luer-lock hub, colour-coded according to size.<br>Individual sterile peel pack.	
Applicable Standards	SANS1124-1:2011.	
Note to Procurement		

Needles: Hypodermic, Dental

NO TECHNICAL SPECIFICATIONS NONE

Catalogue Number	Item Name	Available on Transversal Contract	Contract Number or Code
PHC-C-176	Needle: hypodermic, 30g short	RT296-2015	RT296-01-001
PHC-C-177	Needle: hypodermic, 27g long	RT296-2015	RT296-01-002

Description	Needle, hypodermic: disposable, silicone coated, plastic hub with universal thread, sterile.<br>30g short: 0,3 x 25 mm, 30 gauge short, Lancet bevel point, Box of 100.<br>27g long: 0,4 x38 mm, 27 gauge long, Lancet bevel point, Box of 100.
Use	Administration of local anaesthetic.
Applicable Standards	SANS 1188:1978.
Note to Procurement	

Padding: cast orthopaedic

NO TECHNICAL SPECIFICATIONS NONE

Catalogue Number	Item Name	Available on Transversal Contract	Contract Number or Code
PHC-C-378	Padding: cast orthopaedic 50mm	RT 42/HM02 2015BD	45.01
PHC-C-379	Padding: cast orthopaedic 100mm	RT 42/HM02 2015BD	45.03
PHC-C-380	Padding: cast orthopaedic 150mm	RT 42/HM02 2015BD	45.04

Description	Natural bonded type orthopaedic padding, type 2. In 3m rolls.
Use	Used as padding before application of a cast or splint.
Applicable Standards	SANS 196.
Note to Procurement	

Plaster of Paris bandage

NO TECHNICAL SPECIFICATIONS NONE

Catalogue Number	Item Name	Available on Transversal Contract	Contract Number or Code
PHC-C-381	Plaster of Paris bandage 100mm x 3,5m	RT 42/HM02 2015BD	12.03
PHC-C-382	Plaster of Paris bandage 150mm x 3,5m	RT 42/HM02 2015BD	12.04
PHC-C-383	Plaster of Paris bandage 100mm	RT 42/HM02 2015BD	12.05

Description	Leno weave base cloth Type 1, impregnated with anhydrous calcium sulphate hemihydrate. Individually packed in sealed, moisture-proof containers.
Use	Soaked in water, applied as a splint or cast, and allowed to dry and harden.
Applicable Standards	SANS 1308.
Note to Procurement	

Peak Flow Meter: mouth-piece, disposable

NO TECHNICAL SPECIFICATIONS
NONE

Catalogue Number	Item Name	Available on Transversal Contract	Contract Number or Code
PHC-C-341	Peak Flow Meter: mouth-piece, disposable	No	

Description	Non-plastic tube (waxed card)which is inserted into mouth-piece end of the Peak Flow Meter.
Use	To prevent direct contact between mouth and instrument in a multi-patient environment.
Applicable Standards	
Note to Procurement	Must fit PHC-F-267 – not interchangeable.

Oropharyngeal airway (Guedel)

NO TECHNICAL SPECIFICATIONS
NONE

Catalogue Number	Item Name	Available on Transversal Contract	Contract Number or Code
PHC-C-113	Oropharyngeal airway (Guedel): size 0	RT 13	RT 13-1-005
PHC-C-114	Oropharyngeal airway (Guedel): size 1	RT 13	RT 13-1-06
PHC-C-115	Oropharyngeal airway (Guedel): size 2	RT 13	RT 13-1-007
PHC-C-116	Oropharyngeal airway (Guedel): size 3	RT 13	RT 13-1-008
PHC-C-117	Oropharyngeal airway (Guedel): size 4	RT 13	RT 13-1-009
PHC-C-350	Oropharyngeal airway(Guedel): size 5	RT 13	RT 13-1-010

Description	Oropharyngeal airway, used to keep airway open, when intubation is not necessary or practical.	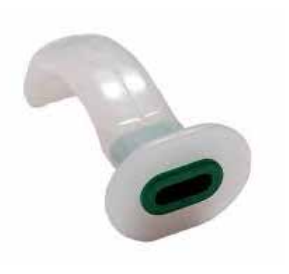
Use	Anatomically-curved plastic airway. Rigid bite block. Flanged. Surgically clean. Re-usable. Clean after use to surgically clean standards.	
Applicable Standards	ISO 5346:2016.	
Note to Procurement		

Plaster: self-adhesive, roll

NO TECHNICAL SPECIFICATIONS
NONE

Catalogue Number	Item Name	Available on Transversal Contract	Contract Number or Code
PHC-C-153	Plaster: self-adhesive, roll	RT 42-2018	42311707-00010

Description	Tape, zinc oxide, surgical, adhesive, B.P., woven cotton cloth, non-stretch Size: 75mm x 5 m individually wrapped roll.
Use	Firm compression over dressings.<br>Wound protection.
Applicable Standards	British Pharmacopoeia standard for Zinc Oxide Surgical Adhesive Tape. Proof must be submitted.
Note to Procurement	

Razor, disposable

NO TECHNICAL SPECIFICATIONS NONE

Catalogue Number	Item Name	Available on Transversal Contract	Contract Number or Code
PHC-C-166	Razor, disposable	No	

Description	Disposable single-blade razor.
Use	For removal of hair prior to procedure or wound suturing.
Applicable Standards	None available.
Note to Procurement	

Sanitary towels: standard

NO TECHNICAL SPECIFICATIONS
NONE

Catalogue Number	Item Name	Available on Transversal Contract	Contract Number or Code
PHC-C-157	Sanitary towels: standard	No	

Description	Normal usage sanitary towels. Pack.
Use	
Applicable Standards	
Note to Procurement	

Sheath: incontinence

NO TECHNICAL SPECIFICATIONS
NONE

Catalogue Number	Item Name	Available on Transversal Contract	Contract Number or Code
PHC-C-373	Sheath: incontinence 25mm	No	
PHC-C-374	Sheath: incontinence 30mm	No	
PHC-C-375	Sheath: incontinence 35mm	No	

Description	Latex-free penile sheath.
Use	For management of incontinence.
Applicable Standards	
Note to Procurement	

Skin traction kit

NO TECHNICAL SPECIFICATIONS
NONE

Catalogue Number	Item Name	Available on Transversal Contract	Contract Number or Code
PHC-C-376	Skin traction kit: adult	No	
PHC-C-377	Skin traction kit: child	No	

Description	A complete kit for applying traction to a lower limb.
Use	A foot plate is attached to the skin of the lower limb, with a fabric strip impregnated with a pressure-sensitive adhesive. Adhesion is reinforced by applying a conformable elastic bandage over the strip. Traction is applied to the foot-plate.
Applicable Standards	
Note to Procurement	

Stockinette: 100mm, roll 25m

NO TECHNICAL SPECIFICATIONS
NONE

Catalogue Number	Item Name	Available on Transversal Contract	Contract Number or Code
PHC-C-154	Stockinette: 100mm, roll 25m	RT 42-2018	42311505-00018

Description	Bandage, tubular, orthopaedic, knitted, cotton-type, stockinette, unbleached Size: width: 100mm Size: length: 25m roll, individually boxed.
Use	For securing dressings over upper extremities. Very light compression.
Applicable Standards	SANS 925.
Note to Procurement	

Stockinette: 150mm, roll 25m

NO TECHNICAL SPECIFICATIONS
NONE

Catalogue Number	Item Name	Available on Transversal Contract	Contract Number or Code
PHC-C-186	Stockinette: 150mm, roll 25m	RT 42-2018	42311505-00019

Description	Bandage, tubular, orthopaedic, knitted, cotton-type, stockinette, unbleached size: width: 150mm size: length: 25m roll, individually boxed.
Use	For securing dressings over lower extremities.<br>Very light compression.
Applicable Standards	SANS 925.
Note to Procurement	

Suture materials

NO TECHNICAL SPECIFICATIONS
NONE

Catalogue Number	Item Name	Available on Transversal Contract	Contract Number or Code
PHC-C-180	Suture: chromic, 0/0, 75cm	RT 302-2016	42312201-00157
PHC-C-181	Suture: sterile, nylon, 2/0, 3/8, 45cm	RT 302-2016	42312201-00340
PHC-C-182	Suture: sterile, nylon, 4/0, 3/8, 45cm	RT 302-2016	42312201-00331

Description	Swaged needles with suturing thread.
Use	For closure of skin and sub-dermal layers.<br>Chromic with swaged needle for subdermal suturing.<br>Nylon with swaged needle in 2 sizes, for dermal suturing.
Applicable Standards	SANS 494-1:2011.
Note to Procurement	

Swab: 70% isopropyl alcohol

NO TECHNICAL SPECIFICATIONS NONE

Catalogue Number	Item Name	Available on Transversal Contract	Contract Number or Code
PHC-C-155	Swab: 70% isopropyl alcohol	RT 42-2018	42311505-00053

Description	24mm X 30mm viscose rayon non-woven fabric.<br>Sterile, individually packed in aluminium foil pouches.<br>Minimum of 0,5ml Isopropyl Alcohol 70% in water.
Use	Skin preparation prior to injection or venepuncture.
Applicable Standards	Must be registered with MCC (proof of registration required).
Note to Procurement	

Syringe: hypodermic

NO TECHNICAL SPECIFICATIONS
NONE

Catalogue Number	Item Name	Available on Transversal Contract	Contract Number or Code
PHC-C-118	Syringe: 3-part; 2ml	No	
PHC-C-119	Syringe: 3-part; 5ml	No	
PHC-C-120	Syringe: 3-part; 10ml	No	
PHC-C-121	Syringe: insulin	No	
PHC-C-349	Syringe: 3-part, 20ml	No	

Description	Single-use hypodermic syringe, for the administration of parenteral therapy.	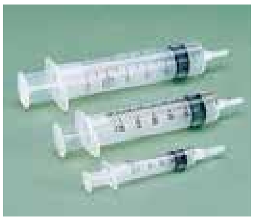 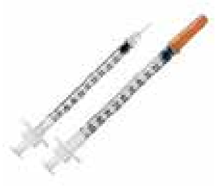
Use	3-part disposable syringe in individual sterile peel pack.<br>Insulin syringe is complete with needle.	
Applicable Standards	SANS 1124-2:2011.<br>Insulin syringe SANS 1166:2011.	
Note to Procurement		

Tampon: vaginal

NO TECHNICAL SPECIFICATIONS NONE

Catalogue Number	Item Name	Available on Transversal Contract	Contract Number or Code
PHC-C-158	Tampon: vaginal	No	

Description	Tampon, vaginal, autoclavable Absorbent cotton wool with gauze made into a tube. Each.
Use	
Applicable Standards	Cotton wool to comply with the latest issue of SANS 228 Absorbent cotton gauze to comply with the latest issue of SANS 446 Compliance certificate to be submitted with bid.
Note to Procurement	

Tape: surgical. Adhesive, microporous, 25mm

NO TECHNICAL SPECIFICATIONS NONE

Catalogue Number	Item Name	Available on Transversal Contract	Contract Number or Code
PHC-C-159	Tape, surgical. Adhesive, microporous, 25mm	RT 42-2018	42311707-00001

Description	Tape, surgical, adhesive film, microporous paper consisting of non-woven synthetic fibres spread with a non-occlusive acrylic adhesive.<br>Size: 25mm x 5m.<br>Individually-wrapped or boxed roll.<br>This type of adhesive has been associated with colonisation of partially- used rolls by Methicillin-Resistant Staphylococcus Aureus, as well as Vancomycin-Resistant Enterococci. In the absence of new antibiotics, healthcare workers must ensure that contamination is avoided, but practicing appropriate hand-washing, and storage of unused tape.<br>If left on the skin without monitoring, there is an association with Medical Adhesive-Related skin Injury.
Use	Skin closure; securing of dressings, cannulas.
Applicable Standards	Surgical tape must permit skin ventilation, be hypo-allergenic, be tolerated by skin for prolonged periods without irritation.<br>BP standards for Permeable Non-woven Surgical Synthetic Adhesive Tape.
Note to Procurement	

Tape: surgical. Adhesive, microporous, 50mm

NO TECHNICAL SPECIFICATIONS NONE

Catalogue Number	Item Name	Available on Transversal Contract	Contract Number or Code
PHC-C-185	Tape, surgical. Adhesive, microporous, 50mm	RT 42-2018	42311707-00002

Description	Tape, surgical, adhesive film, microporous paper consisting of non-woven synthetic fibres spread with a non-occlusive acrylic adhesive.<br>Size: 50mmx5m.<br>Individually wrapped roll to Individually-wrapped or boxed roll.<br>This type of adhesive has been associated with colonisation of partially- used rolls by Methicillin-Resistant Staphylococcus Aureus, as well as Vancomycin-Resistant Enterococci. In the absence of new antibiotics, healthcare workers must ensure that contamination is avoided, but practicing appropriate hand-washing, and storage of unused tape.<br>If left on the skin without monitoring, there is an association with Medical Adhesive-Related skin Injury.”
Use	Skin closure; securing of dressings, cannulas.
Applicable Standards	Surgical tape must permit skin ventilation, be hypo-allergenic, be tolerated by skin for prolonged periods without irritation.<br>BP standards for Permeable Non-woven Surgical Synthetic Adhesive Tape.
Note to Procurement	

Thermal blanket

NO TECHNICAL SPECIFICATIONS NONE

Catalogue Number	Item Name	Available on Transversal Contract	Contract Number or Code
PHC-C-179	Thermal blanket	RT 4-2015	RT 4-05-049 ME

Description	Aluminium foil laminated in plastic sheeting.
Use	Used during resuscitation of hypothermia cases. Used in combination with conventional blanket. Disposable. Individually packaged.
Applicable Standards	None available.
Note to Procurement	

Thermometer probe covers: disposable

NO TECHNICAL SPECIFICATIONS NONE

Catalogue Number	Item Name	Available on Transversal Contract	Contract Number or Code
PHC-C-178	Thermometer probe covers: disposable	No	

Description	Disposable covers for ear probe of thermometer.
Use	Disposable probe cover for ear-type thermometer, to avoid cross-contamination.<br>Thermometers typically have an eject button, so that the user does not need to touch the used cover.
Applicable Standards	
Note to Procurement	This item must be in series with item chosen for PHC-E-130.

Tube: stomach washout

NO TECHNICAL SPECIFICATIONS
NONE

Catalogue Number	Item Name	Available on Transversal Contract	Contract Number or Code
PHC-C-369	Tube: stomach washout 24F	No	
PHC-C-370	Tube: stomach washout 26F	No	
PHC-C-371	Tube: stomach washout 28F	No	
PHC-C-372	Tube: stomach washout 30F	No	

Description	Large-bore oro-gastric tubes.
Use	Used for rapid evacuation of stomach contents and lavage of stomach.
Applicable Standards	
Note to Procurement	

Ultrasound gel

NO TECHNICAL SPECIFICATIONS NONE

Catalogue Number	Item Name	Available on Transversal Contract	Contract Number or Code
PHC-C-173	Ultrasound gel: medium viscosity	No	

Description	Tube of gel for use in ultrasonography. Non-sterile.
Use	Improves contact between ultrasound probe and skin, and therefore transmission of ultrasound.
Applicable Standards	Aqueous. Hypoallergenic. Bacteriostatic.
Note to Procurement	

Urinary catheters (Foley's)

NO TECHNICAL SPECIFICATIONS
NONE

Catalogue Number	Item Name	Available on Transversal Contract	Contract Number or Code
PHC-C-343	Urinary catheter (Foley's): 8F	No	
PHC-C-344	Urinary catheter (Foley's): 10F	No	
PHC-C-345	Urinary catheter (Foley's): 12F	No	
PHC-C-127	Urinary catheters, (Foley's): 14F	No	
PHC-C-346	Urinary catheter (Foley's): 16F	No	
PHC-C-128	Urinary catheters, (Foley's): 18F	No	
PHC-C-347	Urinary catheter (Foley's): 20F	No	
PHC-C-348	Urinary catheter (Foley's): 22F	No	
PHC-C-129	Urinary bag	No	

Description	Urinary catheter, Foley pattern, for draining the urinary system when patient's ability to urinate is restricted, or the patient is incontinent. 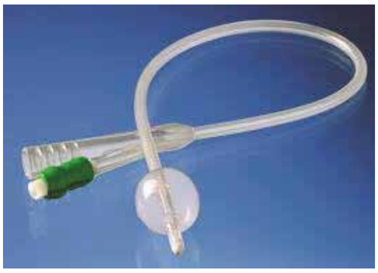
Use	Smooth PVC catheter, with round insertion end, funnel- shaped proximal end.<br>Inflatable bulb when used as in-dwelling catheter.<br>Sterile peel pack. Can be used with urine bag.
Applicable Standards	SANS 51616:1997.
Note to Procurement	

Point of Care Testing

Glucometer consumables

NO TECHNICAL SPECIFICATIONS NONE

Catalogue Number	Item Name	Available on Transversal Contract	Contract Number or Code
PHC-C-181	Glucometer test strips	No	

Description	The glucometer test strips have an absorbent pad, which contains chemicals that bind with glucose, and in so doing, produce a measurable electric current, or a colour change, depending on the technology used by the meter. The meter reads the current or the intensity of the colour, and converts it into a concentration.
Use	A lancets is used to obtain a drop of capillary blood from the patient (usually from a finger). The drop is absorbed on the pad of the test strip. The test strip is then placed inside the appropriate slot in the meter.<br>Observe strict cross-infection control measures.<br>Biosafety waste disposal containers are mandatory.
Applicable Standards	None available.
Note to Procurement	The suppliers of meters and test strips often supply a number of meters at no cost, based on the volumes of strips purchased. it is therefore important to standardise on the type/model of meter, at least regionally, if not nationally.

Haemoglobinmeter consumables and accessories (older models)

NO TECHNICAL SPECIFICATIONS
NONE

Catalogue Number	Item Name	Available on Transversal Contract	Contract Number or Code
PHC-C-167	Haemolysis applicator sticks	No	
PHC-C-168	Haemoglobinmeter clip	No	
PHC-C-169	Haemoglobin chamber: glass, grooved	No	
PHC-C-170	Haemoglobin cover glass: plain	No	

Description	These consumables and accessories are specific to the older type of haemoglobinometer, many of which are still in use.
Use	The capillary blood sample obtained from a lancet-prick, is placed on the glass chamber. The haemolysis applicator stick is swirled inside the blood sample, until it has a uniform translucent appearance. The chamber is then covered with a glass cover slip, and the chamber plus cover slip are inserted into a metal clip.<br>The combined unit is placed into a slot in the haemoglobinometer.<br>A sliding knob is positioned until two images in the viewfinder match in colour. The haemoglobin concentration is then read from a scale on the side of the instrument.<br>The chamber and clip are re-usable. The cover slips and applicator sticks are disposable.<br>Observe strict cross-infection control measures.<br>Biosafety waste disposal containers are mandatory.<br>Wash the chamber and clip thoroughly between uses.
Applicable Standards	None available.
Note to Procurement	None.

Haemoglobinmeter consumables (newer models)

NO TECHNICAL SPECIFICATIONS NONE

Catalogue Number	Item Name	Available on Transversal Contract	Contract Number or Code
PHC-C-182	Hb meter test strips	No	
PHC-C-183	Hb meter microcuvette	No	

Description	Modern electronic haemoglobinometers operate on one of two principles: either by measuring the amount of light of a specific wavelength passing through (or reflected from) a blood sample, or by measuring the intensity of colour change, when blood is mixed with a reagent.
Use	In colorimetric haemoglobinometers, a lancet-prick blood drop is applied to the reagent-containing pad on a test strip.<br>The strip is inserted into the meter, and a reading is obtained.<br>In photometric haemoglobinometers, the blood drop is applied to a microcuvette. The microcuvette is placed in the instrument, and a reading is obtained.<br>All consumables are disposable.<br>Observe strict cross-infection control measures.<br>Biosafety waste disposal containers are mandatory.
Applicable Standards	None available.
Note to Procurement	The suppliers of test strips or microcuvettes may provide a number of free meters, depending on volumes purchased.<br>Hence it is important to standardise regionally, if not nationally.<br>Test strips and microcuvettes are meter-specific, and are not interchangeable among brands.

HIV test kit: rapid

NO TECHNICAL SPECIFICATIONS
NONE

Catalogue Number	Item Name	Available on Transversal Contract	Contract Number or Code
PHC-C-199	HIV test kit: rapid	RT 41-2017	

Description	Cassette-based Rapid test kit for screening for HIV.
Use	For rapid screening for HIV in human serum, plasma, venous or capillary whole blood.<br>The contract was awarded to 5 suppliers. Specifications and Instructions for use vary with supplier.<br>Refer to instructions supplied by the supplier.<br>All kits have in common chromatographic process, in which HIV antibodies, if present, cause one or more coloured lines to appear.<br>Observe strict cross-infection control measures.<br>Biosafety waste disposal containers are mandatory.
Applicable Standards	WHO pre-qualification.
Note to Procurement	The current RT 41 appears to contain kits which are WHO pre-qualified, as well as some which may not be. Future procurement should ensure uniformity of quality.

Lancets, Multipurpose

NO TECHNICAL SPECIFICATIONS NONE

Catalogue Number	Item Name	Available on Transversal Contract	Contract Number or Code
PHC-C-146	Lancets, multipurpose	No	

Description	A single-use, sharp-pointed, sterile, disposable device, containing a short, sharpened needle, and a twist-off protective cap. There are three types of devices:<br>a. Traditional simple stamped – metal strip, with point at one end<br>b. The second type has a plastic body, with an integral twist-off needle cap. Can be used manually, but is often sold with a separate lancing device, into which it is inserted. In this type, the protective cap must be re-installed before discarding<br>c. Spring-loaded – after puncturing the skin, the needle is retracted for safety, and the device cannot be re-used.<br>Individually packed, in Boxes. Needle size 23 Gauge or smaller, protrusion depth approx. 2mm. Stainless steel needle.	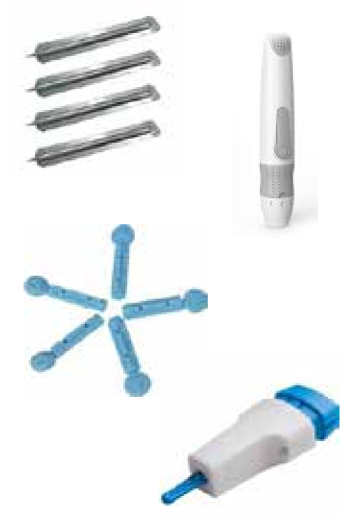
Use	The device is used to obtain a capillary blood sample from a finger-prick. For repeated daily use by a patient, the automatic lancing device is recommendable to lessen pain.<br>Observe skin cleansing and sharps procedures. Observe cross-contamination procedures. Sharps disposal only. Danger of accidental finger-stick in non-self-retracting types.<br>Biosafety waste disposal containers are mandatory.	
Applicable Standards	ISO 23908:2011 Sharps injury protection.	
Note to Procurement	Self-retracting type is preferable, if price is affordable. Factor in the cost of lancing devices for non-self-retracting types.	

Malaria Rapid Diagnostic test kit

NO TECHNICAL SPECIFICATIONS NONE

Catalogue Number	Item Name	Available on Transversal Contract	Contract Number or Code
PHC-C-200	Malaria Rapid Diagnostic test kit	No	

Description	Cassette-type rapid test kit for malaria screening.	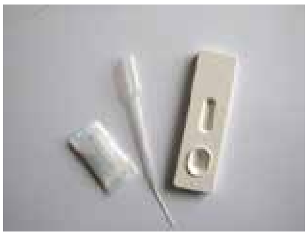
Use	For screening of suspected malaria cases in provinces where malaria is endemic. Not a replacement for microscopy.<br>Detects malaria antigens (proteins produced by the malaria parasite) in human blood. Capillary blood is obtained via a lancet prick.<br>Note that most test kits require a few drops of a buffer solution (supplied with the kit) to be added to the cassette, after the blood specimen.<br>A visible line forms on a chromatography strip, if antigen is present in the sample.<br>Observe strict cross-infection control measures.<br>Biosafety waste disposal containers are mandatory.	
Applicable Standards		
Note to Procurement	WHO pre-qualification would be ideal.	

Pregnancy test kit

NO TECHNICAL SPECIFICATIONS
NONE

Catalogue Number	Item Name	Available on Transversal Contract	Contract Number or Code
PHC-C-201	Pregnancy test kit	No	

Description	Strip or cassette type rapid test kit for pregnancy screening.	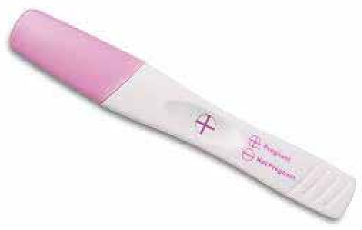
Use	Screening test on mid-stream urine. Urine collection device and pipette included with cassette type, but not with strip type.<br>Test is a antibody-based test for hCG. A colour change is produced in a line if hCG is detected.	
Applicable Standards		
Note to Procurement	In addition to the latest ISO 13485, the following information should be requested from potential suppliers:<br>Analytical sensitivity<br>Analytical specificity<br>Accuracy<br>Clinical sensitivity<br>Clinical specificity.	

Rhesus test kit

NO TECHNICAL SPECIFICATIONS
NONE

Catalogue Number	Item Name	Available on Transversal Contract	Contract Number or Code
PHC-C-204	Rhesus test kit	No	

Description	A simple screening test to determine whether a pregnant woman is Rh-negative.
Use	The test is based on red cell clumping. If the patient has Rh factor, the red cells in the sample will clump. If the patient is Rh-negative, thered cells remain un-clumped. Rh-negative women require monitoring through pregnancy. A decision to administer Anti-D immunoglobulin will be taken, should the patient's Rh status change. Failure to detect Rh-negative status, and intervention if necessary, can lead to Haemolytic disease of the Newborn, if the foetus inherits Rh from the father.
Applicable Standards	None.
Note to Procurement	

Urine dip sticks

NO TECHNICAL SPECIFICATIONS
NONE

Catalogue Number	Item Name	Available on Transversal Contract	Contract Number or Code
PHC-C-205	Urine Dip Sticks	No	

Description	Plastic test strips with reagent-impregnated mats, which change colour depending on the presence of certain substances in the patient's urine.	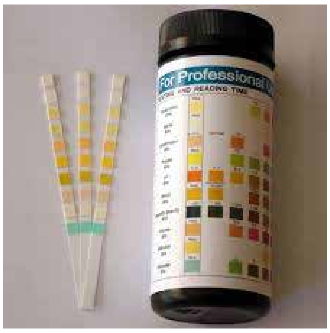
Use	The strip is dipped into a fresh, preferably mid-stream, urine sample. The time taken for the full colour to develop, will be stated by the manufacturer (typically 60 to 120 seconds).<br>The strips are multi-parameter test strips, typically testing for the presence of: Protein, glucose, ketones, haemoglobin, bilirubin, acetone, nitrites, leucocytes, pH and specific gravity. Most of the tests are semi-quantitative, i.e. they give an indication of the concentration of the substance of interest, in the urine sample.	
Applicable Standards	FDA approval or substantial equivalence determination.	
Note to Procurement	It is advisable to submit a random sample of strips from an unrecognised supplier, for comparative testing to the NHLS, before finalising procurement.	

Obstetric

Cord clamp: umbilical

NO TECHNICAL SPECIFICATIONS
NONE

Catalogue Number	Item Name	Available on Transversal Contract	Contract Number or Code
PHC-C-163	Cord clamp: umbilical	No	

Description	Self-locking hinged plastic clamp.	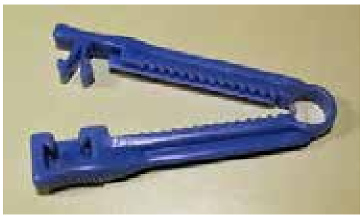
Use	To clamp off umbilical cord before severing. Sterile peel-pack, non re-usable.	
Applicable Standards	CKS 312:2009.	
Note to Procurement		

Sanitary towels: maternity

NO TECHNICAL SPECIFICATIONS
NONE

Catalogue Number	Item Name	Available on Transversal Contract	Contract Number or Code
PHC-C-156	Sanitary towels: maternity	No	

Description	Larger size sanitary towels. Pack.
Use	Post-delivery haemorrhage and discharge.
Applicable Standards	
Note to Procurement	

Oral health

Note: to avoid duplication, the following items are listed elsewhere in this Clinical Consumables section:

- Cotton wool balls
- Local anaesthetic cartridges
- Scalpel blades
- Dental needles - used widely for local anaesthetic.

Acid etch

NO TECHNICAL SPECIFICATIONS NONE

Catalogue Number	Item Name	Available on Transversal Contract	Contract Number or Code
PHC-C-220	Acid etch	RT296	RT296-02-189
PHC-C-221	Composite cement	RT296	RT296-02-038

Description	RT 296-02-189: Resin. restorative, dental: acid etch gel, 35% Ortho-phosphoric acid. 1 Bottle 6ml.<br>RT296-02-038: Cement, zinc oxide, dental: Temporary non-eugenol cement, set Set- 1 tube Base 50g, 1 tube Accelerator 15g.
Use	2 step preparation of cavity for filling. Etch is applied first, then cement.
Applicable Standards	
Note to Procurement	

Amalgam capsule

NO TECHNICAL SPECIFICATIONS
NONE

Catalogue Number	Item Name	Available on Transversal Contract	Contract Number or Code
PHC-C-222	Amalgam capsule	RT296-2015	RT296-02-040

Description	Capsules of a preparation used in dental fillings.
Use	Silver alloy powder and mercury capsule, mixing, dental: amalgam, dispersed phase, admixed, non-gamma 2 capsules, 1 spill regular set: 1 Box 50 pieces, 400 mg each.
Applicable Standards	
Note to Procurement	

Anaesthetic: topical

NO TECHNICAL SPECIFICATIONS
NONE

Catalogue Number	Item Name	Available on Transversal Contract	Contract Number or Code
PHC-C-223	Anaesthetic: topical	RT296	RT296-01-004

Description	Analgesic and anaesthetic gel: local, surface, lignocaine base, 43g tube.
Use	
Applicable Standards	

Apron: dental, plastic

NO TECHNICAL SPECIFICATIONS NONE

Catalogue Number	Item Name	Available on Transversal Contract	Contract Number or Code
PHC-C-224	Apron: plastic, adult	RT296	RT296-08-147
PHC-C-225	Apron: plastic. child	RT296	RT296-08-148

Description	Apron dental, plastic: Adult.<br>Apron dental, plastic: Child.
Use	To protect patient's clothing.
Applicable Standards	
Note to Procurement	

Articulating paper: thin, blue

NO TECHNICAL SPECIFICATIONS NONE

Catalogue Number	Item Name	Available on Transversal Contract	Contract Number or Code
PHC-C-226	Articulating paper: thin, blue	RT296	RT296-07-002

Description	Paper, articulating, dental: Thin, blue, width 20mm 1 box 12 books, 12 sheets per book.
Use	Used to check occlusion of teeth after a reconstructive procedure.
Applicable Standards	
Note to Procurement	

Bib: plastic

NO TECHNICAL SPECIFICATIONS NONE

Catalogue Number	Item Name	Available on Transversal Contract	Contract Number or Code
PHC-C-227	Bib: plastic	RT296	RT296-08-147

Description	Bib dental, disposable, 2 ply paper, 1 ply poly, Approx 40 x 45mm pack of 50.
Use	Applied to protect patient's clothing while on the chair.
Applicable Standards	
Note to Procurement	

Bonding agent: self-etch

NO TECHNICAL SPECIFICATIONS NONE

Catalogue Number	Item Name	Available on Transversal Contract	Contract Number or Code
PHC-C-228	Bonding agent: self-etch	RT296	RT296-02-240

Description	Resin restorative dental: Self- etch adhesive: 1 bottle of 5ml.
Use	1-step etching and bonding liquid, to prepare cavity for restoration.
Applicable Standards	
Note to Procurement	

Brush: applicator

NO TECHNICAL SPECIFICATIONS NONE

Catalogue Number	Item Name	Available on Transversal Contract	Contract Number or Code
PHC-C-229	Applicator brush tip	RT296	RT296-02-191

Description	Tip, applicator brush, dental: inspiral brush tips 1 container 20 pieces.
Use	For the application of acid etch and bonding agents.
Applicable Standards	
Note to Procurement	

Cement: zinc oxide and eugenol

NO TECHNICAL SPECIFICATIONS
NONE

Catalogue Number	Item Name	Available on Transversal Contract	Contract Number or Code
PHC-C-230	Cement: zinc oxide and eugenol	RT296	RT296-02-036

Description	Cement, zinc oxide and eugenol, dental lining/temporary restorative, powder and liquid , set, high compressive strength, Set: 1 bottle powder 40g, 1 bottle liquid 15ml.
Use	
Applicable Standards	
Note to Procurement	

Composites

NO TECHNICAL SPECIFICATIONS
NONE

Catalogue Number	Item Name	Available on Transversal Contract	Contract Number or Code
PHC-C-231	Composite: colour A2	RT296-2015	RT296-02-111
PHC-C-232	Composite: colour B2	RT296-2015	RT296-02-115

Description	Resin, restorative, dental: hybrid composite, ant/post restorative, light cured, with zirconium\silicon filler Capsules. 3.6g capsules (18x0.20g each).<br>RT296-02-111 colour A2 Vita.<br>RT296-02-115 colour B2 Vita.
Use	Facility to choose one of the above colours. For non-amalgam restorations.
Applicable Standards	
Note to Procurement	

Cotton wool pellets

NO TECHNICAL SPECIFICATIONS
NONE

Catalogue Number	Item Name	Available on Transversal Contract	Contract Number or Code
PHC-C-233	Cotton wool pellets	RT296	RT296-08-177

Description	Pellet, absorbent: dental cotton wool , No 4 1 box 1000 pieces.
Use	Used as packing around a tooth during removal of carious dentine, and to pack a cavity.
Applicable Standards	
Note to Procurement	

Dental floss: Waxed

NO TECHNICAL SPECIFICATIONS NONE

Catalogue Number	Item Name	Available on Transversal Contract	Contract Number or Code
PHC-C-234	Dental floss: waxed	RT296	RT296-05-017

Description	Floss, waxed, dental: Nylon 1 reel 4,5 metres.
Use	For cleaning between teeth.
Applicable Standards	
Note to Procurement	

Dry socket alveolar paste

NO TECHNICAL SPECIFICATIONS NONE

Catalogue Number	Item Name	Available on Transversal Contract	Contract Number or Code
PHC-C-235	Dry socket alveolar paste	RT296	RT296-06-016

Description	Therapeutic, post-surgical, dental: Dry socket alveolar paste, based on Iodoform, butyl-paraminobenzoate and eugenol formula 1 jar, 12g.
Use	To pack a tooth socket after extraction, when a blood clot has not formed, and there is a risk of infection. The paste forms a barrier, and has analgesic and anaesthetic properties. It aids haemostasis. It is gradually elminated by mouth and tongue action.
Applicable Standards	
Note to Procurement	

Fissure sealants

NO TECHNICAL SPECIFICATIONS NONE

Catalogue Number	Item Name	Available on Transversal Contract	Contract Number or Code
PHC-C-236	Sealant: glass ionomer kit	RT296-2015	RT296-02-174

Description	Sealant glass ionomer kit, dental, pit and fissure: Temporary sealant for air abrasive technique coloured chemical cured and light cured capsules.
Use	To seal pits and fissures in teeth.
Applicable Standards	
Note to Procurement	

Fluoride trays

NO TECHNICAL SPECIFICATIONS
NONE

Catalogue Number	Item Name	Available on Transversal Contract	Contract Number or Code
PHC-C-242	Fluoride tray: large		RT296-05-006
PHC-C-243	Fluoride tray: medium		RT296-05-007
PHC-C-244	Fluoride tray: small		RT296-05-008

Description	RT296-05-006: Fluoride application tray, dental: Disposable, large double arc h 1 Pack 100 Pieces.<br>RT296-05-007: Fluoride application tray, dental: Disposable, medium double arch 1 Pack 100 Pieces.<br>RT296-05-008: Fluoride application tray, dental: Disposable, small double arch 1 Pack 100 Pieces.
Use	For the prophylactic application of fluoride gel to upper and lower dental arches.
Applicable Standards	
Note to Procurement	

Fluoride gel

NO TECHNICAL SPECIFICATIONS NONE

Catalogue Number	Item Name	Available on Transversal Contract	Contract Number or Code
PHC-C-245	Gel: fluoride, APF	RT296	RT296-05-002
PHC-C-246	Gel: fluoride, 2,0%	RT296	RT296-05-003
PHC-C-247	Gel: fluoride, varnish	RT296	RT296-05-004
PHC-C-238	Resin: protective sealant	RT296-2015	RT296-02-180

Description	RT296-05-002: Fluoride, topical application, dental: APF fluoride gel. 1 bottle.<br>RT296-05-003: Sodium Fluoride Oral Solution, USP: Dental, Topical application, neutral, 2,0 % 1Bottle.<br>RT296-05-004: Sodium fluoride GEL: Dental, Topical application, varnish 50 mg sodium fluoride per ml, fluoride varnish 1 Tube 10ml.<br>RT296-02-180: Resin, restorative, dental: protective sealant (desensitiser and fluoride releaser), Standard package, 1 bottle applicator tips and dishes.
Use	Used in preventive dentistry to enhance tooth resistance to decay.
Applicable Standards	
Note to Procurement	

Glass ionomer

NO TECHNICAL SPECIFICATIONS NONE

Catalogue Number	Item Name	Available on Transversal Contract	Contract Number or Code
PHC-C-248	Glass ionomer: restorative	RT296	RT296-02-042

Description	Glass ionomer, restorative, dental: posterior restorations, primary teeth restorations, core build-up, capsule introductory kit.
Use	For tooth restoration.
Applicable Standards	
Note to Procurement	

Haemostat sponge

NO TECHNICAL SPECIFICATIONS
NONE

Catalogue Number	Item Name	Available on Transversal Contract	Contract Number or Code
PHC-C-249	Haemostat sponge	RT296	RT296-06-012

Description	Haemostatic agent, dental: Haem fibrin sponges, resorbable. 1 Jar 24 sponges.
Use	To staunch bleeding after intra-oral procedures.
Applicable Standards	Non-pyrogenic, biocompatible.
Note to Procurement	

Paste: prophylaxis

NO TECHNICAL SPECIFICATIONS NONE

Catalogue Number	Item Name	Available on Transversal Contract	Contract Number or Code
PHC-C-250	Paste: prophylaxis	RT296	RT296-05-011

Description	Paste, preventive dentistry with fluoride, medium grit, flavoured 1 Box 200 cups with 2 plastic finger rings.
Use	Fluoride paste for caries prophylaxis.
Applicable Standards	
Note to Procurement	

Polishing kit

NO TECHNICAL SPECIFICATIONS NONE

Catalogue Number	Item Name	Available on Transversal Contract	Contract Number or Code
PHC-C-251	Finishing and polishing kit	RT 296	RT296-11-152

Description	Finishing and polishing kit: composite restorations, discs, points, cups, mandrels and gloss kit: 20 finishing discs, 10 finishing cups, 10 finishing points, 25 polishing cups, 1 metal mandrel, 1 x 4g syringe prisma gloss, 1 x 4g syringe prisma gloss extra fine.
Use	Finishing and polishing kit for composite restorations.
Applicable Standards	
Note to Procurement	

Polishing strips

NO TECHNICAL SPECIFICATIONS
NONE

Catalogue Number	Item Name	Available on Transversal Contract	Contract Number or Code
PHC-C-252	Polishing strip: coarse/medium grit	RT296	RT296-08-002
PHC-C-253	Polishing strip: fine/superfine grit	RT296	RT296-08-003

Description	Strip, abrasive, dental: Plastic backed, coarse/medium grit, centre gap 1 box 120 pieces.<br>RT296-08-002: Coarse/medium grit.<br>RT296-08-003: Fine/superfine grit.
Use	For shaping, polishing and finishing restorations.
Applicable Standards	
Note to Procurement	

Polyester strips (composite)

NO TECHNICAL SPECIFICATIONS NONE

Catalogue Number	Item Name	Available on Transversal Contract	Contract Number or Code
PHC-C-254	Polyester strips (composite), curved	RT296	RT296-08-004
PHC-C-255	Polyester strips (composite), straight	RT296	RT296-08-005

Description	Strip, matrix, dental: composite technique, polyester, curved 10mm 1 box.<br>Strip, matrix, dental: composite technique, polyester, straight 10mm 1 box.
Use	Thin polyester strips coated with microfine abrasive particles, for the polishing and finishing of composite restorations.
Applicable Standards	
Note to Procurement	

Suture material: intra-oral

NO TECHNICAL SPECIFICATIONS NONE

Catalogue Number	Item Name	Available on Transversal Contract	Contract Number or Code
PHC-C-330	Suture: chromic, 3/0,needle 19mm, 3/8 reverse cutting	RT302-2016	42312201-00047
PHC-C-331	Suture: chromic, 4/0,needle 19mm, 1/2 reverse cutting	RT302-2016	42312201-00036
PHC-C-332	Suture: chromic, 6/0,needle 11,5mm, 3/8 reverse cutting	RT302-2016	42312201-00034

Description	Absorbable suture material, in sizes suitable for intra-oral work.
Use	For intra-oral suturing, fine skin suturing (oral margins).
Applicable Standards	SANS494-1:2011.
Note to Procurement	

Tip: dental suction (saliva ejector)

NO TECHNICAL SPECIFICATIONS
NONE

Catalogue Number	Item Name	Available on Transversal Contract	Contract Number or Code
PHC-C-256	Tip: dental suction, high volume	RT296-2015	RT296-08-158
PHC-C-257	Tip: dental suction, low volume	RT296-2015	RT296-08-160

Description	Plastic disposable suction tip.<br>High volume, diameter 16 mm 1 pack 50 pieces.<br>Low volume with tapered removable tip, length 150 mm, soft. 1 pack 100 pieces.
Use	Disposable hooked suction tip, placed in patient's mouth for continuous removal of saliva during procedures.
Applicable Standards	
Note to Procurement	

Toothbrush

NO TECHNICAL SPECIFICATIONS
NONE

Catalogue Number	Item Name	Available on Transversal Contract	Contract Number or Code
PHC-C-258	Toothbrush: adult	RT296	RT296-05-027
PHC-C-259	Toothbrush: child	RT296	RT296-05-028
PHC-C-260	Toothbrush: baby	RT296	RT296-05-029

Description	Tooth brush dental adult, straight handle, thumb grip. Head length not exceeding 2.5 cm, flat brushing plane, soft medium textured nylon (0.08micron) round end bristles, pack of 12.<br>Tooth brush dental, child, head length not exceeding 2.3cm with rounded corners in front, flat brushing plane, soft textured nylon (0.08micron) rounded bristles, flexible bended neck, pack of 12.<br>Tooth brush dental baby, head length not exceeding 2cm with rounded corners in front, flat brushing plane, soft flexible grip, soft textured nylon (0.06micron) rounded bristles, pack of 12.
Use	
Applicable Standards	
Note to Procurement	

Toothpaste: dental, fluoride

NO TECHNICAL SPECIFICATIONS NONE

Catalogue Number	Item Name	Available on Transversal Contract	Contract Number or Code
PHC-C-261	Toothpaste: dental, fluoride 25ml	RT296	RT296-05-030
PHC-C-262	Toothpaste: dental, fluoride 50ml	RT296	RT296-05-031
PHC-C-263	Toothpaste: dental, fluoride 100ml	RT296	RT296-05-032

Description	Tooth paste dental, 25ml, active ingredient sodium fluoride and sodium lauryl sulphate not exceeding 1000ppm, ingredients be sugar free, stable in use and storage, pack of 12.<br>Tooth paste dental, 50ml, active ingredient sodium fluoride and sodium lauryl sulphate not exceeding 1000ppm, ingredients be sugar free, stable in use and storage, pack of 12.<br>Tooth paste dental, 100ml, active ingredient sodium fluoride and sodium lauryl sulphate not exceeding 1000ppm, ingredients be sugar free, stable in use and storage, pack of 12.
Use	
Applicable Standards	
Note to Procurement	

Varnish: cavity liner

NO TECHNICAL SPECIFICATIONS
NONE

Catalogue Number	Item Name	Available on Transversal Contract	Contract Number or Code
PHC-C-264	Varnish: cavity liner	RT296	RT296-02-199

Description	Cavity lining, dental: Vanish for cavity seal: 1 bottle, 15ml.
Use	For sealing of cavity in preparation for filling.
Applicable Standards	
Note to Procurement	

Rehabilitation

Batteries: hearing aid

NO TECHNICAL SPECIFICATIONS
NONE

Catalogue Number	Item Name	Available on Transversal Contract	Contract Number or Code
PHC-C-265	Batteries: hearing aid size 675	RT274-2018ME	26111701-00016
PHC-C-266	Batteries: hearing aid size 312	RT274-2018ME	26111701-00017
PHC-C-267	Batteries: hearing aid size 13	RT274-2018ME	26111701-00018
PHC-C-268	Batteries: hearing aid size 10	RT274-2018ME	26111701-00019

Description	Batteries for hearing aids (non-rechargeable).
Use	Replacement batteries for hearing aids.
Applicable Standards	
Note to Procurement	

Bicarbonate of soda: sachets

NO TECHNICAL SPECIFICATIONS NONE

Catalogue Number	Item Name	Available on Transversal Contract	Contract Number or Code
PHC-C-269	Bicarbonate of soda: sachets	No	

Description	Prepacked sachets of bicarbonate of soda.
Use	Treatment: cerumen management.
Applicable Standards	Bicarbonate of Soda BP.
Note to Procurement	

Breadboard: one-handed

NO TECHNICAL SPECIFICATIONS NONE

Catalogue Number	Item Name	Available on Transversal Contract	Contract Number or Code
PHC-C-270	Breadboard: one-handed	No	

Description	A wooden or Perspex chopping board with two nails protruding through the board at one corner, with a border edge of at least 2cm high on two sides of the same corner.
Use	This is for spearing a slice of bread to apply butter with one hand, or to chop vegetables and fruit using one hand.<br>NB: this could be manufactured at facility level. To rehabilitate and enable food preparation activities in hemiplegic patients.
Applicable Standards	
Note to Procurement	Metal needs to be a type which is largely rust resistant.

Cable ties: nylon,

NO TECHNICAL SPECIFICATIONS NONE

Catalogue Number	Item Name	Available on Transversal Contract	Contract Number or Code
PHC-C-271	Cable ties: nylon, medium	No	
PHC-C-272	Cable ties: nylon, long	No	

Description	Medium Self-locking cable ties, 200mm long by 5mm wide, minimum 22kg tensile strength.<br>Long Self-locking cable ties, 400mm long by 7,5mm wide, minimum 50kg tensile strength.<br>UV resistant. One-piece head with locking pawl.
Use	For fixing and manufacturing assistive devices.
Applicable Standards	
Note to Procurement	Equal quantities black and white when ordering.

Cardboard: A4, various colours

NO TECHNICAL SPECIFICATIONS NONE

Catalogue Number	Item Name	Available on Transversal Contract	Contract Number or Code
PHC-C-273	Cardboard,: A4, various colours	No	

Description	Various colours of A4 cardboard sheets in packs of 100.
Use	Manufacture communication boards and visual scheduling boards, as well as in fine motor activities for children with motor, sensory, cognitive or behavioural disorders.
Applicable Standards	
Note to Procurement	It is recommended that this be purchased with associated consumables such as crayons, pencils, scissors, laminating paper and glue, and with items such as a laminator and software such as Boardmaker.

Coloured pencils, pens, crayons

NO TECHNICAL SPECIFICATIONS
NONE

Catalogue Number	Item Name	Available on Transversal Contract	Contract Number or Code
PHC-C-274	Coloured pencils, pens, crayons	No	

Description	Assorted coloured writing instruments.
Use	To assess and treat children with literacy problems, emotional-behavioural problems, language and motor delays, etc.
Applicable Standards	
Note to Procurement	

Curette: cerumen, lighted

NO TECHNICAL SPECIFICATIONS NONE

Catalogue Number	Item Name	Available on Transversal Contract	Contract Number or Code
PHC-C-275	Curette: cerumen, lighted, replacement curettes	RT274-2014ME	RT274-12-017ME
PHC-C-276	Curette: cerumen, lighted, replacement light	RT274-2014ME	RT274-12-016ME

Description	Replacement curettes for illuminated cerumen curette.<br>Replacement light for illuminated cerumen curette.
Use	Spare curettes and light.
Applicable Standards	
Note to Procurement	In series with RT274-12-014ME.

Cushion: wheelchair

NO TECHNICAL SPECIFICATIONS NONE

Catalogue Number	Item Name	Available on Transversal Contract	Contract Number or Code
PHC-C-277	Wedge based, thin foam, positioner	RT233-2016	2.2.1.1 A4a
PHC-C-278	Wedge based, thick pressure care foam	RT233-2016	2.2.1.1 A2a

Description	2.2.1.1 A4a Wedge based, thin foam, positioner with contoured base, extended with no cover, in sizes 46cm, 41cm, 36cm and 30cm, all 50cm long, 4cm high at the rear, 9cm in front.<br>A minimum of 5 per size should be kept in stock at any given time, at the PHC team base. At the clinics themselves, where seating is offered, 2 per size at any given time would be adequate.<br>Each of the stock wheelchairs kept at the clinic should have a cushion and cover.<br>2.2.1.1 A2a. Wedge based, thick pressure care foam cushion with contoured base, extended with no cover, in sizes 46cm, 41cm, 36cm and 30cm, all 50cm long, 4cm high at the rear, 9cm in front.<br>A minimum of 5 per size should be kept in stock at any given time, at the PHC team base. At the clinics themselves, where seating is offered, 2 per size at any given time would be adequate.
Use	Seat cushions permit adjustments for postural asymmetries or lack of postural control, and help prevent pressure sores.
Applicable Standards	
Note to Procurement	

Cushion covers: wheelchair

NO TECHNICAL SPECIFICATIONS
NONE

Catalogue Number	Item Name	Available on Transversal Contract	Contract Number or Code
PHC-C-279	Cushion cover: waterproof, stretchable	RT233	42192208-00348
PHC-C-280	Cushion cover: basic, stretchable	RT233	42192208-00326

Description	Cushion cover, waterproof, stretchable. Stock should include a MINIMUM of 4 of each size, for at least 10”, 12” with 30cm seat depth, 12” with 40cm seat depth, 14’m, 16”, 18”. 20” could be kept as a minimum of 2 on hand at any point in time.<br>Cushion cover, basic, stretchable. Stock should include a MINIMUM of 4 of each size, for at least 10”, 12” with 30cm seat depth, 12” with 40cm seat depth, 14’m, 16”, 18”. 20” could be kept as a minimum of 2 on hand at any point in time.
Use	
Applicable Standards	
Note to Procurement	

Ear mould impression material and scoop

NO TECHNICAL SPECIFICATIONS NONE

Catalogue Number	Item Name	Available on Transversal Contract	Contract Number or Code
PHC-C-281	Ear mould impression material and scoop	RT274-2018ME	42143504-00004
PHC-C-282	Ear mould impression material catalyst	RT274-2018ME	42143504-00005

Description	Silicon based impression material (800g tub) with tube of catalyst, 40 shore.<br>Tube of catalyst for ear mould impression material, 20g tube.
Use	To make ear impressions for hearing aid fitment.
Applicable Standards	
Note to Procurement	Item RT274-10-005ME and 006ME must be awarded as a series.

Ear mould impression Otolight: replacement globe

NO TECHNICAL SPECIFICATIONS
NONE

Catalogue Number	Item Name	Available on Transversal Contract	Contract Number or Code
PHC-C-283	Otolight: replacement globe	RT274-2018	42143504-00011

Description	Lightbulb replacement for ear mould impressions Otolight.
Use	
Applicable Standards	
Note to Procurement	Must be compatible with RT274-11-004ME. Item RT274-11-007ME to 009 must be awarded as a series.

Ear mould impression Otolight: replacement tips

NO TECHNICAL SPECIFICATIONS NONE

Catalogue Number	Item Name	Available on Transversal Contract	Contract Number or Code
PHC-C-284	Otolight: replacement tips	RT274-2018	42143504-00012

Description	Replacement tapered tips of Otolight for ear mould impressions.<br>Hard transparent plastic tips for Otolight that is 0-1.5cm in diameter at the base with a narrow protrusion of 3-4cm in length and approximately 1mm in diameter with a rounded tip. Pack of 2.
Use	
Applicable Standards	
Note to Procurement	Must be compatible with RT274-11-004ME.

Exercise band: latex

NO TECHNICAL SPECIFICATIONS NONE

Catalogue Number	Item Name	Available on Transversal Contract	Contract Number or Code
PHC-C-288	Exercise band: latex, very light 1-1,5kg	No	
PHC-C-289	Exercise band: latex, light 1,5-2kg	No	
PHC-C-290	Exercise band: latex, medium 2-2,7kg	No	
PHC-C-291	Exercise band: latex, heavy 2,7-3,5kg	No	
PHC-C-292	Exercise band: latex, very heavy 3,5-4,5kg	No	

Description	Latex exercise band, elasticated, of varied resistances. Rolls of 46m.
Use	For resistance training in musculoskeletal conditions. Provides resistance throughout range of movement.
Applicable Standards	
Note to Procurement	

Feeding cup

NO TECHNICAL SPECIFICATIONS
NONE

Catalogue Number	Item Name	Available on Transversal Contract	Contract Number or Code
PHC-C-293	Cup: feeding	RT275-2016	85122108-00183

Description	Cup, feeding, specialized, transition, with spout without handles, autoclavable, non-spill valve, durable hard plastic, transparent, bpa free, size: paediatric .
Use	For addressing latching and feeding problems in neonates and infants, and dysphagia in adults.
Applicable Standards	
Note to Procurement	

Feeding spoon

NO TECHNICAL SPECIFICATIONS NONE

Catalogue Number	Item Name	Available on Transversal Contract	Contract Number or Code
PHC-C-294	Spoon: feeding	RT275-2016	85122108-00178

Description	Spoon, feeding, specialized, premature, paediatrics infants aged 4 months and older, shallow bowl of spoon, hard plastic, durable, auto-cleavable, size: 1,5cm bowl width of spoon.<br>The spoon head needs to be at an angle suitable for feeding for each individual client i.e. 0-30 degrees, handle padded with non-slip material with an adjustable circumference that allows for grading in the grip.
Use	For addressing feeding problems in patients with neurological impairments.
Applicable Standards	
Note to Procurement	

Ferrule: rubber

NO TECHNICAL SPECIFICATIONS
NONE

Catalogue Number	Item Name	Available on Transversal Contract	Contract Number or Code
PHC-C-295	Ferrule: rubber, 16mm	No	
PHC-C-296	Ferrule: rubber, 22mm	No	
PHC-C-297	Ferrule: rubber, 25mm	No	

Description	Rubber end stoppers for walking sticks, elbow crutches, axilla crutches, walking frames and quadropods.
Use	To prevent slips and falls in clients using walking sticks, quadropods, crutches or frames.
Applicable Standards	
Note to Procurement	

Foam: EVA

NO TECHNICAL SPECIFICATIONS NONE

Catalogue Number	Item Name	Available on Transversal Contract	Contract Number or Code
PHC-C-298	EVA foam	No	

Description	Very high density foam for padding areas of a device with exceptional wear and tear. Colour: black.
Use	For manufacturing postural support devices and other assistive devices.<br>Must be used with an appropriate cutting tool such as a Stanley knife or hacksaw blade.<br>Sizes 1mm, 2mm, 3mm, 6mm, 8mm, 10mm, 20mm, 30mm, 40mm, 50mm.<br>Sheet dimensions: 2400mm x 1200mm, Hard 2000 x 1000mm.
Applicable Standards	
Note to Procurement	Preferably an adhesive back to the sheet.

Foam: high-density, 36/20

NO TECHNICAL SPECIFICATIONS
NONE

Catalogue Number	Item Name	Available on Transversal Contract	Contract Number or Code
PHC-C-299	Foam: high-density, 36/20, 2,5cm	No	
PHC-C-300	Foam: high-density, 36/20, 5cm	No	

Description	High density foam 36/20, hardness factor 18-21.6, density range 32.1-40kg/m cubed, in sheets 5cm and 2.5cm thickness.
Use	For manufacturing postural support devices and other assistive devices. Therapists should have access to ongoing training in basic, intermediate and advanced seating to improve quality of seating and limit clinical adverse events/secondary complications. Tools such as the electric foam cutter and consumables such as cushion covers and contact adhesive glue should be available with this product.
Applicable Standards	
Note to Procurement	

Foam: HD compressed 1300

NO TECHNICAL SPECIFICATIONS
NONE

Catalogue Number	Item Name	Available on Transversal Contract	Contract Number or Code
PHC-C-301	Foam: HD compressed 1300, 2,5cm thick	No	
PHC-C-302	Foam: HD compressed 1300, 5cm thick	No	

Description	HD compressed (reconstituted, flexible PU) foam 1300, hardness factor 52-63, density 130+10% kg/m cubed (SANS 642-1976) in sheets 5cm and 2.5cm thickness.
Use	For manufacturing postural support devices and other assistive devices. Therapists should have access to ongoing training in basic, intermediate and advanced seating to improve quality of seating and limit clinical adverse events/secondary complications. Tools such as the electric foam cutter and consumables such as cushion covers and contact adhesive glue should be available with this product.
Applicable Standards	SANS 642-1976.
Note to Procurement	

Masonite

NO TECHNICAL SPECIFICATIONS
NONE

Catalogue Number	Item Name	Available on Transversal Contract	Contract Number or Code
PHC-C-303	Masonite board	No	

Description	Masonite board standard brown or white with smooth finish, semi moisture-resistant. Thickness 3mm, 3.2mm, 4.mm, 6.4mm. Sheet size 2440 x 1220mm.
Use	For manufacturing assistive devices.
Applicable Standards	
Note to Procurement	

Oil: arnica

NO TECHNICAL SPECIFICATIONS NONE

Catalogue Number	Item Name	Available on Transversal Contract	Contract Number or Code
PHC-C-304	Oil: arnica	No	

Description	Arnica oil in 1l containers.
Use	For massage when addressing soft tissue restrictions in musculoskeletal, orthopaedic and neuro cases.
Applicable Standards	
Note to Procurement	

Otostops

NO TECHNICAL SPECIFICATIONS
NONE

Catalogue Number	Item Name	Available on Transversal Contract	Contract Number or Code
PHC-C-285	Otostop 5mm	RT274-2018	42182005-00005
PHC-C-286	Otostop 10mm	RT274-2018	42182005-00006
PHC-C-287	Otostop 13mm	RT274-2018	42182005-00007

Description	
Use	
Applicable Standards	
Note to Procurement	

Paper: print-out, tympanometer

NO TECHNICAL SPECIFICATIONS NONE

Catalogue Number	Item Name	Available on Transversal Contract	Contract Number or Code
PHC-C-305	Paper: print-out, tympanometer	No	

Description	Paper for printing out tympanometry report.
Use	Produce hard-copy report of tympanogram.
Applicable Standards	
Note to Procurement	Must be compatible with internal printer attached to tympanometer.

Pelvic straps

NO TECHNICAL SPECIFICATIONS NONE

Catalogue Number	Item Name	Available on Transversal Contract	Contract Number or Code
PHC-C-339	Pelvic strap: basic 5cm	RT233	2.1.1
PHC-C-340	Pelvic strap: basic 3cm	RT233	2.1.2

Description	2.1.1 Pelvic straps, basic, adjustable, with D ring fitting, 5cm wide: a minimum of 10 in stock at PHC team base at any time.<br>2.1.2 Pelvic straps, basic, adjustable, with D ring fitting, 3cm wide: a minimum of 10 in stock at PHC team base at any point in time.
Use	Stabilise wheelchair user.
Applicable Standards	
Note to Procurement	

Pressure garment material: elastonet

NO TECHNICAL SPECIFICATIONS
NONE

Catalogue Number	Item Name	Available on Transversal Contract	Contract Number or Code
PHC-C-306	Pressure garment material: elastonet, beige	No	
PHC-C-307	Pressure garment material: elastonet, white		
PHC-C-308	Pressure garment material: elastonet, dark brown		

Description	85% polyester and 15% spandex material, 15m or 30m roll in beige, white and dark brown.
Use	Elasticated material used to manufacture pressure garments to improve scar healing and to prevent keloid formation.
Applicable Standards	
Note to Procurement	It is recommended that access to a sewing machine is guaranteed before purchasing this product.

Sewing kit

NO TECHNICAL SPECIFICATIONS NONE

Catalogue Number	Item Name	Available on Transversal Contract	Contract Number or Code
PHC-C-309	Sewing kit	No	

Description	High quality hand sewing kit including awl, thimble, unpicker, pins, needles of various sizes including high quality heavy duty leather needles and sewing thread including cotton and thread for sewing leather, webbing and neoprene.<br>Normal needles, Upholstery heavy long needles – straight 3”-12” long curved and 1.5” - 6” long, Tapestry – large eye, Leather, Darning blunt tip large eye, Beading, Embroidery, Sharp needle: size 5-10.<br>Sewing machine needle suitable to use on various materials used during rehabilitation.<br>Wedge point (leather, vinyl) – 14/90, 16/100.<br>Ball point (spandex single knit) –14/90, 10/70, 12/80.<br>Denim jeans – 16/100.<br>Regular point (towelling, flannel) – 16/100, 18/110, 14/90, 11/80, 9/70.<br>Sewing thread: Normal cotton: Rayon thread 40wt and 30wt, 100% spun Polyester thread , Cotton 100% cotton embroidery thread, size 30g, 50wt, Heavy duty nylon thread for upholstery, canvas and webbing – in variety of sizes 15, 33, 46.<br>v-30: 30 tex, x denier 300 x weight 33g.<br>v46: tex 45 x denier 460 x 22g.
Use	For manufacture of assistive devices.
Applicable Standards	
Note to Procurement	

Splinting material: thermoplastic sheets

NO TECHNICAL SPECIFICATIONS
NONE

Catalogue Number	Item Name	Available on Transversal Contract	Contract Number or Code
PHC-C-310	Thermoplastic sheet: 1,6mm	No	
PHC-C-311	Thermoplastic sheet: 2,0mm		
PHC-C-312	Thermoplastic sheet: 2,4mm		
PHC-C-313	Thermoplastic sheet: 2,5mm		
PHC-C-314	Thermoplastic sheet: 3,2mm		
PHC-C-315	Thermoplastic sheet: 4,8mm		

Description	Thermoplastic material for moulding splints for upper and lower limbs, in various thicknesses. Sheet size 45cm x 60cm.
Use	Manufacture of custom splints and supports.<br>Thermoplastic properties, mouldable after 35 seconds at 70-75 °C.<br>Working time for 1.6mm is 1-2min.<br>Working time for 2.4mm is 2-3 minutes.<br>Working time for 3.2mm is 4-6 minutes.<br>Working time 4.8mm is 7-8 minutes.<br>Translucent when heated, allowing identification of landmarks for positioning and pressure points for providing maximum comfort.<br>Stretches easily with excellent drapability, conforming well to contours.<br>100% memory allows repeated reheating and economical splint revisions.<br>Perforations provide superior ventilation assisting normal evaporation while maintaining material strength. Options for solid or perforated should be available for each thickness.
Applicable Standards	
Note to Procurement	It is recommended that this product be purchased with a splinting pan or electric frying pan, heat gun, Stanley knife, heavy duty splinting scissors, and Velcro.

Splinting material: thermoplastic tape

NO TECHNICAL SPECIFICATIONS NONE

Catalogue Number	Item Name	Available on Transversal Contract	Contract Number or Code
PHC-C-316	Thermoplastic tape: 3cm	No	
PHC-C-317	Thermoplastic tape: 6cm	No	

Description	Low temperature thermoplastic type material with a textile-like feel.<br>Rolls of 300cm length, in widths of 3cm or 6cm.<br>Density 20g/m.
Use	Activation temperature 65 °C, with a minimum activation time of 1 minute and a maximum activation time of 30 minutes.<br>Possible activation sources are: Suspan water bath, dry heater, heating plate, microwave oven and heat gun. The activation time depends on the heat source and the product size, and varies from 30 seconds to 5 minutes: 1. Water bath: 65°C: 1 min. 2. Dry heater or heating plate: 1-5 min 3. Microwave oven: 500W: 2-2,5 min 4. Heat gun: 1-4 seconds for reactivation of tape. The heat gun is not meant to be used as heat source to activate the total length of the tape before moulding it onto the patient.<br>Drying time 10 seconds.<br>Moulding time 1 minute.<br>Hardening time minimum 5 minutes.<br>Options for solid or perforated.<br>Excellent elasticity in two directions.<br>Adaptable when needed.<br>Thin and breathable fabric.<br>Easy to cut.<br>Straps, orthosis accessories or reinforcements can also be stuck to the orthosis using the self-adhesive properties of the tape: self-adhesive capacity, especially when dry heated.<br>Lightweight. Allows ventilation. Prevents maceration of the skin.<br>Tape roll allows to use circumferential wrapping and moulding technique.<br>Low to moderate memory. The material can be reactivated and unwrapped if not totally pressed together. This allows small corrections to the orthosis if necessary.
Applicable Standards	
Note to Procurement	

Towelling

NO TECHNICAL SPECIFICATIONS NONE

Catalogue Number	Item Name	Available on Transversal Contract	Contract Number or Code
PHC-C-318	Towelling	RT 60-1-2017	11161608-00002
PHC-C-319	Hand towels	RT26-2017	52121701-00002
PHC-C-320	Face cloth	RT26-2017	52121703-00001

Description	Towelling material of various thicknesses and sizes in a variety of colours.<br>69cm wide. Minimum mass 260g/m^2. Colour blue.<br>Hand towel 55cm x 110cm Royal blue.<br>Face cloth 25cm x25cm Royal blue.
Use	Manufacturing of assistive devices such as drooling cuffs.
Applicable Standards	SANS 1401: 2007 EDITION 2.1, PART 5, TYPE T36 OR SANS: 1613 TYPE DK270 OR DCR275.
Note to Procurement	If it is impossible to buy rolls of toweling material then purchasing bulk facecloths and hand towels will be sufficient.

Varnish: wood

NO TECHNICAL SPECIFICATIONS
NONE

Catalogue Number	Item Name	Available on Transversal Contract	Contract Number or Code
PHC-C-321	Varnish: wood	No	

Description	Wood varnish, clear, in 5l container. Oil-based, quick-drying.
Use	Manufacturing advanced paper technique posture chairs, trays, tables, etc., as well as treating other wood-made assistive devices.
Applicable Standards	
Note to Procurement	Paintbrush, 2.5, 5 and 10cm width, with appropriate solvent.

Velcro: circle, self-adhesive

NO TECHNICAL SPECIFICATIONS NONE

Catalogue Number	Item Name	Available on Transversal Contract	Contract Number or Code
PHC-C-322	Velcro: hook, circle, self-adhesive, 1cm diameter	RT275-2016	85122108-00010
PHC-C-323	Velcro: loop, circle, self-adhesive, 1cm diameter	RT275-2016	85122108-00011

Description	25m roll of white hook Velcro circles, 1cm diameter, with adhesive backing.<br>25m roll of white loop Velcro circles, 1cm diameter, with adhesive backing.
Use	Making picture-based alternative communication systems.
Applicable Standards	
Note to Procurement	

Velcro: loop, 5cm width, 50m roll

NO TECHNICAL SPECIFICATIONS NONE

Catalogue Number	Item Name	Available on Transversal Contract	Contract Number or Code
PHC-C-324	Velcro: loop, 5cm width, 50m roll	No	
PHC-C-325	Velcro: hook, 5cm width, 50m roll	No	

Description	50m roll of loop Velcro, 5cm width, in white/beige/black.<br>50m roll of hook Velcro, 5cm width, in white/beige/black.
Use	Making a variety of assistive devices and splints. Heavy duty scissors should be available, as should appropriate leather sewing needles and thread.
Applicable Standards	
Note to Procurement	Should come with options of adhesive backs or non-adhesive backs. Procure equal quantities of white, beige and black.

Washboard: one-handed

NO TECHNICAL SPECIFICATIONS NONE

Catalogue Number	Item Name	Available on Transversal Contract	Contract Number or Code
PHC-C-326	Washboard: one-handed	No	

Description	50cm x 30cm wooden frame, of which the two lengthwise borders extend an additional 20cm on one side. Corrugated metal sheet (corrugations 1cm breadth) set firmly within wooden frame.
Use	NB: this cannot be manufactured and must be purchased. To rehabilitate and enable clothes washing in hemiplegic patients.
Applicable Standards	
Note to Procurement	

Wheelchair spares kit

NO TECHNICAL SPECIFICATIONS
NONE

Catalogue Number	Item Name	Available on Transversal Contract	Contract Number or Code
PHC-C-327	Wheelchair spares kit	No	

Description	A kit of the most commonly replaced parts on wheelchairs:<br>Wheel bearings front and rear, front seat guides, castor bearings, brake blocks, assorted nuts and bolts.
Use	For on-site repairs to wheelchairs.
Applicable Standards	
Note to Procurement	There should be a standard kit. Used items can be replenished after an outreach trip.

Wheelchair gloves

NO TECHNICAL SPECIFICATIONS NONE

Catalogue Number	Item Name	Available on Transversal Contract	Contract Number or Code
PHC-C-337	Wheelchair gloves	No	

Description	Special gloves for wheelchair users.	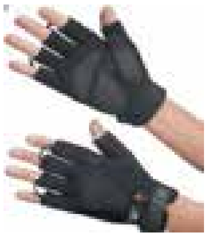
Use	Used to protect patients' hands against friction while propelling a wheelchair, as well as provide improved grip strength.	
Applicable Standards		
Note to Procurement	Available in leather or lycra, full-length and half-length. Will be ordered per patient.	

Wood

NO TECHNICAL SPECIFICATIONS
NONE

Catalogue Number	Item Name	Available on Transversal Contract	Contract Number or Code
PHC-C-328	Wood	No	

Description	A selection of solid wood short planks and dowel rods of various diameters.
Use	Used in the manufacture of assistive devices.
Applicable Standards	
Note to Procurement	

Sterilisation

Bowie-Dick test packs

NO TECHNICAL SPECIFICATIONS
NONE

Catalogue Number	Item Name	Available on Transversal Contract	Contract Number or Code
PHC-C-213	Bowie-Dick test packs	No	

Description	Standardised packs to test the efficacy of steam sterilisation in an autoclave.
Use	A test pack is placed on the lowest, coolest part of the autoclave chamber (over the drain grate). The pre-heated autoclave is run on the Bowie-Dick cycle. A colour change is expected throughout the thermosensitive pattern in the centre of the pack. Incomplete or no colour change, indicates failure of the test.
Applicable Standards	
Note to Procurement	

Helix test kit and strips

NO TECHNICAL SPECIFICATIONS
NONE

Catalogue Number	Item Name	Available on Transversal Contract	Contract Number or Code
PHC-C-214	Helix test kit and strips	No	

Description	The Helix test kit consists of a long, hollow, catheter-like vessel, into which a test strip is placed.
Use	The autoclave is then run on a Helix cycle (or a Bowie-dick cycle). This test assesses the steam's ability to penetrate deep hollow instruments. Lack of a colour change on the test strip means a failure of the test.
Applicable Standards	
Note to Procurement	

Personal protection equipment

NO TECHNICAL SPECIFICATIONS
NONE

Aprons: disposable

Catalogue Number	Item Name	Available on Transversal Contract	Contract Number or Code
PHC-C-137	Apron: disposable	No	

Description	Plastic or non-woven disposable apron.
Use	To prevent contamination of the healthcare worker's clothes or person.
Applicable Standards	Non-woven: CKS 676:2005.
Note to Procurement	

Face shield

NO TECHNICAL SPECIFICATIONS
NONE

Catalogue Number	Item Name	Available on Transversal Contract	Contract Number or Code
PHC-C-080	Face shield	No	

Description	Clear polycarbonate face shield.
Use	To protect healthcare worker's face and eyes from contamination by blood or other biological material.<br>Note the particular requirements for doffing this type of personal protection:<br>Doff after hand-cleansing post-procedure<br>Treat the front of the protection device as contaminated – handle only by the straps, elastic or ear loops<br>Dispose as potentially harmful medical waste.
Applicable Standards	None available.
Note to Procurement	

Gloves: sterile

NO TECHNICAL SPECIFICATIONS
NONE

Catalogue Number	Item Name	Available on Transversal Contract	Contract Number or Code
PHC-C-138	Gloves: sterile size 6 or 6,5 or small (box)	RT 76-2016	42132205-00013
PHC-C-139	Gloves: sterile size 7 or 7,5 or medium (box)	RT 76-2016	42132205-00014
PHC-C-140	Gloves: sterile size 8 or large (box)	RT 76-2016	42132205-00016

Description	Sterile latex gloves.
Use	For sterile procedures. Single use.
Applicable Standards	SANS 68:2003.<br>ISO 10282:2002.
Note to Procurement	Ensure that a percentage of each order is of type suitable for people with Type 1 latex allergy.

Gloves: non-sterile

NO TECHNICAL SPECIFICATIONS NONE

Catalogue Number	Item Name	Available on Transversal Contract	Contract Number or Code
PHC-C-141	Gloves: non-sterile, small (box)	RT 76-2016	42132203-00021
PHC-C-142	Gloves: non-sterile, Medium (box)	RT 76-2016	42132203-00022
PHC-C-143	Gloves, non-sterile, large (box)	RT 76-2016	42132203-00023

Description	Non-sterile latex gloves, single use.
Use	To prevent cross-infection between healthcare worker and patient, during non-sterile examinations.
Applicable Standards	SANS 1193-1:2010.
Note to Procurement	Ensure that a percentage of each order is of type suitable for people with Type 1 latex allergy.

Goggles

NO TECHNICAL SPECIFICATIONS NONE

Catalogue Number	Item Name	Available on Transversal Contract	Contract Number or Code
PHC-C-079	Goggles	No	

Description	Eye protection.
Use	Clear shatter-proof lenses, to protect healthcare worker's eyes from contamination by blood or other biological material. May be worn alone, or over spectacles. Adjustable temples. Re-usable.<br>Note the particular requirements for doffing this type of personal protection:<br>Doff after hand -cleansing post-procedure<br>Treat the front of the protection device as contaminated – handle only by the straps, elastic or ear loops<br>Dispose as potentially harmful medical waste.
Applicable Standards	
Note to Procurement	Non-fogging type preferred.

Gown: disposable

NO TECHNICAL SPECIFICATIONS: NONE

Catalogue Number	Item Name	Available on Transversal Contract	Contract Number or Code
PHC-C-076	Gown: long-sleeve, disposable	No	

Description	Sterile non-woven gown with long sleeves.
Use	To prevent cross-infection between healthcare worker and patient, during sterile procedures and non-sterile examinations.<br>Note the particular requirements for doffing this type of personal protection:<br>Doff after hand -cleansing post-procedure<br>Treat the front of the protection device as contaminated – handle only by the straps, elastic or ear loops<br>Dispose as potentially harmful medical waste.
Applicable Standards	CKS 676:2005.
Note to Procurement	

Particulate mask (N-95 respirator)

NO TECHNICAL SPECIFICATIONS NONE

Catalogue Number	Item Name	Available on Transversal Contract	Contract Number or Code
PHC-C-078	Particulate mask (N-95 respirator)	No	

Description	High-efficiency respirator. Covers lower part of face and nose. Elasticised strap for holding the mask in position, bendable aluminium nose strap to improve seal.<br>May have an exhalation valve for easier breathing.
Use	The respirator must be fitted to the individual healthcare worker, and tested for correct sealing. If incorrect, the respirator is ineffective.<br>Note the particular requirements for doffing this type of personal protection:<br>Doff after hand -cleansing post-procedure<br>Treat the front of the protection device as contaminated – handle only by the straps, elastic or ear loops<br>Dispose as potentially harmful medical waste.
Applicable Standards	SANS 50149:2003 ClassFFP2 or EN149:2001.<br>95% efficiency at filtering 0,3 µm particles of sodium chloride at 200mg loading.
Note to Procurement	The CSIR recommends a normal distribution across sizes, when bulk ordering. 20% Small, 60% medium, and 20% large, is recommended.

Surgical mask

NO TECHNICAL SPECIFICATIONS NONE

Catalogue Number	Item Name	Available on Transversal Contract	Contract Number or Code
PHC-C-077	Surgical mask	No	

Description	Plain half-face mask with ear loops.
Use	To prevent cross-infection between healthcare worker and patient, during sterile procedures and non-sterile examinations.<br>NB not adeqaute protection against air-borne pathogens.<br>Note the particular requirements for doffing this type of personal protection:<br>Doff after hand -cleansing post-procedure<br>Treat the front of the protection device as contaminated – handle only by the straps, elastic or ear loops<br>Dispose as potentially harmful medical waste.
Applicable Standards	SANS 1866:2008.
Note to Procurement	

consumables **general**

BUILDING MAINTENANCE CONSUMABLES 422

- Bolts and nuts: assorted 422
- Contact adhesive 423
- Glue: cyanoacrylate, rapid 424
- Glue: wood, waterproof 425
- Lubricating/releasing oil: spray can 426
- Nails: assorted 427
- Oil: 2-stroke for brush cutter 428
- Oil: 20W50, for petrol lawnmower 429
- Rivets: blind, aluminium, assorted 430
- Screws: chipboard, assorted 431
- Screws: roofing, with sealing washers 432
- Screws: self-tapping, assorted 433
- Sealant: silicone, marine, clear 434
- Tape: insulation 435
- Washers: locking, assorted 436
- Washers: plain, galvanised 437

CLEANING 438

- Aerosol insecticide 438
- Air freshener 439
- Bags: waste 440
- Chlorine compounds (bleach) 441
- Cloths: cleaning 442
- Detergent: liquid or powder 443
- Dishwashing liquid 444
- Drain cleaner 445
- Floor polymer remover 446
- Floor protective polymer 447
- Floor polisher replacement Cleaning disc 448
- Floor polisher replacement Scouring disc 449
- Hand rub: alcohol-based 450
- High level disinfectants 451
- Laundry detergent 452
- Microfibre cloth for static head mop 453
- Paper towel: folded 454
- Paper towel: roll 455
- Sanitary all-purpose cleaner 456
- Soap: antimicrobial 457
- Soap: handwashing, liquid non-antimicrobial 458
- Surgical scrub brush sponges 459
- Toilet paper 460
- Vacuum cleaner replacement filter set 461

GARDEN 462

GENERAL STATIONERY 463

- Files and accessories 463
- Order forms and books 464
- Writing materials 465

MISCELLANEOUS 466

- Disposable paper cups 466
- Identification tag 467
- Water packs for cold box 468

Building maintenance consumables

Bolts and nuts: assorted

NO TECHNICAL SPECIFICATIONS
NONE

Catalogue Number	Item Name	Available on Transversal Contract	Contract Number or Code
PHC-C-040	Bolts and nuts: assorted	No	

Description	Selection of bolts and nuts, from 4mm to 12mm, in lengths 10mm to 50mm, in standard threads. Includes Nylock self-locking nuts of the same sizes.
Use	To replace missing bolts or nuts, and to secure items to each other.
Applicable Standards	
Note to Procurement	

Contact adhesive

NO TECHNICAL SPECIFICATIONS
NONE

Catalogue Number	Item Name	Available on Transversal Contract	Contract Number or Code
PHC-C-217	Contact adhesive, 5l	No	

Description	All-purpose Polychloroprene rubber-based contact adhesive with high immediate bond strength, good brushability, water and heat resistance. Bonds instantly on contact to a variety of surfaces without clamping or sustained pressure. Excellent adhesion to Wood, processed boards (e.g. hardboard, supawood, chipboard, high pressure laminates, masonite, plywood), formica, veneers.
Use	For manufacturing cushions and postural support devices, as well as repairing boots, insoles, assistive devices in general.
Applicable Standards	
Note to Procurement	

Glue: cyanoacrylate, rapid

NO TECHNICAL SPECIFICATIONS
NONE

Catalogue Number	Item Name	Available on Transversal Contract	Contract Number or Code
PHC-C-041	Glue: cyanoacrylate, rapid	No	

Description	Fast-bonding superglue.
Use	For rapid temporary repair of small items of equipment.
Applicable Standards	
Note to Procurement	

Glue: wood, waterproof

NO TECHNICAL SPECIFICATIONS NONE

Catalogue Number	Item Name	Available on Transversal Contract	Contract Number or Code
PHC-C-042	Glue: wood, waterproof	No	

Description	Fast-setting, water-resistant wood glue for interior and exterior uses.
Use	For temporary repairs of damaged wooden equipment or structural items.
Applicable Standards	SANS 1348, Polyvinyl acetate dispersion adhesives for wood.<br>SANS 1349, Phenolic, amino-plastic and one-part polyurethane resin adhesives for the laminating and finger-jointing of timber, and for furniture and joinery.<br>SANS 10183:2009 D2.
Note to Procurement	

Lubricating/releasing oil: spray can

NO TECHNICAL SPECIFICATIONS NONE

Catalogue Number	Item Name	Available on Transversal Contract	Contract Number or Code
PHC-C-043	Lubricating/releasing oil: spray can	No	

Description	Rust-penetrating spray lubricant.
Use	To loosen rusted metal parts, general maintenance lubricant.
Applicable Standards	
Note to Procurement	

Nails: assorted

NO TECHNICAL SPECIFICATIONS NONE

Catalogue Number	Item Name	Available on Transversal Contract	Contract Number or Code
PHC-C-044	Nails: assorted	No	

Description	Selection of Wire nails, 1,8mm to 6mm, lengths 32mm to 75mm.
Use	Used for general repairs and maintenance.
Applicable Standards	
Note to Procurement	

Oil: 2-stroke for brush cutter

NO TECHNICAL SPECIFICATIONS
NONE

Catalogue Number	Item Name	Available on Transversal Contract	Contract Number or Code
PHC-C-045	Oil: 2-stroke for brush cutter	No	

Description	Oil for 2-stroke engines.
Use	Mixed with petrol according to manufacturer's specifications.
Applicable Standards	
Note to Procurement	

Oil: 20W50, for petrol lawnmower

NO TECHNICAL SPECIFICATIONS NONE

Catalogue Number	Item Name	Available on Transversal Contract	Contract Number or Code
PHC-C-046	Oil: 20W50, for petrol lawnmower	No	

Description	Oil for 4-stroke motor in petrol-driven lawn mower.
Use	Used to top up the machine's oil reservoir. Oil needs to be drained and replaced according to manufacturer's specifications.
Applicable Standards	
Note to Procurement	

Rivets: blind, aluminium, assorted

NO TECHNICAL SPECIFICATIONS NONE

Catalogue Number	Item Name	Available on Transversal Contract	Contract Number or Code
PHC-C-047	Rivets: blind, aluminium, assorted	No	

Description	Pop rivets, 4,8mm in lengths 8, 10, 16mm.
Use	Used to effect temporary or permanent repairs in sheet metal parts, in conjunction with a hand riveter.
Applicable Standards	
Note to Procurement	

Screws: chipboard, assorted

NO TECHNICAL SPECIFICATIONS
NONE

Catalogue Number	Item Name	Available on Transversal Contract	Contract Number or Code
PHC-C-048	Screws: chipboard, assorted	No	

Description	Coarse-threaded self-threading screws, for use in wood products.<br>4,5mm in various lengths: 20, 25, 40, 50mm.
Use	To re-attach fittings to wooden structures.
Applicable Standards	
Note to Procurement	

Screws: roofing, with sealing washers

NO TECHNICAL SPECIFICATIONS
NONE

Catalogue Number	Item Name	Available on Transversal Contract	Contract Number or Code
PHC-C-050	Screws: roofing, with sealing washers	No	

Description	TEK-type roofing screws with non-hardening sealing washers and metal protective washers. Standard thickness and length.
Use	To fasten roof sheeting and fascia boards, which have loosened. The screws are self-tapping, and can be driven with a spanner or screwdriver.
Applicable Standards	
Note to Procurement	

Screws: self-tapping, assorted

NO TECHNICAL SPECIFICATIONS NONE

Catalogue Number	Item Name	Available on Transversal Contract	Contract Number or Code
PHC-C-049	Screws: self-tapping, assorted	No	

Description	Selection of self-tapping screws, 3,5 to 6mm, lengths 12mm to 30mm.
Use	Used to re-attach fittings to metal structures, and for general sheet metal repairs.
Applicable Standards	
Note to Procurement	

Sealant: silicone, marine, clear

NO TECHNICAL SPECIFICATIONS NONE

Catalogue Number	Item Name	Available on Transversal Contract	Contract Number or Code
PHC-C-051	Sealant: silicone, marine, clear	No	

Description	Silicone sealant in medium-size squeeze tube.
Use	For effecting temporary sealing and repairs of leaks, around basins, sanitary fittings, windows, roof leaks.
Applicable Standards	SANS 1305: 2009.
Note to Procurement	

Tape: insulation

NO TECHNICAL SPECIFICATIONS
NONE

Catalogue Number	Item Name	Available on Transversal Contract	Contract Number or Code
PHC-C-052	Tape: insulation, black	No	
PHC-C-053	Tape: insulation, white	No	
PHC-C-054	Tape: insulation, red	No	

Description	Insulation tape in various colours, to match common wiring.
Use	For temporary repair of electric cords.
Applicable Standards	SANS 122: 2007.
Note to Procurement	

Washers: locking, assorted

NO TECHNICAL SPECIFICATIONS NONE

Catalogue Number	Item Name	Available on Transversal Contract	Contract Number or Code
PHC-C-056	Washers: locking, assorted	No	

Description	Selection of locking washers (spring or star type), 4mm to 12mm.
Use	Used to prevent loosening of nuts and bolts in metal components subject to movement or vibration.
Applicable Standards	
Note to Procurement	

Washers: plain, galvanised

NO TECHNICAL SPECIFICATIONS NONE

Catalogue Number	Item Name	Available on Transversal Contract	Contract Number or Code
PHC-C-057	Washers: plain, galvanised	No	

Description	Selection of steel flat washers, 6mm to 19mm diameter.
Use	Used to spread the load when using bolts and nuts, and to permit some movement between attached metal components.
Applicable Standards	
Note to Procurement	

Cleaning

NO TECHNICAL SPECIFICATIONS NONE

Aerosol insecticide

Catalogue Number	Item Name	Available on Transversal Contract	Contract Number or Code
PHC-C-071	Aerosol insecticide	No	

Description	Insect killer spray.
Use	Aerosol bio-degradable insecticide, effective against flying and crawling insect infestations. Toxic, store well away from food and medicines.
Applicable Standards	In accordance with A Cleanliness Guideline for Health Workers 2015.<br>Required as non-negotiable cleaning materials in A Cleanliness Guideline for Health Workers 2015, Annexure A.
Note to Procurement	SANS 899:2013 may be applicable, depending on the type of aerosol procured.

Air freshener

NO TECHNICAL SPECIFICATIONS
NONE

Catalogue Number	Item Name	Available on Transversal Contract	Contract Number or Code
PHC-C-069	Air freshener	No	

Description	Air freshening spray.
Use	Aerosol room air deodoriser.
Applicable Standards	In accordance with A Cleanliness Guideline for Health Workers 2015.<br>Required as non-negotiable cleaning materials in A Cleanliness Guideline for Health Workers 2015, Annexure A.
Note to Procurement	

Bags: waste

NO TECHNICAL SPECIFICATIONS NONE

Catalogue Number	Item Name	Available on Transversal Contract	Contract Number or Code
PHC-C-190	Bag: waste, black, large	No	
PHC-C-191	Bag: waste, 90l. Yellow	No	
PHC-C-192	Bag: waste, 90l. Green	No	
PHC-C-193	Bag: waste, 90l. Blue	No	
PHC-C-194	Bag: waste, 90l. White	No	
PHC-C-195	Bag: waste, 90l. Red heavy duty	No	
PHC-C-196	Bag: waste, 20l. Red heavy duty	No	

Description	Bags for bin lining and waste management.
Use	For use in accordance with A Cleanliness Guideline for Health Workers 2015.
Applicable Standards	Required as non-negotiable cleaning materials in A Cleanliness Guideline for Health Workers 2015, Annexure A.<br>Colour-coded bags are intended for specific types of waste, as per the guidelines.
Note to Procurement	

Chlorine compounds (bleach)

NO TECHNICAL SPECIFICATIONS NONE

Catalogue Number	Item Name	Available on Transversal Contract	Contract Number or Code
PHC-C-062	Chlorine compounds (bleach)	No	

Description	Chlorine containing compounds used for surface disinfection.
Use	
Applicable Standards	SANS 1032:2013, SANS 643:2013.<br>In accordance with A Cleanliness Guideline for Health Workers 2015.<br>Required as non-negotiable cleaning materials in A Cleanliness Guideline for Health Workers 2015, Annexure A.
Note to Procurement	

Cloths: cleaning

NO TECHNICAL SPECIFICATIONS NONE

Catalogue Number	Item Name	Available on Transversal Contract	Contract Number or Code
PHC-C-185	Cloth: cleaning, general purpose, 400x420	RT 14-2016	47131502-00000
PHC-C-186	Cloth: cleaning, 350X374. Yellow (50 pack)	RT 14-2016	47131502-00001
PHC-C-187	Cloth: cleaning, 350X374. Green (50 pack)	RT 14-2016	47131502-00003
PHC-C-188	Cloth: cleaning, 350X374. Blue (50 pack)	RT 14-2016	47131502-00004
PHC-C-189	Cloth: cleaning, 330X580. (50 pack)	RT 14-2016	47131502-00002

Description	Cloths for cleaning and dusting.
Use	For use In accordance with A Cleanliness Guideline for Health Workers 2015.
Applicable Standards	Required as non-negotiable cleaning materials in A Cleanliness Guideline for Health Workers 2015, Annexure A.<br>Colour-coded cloths are intended for use in specific locations, as per the guidelines.
Note to Procurement	

Detergent: liquid or powder

NO TECHNICAL SPECIFICATIONS NONE

Catalogue Number	Item Name	Available on Transversal Contract	Contract Number or Code
PHC-C-065	Detergent liquid or powder	No	

Description	Detergent for cleaning soiled or oily surfaces. Non-disinfectant.
Use	For medium to heavy soiling.
Applicable Standards	SANS1344:2017.<br>In accordance with A Cleanliness Guideline for Health Workers 2015.<br>Required as non-negotiable cleaning materials in A Cleanliness Guideline for Health Workers 2015, Annexure A.
Note to Procurement	

Dishwashing liquid

NO TECHNICAL SPECIFICATIONS
NONE

Catalogue Number	Item Name	Available on Transversal Contract	Contract Number or Code
PHC-C-064	Dishwashing liquid	No	

Description	Dishwashing and window-cleaning liquid.
Use	For use in accordance with A cleanliness Guide for Health Workers 2015.
Applicable Standards	SANS 825:2102.<br>In accordance with A Cleanliness Guideline for Health Workers 2015.<br>Required as non-negotiable cleaning materials in A Cleanliness Guideline for Health Workers 2015, Annexure A.
Note to Procurement	

Drain cleaner

NO TECHNICAL SPECIFICATIONS NONE

Catalogue Number	Item Name	Available on Transversal Contract	Contract Number or Code
PHC-C-068	Drain cleaner	No	

Description	Liquid, gel or granules, to dissolve blockages in drains.
Use	Usually strongly alkaline compounds, which saponify fat, making it water-soluble. Toxic and potentially harmful to the user. Use strictly according to the manufacturer's instructions. Store well away from food and medicines.
Applicable Standards	In accordance with A Cleanliness Guideline for Health Workers 2015.<br>Required as non-negotiable cleaning materials in A Cleanliness Guideline for Health Workers 2015, Annexure A.
Note to Procurement	

Floor polymer remover

NO TECHNICAL SPECIFICATIONS
NONE

Catalogue Number	Item Name	Available on Transversal Contract	Contract Number or Code
PHC-C-067	Floor polymer remover (stripper)	No	

Description	Ammoniated or non-ammoniated compounds, for removal of floor polish.
Use	For removal of weathered floor finish, prior to re-application.
Applicable Standards	SANS1224:2017.<br>In accordance with A Cleanliness Guideline for Health Workers 2015.<br>Required as non-negotiable cleaning materials in A Cleanliness Guideline for Health Workers 2015, Annexure A.
Note to Procurement	

Floor protective polymer

NO TECHNICAL SPECIFICATIONS NONE

Catalogue Number	Item Name	Available on Transversal Contract	Contract Number or Code
PHC-C-066	Floor protective polymer	No	

Description	Polymer that provides a hard or soft protective coating on vinyl or other flooring.
Use	To create a protective shiny layer on vinyl or other flooring. When the surface dulls, it must be stripped and the polymer re-applied.
Applicable Standards	SANS1042:2012.<br>In accordance with A Cleanliness Guideline for Health Workers 2015.<br>Required as non-negotiable cleaning materials in A Cleanliness Guideline for Health Workers 2015, Annexure A.
Note to Procurement	

Floor polisher replacement Cleaning disc

NO TECHNICAL SPECIFICATIONS
NONE

Catalogue Number	Item Name	Available on Transversal Contract	Contract Number or Code
PHC-C-208	General purpose cleaning disc	No	

Description	General-purpose cleaning disc for electric single-disc floor polisher.
Use	For general cleaning and polishing with the machine.
Applicable Standards	None available.
Note to Procurement	Disc must be in series with PHC-F-020.

Floor polisher replacement Scouring disc

NO TECHNICAL SPECIFICATIONS
NONE

Catalogue Number	Item Name	Available on Transversal Contract	Contract Number or Code
PHC-C-209	Scouring disc	No	

Description	Abrasive disc for electric single-disc floor polisher.
Use	The abrasive disc is used on the floor polisher to remove (strip) old polymer finish, prior to re-application of the protective finish.
Applicable Standards	None available.
Note to Procurement	Disc must be in series with PHC-F-020.

Hand rub: alcohol-based

NO TECHNICAL SPECIFICATIONS NONE

Catalogue Number	Item Name	Available on Transversal Contract	Contract Number or Code
PHC-C-060	Hand rub: alcohol-based, with emollient	No	

Description	Gel-type compound used for hand disinfection.
Use	Not as effective as scrubbing. Can be used for disinfection between patient contacts, if soap and water are unavailable.
Applicable Standards	SANS 490:2013.<br>In accordance with A Cleanliness Guideline for Health Workers 2015.<br>Required as non-negotiable cleaning materials in A Cleanliness Guideline for Health Workers 2015, Annexure A.
Note to Procurement	

High level disinfectants

NO TECHNICAL SPECIFICATIONS
NONE

Catalogue Number	Item Name	Available on Transversal Contract	Contract Number or Code
PHC-C-063	Glutaraldehydes	No	
PHC-C-384	Sodium perborate powder	No	
PHC-C-385	Phthalaldehydes	No	

Description	Certain equipment is not suitable for sterilisation by autoclaving. For such equipment High-level Disinfection is applicable. The commonest disinfectant is glutaraldehyde, but because of significant health risks to users, and resistance in certain mycobacteria, Ortho-phthalaldehyde is a viable replacement. However, Ortho-phthalladehyde is not sporicidal.<br>Sodium perborate (in combination with other chemicals) forms hydrogen peroxide in solution.
Use	Used for de-contamination of surfaces or instruments.
Applicable Standards	SANS 1593:2015, SANS 1615:2011.<br>In accordance with A Cleanliness Guideline for Health Workers 2015.<br>Required as non-negotiable cleaning materials in A Cleanliness Guideline for Health Workers 2015, Annexure A.
Note to Procurement	

Laundry detergent

NO TECHNICAL SPECIFICATIONS NONE

Catalogue Number	Item Name	Available on Transversal Contract	Contract Number or Code
PHC-C-070	Laundry detergent	No	

Description	Low-foam detergent for use in automatic washing machines.
Use	Non-disinfectant laundry detergent.
Applicable Standards	SANS651:2012.<br>In accordance with A Cleanliness Guideline for Health Workers 2015.<br>Required as non-negotiable cleaning materials in A Cleanliness Guideline for Health Workers 2015, Annexure A.
Note to Procurement	

Microfibre cloth for static head mop

NO TECHNICAL SPECIFICATIONS
NONE

Catalogue Number	Item Name	Available on Transversal Contract	Contract Number or Code
PHC-C-210	Microfibre cloth for static head mop	No	

Description	A ready-made microfibre cloth sleeve, to fit over the frame of the static head mop.<br>Washable and re-usable.
Use	Used for dry-mopping of floors and other large surfaces.
Applicable Standards	
Note to Procurement	This item to be in series with the mop PHC-E-018

Paper towel: folded

NO TECHNICAL SPECIFICATIONS: NONE

Catalogue Number	Item Name	Available on Transversal Contract	Contract Number or Code
PHC-C-072	Paper towel: folded	RT 14	14111703-0003

Description	Absorbent paper towels, individually folded. May be interleaved. Usually supplied in a pack, with a disposable wrapper.	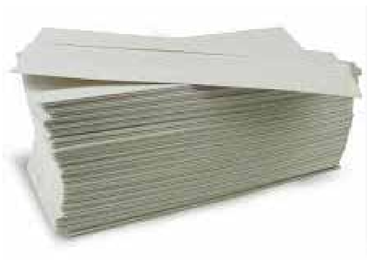
Use	Used in a paper towel dispenser, wall-mounted close to a hand washbasin.	
Applicable Standards	SANS 1887-4: 2015.	
Note to Procurement	Size must be compatible with dispenser PHC-E-020.	

Paper towel: roll

NO TECHNICAL SPECIFICATIONS NONE

Catalogue Number	Item Name	Available on Transversal Contract	Contract Number or Code
PHC-C-073	Paper towel: roll	No	

Description	Absorbent paper towel in roll form, with cardboard tube core. Widely used for domestic purposes, and therefore readily available. Standard and large size (but the width of the roll is unchanged).	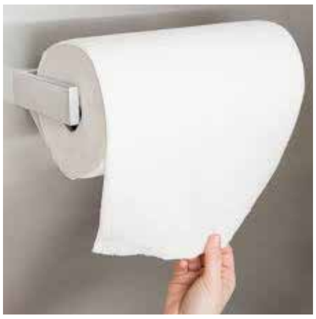
Use	Used in a paper towel roll holder, wall-mounted in the vicinity of hand washbasins or sinks.	
Applicable Standards	SANS 1887-5: 2015.	
Note to Procurement	Size must be compatible with PHC-E-021.	

Sanitary all-purpose cleaner

NO TECHNICAL SPECIFICATIONS
NONE

Catalogue Number	Item Name	Available on Transversal Contract	Contract Number or Code
PHC-C-136	Sanitary all-purpose cleaner	No	

Description	Ammonia-containing liquid or gel cleaner.
Use	All-purpose cleaner and sanitiser.
Applicable Standards	SANS 693:2013, SANS1225:1985.<br>In accordance with A Cleanliness Guideline for Health Workers 2015.<br>Required as non-negotiable cleaning materials in A Cleanliness Guideline for Health Workers 2015, Annexure A.
Note to Procurement	

Soap: antimicrobial

NO TECHNICAL SPECIFICATIONS
NONE

Catalogue Number	Item Name	Available on Transversal Contract	Contract Number or Code
PHC-C-059	Soap: antimicrobial	No	

Description	Liquid antimicrobial soap, purchased in bulk.
Use	To achieve surgical cleanliness, when preparing for a procedure. Used in dedicated soap dispensers.
Applicable Standards	SANS 288:2013.<br>In accordance with A Cleanliness Guideline for Health Workers 2015.<br>Required as non-negotiable cleaning materials in A Cleanliness Guideline for Health Workers 2015, Annexure A.
Note to Procurement	

Soap: handwashing, liquid non-antimicrobial

NO TECHNICAL SPECIFICATIONS
NONE

Catalogue Number	Item Name	Available on Transversal Contract	Contract Number or Code
PHC-C-058	Soap: handwashing, liquid non-antimicrobial	No	

Description	Liquid handwashing soap, purchased in bulk.
Use	Used to refill soap dispensers near hand washbasins.
Applicable Standards	SANS 1924:2007.
Note to Procurement	

Surgical scrub brush sponges

NO TECHNICAL SPECIFICATIONS NONE

Catalogue Number	Item Name	Available on Transversal Contract	Contract Number or Code
PHC-C-061	Surgical scrub brush sponges	No	

Description	Synthetic sponge with layer of semi-soft bristles.
Use	Used to scrub hands and arms in preparation for a procedure. Bristles assist in cleaning under nails.
Applicable Standards	None available.<br>In accordance with A Cleanliness Guideline for Health Workers 2015.<br>Required as non-negotiable cleaning materials in A Cleanliness Guideline for Health Workers 2015, Annexure A.
Note to Procurement	

Toilet paper

NO TECHNICAL SPECIFICATIONS NONE

Catalogue Number	Item Name	Available on Transversal Contract	Contract Number or Code
PHC-C-081	Toilet paper	RT 14	14111704-0000

Description	Toilet paper on roll.
Use	Single-ply, perforated. 600 sheets per roll.
Applicable Standards	SANS 1887-2: 2015 (Ed. 1.03).
Note to Procurement	

Vacuum cleaner replacement filter set

NO TECHNICAL SPECIFICATIONS NONE

Catalogue Number	Item Name	Available on Transversal Contract	Contract Number or Code
PHC-C-216	Replacement filter set: vacuum cleaner	No	

Description	Set of filters for canister-type vacuum cleaner.
Use	Vacuum cleaner filters are cleanable, washable and re-usable, but must eventually be replaced. Replace inlet and outlet filters at the same time.
Applicable Standards	None.
Note to Procurement	Filters must be in series with PHC-F-046.

Garden

NO TECHNICAL SPECIFICATIONS
NONE

Catalogue Number	Item Name	Available on Transversal Contract	Contract Number or Code
PHC-C-082	Bag, grass	No	
PHC-C-083	Magnesium sulphate	No	
PHC-C-084	Lime-Ammonia-Nitrogen (LAN)	No	
PHC-C-085	Weed killer	No	

Description	Consumables for garden maintenance.
Use	Used by grounds maintenance staff.<br>A selection of fertilisers and a general purpose weed-killer (to exterminate grass growing in paving).
Applicable Standards	
Note to Procurement	

General stationery

Files and accessories

NO TECHNICAL SPECIFICATIONS
NONE

Catalogue Number	Item Name	Available on Transversal Contract	Contract Number or Code
PHC-C-002	File: top access, A4	No	
PHC-C-003	File: side access, A4	No	
PHC-C-004	File label: self-adhesive	No	
PHC-C-005	File-out card	No	
PHC-C-006	File baskets/containers	No	

Description	Files, labels and containers, to store loose A4 sheets and documents.	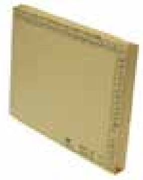 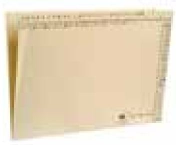 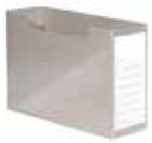
Use	Top access files are used in 4-drawer filing cabinets and similar systems.<br>The files are stored upright in baskets or containers within each drawer.<br>A self-adhesive label with the file number is applied along the top edge. The top edge has a printed scale, to permit accurate positioning of the label. Accurate positioning is vital for efficient filing and retrieval.<br>Side access files are stored on shelves in bulk-filing systems. The right short edge has a printed scale for positioning of the self-adhesive label.<br>A file-out card is inserted as a place-keeper when a file is retrieved, to ensure efficient and accurate re-filing.<br>Heavy-duty A4 files, housing up to 300 A4 sheets.<br>Top-access files have a printed scale along the top long edge.<br>Side-access files have a printed scale along the right short edge.<br>Self-adhesive labels have a transparent protective covering over the writing surface when correctly applied.	
Applicable Standards	Lock away at end of working day, in compliance with POPI Act.	
Note to Procurement		

Order forms and books

NO TECHNICAL SPECIFICATIONS NONE

Catalogue Number	Item Name	Available on Transversal Contract	Contract Number or Code
PHC-C-014	Order Form: goods and supplies	No	
PHC-C-015	Order book: goods and supplies	No	

Description	Order Form or book.
Use	Used to order goods and supplies as per departmental prescripts.
Applicable Standards	
Note to Procurement	

Writing materials

NO TECHNICAL SPECIFICATIONS NONE

Catalogue Number	Item Name	Available on Transversal Contract	Contract Number or Code
PHC-C-007	Marker: permanent, fine-point, black	No	
PHC-C-008	Marker: whiteboard (erasable), medium point, black	No	
PHC-C-009	Marker: whiteboard (erasable), medium point, red	No	
PHC-C-010	Pen: ballpoint, non-retractable, black	No	
PHC-C-011	Pen: ballpoint, non-retractable, red	No	
PHC-C-012	Pencil: HB	No	
PHC-C-013	Eraser: rubber	No	

Description	Writing instruments.
Use	
Applicable Standards	Ballpoint pens: SANS 785:2006.<br>Pencils (wood-cased): SANS 1531:2003.<br>Erasers: SANS 1581:2012.
Note to Procurement	

Miscellaneous

Disposable paper cups

NO TECHNICAL SPECIFICATIONS
NONE

Catalogue Number	Item Name	Available on Transversal Contract	Contract Number or Code
PHC-C-164	Disposable paper cups	No	

Description	Disposable paper cups, for use with watercooler.
Use	No polystyrene.
Applicable Standards	
Note to Procurement	

Identification tag

NO TECHNICAL SPECIFICATIONS
NONE

Catalogue Number	Item Name	Available on Transversal Contract	Contract Number or Code
PHC-C-202	Identification tag, official	No	

Description	Official personnel identification tag – metal or laminated plastic.
Use	For displaying the healthcare worker's name, to permit correct identification by members of the public.<br>Contains the emblem of the facility/District or Provincial Department of Health, and the full names / initials and surname of the staff member.<br>A photo of the staff member is optional.
Applicable Standards	All personal identification items must be safeguarded.
Note to Procurement	

Water packs for cold box

NO TECHNICAL SPECIFICATIONS — NONE

Catalogue Number	Item Name	Available on Transversal Contract	Contract Number or Code
PHC-C-203	Water packs for cooler box	No	

Description	Re-usable, water-fillable containers.
Use	Can be frozen or cooled, according to need. Placed in the vaccine cooler box in the numbers specified by the supplier, to achieve the specified temperature and cold-life. Do not replace with pre-filled type, as these may freeze below 0°C, and inactivate the vaccine.
Applicable Standards	Must comply with, and be pre-qualified on WHO PQS E005: Coolant packs for insulated containers. Proof to be supplied.
Note to Procurement	Each cooler box should be supplied with two sets of packs, in the quantity specified by the manufacturer. Do not purchase pre-filled cooler packs – they may freeze at too low a temperature, and inactivate the vaccine.

D
furniture & **appliances**

CLINICAL 470

Bassinet	470
Bed: hospital, 2-section	471
Bed: hospital, mattress	472
Bed: hospital, mattress cover	473
Bed: hospital, obstetric	474
Bucket for kick-about	475
Cabinet: medicine, mobile	476
Cabinet: scheduled drugs, wall-mounted	477
Couch: treatment, hydraulic Bobath (neuro)	478
Drip stand	479
Examination couch	480
Examination couch: portable	481
Examination couch: rehab	482
Foot stool	483
Kick-about	484
Locker: bedside, steel	485
Refrigerator: vaccine, chest	486
Refrigerator: vaccine, upright model	487
Screen: bedside, wheeled, complete	488
Screen: bedside, replacement curtains	489
Stool: anaethetist	490
Stool: dental assistant	491
Stool: dentist	492
Stool: height-adjustable, with backrest	493
Table: over- bed	494
Trolley: anaesthetic	495
Trolley: emergency	496
Trolley: gas cylinder	497
Trolley: medical dressing, large	498
Trolley: medical instruments, medium	499
Trolley: medical dressing, small	500
Trolley: medical dressing with bucket and bowl attachment	501
Trolley: patient, with mattress	502
Trolley: soiled instruments and linen	503
Trolley: soiled linen, complete	504
Viewing box: X-ray	505
Wheelchair: porter	506

GENERAL 507

Chair: office	507
Chair: operator	508
Chair: stackable, metal frame	509
Chair: stackable, netted mid-back	510
Chair: visitor	511
Credenza	512
Cupboard: sterile stock	513
Desk: 3-drawer, L-shaped	514
Desk: rectangular	515
Filling Cabinet: bulk	516
Filing cabinet: four-drawer	518
Floor polisher: electric, single disc	519
Freezer: chest, human tissue	520
Microwave oven: 25l, 900W manual	521
Mirror	522
Refrigerator: bar type	523
Refrigerator: double door	524
Refrigerator: single door	525
Seating: 2-seater	526
Seating: 3-seater	527
Seating: 4-seater	528
Steel cabinet: lockable, 6 shelves	529
Storage rack: exercise ball, wall-mounted	530
Table: folding	531
Table: trapezoidal	532
Tumble dryer	533
Vacuum cleaner	534
Washing machine: front loader	535
Washing machine: top loader	536

Clinical

Bassinet

TECHNICAL SPECIFICATIONS page 724

Catalogue Number	Item Name	Available on Transversal Contract	Contract Number or Code
PHC-F-025	Bassinet, complete	RT 24	RT 24-02-018

Description	Bassinet, complete (frame, bassinet and mattress).	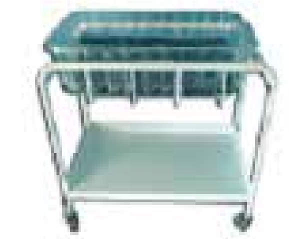
Use	For safe examination of an infant.	
Clinical or User Specifications	Steel frame, Transparent plastic bassinet. Complete with mattress.<br>Castor-mounted.<br>Spare mattress available as item RT 24-02-021. Thickness approximately 50mm. Mattress cover is waterproof and air-permeable.	
Accessories or Related Consumables	Bassinet, plastic, spare PHC-F-026.<br>Mattress for bassinet, spare PHC-F-027.	
Care	Standard cleaning and disinfecting.	
Supplier Warranty	2 years (excluding deliberate or accidental damage).	

Bed: hospital, 2-section

TECHNICAL SPECIFICATIONS page 725

Catalogue Number	Item Name	Available on Transversal Contract	Contract Number or Code
PHC-F-072	Bed: hospital, 2-section	RT24-2016	RT24-02-001

Description	Basic hospital bed	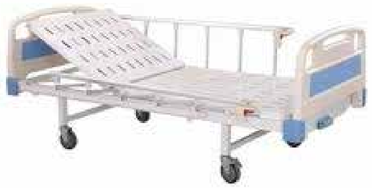
Use	For use in short-stay areas.	
Clinical or User Specifications	Mild steel frame with epoxy/nylon powder-coated finish.<br>Epoxy/nylon powder coating colours: white, cream or grey.<br>Bed must support a patient mass of 180 kg Adjustable backrest with gas spring assist, suitable for 100 kg patient.<br>To be fitted with castors, two swivel, two locking<br>Removable head and foot ends (ABS material is acceptable)<br>With collapsible safety sides.<br>Mattress support other than weldmesh is required.	
Accessories or Related Consumables	Complete with folding cot sides. Drip pole and patient lifting pole and strap optional	
Care	Standard cleaning and disinfection	
Supplier Warranty	2 years	

Bed: hospital, mattress

TECHNICAL SPECIFICATIONS page 726

Catalogue Number	Item Name	Available on Transversal Contract	Contract Number or Code
PHC-F-073	Bed: hospital, mattress	RT24-2016	RT24-02-014

Description	Bed mattress
Use	Can be used independently of bed type. Purchased separately from the bed chassis.
Clinical or User Specifications	Mattress for hospital bed/cot.<br>The mattress with cover must be flexible to accommodate anatomical contouring of all the angles of the bed.<br>Mattress must support a patient of at least 180 kg and return to original shape when not in use.<br>Mattress must be radio-translucent. To be constructed of flexible polyurethane foam ("memory foam").<br>Overall dimensions: Length: 1 980 mm (-0 and +40mm) Width: 860 mm (-0 and +20mm) Thickness: 150 mm (-0 and +5mm).<br>Must include removable cover (also available as replacement item PHC-F-074).
Accessories or Related Consumables	
Care	Standard cleaning and disinfection.
Supplier Warranty	2 years.

Bed: hospital, mattress cover

TECHNICAL SPECIFICATIONS page 727

Catalogue Number	Item Name	Available on Transversal Contract	Contract Number or Code
PHC-F-074	Bed: hospital, mattress cover	RT24-2016	RT24-02-015

Description	Mattress cover.
Use	Removable, replaceable mattress cover.
Clinical or User Specifications	Cover for mattress (for hospital bed/cot).<br>Cover must be removable, water-resistant, air permeable material and radio-translucent.<br>Where a zip is incorporated, it shall be a heavy duty, large- toothed synthetic zip to allow removal of the inner mattress, totally covered by a flap extending over the zip to prevent ingress of fluids.
Accessories or Related Consumables	
Care	Standard cleaning and disinfection.
Supplier Warranty	2 years.

Bed: hospital, obstetric

TECHNICAL SPECIFICATIONS page 728

Catalogue Number	Item Name	Available on Transversal Contract	Contract Number or Code
PHC-F-029	Bed: hospital, obstetric	RT 24	RT 24-02-009

Description	Delivery bed.	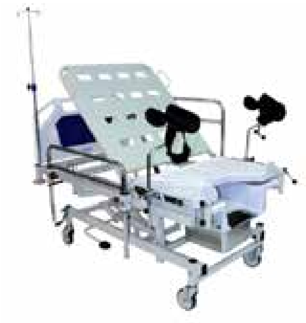
Use	To be used for resuscitations, as well as occasional emergency deliveries in PHC facilities without a MOU. Also used as a regular delivery bed in MOUs.	
Clinical or User Specifications	Epoxy-coated steel frame, 2 sections Complete with mattress.<br>Removable foot section, adjustable backrest, hydraulic adjustment of bed height and Trendelenburg position.	
Accessories or Related Consumables	Two lithotomy poles, height adjustable, swivel action, with leg support (not straps).<br>Douche fitting.<br>Douche tray, stainless steel.<br>Drip rod.	
Care	Standard cleaning and disinfecting.	
Supplier Warranty	5 years (excluding deliberate or accidental damage).	

Bucket for kick-about

TECHNICAL SPECIFICATIONS page 730

Catalogue Number	Item Name	Available on Transversal Contract	Contract Number or Code
PHC-F-033	Bucket for kick-about	RT 24	RT 24-10-004

Description	Bucket for kick-about.	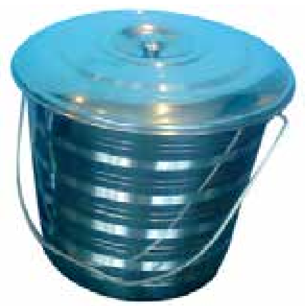
Use	For convenient temporary disposal of medical waste, during procedures.	
Clinical or User Specifications	Stainless steel bucket, to be ordered together with Kick-about RT 24-10-003.	
Accessories or Related Consumables		
Care	Standard cleaning and disinfecting.	
Supplier Warranty	2 years (excluding deliberate or accidental damage).	

Cabinet: medicine, mobile

TECHNICAL SPECIFICATIONS page 731

Catalogue Number	Item Name	Available on Transversal Contract	Contract Number or Code
PHC-F-069	Cabinet: medicine, mobile	RT24-2016	RT24-04-003

Description	Mobile medicine trolley, in ABS plastic.	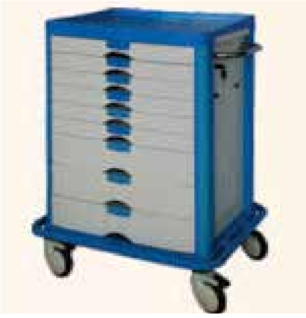
Use	In a ward environment, used to keep and dispense prescribed medication.	
Clinical or User Specifications	Top surface to be flat, suitable for writing and dispensing.<br>Multiple drawers with dividers (4- 6 drawers) Two large storage drawers, no dividers.<br>Drawers must have smooth, durable sliding mechanism with stop to prevent total extraction of drawer.<br>Approximate dimensions: Height: 950 mm Width: 750 mm Depth: 500 mm.<br>Mounted on 4 heavy duty swivel castors 125 mm diameter, the two front castors should be fitted with foot brakes with toe release mechanism.<br>Materials which are easily cleaned and of lasting finish, and low noise generating nature, shall be used.	
Accessories or Related Consumables		
Care	Standard cleaning and disinfection.	
Supplier Warranty	2 years.	

Cabinet: scheduled drugs, wall-mounted

TECHNICAL SPECIFICATIONS page 732

Catalogue Number	Item Name	Available on Transversal Contract	Contract Number or Code
PHC-F-070	Cabinet: scheduled drugs, wall-mounted	RT24-2016	RT24-04-005

Description	Wall-mounted lockable cabinet.	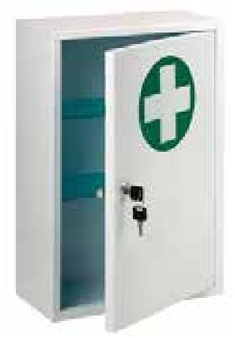
Use	For secure storage of scheduled drugs.	
Clinical or User Specifications	Mild steel with epoxy/nylon powder-coated finish.<br>At least one internal shelf.<br>NOTE: The cabinet must not be marked "Poison".	
Accessories or Related Consumables	All wall-mounting hardware. 2 keys.	
Care	Standard cleaning and disinfection.	
Supplier Warranty	2 years.	

Couch: treatment, hydraulic Bobath (neuro)

TECHNICAL SPECIFICATIONS page 733

Catalogue Number	Item Name	Available on Transversal Contract	Contract Number or Code
PHC-F-060	Treatment couch: hydraulic Bobath (neuro)	No	

Description	Large padded treatment couch/plinth, height adjustable (hydraulic) and modular: for treating patients with neurological impairments.	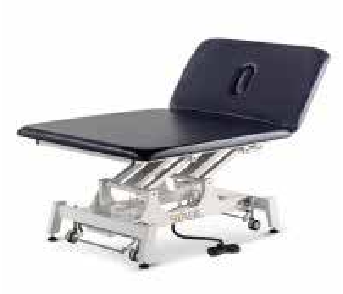
Use	For rehabilitating neuro clients and those with severe mobility impairments. It is critical that it is height adjustable from a patient safety as well as rehabilitation (motor learning) perspective.	
Clinical or User Specifications	Hydraulic is preferable to electric in areas where power supply is erratic. Plinth must be covered with durable vinyl which is easily cleanable.<br>120-125cm wide, 195cm long, height range 46cm-91cm, backrest angle 80-85 degrees.	
Accessories or Related Consumables	None.	
Care	Do not exceed maximum carrying capacity. Standard cleaning and disinfection.	
Supplier Warranty	3 years (excluding deliberate or accidental damage).	

Drip stand

TECHNICAL SPECIFICATIONS page 734

Catalogue Number	Item Name	Available on Transversal Contract	Contract Number or Code
PHC-F-028	Drip stand: mobile	RT 24	RT 24-10-001

Description	Drip stand, mobile.	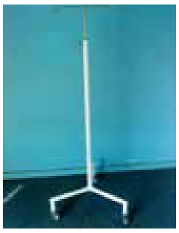
Use	For hanging containers of intravenous fluid, during infusion.	
Clinical or User Specifications	Castor-mounted mobile stand for IV bags.	
Accessories or Related Consumables		
Care	Standard cleaning and disinfecting.	
Supplier Warranty	2 years (excluding deliberate or accidental damage).	

Examination couch

TECHNICAL SPECIFICATIONS page 735

Catalogue Number	Item Name	Available on Transversal Contract	Contract Number or Code
PHC-F-030	Examination couch	RT 24	RT 24-02-024

Description	Examination couch.	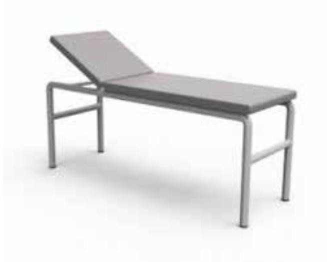
Use	To be used for physical examination of patients.	
Clinical or User Specifications	Two-piece design. Head part can be raised up to 60 degrees above horzontal.<br>Padded upper surface, covered in waterproof, dark-coloured fabric.<br>Epoxy powder-coated Mild steel frame. Rubber ferrules on feet.	
Accessories or Related Consumables		
Care	Standard cleaning and disinfecting.	
Supplier Warranty	5 years (excluding deliberate or accidental damage).	

Examination couch: portable

TECHNICAL SPECIFICATIONS page 736

Catalogue Number	Item Name	Available on Transversal Contract	Contract Number or Code
PHC-F-061	Examination couch: portable	No	

Description	Folding examination couch, modular, height adjustable, with removable head and arm rests in a canvas bag.	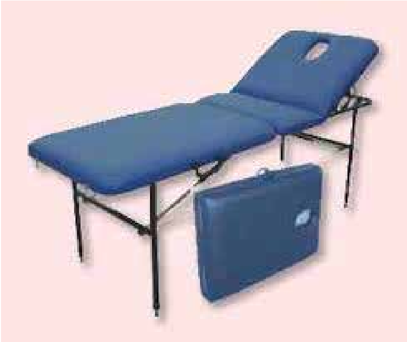
Use	Used extensively in rehabilitating acute musculoskeletal and orthopaedic patients, as well as treating pulmonology.	
Clinical or User Specifications	Maximum weight is important- the more it can carry, the better. A durable carry case is also important to improve care of the product during transport.	
Accessories or Related Consumables	In some models, padded headrest/breathing hole and arm supports may come separately. Should be included.	
Care	Do not exceed maximum carrying capacity. Standard cleaning and disinfection.	
Supplier Warranty	3 years (excluding deliberate or accidental damage).	

Examination couch: rehab

TECHNICAL SPECIFICATIONS page 737

Catalogue Number	Item Name	Available on Transversal Contract	Contract Number or Code
PHC-F-062	Examination couch: rehab	No	

Description	Examination couch with breathing hole. The top 1/3 is adjustable to 60 degrees from the horizontal.	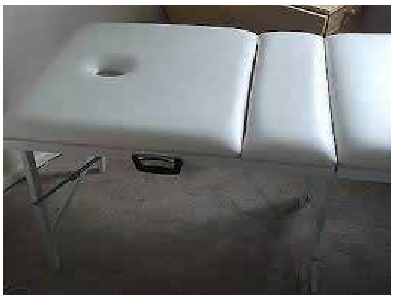
Use	Used extensively in rehabilitating acute musculoskeletal and orthopaedic patients, as well as treating pulmonology.	
Clinical or User Specifications	Must have breathing hole and adjustable top 1/3.<br>Need one of this type per facility; remainder can be standard examination couches.	
Accessories or Related Consumables	None.	
Care	Standard cleaning and disinfection.	
Supplier Warranty	2 years (excluding deliberate or accidental damage).	

Foot stool

TECHNICAL SPECIFICATIONS page 738

Catalogue Number	Item Name	Available on Transversal Contract	Contract Number or Code
PHC-F-031	Foot stool: 2-step	RT 24	RT 24-11-003

Description	Foot stool, 2-step.	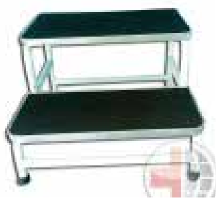
Use	To facilitate patient's access to the examination couch.	
Clinical or User Specifications	Robust, reinforced 2-step all-metal stool. White epoxy-coated tubular frame.<br>Non-scratch rubber ferrules on legs.<br>Non-slip chequered surface on steps.<br>All sharp edges are enclosed in a smooth metal band around each step.	
Accessories or Related Consumables		
Care	Standard cleaning and disinfecting.	
Supplier Warranty	2 years (excluding deliberate or accidental damage).<br>**NB**: these steps are guaranteed to carry at least 180Kg without distortion. If any example bends under normal use, contact SCM to initiate recourse proceedings against supplier.	

Kick-about

TECHNICAL SPECIFICATIONS page 739

Catalogue Number	Item Name	Available on Transversal Contract	Contract Number or Code
PHC-F-032	Kick-about	RT 24	RT 24-10-003

Description	Kick-about for bucket.	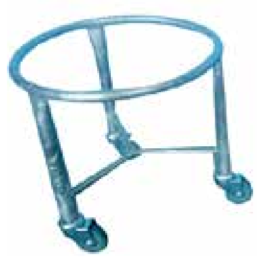
Use	For convenient temporary disposal of medical waste, during procedures.	
Clinical or User Specifications	Castor-mounted kick-about stand. To be ordered together with stainless steel bucket, RT 24-10-004.	
Accessories or Related Consumables		
Care	Standard cleaning and disinfecting.	
Supplier Warranty	2 years (excluding deliberate or accidental damage).	

Locker: bedside, steel

TECHNICAL SPECIFICATIONS page 740

Catalogue Number	Item Name	Available on Transversal Contract	Contract Number or Code
PHC-F-071	Locker: bedside, steel	RT24-2016	RT24-07-001

Description	Bedside locker in steel.	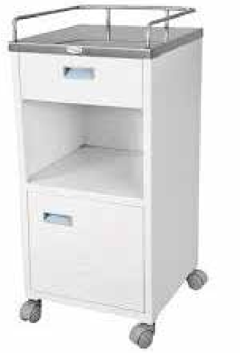
Use	Temporary storage of personal non-valuable belongings.	
Clinical or User Specifications	Stainless steel top, single drawer.<br>Must have a rail on both sides Pull-out drawer Locker mounted on 4 castors.<br>Drawer must have a durable sliding mechanism and stop to prevent total extraction of drawer.	
Accessories or Related Consumables		
Care	Standard cleaning and disinfection.	
Supplier Warranty	2 years.	

Refrigerator: vaccine, chest

TECHNICAL SPECIFICATIONS page 741

Catalogue Number	Item Name	Available on Transversal Contract	Contract Number or Code
PHC-F-049	Refrigerator: vaccine, chest model	No	

Description	Chest-type refrigerator for vaccine and blood products. 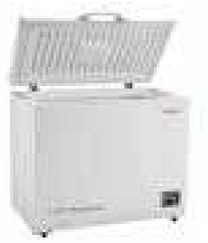
Use	Used to store vaccines and blood products.
Clinical or User Specifications	Chest-type refrigerator, minimum 100l. Alternative upright model is available, if more appropriate for the location.<br>Will keep temperature low for at least 48 hours, in the event of a power failure, i.e. over a weekend.<br>This unit is designed to maintain the recommended cold-chain conditions for vaccines and blood products, i.e. must maintain the temperature between specified upper and lower limits. The unit must provide protection against freezing, as freezing will inactivate the vaccine.<br>A separate data-logging thermometer will be used to keep a record of daily temperature variations.
Accessories or Related Consumables	None.
Care	Clean interior and exterior with standard cleaning and disinfectant solutions.
Supplier Warranty	3 years (excluding deliberate or accidental damage).

Refrigerator: vaccine, upright model

TECHNICAL SPECIFICATIONS page 742

Catalogue Number	Item Name	Available on Transversal Contract	Contract Number or Code
PHC-F-050	Refrigerator: vaccine, upright model	No	

Description	Upright refrigerator for vaccine and blood products.	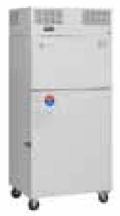
Use	Used to store vaccines and blood products.	
Clinical or User Specifications	Upright refrigerator, minimum 100l. Alternative chest model is available, if more appropriate for the location.<br>This unit is designed to maintain the recommended cold-chain conditions for vaccines and blood products, i.e. must maintain the temperature between specified upper and lower limits. The unit must provide protection against freezing, as freezing will inactivate the vaccine.<br>Will keep temperature low for at least 48 hours, in the event of a power failure, i.e. over a weekend.<br>A separate data-logging thermometer will be used to keep a record of daily temperature variations.	
Accessories or Related Consumables	None.	
Care	Clean interior and exterior with standard cleaning and disinfectant solutions.	
Supplier Warranty	3 years (excluding deliberate or accidental damage).	

Screen: bedside, wheeled, complete

TECHNICAL SPECIFICATIONS page 743

Catalogue Number	Item Name	Available on Transversal Contract	Contract Number or Code
PHC-F-063	Screen: bedside, wheeled. Complete with curtain	RT24-2016	RT24-11-009

Description	Four-panel concertina- foldable screen, consisting of steel frames on castors, and plastic curtains.
Use	To provide visual privacy when there are no other screening curtains available.
Clinical or User Specifications	Plastic curtains hinges and curtain hooks should be placed in such a way that there is no opening on the sides of curtains and between panels.
Accessories or Related Consumables	Replacement curtains RT24-11-010.
Care	Curtains washable. Standard cleaning and disinfection.
Supplier Warranty	5 years on frames, 1 year on curtains (excluding deliberate or accidental damage).

Screen: bedside, replacement curtains

TECHNICAL SPECIFICATIONS page 744

Catalogue Number	Item Name	Available on Transversal Contract	Contract Number or Code
PHC-F-064	Screen: bedside, replacement curtains	RT24-2016	RT24-11-010

Description	Replacement curtain for 4-panel folding screen on castors.
Use	To provide visual privacy when there are no other screening curtains available.
Clinical or User Specifications	Strong non-transparent plastic, sides to be strengthened, strong non-transparent, sides to be strengthened and curtains to be full fit in frame sections.
Accessories or Related Consumables	None.
Care	Standard cleaning and disinfection.
Supplier Warranty	1 year (excluding deliberate or accidental damage).

Stool: anaethetist

TECHNICAL SPECIFICATIONS page 745

Catalogue Number	Item Name	Available on Transversal Contract	Contract Number or Code
PHC-F-034	Stool: anaesthetist	RT 24	RT 24-11-002

Description	All-metal stool, height-adjustable by rotating seat.	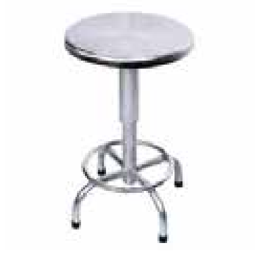
Use	To be used during intubation and other emergency procedures.	
Clinical or User Specifications	Round metal seat – smooth unpainted aluminium.<br>Castor-mounted.<br>Seat height is adjusted up or down by rotating the seat, which is mounted on a robust threaded rod.	
Accessories or Related Consumables		
Care	Standard cleaning and disinfecting.	
Supplier Warranty	2 years (excluding deliberate or accidental damage).	

Stool: dental assistant

TECHNICAL SPECIFICATIONS page 746

Catalogue Number	Item Name	Available on Transversal Contract	Contract Number or Code
PHC-F-065	Stool: dental assistant		

Description	A height-adjustable stool on castors, with an adjustable foot ring and adjustable lumbar support.
Use	Used chair-side by a dental assistant.
Clinical or User Specifications	Ergonomic stool. Height adjustment by gas cylinder or spring – lever-operated.<br>Durable upholstery for easy cleaning and durability.<br>2-way adjustable backrest.<br>5-legged base with twin-wheel castors.<br>Adjustable torso support.<br>Height-adjustable foot ring.<br>Must conform to the aesthetics of the dental chair.
Accessories or Related Consumables	
Care	Standard cleaning and disinfection.
Supplier Warranty	5 years (excluding deliberate or accidental damage).

Stool: dentist

TECHNICAL SPECIFICATIONS page 747

Catalogue Number	Item Name	Available on Transversal Contract	Contract Number or Code
PHC-F-066	Stool: dentist		

Description	An ergonomic stool used by the operator.
Use	Used chair-side by the dentist or oral hygienist.
Clinical or User Specifications	Ergonomic stool. Height adjustment by gas cylinder or spring – lever-operated.<br>Durable upholstery for easy cleaning and durability.<br>2-way adjustable backrest.<br>5-legged base with twin-wheel castors.<br>Adjustable torso support.<br>Must conform to the aesthetics of the dental chair.
Accessories or Related Consumables	
Care	Standard cleaning and disinfection.
Supplier Warranty	5 years (excluding deliberate or accidental damage).

Stool: height-adjustable, with backrest

TECHNICAL SPECIFICATIONS page 748

Catalogue Number	Item Name	Available on Transversal Contract	Contract Number or Code
PHC-F-035	Stool, height-adjustable, with back-rest		

Description	All-metal stool, height-adjustable, with backrest.	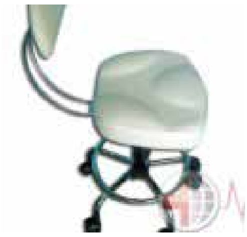
Use	To be used during dental procedures.	
Clinical or User Specifications	Round upholstered seat. Can swivel, but not tilt.<br>Castor-mounted. Castors mounted under a metal ring, which serves as a footrest.<br>Seat height is adjusted up or down by pressing a lever which actuates a gas strut.	
Accessories or Related Consumables		
Care	Standard cleaning and disinfecting.	
Supplier Warranty	2 years (excluding deliberate or accidental damage).	

Table: over- bed

TECHNICAL SPECIFICATIONS page 749

Catalogue Number	Item Name	Available on Transversal Contract	Contract Number or Code
PHC-F-075	Table: over- bed	RT24-2016	RT24-07-003

Description	Overbed table, cantilever type.	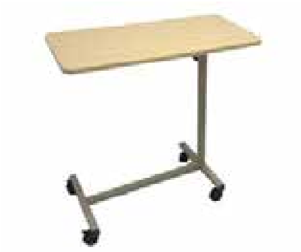
Use	Used to serve patients meals and medications.	
Clinical or User Specifications	Can be slid into position from the side of the bed, due to cantilever design.<br>Adjustable height from 740 mm to 1 090 mm. Automatic lock.<br>Height must be adjustable by patient.<br>Bonded wood melamine resin surface table-top with aluminium or similar edging.	
Accessories or Related Consumables		
Care	Standard cleaning and disinfection.	
Supplier Warranty	2 years.	

Trolley: anaesthetic

TECHNICAL SPECIFICATIONS page 750

Catalogue Number	Item Name	Available on Transversal Contract	Contract Number or Code
PHC-F-036	Trolley: anaesthetic	RT 24	RT 24-12-005

Description	Trolley, anaesthetic.	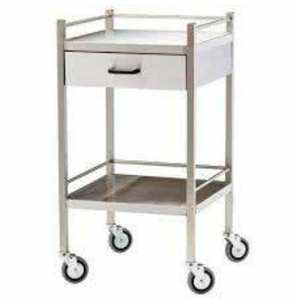
Use	To be used as couch-side trolley in consulting rooms.	
Clinical or User Specifications	Stainless steel trolley, with single drawer. On castors.	
Accessories or Related Consumables		
Care	Standard cleaning and disinfecting.	
Supplier Warranty	2 years (excluding deliberate or accidental damage).	

Trolley: emergency

TECHNICAL SPECIFICATIONS page 752

Catalogue Number	Item Name	Available on Transversal Contract	Contract Number or Code
PHC-F-040	Trolley: emergency	RT 24-2016	RT 24-12-001

Description	Emergency trolley ("crash cart").	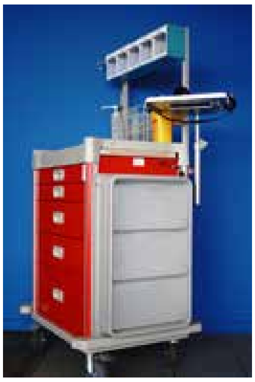
Use	For use in emergencies and resuscitations.<br>The trolley should be so configured, that it has all of the essential resuscitation equipment and drugs on board. If it becomes necessary to resuscitate a collapsed patient who cannot be moved, the trolley can then be moved to the patient.<br>The first responder will have everything to hand on the trolley.	
Clinical or User Specifications	The trolley has dedicated space or attachments for all equipment and consumables required in an emergency.<br>The trolley will be stocked with the necessary items, as stipulated in the Framework Document.	
Accessories or Related Consumables	Cardiac board, drugs insert, laryngoscope and ET tube insert.<br>Half-height plastic bins for syringes and needles. Drawer dividers optional.	
Care	Stock each drawer against a standard checklist, so that the configuration of the trolley is standard in every PHC facility.<br>Check the contents of the trolley, including the drug drawer, daily.<br>Lock the drug drawer and apply a tamper-proof seal, after each inspection.<br>Test the attached monitor and AED daily.<br>Keep an oxygen cylinder on a trolley nearby.<br>Replenish used items after each use.	
Supplier Warranty	2 years (excluding deliberate or accidental damage).	

Trolley: gas cylinder

TECHNICAL SPECIFICATIONS page 752

Catalogue Number	Item Name	Available on Transversal Contract	Contract Number or Code
PHC-F-041	Trolley: gas cylinder	RT 24	RT 24-12-003

Description	Trolley, gas cylinder.	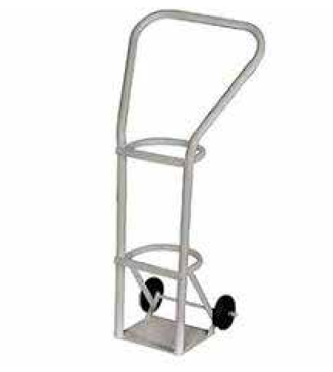
Use	To be used for moving a standard oxygen cylinder.	
Clinical or User Specifications	Trolley on wheels. Stable in vertical position.<br>To fit medium cylinder available on RT 50.	
Accessories or Related Consumables		
Care	Standard cleaning and disinfecting.	
Supplier Warranty	2 years (excluding deliberate or accidental damage).	

Trolley: medical dressing, large

TECHNICAL SPECIFICATIONS page 753

Catalogue Number	Item Name	Available on Transversal Contract	Contract Number or Code
PHC-F-039	Trolley: medical dressing, large	RT 24	RT 24-12-008

Description	Trolley, medical dressing, large.	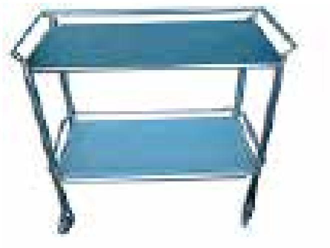
Use	General purpose medical dressing trolley.	
Clinical or User Specifications	Stainless steel trolley, with two open shelves.<br>Guard rails around shelves. On castors.	
Accessories or Related Consumables		
Care	Standard cleaning and disinfecting.	
Supplier Warranty	2 years (excluding deliberate or accidental damage).	

Trolley: medical instruments, medium

TECHNICAL SPECIFICATIONS page 754

Catalogue Number	Item Name	Available on Transversal Contract	Contract Number or Code
PHC-F-038	Trolley, medical instruments, medium	RT 24	RT 24-12-013

Description	Trolley, medical instruments, medium.	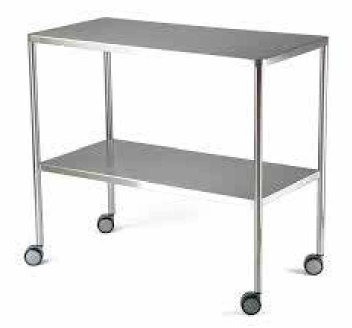
Use	General purpose medical dressing trolley.	
Clinical or User Specifications	Stainless steel, with two open stainless steel shelves.<br>No guard rails. On castors.	
Accessories or Related Consumables		
Care	Standard cleaning and disinfecting.	
Supplier Warranty	2 years (excluding deliberate or accidental damage).	

Trolley: medical dressing, small

TECHNICAL SPECIFICATIONS page 755

Catalogue Number	Item Name	Available on Transversal Contract	Contract Number or Code
PHC-F-037	Trolley: medical dressing, small	RT 24	RT 24-12-009

Description	Trolley, medical dressing small.	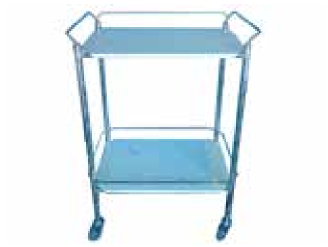
Use	General purpose medical dressing trolley.	
Clinical or User Specifications	Mild steel trolley, with two open stainless steel shelves.<br>Guard rails around shelves. On castors.	
Accessories or Related Consumables		
Care	Standard cleaning and disinfecting.	
Supplier Warranty	2 years (excluding deliberate or accidental damage).	

Trolley: medical dressing with bucket and bowl attachment

TECHNICAL SPECIFICATIONS page 756

Catalogue Number	Item Name	Available on Transversal Contract	Contract Number or Code
PHC-F-044	Trolley: medical dressing with bucket and bowl attachment	RT 24-2016	RT 24-12-010

Description	Medical dressing trolley on castors, with two shelves, a bowl and a bucket.	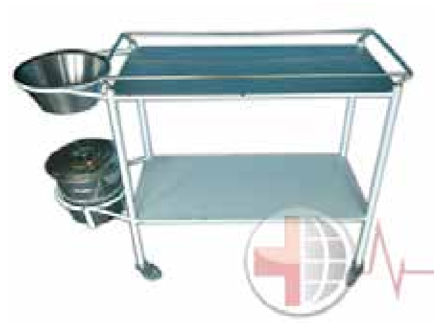
Use	Generally used in a procedure room, where instruments require rinsing during the procedure.	
Clinical or User Specifications	Trolley has two stainless steel shelves. Top shelf has a guard rail on all four sides, bottom shelf has no rail. On one end, the trolley has an upper bracket to accommodate a stainless steel bowl, and below that another bracket to hold a stainless steel bucket.	
Accessories or Related Consumables	Supplied complete with bowl and bucket.	
Care	Standard cleaning and disinfecting.	
Supplier Warranty	2 years (excluding deliberate or accidental damage).	

Trolley: patient, with mattress

TECHNICAL SPECIFICATIONS page 757

Catalogue Number	Item Name	Available on Transversal Contract	Contract Number or Code
PHC-F-051	Trolley: patient, complete with mattress	RT 24	RT 24-12-018

Description	Trolley, patient.	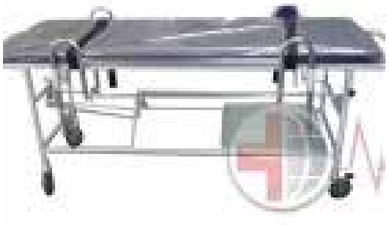
Use	To be used when moving a patient who must remain horizontal.	
Clinical or User Specifications	Trolley on castors, two swivelling and two fixed (with brakes).<br>Adjustable backrest. Collapsible safety sides. Stainless steel top.<br>Compatible mattress, 1 780x530x120mm.<br>Replaceable waterproof, non-slip mattress cover.	
Accessories or Related Consumables	Safety sides, drip rod, oxygen cylinder holder.<br>Mattress for patient trolley PHC-F-052.<br>Cover for Mattress PHC-F-053.	
Care	Standard cleaning and disinfecting.	
Supplier Warranty	2 years (excluding deliberate or accidental damage).	

Trolley: soiled instruments and linen

TECHNICAL SPECIFICATIONS page 758

Catalogue Number	Item Name	Available on Transversal Contract	Contract Number or Code
PHC-F-054	Trolley: soiled instruments and linen, complete	RT 24-2016	RT 24-12-027

Description	Trolley with three shelves for soiled instruments plus a polyester receptacle for soiled linen. 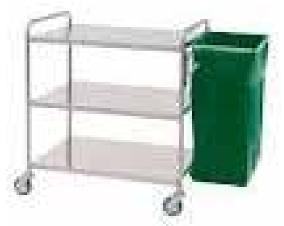
Use	To gather up soiled instruments and linen, after a procedure.
Clinical or User Specifications	The trolley has a large polyester receptacle for soiled linen, held in the open position on the frame by a retainer. Soiled linen can easily be inserted.<br>Alongside the receptacle are three stainless steel shelves, to accommodate soiled instruments.<br>The trolley is castor-mounted for easy movement into and out of rooms.
Accessories or Related Consumables	Spare polyester linen receptacle. PHC-F-055.
Care	Clean after use as with standard cleaning and disinfectant solutions.<br>The polyester receptacle is washable. A second receptacle permits rapid turnaround of the trolley.
Supplier Warranty	2 years on all components (excluding deliberate or accidental damage).

Trolley: soiled linen, complete

TECHNICAL SPECIFICATIONS page 760

Catalogue Number	Item Name	Available on Transversal Contract	Contract Number or Code
PHC-F-056	Trolley: soiled linen, complete	RT 24-2016	RT 24-12-030

Description	Soiled linen trolley.	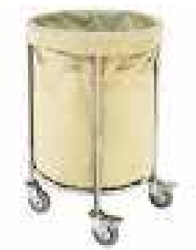
Use	For removal of soiled linen.	
Clinical or User Specifications	The trolley holds a large woven polyester linen bag.<br>The trolley is castor-mounted for easy movement into and out of rooms.	
Accessories or Related Consumables	Replaceable polyester bag, with draw cord. PHC F-057.	
Care	Clean after use with standard cleaning and disinfectant solutions.<br>Polyester bag is washable.	
Supplier Warranty	2 years on all components (excluding deliberate or accidental damage).	

Viewing box: X-ray

TECHNICAL SPECIFICATIONS page 761

Catalogue Number	Item Name	Available on Transversal Contract	Contract Number or Code
PHC-F-042	Viewing box: X-ray	RT 24	RT 24-12-013

Description	Illuminated viewing box to enhance x-rays.
Use	Use in circumstances where X-ray films are still in use.
Clinical or User Specifications	Viewer box for single full-size x-ray film.<br>Separate switch for higher-intensity lamp, to brighten area of interest on film.
Accessories or Related Consumables	None.
Care	Clean with standard cleaning and disinfectant solutions.<br>Contact departmental technical personnel if bulbs burn out.
Supplier Warranty	1 year (deliberate or accidental damage excluded).

Wheelchair: porter

TECHNICAL SPECIFICATIONS page 762

Catalogue Number	Item Name	Available on Transversal Contract	Contract Number or Code
PHC-F-024	Wheelchair: porter	RT 233-2017	42192210-00259

Description	Wheelchair, porter.	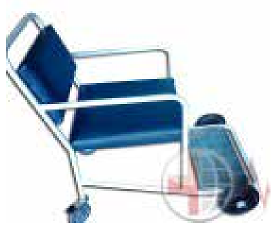
Use	For transport of disabled patients who do not require to recline.	
Clinical or User Specifications		
Accessories or Related Consumables		
Care	Standard cleaning and disinfecting.	
Supplier Warranty	2 years (excluding deliberate or accidental damage).	

General

Chair: office

TECHNICAL SPECIFICATIONS page 763

Catalogue Number	Item Name	Available on Transversal Contract	Contract Number or Code
PHC-F-001	Chair: office	No	

Description	Office chair.	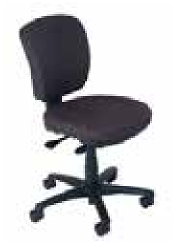
Use	For use by administrative staff.	
Clinical or User Specifications	Durable, comfortable seating. No arms. Height-adjustable, swivel and tilt.<br>Adequate support for good posture, suitable for prolonged use during working hours.<br>Castor-mounted on 5-star base. Allows maximum mobility of the user.<br>Mesh back prevents heat build-up and discomfort in warm environments.	
Accessories or Related Consumables	None.	
Care	Standard cleaning and disinfection.	
Supplier Warranty	2 years on seat.<br>5 years on frame and back.	

Chair: operator

TECHNICAL SPECIFICATIONS page 764

Catalogue Number	Item Name	Available on Transversal Contract	Contract Number or Code
PHC-F-002	Chair: operator, high back	No	

Description	Operator mesh back chair.	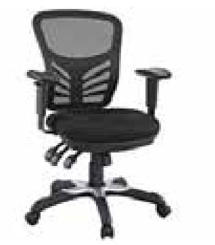
Use	For use by clinicians.	
Clinical or User Specifications	Durable, comfortable seating. No arms. Height-adjustable, swivel and tilt.<br>Adequate support for good posture, suitable for prolonged use during working hours.<br>Castor-mounted on 5-star base. Allows maximum mobility of the user.<br>Mesh back prevents heat build-up and discomfort in warm environments.	
Accessories or Related Consumables	None.	
Care	Standard cleaning and disinfection.	
Supplier Warranty	2 years on seat.<br>5 years on frame and back.	

Chair: stackable, metal frame

TECHNICAL SPECIFICATIONS page 765

Catalogue Number	Item Name	Available on Transversal Contract	Contract Number or Code
PHC-F-003	Chair: stackable	No	

Description	Stackable chair with metal frame with ABS back and seat shell.	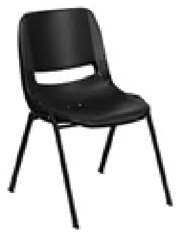
Use	Multipurpose chair, for use in temporary seating arrangements.	
Clinical or User Specifications	Powder-coated metal frame. Anti-tilt design.<br>One-piece ABS plastic back and seat shell.<br>Rubber ferrules on feet.<br>Stackable for storage purposes.	
Accessories or Related Consumables	None.	
Care	Standard cleaning and disinfection.	
Supplier Warranty	3 years on frame and shell.	

Chair: stackable, netted mid-back

TECHNICAL SPECIFICATIONS page 766

Catalogue Number	Item Name	Available on Transversal Contract	Contract Number or Code
PHC-F-004	Chair, stackable, netted mid-back	No	

Description	Occasional chair.	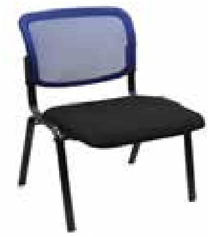
Use	For use in multipurpose rooms.	
Clinical or User Specifications	Durable, comfortable seating. Strong frame. Non-scratch rubber floor protectors.<br>Mesh back prevents heat build-up and discomfort in warm environments.	
Accessories or Related Consumables	None.	
Care	Standard cleaning and disinfection.	
Supplier Warranty	2 years on seat.<br>5 years on frame and back.	

Chair: visitor

TECHNICAL SPECIFICATIONS page 767

Catalogue Number	Item Name	Available on Transversal Contract	Contract Number or Code
PHC-F-005	Chair: visitor, mid back	No	

Description	Visitor chair.	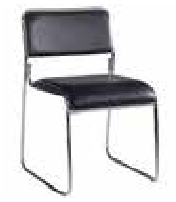
Use	Chair without arm rests for use by patients in consulting rooms and offices.	
Clinical or User Specifications	Durable, comfortable seating. Strong frame. Non-scratch rubber floor protectors.	
Accessories or Related Consumables		
Care	Standard cleaning and disinfection.	
Supplier Warranty	2 years on seat.<br>5 years on frame and back.	

Credenza

TECHNICAL SPECIFICATIONS page 768

Catalogue Number	Item Name	Available on Transversal Contract	Contract Number or Code
PHC-F-006	Credenza	No	

Description	Office credenza.	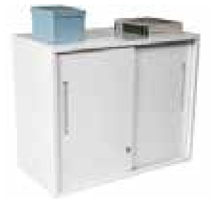
Use	For use by operational manager.	
Clinical or User Specifications	Construction and materials to match L-shaped desk.<br>Light grey top and sides of carcass, darker grey accents.<br>Double doors of same material and colour as sides and top.<br>Two shelves, adjustable spacing.<br>Height to match height of L-shaped desk.	
Accessories or Related Consumables	2 keys.	
Care	Standard cleaning and disinfection.	
Supplier Warranty	5 years on all components (excluding accidental or deliberate damage).	

Cupboard: sterile stock

TECHNICAL SPECIFICATIONS page 787

Catalogue Number	Item Name	Available on Transversal Contract	Contract Number or Code
PHC-F-011	Sterile stock cupboard	No	

Description	Cabinet for storing packs and instruments.	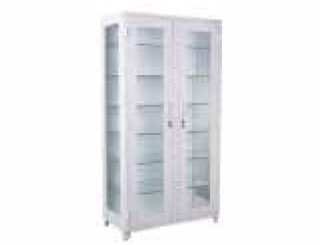
Use	Used to store packs and autoclaveable instruments after sterilisation.	
Clinical or User Specifications	All-steel cabinet, with lockable double doors. Doors have glass panes to permit view of contents without opening.<br>Six Shelves.<br>Rubber ferrules on feet, or rubber floor protectors.<br>Antibacterial coating on the interior.	
Accessories or Related Consumables	Key and spare key.	
Care	Clean according to the supplier's instructions, to preserve the antibacterial coating.	
Supplier Warranty	2 years on cabinet itself (excluding deliberate or accidental damage).<br>5 years on locks, lock bars, hinges and handles.	

TECHNICAL SPECIFICATIONS page 770

Desk: 3-drawer, L-shaped

Catalogue Number	Item Name	Available on Transversal Contract	Contract Number or Code
PHC-F-007	Desk: L-shape	No	

Description	L-shaped desk.	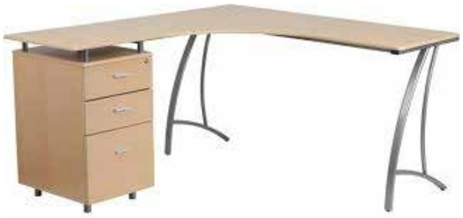
Use	For use by clinicians and clinic manager.	
Clinical or User Specifications	The desk is designed to provide adequate space for clinical purposes, without occupying unnecessary space in a room.<br>Light grey top, darker grey accents.<br>The design will allow the clinician maximum freedom, in moving from examination couch to desk and back.<br>For infection control purposes, the desk will be fixed in position, attached to a wall. Attachment will be performed by your own maintenance team, not by the supplier.<br>Drawer unit with three drawers, can be located on either side of the desk.	
Accessories or Related Consumables	Keys for lockable drawer.<br>Pen and pencil tray for top drawer.	
Care	Standard cleaning and disinfection.	
Supplier Warranty	5 years on all components (excluding accidental or deliberate damage).	

TECHNICAL SPECIFICATIONS page 772

Desk: rectangular

Catalogue Number	Item Name	Available on Transversal Contract	Contract Number or Code
PHC-F-008	Desk: rectangular	No	

Description	Rectangular desk.	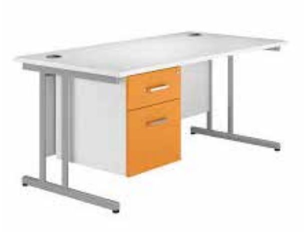
Use	For use in rooms other than consulting rooms.	
Clinical or User Specifications	The desk is designed to provide adequate space for recording vital signs, without occupying unnecessary space in a room. Can also be used in administrative areas.<br>Light grey top, darker grey accents.<br>Two drawers, to hold additional cuffs, testing equipment and materials.<br>If space permits, for infection control purposes, the desk will be fixed in position, attached to a wall. Attachment will be performed by your own maintenance team, not by the supplier	
Accessories or Related Consumables	None.	
Care	Standard cleaning and disinfection.	
Supplier Warranty	5 years on all components (excluding accidental or deliberate damage).	

Filling Cabinet: bulk

TECHNICAL SPECIFICATIONS page 774

Catalogue Number	Item Name	Available on Transversal Contract	Contract Number or Code
PHC-F-009	Filing system, bulk	No	

Description	High-density filing system.	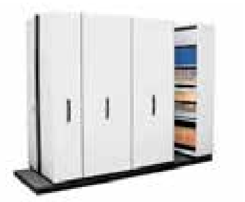
Use	The most space-efficient method of storing large numbers of files or folders. The files are stored vertically, with the long side supported on the shelf, and the open side at the top.<br>The short side facing the user, should have a self-adhesive label applied, containing the approved serial number of the file, to permit easy retrieval and re-filing.<br>Vertical steel dividers placed at intervals along the shelf, prevent the files from falling over if several files are retrieved.<br>To avoid excessive searching among cabinets, the user should develop a disciplined retrieval order, from first to last cabinet.<br>Ensure that no other user is standing between cabinets, before moving them.<br>When a file is retrieved, a brightly-coloured file-out card is inserted as a place-holder, to facilitate re-filing. The card also serves as a visual cue for a missing file.<br>Calculation of requirements:<br>Number of active files + number of expected new files per year estimate for 5 years) divided by 100 = number of linear metres of filing (each file = 1cm). Divide by the number of linear metres per cabinet (typically 7.2m., may vary by model) = number of cabinets<br>Dividers: number of bays X 6 shelves per bay X 12 dividers per shelf<br>File-out cards: Daily headcount + 10% extra.	

Clinical or User Specifications	Mobile steel cabinets (called bays) with adjustable horizontal shelves. The cabinets move forward and backward only, as their wheels run in steel rails which are fixed to the floor, and levelled by the supplier, to provide a low-friction platform for motion. The space between the rails is filled with carpeted panels, to provide hindrance-free movement for users.<br>At the end of the working day, the cabinets are pushed to the fully-closed position, and locked.<br>The cabinets may have physical devices to make forward and backward movement easier by a smaller user.<br>The cabinets have sufficient friction to prevent uncontrolled movement.<br>Cabinets vary in size according to the manufacturer, but are approximately 2200mm high X 1000 to1200mm wide. There are two standard depths: 360mm and 430mm. The greater depth is required for pull-out filing systems, and is not recommended for use with lateral files – the greater depth is wasted (and more expensive).<br>The track length is normally calculated as the sum of the depths of all the required cabinets, plus 800mm access room. However, it is recommended that the access room should be a minimum of 1000mm.<br>The bottom shelf is not usable for lateral files, and should be reserved for filing consumables and accessories.<br>In rooms with low ceilings, it may be possible to have cabinets custom manufactured to a lower height.<br>N.B. When fully loaded, such a system may exceed the weight-carrying capacity of the floor, and cause damage to the floor. The floor should be certified by the Provincial engineering personnel, before finalising procurement.<br>In a smaller room, it may be necessary to mount cabinets in tandem, i.e. two side-by-side. The middle bays (i.e. excluding first and last) are usually bolted back to back, for improved efficiency.
Accessories/related consumables	Vertical dividers, steel PHC-F-010.<br>File-out cards PHC-C-005.<br>Side-access files and self-adhesive file labels (see Consumables – General – Stationery).
Care	Clean with standard cleaning solutions and dusting.<br>Lock at end of working day, in compliance with POPPI Act.<br>Visually inspect for file-out cards (i.e. misfiled or missing files) weekly.<br>Archive files according to the Archiving policy – the system cannot expand indefinitely.<br>Do not force cabinets if movement becomes difficult – contact the supplier.
Maintenance	Any adjustments (cabinets distorted, difficult to move or lock) must be carried out by the supplier.
Supplier Warranty	5 years (excluding deliberate or accidental damage).

Filing cabinet: four-drawer

TECHNICAL SPECIFICATIONS page 776

Catalogue Number	Item Name	Available on Transversal Contract	Contract Number or Code
PHC-F-012	Filing cabinet, 4-drawer	No	

Description	4-drawer filing cabinet.	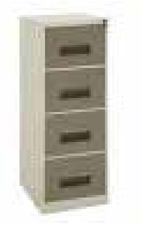
Use	For use in limited space situations, and for use in OM office for personnel records, etc. File containers are placed in each drawer, to prevent files from falling over and sliding around, when drawer is not full.	
Clinical or User Specifications	All-steel construction.<br>Four drawers.<br>Single lock with internal locking mechanism to secure all four drawers.	
Accessories/related consumables	2 keys.<br>File containers PHC-C-006.<br>Top-access files and self-adhesive file labels (see Consumables – General – Stationery.	
Care	Clean with standard cleaning solutions and dusting.<br>Lock at end of working day, in compliance with POPI Act.	
Maintenance	Not required in normal use.	
Supplier Warranty	5 years (excluding deliberate or accidental damage).	

Floor polisher: electric, single disc

TECHNICAL SPECIFICATIONS page 777

Catalogue Number	Item Name	Available on Transversal Contract	Contract Number or Code
PHC-F-020	Floor polisher: electric, single disc	No	

Description	Electric floor scrubber and polisher.	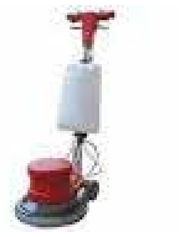
Use	For cleaning of floors and removing old polish or sealants.	
Clinical or User Specifications	Heavy-duty electric single rotary disc machine.<br>The weight of the machine is sufficient to ensure a thorough cleaning and scrubbing action.<br>Different discs for polishing, scrubbing or removing old sealants.	
Accessories or Related Consumables	General purpose cleaning disc PHC-C-208.<br>Scouring disc for removing old finish PHC-C-209.	
Care	Standard cleaning and disinfection.<br>Change discs when worn, or for different uses.<br>If cable is damaged or worn, contact maintenance personnel.	
Supplier Warranty	2 years on all components except discs (excluding deliberate or accidental damage).	

Freezer: chest, human tissue

NO TECHNICAL SPECIFICATIONS NONE

Catalogue Number	Item Name	Available on Transversal Contract	Contract Number or Code
PHC-F-076	Freezer: chest, human tissue	No	

Description	Domestic-type small chest freezer.
Use	For storage of placentas, foreskins from MMC, and products of conception from TOP.
Clinical or User Specifications	Small chest type freezer.<br>Approximately 120l capacity.<br>Energy-efficient.<br>Lockable lid.
Accessories or Related Consumables	2 keys.
Care	Standard cleaning and disinfection.
Supplier Warranty	2 years.

Microwave oven: 25l, 900W manual

TECHNICAL SPECIFICATIONS page 778

Catalogue Number	Item Name	Available on Transversal Contract	Contract Number or Code
PHC-F-021	Microwave oven	No	

Description	Microwave oven.	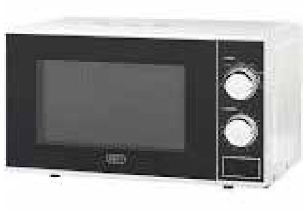
Use	For rapid cooking and re-heating of meals.	
Clinical or User Specifications	Standard manual microwave oven.	
Accessories or Related Consumables	Oven plate and carousel.	
Care	Clean after use according to manufacturer's instructions.	
Supplier Warranty	2 years (excluding deliberate or accidental damage).	

Mirror

TECHNICAL SPECIFICATIONS page 779

Catalogue Number	Item Name	Available on Transversal Contract	Contract Number or Code
PHC-F-014	Mirror	No	

Description	Mirror.	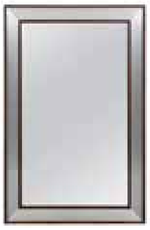
Use	For all areas where there is a hand washbasin.	
Clinical or User Specifications	Mirror in non-rusting frame.<br>Must be affixed to the wall, in proximity to the washbasin.	
Accessories or Related Consumables	To be supplied with wall mounting hardware (stainless steel screws).	
Care	Standard cleaning and disinfection.	
Supplier Warranty	1 year on frame (excluding accidental or deliberate damage).	

Refrigerator: bar type

TECHNICAL SPECIFICATIONS page 780

Catalogue Number	Item Name	Available on Transversal Contract	Contract Number or Code
PHC-F-022	Refrigerator: bar type	No	

Description	Small bar type refrigerator.	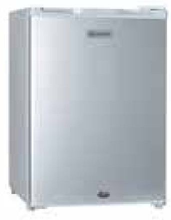
Use	For storing specimens awaiting collection by the NHLS.	
Clinical or User Specifications	Domestic –type small bar refrigerator.<br>To be kept in a protected location.<br>For specimen storage only.	
Accessories or Related Consumables		
Care	Clean external and internal surfaces with standard cleaning and disinfectant solutions.<br>Danger of cross-contamination if used for other purposes.	
Supplier Warranty	3 years on all components (excluding deliberate or accidental damage).	

Refrigerator: double door

TECHNICAL SPECIFICATIONS page 781

Catalogue Number	Item Name	Available on Transversal Contract	Contract Number or Code
PHC-F-047	Refrigerator: double door	No	

Description	Upright, double door refrigerator, with freezer compartment for larger facilities.	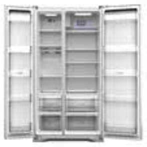
Use	Used to store temperature sensitive medicines, and to prepare icepacks for cooler boxes.	
Clinical or User Specifications	Upright refrigerator, approximately 350l.<br>Self-closing, lockable doors.<br>Automatic defrost.<br>Forced air circulation.<br>Will keep temperature low for at least 4 hours, in the event of a power failure.<br>5 or 6 shelves per side.<br>Alarm for open door, system failure, power failure.	
Accessories or Related Consumables	None.	
Care	Clean interior and exterior with standard cleaning and disinfectant solutions.	
Supplier Warranty	2 years on compressor, 5 years on other components (excluding deliberate or accidental damage).	

Refrigerator: single door

TECHNICAL SPECIFICATIONS page 782

Catalogue Number	Item Name	Available on Transversal Contract	Contract Number or Code
PHC-F-043	Refrigerator: single door	No	

Description	Upright, single-door refrigerator, with freezer compartment. 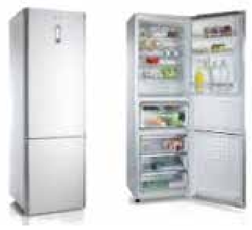
Use	Used to store temperature sensitive medicines, and to prepare icepacks for cooler boxes.
Clinical or User Specifications	Upright refrigerator, approximately 260l.<br>Self-closing, lockable door.<br>Automatic defrost.<br>Forced air circulation.<br>Will keep temperature low for at least four hours, in the event of a power failure.<br>5 or 6 shelves.<br>Alarm for open door, system failure, power failure.
Accessories or Related Consumables	None.
Care	Clean interior and exterior with standard cleaning and disinfectant solutions.
Supplier Warranty	2 years on compressor, 5 years on other components (excluding deliberate or accidental damage).

Seating: 2-seater

TECHNICAL SPECIFICATIONS page 783

Catalogue Number	Item Name	Available on Transversal Contract	Contract Number or Code
PHC-F-015	Seating: waiting area, 2-seater	No	

Description	All metal bench style seating for waiting areas ("airport style").	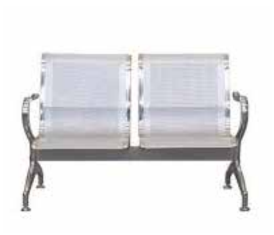
Use	2-seater unit for waiting area.	
Clinical or User Specifications	Heavy-duty seating for waiting area. Can be used in combination with other units of the same or different sizes, to achieve an aesthetically-pleasing and durable environment for patients waiting to be seen.	
Accessories or Related Consumables	None.	
Care	Standard cleaning and disinfection.	
Supplier Warranty	5 years on all components (excluding accidental or deliberate damage).	

Seating: 3-seater

TECHNICAL SPECIFICATIONS page 784

Catalogue Number	Item Name	Available on Transversal Contract	Contract Number or Code
PHC-F-016	Seating: waiting area, 3-seater	No	

Description	All metal bench style seating for waiting areas ("airport style").	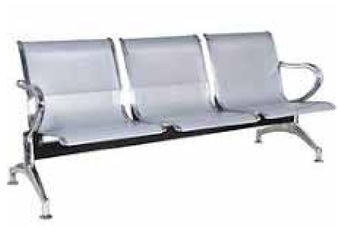
Use	3-seater unit for waiting area.	
Clinical or User Specifications	Heavy-duty seating for waiting area. Can be used in combination with other units of the same or different sizes, to achieve an aesthetically-pleasing and durable environment for patients waiting to be seen.	
Accessories or Related Consumables	None.	
Care	Standard cleaning and disinfection.	
Supplier Warranty	5 years on all components (excluding accidental or deliberate damage).	

Seating: 4-seater

TECHNICAL SPECIFICATIONS page 785

Catalogue Number	Item Name	Available on Transversal Contract	Contract Number or Code
PHC-F-017	Seating, waiting area, 4-seater	No	

Description	All metal bench style seating for waiting areas ("airport style").	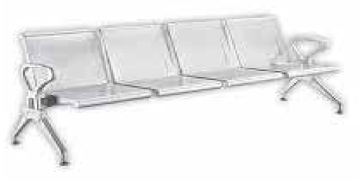
Use	4-seater unit for waiting area.	
Clinical or User Specifications	Heavy-duty seating for waiting area. Can be used in combination with other units of the same or different sizes, to achieve an aesthetically-pleasing and durable environment for patients waiting to be seen.	
Accessories or Related Consumables	None.	
Care	Standard cleaning and disinfection.	
Supplier Warranty	5 years on all components (excluding accidental or deliberate damage).	

Steel cabinet: lockable, 6 shelves

TECHNICAL SPECIFICATIONS page 786

Catalogue Number	Item Name	Available on Transversal Contract	Contract Number or Code
PHC-F-018	Stationery cabinet	No	

Description	Stationery cabinet.	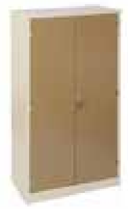
Use	Used to store daily medication issuing quantities in consulting rooms. Can also be used for storage of garden tools and equipment.	
Clinical or User Specifications	All-steel cabinet, with lockable double door.<br>Six Shelves.	
Accessories or Related Consumables	Key and spare key.	
Care	Standard cleaning and disinfection.	
Supplier Warranty	2 years on cabinet itself (excluding deliberate or accidental damage).<br>5 years on locks, lock bars, hinges and handles.	

Storage rack: exercise ball, wall-mounted

TECHNICAL SPECIFICATIONS page 788

Catalogue Number	Item Name	Available on Transversal Contract	Contract Number or Code
PHC-F-067	Storage rack: exercise ball, wall-mounted	No	

Description	Frame, able to accommodate all Bobath/physio balls. Wall-mounted.	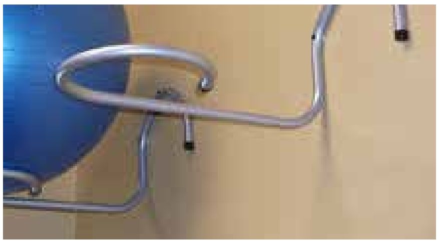
Use	Keeps the balls off the ground when not in use, and conserves free space.	
Clinical or User Specifications	Holds up to 4 balls, either in a frame or on rings.<br>Epoxy-coated metal.<br>Mounting height must allow easy access, while maximising free space below the rack.	
Accessories or Related Consumables	All wall-mounting hardware.	
Care	Standard cleaning and disinfection.	
Supplier Warranty	2 years.	

Table: folding

TECHNICAL SPECIFICATIONS page 789

Catalogue Number	Item Name	Available on Transversal Contract	Contract Number or Code
PHC-F-068	Table: folding	No	

Description	Folding table, robust, portable.	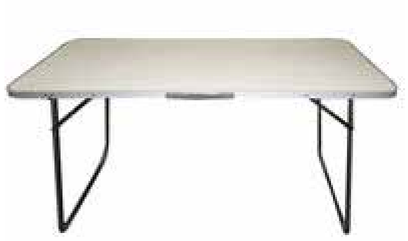
Use	Administrative work and clinical assessments, especially by outreach teams.	
Clinical or User Specifications	Minimum size 120 cm X 75cm (larger preferred), height 70-75cm.<br>Impact- and scratch-resistant polyethylene top. Dark colour preferred.<br>Aluminium fame preferred. Legs fold under top and stay in folded position.<br>Top may be single piece or preferably two hinged pieces, for ease of transportation.<br>Carrying handle.	
Accessories or Related Consumables	None.	
Care	Standard cleaning and disinfection.	
Supplier Warranty	1 year.	

Table: trapezoidal

TECHNICAL SPECIFICATIONS page 790

Catalogue Number	Item Name	Available on Transversal Contract	Contract Number or Code
PHC-F-019	Table: trapezoidal	No	

Description	Trapezoidal table.	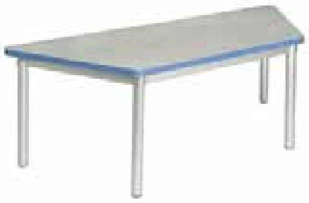
Use	Multi-purpose table.	
Clinical or User Specifications	The design allows several tables to be configured in various ways (e.g. U-shape, circle) to meet specific needs (committee meetings, education sessions, etc.).	
Accessories or Related Consumables	None.	
Care	Standard cleaning and disinfection.	
Supplier Warranty	5 years on all components (excluding accidental or deliberate damage).	

Tumble dryer

TECHNICAL SPECIFICATIONS page 792

Catalogue Number	Item Name	Available on Transversal Contract	Contract Number or Code
PHC-F-045	Tumble dryer	No	

Description	Tumble dryer, electric.	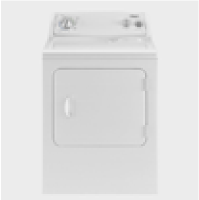
Use	For drying of laundry, where air drying is not practical.	
Clinical or User Specifications	Large capacity, electric tumble dryer.<br>Front-loading.<br>Vented type – must be mounted against outside wall, with hole through the wall for moisture exhaust hose.<br>Can be mounted on the wall at eye-level to minimise floor space usage.	
Accessories or Related Consumables	Venting hose and wall-mounting bracket supplied with the unit.	
Care	Must be installed by technical/maintenance personnel, as a hole must be drilled through the wall.<br>Clean external surfaces with standard cleaning and disinfectant solutions.<br>Clean the lint filter after use, or when the filter indicator appears during use (see supplier instructions).	
Supplier Warranty	3 years on all components (excluding deliberate or accidental damage).	

Vacuum cleaner

TECHNICAL SPECIFICATIONS page 793

Catalogue Number	Item Name	Available on Transversal Contract	Contract Number or Code
PHC-F-046	Vacuum cleaner	No	

Description	Vacuum cleaner: Canister type, electric.	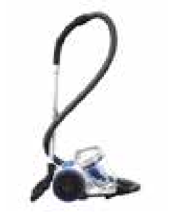
Use	For vacuuming premises.	
Clinical or User Specifications	Domestic vacuum cleaner – not intended for use throughout the facility, but for hard-to-reach areas, fabric surfaces.	
Accessories or Related Consumables	Complete with flexible hose, extension tubes, brushes and attachments, set of filters, instruction manual.<br>Replacement filter set: vacuum cleaner PHC-C-216.	
Care	Clean external surfaces with standard cleaning and disinfectant solutions.<br>Clean the filters after use (see supplier instructions).<br>Replace the filters when vacuum efficiency deteriorates.	
Supplier Warranty	2 years on motor and body.	

Washing machine: front loader

TECHNICAL SPECIFICATIONS page 794

Catalogue Number	Item Name	Available on Transversal Contract	Contract Number or Code
PHC-F-058	Washing machine, front loader	No	

Description	Washing machine, front loader, electric.
Use	For washing of soiled linen on premises – for smaller facilities.
Clinical or User Specifications	Medium capacity electric front loader washing machine. 8 to 9kg dry load.<br>Automatic, with pre-set washing, rinsing and spinning cycles.
Accessories or Related Consumables	
Care	Must be installed by technical/maintenance personnel – requires levelling on the floor, connection to cold water supply.<br>Clean external surfaces with standard cleaning and disinfectant solutions.<br>Clean the filter after use (see supplier instructions).
Supplier Warranty	3 years on all components (excluding deliberate or accidental damage).

Washing machine: top loader

TECHNICAL SPECIFICATIONS page 795

Catalogue Number	Item Name	Available on Transversal Contract	Contract Number or Code
PHC-F-048	Washing machine: top loader	No	

Description	Washing machine: top loader, electric.	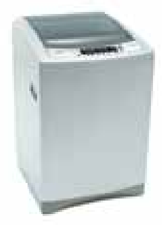
Use	For washing soiled linen on premises.	
Clinical or User Specifications	Large capacity electric top loader washing machine.<br>Automatic, with pre-set washing, rinsing and spinning cycles.	
Accessories or Related Consumables		
Care	Must be installed by technical/maintenance personnel – requires levelling on the floor, connection to cold water supply.<br>Clean external surfaces with standard cleaning and disinfectant solutions.<br>Clean the filter after use (see supplier instructions).	
Supplier Warranty	3 years on all components (excluding deliberate or accidental damage).	

E
APPENDICES

appendix 1

technical specifications

Introduction to the Technical Specifications

WHAT ARE TECHNICAL SPECIFICATIONS?

Technical specifications go into detail about the functions, construction and performance of items of equipment or instrumentation, instruments, consumables and furniture, used in Primary Health Care facilities.

The purpose is to ensure that items procured are fit-for-purpose, and deliver value for money. A cheap alternative which is inaccurate, or fails prematurely, is a waste of money, and not fit-for-purpose.

Items such as blood pressure monitors which are designed for occasional domestic use, are not suitable for repetitive daily use in a busy clinic. The specifications therefore spell out the type of machine or instrument which will be suitable, i.e. the specifications set a minimum acceptable standard.

The specifications address several areas: the required functionality and construction, accepted standards (local or international) that must be met, and certain performance levels that must be met.

The specifications further note what accessories must be supplied with the item, what consumables may be needed during the use of the item, specific actions required of the suppliers, and notes to Supply Chain Management personnel regarding special aspects of procurement.

A WORD ABOUT COMMON STANDARDS

ISO9001/2 or ISO13485 certification. These standards relate to quality management systems – in the case of ISO13485, "for a comprehensive quality management system for the design and manufacture of medical devices".

In this catalogue, ISO9001/2 and ISO13485, are treated as **default minimum standards**, and are not named separately. They are applicable to all medical equipment, instruments and consumables even if not explicitly stated, and particularly when no other standard is available. However, these standards are necessary, but not sufficient conditions, for ensuring the quality and durability of medical devices – they say nothing about the devices themselves.

CE marking is mandatory for devices for sale in the European Economic Area, and shows that a device conforms to all applicable directives for its category.

The directives promote *safety, health and environmental protection*. The CE marking is obtained largely by a self-certification process. Some categories of devices require assessment of conformity by an authorised third party (called a Notified Body e.g. TUV).

STANDARDS APPROACH IN THIS CATALOGUE

An attempt has been made to find more specific standards, to improve quality and fitness-for-purpose.

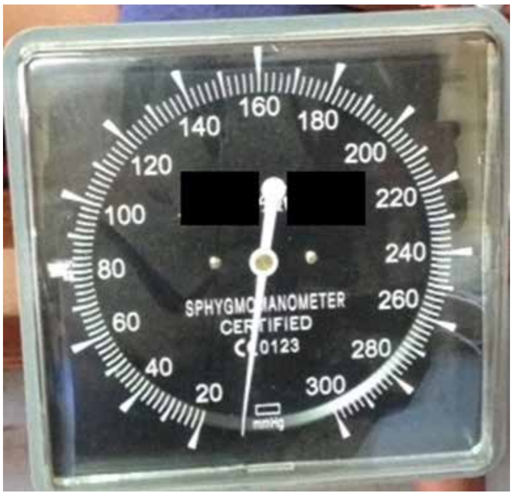

In this example of one of many poor-quality, short-lived sphygmomanometers taken from one of our clinics, the CE0123 can be seen prominently. 0123 is the code for the Notified Body, in this case TUV. In this case, conformity with the directives, assumed to be authentic, is not enough – it can be seen that the needle has not returned to the proper resting position (the machine had not functioned for long). That machine could be inaccurate by as much as 10mmHg. – more than enough to misdiagnose someone as hypertensive, and begin chronic treatment unnecessarily. We would prefer certification against EN1060-1:1995, EN1060-2:1996+A1:2009. In some cases, even such standards are insufficient, and we have recommended that clinical studies validating items of equipment (such as sphygmomanometers) should be demanded. This is becoming best-practice in procurement in other jurisdictions; since it is unlikely that we are paying less for these products than other countries, there is no reason to accept lower quality.

Precedence has been given to **South African National Standards,** where they exist.

WHERE SPECIFIC STANDARDS ARE NOTED IN THIS CATALOGUE, COMPLIANCE IS MANDATORY

There has been widely-reported fraud around the use of standards and markings. Requesting certificates with bids is necessary, but verifying their authenticity is also necessary. Should an item not live up to the applicable standards, investigation and action must follow. It is therefore the duty of healthcare workers to report poor performance of catalogue items.

Regrettably, for many items there are as yet no standards. However, standards are constantly evolving, and new standards are emerging. The standards in this catalogue will be updated from edition to edition.

technical **specifications**

**CLINICAL EQUIPMENT**	**542**
Diagnostic	***542***
Essential	***570***
Specialised	***577***
Resuscitation	577
Point of Care Testing	599
Sterilisation	602
Oral Health	608
**GENERAL EQUIPMENT**	**631**
Building maintenance	***631***
Cleaning	***667***
Cold chain	***686***
Fire safety	***690***
Garden	***693***
Kitchen	***712***
Miscellaneous	***716***
**FURNITURE AND APPLIANCES**	**724**
Clinical	***724***
General	***763***

Clinical equipment

Diagnostic

Catalogue Number	Item Name	Available on Transversal Contract	Contract Number or Code
PHC-E-115	***Sphygmomanometer: aneroid***	No	

Functional Specifications	Mounted.<br>Aneroid sphygmomanometer.
Applicable Standards	Instrument must conform to EN 1060-1: 1995, Non-invasive sphygmomanometers **Part 1**: General Requirements; European Standard EN 1060-2: 1996, Non-invasive sphygmomanometers.<br>**Part 2**: General Requirements; or equivalent (e.g. BS EN ISO 81060-1:2012) , BS EN 1060-2:1995+A1:2009, Non-invasive sphygmomanometers: Supplementary requirements for mechanical sphygmomanometers.<br>Certificate to be supplied. Independent published studies preferred.<br>Casters compliant with SABS 621.
Performance Specifications	Dial to be large, clear, high-contrast.<br>Marked in 1mm Hg divisions.<br>Precision: Standard deviation <1mm Hg in 30 consecutive measurements at 120/80 mmHg.<br>Must be cleanable and disinfectable with standard solutions.
Accessories	Must be supplied with re-usable adult, large adult and child cuffs. Sizes to be clearly marked on cuffs.<br>Cuffs may be one-piece or two-piece.<br>Must have options to attach to the wall unit of an ophthalmoscope/otoscope wall-mounted cradle, or directly onto a wall, or onto an instrument rail or pole. Mounting hardware must be provided as options, with pricing. In either case, the mounting system must include a cradle for the cuff, when not in use.<br>Mobile floor stand can be offered as an option (5-star base, casters compliant with SABS 621).

Warranties	At least 2 year guarantee on cuffs **and** covers (including bladders, tubes, outer covers, inflation bulbs, air inlet valves, release valves, filters and cuff fastening mechanism – velcro or other). Cuffs and covers must be easily cleanable.<br>At least 10 year guarantee on instrument. At least 10 year calibration guarantee by supplier/ manufacturer. Calibration frequency, procedure and requirements to be clearly stated.<br>Where calibration can be performed by user technicians, any necessary proprietary reference instruments to be offered with pricing.
Instructions to Suppliers	Supplier must specify available after-sales support, with relevant pricing.
Note to Procurement	Spare cuffs in four sizes and covers, bulbs, tubes, inlet and release valves, must be in series with this instrument.<br>The fact that an instrument is marked CE 0123, is no guarantee of the accuracy or durability of the instrument, or suitability for the South African operating environment.

Catalogue Number	Item Name	Available on Transversal Contract	Contract Number or Code
PHC-E-116	***Blood pressure monitor: automated, non-invasive, desktop model***	No	

Functional Specifications	The unit will automatically inflate and deflate the cuff once a switch is pressed.<br>Automatic timed repetitive readings are not required, as this machine will not be used for ongoing monitoring. However, a unit which automatically performs several measurements and displays the average, would be desirable.<br>**Modes**: Manual/stat (optionally average of multiple readings). Continuous monitoring not required.<br>Given the operational environment, the smallest number of controls is preferred.<br>Ideally, unit will automatically go to power-saving mode between measurements.<br>Although not mandatory, external communication port would be an advantage.<br>Could be optional (USB or RJ).<br>Must be suitable for adults, children and neonates.<br>The unit must use the oscillometric method of measurement.<br>Unit should have optional module to measure temperature, or alternative model incorporating temperature measurement should be proposed.<br>Temperature measured and displayed in degrees Centigrade, state range available.<br>Values must be displayed numerically via LED or backlit LCD (colour or monochrome). Must be clearly visible under all lighting conditions.<br>Display should also indicate battery- and charging status.<br>Auto zero when unit is switched on reusable cuffs must be supplied: Large Adult, Adult, paediatric, neonatal.<br>Cuffs and cuff covers must be washable, disinfectable.<br>The air hose must be at least 1,5 metres long.<br>The unit must charge from 220 V, 50 Hz mains supply.<br>Rechargeable battery and charger incorporated in the unit.<br>The battery must be charged while the unit is operating from the mains supply.<br>Battery must be replaceable.<br>Battery charger to be incorporated in the unit.<br>The battery must automatically take over in the event of a mains power failure.<br>The length of the mains cable must be a minimum of three metres.<br>The battery must operate for at least eight hours with measurements every five minutes.<br>Data storage: last reading, or last average.<br>The unit must be impervious to liquids.<br>The case of the unit must be robust and impact resistant. Must have mounting point for wall and pole.

Applicable Standards	Measurement accuracy must have been clinically validated (AASI/AAMI SP10-2002, BHS protocol, or ESH protocol). PROOF TO BE SUBMITTED, PREFERABLY PEER-REVIEWED PUBLISHED REPORT.<br>Unpublished independent clinical validation study may be accepted in absence of peer-reviewed published report. Minimum is in-house clinical validation study and report.<br>Evidence of measurement repeatability required (SD <1mm Hg from 30 consecutive measurements of 120/80mm Hg).<br>Must comply with latest version of EN 1060-4, EN 1060-3 with Amendment A1:2005.<br>Supplier must supply certificate issued by Directorate of Radiation Control of the Department of Health, in terms of the Hazardous Substances Act (Act 15 of 1973).
Performance Specifications	State whether measurements are performed on inflation or deflation part of cycle.<br>State BP measurement algorithm.<br>Pressure measurements:<br>**Adult**: Systolic: 30-270mm Hg, Diastolic: 20-240mm Hg, MAP: 20-250mm Hg<br>**Paediatric**: Systolic: 30-160mm Hg, Diastolic: 20-120, MAP: 20-120mm Hg<br>**Neonate**: Systolic: 30-130mm Hg, Diastolic: 20-100mm Hg, MAP: 20 – 120mm Hg<br>**Heart rate**: 30-240 /min. Indication of pulse irregularity desirable<br>**Low battery**: Alarm at least 10 minutes before battery power is exhausted<br>**Over pressure protection**: Automatic deflation at 300mm Hg adult, 150mm Hg neonate<br>**Average measurement time**: approximately 30s.<br>If measurement exceeds two minutes the cuff must deflate, an alarm must sound and likely faults indicated.<br>Supplier to state Mean Time Before Failure for Unit and any parts which may have different MTBF.
Accessories	**Reusable cuffs must be supplied**: Large adult, adult, paediatric, neonatal.<br>Cuffs and cuff covers must be washable, disinfectable.
Warranties	3 years on all parts (including cuffs and cuff covers) except battery.<br>Specify expected battery life under 8 hr continual operations, daily cycling.<br>Guarantee to include calibration.

Instructions to Suppliers	Supplier to stipulate any costs associated with service over the Guarantee period, and whether a service plan is available (specify price).<br>Supplier to stipulate recommended recalibration intervals.<br>Cuffs and covers must be cleanable and disinfectable. Indicate whether sterilisable, preferred method, and number of cycles.<br>Supplier must specify available after-sales support, with relevant pricing.<br>Quote prices inc.l VAT for:<br>Large adult cuff<br>Adult cuff<br>Paediatric cuff<br>Neonatal cuff<br>Air hose<br>Battery<br>Replaceable main board (if user serviceable)<br>Temperature sensor (if disposable, quote price per 100)<br>Optional wall-mounting bracket<br>Optional pole-mounting fixture<br>Optional bracket to fix unit to a table top<br>Optional mobile stand on 5-star base with non-marking castors SABS 601)<br>Optional cradle for cuff when not in use (wall/pole/stand)<br>Supplier to deliver and commission units and train users.
Note to Procurement	The following items should be in series with this instrument:<br>Large adult cuff<br>Adult cuff<br>Paediatric cuff<br>Neonatal cuff<br>Air hose<br>Battery<br>Replaceable main board (if user serviceable).<br>Temperature sensor (if disposable, quote price per 100)<br>Optional wall-mounting bracket<br>Optional pole-mounting fixture.<br>**Note**: the above specifications are more stringent than RT 2 Item code 42272501-00002, and should be referred to for future procurement. The item in RT 2 has continuous monitoring capabilities, which are not required. The lack of quality measures means that there are a wide range of units on offer. This defeats standardisation.

Catalogue Number	Item Name	Available on Transversal Contract	Contract Number or Code
PHC-E-265	***Cardiotocograph***	RT2-2016	42181701-00005

Functional Specifications	Required for external monitoring of foetal heart rate (FHR), and external monitoring of uterine activity of pregnant patients.<br>Twins monitoring is required.<br>Must perform automatic self-check when switched on.<br>There must a visible Foetal Heart Beat indicator which will flash with each detected heart beat.<br>The unit must provide an audio signal, with variable volume, for every heart beat detected.<br>The foetal heart rate must be measured by means of wide beam pulsed Doppler ultrasound, with auto correlation processing.<br>Must be able to detect and display multiple FHR while discriminating between different foetuses.<br>The transducer must be manufactured of high-impact plastic that is robust and watertight.<br>The belts and buckles supplied for positioning of the transducer must allow easy angling for optimum positioning.<br>Adjustable baseline position.<br>A test button must be provided that produces a series of pulses for calibration which must be recorded and displayed.<br>Display: > 16 cm (corner to corner).<br>Displays must be clearly visible under all lighting conditions.<br>Must allow Maternal Heart Rate (MHR) and Fetal Heart Rate(FHR) coincidence detection.<br>The following parameters must be displayed:<br>Foetal heart rate approximately 50 to 200 beats per minute<br>Uterine activity: approximately 0 to 100 relative units.<br>Foetal heart rate must update once per second when FHR is greater than 60 and once each beat when FHR is less than 60.<br>The monitor must have the following controls:<br>Volume Up/Down<br>TOCO/IUP Zero<br>Printer On/Off<br>Printer fast forward.<br>Must have audible and visual alarms for low/high FHR.<br>Electronic storage data capacity.<br>RECORDER<br>Integrated dual channel thermal recorder.<br>Dual heart rate recording for twins must be available.<br>All dot print test.<br>The strip chart paper must be easy to load.

	TRANSDUCERS<br>Ultrasound and toco transducers must be supplied.<br>All transducers must be of very robust design. Suppliers must guarantee shock resistance on the transducers e.g. will withstand a drop from one metre high onto a hard surface.<br>Suppliers must state whether the ultrasound transducer is repairable and which parts are replaceable or whether it is a sealed unit that must be replaced when faulty.<br>The ultrasound transducer cable must be a minimum of 2 m long.<br>Connecting plug must be keyed and colour coded to avoid it being plugged or forced into the wrong receptacle on the unit.<br><br>GENERAL<br>The unit must operate off 220V, 50Hz power supply.<br>All metal parts must be earthed.<br>Mains cable at least three metres long with SABS approved 15A three prong plug.<br>Must be fitted with rechargeable batteries.<br>The unit must be fitted with an internal battery charger.<br>Indicator for battery status.<br>Must store trends for at least 8 hours.<br>A cart/trolley must be supplied.<br>The cart must have four castor wheels, two of them with brakes.<br>The cart must have a basket/holder for accessories.
Applicable Standards	ISO 80601-1-11:2015.
Performance Specifications	The oscillator frequency < 1,2 MHz.<br>Ultrasound intensity must be less than 10 mW/cm2.<br>Foetal heart rate:<br>Range: approximately 50 to 210 beats per minute<br>Vertical chart: at least 7 cm<br>Vertical scale sensitivity: approximately 20 bpm per cm<br>Resolution: 1 bpm.<br>Uterine activity:<br>Range: 0 – 100 units<br>Vertical chart: at least 4 cm<br>Vertical scale sensitivity: approximately 25 relative units per cm<br>Resolution: 1 relative unit.<br>Paper chart speed must be selectable for 1, 2 and 3 cm per minute.<br>The time and date must be printed by the recorder at least every 10 minutes.<br>Recording time must be at least 24 hours at 1 cm per minute.<br>The unit must have a facility for remote event marking.

Accessories	Ultrasound and toco transducers and connecting cables.<br>Mains cable.<br>Transducer holders must be provided.<br>Cart/trolley.
Warranties	2 years.
Instructions to Suppliers	The unit must be handed over in full operating orderA starter-pack of consumables must be supplied with the unit.<br>List ALL available consumables with prices incl VAT.<br>List ALL available accessories with prices incl VAT.<br>On-site training to be given.<br>Specify testing procedures and frequency of maintenance.
Note to Procurement	Purchase with a 5-year maintenance contract.

Catalogue Number	Item Name	Available on Transversal Contract	Contract Number or Code
PHC-E-117	***Diagnostic set: portable***	RT 252	RT 252-01-051 ME

Functional Specifications	Otoscope and ophthalmoscope, with battery handle and throat examination head, in a carry case.<br>Handle based Rheostat for light intensity adjustment.<br>Set of 4 re-usable plastic specula for the otoscope.<br>The Otoscope to be supplied with four re-usable polypropylene specula sizes 2.5, 3, 4 and 5mm. The Otoscope head is to be supplied with a positive-lock connection to the handle.<br>The Otoscope is to utilise fibre optic cool-light supply with no reflections. The Otoscope is to have a Wide-angled viewing lens for instrumentation whilst under magnification. The unit is to be completely sealed for Pneumatic otoscopy (i.e. not open operating model). Light LED (optimized for best tissue viewing).<br>Standard Ophthalmoscope head to fit onto the same handle. The Ophthalmoscope head is to cover 25 to 40 Diopters. Illuminated lens dial with clear indication of settings. Red-free filter. Very small, small, large, target and slit apertures. Sealed optics for dust protection. The Ophthalmoscope head is to be supplied with a positive-lock connection to the handle. Light supply LED (optimized for best tissue viewing).<br>Illuminated tongue depressor holder, to fit same handle. To accept standard disposable wooden tongue depressors.<br>**Batteries**: 2 non-corrosive 1,5 volt cells, AA or C size.
Applicable Standards	ISO 10942:2006 Hand-held Direct Ophthalmoscopes. Proof to be supplied.
Performance Specifications	Must be cleanable and disinfectable with standard solutions.
Accessories	1 Spare Globe for each head, 2 sets of batteries, 4 specula, carry case.
Warranties	Minimum 7 year warranty on otoscope, ophthalmoscope, throat illuminator heads and handle. Longer preferred.
Instructions to Suppliers	Supplier must specify available after-sales support, with relevant pricing.
Note to Procurement	These specifications may differ from those used in RT 252 -01-051 ME.<br>Spare globes for each head to be in series with this item.

Catalogue Number	Item Name	Available on Transversal Contract	Contract Number or Code
PHC-E-118	***Diagnostic set: wall-mounted***	RT 252	RT 252-01-050 ME

Functional Specifications	A wall-mounted unit, combining a wall-mounted cradle, an Otoscope, an ophthalmoscope, and the necessary transformer.<br>The wall unit must be of a durable, easy-to-clean material, such as aluminium or high-impact ABS.<br>The unit must have cradles for the other two instruments, and optionally have a mounting point for the aneroid sphygmomanometer.<br>The cradles should have sensors or switches to automatically turn the instruments on when lifted out of their cradles, and switch off when they are placed back in the cradles. Connecting cables between wall unit and instruments can be coiled or straight, but must have positive-lock connections to the instrument heads.<br>Cables must be covered in latex-free rubber or PVC, no woven covering.<br>Handle based Rheostats for light adjustments for both instrument.<br>Cassette for specula not required.<br>The transformer may be integrated into the wall mounting plate, or be external, for direct insertion into a wall socket. In either case, mains connecting cable minimum length 2 meters must be supplied, with a moulded plug to fit standard SABS-approved 15-amp three pin outlets.<br>The transformer must accept 220 volts AC at 50 Hz. Guarantee on all cables and power supplies must be 5 years or better.<br>Otoscope head to fit onto the handle of the wall mounted transformer. The Otoscope to be supplied with four polypropylene specula sizes 2.5, 3, 4 and 5mm (6 of each size, re-usable quality). The Otoscope head is to be supplied with a positive-lock connection to the handle.<br>The Otoscope is to utilize fibre optic cool-light supply with no reflections. The Otoscope is to have a Wide-angled viewing lens for instrumentation whilst under magnification. The unit is to be completely sealed for Pneumatic otoscopy (i.e. not open operating model). Light LED (optimized for best tissue viewing).<br>Standard Ophthalmoscope head to fit onto the second handle of the wall mounted transformer.<br>The Ophthalmoscope head is to cover -25 to 40 Diopters. Illuminated lens dial with clear indication of settings. Red-free filter. Very small, small, large, target and slit apertures. Sealed optics for dust protection. The Ophthalmoscope head is to be supplied with a positive-lock connection to the handle. Light supply LED (optimized for best tissue viewing).
Applicable Standards	ISO 10942:2006 Hand-held Direct Ophthalmoscopes. Proof to be supplied.<br>Supplier must supply certificate issued by Directorate of Radiation Control of the Department of Health, in terms of the Hazardous Substances Act (Act 15 of 1973).
Performance Specifications	Must be cleanable and disinfectable with standard solutions.

Accessories	1 spare globe for each head, 4 re-usable specula. All wall-mounting hardware.
Warranties	Minimum 7 year warranty on otoscope and ophthalmoscope heads, longer preferred. 2 years on cables, 1 year on transformer.
Instructions to Suppliers	Supplier to deliver and install, including electrical connection.<br>Supplier must specify available after-sales support, with relevant pricing.
Note to Procurement	These specifications may differ from those used in RT 252 -01-050 ME.<br>Spare globes to be in series with this item.

Catalogue Number	Item Name	Available on Transversal Contract	Contract Number or Code
PHC-E-119	***Diagnostic combination set: wall-mounted***	No	

Functional Specifications	This unit combines the instruments of PHC-E-119 with an aneroid sphygmomanometer, PHC-E-115.<br>It therefore has the functional specifications of both these units.<br>The unit may additionally have a cassette holder for disposable otoscope tips, but re-usable tips are preferred.
Applicable Standards	ISO 10942:2006 Hand-held direct ophthalmoscopes. Proof to be supplied.<br>Supplier must supply certificate issued by Directorate of Radiation Control of the Department of Health, in terms of the Hazardous Substances Act (Act 15 of 1973).
Performance Specifications	As per PHC-E-115 together with PHC-E-118.<br>Must be cleanable and disinfectable with standard cleaning solutions.
Accessories	1 spare globe for each head, 4 re-usable specula. All wall-mounting hardware.<br>Must be supplied with re-usable Adult, Large Adult and Child cuffs. Sizes to be clearly marked on cuffs. Cuffs may be one-piece or two- piece.<br>Cradle for cuff when not in use.
Warranties	Minimum 7year warranty on otoscope and ophthalmoscope heads, longer preferred. 2 years on cables, 1 year on transformer.<br>At least 2 year guarantee on cuffs **and** covers (including bladders, tubes, outer covers, inflation bulbs, air inlet valves, release valves, filters and cuff fastening mechanism – Velcro or other). Cuffs and covers must be easily cleanable.<br>At least 10 year guarantee on aneroid instrument. At least 10 year calibration guarantee by supplier/ manufacturer. Calibration frequency, procedure and requirements to be clearly stated.<br>Where calibration can be performed by user technicians, any necessary proprietary reference instruments to be offered with pricing.
Instructions to Suppliers	Supplier to deliver and install, including electrical connection.<br>Supplier must specify available after-sales support, with relevant pricing.
Note to Procurement	These specifications may differ from those used in RT 252 -01-050 ME.<br>The Ophthalmoscope head and Otoscope head should be identical to those in PHC-E-118.<br>Ideally, the Sphygmomanometer should be identical to PHC-E-115, with mounting hardware to attach to the wall unit.<br>Spare globes to be in series with this item.<br>Spare cuffs in four sizes and covers, bulbs, tubes, inlet and release valves, must be in series with this instrument.<br>The fact that an instrument is marked CE 0123, is no guarantee of the accuracy or durability of the instrument, or suitability for the South African operating environment.

Catalogue Number	Item Name	Available on Transversal Contract	Contract Number or Code
PHC-E-165	***Electrocardiograph (ECG): 12 channel***	RT 2-2016	42295104-00001

Functional Specifications	ELECTROCARDIOGRAPH (12 CHANNEL) with trolley.<br>Twelve channel portable electrocardiograph.<br>The unit must contain a recorder/printer.<br>The unit must have a colour TFT LCD display of approximately 115 x 85mm.<br>High resolution display: at least 800 x 600.<br>The unit must do a self-test at power-on.<br>The following must be displayed:<br>Heart rate<br>Patient ID<br>12 lead waveforms<br>Lead identification<br>ECG gain<br>Paper speed<br>Filters<br>Electrode connection indicator<br>Messages and prompts<br>Clock with date and time<br>Battery power status<br>R-R analysis for 30 to 300 sec<br>Pacemaker detection.<br>The display screen must be capable of displaying two lines of text and a minimum of three ECG waveforms.<br>The unit must be supplied with a 10 lead ECG cable and be able to record 3, 6 and 12 lead configurations.<br>12 lead configuration:<br>Lead I, Lead II, Lead III<br>AVR, AVL, AVF<br>V1, V2, V3, V4, V5, V6.<br>Manual and automatic modes.<br>The unit must be capable of ECG interpretation for paediatric to adult.<br>Full alphanumeric keyboard or touchscreen.<br>The unit must have facility for entering patient data and text.<br>The unit must offer a minimum of six pre-set ECG programs.<br>Unit must be fitted with a selector switch for QRS sound.<br>Event markers.<br>Automatic indication of disconnected leads/electrodes.<br>Automatic check for incorrect lead placement.

	Full screen print preview of ECG waveforms prior to printing.<br>Heart rate recognition: peak-to peak detection. 30-300 bpm, accuracy 1 bpm.<br>Electrode connection quality check.<br>Printer.<br>Automatic and manual printout mode.<br>In addition to the required ECG waveforms the printer must be able to print:<br>device settings<br>heart rate<br>date and time<br>lead identification<br>calibration pulse<br>patient data.<br>The ECG printout must be available on A4 size, Z fold paper.<br>Printer quality: 8 dots/mm (vertical), 20 dots/mm (horizontal 25mm/s).<br>Paper speed must be selectable as follows: 5, 25 and 50mm/s.<br>Internal memory capable of storing at least 200 patient files.<br>Facility for transmitting this stored ECG to an external device if and when required.<br>External storage by means of SD card (or other) for 2 000 patient files.<br>Internal protection against damage from 360 J defibrillator discharge.<br>The unit must have an internal battery with integrated charger.<br>Battery capacity: at least 2 hours of continuous recording.<br>The unit must be fully operable from mains power regardless of the internal battery condition.<br>Mains cable at least three metres long with SABS approved 15A three prong plug.
Applicable Standards	Must conform to the following standards:<br>I EN 60601-1:1990 +A1: 1993 + A2:1995 (General requirements for safety)<br>I EN 60601-1-2:2007 (Electromagnetic compatibility)<br>I EN 60601-2-25:1995 + A1:1999 (Particular requirements for safety of ECGs)<br>I EN 60601-2-51:2003 (Particular requirements for safety).

Performance Specifications	ECG amplifier specs.<br>Simultaneous acquisition of 12 leads.<br>Frequency response: 0.05-150 Hz.<br>Sensitivity: Automatic or selectable: 2.5-20mm/mV.<br>Automatic amplitude adjustment in the range of 2.5 to 40mm/mV.<br>Sensitivity threshold: 20 µV.<br>Input impedance: > 50 MΩ.<br>Patient leakage current: < 10 µA.<br>Calibration pulse: 1 mV.<br>Filters:<br>50 Hz<br>Low pass: 25, 35, 75, 100, 150 Hz.<br>CMRR: > 100 dB.<br>Sampling frequency: 1 kHz.<br>Artefact rejection.<br>Baseline stabiliser.
Accessories	A sturdy trolley to house the ECG machine and accessories must be supplied.<br>Trolley must have at least one drawer.<br>Trolley must have at least four castors, two with brakes.<br>A starter-pack of consumables must be supplied with the unit.<br>The following accessories must be supplied with the unit:<br>ECG cable with adaptor clips<br>Operator's manual<br>ECG electrodes: disposable, self-adhesive, for resting ECG PHC-C-212.
Warranties	2 years.<br>5- year maintenance contract.
Instructions to Suppliers	The unit must be handed over in full operating order.<br>List ALL available consumables with prices incl. VAT.<br>List ALL available accessories with prices incl. VAT.
Note to Procurement	These specifications were unable to discriminate between seven machines, the most expensive of which cost almost 3 times as much as the cheapest. Need to be improved with respect to quality.

Catalogue Number	Item Name	Available on Transversal Contract	Contract Number or Code
PHC-E-120	**Patella hammer**	No	

Functional Specifications	Queen's Square Pattern, Babinski pattern also acceptable. Head is disc-shaped, stainless steel or chrome-plated brass, with doughnut-shaped rubber bumper. Non-hardening rubber bumper.<br>Handle is white nylon, threaded at one end to screw into the head, pointed at the other end to test for the Babinski reflex.<br>Handle length approximately 45cm.<br>Nylon must be flexible, guaranteed non-hardening and shatterproof for 10 years.
Applicable Standards	Expected service life 15 years.
Performance Specifications	Must be cleanable and disinfectable with standard cleaning solutions.
Accessories	
Warranties	10 years on handle and rubber bumper.
Instructions to Suppliers	
Note to Procurement	This specification differs from that of RT 252-01-163 ME, which is for the Dejerine Pattern Percussion Hammer. The Queens Square pattern is more commonly used.

Catalogue Number	Item Name	Available on Transversal Contract	Contract Number or Code
PHC-E-267	***Peak flow meter***	No	

Functional Specifications	Simple aerodynamic, non-electrical device.<br>One-way valve in mouth-piece to prevent inhalation cross-infection.<br>Easy-to-read scale.<br>Professional-grade instrument suitable for multi-patient screening.
Applicable Standards	ISO 23747:2015.
Performance Specifications	Sterilisable.<br>Measure flow from approximately 50 to 900 l/minute.<br>Repeated readings must show <5% discrepancy.<br>Accuracy must be within 10% of a calibrated spirometer.
Accessories	Disposable mouthpiece PHC-C-341.
Warranties	2 years.
Instructions to Suppliers	Describe sterilisation method.
Note to Procurement	PHC-C-341 must fit the procured item.

Catalogue Number	Item Name	Available on Transversal Contract	Contract Number or Code
PHC-E-139	***Pulse oximeter: stand-alone***	RT 4	RT 4-05-055 ME

Functional Specifications	Portable battery-operated hand-held robust pulse oximeter.<br>Visual display must be brightly illuminated for low-light viewing.<br>Display O_2 saturation, pulse rate, plethysmographic wave form.
Applicable Standards	SANS 80601-2-61:2014.
Performance Specifications	Oxygen saturation range 0-100%.<br>Pulse range 20-300 beats per minute.<br>Accuracy.<br>Operating temperature 0-50°C.<br>Must be cleanable with standard cleaning and disinfectant solutions.<br>Expected service life: 5 years.
Accessories	Must include one adult and one paediatric re-usable probes.
Warranties	2 years.
Instructions to Suppliers	Indicate whether re-calibration is necessary. If it is, indicate frequency, method and quote cost.
Note to Procurement	Replacement re-usable adult and paediatric probes must be in series with this item.

Catalogue Number	Item Name	Available on Transversal Contract	Contract Number or Code
PHC-E-122	***Scale: adult, with height board***	RT 24	RT 24-08-005

Functional Specifications	Scale, with large dial.<br>Comply with the specifications on Appendix H, Type 2, see attached.<br>Capacity: at least 150kg, dial to be at least 150mm in diameter.<br>Must include height measuring rod.<br>Mounted on castors, roller size: 30-50mm.<br>Fixed direction, scale to be able to tilt for moving.
Applicable Standards	SANS 1649:2014.
Performance Specifications	Weighing capacity up to 150kg.<br>Expected service life: 10 years.<br>Accuracy: within 1gm per 1000gm.<br>Repeatability: Standard deviation less than 10gm after 20 measurements at 50kg.
Accessories	
Warranties	2 years.
Instructions to Suppliers	Specify method, frequency and cost of re-calibration.
Note to Procurement	Future procurement to increase capacity to at least 180Kg, and specify accuracy requirements.

Catalogue Number	Item Name	Available on Transversal Contract	Contract Number or Code
PHC-E-123	***Scale: adult, without height board***	RT 24	RT 24-08-003

Functional Specifications	Scale, analog/mechanical, bathroom type, weighing capacity: 0 – 150 kg at least. Large dial display. Spring lever mechanism with vanadium steel spring. Heavy, hardened steel pivots and bearing.<br>Scale must be of robust construction.
Applicable Standards	SANS 1649:2014.
Performance Specifications	Weighing capacity up to 150kg.<br>Accuracy: 0.1% throughout measurement range.<br>Expected service life: 15 years.<br>Accuracy: within 10gm per 1000gm.<br>Repeatability: Standard deviation less than 10gm after 20 measurements at 50kg.
Accessories	
Warranties	2 years.
Instructions to Suppliers	None.
Note to Procurement	Future procurement to increase capacity to at least 180kg, and specify accuracy requirements.

Catalogue Number	Item Name	Available on Transversal Contract	Contract Number or Code
PHC-E-124	***Scale: baby***	RT 24	RT 24-08-001

Functional Specifications	Scale, weighing electronic, baby and child convertible. Battery operated.<br>Automatic switch off.<br>Increments: 10g up to 10kg, 20g > 10kg LCD display: digits at least 20mm high. Electronic damper.<br>Hold feature to retain weight reading. Function to display breast milk intake.
Applicable Standards	None available.
Performance Specifications	Weighing capacity up to 20kg.<br>Must be accurate within 10gm at 20kg.<br>Repeatability: Standard deviation less than 10gm after 20 measurements at 10kg.<br>Expected service life 10 years.
Accessories	Set of batteries.
Warranties	2 years.
Instructions to Suppliers	None.
Note to Procurement	None.

Catalogue Number	Item Name	Available on Transversal Contract	Contract Number or Code
PHC-E-125	***Stadiometer***	RT 24	RT 24-08-009

Functional Specifications	Stadiometer (Height board).<br>To measure height of children and adults while standing.<br>Range: 20-125cm, graduated in m, cm and mm, with numbers at metre and cm positions.<br>Measuring surface must be free of all moving parts that may affect the markings.<br>Movable headboard must slide easily on the vertical section and lock in the measurement position.<br>Headboard must lock at 90° to the vertical measuring section.<br>Locking mechanism must not affect the measuring surface.<br>Sturdy and stable footplate. Must allow the heels, buttocks and back of the head to make contact with the vertical surface during measurement. Must be made of durable, non-flexible, smooth material that is water resistant, easy to clean, with no sharp edges or unfinished parts.
Applicable Standards	None.
Performance Specifications	Must be accurate to at least 0,5mm per metre.<br>Expected service life 15 years.
Accessories	None.
Warranties	2 years on all components.
Instructions to Suppliers	None.
Note to Procurement	None.

Catalogue Number	Item Name	Available on Transversal Contract	Contract Number or Code
PHC-E-126	***Stethoscope: dual head***	No	

Functional Specifications	Stethoscope, binaural twin-head. Head to be one-piece stainless steel or medical-grade aluminium, with air-tight seal between head and shaft. Non-removable bell and non-corrugated diaphragm.<br>Bell to be of shallow type, with replaceable non-chill sleeve. Diaphragm to be of good quality reinforced plastic. Rotation mechanism must have positive click-stop in both bell and diaphragm position, such that head does not rotate during auscultation.<br>Tubing must be of sufficient thickness to attenuate environmental noise. Latex-free material. Single tube (although may be double-lumen). Longer tube length preferred (minimum 70cm from earpiece to head). Ear tubes must be adjustable (i.e. rotatable) or angled forward at 15 degrees.<br>Tubes must be of stainless steel with screw-on earpieces. Junction between tubes and earpieces must be airtight. Headpiece spring may be internal (preferred) or external, stainless steel or chrome-plated spring steel. Earpieces should be soft, formfitting, but standard hard plastic earpieces must also be supplied.
Applicable Standards	None available.
Performance Specifications	Head must amplify low frequency <100hz by approximately 15db, attenuate higher frequencies >500hz. **Performance certificate required.**<br>Entire unit must be disinfectable with standard solutions.<br>Expected service life: 15 years.
Accessories	Unit to be supplied complete with spare diaphragm and earpieces.
Warranties	Minimum 5 year guarantee on all components, including rotation of head and headpiece spring (at least 100 000 rotations and flexions).
Instructions to Suppliers	An optional model with paediatric head to be proposed.<br>Specify any cleaning materials not compatible with the unit.
Note to Procurement	

Catalogue Number	Item Name	Available on Transversal Contract	Contract Number or Code
PHC-E-127	***Syringe: Ear***	No	

Functional Specifications	Stainless steel cylinder and plunger. Finger rings or half-rings for one-handed control of irrigation.
Applicable Standards	None available.
Performance Specifications	Must be cleanable and disinfectable with standard solutions.<br>Envisaged service life minimum 15 years.
Accessories	Re-usable silicon rubber tips.
Warranties	Minimum 2 years.
Instructions to Suppliers	
Note to Procurement	

Catalogue Number	Item Name	Available on Transversal Contract	Contract Number or Code
PHC-E-130	***Thermometer: digital, ear-type***	No	

Functional Specifications	Ear type thermometer.<br>Hand-held, battery operated, batteries in handle. Batteries may be conventional alkaline or rechargeable (if the latter, price must include charger) Probe tip and sensor. Probe cover detection system. Automatic probe cover eject button. Easy-to-read, LCD displays temperatures in degrees Celsius.
Applicable Standards	None available.
Performance Specifications	Accuracy within 0.1°C.<br>Must be cleanable with standard cleaning and disinfectant solutions.<br>Expected service life: 10 years.
Accessories	None.
Warranties	2-year guarantee.
Instructions to Suppliers	Supplier to provide pricing of probe covers and any other spares/consumables. Supplier to indicate expected length of operation on one set of batteries or one charge. If rechargeable, state expected battery life under normal operation.<br>State expected Mean Time Before Failure of the unit.
Note to Procurement	Disposable probe covers PHC-C-178 to be in series with this item.

Catalogue Number	Item Name	Available on Transversal Contract	Contract Number or Code
PHC-E-131	***Thermometer, digital pen type***	No	

Functional Specifications	Pen-type thermometer.<br>Electronic clinical thermometer, with easily visible digital display in degrees Celsius. Rapid measuring time, less than 10 secs. Auto-switch-off. Waterproof, impact-resistant, disinfectable. Replaceable battery.
Applicable Standards	None available.
Performance Specifications	Accuracy within 0.1°C.<br>Must be cleanable with standard cleaning and disinfectant solutions.
Accessories	None.
Warranties	2-year guarantee.
Instructions to Suppliers	If replaceable sheaths are available, supplier to supply pricing.
Note to Procurement	If replaceable sheaths are available, to be in series with this item.

Catalogue Number	Item Name	Available on Transversal Contract	Contract Number or Code
PHC-E-121	***Torch: pupillary***	No	

Functional Specifications	One or two-cell pupil examination penlight torch.<br>Pushbutton latching on-off switch. No sliding or rotary switch, no push-to-hold switch.<br>Single super-bright white LED light source (no multi-LED types).<br>Non-slip body, preferably splash-proof.<br>Must be cleanable and disinfectable with standard cleaning solutions.
Applicable Standards	None.
Performance Specifications	Must have a uniform beam of light at up to 50cm – no dark centre spot with bright outer ring.<br>Must withstand a 1m drop onto a concrete surface, without malfunction.<br>Expected service life 10 years.
Accessories	Two sets of non-corrosive batteries.
Warranties	Warranted free of manufacturing defects for a 6-month period.
Instructions to Suppliers	None.
Note to Procurement	None.

Catalogue Number	Item Name	Available on Transversal Contract	Contract Number or Code
PHC-E-132	***Tuning fork set***	No	

Functional Specifications	Tuning fork to be manufactured from medical grade stainless steel. Attached flat base for vibration and bone conduction tests. Models without weights on tines preferred. Set of one each 128Hz and 256Hz .
Applicable Standards	None available.
Performance Specifications	Each fork to be clearly marked with vibration frequency.<br>Accurate to 0,2 HZ.<br>Expected service life: 20 years.
Accessories	None.
Warranties	None.
Instructions to Suppliers	
Note to Procurement	

Essential

Catalogue Number	Item Name	Available on Transversal Contract	Contract Number or Code
PHC-E-111	***Examination light: clinical. Ceiling-mounted, directional***	RT 2	41111931-00002

Functional Specifications	Ceiling-mounted examination light for general medical examination. LED light source. Easy switching method. Self-balancing mechanism (spring or friction clutch based), so that the light head holds its position wherever directed. To have sufficient swing radius to cover both ends of standard examination couch, from a mounting point on ceiling at mid-length of the couch.<br>Adequate length of suitable approved electrical cable, to permit connection to in-ceiling power outlet.
Applicable Standards	IEC 60601-1, IEC 60601-1- 2 and IEC 60601-2-41.
Performance Specifications	Daylight colour temperature of light source (~6000K)).<br>Light intensity at least 35 000 lux at 60cm.<br>Must be cleanable and disinfectable with standard solutions.
Accessories	To be supplied with wall mounting hardware.
Warranties	Minimum 5 year Guarantee on all parts including light source.
Instructions to Suppliers	Supplier must specify power connection type and any structural mounting (i.e. load-bearing) requirements.<br>Supplier must specify available after-sales support, with relevant pricing.<br>Supplier must supply certificate issued by Directorate of Radiation Control of the Department of Health, in terms of the Hazardous Substances Act (Act 15 of 1973).
Note to Procurement	Spare LED bulbs to be in series with this item.

Catalogue Number	Item Name	Available on Transversal Contract	Contract Number or Code
PHC-E-098	***Examination light: headlamp***	No	

Functional Specifications	Lightweight head-worn LED light.<br>Adjustable headband, with over-the-head strap to prevent slippage.<br>Tilt-adjustable. CREE LED element. Daylight colour temperature preferred.<br>Easy access on-off switch on lamp. Easy access focus ring on lamp.<br>Rechargeable Lithium-ion batteries – on headband or belt-worn.<br>Should also accept equivalent size of alkaline cells in emergency.<br>Intermittent flash, different power modes not required.<br>Splashproof, at least Ingress Protection 44 rating.
Applicable Standards	
Performance Specifications	Max weight of headgear less than 180gm.<br>Minimum light output 200 lumens.<br>Operating time at least 5 hours on full output.<br>Focusable from narrow to wide beam.<br>Beam must project light uniformly at 1 metre – no rings or dark spots.<br>Must be cleanable and disinfectable.
Accessories	Charger, all cables. Complete with Li-Ion batteries.
Warranties	1 year.
Instructions to Suppliers	
Note to Procurement	Spare LED bulbs to be in series with this item.

Catalogue Number	Item Name	Available on Transversal Contract	Contract Number or Code
PHC-E-154	***Examination light: mobile, without battery***	RT 2-2016	41111931-00004

Functional Specifications	Mobile examination light for general medical examination. LED light source. Easy switching method. Self-balancing mechanism (spring- or friction clutch-based), so that the light head holds its position wherever directed. To have sufficient swing radius to cover both ends of standard examination couch, from a standing point on floor at mid-length of the couch.<br>Unit mounted on tilt-resistant base, with castors.<br>Epoxy-coated steel construction.<br>Power cord to be fixed to light, 3meter length with moulded SABS approved 15A 3-pin plug.
Applicable Standards	IEC 60601-1, IEC 60601-1- 2 and IEC 60601-2-41.<br>SANS 778 paragraph 5.2, SANS 621.
Performance Specifications	Daylight colour temperature of light source (6000 K).<br>Light intensity at least 35 000 lux at 60cm.<br>Must be cleanable and disinfectable with standard solutions.
Accessories	
Warranties	Minimum 5yr Guarantee on all parts including light source.
Instructions to Suppliers	Supplier must specify available after-sales support, with relevant pricing.<br>Supplier must supply certificate issued by Directorate of Radiation Control of the Department of Health, in terms of the Hazardous Substances Act (Act 15 of 1973).
Note to Procurement	Spare LED bulbs to be in series with this item.

Catalogue Number	Item Name	Available on Transversal Contract	Contract Number or Code
PHC-E-110	***Examination light: clinical, wall-mounted, directional***	RT 2	41111931-00005

Functional Specifications	Wall-mounted examination light for general medical examination. LED light source. Easy switching method. Self-balancing mechanism (spring or friction clutch-based), so that the light head holds its position wherever directed. To have sufficient swing radius to cover both ends of standard examination couch, from a mounting point on wall at mid-length of the couch.
Applicable Standards	IEC 60601-1, IEC 60601-1- 2 and IEC 60601-2-41.
Performance Specifications	Daylight colour temperature of light source (~6000K).<br>Light intensity at least 35 000 lux at 60cm.<br>Power cord to be fixed to light, 2m length with moulded SABS approved 15A 3-pin plug.<br>Must be cleanable and disinfectable with standard solutions.
Accessories	To be supplied with wall mounting hardware.
Warranties	Minimum 5years on all parts including light source.
Instructions to Suppliers	Supplier must specify available after-sales support, with relevant pricing.<br>Supplier must supply certificate issued by Directorate of Radiation Control of the Department of Health, in terms of the Hazardous Substances Act (Act 15 of 1973).<br>Supplier to specify ceiling load-bearing and fixing requirements.
Note to Procurement	Spare LED bulbs to be in series with this item.

Catalogue Number	Item Name	Available on Transversal Contract	Contract Number or Code
PHC-E-113	***Tape measure: clinical***	No	

Functional Specifications	Flat tailor-type tape measure, in durable flexible plastic or fiberglass.<br>Durable, non-fade, non-smudge, high-contrast markings in millimetres and centimetres.<br>The ends of the tape may be reinforced with plastic or metal strips.
Applicable Standards	None.
Performance Specifications	Non-stretch tape.<br>Length must be at least 150cm.<br>Must be cleanable with standard cleaning and disinfectant solutions.
Accessories	
Warranties	None.
Instructions to Suppliers	None.
Note to Procurement	Before finalising procurement, test tape for accuracy by comparing to a certified steel 1m ruler, and then applying tension to the tape to check for non-stretchability.

Catalogue Number	Item Name	Available on Transversal Contract	Contract Number or Code
PHC-E-151	***Bedpan***	RT 24	RT 24-03-001

Functional Specifications	For urine collection from female patients.<br>Standard Oval or Triangular contour bedpan.<br>Stainless steel, one-piece body.<br>Polished, smooth contours, no sharp edges.
Applicable Standards	
Performance Specifications	Must be sufficiently robust to support 180kg weight across the top of the unit (while resting on a solid surface), without distorting.<br>Must be autoclaveable.
Accessories	
Warranties	10 years.
Instructions to Suppliers	
Note to Procurement	These specifications differ from those in RT 24, which are for a slipper-shaped plastic bedpan. The latter is more suitable for bed-ridden patients. The model described here is more suitable for ambulant female patients.<br>When procurement according to these specifications commences, a 6-unit bedpan stand, to be mounted in the sluice-room, should also be procured. Stand must comply with CKS 217:2010.

Catalogue Number	Item Name	Available on Transversal Contract	Contract Number or Code
PHC-E-158	***Urine jar or flask***	RT 24	RT 24-01-010

Functional Specifications	Stainless Steel jar or flask, with broad base to resist tipping. Inverted cone design ideal.<br>With or without hinged lid.
Applicable Standards	
Performance Specifications	At least 600ml.<br>Must be autoclaveable.
Accessories	
Warranties	10 years.
Instructions to Suppliers	
Note to Procurement	These specifications differ from those in RT 24, which are for a plastic measuring jug.<br>These specifications should be used in future procurements.

Specialised

RESUSCITATION

Catalogue Number	Item Name	Available on Transversal Contract	Contract Number or Code
PHC-E-133	***Automated external defibrillator (AED)***	RT 4	RT 4-05-072-A-ME

Functional Specifications	The unit being offered must be a button operation Biphasic semi-automated external defibrillator and it must be suitable for ground mobile, ambulance use and ward use in hospitals.<br>It must be robustly constructed.<br>It must incorporate the latest and reliable Biphasic technology.<br>The unit offered must be able to perform impedance compensation before delivering a shock to a patient.<br>The supplier must state the minimum and maximum defibrillator energy charge that is available on the unit being offered for when unit is in manual mode.<br>The unit must provide clearly visible screen text messages and clear audio voice prompts in the english language to guide the user through the whole operational of the unit.<br>The visible screen text messages must be clearly visible under all lighting conditions.<br>The supplier must state the total weight of the unit including the battery.<br>The unit offered must function off a reliable rechargeable battery and must include the charger with a reconditioning button in the final price.<br>Must be provided with additional set of backup batteries to ensure continuous operation.<br>The unit must offer automated self-checks and manual check must be carried out every time the machine is switched on as well as weekly and monthly, and all the data of the self-check must be stored in the internal memory.<br>The weekly and monthly automated self-check must include at least the following: battery, internal electronic circuitry and energy charges.<br>The unit must be fitted with an audible alarm if any of self-tests or battery fails as well as a LCD screen that will indicate a failure of the device. The unit must have a minimum of 10 audible and 10 visual screen prompts.<br>The length of the pad connecting cable must be at least 0.8 meters.<br>The unit must be supplied with a patient cable that will be able to be connected to ECG electrodes for monitoring without connecting the pads.<br>The ECG Waveform screen must be a backlit LCD screen with a size of at least 6cm x 4cm or 6cm diagonally.<br>The unit must be supplied with a screen protector and or protective device.<br>The unit must be able to be configured for paediatric patients. Paediatric adaptor, pads/all paddles should be included.<br>The unit must have a manual override with password protection.<br>The unit must be able to download information to a computer for legal purposes.<br>The supplier must supply a set of disposable defibrillator pads, which includes the connecting cable and the cost of this set of defibrillator pads must be included in the final bid price.<br>The disposable defibrillator pads for the unit offered must have a shelf life of at least 2 years.<br>A carry case constructed of durable waterproof material, which has should straps must be supplied and included in the final bid price.<br>The carry case offered must adequately protect the unit when used by ambulance and emergency personnel in poor and wet weather conditions.

Applicable Standards	The unit must conform to IEC601-2-4.<br>"Particular requirements for the safety of cardiac defibrillators".<br>The unit shall comply with the necessary safety standards.<br>The unit must be able to withstand vibration and shock as per Mil Std 810E or Equivalent.<br>The supplier must provide details.<br>The unit offered must be water resistant to a rating of at least IEC 529 IPX4. Supplier must provide details.<br>The unit must be aviation certified.
Performance Specifications	The unit offered must be lightweight less than 4kg.<br>The rechargeable batteries must have a capacity of a minimum of 50 x 150J shocks or at least 2 hours of monitoring time.<br>The rechargeable batteries must have a charge time of less than 3 hours.<br>The unit must be able to withstand droppage of at least 1m on any corner. Supplier must provide details.<br>To be splash proof.<br>The unit must be equipped with internal memory of a minimum of 1GB.<br>The unit must be equipped with a serial port, USB or data card system for download purposes.<br>The software to download must be included in the bid price and supplied with every AED delivered by the successful supplier.<br>Expected service life: at least 8 years.
Accessories	All accessories will be supplied with the unit at no additional cost.<br>The unit must be supplied with two rechargeable batteries and the charger unit.
Warranties	Minimum mandatory guarantee is 2 years. 5 years preferred.<br>The unit must be offered with a minimum of a 2 year full unconditional replacement operating battery guarantee.<br>The disposable defibrillator pads for the unit offered must have a shelf life of at least 2 years.

Instructions to Suppliers	Consumables should be quoted for separate sourcing as after-sales services.<br>Supplier to indicate which consumables and non-consumables items is required for the normal operation and standard maintenance of the equipment during the guarantee period.<br>The equipment may be maintained by the purchaser after the guarantee period has expired, therefore: The supplier must provide detailed preventative maintenance and calibration procedures. The supplier must provide technical training in the theory of operation, fault finding and calibration.<br>Manuals must be comprehensive, including circuit diagrams in case of electronic/electrical equipment, enabling resident technical staff to deliver complete technical support in case of equipment failure, as well as routine servicing.<br>Manuals will be treated as confidential and for the sole use on equipment owned by the purchasing department.<br>The supply of workshop/service manuals is a mandatory requirement of this bid and they must be in accordance with the requirements laid down above.<br>The complete unit must include each replaceable accessory as specified in a series at no additional cost. The cost must be included in the final bid price.<br>The supplier must undertake to provide a comprehensive training schedule when required, for end-user departments and clinical engineering staff of the end users department to ensure:<br>Correct use of equipment<br>Comprehensive technical support capability of the equipment, of at least 2nd level by eligible resident clinical engineering staff.<br>Training of users shall be provided at no extra cost.
Note to Procurement	This equipment is part of a series, from RT 4-05-072-A-ME to RT 4-05-076-A-ME, inclusive. Any replacement should have equivalent serialisation. Replacement pads RT 4-05-105 ME and RT 4-05-106 ME.

Catalogue Number	Item Name	Available on Transversal Contract	Contract Number or Code
PHC-E-134	***Cardiac arrest board***	No	

Functional Specifications	Rigid, smooth board with rounded corners and smooth edges, to be inserted under the thorax of an unconscious patient.<br>The board must provide a firm base against which to perform chest compression.<br>For ease of insertion and removal, must have no rough fabric-snagging details.<br>Cut-out hand-holds.<br>Size approximately 60x40cm. Thickness minimum 10mm.<br>Material: polypropylene, ABS or quality varnished plywood.
Applicable Standards	None.
Performance Specifications	Must be cleanable with standard cleaning and disinfectant solutions.
Accessories	None.
Warranties	
Instructions to Suppliers	None.
Note to Procurement	Usually supplied with Emergency Trolley RT 24-12-01 but can be ordered as replacement or addition.

Catalogue Number	Item Name	Available on Transversal Contract	Contract Number or Code
PHC-E-190	***Infant warmer: radiant, mobile***	RT2-2016	42191811-00001

Functional Specifications	The unit must offer long-term care of a neonate.<br>PLATFORM AND MATTRESS:<br>Adjustable tilt to Trendelenburg and reverse Trendelenburg positions<br>Locking mechanism to keep the mattress platform locked in all positions<br>The mattress must be waterproof and must be X-ray transparent<br>Mattress size: approximately 500 x 650 mm<br>Mattress thickness: at least 25 mm<br>Mattress must support patient weight up to 4,5 kg<br>All four sides of the platform must be fitted with transparent guards/panels<br>The front and two side panels must be hinged.<br>HEATER<br>Radiant heater housed in an overhead canopy, with internal reflector, mounted on the rear mainframe.<br>Indicator to show heater is switched ON.<br>The overhead canopy must swivel left and right and must lock in the desired position.<br>CONTROLS<br>Heat control: adjustable heater power from 0 - 100%.<br>If a malfunction occurs affecting safety or performance the heater must be automatically turned off.<br>Skin temperature probe to monitor infant temperature.<br>Heat output must be controlled manually or through baby mode (feedback mode) for thermoregulation.<br>DISPLAYS<br>Heater power.<br>Alarms.<br>Visual and audio alarms must be present for safety.<br>Apgar timer, with audible alarm, with digital display: 1, 5 and 10 minutes elapsed time.<br>Apgar timer with on/off switch and reset button.<br>OTHER<br>The overhead canopy must be provided with an examination lamp, with on/off switch.<br>The infant radiant warmer must have a drawer.<br>The minimum acceptable height of the heater canopy must be 1.85 metres above floor.<br>Adjustable mattress platform height with a minimum of 900 mm.<br>The mains cable of the unit must be at least three metres long and be fitted with an SABS approved 15A three pin plug.<br>The unit must be mobile, but stable during movement.<br>4 swivel castors of minimum diameter 100 mm.<br>Two of the castors must be lockable.

Applicable Standards	SANS/ANSI/AAMI/IEC 60601-2-21:2009 (Particular Requirements for the Safety of Infant Radiant Warmers). The relevant certification from the manufacturer must be supplied.
Performance Specifications	Must maintain temperature within 0,5°C of setting.<br>Automatic shut-off if temperature exceeds maximum setting, until temperature drops back to within required range.<br>Temperature sensitivity <0,2C.<br>Antibacterial mattress cover.
Accessories	Complete with re-usable temperature probe, mattress, power cable.<br>A starter-pack of consumables must be supplied with the unit.
Warranties	2 years.
Instructions to Suppliers	To be supplied with 5-year maintenance contract.<br>Specify maintenance routines and service intervals.<br>The unit must be handed over in full operating order.<br>List ALL available consumables with prices incl. VAT.<br>List ALL available accessories with prices incl. VAT.<br>Optional Items, quote prices incl. VAT.<br>LED Phototherapy.<br>The phototherapy light must be either built into the overhead heater canopy or it must be a separate dedicated module that mounts onto the side of the overhead heater canopy.<br>The phototherapy light must work off the 220 Volt, 50 Hz single-phase supply.<br>The phototherapy light must produce UV light with a wavelength between 450 and 470nm.<br>The phototherapy light must have an intensity >30uW/cm^2/µm.<br>IV pole.<br>Disposable temperature probe.<br>Accessory rail.<br>Monitor stand.<br>Oxygen cylinder holder.
Note to Procurement	Note that there has been a split award. Any spares must be compatible with chosen model.

Catalogue Number	Item Name	Available on Transversal Contract	Contract Number or Code
PHC-E-135	***Laryngoscope set for PHC facilities***	No	

Functional Specifications	Laryngoscope set handle: Handle fabricated from stainless steel. The bulb is housed in the handle head. The lamp to be of LED type. Lamp to switch on when blade is extended into operating position. Quick-release head, Batteries to be housed in handle. May have second smaller handle for paediatric blades. Spare set of batteries to be supplied.<br>Blades to be supplied: The blade sizes 0, 1, straight fibre optic (Miller), and 1,2,3,4,5 curved fibre-optic(Macintosh pattern). Blades are to be manufactured from medical grade stainless steel. With hook-on fitting and positive-locking engagement to keep blade attached to handle in closed or operating position. Integrated, non-removable light path.
Applicable Standards	Must comply with ISO 7376:2009 (or later) or equivalent.
Performance Specifications	Cool white or daylight LED colour temperature.<br>Handle must have non-slip surface or knurling.<br>Expected service life: 15 years.
Accessories	To be supplied in a plastic container with closure. Spare bulb to be supplied, with instructions for replacement.<br>Spare set of batteries.
Warranties	5-year guarantee on handles, blades, hook-on mechanism and switches. At least 2-year guarantee on light source.
Instructions to Suppliers	Supplier to provide a list of accessories and parts for the laryngoscope, with pricing. Supplier to include a price for an optional. Paediatric handle, if not included with the set. Supplier is also to offer a sheet of high-density closed-cell foam with cut-outs for the handle, blades and spare bulbs, to be fitted inside a drawer of the crash cart trolley.
Note to Procurement	There is a similar set available as RT 4-05-040 ME, but it does not have the blade selection required. This shortcoming must be addressed in the next tender.

Catalogue Number	Item Name	Available on Transversal Contract	Contract Number or Code
PHC-E-136	***Monitor: multifunction vital signs, including SpO_2***	RT 4-2015	RT 4-05-030 ME

Functional Specifications	The unit will automatically acquire and display vital measurements once switched on and connected to the patient. Data acquisition will continue at pre-set intervals, until switched off. A transport-type unit is envisaged.<br>Given the operational environment, the smallest number of controls is preferred.<br>Although not mandatory, external communication port would be an advantage – could be optional (USB or RJ).<br>Power-on self-test.<br>The interval between measurements must be selectable from 1 – 120 minutes.<br>Must be compatible with Automatic External Defibrillator.<br>Unit should have optional module to measure temperature, or alternative model incorporating temperature measurement should be proposed. Temperature in degrees Centigrade, state range.<br>Must be suitable for adults, children and neonates.<br>Reusable cuffs must be supplied: Large adult, adult, paediatric, neonatal. Cuffs and cuff covers must be washable, disinfectable. Minimum cuff tube length 1,5m.<br>The unit must use the oscillometric method of measurement.<br>Modes: Manual/stat and automatic.<br>$Sp0_2$ sensor:<br>Sensor: Dual LED, Infra-red/visible red, which does not heat the point of application to the patient.<br>The following reusable sensors with cable and plug must be provided:<br>Finger sensor for adults<br>Finger sensor for children<br>Earlobe sensor for adults<br>Earlobe sensor for children<br>The sensor lead must be approximately 3 metres long.<br>LCD display:<br>The display must offer clear viewing under all lighting conditions.<br>The following must be displayed:<br>Systolic, diastolic and mean arterial pressures (numeric)<br>Oxygen saturation ($Sp0_2$ %)

Applicable Standards	Measurement accuracy must have been clinically validated (AASI/AAMI SP10-2002, BHS protocol, or ESH protocol). **Proof to be submitted, preferably peer-reviewed published report**. Unpublished independent clinical validation study may be accepted in absence of peer-reviewed published report. Minimum is in-house clinical validation study and report.<br>Evidence of measurement repeatability required (SD<1mm Hg from 30 consecutive measurements of 120/80mm Hg).<br>Must comply with latest version of EN 1060-4, EN 1060-3 with Amendment A1:2005.
Performance Specifications	State whether measurements are performed on inflation or deflation part of cycle.<br>State BP measurement algorithm.<br>Pressure measurements:<br>**Adult**: Systolic: 30 – 270mm Hg, **Diastolic**: 20-240mm Hg, MAP: 20 – 250mm Hg<br>**Paediatric**: Systolic: 30 – 160mm Hg, **Diastolic**: 20-120, MAP: 20 – 120mm Hg<br>**Neonate**: Systolic: 30 – 130mm Hg, **Diastolic**: 20-100mm Hg, MAP: 20 – 120mm Hg<br>**Heart rate**: 30 – 240 min. Indication of pulse irregularity desirable.<br>Unit should have optional module to measure temperature, or alternative model incorporating temperature measurement should be proposed. Temperature in degrees Centigrade, state range.<br>**Pulse oximeter.**<br>State SpO_2 measurement method.<br>Suitable for adult, paediatric and neonatal use.<br>The unit must continuously measure and display SpO_2 % .<br>Oxygen saturation (SpO_2 %): Range of measurement: 0-100%.<br>The accuracy must be better than 2,5 % (70-100%) and 5% (50-70%).<br>Pulse rate (beats/min): Range of measurement: 20-300 bpm.<br>The pulse rate accuracy must be better than ± 2 %.<br>An audible bleep should sound for each heartbeat, the pitch of which should vary with the oxygen saturation.<br>Automatic rejection of motion artefacts.<br>Must be protected against electrosurgical and defibrillation voltages.<br>The unit must have trending for at least 72 hours.<br>Expected service life: 10 years.
Accessories	Must be supplied with all patient cables (incl. at least three-lead ECG cable), SpO_2 sensors, power cable at least 3m long with standard South African 3-pin plug, user manual.
Warranties	3-year guarantee on all parts (including cuffs and cuff covers) except battery. Specify expected battery life under 8 hour continual operations, daily cycling. Guarantee to include calibration.

Instructions to Suppliers	If unit has an optional module for ECG recording (or if there is a separate model which includes this capability, supplier to provide comprehensive technical specifications, pricing, and pricing of any patient cables, consumables and replaceable parts.<br>Supplier to deliver, commission and train users.<br>Supplier to stipulate any costs associated with service over the guarantee period, and whether a service plan is avaialble (specify price).<br>Supplier to stipulate recommended recalibration intervals.<br>Cuffs and covers must be cleanable and disinfectable. Indicate whether sterilisable, preferred method, and number of cycles.<br>Supplier to state Mean Time Before Failure for Unit and any parts which may have different MTBF.<br>Supplier must supply certificate issued by Directorate of Radiation Control of the Department of Health, in terms of the Hazardous Substances Act (Act 15 of 1973).<br>Supplier must specify available after-sales support, with relevant pricing.
Note to Procurement	These specifications exceed those of RT 4-05-030 ME, and should be used for future procurements.<br>The following items must be in series with this instrument:<br>Thigh cuff<br>Large adult cuff<br>Adult cuff<br>Paediatric cuff<br>Neonatal cuff<br>Air hose<br>Battery<br>Finger sensor for adults<br>Finger sensor for children<br>Earlobe sensor for adults<br>Earlobe sensor for children<br>Disposable sensors for adults, pack of ten<br>Disposable sensors for children, pack of ten<br>Sensor for neonates<br>Replaceable main board (if user- serviceable)<br>Temperature sensor if disposable, quote price per 100)<br>Optional wall-mounting bracket<br>Optional pole-mounting fixture<br>Optional mounting to instrument rail (built-in hanging hooks not suitable)<br>Optional bracket to fix unit to a table top<br>Optional mobile stand on 5-star base with non-marking castors (SABS 601)<br>Optional cradle for cuff when not in use (wall/pole/stand).

Catalogue Number	Item Name	Available on Transversal Contract	Contract Number or Code
PHC-E-137	***Nebuliser***	No	

Functional Specifications	**General specifications:** The unit must be a portable, lightweight unit of approximately 2-3kg. Ultrasonic. Should be able to connect directly to a mask. Unit to be cleanable by either boiling (5-10mins) or with a chemical disinfectant or sterilisable in autoclave at 121°C (1,2bar). Unit to include power switch to activate blower and transducer, flow rate adjustment controller. Unit to have external air inlet with replaceable high efficiency filter (50/70um). Unit to have a low operating sound level (max 60dB). Time-out function 0-60 minutes.<br>**Power requirements** Mains voltage: 220V, 50-60Hz. The unit to be supplied complete with 3m power cord.
Applicable Standards	ISO 27427:2013.<br>Supplier must provide certificate issued by Directorate of Radiation Control of the Department of Health, in terms of the Hazardous Substances Act (Act 15 of 1973).
Performance Specifications	Unit to operate with maximum pressure of 1,6 bar approximately.<br>Unit to have a nebulisation capacity of 2-5 ml per minute. Medicine chamber capacity 50-100ml. Particle size 3-8 micron.<br>Must be cleanable with standard cleaning and disinfectant solutions.<br>State expected Mean Time Before Failure of the unit.<br>Expected service life: 10 years.
Accessories	None.
Warranties	3 years.
Instructions to Suppliers	All necessary documentation and operating/service manuals in English to be included in tender price. Supplier to list consumable and replacement items with relevant prices for these.<br>Supplier to indicate availability of after-sales support, with relevant pricing. Details of recommended service and service intervals to be clearly stated.
Note to Procurement	

Catalogue Number	Item Name	Available on Transversal Contract	Contract Number or Code
PHC-E-138	***Oxygen regulator with flow-meter***	No	

Functional Specifications	A medical gas regulator incorporating a flow meter. To incorporate a safety pressure relief valve. Regulator body to be manufactured from non-corrosive metal.<br>The flow meter and flow adjustment knob to be manufactured from an impact resistant plastic (polycarbonate).<br>High visibility flow indicator.<br>Bullnose fitting.<br>Nipple for direct connection of oxygen masks or tubing.
Applicable Standards	None.
Performance Specifications	Flow meter to be graduated from 1 to 15l per minute.<br>Must be cleanable with standard cleaning and disinfectant solutions.<br>Expected service life: 15 years.
Accessories	
Warranties	5 years on needle valve, 10-year guarantee on valve body and flowmeter.
Instructions to Suppliers	None.
Note to Procurement	The bullnose regulator in RT 4 has been discontinued. A replacement needs to be found for use with larger oxygen cylinders.<br>These specifications differ from those for RT 4-05-035 ME, and should be considered for future procurements. The dial-type flowmeters requested in RT 4 have proven to be too fragile for our operating environment.<br>As flowmeters tend to get damaged, spare flowmeters should be in series with this item.

Catalogue Number	Item Name	Available on Transversal Contract	Contract Number or Code
PHC-E-140	***Infant manual resuscitator***	RT 4	RT 4-05-026 ME

Functional Specifications	Usage for premature neonates and infants 0kg to 4kg.<br>Shall allow for single-handed operation.<br>Bag capable of rapid and full re-expansion.<br>Swivel connector present to allow for changing angle of operation.<br>Function not affected by extremes temperatures.<br>Allow for oxygen inlet via oxygen tubing.<br>Face mask shall be clear and provide a leak proof seal. Mask sizes 00,0 and1 to be supplied with each item.<br>Compatible valve fitting to standard external PEEP fitting W/POP-OFF minimum 40cm H20.<br>Non-disposable silicone or PVC.<br>Shall have a reservoir bag. (Minimum 600ml).<br>Must be supplied in a soft carrying case made of durable, washable material with appropriate carrying straps.<br>**Physical characteristics**:<br>May be stored in any configuration without losing its shape and / or functional capability.<br>**Documentation**:<br>A set of operating manuals shall be supplied in the English language.
Applicable Standards	ISO 10651-4 (2013).
Performance Specifications	Maximum bag volume of 280ml.<br>Maximum tidal volume of 150ml.
Accessories	Must be supplied in a soft carrying case made of durable, washable material with appropriate carrying straps.
Warranties	2 years.
Instructions to Suppliers	
Note to Procurement	Must run as series with Child and Adult manual Resuscitators, And PEEP valve.

Catalogue Number	Item Name	Available on Transversal Contract	Contract Number or Code
PHC-E-141	***Child manual resuscitator***	RT 4	RT 4-05-025 ME

Functional Specifications	Usage for children.<br>Shall allow for single-handed operation.<br>Bag capable of rapid and full re-expansion.<br>Swivel connector present to allow for changing angle of operation.<br>Function not affected by extremes temperatures.<br>Allow for oxygen inlet via oxygen tubing.<br>Cuffed mask shall be clear and provide a leak proof seal.<br>Compatible valve fitting to standard external PEEP fitting W/POP-OFF minimum 30cm H20.<br>Non-disposable silicone or PVC.<br>Shall have a reservoir bag. (Minimum 600ml).<br>Must be supplied in a soft carrying case made of durable, washable material with appropriate carrying straps.<br>**Physical characteristics:**<br>May be stored in any configuration without losing its shape and/or functional capability.<br>**Documentation**:<br>A set of operating manuals shall be supplied in the English language.
Applicable Standards	ISO 10651-4 (2013).
Performance Specifications	Maximum bag volume of 500ml.<br>Maximum tidal volume of 350ml.
Accessories	Mask sizes 1,2 and 3 to be supplied with each item.<br>Must be supplied in a soft carrying case made of durable, washable material with appropriate carrying straps.
Warranties	2 years.
Instructions to Suppliers	
Note to Procurement	Must run as series with Infant and Adult Manual Resuscitators, and PEEP valve.

Catalogue Number	Item Name	Available on Transversal Contract	Contract Number or Code
PHC-E-142	***Adult manual resuscitator***	RT 4	RT 4-05-024 ME

Functional Specifications	Usage for adults.<br>Shall allow for single-handed operation.<br>Bag capable of rapid and full re-expansion.<br>Swivel connector present to allow for changing angle of operation, with standard 22mm outer diameter and 15mm internal diameter fittings.<br>Function not affected by extremes temperatures.<br>Allow for oxygen inlet via oxygen tubing.<br>Cuffed mask shall be clear and provide a leak proof seal.<br>Compatible valve fitting to standard external PEEP fitting W/POP-OFF minimum 60cm H20.<br>Non-disposable silicone or PVC.<br>Shall have a reservoir bag. (Minimum 600ml).<br>Must be supplied in a soft carrying case made of durable, washable material with appropriate carrying straps.<br>**Physical characteristics:**<br>Compatible with all methods of sterilisation and disinfection. Bag should be autoclaveable to 134 °C.<br>May be stored in any configuration without losing its shape and/or functional capability.<br>**Documentation**:<br>A set of operating manuals shall be supplied in the English language.
Applicable Standards	ISO 10651-4 (2013).
Performance Specifications	Maximum bag volume of 1500ml.<br>Maximum tidal volume of 1000ml.
Accessories	Mask sizes 4 and 5 to be supplied with each item.<br>Must be supplied in a soft carrying case made of durable, washable material with appropriate carrying straps.
Warranties	
Instructions to Suppliers	
Note to Procurement	Must run as series with Infant and Child Manual Resuscitators, and PEEP valve.

Catalogue Number	Item Name	Available on Transversal Contract	Contract Number or Code
PHC-E-143	***PEEP valve***	RT 4-2015	RT 4-05-027 ME

Functional Specifications	Clear plastic.<br>Autoclaveable.
Applicable Standards	ISO 10651-4 (2013).
Performance Specifications	Pressure adjustable from 5-20c. of H_2O.<br>Must be cleanable with standard cleaning and disinfectant solutions. And autoclaveable.
Accessories	Must include one adult and one paediatric re-usable probes.
Warranties	2-year warranty.
Instructions to Suppliers	None.
Note to Procurement	Must be in series with RT 4-05-026 ME, RT 4-05-025 ME, RT 4-05-024 ME.

Catalogue Number	Item Name	Available on Transversal Contract	Contract Number or Code
PHC-E-146	***Suction machine: portable***	RT 4	RT 4-05-041 ME

Functional Specifications	Unit must be self-contained in use, and easily carried by hand.<br>Must contain a rechargeable battery (non-memory forming) with built-in or external charger.<br>The gauge must be marked in kPa and mmHg.<br>Must have a battery charge level indicator.<br>On-off switch must be illuminated and have a splash-proof cover.<br>Must have suction control for accurate regulation of the vacuum.<br>One receiver jar of 2l capacity, graduated in 100ml steps.<br>Receiver jar must be high impact crack resistant polycarbonate.<br>Receiver jar and lid must be removable and autoclavable.<br>Receiver jar lid must have overflow protection.<br>Receiver jar must operate without liners.<br>Vacuum pump regulator must be fitted with a hydrophobic bacterial filter to prevent aspirated fluid from entering the suction pump.<br>The suction inlets and connectors must be stainless steel.<br>The pump must be oil free and maintenance free.<br>The unit must operate from a 220 V AC supply.<br>Must have a mains cable at least 3m long fitted with a SABS approved three pin 15A plug.<br>Battery status indicator.<br>Battery capacity: at least 1 hour operation.<br>Battery must be sealed, maintenance free.<br>The suction unit must be easy to service and maintain.
Applicable Standards	ISO 10079-1:2015.
Performance Specifications	Vacuum pressure minimum 75mmHG and adjustable up to 150mmHG.<br>The suction capacity must be at least 25l per minute.<br>Working noise less than 50 dB.
Accessories	Supplied with re-usable, autoclaveable suction tube and cannula.<br>Filter: antibacterial PHC-C-162.

Warranties	2 years.
Instructions to Suppliers	List ALL available consumables with prices incl VAT.<br>List ALL available accessories with prices incl VAT.<br>The unit must be handed over in full operating order.<br>A starter-pack of consumables must be supplied with the unit.<br>Specify any mandatory service required, to keep unit at optimal operating condition.
Note to Procurement	Hydrophobic bacterial filters, must be in series with this item.<br>In this and any future procurement, preference should be given to machines which have reusable autoclave able suction hoses and cannulas, to minimise logistical requirements.<br>Two sets of such re-usable suction hoses and cannula should be ordered, so that one is always available.<br>Under this particular contract RT 4, the machine offered has a 1l jar. However, the supplier should be requested to supply 2 jars of 2l instead.

Catalogue Number	Item Name	Available on Transversal Contract	Contract Number or Code
PHC-E-144	***Suction: trolley-type***	RT 2-2016	42171801-00001

Functional Specifications	Unit must be mounted on mobile stand with at least 4 x castor wheels.<br>Must contain a rechargable battery with built-in charger.<br>Two of the castors must be lockable.<br>Operating pressure: 0 to -80 kPa.<br>The gauge must be marked in kPa and mmHg.<br>The gauge must be fully removable for maintenance.<br>On-off switch must be illuminated and have a splash-proof cover.<br>Must have suction control for accurate regulation of the vacuum.<br>Two receiver jars, each of 2l capacity, graduated in 100 ml steps.<br>Receiver jars must be high impact crack resistant polycarbonate.<br>Receiver jars and lids must be removable and autoclavable.<br>Receiver jar lid must have overflow protection.<br>Receiver jar must operate with and without liners.<br>Vacuum pump regulator must be fitted with a hydrophobic bacterial filter to prevent aspirated fluid from entering the suction pump.<br>The suction inlets and connectors must be stainless steel.<br>The pump must be oil free and maintenance free.<br>The vacuum regulator must be fitted with a probe of the S.A.B.S./Heyer type.<br>The mobile stand must have a handle for pushing.<br>The unit must operate from a 220 V AC supply.<br>Must have a mains cable at least 3 m long fitted with a SABS approved three pin 15A plug.<br>Battery status indicator.<br>Battery capacity: at least 1 hour operation.<br>Battery must be sealed, maintenance free.<br>The unit must meet the appropriate ISO standards.<br>The unit must be CE approved.<br>The suction unit must be easy to service and maintain.
Applicable Standards	ISO 10079-1:2015.

Performance Specifications	The suction capacity must be at least 25l per minute.<br>Working noise less than 50 dB.<br>Vacuum regulator should operate on high vacuum 7 to 67 kPa and low vacuum 0 to 19 kPa.
Accessories	Supplied with re-usable, autoclaveable suction tube and cannula.<br>Filter: antibacterial PHC-C-161.
Warranties	2 years.
Instructions to Suppliers	List ALL available consumables with prices incl VAT.<br>List ALL available accessories with prices incl VAT.<br>The unit must be handed over in full operating order.<br>A starter-pack of consumables must be supplied with the unit.<br>Specify any mandatory service required, to keep unit at optimal operating condition.
Note to Procurement	Hydrophobic bacterial filters, must be in series with this item.<br>In this and any future procurement, preference should be given to machines which have reusable autoclave able suction hoses and cannulas, to minimise logistical requirements.<br>Two sets of such re-usable suction hoses and cannula should be ordered, so that one is always available.

Catalogue Number	Item Name	Available on Transversal Contract	Contract Number or Code
PHC-E-128	***Broselow tape***	RT 4	RT 4-05-010 ME

Functional Specifications	Durable tape with standard length markings and colour zones.<br>Each colour zone is associated with recommended dosages of resuscitation drugs and defibrillation energy for use in infants, where the weight is unknown.<br>Flexible type preferred, for storage purposes. Non-smudge, non-fade. Must be cleanable with detergents and standard disinfectants. Supplier to state which cleaning solutions are unsuitable.<br>Supplier to state whether the basis for the year edition of the tape (the US National Health and Nutrition Examination Survey – NHANES data) has been correlated with similar South African data.
Applicable Standards	None.
Performance Specifications	Supplier to state year edition of tape. Supplier to state whether all required clinical information is printed on tape, or whether a laminated card with resuscitation algorithms is required. In the latter case, supplier to propose such a card, specifying cost.
Accessories	Clear plastic protective cover.
Warranties	None.
Instructions to Suppliers	State expected service life of tape under normal use.
Note to Procurement	These specifications are more extensive than those in RT 4, and should be used in future procurements.

Catalogue Number	Item Name	Available on Transversal Contract	Contract Number or Code
PHC-E-129	***PAWPER tape***	No	

Functional Specifications	The Pawper (Paediatric Advanced Weight Prediction in the Emergency Room) tape is a paediatric resuscitation tape validated for the South African context.<br>Dosage information is on a separate card.
Applicable Standards	None.
Performance Specifications	Supplier to state year edition of tape/card.
Accessories	
Warranties	None.
Instructions to Suppliers	State expected service life of tape under normal use.
Note to Procurement	

POINT OF CARE TESTING

Catalogue Number	Item Name	Available on Transversal Contract	Contract Number or Code
PHC-E-149	***Glucometer***	No	

Functional Specifications	Portable, handheld blood glucose monitor/meter with proven accuracy.<br>Determination of glucose in fresh capillary blood, serum, venous blood and arterial blood by reflectance photometry or amperometric enzyme electrodes, making use of electronic circuitry.<br>Visual back-up (comparison colour in the control window on the back of the strip with colour range on strip vial label).<br>No memory loss during battery changes.<br>Memory capacity- minimum of 500 measurements.<br>Neonatal Clearance from FDA for test measurements.<br>Weight: approximately 50grams (without batteries).<br>Display: LCD with reasonable size display and visible under different lighting conditions.<br>Battery operated (commonly available batteries). Must use replaceable dry cell batteries.<br>Battery indicator warning for low battery.<br>Operator must be able to replace batteries easily without using tools.<br>On/off switch.<br>Button for searching through memory (blood values).<br>Insertion slot to insert test strips.<br>Test strip guide with an indicator that the machine is ready to perform measurements.<br>Automatic measuring of sample.<br>Guarantee on shelf life of test strips irrespective of whether the pack of strips is opened or not.
Applicable Standards	ISO: 15197:2013.<br>FDA Guidance October 2016.
Performance Specifications	Home-use single user devices are not suitable. The device must be disinfectable to prevent blood-borne disease transmission. Specify validated disinfection procedures.<br>Accuracy: 95% of all readings must be within max 15% of lab measurement (12% preferred).<br>99% of measurements must be within 20% of lab measurement (15% preferred). Supply studies.<br>Measuring range 0.6 -30.0 mmol per litre.<br>Measuring time approximately 5 seconds.<br>Automatic cut-off after ± 120 seconds.

Accessories	One pack of glucometer test strips PHC-C-181.<br>One pack of multipurpose lancets PHC-C-146.
Warranties	2 years on all components. Guarantee on shelf life of test strips irrespective of whether the pack of strips is opened or not.
Instructions to Suppliers	After-sales service, must be available.<br>Training must be supplied locally free of charge.
Note to Procurement	Provide for re-calibration services in procurement.<br>Consumables (test strips) must be in series with this item.<br>Lancets are generic to other test equipment (e.g. Haemoglobinometer, HIV test kit), and need not be in series.

Catalogue Number	Item Name	Available on Transversal Contract	Contract Number or Code
PHC-E-152	***Haemoglobinmeter***	No	

Functional Specifications	The meter must be lightweight (approximately 300 grams), robust and simple to operate.<br>Must use either replaceable dry cell batteries or rechargeable batteries.<br>Specify the type and number of dry cell batteries, and expected operating time per charge. If rechargeable, state expected service life of batteries.<br>If the unit has rechargeable batteries, a battery charger, operating from 220V mains supply must be provided.<br>Cable for the battery charger must be at least two metres long and be fitted with a SABS approved 15A plug.<br>Automatic calibration.<br>The display must provide indication of a low battery condition.<br>The unit must display blood haemoglobin test results in g/dl or mmol/l .<br>State whether the unit on offer makes use of disposable cuvettes, or test strips (specify type).<br>The meter must perform self-test/ self-calibrating on power-on. State method of self-calibration, and whether periodic user calibration is required. If so, detail method and materials required and cost thereof.<br>The meter must automatically compensate for turbid blood samples and thereby still provide accurate measurement results.
Applicable Standards	None.
Performance Specifications	The minimum measuring range of the meter must be as follows:<br>0 – 25,6g/dl<br>0 – 15,9mmol/l.<br>The meter must display test results within 60 seconds.<br>The accuracy of the meter must be ± 1,5 %.
Accessories	Case for storage.<br>Lancets, multipurpose PHC-C-146.<br>Hb meter test strips PHC-C-182.<br>Hb meter microcuvette PHC-C-183 (Depending on the model procured).
Warranties	Minimum 2-year guarantee on meter.<br>Supplier to indicate expected number of examinations in average service life of meter.
Instructions to Suppliers	State frequency of recalibration by supplier if any. Such recalibration will be at no cost.<br>Supplier must state the price of all consumables per 100.<br>Includes delivery and group training.<br>Supplier must specify available after-sales support, with relevant pricing.<br>Supplier to train one person per facility.
Note to Procurement	Consumables (Cuvettes or test strips) must be in series with this item.<br>Provide for re-calibration services in procurement.

STERILISATION

Catalogue Number	Item Name	Available on Transversal Contract	Contract Number or Code
PHC-E-157	***Autoclave: tabletop***	No	

The Class B tabletop autoclave must be suitable for a larger clinic or Community Health Centre.
Floor-standing model on castors will be acceptable.
The autoclave must use steam sterilisation.
The autoclave must be fully automatic.
The chamber must be 316 stainless steel. A certificate from the OEM to be attached.
Chamber volume: 40-80l.
The working depth must be at least 400 mm.
Describe the chamber capacity in terms of DIN-StU standard system trays/baskets.
The unit must operate from a standard 220V 50Hz single phase supply. The unit must have a (minimum). 2-metre power cable, with a SABS approved 3-pin 15-amp plug top.
Electric steam generator to be incorporated.
Heating element must be isolated from the water.
Programmable Logic Controller must control all system functions and monitor system operations.
User-friendly operating instructions and procedures.
All text displays must be in English.
The control software must automatically generate information alerts (service intervals, maintenance etc.).
Pressure gauge to monitor chamber pressure.
The following parameters must be displayed during the operation of any programme:

- Chamber temperature
- Chamber pressure
- Steam generator temperature
- Steam generator pressure
- Vacuum indicator
- Operating programme
- Phase of operation
- The controls must be steam and moisture proof.

The autoclave must be able to sterilize the following:

- unwrapped solid and hollow instruments
- pouched solid and hollow instruments
- single wrapped solid and hollow instruments
- double wrapped solid and hollow instruments
- porous items
- prion cycle.

Temperature alarms for all cycles at +1°C.
A Bowie & Dick test cycle must be available.
Leak test facility must be available.
Internal edges and corners to be rounded for effective cleaning.
Internal floor must slope to drain.
Removable stainless steel/brass strainer to prevent drain blockage.
All valves to be stainless steel/brass.
Valves: electric solenoid type.

Functional Specifications	Automatic lock on door when a programme starts.<br>It must not be possible to pressurize the chamber if the door is not fully closed.<br>Safety interlock must prevent the door opening, until the chamber is at atmospheric pressure.<br>Door seal must be replaceable.<br>Visual and audible alarms for cycle malfunctions.<br>Self-diagnostic system with the following warnings:<br>Vacuum period exceeded<br>Steam discharge period exceeded<br>Door lock failure<br>Pressure failure<br>Temperature failure<br>Heater failure<br>Air detector failure<br>Vacuum leakage<br>Door gasket failure<br>Power failure<br>Low chamber steam pressure<br>High chamber steam pressure<br>High chamber temperature<br>No water alarm<br>Low water alarm<br>Temperature & pressure sensor failure<br>Phase time-outs<br>Door not properly closed<br>Continuous self-checking of all safety devices.<br>Air removal by vacuum pulse.<br>Describe type of vacuum pump, and maximum negative pressure.<br>Describe details of water connection. Filter incorporated or external? De-ionised water necessary?<br>Thermal insulation must completely surround the chamber.<br>Chamber must withstand at least 250 kPa overpressure and vacuum.<br>Water quality sensor.<br>HEPA Air filter, easily replaceable. Efficiency greater than 99.9% for particles greater than 0.3 µm.<br>Overpressure valve and all other safety valves must vent into a safe area, and not into working area.<br>Operator controls must be by touch-type, and not switches, for infection control purposes.<br>The heating element must be protected by means of an overheat cut off switch.<br>Describe air venting method and requirements.<br>The unit must store data for at least the last 20 cycles. This data must be available in a printable and downloadable format. The data required is:<br>Type of cycle<br>Date and time<br>Total time for the plateau (Sterilising)<br>Temperature at the plateau (Sterilising)<br>Pressure at peak plateau temperature<br>Indication if a cycle has passed or failed (On printout and downloadable).<br>USB port for data download. Ethernet port will be accepted.<br>Optional Printer to print vital sterilizer data.

Applicable Standards	The autoclave must conform to the following standards. Relevant certificates must be provided: (certificates for equivalent standards may be provided)<br>High pressure high vacuum steam sterilisers: SANS 982 (2009)<br>Pressure Equipment Regulations: Occupational Health and Safety Act (Act No. 85 of 1993, Section 43)<br>ISO 9001<br>Machinery Directive 2006/42/EC<br>Low Voltage Directive 2006/95/EC<br>Pressure Equipment Directive 97/23/EC<br>Sterilization – Large Steam Sterilizers EN 285<br>Sterilization – Moist heat – Validation and routine control EN ISO 17665-1:2006<br>EN 61010 and EN 61010-2-041.
Performance Specifications	Pressure sensor: temperature compensated, 0-400 kPa:<br>Accuracy of 2% over the range<br>Accuracy of 5 kPa at operating pressure.<br>Temperature sensors: 50-150°C:<br>Accuracy of 1% over the range<br>Accuracy of 0.5°C at sterilisation temperature.
Accessories	The unit must include at least three perforated trays and not the box type. The box type may be offered as an extra.<br>Each tray must hold at least 10 dental instruments. The chamber must be long enough to sterilise instruments up to 400mm long to enable sterilising of maternity instruments.<br>1 replacement door seal.<br>1 helix test kit PHC-C-214.<br>1 Bowie-Dick test pack PHC-C-213.
Warranties	2 year guarantee.<br>Vacuum pump to be guaranteed for 5 years.<br>Heating elements guaranteed for ten years.
Instructions to Suppliers	Technical details to be provided in a separate document.<br>Specify power and water consumption per cycle.<br>Indicate whether energy/water- saving features are standard or optional.<br>Any other relevant certificates may be submitted.<br>Suppliers to fully describe, in a separate document, all relevant information regarding the cycles available (Vacuum, Pressure, Temperature etc.).<br>Range of accessories that can be ordered as required.<br>Bidders must stipulate the cost of the following spare parts V.A.T. included:<br>Door gasket.<br>A schedule of the preventive maintenance programme must be supplied.<br>Supplier to indicate which consumable items are required for (a) normal operation (b) standard maintenance and (c) testing of the equipment.
Note to Procurement	5-year all-inclusive maintenance contract to be included.<br>Procurement of Bowie-Dick PHC C-213 and Helix tests kits PHC-C-214 required.

Catalogue Number	Item Name	Available on Transversal Contract	Contract Number or Code
PHC-E-112	***Instrument steriliser: desktop***	No	

Functional Specifications	A Table top B class autoclave with a PRION Cycle.<br>Mains voltage MUST operate at 220 – 240V.<br>Current MUST not exceed 15 Amps.<br>MUST have no re-circulation of used water.<br>Chamber MUST be constructed of stainless steel or copper brass alloy. MUST be ASME or SABS approved.<br>The unit must have ability to heat the entire chamber efficiently.<br>MUST have heat insulated casing with built-in condensation coils and tank.<br>MUST have automatic timer to complete sterilizing cycle.<br>MUST be fitted with safety pressure valve.<br>MUST be fitted with temperature and pressure gauges.<br>Complete cycle after warm up MUST not exceed 30 min.<br>Chamber dimensions MUST be ±510mm deep by ±360mm diameter.<br>Minimum reservoir capacity 4l.<br>15 Amp plug MUST be supplied.<br>MUST have separate drying cycles which shut off automatically after 7 – 12 minutes.<br>The unit MUST be of a horizontal, chamber-type autoclave, and the entire unit MUST be microprocessor controlled.<br>The unit MUST monitor the water level, and if low, switch off to protect the elements from overheating and damage. The unit MUST switch off when water level low, and not when overheating.<br>The unit MUST include at least 3 perforated trays with a detacheable handle.<br>Each tray MUST hold at least 10 dental instruments. The chamber MUST be long enough to sterilize instruments up to 300mm long.<br>All cycles MUST be automatic – no user intervention MUST be required, except to select the correct cycle and start the cycle.<br>End of cycle, and all error messages, MUST be indicated by a clear audible signal.<br>It MUST be possible to terminate any cycle at any stage.<br>The unit MUST display the operating temperature in an electronic format, and also display pressure.<br>The unit MUST be automatically controlled by temperature. Control for pressure as an additional measure will serve as a recommendation.<br>The unit MUST automatically protect itself and the operator against over temperature and overpressure.<br>The unit must also store data of at least the last 40 cycles. This data must be available in a printable and downloadable format. The unit MUST be able to connect to a computer, via a USB connection, to download the required information. **If any software program is needed to perform this, it must be included in the bid price as an accessory.**

	Water MUST be drained from the chamber after each and every cycle.<br>External elements must be used, please state the wattage of the elements.<br>The unit MUST have an insulated shell to protect operator from accidental heat injuries.<br>Overpressure valve and all other safety valves must vent into a safe area, and not into working area.<br>Unit must monitor the water quality in chamber, and stop cycle if the quality is not satisfactory.<br>Reservoir and chamber to have a manual drain facility that is controlled with a positive on/off valve.<br>Operator controls MUST be by touch-type, and not switches for infection control purposes.<br>All electrical boards/electronic components MUST be well shielded/insulated from heat and potential water spillage.<br>The door mechanism MUST have an auto-lock safety facility to prevent the door being opened during a cycle.<br>It MUST not be possible to start a cycle if the door is not properly closed and sealed.<br>The autoclave is to display time lapsed and the time remaining on cycle.<br>The autoclave is to display time lapsed and the time remaining on cycle.<br>The entire unit must form a self-contained working unit. It must be easy to move the unit to where it's required.<br>Capacity of the chamber MUST be 16+ litres.
Applicable Standards	IEC 601-1, IEC 601-2, ISO 13458, Applicable parts of SANS 40:2009 (ed2.03 or later), ANSI/AAMI ST55:2010/ (R) 2014.
Performance Specifications	Operating pressure MUST be 240 KPa.<br>Operating temperature MUST not exceed 135°C.<br>The unit MUST be able to perform the following cycle:<br>134º with vacuum with drying<br>134º with vacuum without drying<br>134º non-vacuum with drying<br>134º non-vacuum without drying<br>121º with vacuum with drying<br>121º with vacuum without drying<br>121º non-vacuum with drying<br>121º non-vacuum without drying.<br>Unit MUST have a Prion cycle.<br>The units MUST be able to sterilize unwrapped solids and hollow instruments, pouched solid and hollow instruments, single wrapped solid and hollow instruments, double wrapped solid and hollow instruments and porous items.<br>Expected service life: 10 years.<br>The up-time of the unit must be better than 98%, excluding scheduled preventative maintenance and software upgrades, measured on a quarterly basis. The percentage lower than 98% will be added to the warranty period. A sliding scale penalty clause will form part of the service contract. This will result in the maintenance payment being reduced by a pro rata amount that the up-time is less than 98%.

Accessories	Unit must be supplied with a spare door seal, and all necessary parts and consumables, to allow immediate operation after commissioning.<br>Bowie-Dick test packs PHC-C-213.<br>Helix test kits and strips PHC-C-214.
Warranties	2 years.<br>Availability of spares guaranteed for 10 years.<br>If unit requires off-site repair, a loan unit will be provided at no additional cost.<br>Software changes to the equipment which are corrective in nature and initiated due to software errors, regulatory requirements or safety reasons, shall be delivered and installed at no charge for the life of the equipment.
Instructions to Suppliers	Please provide cycle times for each of the programs for hot and cold cycle.<br>Water distiller/demineraliser to be included for each autoclave. This must meet the required water quality for this autoclave. The price of this unit must be included in the bid price.<br>Supply an all- inclusive, fully comprehensive five year preventative maintenance, service and repair contract covering all equipment, hardware and software. This contract would cover, but not be limited to the following: ALL PARTS, labour, traveling, accommodation, service and maintenance. The five- year maintenance plan must also include all quality check and quality assurance requirements, including all required calibrations. This contract will commence after the two- year warranty period has expired. Software updates and upgrades to be included.<br>Supplier to deliver and commission.<br>Supplier to train staff at each facility on correct operation.
Note to Procurement	Procurement must include minimum 5-year maintenance plan.

ORAL HEALTH

Note:
- The technical specifications in this sub- section have been included for guidance. Term contracts for almost all the items will be released in the near future, and it is recommended that purchases should be made from the term contracts, as the offerings will have been diligently selected.
- The layout and installation of much of this equipment is critical with regard to space utilisation and efficient workflow. Floor-mounted items require under-floor services. Installation should be carefully planned, in consultation with competent professionals in the field, as well as Provincial infrastructure officials.
- Dental Assistant's stool and Dentist's Stool are located in Furniture – Clinical.

Catalogue Number	Item Name	Available on Transversal Contract	Contract Number or Code
PHC-E-164	***Amalgam separator: 2 surgeries***	In preparation	

Functional Specifications	A fixed installation amalgam separator for up to 2 surgeries. The unit MUST have the following features:<br>Fixed installation<br>Equipped for connection of several treatment units<br>Serve number of surgeries: Maximum 2<br>Output power: Single-phase, Approximately 115W<br>Noise level: No more than 60dB(A)<br>Volume of collector vessel: Approximately 90 cm^3<br>Separation rate: At least 95%<br>Flow rate at least 4 l/min<br>Automatic filling level check<br>Automatic switch on when waste water is available from treatment unit<br>Centrifugal separator with hydrodynamic self-cleaning function.
Applicable Standards	ISO 11143: 1999.
Performance Specifications	Expected service life 10 years.
Accessories	Collection Container with recycle kit (optional).
Warranties	2 years.<br>Availability of spares guaranteed for 10 years.
Instructions to Suppliers	Installation should be carefully planned, in consultation with competent professionals in the field, as well as Provincial infrastructure officials.
Note to Procurement	

Catalogue Number	Item Name	Available on Transversal Contract	Contract Number or Code
PHC-E-166	***Amalgamator: digital***	In preparation	

Functional Specifications	Digitally controlled amalgamator.<br>Must accept mercury and glass ionomer capsules.<br>Automatic switch-off. Electronic time control.<br>Operating cycle up to 99 seconds.<br>Body of shcock-resistant plastic. Double-insulated.<br>Must operate on 240V AC, 50/60 Hz.<br>Must be fitted with power cord and SABS-approved moulded 15-amp, 3-prong plug.<br>Unit to have a sealed and isolated mixing chamber which deactivates on opening.
Applicable Standards	ISO 7488 most recent.<br>ISO 13897: 2018.<br>IEC 80601-2-60: 2012.
Performance Specifications	Noise level less than 65dB.<br>Expected service life 10 years.
Accessories	Replaceable capsule holder.
Warranties	2 years.<br>Availability of spares guaranteed for 10 years.
Instructions to Suppliers	Installation should be carefully planned, in consultation with competent professionals in the field, as well as Provincial infrastructure officials.
Note to Procurement	

Catalogue Number	Item Name	Available on Transversal Contract	Contract Number or Code
PHC-E-167	***Desktop Autoclave S-Type for Dental Instruments***	In preparation	

Functional Specifications	Cassette-type autoclave.<br>Distilled water reservoir.<br>Steam generator.<br>Must have S cycle – chamber air displaced by pressurised steam. Positive Pressure Pulsed Air displacement and shorter cycle times preferred.<br>Cassette measurements (external): minimum 48cm x 17cm X8cm.<br>Sterilisation chamber volume minimum 1,6l.<br>Thin-walled cassette, with external cool-touch handle.<br>Bacterial filter for drying cycle.<br>Easy cycle selection. Automatic temperature, pressure and time control and recording.<br>2m power lead with moulded SABS-approved 15 Amp 3-prong plug.
Applicable Standards	93/42/EEC Medical Device Directive.<br>PrEn 13060 Small steam sterilizer – General Requirements.<br>EN 61010-1 laboratory equipment – Safety requirements.<br>EN 61010 -2-041 Specific instructions for steam steriliser.<br>EN 61326 Electromagnetic compatibility.
Performance Specifications	Temperature 134°C for normal instruments, 121°C for rubber or plastics.<br>Full sterilisation cycle (including drying) in less than 18 minutes.<br>Automatic cycle abortion and alarm if any parameter not reached or maintained.<br>Expected service life 15 years.
Accessories	Thin-walled cassette.<br>Bowie-dick test pack PHC-C-213.<br>Helix Test Kit PHC-C-214.
Warranties	2 years.<br>Availability of spares guaranteed for 10 years.
Instructions to Suppliers	Provide pricing for additional cassette.<br>Describe how cycle data can be extracted from the autoclave, and provide pricing for any options (e.g. data logger, USB or Ethernet connectivity, built-in printer or printer port).<br>Specify whether a demineraliser or a distiller is required.<br>Water demineraliser and/or distiller must be priced separately.
Note to Procurement	In most cases, a water demineraliser or distiller will be required (unless the facility has an existing distiller). Will always be required for use by an outreach team.<br>Procurement of Bowie-Dick PHC C-213 and Helix tests kits PHC-C-214 required.<br>Multi-year all-inclusive maintenance plan preferred.

Catalogue Number	Item Name	Available on Transversal Contract	Contract Number or Code
PHC-E-168	***Compressor: 2-4 surgeries***	In preparation	

Functional Specifications	Voltage 220/240 V 1,5 kW with built in thermal overload protection as per user requirement.<br>Power 0.75Kw 1HP.<br>Fitted with ¼" safety release valve.<br>Tank pressure gauge.<br>Pressure range control switch, 6 – 10 bar.<br>Noise level maximum 60 DB (A).<br>Either piston type or turbo type.<br>Reverse pressure exhaust valve for motor protection.<br>Air filter for filtration of 10 micron particles, with automatic drain valve.<br>Oil free with automatic drainage system.
Applicable Standards	ISO 22052.<br>ISO 22595-2:2008.<br>ISO 80601-2-2-12.
Performance Specifications	Delivery capacity not less than 150 1/min maximum pressure 1000 kPa.<br>Storage Capacity at least 35l-75l.<br>Expected service life 15 years.
Accessories	
Warranties	2 years.<br>Availability of spares guaranteed for 10 years.
Instructions to Suppliers	Inspect premises and agree optimal installation location with competent professional personnel and provincial infrastructure personnel.
Note to Procurement	5-year maintenance contract preferred. This item is not needed for single surgery-mobile dental delivery system has built-in compressor.

Catalogue Number	Item Name	Available on Transversal Contract	Contract Number or Code
PHC-E-169	***Cuspidor/Spittoon/ Water Unit***	In preparation	

Functional Specifications	Chair-mounted either left or right.<br>Spittoon must be removable without any tools, for easy cleaning.<br>Ceramic bowls spittoon.<br>Warm water tumbler filler.<br>Spittoon flush facilities.<br>Solids trap for spittoon bowl.<br>Selective holder consisting of:<br>Solids collector<br>One HVE suction tube<br>One saliva ejector tube<br>Cup fill shut off valve incorporated<br>Low voltage remote control for suction.
Applicable Standards	ISO 7494-2:2015.<br>ISO 16945: 2015 Test methods for dental unit waterline biofilm treatment.<br>IEC 80601-2-60: 2012.
Performance Specifications	Expected service life 15 years.
Accessories	Autoclaveable HVE cannulas to be supplied.
Warranties	5 years.<br>Availability of spares guaranteed for 10 years.
Instructions to Suppliers	Inspect premises and agree optimal installation location with competent professional personnel and provincial infrastructure personnel.
Note to Procurement	5-year maintenance contract preferred.

Catalogue Number	Item Name	Available on Transversal Contract	Contract Number or Code
PHC-E-170	***Delivery system: mobile, with hand pieces, non-fibre-optic***	In preparation	

Functional Specifications	Dental cabinet housing the following:<br>3 handpiece outlets (4-6 holes)<br>3 in 1 syringe<br>Selector switch<br>Foot control<br>Master shut off assembly<br>Compressor (25l-50l; ≥100l/min)<br>Cleanwater system<br>Self-illuminating LED handpiece with coupling<br>Internal spray straight handpiece<br>Contra angle blue handpiece<br>Prophylaxis handpiece<br>Air motor 4-hole<br>The unit must operate on 240vac 50/60hz fitted with 3-prongs plug<br>Unit to be fitted with smooth running four swivel lockable wheels<br>Height = 700mm-900mm width = 400mm-500mm depth = 480mm-600mm.
Applicable Standards	ISO 7494-1:2011.<br>ISO 7494-2:2015.<br>ISO 16954: 2015.<br>IEC 80601-2-60: 2012.<br>ISO 14457:2017 handpieces and motors.
Performance Specifications	Expected service life 15 years.
Accessories	All handpieces, heads and connectors. All installation hardware.
Warranties	5 years.<br>Availability of spares guaranteed for 10 years.
Instructions to Suppliers	Inspect premises and agree optimal installation location with competent professional personnel and provincial infrastructure personnel.
Note to Procurement	This is the preferred option for a single surgery, in a facility without a central compressor. Not compatible with fibre-optic hand pieces.<br>5 year maintenance contract preferred.

Catalogue Number	Item Name	Available on Transversal Contract	Contract Number or Code
PHC-E-171	***Delivery system: fixed, with hand pieces, non-fibre-optic***	In preparation	

Functional Specifications	A Dental delivery system, complete with tubing, spittoon, scaler, clean water system and curing light.<br>Control block must have 4-chair position controls (including reset button).<br>The unit must be connected to the cuspidor/ spittoon/ water unit.<br>Unit must have four/ six (4/6) Handpiece tube holes or more (all modes/functions) with self-activations.<br>Automatic selection of cutting H/P.<br>3 in 1 Syringe with pinch valve technique and sterilisable sleeve and nozzle.<br>The unit to have at least two brake handles (one on each side of delivery head).<br>To have touchpad for controls.<br>Console with water flow control valve for each handpiece, if not incorporated in handpiece: must have a minimum of 4 tubes.<br>Console with 4 control switches for the following:<br>on/off Handpiece water/air spray<br>on/off handpiece air spray coolant<br>on/off tumbler filler<br>on/off spittoon bowel rinse.<br>Individual handpiece pressure adjustment reliable water retraction system required.<br>Self-illuminating LED handpiece with coupling.<br>Internal spray straight handpiece.<br>Contra angle blue handpiece.<br>Prophylaxis handpiece.<br>Air motor 4-hole.<br>The heads must be durable, providing many years of reliability.<br>Free hanging straight/ whip arm air outlet hoses are preferred.<br>The clean water bottle must be standard (1l).<br>It must come with a piezo scaler system with foot switch.<br>The curing light (LED with at least 3 settings levels) must also be supplied with the system.<br>Master on/off switch provided at connection box.<br>SABS PLUG (2 Phase).
Applicable Standards	ISO 7494-1:2011.<br>ISO 7494-2:2015.<br>ISO 16954: 2015.<br>IEC 80601-2-60: 2012.<br>ISO 10650:2015 Powered polymerisation activators.

Performance Specifications	Expected service life 15 years.
Accessories	All handpieces, heads and connectors. All installation hardware.
Warranties	5 years.<br>Availability of spares guaranteed for 10 years.
Instructions to Suppliers	Inspect premises and agree optimal installation location with competent professional personnel and provincial infrastructure personnel.
Note to Procurement	

Catalogue Number	Item Name	Available on Transversal Contract	Contract Number or Code
PHC-E-172	***Dental chair: basic***	In preparation	

Functional Specifications	Anatomically contoured seat and backrest for comfortable full support in all positions suitable for treatment of both adults & children.<br>The chair offered must have double articulating headrest which is reversible (cushion included).<br>Adjustable backrest-thickness 25mm-50mm.<br>Upholstery must be seamless durable waterproof material that can be easily cleaned and withstand disinfectants and cleaning chemicals.<br>Upholstery must be removable.<br>Replaceable plastic cover must be provided to protect upholstery in shoe area.<br>Armrest: Positions swing out/ down Arm.<br>Chair must be Programmable.<br>The chair must be floor-mounted.<br>Accessory foot controls MUST be fitted to chair base to facilitate both height and Trendelenburg adjustment.<br>Total length at maximum extension 1820mm-2000mm.<br>Base (H x W x D) : 600mm -650mm x 500mm-560mm x 490mm-510mm.<br>Electro mechanical/hydraulic lift system fitted in chair base.<br>The seat surface must be 410mm-480mm x 400mm-480mm (LxB).<br>Chair swivel 60°-70° (≤35° each side of center).<br>To operate on 240 VAC 50/60Hz, fitted with standard hospital 3-prongs plug.
Applicable Standards	ISO 6875: 2011.
Performance Specifications	Range of lift height 342.90 mm to 800.10 mm.<br>Recline range +62° to -12°.<br>Weight 200kg SAMHS (to provide (carry capacity of 200kg).<br>Expected service life 15 years.
Accessories	
Warranties	5 years.<br>Availability of spares guaranteed for minimum 10 years.
Instructions to Suppliers	Inspect premises and agree optimal installation location with competent professional personnel and provincial infrastructure personnel.
Note to Procurement	5-year maintenance contract preferred.

Catalogue Number	Item Name	Available on Transversal Contract	Contract Number or Code
PHC-E-173	***Dental chair: portable***	In preparation	

Functional Specifications	A Portable chair that can be carried by an operator.<br>The unit must be able to carry up to 200kg.<br>It must have medium alloy construction.<br>The unit must be able to be used as a sit-up chair.<br>The maximum weight must not be more than 12.5kg.<br>The dimensions must not be more than 510 x 270 x1000mm.<br>It must come complete with a back pack.<br>The unit must make provision for a portable dental light, spit funnel and tray.
Applicable Standards	ISO 6875: 2011.
Performance Specifications	The seat must adjustable from 35 to 60cm.<br>Expected service life 10 years.
Accessories	Backpack.<br>Compatible portable dental light, spit funnel and tray.
Warranties	2 years.<br>Availability of spares guaranteed for 10 years.
Instructions to Suppliers	
Note to Procurement	Ensure that order includes portable dental light, spit funnel and tray, unless specifically excluded by the requesting user.

Catalogue Number	Item Name	Available on Transversal Contract	Contract Number or Code
PHC-E-174	***Dental curing light: corded***	No	
PHC-E-175	***Dental curing light: cordless***	No	

Functional Specifications	Quart tungsten halogen or LED technology.<br>Built-in cooling system.<br>In cordless models, audio-visual warning when battery charge is dropping below adequate levels to deliver stated irradiance. Auto-shutdown when power too low.<br>Selectable exposure time. Accurate timer control.<br>Corded model to have power cord with moulded SABS-approved plug. Double-insulated.
Applicable Standards	ISO 10650:2015 Powered polymerisation activators.
Performance Specifications	The ISO standard is insufficient. Additional requirements:<br>Verifiable documentation of irradiance (milliWatt/cm^2 at light tip, and decrease in irradiance as a function of distance from the tip (in mm, up to 10mm)<br>Verifiable documentation of spectral power vs wavelength of light<br>Verifiable analysis of the irradiance distribution (beam profile)<br>Expected service life 10 years.
Accessories	All necessary curing probes/tips.<br>Cordless model must be supplied with charger (complete with power cord).
Warranties	2 years.<br>Availability of spares guaranteed for 5 years.
Instructions to Suppliers	Describe the cooling system of the device.
Note to Procurement	Some low-cost devices that nominally meet the ISO standard, fail to achieve adequate resin hardening in practice. It is therefore necessary to insist on the validations listed above, in addition to proof of compliance with ISO 10650:2015.

Catalogue Number	Item Name	Available on Transversal Contract	Contract Number or Code
PHC-E-176	***Turbine: full range***	In preparation	
PHC-E-177	LED straight handpiece: for micromotor – 1:1		
PHC-E-178	LED contra-angle handpiece for micromotor – 1:1		
PHC-E-179	LED contra-angle handpiece for micromotor – 1:1 blue		
PHC-E-180	LED contra-angle handpiece for micromotor – reduction, green		
PHC-E-181	LED contra-angle handpiece for micromotor – increasing, red		
PHC-E-182	LED Prophylaxis handpiece: reduction, green		
PHC-E-183	Micromotor: air		

Functional Specifications	**TURBINE FULL RANGE**<br>To fit 4-6 hole standard supply tubing.<br>Speed 350 000 rpm minimum at 2.2 kg per cm.<br>Ballbearing, output approximately 10 watts.<br>Field replaceable rotor, one spare rotor to be supplied.<br>Metal bur change chuck, push type system.<br>Quick action coupling for easy lubrication.<br>Guarantee time of turbine must be indicated – minimum 12 months.<br>Air consumption MUST exceed 36 1/m.<br>Noise level not to exceed 75 db.<br>Autoclavable up to 135°C.<br><br>**With non fibre optic function**<br>To come with durable metal casing (stainless steel).<br><br>**LED STRAIGHT HANDPIECE FOR MICROMOTOR – AIR FULL RANGE**<br>Standard 1 : 1 transmission.<br>Ballbearing.<br>To take burs ISO type 104 44, 50 mm with diameter of 2,35 mm.<br>Maximum speed 40 000 rpm.<br>E-type quick disconnection.<br>Water spray incorporated internally.<br>Autoclavable up to 135°C.<br>Autoclavable up to 135°C.<br>Quick action coupling for easy lubrication.<br>To come with durable metal casing (stainless steel).

Functional Specifications	**LED CONTRA-ANGLE HANDPIECE FOR MICROMOTOR – AIR FULL RANGE**<br>Standard transmission 1 : 1 blue.<br>Push button type system.<br>Ballbearing type.<br>Maximum speed 40 000 rpm.<br>To accommodate ISO type 204 2.35 mm diameter latch type burs.<br>E-type quick disconnection.<br>Water spray incorporated internally.<br>Autoclavable up to 135°C.<br>Quick action coupling for easy lubrication.<br>To come with durable metal casing (stainless steel).<br><br>**LED CONTRA-ANGLE HANDPIECE FOR MICROMOTOR – AIR FULL RANGE**<br>Reduction hand piece Green.<br>Push button type system.<br>Ballbearing type.<br>To accommodate ISO type 204 2.35 mm diameter latch type burs.<br>E-type quick disconnection.<br>Water spray incorporated internally.<br>Autoclavable up to 135°C.<br>Quick action coupling for easy lubrication.<br>To come with durable metal casing (stainless steel).<br><br>**LED CONTRA-ANGLE HANDPIECE FOR MICROMOTOR – AIR FULL RANGE**<br>Speed increasing red.<br>Push button type system.<br>Ballbearing type.<br>To accommodate ISO type 204 2.35 mm diameter latch type burs.<br>E-type quick disconnection.<br>Water spray incorporated internally.<br>Autoclavable up to 135°C.<br>Quick action coupling for easy lubrication.<br>To come with durable metal casing (stainless steel).<br><br>**LED PROPHYLAXIS HANDPIECE FULL RANGE**<br>Reduction Green.<br>To fit E-Type quick disconnection.<br>Sealed ball bearing.<br>To accept snap-on and or screw in cups and brushes.<br>Autoclavable up to 135°C.<br>Quick action coupling for easy lubrication.<br>To come with durable metal casing (stainless steel).

Functional Specifications	**MICROMOTOR – AIR FULL RANGE**<br>Speed range 5 000 – 40 000 rpm.<br>Output 20W Power – min Ncm Torque min.<br>E-Type quick coupling.<br>Forward and reverse rotation.<br>Weight not to exceed 90gm. Speed control selector by means of control ring.<br>Water spray/air facility to be incorporated internally.<br>Maximum air consumption 45 1/m.<br>Autoclavable up to135°C.<br>Quick action coupling for easy lubrication.<br>To come with durable metal casing (stainless steel).
Applicable Standards	ISO 14457: 2017.
Performance Specifications	Expected service life 10 years.
Accessories	
Warranties	2 years.
Instructions to Suppliers	
Note to Procurement	

Catalogue Number	Item Name	Available on Transversal Contract	Contract Number or Code
PHC-E-184	***Dental light: LED***	In preparation	

Functional Specifications	The light MUST use LED technology.<br>Touch sensor ON/OFF and manual control.<br>Voltage 220 – 240, 50 Hz.<br>Can be mounted to Chair or Ceiling.<br>Rotational axes: Horizontal, vertical, diagonal.<br>Constant colour temperature 5,000K.<br>Temperature increase not more than 5 degrees C above room temperature at maximum intensity.<br>Light pattern: 85mm-95mm x 145mm-160mm at 700mm.<br>Shadow-free illumination.<br>On/Off switch in lamp yoke, and provision for altering light intensity, without changing colour temperature.<br>Power Consumption: 12W-17W or less.<br>With Mode Selection.<br>The light must have a shield that is scratch and discolouration resistant for protection against bulb shattering.
Applicable Standards	ISO 9670: 2014.
Performance Specifications	Adjustable light intensity: 15000Lux-30000Lux at constant colour temperature.<br>Expected service life 15 years.
Accessories	All mounting hardware.<br>1 spare bulb.
Warranties	5 years.<br>Availability of spares guaranteed for 10 years.
Instructions to Suppliers	Describe ceiling weight-bearing requirements.<br>Inspect premises and agree optimal installation location with competent professional personnel and provincial infrastructure personnel. Deliver, install, connect and commission.
Note to Procurement	Spare LED bulb in series with this item.

Catalogue Number	Item Name	Available on Transversal Contract	Contract Number or Code
PHC-E-185	***Dental scaler: air***	In preparation	

Functional Specifications	Delivery-unit mountable scaler.<br>Handpiece washable and autoclaveable up to 135ºc.<br>At least 3 scaling tips.<br>Tip wrench with torque limiter.<br>Compatible with 4/6 hole tubing.
Applicable Standards	ISO 18397: 2016.
Performance Specifications	Expected service life 10 years.
Accessories	Including all accessories.
Warranties	2 years.<br>Availability of spares guaranteed for 10 years.
Instructions to Suppliers	
Note to Procurement	

Catalogue Number	Item Name	Available on Transversal Contract	Contract Number or Code
PHC-E-186	***Suction: dry, 2-4 surgeries***	In preparation	

Functional Specifications	Fixed installation.<br>Serve number of surgeries: 2-3.<br>Output power: Single-phase, Approximately 1.8kW-5A, 50 Hz.<br>Speed of motor: No less than 2800 rpm.<br>Vacuum setting at approximately 170 mBar.<br>Noise level without housing: No more than 65dB(A).
Applicable Standards	ISO 10637:1999.<br>ISO 22595-1:2006.
Performance Specifications	Expected service life 15 years.
Accessories	
Warranties	2 years.<br>Availability of spares guaranteed for 10 years.
Instructions to Suppliers	Installation should be carefully planned, in consultation with competent professionals in the field, as well as Provincial infrastructure officials.
Note to Procurement	

Catalogue Number	Item Name	Available on Transversal Contract	Contract Number or Code
PHC-E-187	***Suction: mobile***	In preparation	

Functional Specifications	A mobile suction unit for continuous utilisation in a dental surgery. The unit MUST have the following features:<br>Mobile unit to be steered around chair and between surgeries<br>Equipped with 4 double wheel castors, one per corner<br>Designed for continuous service<br>Capacity of canister: minimum 4l<br>Canister to be emptied manually.<br>Noise level: No more than 65dB(A).<br>Dimensions: Approximately 550mm W, 350mm D, 750mm H.<br>Net weight: Approximately 35kg.<br>Gross weight: Approximately 40kg.
Applicable Standards	
Performance Specifications	Expected service life 15 years.
Accessories	
Warranties	2 years.<br>Availability of spares guaranteed for 10 years.
Instructions to Suppliers	
Note to Procurement	

Catalogue Number	Item Name	Available on Transversal Contract	Contract Number or Code
PHC-E-188	***Suction: wet, 2-4 surgeries***	In preparation	

Functional Specifications	Fixed installation.<br>Serve number of surgeries: 2-4.<br>Output power: Single-phase, Approximately 1.7kW-9A.<br>Maximum flow: No less than 820 l/min.<br>Operating head for continuous service: Maximum 3300mm H2O.<br>Average water consumption: Approximately 6 l/min.<br>Piping pressure: Approximately 4 bar.<br>Dimensions: Approximately 370mm W, 300mm D, 420mm.
Applicable Standards	ISO 10637:1999.<br>ISO 22595-1:2006.
Performance Specifications	Maximum flow: No less than 820 l/min.<br>Operating head for continuous service: Maximum 3300mm H2O.<br>Expected service life 15 years.
Accessories	
Warranties	2 years.<br>Availability of spares guaranteed for 10 years.
Instructions to Suppliers	Installation should be carefully planned, in consultation with competent professionals in the field, as well as Provincial infrastructure officials.
Note to Procurement	

Catalogue Number	Item Name	Available on Transversal Contract	Contract Number or Code
PHC-E-189	***Ultrasonic cleaner***	In preparation	

Functional Specifications	An ultrasonic bath utilised for the cleaning of dental instruments prior to sterilisation. The unit shall have the following features:<br>Minimum 4.5l tank (working capacity)<br>Supplied with instrument basket<br>Equipped with an integrated timer<br>Integrated hinged lid<br>SABS PLUG.
Applicable Standards	
Performance Specifications	Expected service life 10 years.
Accessories	Instrument basket – standard.<br>Optional items:<br>Basket<br>Beaker<br>Bur tray.
Warranties	1 year.<br>Availability of spares guaranteed for 5 years.
Instructions to Suppliers	Describe validation procedures, and recommended frequency of validation. Note any equipment/ consumables required, with cost.
Note to Procurement	

Catalogue Number	Item Name	Available on Transversal Contract	Contract Number or Code
PHC-E-191	***Water distillation***	In preparation	

Functional Specifications	A desktop water distillation unit for dental autoclaves. The unit MUST have the following features:<br>Must be able to supply at least 4l of distilled water to supply different types of autoclaves<br>SABS Plug.
Applicable Standards	
Performance Specifications	Expected service life 10 years.
Accessories	
Warranties	1 year.
Instructions to Suppliers	Full installation of the water distillation unit on delivery.
Note to Procurement	

Catalogue Number	Item Name	Available on Transversal Contract	Contract Number or Code
PHC-E-192	***X-ray: wall-mounted***	In preparation	

Functional Specifications	An X-ray unit for capturing intra-oral analogue and digital images (Floor and wall mounted options).<br>The unit must be a wall mount type (with an option of a long arm).<br>The tube head MUST be between 60kV to 70kV<br>The unit must have a computerized timer with the following functions:<br>Automatic setting of technique factors in mAs<br>Object program selection<br>Tooth type<br>Patient size<br>Manual override.<br>The focal point of the unit MUST be from 0.4mm to 0.8mm.<br>The power supply of the unit must be mounted on the unit.<br>The unit MUST use a line voltage of 220VAC to 240VAC.<br>The line frequency MUST be 50Hz.<br>Analogue and digital.<br>SABS Plug (2Phase).
Applicable Standards	IEC 80601-2-60: 2012.
Performance Specifications	Expected service life 15 years.
Accessories	Remote control – corded or cordless.
Warranties	10 years on arm.<br>5 years on X-ray tube.
Instructions to Suppliers	Installation should be carefully planned, in consultation with competent professionals in the field, as well as Provincial infrastructure officials. Positioning is critical for smooth workflow.<br>State wall load-bearing requirements.
Note to Procurement	This item must be procured together with a digital imaging plate and laptop/PC, unless it is already included in the offering.

Catalogue Number	Item Name	Available on Transversal Contract	Contract Number or Code
PHC-E-193	***X-ray: Digital oral imaging plate with a computer***	In preparation	

Functional Specifications	An X-ray system utilising plates for quick digitising of intra-oral images. The unit MUST have the following features:<br>Must be compatible with AC and DC intra oral X-ray Units<br>Software including implant module and implant archive<br>Fast Ethernet connection. Must include all cabling and installation from the unit to the surgeries<br>4 X imaging plate sets per unit (1 for every surgery) MUST be supplied and includes (per surgery):<br>a. 2 x 22x33mm plates<br>b. 2 X 24x40mm plates<br>c. 5 X 31x41 mm plates<br>d. 1 X 27x45mm plate<br>e. 1 X storage box for plates<br>f. 100 pieces of hygiene bags per size.<br>The unit must be provided with an up to date desktop/laptop computer with the following requirements:<br>≥4GB Ram<br>≥1TB Hard drive<br>17 inch LED screen<br>Software for processing all captured images<br>A keyboard and mouse (cordless/cord)<br>The unit MUST use a line voltage of 220VAC to 240VAC<br>The line frequency MUST be 50Hz<br>At least windows 10<br>The unit must be free standing with height adjustment<br>SABS Plug (2 Phase).
Applicable Standards	
Performance Specifications	Expected service life 10 years.
Accessories	
Warranties	2 years.<br>Spares availability guarantee 5 years. ISO 7493: 2006.
Instructions to Suppliers	
Note to Procurement	Procure suitable laptop/PC from available term contracts.

General equipment

Building maintenance

Catalogue Number	Item Name	Available on Transversal Contract	Contract Number or Code
PHC-E-194	***Allen key set***	No	

Functional Specifications	Drop forged steel. T-style Allen keys, with short Allen key of same size protruding from one end of the handle.<br>Sizes: 1.5, 2, 2.5, 3, 4, 5, 6, 8, 10 mm.<br>Spherical head preferred, but not essential.
Applicable Standards	ANSI B18.3.2M-1979.
Performance Specifications	Expected service life 10 years.
Accessories	
Warranties	1 year.
Instructions to Suppliers	
Note to Procurement	

Catalogue Number	Item Name	Available on Transversal Contract	Contract Number or Code
PHC-E-195	***Clamps: adjustable (pair)***	No	

Functional Specifications	F-style clamps, length of bar approximately 200mm, reach of jaws at least 60mm. Bar at least 5mm x 20mm. Serrated. No clutch levers.<br>Acme-threaded screw. Handle of solid wood or polyethylene.<br>Swivel head on lower jaw. Replaceable non-marring pads both jaws.
Applicable Standards	
Performance Specifications	Expected service life 10 years.
Accessories	
Warranties	1 year.
Instructions to Suppliers	
Note to Procurement	

Catalogue Number	Item Name	Available on Transversal Contract	Contract Number or Code
PHC-E-196	***Drill, cordless***	No	

Functional Specifications	Cordless 18Volt drill, 2-speed with hammer-drill function.<br>Lithium-ion battery – at least 2 amp-hours.<br>Forward/reverse switch. Trigger-operated variable speed control. Gearbox least 2-speed, slide – or lever-selectable.<br>Torque at least 15Nm. Adjustable clutch.<br>No load speed on high: minimum 1500 rpm.<br>Chuck 13mm.
Applicable Standards	
Performance Specifications	Expected service life 10 years.
Accessories	Charge cord (with power cord). 2 batteries, robust carry case, accessory handle.
Warranties	2 years.
Instructions to Suppliers	
Note to Procurement	

Catalogue Number	Item Name	Available on Transversal Contract	Contract Number or Code
PHC-E-197	***Drill bit set***	No	

Functional Specifications	Set of good-quality drill bits, suitable for metal, wood and composite materials.<br>Sizes: 2-12 mm, in 0,5mm increments. Additionally three masonry bits in 6mm, 8mm, and 10mm sizes.
Applicable Standards	
Performance Specifications	
Accessories	Carry case.
Warranties	N/A.
Instructions to Suppliers	
Note to Procurement	

Catalogue Number	Item Name	Available on Transversal Contract	Contract Number or Code
PHC-E-069	***Extension cable: electrical, general purpose, 3m***	No	
PHC-E-070	***Extension cable: electrical, general purpose, 5m***	No	
PHC-E-071	***Extension cable: electrical, general purpose, 10m***	No	
PHC-E-211	***Extension cable: on reel, 20m***	No	

Functional Specifications	3-core, 2,5mmsq per core. Moulded SABS compliant 15-A three pin plug one end, moulded double 3-pin 15A female socket connector at the other. Can be Janus type or flat connector. Live and neutral openings to be shuttered.<br>Available in 3m, 5m, 10m lengths.<br>The reel-mounted extension must have a retraction mechanism for coiling it back onto a reel, enclosed inside a plastic/metal container. The plastic/metal container must have at least two switched 15-amp three prong sockets.
Applicable Standards	Must meet or exceed SANS 1574.
Performance Specifications	5-year design life.
Accessories	None.
Warranties	1 year.
Instructions to Suppliers	None.
Note to Procurement	None.

Catalogue Number	Item Name	Available on Transversal Contract	Contract Number or Code
PHC-E-078	***File, flat, combined***	No	

Functional Specifications	Forged hardened steel. Length about 350mm.<br>Preferably Bastard Cut one face, Second Cut on other face.<br>Complete with securely attached polypropylene handle.
Applicable Standards	SANS 1172: 2008.
Performance Specifications	Expected service life 5 years.
Accessories	
Warranties	1 year.
Instructions to Suppliers	
Note to Procurement	

Catalogue Number	Item Name	Available on Transversal Contract	Contract Number or Code
PHC-E-198	***Foam cutter, electric***	No	

Functional Specifications	Electric foam cutter saw (not electric carving knife and not hot-wire types). Reciprocating twin blades. Depth of cut in foam 300mm. Power at least 350Watt. Must be suitable for left- or right-handed use.
Applicable Standards	Double-insulated.
Performance Specifications	Expected service life 10 years.
Accessories	1 set of blades.<br>Sole plate.<br>Blade guide.
Warranties	2 years.<br>Spares availability 10 years.
Instructions to Suppliers	
Note to Procurement	Some manufacturers do not supply a blade guide as standard. Please ensure that a 300mm blade guide is included in the quote and pricing.

Catalogue Number	Item Name	Available on Transversal Contract	Contract Number or Code
PHC-E-199	***Frying pan, electric***	No	

Functional Specifications	For splint making in PHC facilities – size limited to upper-limb splints.<br>Square/rectangular shape. At least 300x300mm, larger preferred. Depth not critical. On/off switch, detachable power cord with temperature control, pilot light. At least 1500 Watts.<br>Non-stick coating preferred. Heat-resistant handles.
Applicable Standards	
Performance Specifications	Expected service life 10 years.
Accessories	
Warranties	2 years preferred.
Instructions to Suppliers	
Note to Procurement	

Catalogue Number	Item Name	Available on Transversal Contract	Contract Number or Code
PHC-E-080	***Funnel, plastic, medium***	No	

Functional Specifications	Medium-sized plastic funnel with long spout (for adding oil to machines).<br>Must be oil- and solvent resistant.
Applicable Standards	
Performance Specifications	
Accessories	
Warranties	N/A.
Instructions to Suppliers	
Note to Procurement	

Catalogue Number	Item Name	Available on Transversal Contract	Contract Number or Code
PHC-E-081	***Hacksaw, all-metal***	No	

Functional Specifications	Metal handle and frame.<br>Alloy handle. Tempered steel frame, chrome-plated, wing-nut tensioning system.<br>No lateral deflection of frame when under tension.<br>Handle joined to frame with a recessed threaded bolt.
Applicable Standards	SANS 1219:2007.
Performance Specifications	Expected service life 10 years.
Accessories	
Warranties	1 year.
Instructions to Suppliers	
Note to Procurement	

Catalogue Number	Item Name	Available on Transversal Contract	Contract Number or Code
PHC-E-082	***Hacksaw blades, assorted***	No	

Functional Specifications	Bi-metal blades, hardened teeth, 300mm length.<br>2 each in 18, 24, 32 teeth per inch.
Applicable Standards	SANS 1219:2007.
Performance Specifications	
Accessories	
Warranties	N/A.
Instructions to Suppliers	
Note to Procurement	

Catalogue Number	Item Name	Available on Transversal Contract	Contract Number or Code
PHC-E-072	***Hammer: claw, approximately 450gm***	No	

Functional Specifications	Solid hickory wood or fibreglass handle inserted into head and locked with wedges.<br>Head: forged, smooth head, rip claw. Approximately 450gm.
Applicable Standards	Wooden Handle: SANS 292:2017.<br>Head: SANS 387:2014.
Performance Specifications	Expected service life 5 years.
Accessories	
Warranties	2 year.
Instructions to Suppliers	None.
Note to Procurement	None.

Catalogue Number	Item Name	Available on Transversal Contract	Contract Number or Code
PHC-E-200	***Hearing protector (muffs)***	No	

Functional Specifications	Hearing protection while using power tools.<br>Over-the-ear style. Thickly-padded ear cushions to permit use with goggles and spectacles.<br>Padded head band, should swivel to allow over-the-head or behind-the-head use.<br>Cushions must be replaceable.
Applicable Standards	
Performance Specifications	Must be cleanable and disinfectable.<br>Noise attenuation rating at least 27 decibels.<br>Expected service life 10 years.
Accessories	
Warranties	1 year.
Instructions to Suppliers	
Note to Procurement	

Catalogue Number	Item Name	Available on Transversal Contract	Contract Number or Code
PHC-E-201	***Heat gun, electric***	No	

Functional Specifications	Electric heat gun. Variable temperature and airflow approx. 300ºC to 600ºC.<br>Cool body. Must be able to stand upright for hands-free use.
Applicable Standards	Double-insulated.
Performance Specifications	Expected service life 10 years.
Accessories	Various nozzles to concentrate or disperse flow of hot air.
Warranties	2 years.
Instructions to Suppliers	
Note to Procurement	

Catalogue Number	Item Name	Available on Transversal Contract	Contract Number or Code
PHC-E-202	***Jigsaw, electric***	No	

Functional Specifications	D-handle type jigsaw. Pendulum cutting action. Tool-free blade change. Blower function to keep cutting line free of sawdust. Minimum 600 Watts. Variable speed control.
Applicable Standards	Double-insulated.
Performance Specifications	Expected service life 10 years.
Accessories	
Warranties	2 years.
Instructions to Suppliers	
Note to Procurement	

Catalogue Number	Item Name	Available on Transversal Contract	Contract Number or Code
PHC-E-203	***Jigsaw blades, set***	No	

Functional Specifications	Set of jigsaw blades in holder. Bi-metal. At least one each Rip, crosscut, fine cut and scroll cut in wood. Two metal-cutting blades.
Applicable Standards	
Performance Specifications	
Accessories	
Warranties	N/A.
Instructions to Suppliers	
Note to Procurement	There are two commonly used type of jigsaw blade: T-shank and U-shank. Ensure that the type procured is compatible with the jigsaw procured. Tool-free jigsaws almost always accept only t-shank types.

Catalogue Number	Item Name	Available on Transversal Contract	Contract Number or Code
PHC-E-068	***Ladder: aluminium***	No	

Functional Specifications	Heavy-duty Ladder to be manufactured from high grade aluminium. A-style ladder, with locking top hinges.<br>6 steps per side. Dual-purpose – can be used a 6-step double-sided A-frame, or 12-step single ladder.<br>Rust-proof Locking hinges.<br>Non-slip treads.
Applicable Standards	SANS 1304-2:209 part 3.
Performance Specifications	10-year design life.<br>Working load 110 – 130kg.
Accessories	None.
Warranties	1 year.
Instructions to Suppliers	None.
Note to Procurement	None.

Catalogue Number	Item Name	Available on Transversal Contract	Contract Number or Code
PHC-E-204	***Measuring rule, folding***	No	

Functional Specifications	Fibreglass or wood multi-section folding rule, hinged. Extended length approx. 2 metres, folded length approx. 12cm. Spring hinges, lock in extended and folded position.<br>Graduations imperial and metric.
Applicable Standards	
Performance Specifications	Expected service life 10 years.
Accessories	
Warranties	1 year.
Instructions to Suppliers	
Note to Procurement	

Catalogue Number	Item Name	Available on Transversal Contract	Contract Number or Code
PHC-E-210	***Multi-plug***	No	

Functional Specifications	Adaptor for connecting several electrical machines to a single wall power outlet.<br>1,5 to 2m cable, ending in moulded SABS approved 15Amp plug.<br>Multi-plug body to be made of robust plastic.<br>Integral overload protection, with reset button/switch.<br>Integral surge protector – replaceable if blown.<br>At least 3 x 15 Amp individually switched outlets, with illuminated switches.<br>At least three SANS 164-2 sockets.<br>At least 1 USB 2Amp socket preferred.<br>Either through holes or a rear snap-on bracket for mounting to a surface.
Applicable Standards	SANS 164, SANS 60884-1:2013.
Performance Specifications	Expected service life 10 years.
Accessories	Bracket for wall-mounting, if applicable to model.
Warranties	1 year.
Instructions to Suppliers	
Note to Procurement	

Catalogue Number	Item Name	Available on Transversal Contract	Contract Number or Code
PHC-E-077	***Pliers set***	No	

Functional Specifications	1 Long-nose pliers, 180mm serrated jaws.<br>1 combination pliers (lineman), 180mm.<br>1 side-cutter, 180mm.<br>1 slip-joint plier 180-200mm.<br>Forged steel, induction-hardened jaws. Non-slip rubber grips.
Applicable Standards	SANS 1120: 2008.
Performance Specifications	Expected service life 10 years.
Accessories	
Warranties	1 year.
Instructions to Suppliers	
Note to Procurement	

Catalogue Number	Item Name	Available on Transversal Contract	Contract Number or Code
PHC-E-083	***Pruning saw, medium***	No	

Functional Specifications	Metal bow saw, with lever-type tensioning system.<br>Carbon steel blade with large gullets for pruning wet wood.
Applicable Standards	SANS 1284:2014.
Performance Specifications	Expected service life 10 years.
Accessories	
Warranties	2 years on frame.
Instructions to Suppliers	
Note to Procurement	

Catalogue Number	Item Name	Available on Transversal Contract	Contract Number or Code
PHC-E-084	***Pruning shears, bypass, heavy-duty***	No	

Functional Specifications	Heavy-duty by-pass type pruning shears. Approx. 200mm.<br>Adjustable lock-nut to control gap between blades.<br>Forged aluminium handles. Replaceable carbon-steel blades.<br>Spring self-opening mechanism. Sliding lock to hold the closed position.
Applicable Standards	
Performance Specifications	Expected service life 5 years.
Accessories	1 spare set of blades and 1 spare spring.
Warranties	1 year.
Instructions to Suppliers	
Note to Procurement	

Catalogue Number	Item Name	Available on Transversal Contract	Contract Number or Code
PHC-E-205	***Punch, revolving***	No	

Functional Specifications	Heavy-duty revolving- head hole punch, for leather, webbing and other fabrics.<br>Non-slip grip. Forged steel body. Carbon steel hollow punches. Brass anvil.<br>Hole sizes 2,0, 2,5, 3.0, 3,5, 4,0, 4,5 mm. Edge margin 3,8mm.
Applicable Standards	
Performance Specifications	Expected service life 10 years.
Accessories	
Warranties	2 years.
Instructions to Suppliers	
Note to Procurement	

Catalogue Number	Item Name	Available on Transversal Contract	Contract Number or Code
PHC-E-206	***Punch, eyelet***	No	

Functional Specifications	Combination single-hole punch and eyelet setting tool.<br>Self-opening jaws.
Applicable Standards	
Performance Specifications	Expected service life 10 years.
Accessories	
Warranties	
Instructions to Suppliers	
Note to Procurement	

Catalogue Number	Item Name	Available on Transversal Contract	Contract Number or Code
PHC-E-156	***Riveter, hand***	No	

Functional Specifications	Rolled steel or forged alloy handles, with plastic grips. 250mm.<br>Hardened steel jaws. Hardened steel cross-pin.<br>For aluminium and steel rivets.
Applicable Standards	
Performance Specifications	Expected service life 10 years.
Accessories	Nozzles for 3,2mm and 4,8mm rivets (other sizes not essential).
Warranties	1 year.
Instructions to Suppliers	
Note to Procurement	

Catalogue Number	Item Name	Available on Transversal Contract	Contract Number or Code
PHC-E-086	***Scraper, paint, medium***	No	

Functional Specifications	Flexible tempered tool steel blade, approx. 35x 60mm Blade may be straight or flared.<br>Riveted wood or moulded polypropylene handle.<br>Rust-resistant steel.
Applicable Standards	
Performance Specifications	
Accessories	
Warranties	N/A.
Instructions to Suppliers	
Note to Procurement	

Catalogue Number	Item Name	Available on Transversal Contract	Contract Number or Code
PHC-E-073	***Screwdriver set***	No	

Functional Specifications	1 slotted head (flat) large, long-reach.<br>1 slotted head (flat) medium.<br>1 slotted head (flat) electrician.<br>1 slotted head (flat) stubby medium.<br>1 Philips No1.<br>1 Philips No2.<br>1 Pozidriv No 1.<br>1 Pozidriv No 2.<br>1 Square drive No 2.<br>Rubberised, non-slip handles.
Applicable Standards	Slotted head: SANS 110:2013.<br>Others: SANS 8764-1:2009, SANS 8764-2:2009.
Performance Specifications	Rust-resistant.<br>Expected service life 5 years.
Accessories	
Warranties	1 year.
Instructions to Suppliers	
Note to Procurement	

Catalogue Number	Item Name	Available on Transversal Contract	Contract Number or Code
PHC-E-079	**Sharpening stone, medium/fine combined**	No	

Functional Specifications	Aluminium oxide rectangular sharpening stone, about 250x75mm.<br>One medium face, one fine face.<br>Oil or water as lubricant.
Applicable Standards	
Performance Specifications	Expected service life 10 years.
Accessories	
Warranties	1 year.
Instructions to Suppliers	
Note to Procurement	

Catalogue Number	Item Name	Available on Transversal Contract	Contract Number or Code
PHC-E-074	***Shifting spanner: 0-40mm***	No	

Functional Specifications	Drop-forge hardened steel alloy.
Applicable Standards	SANS 1211: 2007.
Performance Specifications	Expected service life 10 years.
Accessories	
Warranties	1 year.
Instructions to Suppliers	
Note to Procurement	

Catalogue Number	Item Name	Available on Transversal Contract	Contract Number or Code
PHC-E-075	***Swivel-head spanner set***	No	

Functional Specifications	Drop-forged chrome vanadium. Combination open-end and swivel head, serves most functions of a spanner and a socket wrench.<br>Metric sizes 8,10,13,14,17,19, 21mm.
Applicable Standards	SANS 1112: 2008.
Performance Specifications	Expected service life 10 years.
Accessories	
Warranties	1 years.
Instructions to Suppliers	
Note to Procurement	

Catalogue Number	Item Name	Available on Transversal Contract	Contract Number or Code
PHC-E-207	***Tape measure: industrial***		

Description	Retractable, industrial-duty tape measure, with metric and imperial markings. Blade 5m X 25mm.
Use	For measuring, e.g. distance for standardized movement tests, height, wheelchair biometric assessments.
Clinical or User Specifications	Retract button should not jam after multiple uses.
Accessories	None.
Care	
Supplier Warranty	2 years (excluding deliberate or accidental damage).

Catalogue Number	Item Name	Available on Transversal Contract	Contract Number or Code
PHC-E-157	***Tool box***	No	

Functional Specifications	Heavy-duty all-metal construction. At least three tiers. Lower tiers may be pull-out drawers on ball-bearing slides, or the box may be of cantilevered design, where the lower tiers are exposed as the two halves of the top tier are moved apart.<br>Closed-cell foam lining for each tier, to reduce noise and wear on the metal surfaces.<br>Lockable.
Applicable Standards	None.
Performance Specifications	Expected service life 5 years.
Accessories	
Warranties	1 year.
Instructions to Suppliers	
Note to Procurement	

Catalogue Number	Item Name	Available on Transversal Contract	Contract Number or Code
PHC-E-208	***Utility knife, retractable blade***	No	

Functional Specifications	Utility knife (also known as box-cutter). Cast alloy or plastic non-folding body, ergonomic shape, blade storage in the handle. Length approx. 13cm. Non-snap-off blades.
Applicable Standards	
Performance Specifications	Expected service life 10 years.
Accessories	Box of 50 standard utility knife blades (non-snap-off).
Warranties	1 year.
Instructions to Suppliers	
Note to Procurement	

Catalogue Number	Item Name	Available on Transversal Contract	Contract Number or Code
PHC-E-085	***Voltage tester, contactless***	No	

Functional Specifications	Contactless voltage tester for safely determining presence of AC mains current in a wire, switch, outlet.<br>50-300 V minimum range. At least 50-60Hz frequency.<br>Insulated plastic body.<br>Audible and visible voltage indicator.
Applicable Standards	
Performance Specifications	Expected service life 10 years.
Accessories	
Warranties	1 year.
Instructions to Suppliers	
Note to Procurement	

Catalogue Number	Item Name	Available on Transversal Contract	Contract Number or Code
PHC-E-076	***Water-pump pliers***	No	

Functional Specifications	Drop-forged chrome vanadium. Medium size. Serrated straight or curved jaws. Groove-joint. Nonslip coated handles.
Applicable Standards	SANS 1120: 2008.
Performance Specifications	Expected service life 5 years.
Accessories	
Warranties	1 year.
Instructions to Suppliers	
Note to Procurement	

Catalogue Number	Item Name	Available on Transversal Contract	Contract Number or Code
PHC-E-209	***Workbench, folding, portable***	No	

Functional Specifications	Powder-coated rolled or tubular metal frame, which can be opened or closed. When open, the frame is locked in position – positive action is needed to close the frame. The working surface (top) consists of two pieces of laminated board. One piece is fixed, the other can be moved backward and forward by rotating two cranked handles. The boards are drilled at intervals with 19mm holes, to accept plastic "bench dogs". A workpiece can be laid flat on the top, and either clamped to the top with clamps, or secured between two sets of bench dogs. Work can also be clamped vertically between the boards, which function like vice jaws.
Applicable Standards	
Performance Specifications	Expected service life 10 years.<br>The bench must support at least 75kg when open.
Accessories	Set of 4 plastic bench dogs.
Warranties	1 year.
Instructions to Suppliers	
Note to Procurement	

Cleaning

Catalogue Number	Item Name	Available on Transversal Contract	Contract Number or Code
PHC-E-001	***Bin: pedal, 4,5l***	No	

Functional Specifications	All stainless steel construction.<br>No plastic in the lid-lifting mechanism.<br>All levers to be secured in position with circlips or split pins.<br>Usable with or without a liner.<br>Noise-deadening bumpers to reduce lid noise.
Applicable Standards	
Performance Specifications	At least 5000 lid openings.<br>Standard cleaning and disinfectant solutions.
Accessories	
Warranties	2 years.
Instructions to Suppliers	
Note to Procurement	

Catalogue Number	Item Name	Available on Transversal Contract	Contract Number or Code
PHC-E-002	***Bin: pedal, 20l***	No	

Functional Specifications	All stainless steel construction.<br>No plastic in the lid-lifting mechanism.<br>All levers to be secured in position with circlips or split pins.<br>Usable with or without a liner.<br>Noise-deadening bumpers to reduce lid noise.
Applicable Standards	
Performance Specifications	At least 10 000 lid openings.<br>Standard cleaning and disinfectant solutions.
Accessories	
Warranties	2 years.
Instructions to Suppliers	
Note to Procurement	

Catalogue Number	Item Name	Available on Transversal Contract	Contract Number or Code
PHC-E-003	***Bin: wall-mounted, refuse bag holding unit***	No	

Functional Specifications	Consists of:<br>White epoxy-coated wall bracket, pre-drilled for wall mounting<br>Rectangular white epoxy-coated or rubberised metal frame, approximately 40cm wide x 35cm deep, securely attached to wall bracket, but offset sufficiently to allow plastic bag to be draped over complete circumference of frame<br>Second frame, white epoxy-coated or chromed, to fit snugly over the mounted frame in order to secure the plastic refuse bag. Preferably should have a small lip to prevent slipping down. May be hinged to the inner ring, or separate.
Applicable Standards	SANS 778 paragraph 5.2, proof of compliance must be submitted.
Performance Specifications	Must support weight of 40kg without deflection when mounted on wall.<br>Cleanable with standard cleaning and disinfectant solutions.
Accessories	All wall-mounting hardware (stainless steel screws).
Warranties	2 years warranty.
Instructions to Suppliers	Supplier to deliver and install.
Note to Procurement	

Catalogue Number	Item Name	Available on Transversal Contract	Contract Number or Code
PHC-E-004	***Bin: sanitary towel***	No	

Functional Specifications	All stainless steel construction.<br>Well-fitting, hinged lid.<br>Wall-mounted, but can be floor-mounted, depending on space available.
Applicable Standards	
Performance Specifications	At least 5 000 lid openings.<br>Standard cleaning and disinfectant solutions.
Accessories	All wall-mounting hardware (stainless steel screws).
Warranties	2 years.
Instructions to Suppliers	
Note to Procurement	Suitable plastic bag to be available for this item.

Catalogue Number	Item Name	Available on Transversal Contract	Contract Number or Code
PHC-E-005	***Bin: sharps***	No. Usually supplied by a waste management service provider.	

Functional Specifications	Puncture-proof plastic (polypropylene), yellow with red contrast.<br>Tamper-proof, leak-proof. Lid cannot be re-opened once closed.<br>Spill-proof (entry port should not permit contents to fall out if the bin is knocked over).<br>Labelled "Danger, contaminated sharps".<br>International hazardous waste symbol.
Applicable Standards	SANS 452:2008.
Performance Specifications	Cleanable with standard cleaning and disinfectant solutions.<br>Must withstand Hot Water Wash (90°C).
Accessories	Bracket to hold securely to wall, pole or counter-top.
Warranties	2 years.
Instructions to Suppliers	Ensure that a suitable supporting bracket is provided.
Note to Procurement	

Catalogue Number	Item Name	Available on Transversal Contract	Contract Number or Code
PHC-E-006	***Broom: interior use***	No	

Functional Specifications	Household broom. Head approx. 45cm wide. Long soft synthetic bristle.<br>Handle attached to head via metal fixing – not plastic threaded connector.<br>Handle solid wood or aluminium – not plastic-coated thin mild steel.<br>Robust hanging hook or hole.
Applicable Standards	SANS 77:2008 wooden handles for brooms.
Performance Specifications	
Accessories	None.
Warranties	
Instructions to Suppliers	
Note to Procurement	Reject if a magnet is attracted to the handle.

Catalogue Number	Item Name	Available on Transversal Contract	Contract Number or Code
PHC-E-007	**Buckets: colour-coded**	No	

Functional Specifications	Medium-duty polypropylene buckets, approximately 20l.<br>Rectangular, with folding handle.<br>Colours: red, blue, green, black.
Applicable Standards	
Performance Specifications	Cleanable with standard cleaning and disinfectant solutions.<br>Chemical resistant.
Accessories	
Warranties	1 year.
Instructions to Suppliers	
Note to Procurement	Size to fit janitor trolley.

Catalogue Number	Item Name	Available on Transversal Contract	Contract Number or Code
PHC-E-010	***Dust pan and brush set***	No	

Functional Specifications	Heavy-duty polypropylene dust pan, large size. Hanging hole in handle.<br>Heavy-duty soft-bristle plastic brush with handle. Hole in handle for hanging.<br>Domestic quality not suitable.
Applicable Standards	
Performance Specifications	Cleanable with standard cleaning and disinfectant solutions.<br>Chemical resistant.
Accessories	
Warranties	1 year.
Instructions to Suppliers	
Note to Procurement	

Catalogue Number	Item Name	Available on Transversal Contract	Contract Number or Code
PHC-E-015	***Mop: red***	No	
PHC-E-016	***Mop: blue***	No	
PHC-E-017	***Mop: black***	No	

Functional Specifications	Standard wooden handle. Painted Red, Blue or Black. Non-chip, water-resistant paint.<br>Head: metal, rust-free. Long hair mop. Handle must attach to head-part securely.
Applicable Standards	None.
Performance Specifications	
Accessories	None.
Warranties	6 months.
Instructions to Suppliers	
Note to Procurement	Procure equal numbers of the different colours.

Catalogue Number	Item Name	Available on Transversal Contract	Contract Number or Code
PHC-E-019	***Mop: static head, complete***	No	

Functional Specifications	Head 60cm wide. ABS/polypropylene base, aluminium frame.<br>Microfiber sleeve, 60cm, washable.<br>Handle approx. 1,2m long. Secure swivelling connection between head and handle.<br>Solid wood or aluminium one-piece handle – not thin steel covered with plastic.
Applicable Standards	None.
Performance Specifications	
Accessories	Microfibre sleeve for static head mop PHC-C-210.
Warranties	1 year.
Instructions to Suppliers	
Note to Procurement	Reject if a magnet is attracted to the handle.

Catalogue Number	Item Name	Available on Transversal Contract	Contract Number or Code
PHC-E-020	***Paper towel dispenser: folded towels***	RT 14-2016	47131710-00003

Functional Specifications	Wall-mounted polycarbonate dispenser for folded paper towels.
Applicable Standards	
Performance Specifications	Cleanable with standard cleaning and disinfectant solutions.<br>Chemical resistant.<br>Expected service life: 15 years.
Accessories	All wall-mounting hardware (stainless steel screws).
Warranties	2 years on all components.
Instructions to Suppliers	Supplier to deliver and install.
Note to Procurement	Suitable folded paper towels available on RT 14 as Item 14111703-0003.

Catalogue Number	Item Name	Available on Transversal Contract	Contract Number or Code
PHC-E-021	***Paper towel dispenser: roll***	No	

Functional Specifications	Dispenser of paper towels for drying hands.<br>Stainless steel U-shaped metal frame minimum 3mm thickness, with stainless steel tubular cross-member, to take standard width perforated paper roller towels. Unit must accommodate large diameter rolls (not industrial sizes). Cross-member to have positive retaining system, to prevent slippage and displacement of roll. Pre-drilled for wall mounting.
Applicable Standards	
Performance Specifications	Cleanable with standard cleaning and disinfectant solutions.<br>Expected service life: 15 years.
Accessories	All wall-mounting hardware (stainless steel screws).
Warranties	5 years on all components.
Instructions to Suppliers	Supplier to deliver and install.
Note to Procurement	Suitable folded paper towels available on RT 14 as Item 14111703-0003.

Catalogue Number	Item Name	Available on Transversal Contract	Contract Number or Code
PHC-E-014	***Soap dispenser***	No	

Functional Specifications	Simple, heavy-duty stainless steel cage-type unit, **without** hinged lever arm.<br>Cage to be securely welded to stainless steel wall-mounting plate, with 3-4cm projection from front-surface of plate. May be canted forwards, for easier elbow access to pump mechanism on bottle.<br>Cage must accept non-proprietary standard, replaceable refillable, pump bottles, approximately 65mm diameter, about 500ml volume.<br>Mounting plate to be pre-drilled for wall mounting.
Applicable Standards	
Performance Specifications	Cleanable with standard cleaning and disinfectant solutions.<br>Expected service life: 15 years.
Accessories	Two empty bottles. All wall-mounting hardware (stainless steel screws).
Warranties	5 years on metal components.
Instructions to Suppliers	Supplier to deliver and install.
Note to Procurement	Suitable bottles to be procured as spares, in series with this item.

Catalogue Number	Item Name	Available on Transversal Contract	Contract Number or Code
PHC-E-022	***Spray bottle: 750ml***	No	

Functional Specifications	Simple trigger-action mechanism, on a robust plastic bottle.<br>For use with bilk-purchased liquid detergents and window-cleaning solutions.<br>Bottle should not be of the thin, transparent, blow-moulded type.<br>Spray pattern is adjustable at the nozzle.
Applicable Standards	None.
Performance Specifications	
Accessories	None.
Warranties	
Instructions to Suppliers	
Note to Procurement	

Catalogue Number	Item Name	Available on Transversal Contract	Contract Number or Code
PHC-E-023	***Spray bottle: 2l***	No	

Functional Specifications	Robust plastic bottle. Cap has a built-in pump to pressurise the bottle and its contents.<br>Trigger –activated spray release. Spray pattern is adjustable at the nozzle.
Applicable Standards	None.
Performance Specifications	
Accessories	None.
Warranties	
Instructions to Suppliers	
Note to Procurement	

Catalogue Number	Item Name	Available on Transversal Contract	Contract Number or Code
PHC-E-024	***Spray bottle: 10l backpack***	No	

Functional Specifications	Heavy-duty plastic bottle, with harness for easy carrying in the user's back.<br>Built in pump to pressurise the bottle and its contents. The pump may be built into the cap, or be lever-operated, for continuous use without removing from the carrying position.<br>Pressure hose approx. 1m with a rigid non-rusting metal wand (aluminium or Stainless steel).<br>The wand has a trigger release, and an adjustable spray nozzle.<br>Generally used for application of weed-killer, insecticide or fertiliser solutions.
Applicable Standards	None.
Performance Specifications	
Accessories	Carrying harness.
Warranties	1 year.
Instructions to Suppliers	
Note to Procurement	

Catalogue Number	Item Name	Available on Transversal Contract	Contract Number or Code
PHC-E-150	***Squeegee***	No	

Functional Specifications	Double-sided aluminium headpiece, approx. 30cm wide. Replaceable live rubber edge on one side, replaceable cellulose sponge in gauze wrapper on the other.<br>Handle: two-piece aluminium, telescopic. Locking mechanism must not allow head to rotate, in the extended or retracted positions.<br>Extended length approx. 1,2m.
Applicable Standards	None.
Performance Specifications	
Accessories	Spare sponge and rubber edge.
Warranties	1 year.
Instructions to Suppliers	
Note to Procurement	Procure with one set of replacement parts. After replacing the rubber edge and sponge once, the whole squeegee should be replaced, so no spares are necessary for this item.

Catalogue Number	Item Name	Available on Transversal Contract	Contract Number or Code
PHC-E-025	***Dispenser: toilet roll***	RT 14-2016	47131710-00000

Functional Specifications	Wall-mounted, tamper-resistant toilet roll holder.<br>All stainless steel construction.<br>Capacity 2 rolls.
Applicable Standards	
Performance Specifications	Cleanable with standard cleaning and disinfectant solutions.<br>Expected service life: 15 years.
Accessories	All wall-mounting hardware (stainless steel screws).
Warranties	2 years.
Instructions to Suppliers	Supplier to deliver and install.
Note to Procurement	

Catalogue Number	Item Name	Available on Transversal Contract	Contract Number or Code
PHC-E-030	***Trolley: janitor***	No	

Functional Specifications	The trolley holds a large refuse bad, held in the open position on the frame by a retainer. There is a sealing lid over the mouth of the bag, to prevent spillage and odours escaping.<br>Alongside the receptacle are three shelves. The top shelf is sunken, to accommodate spray bottles and other containers. The middle and lower shelves are enclosed by a rail on all sides, and house cloths and brushes.<br>The free edge of the top shelf has hooks or snap holders, into which mop handles can be secured when the unit is moving. Space for three mops (or mop/broom combinations).<br>The floor of the trolley extends beyond the shelves, to provide a support for two heavy-duty buckets.<br>The trolley is castor-mounted for easy movement into and out of rooms. The trolley shall be fitted with 75mm diameter castors.<br>Castors must comply with the latest issue of SANS 621, proof of compliance must be submitted.<br>Where castors are fitted into steel tubular legs, the tube shall be of wall thickness not less than 2,0mm and castors shall be fixed to the tube by one of the following methods:<br>a. Solid plug (long) complying with SANS 621 subsection 3.5.6 or<br>b. Screwed into 35mm long sleeves welded into the tubular members and locked in an acceptable manner<br>c. Rubber or plastic expanding sleeves for fitting castors are not acceptable.
Applicable Standards	SANS 778 paragraph 5.2, Proof of compliance must be submitted.<br>To comply with SANS 896 specifications.<br>SANS 621, proof of compliance must be submitted.
Performance Specifications	Cleanable with standard cleaning and disinfectant solutions.<br>Expected service life: 15 years for trolley body.
Accessories	Available separately as Cleaning Equipment and Cleaning Consumables.
Warranties	2 years.
Instructions to Suppliers	
Note to Procurement	Replacement linen receptacle RT 24-12-029 in series with this item, to use interchangeably with, or to replace, original receptacle.

Cold chain

Catalogue Number	Item Name	Available on Transversal Contract	Contract Number or Code
PHC-E-035	***Vaccine cooler box***	No	
PHC-C-203	***Water packs for vaccine cool box***	No	

Functional Specifications	Specifications suitable for a cool box for a mixed outreach programme, or for use within a PHC facility.<br>Material: external polyethylene, internal polyethylene or polystyrene, insulation polyurethane.<br>Maximum external dimensions 30x30x30cm.<br>Insulation thickness minimum 28mm.<br>Storage volume approximately 2l.
Applicable Standards	Must comply with, and be pre-qualified on, WHO PQS E004 insulated containers. Proof to be supplied.
Performance Specifications	Cool life at 43° C: 24 hours.
Accessories	Adequate number of water packs to achieve cool life rating.<br>2 sets of packs, to allow one set to be in use while the other is being chilled.<br>Water packs for vaccine cooler box PHC-C-203.
Warranties	2 years.
Instructions to Suppliers	
Note to Procurement	Replacement water packs to be procured in series with this item.<br>There are 3 standard water pack sizes, depending on the type and size of cool box. If possible, standardise on one size, for ease of procurement. Do not purchase pre-filled water packs, as these may freeze the vaccine. Freezing destroys most vaccines.

Catalogue Number	Item Name	Available on Transversal Contract	Contract Number or Code
PHC-E-042	***Data logging thermometer***	No	

Functional Specifications	To be installed outside, but next to, the refrigerator, with probe and cable of reasonable length to reach inside fridge.<br>Must have a cradle or port for external communication/connectivity. In case of a cradle, one must be supplied per facility. In case of a port, the relevant connecting cable must be supplied, one per facility.<br>Ideally has audible as well as visual alarm.<br>Preferably automatically generates monthly report.
Applicable Standards	Must comply with, and be pre-qualified on, WHO PQS E006/TH02 " User programmable temperature data logger multi-use" (proof to be supplied).
Performance Specifications	Must store readings at pre-defined intervals, and keep readings for one month before over-writing.<br>Must allow user to scroll through previous readings within the month.<br>Display must indicate an alarm condition, if temperature boundaries have been exceeded.<br>Must be cleanable and disinfectable with standard materials.<br>Ideally, should be able to be connected via a network, to a central control location (provide details of connectivity and any hardware/software requirements).
Accessories	Temperature probe.
Warranties	2 years.
Instructions to Suppliers	To be delivered with agreed standard configuration.<br>Includes installation and training.<br>If any specific hardware or software is required for downloading or reading stored readings or report, this must be supplied at no additional cost.
Note to Procurement	The thermometer has a 2-year service life. Used with vaccine and medicine refrigerators. A 2-year planned replacement cycle must be catered for.

Catalogue Number	Item Name	Available on Transversal Contract	Contract Number or Code
PHC-E-153	***Thermometer: refrigerator***	No	

Functional Specifications	Analogue bi-metal mechanism.<br>Stainless steel case, sealed.<br>Placed inside the refrigerator.
Applicable Standards	
Performance Specifications	At least -20°C to +20°C.<br>Non-rusting.
Accessories	
Warranties	2 years.
Instructions to Suppliers	
Note to Procurement	

Catalogue Number	Item Name	Available on Transversal Contract	Contract Number or Code
PHC-E-114	***Room thermometer***	No	

Functional Specifications	Wall-mounted electronic thermometer unit.<br>Large LCD display showing maximum and minimum temperature.<br>Powered by button cell or non-corrosive battery.
Applicable Standards	None.
Performance Specifications	Sampling rate at least every hour.<br>Preferably the day of week or date/time can be set.<br>Re-set button for daily readings.<br>Minimum battery life: 1 year.
Accessories	Mounting bracket or fixing for wall mounting. Must be detachable from wall for battery change.
Warranties	2 years.
Instructions to Suppliers	None.
Note to Procurement	None.

Fire safety

Catalogue Number	Item Name	Available on Transversal Contract	Contract Number or Code
PHC-E-043	***Fire extinguisher***	No	

Functional Specifications	Fire extinguisher must be suitable for Class A, B, and C fires.<br>Type of extinguisher (water type, foam type, dry chemical powder type, or CO2 type) depends on the recommendation of the local fire department.<br>Number and location of extinguishers is laid out in SANS 10400, but a fire extinguisher typically should be located within a 10m radius, and the radii of nearby extinguishers should overlap.
Applicable Standards	SANS 10400 National Building Regulations.<br>SANS 1910:2009 Portable refillable fire extinguishers.<br>SANS 1567 CO2 fire extinguishers.<br>SANS 10105-1 and SANS 1475-1 control, inspection and maintenance of fire extinguishers.
Performance Specifications	4,5 kg size.
Accessories	None.
Warranties	2 years.
Instructions to Suppliers	To be wall-mounted and signposted in accordance to SANS 10400.
Note to Procurement	The inspection and maintenance of extinguishers is the responsibility of the owner of the building, after installation.<br>Procurement of a maintenance contract is advisable.<br>Extinguishers will have to be retired at some point – make provision for replacement.<br>Note that where there is no mains water supply, several 9kg water type extinguishers may be required per floor.

Catalogue Number	Item Name	Available on Transversal Contract	Contract Number or Code
PHC-E-044	***Fire hose on reel***	No	

Functional Specifications	Reinforced fire-fighting hose, 30m, on rotating reel with hub attached to water supply.<br>Hose complete with approved nozzle.<br>Number of hoses required is determined by the fire loading of the building. Each hose can cover a maximum radius of its own length. The radiuses of hoses should overlap, to provide total coverage.
Applicable Standards	Hose: SANS 1086 or EN694.<br>Reel: SANS 543.<br>Installation: SANS 10400 National Building Regulations Part W.<br>SANS 10105-2: 2010.
Performance Specifications	Location, installation and testing, must be to the satisfaction of the Authority Having Jurisdiction (usually the local fire department).<br>The hose and all couplings must comply with the burst rating for the intended use.
Accessories	Fitted with approved nozzle.
Warranties	2 years.
Instructions to Suppliers	
Note to Procurement	Fire hoses have an increased risk of failure after a 10-year service life. A replacement programme should be in place.

Catalogue Number	Item Name	Available on Transversal Contract	Contract Number or Code
PHC-E-045	**Smoke alarm**	No	

Functional Specifications	Electronic ceiling-mounted, battery-operated unit, with low quiescent current and low battery drain.<br>Detects smoke via opto-electronic sensor.<br>Built-in electronic siren, approximately 110 dB.<br>LED light(s) indicating active state and level of battery charge.<br>Ideally should have radio frequency linkage between nearby units, such that if one unit is triggered, a nearby one also sounds the alarm, to maximise ability to attract attention.<br>Replace battery when fire extinguishers are serviced.
Applicable Standards	None.
Performance Specifications	
Accessories	Non-corrosive battery(ies).
Warranties	2 years.
Instructions to Suppliers	Supplier to install. Ceiling mounting mechanism must permit easy battery replacement.
Note to Procurement	One unit per room that is closed off in normal use.

Garden

Catalogue Number	Item Name	Available on Transversal Contract	Contract Number or Code
PHC-E-046	***Bin: general Waste, 240l, black, with wheels***	No	
PHC-E-047	***Bin: Healthcare Risk Waste, 140l, red, with wheels***	No	
PHC-E-048	***Bin: general Waste, 140l, green, with wheels***	No	

Functional Specifications	Mobile refuse bin.<br>Thermoplastic material, colour black.<br>Hinged lid.<br>Nominal capacity 240l or 140l.<br>Two wheels for manual handling.<br>Handles for use with mechanical lifting and tilting equipment.
Applicable Standards	SANS 1494: 2014(edition 2.00).<br>SANS 1301: 2012.
Performance Specifications	
Accessories	
Warranties	2 years.
Instructions to Suppliers	
Note to Procurement	

Catalogue Number	Item Name	Available on Transversal Contract	Contract Number or Code
PHC-E-052	**Broom: garden**	No	

Functional Specifications	Heavy -duty, large broom, wooden handle, stiff bristles.
Applicable Standards	None available.
Performance Specifications	
Accessories	None.
Warranties	1 year.
Instructions to Suppliers	None.
Note to Procurement	None.

Catalogue Number	Item Name	Available on Transversal Contract	Contract Number or Code
PHC-E-050	***Edge trimmer/brush cutter: petrol, heavy duty***	No	

Functional Specifications	Professional-duty brush-cutter, 2-stroke petrol-driven, minimum 38 cc/1.6kW. To be supplied with blade, and interchangeable metal quick-load line head (non-plastic self-feeding type). Must be capable of extended operation – carburettor compensator or self-cleaning mechanism.
Applicable Standards	None.
Performance Specifications	Professional operation. Domestic models not suitable.
Accessories	Harness, safety glasses, hearing protectors, tool kit, manual.<br>Fuel mixing bottle.<br>100m recommended line.<br>Blade and line heads.
Warranties	Minimum 2 years, longer preferred. State expected MTBF.
Instructions to Suppliers	Specify price of all replaceable parts. Specify service types and intervals (user- and/or supplier-performed).<br>Supplier must deliver and train operators on use and maintenance.<br>Supplier to offer suitable steel wall-mounting bracket (to hold unit in vertical position).<br>Specify cost of annual service, and other available after-sales support.
Note to Procurement	2-stroke engine oil, spare filters and recommended blades and cutting line to be in series with this item.<br>Safety goggles and ear protectors also need frequent replacement.

Catalogue Number	Item Name	Available on Transversal Contract	Contract Number or Code
PHC-E-051	***Extension cable: electrical, garden***	No	

Functional Specifications	3-core, 2,5mmsq per core. 15m length, moulded SABS compliant 15-A three pin plug one end, moulded female CEE 7/3 (Shuko) connector at the other.
Applicable Standards	Must meet or exceed SANS 1574; CEE 7/3.
Performance Specifications	5-year design life.
Accessories	None.
Warranties	1 year.
Instructions to Suppliers	None.
Note to Procurement	None.

Catalogue Number	Item Name	Available on Transversal Contract	Contract Number or Code
PHC-E-053	***Fork: garden, 4-pronged***	No	

Functional Specifications	Heavy-duty, 4-pronged, garden fork.<br>All-steel construction. Steel grip.
Applicable Standards	SANS 390:2013.
Performance Specifications	
Accessories	None.
Warranties	1 year.
Instructions to Suppliers	None.
Note to Procurement	None.

Catalogue Number	Item Name	Available on Transversal Contract	Contract Number or Code
PHC-E-059	***Gardening set: 3-piece***	No	

Functional Specifications	A set consisting of hand trowel, hand fork, and hand cultivator.<br>Heavy duty steel construction – flimsy domestic models not suitable.<br>Medium length solid wood or polypropylene handles.
Applicable Standards	None.
Performance Specifications	
Accessories	None.
Warranties	2 years.
Instructions to Suppliers	None.
Note to Procurement	None.

Catalogue Number	Item Name	Available on Transversal Contract	Contract Number or Code
PHC-E-060	***Hedge shears***	No	

Functional Specifications	Heavy-duty steel blades continuous with steel handle. Blades may be straight or wavy.<br>Hardened steel pivot bolt (self-locking nut).<br>Handle covers wood or moulded polyethylene or nylon. No hollow aluminium handles.
Applicable Standards	None.
Performance Specifications	
Accessories	None.
Warranties	1 year.
Instructions to Suppliers	None.
Note to Procurement	None.

Catalogue Number	Item Name	Available on Transversal Contract	Contract Number or Code
PHC-E-061	***Hoe***	No	

Functional Specifications	Heavy-duty hoe, preferably toothed for weed removal.<br>All-steel construction.
Applicable Standards	None.
Performance Specifications	
Accessories	None.
Warranties	1 year.
Instructions to Suppliers	None.
Note to Procurement	None.

Catalogue Number	Item Name	Available on Transversal Contract	Contract Number or Code
PHC-E-062	***Hosepipe: garden***	No	

Functional Specifications	20mm diameter, 50m length. Standard brass female connector for threaded bibcock (not plastic quick-connector), with reducer bushing for use with 15mm taps, good-quality worm-drive clamps, high-quality quick connector at other end for attachment to sprays and spouts. to be supplied with one high-quality multi-pattern spray head.
Applicable Standards	SANS271:2005.
Performance Specifications	10-year design life, non-twist, non-kink, ultra-violet resisitant.
Accessories	Tap connector, quick-connector, multi-pattern spray head, wall-mounted hose holder, spare washer kit, hose joiner (for mending), two good quality worm-drive clamps.
Warranties	1 year.
Instructions to Suppliers	The envisaged wall-mounted hose holder must be durable (epoxy-coated steel), simple, of open construction, to permit the hose to be loosely coiled, off the ground. No reels, trolleys or self-retracting mechanisms.
Note to Procurement	A simple, wall-mounted powder-coated mild-steel hose holder (over which the hose can be coiled in loose loops), should be supplied with this item.

Catalogue Number	Item Name	Available on Transversal Contract	Contract Number or Code
PHC-E-063	***Lawn mower: electric, heavy duty***	No	

Functional Specifications	Rotary type lawnmower with cutting diameter of approximately 400mm.<br>Induction motor (not universal motor).<br>Steel large diameter wheels with ball bearings (no plastic wheels with metal sleeve bearings).<br>Chassis and body must be all-steel. Supplier to specify steel thickness. Grass catcher can be of durable ABS plastic.
Applicable Standards	Must meet or exceed SANS 60335-2-77 (IEC 60335-2-77:1996, MOD).
Performance Specifications	Must be suitable for large area (up to 4 000m^2).<br>At least 3000 Watt input power.
Accessories	Heavy duty replaceable 20metre cable with moulded male Shuko plug.<br>Grass catcher box.<br>User manual.
Warranties	Minimum 2 years on motor, chassis, body, wheels (longer preferred). Damage and abuse excluded.
Instructions to Suppliers	Specify price of all replaceable parts. Specify service types and intervals (user and/or supplier performed).<br>Supplier must deliver to site and train operators on use and maintenance.<br>Supplier must specify available after-sales support, with relevant pricing.
Note to Procurement	None.

Catalogue Number	Item Name	Available on Transversal Contract	Contract Number or Code
PHC-E-064	***Lawnmower: petrol, heavy duty***	No	

Functional Specifications	4-wheel, non-self-propelled. 3-wheeler can be proposed if capable of smooth finish cut on lawn. Wheels must be steel, large diameter with ball-bearing hubs. All steel chassis and body. Supplier to document steel thickness.<br>4-stroke engine, at least 6hp. Preferably snorkel type air cleaner. Will be used on premises exceeding 4000sq m, ground may be uneven.<br>Grass box may be robust ABS or metal.
Applicable Standards	None.
Performance Specifications	Professional operation. Domestic models not suitable.
Accessories	Safety glasses, hearing protectors, tool kit, manual.<br>Spare air cleaner.<br>Spare set of blades.<br>Suitable metal petrol container, 10 – 15l, with sealing lid.
Warranties	Minimum 2 years (longer preferred). State expected MTBF.
Instructions to Suppliers	Specify recommended grade of engine oil.<br>Specify price of all replaceable parts.<br>Specify service types and intervals (user and/or supplier performed).<br>Supplier must deliver, fill engine oil and petrol, and train operators on use and maintenance.<br>Specify cost of annual service, and other available after-sales support.
Note to Procurement	Recommended grade of engine oil, spare filters and recommended blades to be in serial with this item.<br>Safety goggles and ear protectors also need frequent replacement.

Catalogue Number	Item Name	Available on Transversal Contract	Contract Number or Code
PHC-E-065	***Pick mattock***	No	

Functional Specifications	Pick head mattock type: flat cutting blade at one end, and diamond point at other end.<br>Handle: plastic preferred.
Applicable Standards	SANS 341:2007. If wooden handle, handle must comply with SANS 268:2015.
Performance Specifications	Head weight approximately 2,3kg.
Accessories	None.
Warranties	1 year on head and handle.
Instructions to Suppliers	None.
Note to Procurement	None.

Catalogue Number	Item Name	Available on Transversal Contract	Contract Number or Code
PHC-E-054	***Rake: leaf and grass, heavy duty***	No	

Functional Specifications	Heavy-duty, long-tooth rake, suitable for gathering leaves or lawn clippings.<br>All-steel construction.
Applicable Standards	SANS 390:2013.
Performance Specifications	
Accessories	None.
Warranties	1 year.
Instructions to Suppliers	None.
Note to Procurement	None.

Catalogue Number	Item Name	Available on Transversal Contract	Contract Number or Code
PHC-E-055	***Rake: steel, heavy duty***	No	

Functional Specifications	Heavy-duty garden rake.<br>Steel handle.
Applicable Standards	SANS 390:2013.
Performance Specifications	
Accessories	None.
Warranties	1 year.
Instructions to Suppliers	None.
Note to Procurement	None.

Catalogue Number	Item Name	Available on Transversal Contract	Contract Number or Code
PHC-E-056	***Shovel: square mouth***	No	

Functional Specifications	Heavy-duty garden shovel.<br>All-steel construction. Steel grip.
Applicable Standards	SANS 284:2013.
Performance Specifications	
Accessories	None.
Warranties	1 year.
Instructions to Suppliers	None.
Note to Procurement	None.

Catalogue Number	Item Name	Available on Transversal Contract	Contract Number or Code
PHC-E-057	***Slasher***	No	

Functional Specifications	Tempered steel grass slasher. Wood or nylon handle.
Applicable Standards	None.
Performance Specifications	
Accessories	None.
Warranties	1 year.
Instructions to Suppliers	None.
Note to Procurement	None.

Catalogue Number	Item Name	Available on Transversal Contract	Contract Number or Code
PHC-E-058	***Spade, garden***	No	

Functional Specifications	Heavy-duty garden spade.<br>All-steel construction. Steel grip.
Applicable Standards	SANS 284:2013.
Performance Specifications	
Accessories	None.
Warranties	1 year.
Instructions to Suppliers	None.
Note to Procurement	None.

Catalogue Number	Item Name	Available on Transversal Contract	Contract Number or Code
PHC-E-066	***Watering can***	No	

Functional Specifications	10-15l, heavy-duty galvanised steel, with threaded spout and detachable rose.
Applicable Standards	None.
Performance Specifications	None.
Accessories	Threaded rose.
Warranties	1 year.
Instructions to Suppliers	None.
Note to Procurement	None.

Catalogue Number	Item Name	Available on Transversal Contract	Contract Number or Code
PHC-E-067	**Wheelbarrow**	No	

Functional Specifications	Heavy-duty concrete-type wheelbarrow, not DIY type.<br>Pan: metal preferred, but Polypropylene can be proposed.<br>Solid rubber wheel on steel rim.<br>Capacity: minimum 60l.
Applicable Standards	SANS 795:2014 Type 4 or 5.
Performance Specifications	5-year design life.
Accessories	None.
Warranties	1 year.
Instructions to Suppliers	None.
Note to Procurement	None.

Kitchen

Catalogue Number	Item Name	Available on Transversal Contract	Contract Number or Code
PHC-E-092	***Kettle: electric***	No	

Functional Specifications	All-stainless steel construction. Cordless, i.e. separate from base, which connects to power outlet via 1,5m cord with moulded 15A 3-pin SABS-approved plug.<br>Separate lid, not hinged.<br>Auto-shutoff. Boil-dry protection.<br>Heating element enclosed in separate chamber.<br>Rapid-boiling, 2000W.
Applicable Standards	SANS 60335 – applicable section.
Performance Specifications	5-year design life.
Accessories	None.
Warranties	1 year.
Instructions to Suppliers	None.
Note to Procurement	None.

Catalogue Number	Item Name	Available on Transversal Contract	Contract Number or Code
PHC-E-093	***Rack: dish***	No	
PHC-E-094	***Rack: cutlery***	No	

Functional Specifications	All-stainless steel construction. Countertop models, with stainless steel drip trays.
Applicable Standards	
Performance Specifications	5-year design life.
Accessories	None.
Warranties	1 year.
Instructions to Suppliers	None.
Note to Procurement	None.

Catalogue Number	Item Name	Available on Transversal Contract	Contract Number or Code
PHC-E-096	***Urn: electric***	No	

Functional Specifications	All stainless steel construction.<br>Thermostat to maintain water at desired temperature.<br>Metal spigot tap preferred.<br>Has safety cut-out and anti- boil dry. Pilot light.<br>Power cord at least 1,5m with moulded 15A 3-pin plug.
Applicable Standards	SANS 60335 applicable sections.
Performance Specifications	At least 20 l capacity.<br>5-year design life.
Accessories	
Warranties	2 years.
Instructions to Suppliers	State available spare parts and after-sales support.<br>State guaranteed minimum spares availability (years).
Note to Procurement	None.

Catalogue Number	Item Name	Available on Transversal Contract	Contract Number or Code
PHC-E-097	***Watercooler: free-standing, bottled***	No	

Functional Specifications	Free-standing floor-mounted unit.<br>The unit accepts standard 15 – 20 l bottles.<br>Dispenses cold water and room-temperature water via push-to-fill taps.<br>Bottle preferably refillable without removing.<br>Drip tray to prevent spillage onto floor.<br>Power cord at least 2 m with moulded 15A 3-pin plug.
Applicable Standards	SANS 60335 applicable section.
Performance Specifications	Minimum 30 cups of cold water per hour.<br>5-year design life.
Accessories	Disposable paper cups PHC-C-164.
Warranties	2 years.
Instructions to Suppliers	State available spare parts and after-sales support.<br>State guaranteed minimum spares availability (years).<br>State cost of 3-monthly sanitising.
Note to Procurement	Obtain quote for 3-monthly sanitising service, and also optional kit for sanitising by own personnel.

Miscellaneous

Catalogue Number	Item Name	Available on Transversal Contract	Contract Number or Code
PHC-E-159	***Brazier bin: 105x135x75***	No	
PHC-E-160	***Brazier bin: 105x190x75***	No	
PHC-E-161	***Brazier bin: 140x210x130***	No	
PHC-E-162	***Brazier bin: 140x280x130***	No	

Functional Specifications	Open-top bins made of durable plastic, in range of colours and sizes.<br>Can be arranged side-by-side on shelves. Can attach to wall-mounting plate via lip on rear.<br>Front edge has a flat portion to accept a label.
Applicable Standards	
Performance Specifications	5-year design life.
Accessories	
Warranties	2-year guarantee.
Instructions to Suppliers	
Note to Procurement	Obtain quote for wall-mounting plate, in different sizes.

Catalogue Number	Item Name	Available on Transversal Contract	Contract Number or Code
PHC-E-099	***Clock: wall***	No	

Functional Specifications	250-300mm. diameter, round. Battery-operated. Quartz-regulated direct-drive or stepper motor.<br>Body of high-impact plastic, with clear dial cover.<br>Dial plate white with bold black Arabic numerals.<br>Hour and minute hands black, sweep second hand may be black or red.<br>Built-in screw-head retainer for wall-mounting.
Applicable Standards	
Performance Specifications	Accuracy ±5 sec per 24 hours.<br>5-year design life.
Accessories	
Warranties	2 years.
Instructions to Suppliers	
Note to Procurement	

Catalogue Number	Item Name	Available on Transversal Contract	Contract Number or Code
PHC-E-100	***Complaints and suggestions box***	No	

Functional Specifications	Backboard 420mm wideX530mm high.<br>Attached to the backboard, aligned with the bottom and left edges, is a box with lid, 210mm wide X 230mm high X 113mm deep.<br>The lid of the box is hinged at the back, and has a hasp-and-staple closure on the front. The lid can be recessed into the sidewalls of the box. Slit in lid for posting: 100mm X 10mm.<br>To the right of the box, continuous with the front surface of the box, and aligned with the bottom and right edges of the backboard, is an unlidded box – storage space for forms. Box size is 210mm wide X 153mm high (i.e. shorter than closed box) X 113mm deep.<br>Entire construction in white frosted sheet acrylic (Perspex ®), 5mm thick.<br>Hinges and closure stainless steel.<br>Printing: Printed directly on the backboard (no adhesive labels).<br>Top row: (font Arial 72) "Complaint, compliment and suggestion box".<br>2nd and 3rd rows: Repeat text translated into the two most prevalent languages in the Province.<br>4th row: (font Arial 32) "Note: this box will be opened weekly".<br>Colour of printing to be determined by the Province, using Colour model CMYK.<br>Lock: solid brass combination-wheel luggage-type lock.<br>Must be mounted on wall. Bottom edge of backboard 1,2m above finished ground level.
Applicable Standards	None.
Performance Specifications	
Accessories	
Warranties	6 months.
Instructions to Suppliers	Supply and install on wall, in location specified by the Operational Manager.
Note to Procurement	

Catalogue Number	Item Name	Available on Transversal Contract	Contract Number or Code
PHC-E-101	**Gun safe**	No	

Functional Specifications	Wall-mounted safe for storage of hand-guns.<br>(a) **Material**: the construction of the safe shall be of mild steel of commercial (or higher) quality<br>(b) **Side thickness**: the thickness of the floor, roof and sides shall be at least 2mm and the wall thickness of the door shall be at least 3mm.<br>Specific requirements in respect of the design and construction of the doors of a safe referred to in this regulation, shall be the following:<br>(a) **Openings**: When the door is closed, there shall not be an opening of more than 1mm between the door and the door-frame<br>(b) **Door-frame**: If the hinges are concealed, the door-frame shall have a bended and welded construction with a reinforced rebate on three sides of the frame and in the case of hinges with a normal construction, the door-frame shall have a bended and welded construction with a reinforced rebate on all four sides of the frame. There shall be a protection guard (for instance a deflecting plate) to protect the lockpin<br>(c) **Bolts**: When the door is closed and locked, it shall not noticeably move if force is exerted by hand or with the handle, and the intervening space between any bolt and the rebate shall not exceed 1mm. Doors of safes which are higher than 500mm shall be equipped with at least three bolts<br>d) **Hinges**: Doors shall have an upper and a lower hinge which shall be so constructed that the door may open through at least an angle of 100 degrees and that the distance between the hinges does not exceed 350mm<br>(e) **Locks**: The safe door shall be equipped with one lever lock which shall have not less than ten levers of which not less than three shall be provided with false notches or a combination lock of the type which has three gears or an electronic lock which complies with the requirements approved of the South Africa Bureau of Standards.<br>The safe shall to the satisfaction of the Commissioner:<br>(a) be affixed flush to a floor, wall or other immovable structure or part thereof of the house, flat, residence or other dwelling place of an applicant concerned<br>Requirement is for two non-identical locks – both need to be opened with separate keys, to open the safe.
Applicable Standards	SANS953-1:2008.<br>Fire Arm Control Act 60 of 2000. Compliance must be certified.
Performance Specifications	Resistance to attack as per SANS953-1:2008.<br>15-year design life.
Accessories	2 sets of 2 keys.
Warranties	5-year guarantee.
Instructions to Suppliers	Supplier to install in collaboration with provincial technical personnel.
Note to Procurement	

Catalogue Number	Item Name	Available on Transversal Contract	Contract Number or Code
PHC-E-102	***Notice board: A3 size***	No	
PHC-E-103	***Notice board: A2 size***	No	

Functional Specifications	Anodised aluminium frame.<br>Mounted direct to wall (not hung on cord).<br>Fibreboard backing, faced with matt-finish polyester felt fabric.<br>Dark blue, dark green or black.<br>Fibreboard accepts chart pins securely.
Applicable Standards	
Performance Specifications	5-year design life.
Accessories	Wall-mounting hardware. Box of multi-coloured plastic push pins.
Warranties	2 years.
Instructions to Suppliers	Installation by departmental technical personnel.
Note to Procurement	

Catalogue Number	Item Name	Available on Transversal Contract	Contract Number or Code
PHC-E-104	***Pamphlet-holding boxes***	No	

Functional Specifications	White backing board.<br>Individual clear acrylic pockets, to hold A4 size pamphlets or smaller.<br>2 horizontal rows of 4 pockets.
Applicable Standards	
Performance Specifications	5-year design life.
Accessories	Wall-mounting hardware.
Warranties	2 years.
Instructions to Suppliers	Installation by departmental technical personnel.
Note to Procurement	

Catalogue Number	Item Name	Available on Transversal Contract	Contract Number or Code
PHC-E-105	***Poster display boards: A1 size***	No	
PHC-E-106	***Poster display boards: A0 size***	No	

Functional Specifications	Anodised aluminium frame.<br>Mounted direct to wall (not hung on cord).<br>White hardboard backing. Poster adhere with double-sided tape.<br>Transparent vinyl sheet to protect surface of poster.
Applicable Standards	
Performance Specifications	5-year design life.
Accessories	Wall-mounting hardware.
Warranties	2 years.
Instructions to Suppliers	Installation by departmental technical personnel.
Note to Procurement	

Catalogue Number	Item Name	Available on Transversal Contract	Contract Number or Code
PHC-E-107	***Whiteboard: 1200x1000***	No	
PHC-E-108	***Whiteboard: 1800x1200***	No	
PHC-E-109	***Whiteboard: 3000x1200***	No	

Functional Specifications	Anodised aluminium frame.<br>Mounted direct to wall (not hung on cord).<br>Magnetic white epoxy-coated sheet metal writing surface, with sound-deadening backing.
Applicable Standards	
Performance Specifications	5-year design life.
Accessories	Wall-mounting hardware. Whiteboard felt eraser.
Warranties	2 years.
Instructions to Suppliers	Installation by departmental technical personnel.
Note to Procurement	

Furniture and appliances

Clinical

Catalogue Number	Item Name	Available on Transversal Contract	Contract Number or Code
PHC-F-025	***Bassinet***	RT 24	RT 24-02-018
PHC-F-026	***Bassinet, plastic, spare***	RT 24	RT 24-02-019
PHC-F-027	***Mattress for bassinet, spare***	RT 24	RT 24-02-021

Functional Specifications	Bassinet, hospital, type A, with stand complete with mattress.<br>To comply with the latest issue of CKS 237, type A<br>**Bassinet**: durable, transparent plastic material<br>Mild steel frame with epoxy/nylon powder-coated finish to comply with SANS 778 paragraph 5.2, proof of compliance must be submitted<br>**Mattress**: Must be in accordance with item RT 24-02-021<br>On castors.
Applicable Standards	To comply with the latest issue of CKS 237, type A.<br>SANS 778 paragraph 5.2, proof of compliance must be submitted.<br>Castors shall be in accordance with SANS 621 (see Special Conditions).
Performance Specifications	Expected service life of frame: 15 years.
Accessories	None.
Warranties	2 years on all components.
Instructions to Suppliers	None.
Note to Procurement	Item RT 24-02-021 spare mattress and RT 24-02-021 spare mattress cover in series with this item.

Catalogue Number	Item Name	Available on Transversal Contract	Contract Number or Code
PHC-F-072	***Bed: hospital, 2-section***	RT24-2016	RT24-02-001

Functional Specifications	Mild steel frame with epoxy/nylon powder coating colours: white, cream or grey.<br>To be fitted with castors, two swivel, two locking.<br>Removable head and foot ends (ABS material is acceptable).<br>Mattress support: mattress support other than weldmesh is required, provide details.
Applicable Standards	To comply with the latest issue of SANS 521, in subsections 3.1 (a) and 5.1 (Type) of the latest issue of SANS 521 Note: where the item offered differs from the specification in SANS 521, the supplier must indicate the deviation and supply relevant details.<br>Must comply with IEC 60601-2-52 (Particular requirements for basic safety and essential performance of medical beds) paragraphs: 201.1, 201.3, 201.7, 201.9, 201.13, 201.15, annex BB and annex CC.<br>Epoxy/nylon powder-coated finish to comply with SANS 778 paragraph 5.2, proof of compliance must be submitted.<br>Castors must comply with the latest issue of SANS 621, proof of compliance must be submitted. Where castors are fitted into steel tubular legs, the tube shall be of wall thickness not less than 2,0mm and castors shall be fixed to the tube by one of the following methods: a. Solid plug (long) complying with SANS 621 subsection 3.5.6 or b. Screwed into 35mm long sleeves welded into the tubular members and locked in an acceptable manner c. Rubber or plastic expanding sleeves for fitting castors are not acceptable.<br>Collapsible safety sides, to comply with IEC 60601-2-52.
Performance Specifications	Bed must support a minimum patient mass of 180 kg. 200kg preferred. Adjustable backrest with gas spring assist, suitable for 100 kg patient.<br>Bed must not disassemble if sides or ends are lifted – collapsible "emergency" beds are not fit for this purpose.
Accessories	Complete with folding cot sides. Drip pole and patient lifting pole and strap optional.
Warranties	2 years.
Instructions to Suppliers	Provide separate pricing for accessory drip pole, patient lifting pole and strap.
Note to Procurement	Item RT 24-02-021 spare mattress and RT 24-02-021 spare mattress cover in series with this item.

Catalogue Number	Item Name	Available on Transversal Contract	Contract Number or Code
PHC-F-073	***Bed: hospital, mattress***	RT24-2016	RT24-02-014

Functional Specifications	To be constructed of flexible.<br>Overall dimensions: Length: 1 980 mm (-0 and +40mm) Width: 860 mm (-0 and +20mm) Thickness: 150 mm (-0 and +5mm).
Applicable Standards	Polyurethane foam complying with class 30, grade no.12 of SANS 640.<br>Overall to comply with the latest issue of SANS 640 AND 1291-1 (type 2), except for thickness indicated below. Proof of compliance must be submitted.
Performance Specifications	Mattress must support a minimum patient mass of 180 kg. 200kg preferred. Must resume original shape when not in use.<br>Mattress must be radio-translucent.
Accessories	
Warranties	2 years.
Instructions to Suppliers	Provide separate pricing for accessory drip pole, patient lifting pole and strap.
Note to Procurement	Must fit standard beds, must be in series with mattress cover PHC-F-074.

Catalogue Number	Item Name	Available on Transversal Contract	Contract Number or Code
PHC-F-074	***Bed: hospital, mattress cover***	RT24-2016	RT24-02-015

Functional Specifications	Cover must be removable, water-resistant, air permeable material and radio-translucent.<br>Where a zip is incorporated, it shall be a heavy duty, large- toothed synthetic zip to allow removal of the inner mattress, totally covered by a flap extending over the zip to prevent ingress of fluids.
Applicable Standards	To comply with the latest issue of SANS 1291-1, proof of compliance must be submitted.
Performance Specifications	
Accessories	
Warranties	2 years.
Instructions to Suppliers	
Note to Procurement	

Catalogue Number	Item Name	Available on Transversal Contract	Contract Number or Code
PHC-F-029	***Bed: hospital, obstetric***	RT 24	RT 24-02-009

Functional Specifications	Bed, hospital, obstetric, high-low, tilting, 2 section, complete with mattress.<br>Bed and fittings must accommodate various labour and delivery positions<br>Mild steel frame with epoxy/nylon powder-coated finish to comply with SANS 778 paragraph 5.2, proof of compliance must be submitted<br>Epoxy/nylon powder coating colours: white, cream or grey<br>Mattress platform material<br>Removable leg section<br>Adjustable backrest with gas spring assist (0 to 60 degrees) with quick release<br>Controls at both sides of bed<br>Hydraulically operated variable height operated by dual sided foot pedals<br>Height range (mattress platform): 500 to 750mm (approximately)<br>Trendelenburg tilt, 12 degrees<br>To be fitted with 125mm castors with a central castor locking system<br>Castors must comply with the latest issue of SANS 621, proof of compliance must be submitted<br>Where castors are fitted into steel tubular legs, the tube shall be of wall thickness not less than 2,0mm,with collapsible safety sides, to comply with IEC 60601-2-52. Rubber buffer wheels at the corners of bed head end<br>Mattress, two section, for body and foot sections, with cover. Body section must be suitable for all profile angles.
Applicable Standards	Must comply with IEC 60601-2-52 (Particular requirements for basic safety and essential performance of medical beds) paragraphs: 201.1, 201.3, 201.7, 201.9, 201.13, 201.15, annex BB and annex CC.<br>Epoxy/nylon powder-coated finish to comply with SANS 778 paragraph 5.2, proof of compliance must be submitted.<br>Castors must comply with the latest issue of SANS 621, proof of compliance must be submitted.<br>Collapsible safety sides, to comply with IEC 60601-2-52.<br>Mattress to comply with the latest issue of SANS 640 AND 1291-1 (type 2), except for thickness, as below, proof of compliance must be submitted.<br>Mattress To be constructed of flexible polyurethane foam complying with class 30, grade no.12 of SANS 640.

Performance Specifications	Expected service life minimum 10 years.<br>Bed must support a patient mass of 180kg.<br>Mattress must support a patient of at least 180kg and return to original shape when not in use.<br>Thickness: 150mm (-0 and +5mm).<br>Must be cleanable and disinfectable with standard solutions.
Accessories	Two lithotomy poles, height adjustable, swivel action, with leg support (not straps).<br>Douche fitting.<br>Douche tray, stainless steel.<br>Drip rod.
Warranties	Manufacturer must supply a 5-year warranty on the mattress.
Instructions to Suppliers	None.
Note to Procurement	

Catalogue Number	Item Name	Available on Transversal Contract	Contract Number or Code
PHC-F-033	***Bucket for kick-about***	RT 24	RT 24-10-004

Functional Specifications	Bucket for kick-about.<br>Stainless steel, with medium directional satin finish.
Applicable Standards	To comply with the latest issue of CKS 294, Type B.<br>Stainless steel must comply with the latest issue of SANS 417, subsection 4.1.2, proof of compliance must be submitted.
Performance Specifications	Service life minimum 10 years.<br>Must be cleanable and disinfectable with standard solutions.
Accessories	None.
Warranties	2 years on all components.
Instructions to Suppliers	None.
Note to Procurement	Item to be evaluated as series with item RT 24-10-003 in terms of paragraph 16.4 in the Special Conditions of Contract.

Catalogue Number	Item Name	Available on Transversal Contract	Contract Number or Code
PHC-F-069	***Cabinet: medicine, mobile***	RT24-2016	RT24-04-003

Functional Specifications	Multi-drawer mobile medicine trolley, for use in a ward environment.<br>Flat top surface, for writing and dispensing.<br>Must have 4-6 drawers with adjustable dividers, 2 large storage drawers without dividers.<br>Drawers must have smooth, durable sliding mechanism with stop to prevent total extraction of drawer.<br>Mounted on 4 heavy duty swivel castors 125 mm diameter, the two front castors should be fitted with foot brakes with toe release mechanism.<br>Approximate dimensions: Height: 950 mm Width: 750 mm Depth: 500 mm.<br>Materials which are easily cleaned and of lasting finish, and low noise generating nature, shall be used.
Applicable Standards	Castors must comply with SANS 621 and proof of compliance must be submitted. Castors must be antistatic. Where castors are fitted into steel tubular legs, the tube shall be of wall thickness not less than 2,0mm and castors shall be fixed to the tube by one of the following methods: a. Solid plug (long) complying with SANS 621 subsection 3.5.6 or b. Screwed into 35mm long sleeves welded into the tubular members and locked in an acceptable manner c. Rubber or plastic expanding sleeves for fitting castors are not acceptable.
Performance Specifications	
Accessories	Drawer dividers.
Warranties	2 years.
Instructions to Suppliers	
Note to Procurement	

Catalogue Number	Item Name	Available on Transversal Contract	Contract Number or Code
PHC-F-070	***Cabinet: scheduled drugs, wall-mounted***	RT24-2016	RT24-04-005

Functional Specifications	Wall-mounted, lockable cabinet.<br>Mild steel construction, with epoxy/nylon powder-coated finish.<br>At least one internal shelf.<br>The mild steel plate shall have a nominal thickness of not less than 1,20 mm.<br>Lock must be robust, and engage fully with the cabinet body. Not of the cam-and-lever variety. Preferably locking by retractable bolts.<br>NOTE: The cabinet must not be marked "Poison".
Applicable Standards	Mild steel frame with epoxy powder-coated or nylon powder-coated finish to comply with SANS 778 paragraph 5.2, proof of compliance must be submitted.
Performance Specifications	Adjustable height from 740 mm to 1 090 mm. Automatic lock.<br>Minimum weight bearing capacity measured at free end of table: 20kg without flexing or distortion of frame.
Accessories	All wall-mounting hardware. 2 keys.
Warranties	2 years.
Instructions to Suppliers	
Note to Procurement	Please note the requirement for the lock – this may differ from the item currently on RT24.

Catalogue Number	Item Name	Available on Transversal Contract	Contract Number or Code
PHC-F-060	***Treatment couch: hydraulic Bobath (neuro)***	No	

Functional Specifications	Large padded treatment couch/plinth, height adjustable (hydraulic) and modular:<br>120-125cm wide, 195cm long, height range 46cm-91cm, backrest angle 80-85 degrees. Safe working load of 250kg. Hydraulic height operation.
Applicable Standards	
Performance Specifications	
Accessories	None.
Warranties	3 years.
Instructions to Suppliers	
Note to Procurement	

Catalogue Number	Item Name	Available on Transversal Contract	Contract Number or Code
PHC-F-028	***Drip stand: mobile***	RT 24	RT 24-08-009

Functional Specifications	Drip stand, mobile.<br>Mild steel frame with epoxy powder-coated or nylon powder-coated finish to comply with SANS 778 paragraph 5.2, proof of compliance (Test Report) must be submitted.<br>Castors must comply with the latest issue of SANS 621, proof of compliance (Test Report) must be submitted.<br>Where castors are fitted into steel tubular legs, the tube shall be of wall thickness not less than 2,0mm and castors shall be fixed to the tube by one of the following methods:<br>a. Solid plug (long) complying with SANS 621 subsection 3.5.6 or<br>b. Screwed into 35mm long sleeves welded into the tubular members and locked in an acceptable manner<br>c. Rubber or plastic expanding sleeves for fitting castors are not acceptable.<br><br>Drag chain to be supplied.
Applicable Standards	To comply with the latest issue of CKS 179, Type A.<br>SANS 778 paragraph 5.2.<br>SANS 621.
Performance Specifications	Must be cleanable and disinfectable with standard solutions.<br>Expected service life: 15 years.
Accessories	None.
Warranties	2 years on all components.
Instructions to Suppliers	None.
Note to Procurement	None.

Catalogue Number	Item Name	Available on Transversal Contract	Contract Number or Code
PHC-F-030	***Examination couch***	RT 24	RT 24-02-024

Functional Specifications	Examination couch, consisting of mild steel frame (tubular or square section).<br>Epoxy/nylon powder-coated finish to comply with SANS 778 paragraph 5.2, proof of compliance must be submitted<br>Two-piece design, head piece hinged, elevatable to 60 degrees above horizontal. Notched steel supports preferred over friction stay<br>Platform of sealed medium-density fibreboard or 16mm plywood. Sealed.<br>Padding minimum 70mm<br>Covering material must be durable and waterproof. Dark colour preferred.<br>Must be re-upholsterable<br>Heavy-duty rubber ferrules on feet.
Applicable Standards	CSK 243 latest version.
Performance Specifications	Service life at least 10 years.<br>Must be cleanable and disinfectable with standard solutions.
Accessories	
Warranties	2 years.
Instructions to Suppliers	None.
Note to Procurement	None.

Catalogue Number	Item Name	Available on Transversal Contract	Contract Number or Code
PHC-F-061	***Examination couch: portable***	No	

Functional Specifications	Folding examination couch, modular, height adjustable, with removable head and arm rests in a canvas bag.<br>Minimum weight-bearing capacity 180kg.
Applicable Standards	
Performance Specifications	
Accessories	In some models, padded headrest/breathing hole and arm supports may come separately. Should be included.
Warranties	3 years.
Instructions to Suppliers	
Note to Procurement	In some models, padded headrest/breathing hole and arm supports may come separately. Should be included.

Catalogue Number	Item Name	Available on Transversal Contract	Contract Number or Code
PHC-F-062	***Examination couch: rehab***	No	

Functional Specifications	Examination couch with breathing hole.<br>Mild steel frame with epoxy/nylon powder-coated finish to comply with SANS 778 paragraph 5.2, proof of compliance must be submitted.<br>Two-piece design, backrest hinged, elevatable to 60 degrees above horizontal. Notched steel supports preferred over friction stay.<br>Platform of sealed medium-density fibreboard or 16mm plywood. Sealed. Padding minimum 70mm.<br>Covering material must be durable and waterproof. Dark colour preferred. Must be re-upholsterable.<br>Heavy-duty rubber ferrules on feet.
Applicable Standards	SANS 778 paragraph 5.2.
Performance Specifications	Service life at least 10 years.<br>Must be cleanable and disinfectable with standard solutions.
Accessories	
Warranties	2 years.
Instructions to Suppliers	
Note to Procurement	Need one of this type per facility; remainder can be standard examination couches.

Catalogue Number	Item Name	Available on Transversal Contract	Contract Number or Code
PHC-F-031	***Foot stool***	RT 24	RT 24-11-003

Functional Specifications	Stool, foot, double step.<br>To comply with the specification CKS 233 in Annexure J, see attached.<br>Aluminium chequered plate top.<br>Stainless steel edging.<br>Mild steel frame and legs with epoxy/nylon powder-coated finish, to comply with SANS 778 paragraph 5.2, proof of compliance must be submitted.<br>Heavy-duty rubber non-slip, non-scratching ferrules on feet.
Applicable Standards	CKS233 in the specification in Appendix J.<br>SANS 778 paragraph 2, proof of compliance must be submitted.
Performance Specifications	Service life minimum 10 years.<br>Must be cleanable and disinfectable with standard solutions.
Accessories	None.
Warranties	2 years.
Instructions to Suppliers	None.
Note to Procurement	Please ensure that there is additional bracing under the steps, as arranged with the supplier in 2015. Load measurement method in CKS233 is inadequate. Load is too light, spread over a surface area much larger than a person's foot. Hence there have been premature failures – buckling of the steps. Future procurement to increase capacity to at least 180kg, with a load area no wider than 10cmx30cm. Aluminium chequered plate is more durable than non-slip rubber covering on the steps.

Catalogue Number	Item Name	Available on Transversal Contract	Contract Number or Code
PHC-F-032	***Kick-about***	RT 24	RT 24-10-003

Functional Specifications	Kick-about, for bucket.<br>Stainless steel, with medium directional satin finish.<br>Mounted on four legs fitted with castors.<br>Castors must comply with the latest issue of SANS 621, proof of compliance must be submitted.<br>Where castors are fitted into steel tubular legs, the tube shall be of wall thickness not less than 2,0mm and castors shall be fixed to the tube by one of the following methods:<br>a. Solid plug (long) complying with SANS 621 subsection 3.5.6 or<br>b. Screwed into 35mm long sleeves welded into the tubular members and locked in an acceptable manner<br>c. Rubber or plastic expanding sleeves for fitting castors are not acceptable.<br>The horizontal braces to be arranged in a flat cross formation.
Applicable Standards	To comply with the latest issue of CKS 294, Type B.<br>Stainless steel must comply with the latest issue of SANS 417, subsection 4.1.2, proof of compliance must be submitted.<br>SANS 62.
Performance Specifications	Service life minimum 10 years.<br>Must be cleanable and disinfectable with standard solutions.
Accessories	None.
Warranties	2 years on all components.
Instructions to Suppliers	None.
Note to Procurement	Item to be evaluated as series with item RT 24-10-004 in terms of paragraph 16.4 in the Special Conditions of Contract.

Catalogue Number	Item Name	Available on Transversal Contract	Contract Number or Code
PHC-F-071	***Locker: bedside, steel***	RT24-2016	RT24-07-001

Functional Specifications	Steel bedside locker.<br>Mild steel construction, with epoxy/nylon powder-coated finish.<br>Materials which are easily cleaned and of lasting finish, and low noise generating nature, must be used.<br>Stainless steel top, with rail on three sides.<br>Single pull-out drawer, with durable sliding mechanism and stop to prevent total extraction of drawer.<br>Locker mounted on 4 castors.
Applicable Standards	To comply with the latest issue of SANS 343, Type 1 Material: Mild steel with epoxy/nylon powder-coated finish to comply with SANS 778 paragraph 5.2, proof of compliance must be submitted. Stainless steel must comply with the latest issue of SANS 417, subsection 4.1.2.<br>Castors must comply with the latest issue of SANS 621, proof of compliance (Test Report) must be submitted. Where castors are fitted into steel tubular legs, the tube shall be of wall thickness not less than 2,0mm and castors shall be fixed to the tube by one of the following methods: a. Solid plug (long) complying with SANS 621 subsection 3.5.6 or b. Screwed into 35mm long sleeves welded into the tubular members and locked in an acceptable manner c. Rubber or plastic expanding sleeves for fitting castors are not acceptable.
Performance Specifications	
Accessories	
Warranties	2 years.
Instructions to Suppliers	
Note to Procurement	

Catalogue Number	Item Name	Available on Transversal Contract	Contract Number or Code
PHC-F-049	***Refrigerator: vaccines, chest model***	No	

Functional Specifications	Chest model.<br>As per WHO specification E003/RF01.2 " Refrigerator or combined refrigerator water-pack freezer: compression cycle", with the following modifications (not in WHO spec).<br>Minimum internal volume = 100l.<br>No water-pack freezer compartment (we will cater for this need by specifying a medicine fridge with freezer compartment).<br>Will be used with an externally readable data logging thermometer, procured as separate item.<br>Power supply: 220V /50Hz 1 phase.
Applicable Standards	Refrigerant: CFC free.<br>WHO specification E003/RF01.2 Pre-qualification on WHO PQS – with proof – will be an advantage.
Performance Specifications	Warning device for min. 12h in the event of power failure (power failure alarm).<br>Hold-over period 48 hrs or better.<br>Must be cleanable and disinfectable with standard materials.
Accessories	None.
Warranties	3 years.
Instructions to Suppliers	Optionally the supplier can propose more than one model, for use in moderate and hot temperature zones.<br>Includes delivery, installation, training.<br>Supplier must specify available after-sales support, with relevant pricing.<br>Supplier should propose a shelving solution as an optional costed item, to overcome the inherent disadvantage of this type of model (i.e. accessing items located below other items).
Note to Procurement	A separate data logging thermometer is required for use with this item. The thermometer has a 2-year service life.

Catalogue Number	Item Name	Available on Transversal Contract	Contract Number or Code
PHC-F-050	***Refrigerator: vaccines, upright model***	No	

Functional Specifications	Upright model.<br>As per WHO specification E003/RF01.2 " Refrigerator or combined refrigerator water-pack freezer, compression cycle", with the following modifications (not in WHO spec):<br>Minimum internal volume = 100l<br>No water-pack freezer compartment (we will cater for this need by specifying a medicine fridge with freezer compartment)<br>Will be used with an externally readable data logging thermometer, procured as separate item<br>Power supply: 220V /50Hz 1 phase.
Applicable Standards	Refrigerant: CFC free.<br>WHO specification E003/RF01.2 Pre-qualification on WHO PQS -with proof – will be an advantage.
Performance Specifications	Warning device for min. 12h in the event of power failure (power failure alarm).<br>Hold-over period 48 hours or better.<br>Must be cleanable and disinfectable with standard materials.
Accessories	None.
Warranties	3 years.
Instructions to Suppliers	Optionally the supplier can propose more than one model, for use in moderate and hot temperature zones.<br>Includes delivery, installation, training.<br>Supplier must specify availability of after-sales support, with relevant pricing.
Note to Procurement	A separate data logging thermometer is required for use with this item. The thermometer has a 2-year service life.

Catalogue Number	Item Name	Available on Transversal Contract	Contract Number or Code
PHC-F-063	***Screen: bedside, wheeled. Complete with curtain.***	RT24-2016	RT24-11-009

Functional Specifications	Four-panel concertina- foldable screen, consisting of steel frames on castors, and plastic curtains.<br>Must comply with the latest issue of SANS 897, fig.3, Type C.<br>Overall height 1 680 mm.<br>Mild steel frame and legs with epoxy/nylon powder-coated finish, to comply with SANS 778 paragraph 5.2, proof of compliance must be submitted. Castors: 75 mm Castors must comply with the latest issue of SANS 621, proof of compliance must be submitted.<br>Where castors are fitted into steel tubular legs, the tube shall be of wall thickness not less than 2,0mm and castors shall be fixed to the tube by one of the following methods: a. Solid plug (long) complying with SANS 621 subsection 3.5.6 or b. Screwed into 35mm long sleeves welded into the tubular members and locked in an acceptable manner c. Rubber or plastic expanding sleeves for fitting castors are not acceptable.<br>Curtain to be as follows: Strong non-transparent plastic Sides to be strengthened and to be full fit in frame sections. Curtain must be easily removable.
Applicable Standards	SANS 897, fig.3, Type C.<br>SANS 778 paragraph 5.2.<br>SANS 621.
Performance Specifications	Curtains must be washable. Expected service life:<br>Frames 10 years<br>Curtains 3 years.
Accessories	Replacement curtains RT24-11-010.
Warranties	5 years on frames, 1 year on curtains.
Instructions to Suppliers	
Note to Procurement	

Catalogue Number	Item Name	Available on Transversal Contract	Contract Number or Code
PHC-F-064	***Screen: bedside, replacement curtains***	RT24-2016	RT24-11-010

Functional Specifications	Replacement curtain for 4-panel folding screen on castors.<br>Strong non-transparent plastic sides to be strengthened.<br>Strong non-transparent sides to be strengthened and curtains to be full fit in frame sections.
Applicable Standards	
Performance Specifications	Must be washable.<br>Expected service life 3 years.
Accessories	None.
Warranties	1 year.
Instructions to Suppliers	
Note to Procurement	Must be compatible with item RT24-11-009.

Catalogue Number	Item Name	Available on Transversal Contract	Contract Number or Code
PHC-F-034	***Stool: anaesthetist***	RT 24	RT 24-11-002

Functional Specifications	Stool, anaesthetist, with castors.<br>To comply with CKS233 Type B.<br>Aluminium flat seat.<br>Spindle to be threaded with square or acme thread.<br>**Castors**: 75mm.<br>Castors must comply with the latest issue of SANS 621, proof of compliance must be submitted.<br>Where castors are fitted into steel tubular legs, the tube shall be of wall thickness not less than 2,0mm and castors shall be fixed to the tube by one of the following methods:<br>a. Solid plug (long) complying with SANS 621 subsection 3.5.6 or<br>b. Screwed into 35mm long sleeves welded into the tubular members and locked in an acceptable manner<br>c. Rubber or plastic expanding sleeves for fitting castors are not acceptable.
Applicable Standards	CKS233 Type B.<br>SANS 621, proof of compliance must be submitted.
Performance Specifications	Height adjustments, floor to top of seat: 485mm to 685mm.<br>Service life minimum 10 years.<br>Must be cleanable and disinfectable with standard solutions.
Accessories	None.
Warranties	2 years.
Instructions to Suppliers	None.
Note to Procurement	

Catalogue Number	Item Name	Available on Transversal Contract	Contract Number or Code
PHC-F-065	***Stool: dental assistant***	In preparation	

Functional Specifications	An ergonomic stool used by the Dental assistant.<br>2-way adjustable backrest for lumbar support. Anatomically shaped.<br>Contoured seat cushions with a "rolled" edge to reduce pressure on the legs.<br>Easy to reach adjustment paddle for increased lumbar support.<br>Tear-resistant upholster.<br>Seamless upholstery for easier cleaning.<br>Upholstery, design and colour to be in line with the patient operator chair.<br>5 leg cast aluminium base.<br>Twin wheel castors.<br>Adjustable torso support that can be converted for left or right handed operators.<br>Height adjustable foot ring.<br>The unit must be height adjustable (gas cylinder or spring).
Applicable Standards	ISO 7493: 2006.
Performance Specifications	Expected service life 10 years.
Accessories	None.
Warranties	5 years.<br>Spares availability guarantee 5 years.
Instructions to Suppliers	None.
Note to Procurement	

Catalogue Number	Item Name	Available on Transversal Contract	Contract Number or Code
PHC-F-066	***Stool: dentist***	In preparation	

Functional Specifications	An ergonomic stool used by the operator.<br>2-way adjustable backrest for lumbar support. Anatomically shaped.<br>Contoured seat cushions with a "rolled" edge to reduce pressure on the legs.<br>Easy to reach adjustment paddle for increasing lumbar support.<br>Tear- resistant upholstery.<br>Seamless upholstery for easier cleaning.<br>Upholstery, design and colour to be in line with the patient chair.<br>5 leg cast aluminium base.<br>Twin wheel castors.<br>The unit must be height adjustable.<br>The unit must have active and passive backrest.
Applicable Standards	ISO 7493: 2006.
Performance Specifications	Expected service life 10 years.
Accessories	None.
Warranties	5 years.<br>Spares availability guarantee 5 years.
Instructions to Suppliers	None.
Note to Procurement	

Catalogue Number	Item Name	Available on Transversal Contract	Contract Number or Code
PHC-F-035	***Stool: height adjustable, with backrest***	No	

Functional Specifications	Stool, dental-type, with castors.<br>To comply with CKS233 Type B.<br>Upholstered seat.<br>Spindle to be threaded with square or acme thread.<br>Can swivel, but not tilt. Footrest ring above level of castors.<br>**Castors**: 75mm.<br>Castors must comply with the latest issue of SANS 621, proof of compliance must be submitted.<br>Where castors are fitted into steel tubular legs, the tube shall be of wall thickness not less than 2,0mm and castors shall be fixed to the tube by one of the following methods:<br>a. Solid plug (long) complying with SANS 621 subsection 3.5.6 or<br>b. Screwed into 35mm long sleeves welded into the tubular members and locked in an acceptable manner<br>c. Rubber or plastic expanding sleeves for fitting castors are not acceptable.<br>Backrest: padded, height adjustable relative to seat.
Applicable Standards	CKS233 Type B.<br>SANS 621, proof of compliance must be submitted.
Performance Specifications	Height adjustments, floor to top of seat: 485mm to 685mm.<br>Service life minimum 10 years.<br>Must be cleanable and disinfectable with standard solutions.
Accessories	None.
Warranties	2 years.
Instructions to Suppliers	None.
Note to Procurement	

Catalogue Number	Item Name	Available on Transversal Contract	Contract Number or Code
PHC-F-075	***Table: over- bed***	RT24-2016	RT24-07-003

Functional Specifications	Cantilever-type over-bed table – can be placed in position from the side of the bed.
Applicable Standards	To comply with the latest issue of SANS 777, Type B Mild steel frame with epoxy powder-coated or nylon powder-coated finish to comply with SANS 778 paragraph 5.2, proof of compliance must be submitted.<br>Castors must comply with the latest issue of SANS 621, proof of compliance must be submitted. Where castors are fitted into steel tubular legs, the tube shall be of wall thickness not less than 2,0mm and castors shall be fixed to the tube by one of the following methods: a. Solid plug (long) complying with SANS 621 subsection 3.5.6 or b. Screwed into 35mm long sleeves welded into the tubular members and locked in an acceptable manner c. Rubber or plastic expanding sleeves for fitting castors are not acceptable.
Performance Specifications	Adjustable height from 740 mm to 1 090 mm. Automatic lock.<br>Minimum weight bearing capacity measured at free end of table: 20kg without flexing or distortion of frame.
Accessories	
Warranties	2 years.
Instructions to Suppliers	
Note to Procurement	

Catalogue Number	Item Name	Available on Transversal Contract	Contract Number or Code
PHC-F-036	***Trolley: anaesthetic***	RT 24	RT 24-12-005

Functional Specifications	Trolley, anaesthetic. Type 3 , size C.<br>To comply with the latest issue of SANS 726 (see fig 2 and table 2).<br>Size: Length: 455mm (± 5mm).<br>**Width**: 455mm (± 5mm).<br>The upper part is enclosed to contain a drawer (with no divisions) opening to the front.<br>Drawer must have a stopper to prevent complete removal of drawer.<br>Durable rubber plugs (or similar noise reducing material) must be inserted into rails below removable shelves.<br>The top shelf has a rail along sides and back.<br>Bottom shelf must have a continuous rail on all four sides.<br>Materials which are easily cleaned and of lasting finish, and low noise generating nature, shall be used.<br>Stainless steel throughout. Stainless steel must comply with subsection 4.1.2 of the latest issue of SANS 417 (See special conditions).<br>Four swivel castors with a diameter of 75mm each.<br>Castors shall be in accordance with SANS 621 (see Special Conditions).<br>Where castors are fitted into steel tubular legs, the tube shall be of wall thickness not less than 2,0mm and castors shall be fixed to the tube by one of the following methods:<br>a. Solid plug (long) complying with SANS 621 subsection 3.5.6 or<br>b. Screwed into 35mm long sleeves welded into the tubular members and locked in an acceptable manner.
Applicable Standards	To comply with the latest issue of CKS 294, Type B.<br>Stainless steel must comply with the latest issue of SANS 417, subsection 4.1.2, proof of compliance must be submitted.<br>SANS 621.
Performance Specifications	Service life minimum 10 years.<br>Must be cleanable and disinfectable with standard solutions.
Accessories	None.
Warranties	2 years on all components.
Instructions to Suppliers	None.
Note to Procurement	None.

Catalogue Number	Item Name	Available on Transversal Contract	Contract Number or Code
PHC-F-040	***Trolley: emergency***	RT 24-2016	RT 24-12-001

Functional Specifications	Dedicated "crash cart" type trolley.<br>All metal or ABS construction<br>Side-accessible slide-out tray for medication preparation<br>Top drawer lockable half-height, with Moulded insert for emergency drugs. Facility for Tamper–proof seals<br>Four half-height drawers, for resuscitation equipment, bandages, syringes, needles, ET tubes<br>One deep drawer for manual resuscitation ventilators and masks<br>Side bracket for portable suction machine<br>Side accessory tilt-out drawers<br>On-board attached multi-plug with at least 5 15A 3-pin sockets (switchable) and 5 Euro-style sockets. Single power lead, 3-core 2,5mm^2, terminated with moulded 15A 3-pin plug (SANS approved)<br>Raised bridge rail at rear of top, to attach NIBP monitor and AED<br>Swing-out trays not suitable<br>Built-in extendable drip stand, left- or right-mounting<br>Mounted on 4 approved castors, with non-loosening fixings<br>Hooks for mounting cardiac board at rear<br>Oxygen cylinder holder not required<br>Cleanable and disinfectable with standard solutions.
Applicable Standards	SANS 621.
Performance Specifications	Service life minimum 10 years.<br>Must be cleanable and disinfectable with standard solutions.<br>Dimensions must allow passage through standard doorway.
Accessories	Cardiac board, drugs insert, laryngoscope and ET tube insert.<br>Half-height plastic bins for syringes and needles. Drip T-rod.
Warranties	2 years on all components.
Instructions to Suppliers	Delivered and assembled. Propose optional drawer dividers (priced per set).
Note to Procurement	These specifications exceed those in RT 24, and should be used for future procurement. The existing configuration was found to be inadequate for PHC purposes.

Catalogue Number	Item Name	Available on Transversal Contract	Contract Number or Code
PHC-F-041	***Trolley: gas cylinder***	RT 24	RT 24-12-003

Functional Specifications	Gas cylinder trolley, for 300mm diameter cylinder.<br>To comply with the latest issue of SANS 895.<br>Material: Mild steel frame with epoxy/nylon powder-coated finish to comply with SANS 778 paragraph 5.2, proof of compliance must be submitted.
Applicable Standards	SANS 895.<br>SANS 778 paragraph 5.2.
Performance Specifications	Diameter of cylinder: 300mm.<br>Length of cylinder: 1 300mm.
Accessories	
Warranties	2 years.
Instructions to Suppliers	None.
Note to Procurement	Must have at least two wheels.

Catalogue Number	Item Name	Available on Transversal Contract	Contract Number or Code
PHC-F-039	***Trolley: medical dressing, large***	RT 24	RT 24-12-008

Functional Specifications	Trolley, medical dressing.<br>To comply with the latest issue of SANS 726 (see fig 4).<br>Length: 915mm (± 5mm).<br>Width: 455mm (± 5mm).<br>The trolley is open and has two removable stainless steel shelves.<br>Durable rubber plugs (or similar noise reducing material) must be inserted into rails below removable shelves.<br>It has a continuous guard rail along the sides and back of the top shelf and a continuous rail fitted around the bottom, above the bottom shelf.<br>Materials which are easily cleaned and of lasting finish, and low noise generating nature, shall be used.<br>Stainless steel throughout.<br>Four swivel castors with a diameter of 75mm each. Where castors are fitted into steel tubular legs, the tube shall be of wall thickness not less than 2,0mm and castors shall be fixed to the tube by one of the following methods:<br>a. Solid plug (long) complying with SANS 621 subsection 3.5.6 or<br>b. Screwed into 35mm long sleeves welded into the tubular members and locked in an acceptable manner.
Applicable Standards	To comply with the latest issue of SANS 726, Type 4, size A.<br>Stainless steel must comply with the latest issue of SANS 417, subsection 4.1.2, proof of compliance must be submitted.<br>Castors shall be in accordance with SANS 621 (see Special Conditions).
Performance Specifications	Service life minimum 10 years.<br>Must be cleanable and disinfectable with standard solutions.
Accessories	None.
Warranties	2 years on all components.
Instructions to Suppliers	None.
Note to Procurement	

Catalogue Number	Item Name	Available on Transversal Contract	Contract Number or Code
PHC-F-038	***Trolley: medical instruments, medium***	RT 24	RT 24-12-013

Functional Specifications	Trolley, medical dressing, medium.<br>To comply with the latest issue of SANS 726 (see fig 4).<br>Length: 915mm (± 5mm).<br>Width: 455mm (± 5mm).<br>The trolley is open and has two removable stainless steel shelves.<br>No guard on top shelf, and continuous guard rail on all four sides of bottom shelf.<br>Durable rubber plugs (or similar noise reducing material) must be inserted into rails below removable shelves.<br>It has a continuous guard rail along the sides and back of the top shelf and a continuous rail fitted around the bottom, above the bottom shelf.<br>Materials which are easily cleaned and of lasting finish, and low noise generating nature, shall be used. Stainless steel throughout.<br>Four swivel castors with a diameter of 75mm each. Where castors are fitted into steel tubular legs, the tube shall be of wall thickness not less than 2,0mm and castors shall be fixed to the tube by one of the following methods:<br>a. Solid plug (long) complying with SANS 621 subsection 3.5.6 or<br>b. Screwed into 35mm long sleeves welded into the tubular members and locked in an acceptable manner.
Applicable Standards	To comply with the latest issue of SANS 726, Type 2, size B.<br>Stainless steel must comply with the latest issue of SANS 417, subsection 4.1.2, proof of compliance must be submitted.<br>Castors shall be in accordance with SANS 621 (see Special Conditions).
Performance Specifications	Shelf size 912x455mm (±5mm).<br>Service life minimum 10 years.<br>Must be cleanable and disinfectable with standard solutions.
Accessories	None.
Warranties	2 years on all components.
Instructions to Suppliers	None.
Note to Procurement	

Catalogue Number	Item Name	Available on Transversal Contract	Contract Number or Code
PHC-F-037	***Trolley: medical dressing, small***	RT 24	RT 24-12-009

Functional Specifications	Trolley, medical dressing, small.<br>To comply with the latest issue of SANS 726 (see fig 4).<br>Length: 455mm (± 5mm).<br>Width: 455mm (± 5mm).<br>The trolley is open and has two removable stainless steel shelves.<br>Continuous guard rail on three sides of top shelf, and all four sides of bottom shelf.<br>Durable rubber plugs (or similar noise reducing material) must be inserted into rails below removable shelves.<br>It has a continuous guard rail along the sides and back of the top shelf and a continuous rail fitted around the bottom, above the bottom shelf.<br>Materials which are easily cleaned and of lasting finish, and low noise generating nature, shall be used. Stainless steel throughout.<br>Four swivel castors with a diameter of 75mm each. Where castors are fitted into steel tubular legs, the tube shall be of wall thickness not less than 2,0mm and castors shall be fixed to the tube by one of the following methods:<br>a. Solid plug (long) complying with SANS 621 subsection 3.5.6 or<br>b. Screwed into 35mm long sleeves welded into the tubular members and locked in an acceptable manner.
Applicable Standards	To comply with the latest issue of SANS 726, Type 4, size C.<br>Stainless steel must comply with the latest issue of SANS 417, subsection 4.1.2, proof of compliance must be submitted.<br>Castors shall be in accordance with SANS 621 (see Special Conditions).
Performance Specifications	Shelf size 455x455mm (±5mm).<br>Service life minimum 10 years.<br>Must be cleanable and disinfectable with standard solutions.
Accessories	None.
Warranties	2 years on all components.
Instructions to Suppliers	None.
Note to Procurement	

Catalogue Number	Item Name	Available on Transversal Contract	Contract Number or Code
PHC-F-044	***Trolley: medical dressing with bucket and bowl attachment***	RT 24-2016	RT 24-12-010

Functional Specifications	Trolley, medical dressing with bucket and bowl attachment. Type 6. The trolley is open and has two removable stainless steel shelves. Durable rubber plugs (or similar noise reducing material) must be inserted into rails below removable shelves. It has a continuous top guard rail around the four sides.<br>No guard rail on the bottom shelf. Bowl attachment (upper bracket) to accommodate a bowl (size 3) as per paragraphs 4 and 5.21 of SANS 417. Bucket attachment (lower bracket) to accommodate a 14l bucket as per paragraphs 4 and 5.18 of SANS 417. Materials which are easily cleaned and of lasting finish, and low noise generating nature, shall be used. Shelves to be of stainless steel. Stainless steel must comply with subsection 4.1.2 of the latest issue of SANS 417 (See special conditions), of compliance must be submitted. Four swivel castors with a diameter of 75 mm each. Castors shall be in accordance with SANS 621 (see Special Conditions). Where castors are fitted into steel tubular legs, the tube shall be of wall thickness not less than 2,0mm and castors shall be fixed to the tube by one of the following methods: i) Solid plug (long) complying with SANS 621 subsection 3.5.6 or ii) Screwed into 35mm long sleeves welded into the tubular members and locked in an acceptable manner.
Applicable Standards	To comply with the latest issue of SANS 726 (see fig 6).<br>SANS 417.<br>SANS 621.<br>Mild steel with epoxy/nylon powder-coated finish to comply with SANS 778 paragraph 5.2. (See Special Conditions). Proof of compliance must be submitted.
Performance Specifications	Expected service life: 15 years.
Accessories	Trolley to be supplied with a bucket and bowl as specified.
Warranties	2 years.
Instructions to Suppliers	
Note to Procurement	

Catalogue Number	Item Name	Available on Transversal Contract	Contract Number or Code
PHC-F-051	***Trolley: patient***	RT 24	RT 24-12-018

Functional Specifications	Trolley, patient, with mattress.<br>To comply with the latest issue of SANS 778. Type A (fig 1).<br>Adjustable backrest with collapsible safety sides.<br>Overall length of stretcher top: 2 060mm (± 5mm).<br>Stainless steel top with a medium directional satin finish.<br>Mild steel with epoxy/nylon powder-coated finish to comply with SANS 778 paragraph 5.2. (See Special Conditions).<br>Proof of compliance must be submitted.<br>Two hooks on the drip rod.<br>Oxygen cylinder holder.<br>Mounted on 4 heavy duty locking, swivel castors 125mm diameter, two swivel, two braking. Castors must be antistatic.<br>Castors shall be in accordance with SANS 621 (see Special Conditions).<br>Where castors are fitted into steel tubular legs, the tube shall be of wall thickness not less than 2,0mm and castors shall be fixed to the tube by one of the following methods:<br>c. Solid plug (long) complying with SANS 621 subsection 3.5.6 or<br>d. Screwed into 35mm long sleeves welded into the tubular members and locked in an acceptable manner.<br>**Mattress**: Must comply with item RT 24-12-019.
Applicable Standards	Stainless steel must comply with subsection 4.1.2 of the latest issue of SANS 417 (See special conditions). Proof of compliance must be submitted.<br>SANS 778 Type A, proof of compliance must be submitted.<br>SANS 778 paragraph 5.2.
Performance Specifications	Must support a patient of 180Kg mass.<br>Expected service life: 15 years.
Accessories	Safety sides, drip rod, oxygen cylinder holder.<br>Mattress for patient trolley PHC-F-052.<br>Cover for Mattress PHC-F-053.
Warranties	2 years.
Instructions to Suppliers	None.
Note to Procurement	Mattress RT 24-12-019 and mattress cover RT 24-12-019 in series with this item.

Catalogue Number	Item Name	Available on Transversal Contract	Contract Number or Code
PHC-F-054	***Trolley: soiled instruments and linen, complete***	RT 24-2016	RT 24-12-027

Functional Specifications	Trolley, soiled instruments and linen, complete with polyester linen receptacle.<br>Mild steel with epoxy/nylon powder-coated finish to comply with SANS 778 paragraph 5.2. Proof of compliance must be submitted.<br>The shelves shall be constructed of 1,2mm stainless steel sheeting.<br>The shelves shall be shaped to fit neatly between the legs of the trolley and be supported on all sides by 15mm OD tubular rails.<br>The shelves shall be free of sharp edges and shall be attached to the rails in a manner to eliminate vibrations between the shelves and the rails. The necessary fittings to achieve this shall be concealed.<br>The bottom shelf shall extend all the way to the fifth leg as indicated in drawing SANS 896 and shall be braced by a 15mm OD transverse member between the other two legs forming a triangle to support the weight of the canvas receptacle.<br>Polyester linen receptacle as per RT 24-12-028. The receptacles has eyelets for attachment to the frame, as well as a draw-cord and stitched-on handles.<br>**Size**: Diameter: 500mm (± 10mm).<br>**Height**: 800mm (± 10mm) including 100mm fold over lip.<br>The trolley shall be fitted with 75mm diameter castors.<br>Castors must comply with the latest issue of SANS 621, proof of compliance must be submitted.<br>Where castors are fitted into steel tubular legs, the tube shall be of wall thickness not less than 2,0mm and castors shall be fixed to the tube by one of the following methods:<br>a. Solid plug (long) complying with SANS 621 subsection 3.5.6<br>b. Screwed into 35mm long sleeves welded into the tubular members and locked in an acceptable manner<br>c. Rubber or plastic expanding sleeves for fitting castors are not acceptable.
Applicable Standards	SANS 778 paragraph 5.2. Proof of compliance must be submitted.<br>To comply with SANS 896 specifications.<br>SANS 621, proof of compliance must be submitted.
Performance Specifications	Cleanable with standard cleaning and disinfectant solutions.<br>Washable receptacle.<br>Expected service life: 15 years for trolley body.
Accessories	Woven polyester linen receptacle PHC-F-055.

Warranties	2 years.
Instructions to Suppliers	
Note to Procurement	Replacement linen receptacle RT 24-12-029 in series with this item, to use interchangeably with, or to replace, original receptacle.

Catalogue Number	Item Name	Available on Transversal Contract	Contract Number or Code
PHC-F-056	***Trolley: soiled linen, complete***	RT 24-2016	RT 24-12-030

Functional Specifications	Castor-mounted, epoxy-coated mild-steel trolley.<br>Vertical members support a horizontal circular or square frame, onto which a woven polyester soiled linen receptacle attaches.<br>The receptacles has eyelets for attachment to the frame, as well as a draw-cord and stitched-on handles.<br>Where castors are fitted into steel tubular legs, the tube shall be of wall thickness not less than 2,0mm and castors shall be fixed to the tube by one of the following methods:<br>a. Solid plug (long) complying with SANS 621 subsection 3.5.6; or<br>b. Screwed into 35mm long sleeves welded into the tubular members and locked in an acceptable manner.<br>(Rubber or plastic expanding sleeves for fitting castors are not acceptable.)
Applicable Standards	SANS 778 paragraph 5.2, Proof of compliance must be submitted.<br>SANS 896, Type A.<br>SANS 621, proof of compliance must be submitted.
Performance Specifications	Cleanable with standard cleaning and disinfectant solutions.<br>Expected service life: 15 years for trolley body.
Accessories	Woven polyester linen receptacle PHC-F-057.
Warranties	2 years.
Instructions to Suppliers	
Note to Procurement	Replacement linen receptacle RT 24-12-031 in series with this item, to use interchangeably with, or to replace, original receptacle.

Catalogue Number	Item Name	Available on Transversal Contract	Contract Number or Code
PHC-F-042	***X-Ray viewing box: wall mounted***	RT 24	RT 24-12-013

Functional Specifications	Viewing boxes for viewing radiographic images:<br>White powder-coated steel box, fitted with keyhole slots for wall mounting<br>1/2 fluorescent tubes, built-in high intensity bulb (100W or equiv. globe to be fitted), separate switches for normal and sharp lights spring-loaded film clips<br>Size: to fit 1x(350x430mm) film<br>Power cord 3 metres fixed to box, ending in moulded SABS approved 15A 3-pin plug.
Applicable Standards	None available.
Performance Specifications	Brightness 1 700 nit min 1 500 nit.
Accessories	None.
Warranties	1 year.
Instructions to Suppliers	Supplier to deliver, mount, connect to electricity supply.
Note to Procurement	None.

Catalogue Number	Item Name	Available on Transversal Contract	Contract Number or Code
PHC-F-024	***Wheelchair: porter***	RT 233-2017	42192210-00259

Functional Specifications	Porter chair for institutional use.<br>Size 51cm wide.
Applicable Standards	Technical spec 1.12.1 of RT 2-2016.
Performance Specifications	Expected service life: 10 years.
Accessories	
Warranties	2 years.
Instructions to Suppliers	
Note to Procurement	

General

Catalogue Number	Item Name	Available on Transversal Contract	Contract Number or Code
PHC-F-001	***Chair: office, mid-back***	No	

Functional Specifications	Must comply with SANS 660:12, with the following additions/modifications:<br>Chair manufactured from epoxy powder-coated mild steel frame without arm rests. Chair to swivel and tilt. Chair to be fitted with a "synchro" mechanism, adjustable for user up to 200kg. Height-adjustable with heavy-duty gas strut<br>Base: Mounted on a five-star (5) cast alloy (chromed or black powder coated) base. High-impact ABS or reinforced nylon/plastic will be considered, if of same strength as cast alloy (evidence to be provided). Base to be fitted with heavy duty double-wheel casters. Casters must fit securely and not fall out of base after prolonged use. Supplier to specify fixing method<br>Chair to have fully moulded shell. Chair to have a durable polyurethane back in black mesh. Mesh to be moulded in a frame, replaceable<br>Chair back to be of high type<br>The seat of the chair to be well upholstered in heavy-duty foam and covered with heavy duty impervious. easily-cleanable synthetic material. Upholstery material must be resistant to common cleaning agents, and to dilute 10% glutaraldehyde solution. Seat must be replaceable.
Applicable Standards	SANS 660:12, as modified herein.
Performance Specifications	Chair must support a body weight of 200 kg without flexing or deforming. Arm rests must not splay, loosen or break off when a heavy occupant leans on them to rise up.<br>Expected service life of at least 5 years.<br>Must be cleanable and disinfectable with standard solutions.
Accessories	
Warranties	Guarantee on seat: 2 years.<br>Guarantee on frame and back: 5 years.<br>Spares availability must be guaranteed for minimum of 5 years.
Instructions to Suppliers	Supplier to indicate available colours.<br>Supplier to indicate what spare parts are available, with pricing.<br>Supplier to deliver and train users on adjustments.
Note to Procurement	None.

Catalogue Number	Item Name	Available on Transversal Contract	Contract Number or Code
PHC-F-002	***Chair: operator, high back***	No	

Functional Specifications	Must comply with SANS 660:12, with the following additions/modifications:<br>Chair manufactured from epoxy powder-coated mild steel frame with adjustable arm rests. Chair to swivel and tilt. Chair to be fitted with a "synchro" mechanism, adjustable for user up to 200kg<br>Height-adjustable with heavy-duty gas strut<br>Base: Mounted on a five-star cast alloy (chromed or black powder coated) base. High-impact ABS or reinforced nylon/plastic will be considered, if of same strength as cast alloy (evidence to be provided). Base to be fitted with heavy duty double-wheel casters. Casters must fit securely and not fall out of base after prolonged use. Supplier to specify fixing method<br>Chair to have fully moulded shell. Chair to have a durable polyurethane back in black mesh. Mesh to be moulded in a frame, replaceable<br>Chair back to be of high type<br>The seat of the chair to be well upholstered in heavy-duty foam and covered with heavy duty impervious. easily-cleanable synthetic material. Upholstery material must be resistant to common cleaning agents, and to dilute 10% glutaraldehyde solution. Seat must be replaceable.
Applicable Standards	SANS 660:12, as modified herein.
Performance Specifications	Chair must support a body weight of 200 kg without flexing or deforming. Arm rests must not splay, loosen or break off when a heavy occupant leans on them to rise up.<br>Expected service life of at least 5 years.<br>Must be cleanable and disinfectable with standard solutions.
Accessories	
Warranties	Guarantee on seat: 2 years.<br>Guarantee on frame and back: 5 years.<br>Spares availability must be guaranteed for minimum of 5 years.
Instructions to Suppliers	Supplier to indicate available colours.<br>Supplier to indicate what spare parts are available, with pricing.<br>Supplier to deliver and train users on adjustments.
Note to Procurement	None.

Catalogue Number	Item Name	Available on Transversal Contract	Contract Number or Code
PHC-F-003	***Chair: stackable, ABS shell***	No	

Functional Specifications	Must comply with SANS 660:12, with the following additions/modifications:<br>The chair to be manufactured from mild steel tubing, which is to be chrome or black powder-coated epoxy-finished, with replaceable plastic anti-scratch anti-noise protector clips<br>The frame should preferably be of the sled type, anti-tilt in normal usage<br>No arms<br>Stackable design preferred<br>Chair to have fully moulded shell<br>Seat must be replaceable.
Applicable Standards	SANS 660:12, as modified herein.
Performance Specifications	Chair must support a body weight of 200 kg without flexing or deforming.<br>Expected service life of at least 5 years.<br>Must be cleanable and disinfectable with standard solutions.
Accessories	
Warranties	Guarantee on frame and back: 5 years.<br>Spares availability must be guaranteed for minimum of 5 years.
Instructions to Suppliers	Supplier to indicate available colours.<br>Supplier to indicate what spare parts are available, with pricing.
Note to Procurement	None.

Catalogue Number	Item Name	Available on Transversal Contract	Contract Number or Code
PHC-F-004	***Chair: stackable, netted, mid-back***	No	

Functional Specifications	Must comply with SANS 660:12, with the following additions/modifications:<br>The chair to be manufactured from mild steel tubing, which is to be chrome or black powder-coated epoxy-finished, with replaceable plastic anti-scratch anti-noise protector clips<br>The frame should preferably be of the sled type, anti-tilt in normal usage<br>No arms<br>Stackable design preferred<br>The seat of the chair to be well upholstered in heavy-duty foam and covered with heavy duty impervious. easily-cleanable synthetic material. Upholstery material must be resistant to common cleaning agents, and to dilute 10% glutaraldehyde solution. Seat must be replaceable<br>Chair to have a durable polyurethane back in black mesh. Mesh to be moulded in a frame, replaceable.
Applicable Standards	SANS 660:12, as modified herein.
Performance Specifications	Chair must support a body weight of 200 kg without flexing or deforming.<br>Expected service life of at least 5 years.<br>Must be cleanable and disinfectable with standard solutions.
Accessories	
Warranties	Guarantee on frame and back: 5 years.<br>Spares availability must be guaranteed for minimum of 5 years.
Instructions to Suppliers	Supplier to indicate available colours.<br>Supplier to indicate what spare parts are available, with pricing.
Note to Procurement	None.

Catalogue Number	Item Name	Available on Transversal Contract	Contract Number or Code
PHC-F-005	***Chair: visitor, mid back***	No	

Functional Specifications	Must comply with SANS 660:12, with the following additions/modifications:<br>The chair to be manufactured from mild steel tubing, which is to be chrome or black powder-coated epoxy-finished, with replaceable plastic anti-scratch anti-noise protector clips. The frame should preferably be of the sled type, anti-tilt in normal usage<br>No arms<br>Stackable design preferred<br>Chair to have fully moulded shell. Chair to have a durable polyurethane upholstered back in black mesh. Mesh to be moulded in a frame, replaceable<br>The seat of the chair to be well upholstered in heavy-duty foam and covered with heavy duty impervious. easily-cleanable synthetic material. Upholstery material must be resistant to common cleaning agents, and to dilute 10% glutaraldehyde solution. Seat must be replaceable.
Applicable Standards	SANS 660:12, as modified herein.
Performance Specifications	Chair must support a body weight of 200 kg without flexing or deforming.<br>Expected service life of at least 5 years.<br>Must be cleanable and disinfectable with standard solutions.
Accessories	
Warranties	Guarantee on seat 2 years.<br>Guarantee on frame and back: 5 years.<br>Spares availability must be guaranteed for minimum of 5 years.
Instructions to Suppliers	Supplier to indicate available colours.<br>Supplier to indicate what spare parts are available, with pricing.
Note to Procurement	None.

Catalogue Number	Item Name	Available on Transversal Contract	Contract Number or Code
PHC-F-006	***Credenza***	No	

Functional Specifications	Credenza to complement the L-shaped desk PHC-F-007.<br>**Carcass and doors**: Particleboard or medium density fibreboard, 16mm, clad in continuous decorative laminate. To be edge-banded in same material.<br>Doors to be edged (see edging, below), 2mm edging<br>Doors may be sliding type, in inlaid top and bottom rails, or hinged (Euro-hinge 35mm, inset type)<br>Two cladded shelves, adjustable spacing.<br>**Worktop to match that of desk**:<br>**Material**: Tops to be constructed of either moisture resistant particleboard (must comply with SABS EN 312-5: 2003, proof to be supplied) or medium density fibreboard (must comply with SABS 540-1: 1991, proof to be supplied). 20-22mm thickness preferred<br>**Work surface:** continuous decorative laminate, at least 0.8mm thick, complying with general purpose laminates, SANS 4586-2, bonded to the substrate. A high-pressure laminate may be proposed. Pale grey colour preferred, supplier to provide samples. Abrasion test results as per CKS 36:2004 or equivalent, to be supplied. The worktops are to be counter-laminated on the inferior surface<br>**Edging**: Polyurethane or high-impact thermoplastic. No self-adhesive laminate edging. At least 4mm thick. Rectangular cross-section preferred, to minimise gap when the two tops are placed in contiguity. Edging must be securely adhered to the substrate, without visible glue line, gaps or protruding edges. Edging (and the adhesive joint) must be impact-, water-, and heat-resistant, at upper and lower joints with laminate. Colour of edging may be different to colour of top laminate – supplier to provide colour range available<br>**Door handles**: simple, minimally-protruding type, suitable for operation with gloved hands. Non-corrosive, resistant to disinfectants. Wooden knobs and cheap plastic not acceptable.
Applicable Standards	Compliance with SANS 660:2012 is required, with particular reference to strength and impact requirements.<br>SABS EN 312-5: 2003.<br>SABS 540-1: 1991.<br>General Purpose laminates, SANS 4586-2.<br>CKS 36:2004 or equivalent.<br>Fittings, fasteners, clips, locks, rivets, washers, must comply with SANS 135 or equivalent.
Performance Specifications	Approximately 900mm wide by 450mm deep. Height to be same as desk.<br>Must be cleanable and disinfectable with standard solutions.<br>A minimum 10-year service life is desired.

Accessories	The unit to be supplied with a key and a spare key.
Warranties	Minimum guarantee 5 years on all components. Spares availability guaranteed for minimum of 5 years.
Instructions to Suppliers	Supplier to deliver and assemble.
Note to Procurement	These specifications are superior to the quality generally available at discount retail outlets. Inferior construction and materials, lead to vastly reduced service life.

Catalogue Number	Item Name	Available on Transversal Contract	Contract Number or Code
PHC-F-007	***Desk: L-shape, 3-drawer***	No	

Functional Specifications	Asymmetrical L-shaped desk, which can be configured for R- or L-handed orientation. Preference is for two individual tops, but single work surface can be proposed.<br>**Frame**:<br>Desk to have a self-supporting, metal frame. Tops must not form part of structural integrity. Frame of mild steel, epoxy powder-coated (comply with Type 2 of SANS 1274). Supplier to specify steel thickness. Preference is for open frame with individual legs at ground level, for easy floor cleaning. Legs to have suitable durable plugs and/or ferrules, with adequate capacity for levelling. Plug/Ferrule material must withstand normal cleaning materials used on flooring, as well as diluted bleach and 10% glutaraldehyde solution. Ferrules and levellers must be non-staining, non-corroding, non-rusting<br>If two separate frames are proposed, design must be such that user can swivel from side to side without obstruction, when frames are combined into L-shape.<br>**Worktops**:<br>**Material**: tops to be constructed of either moisture resistant particleboard (must comply with SABS EN 312-5: 2003, proof to be supplied) or medium density fibreboard (must comply with SABS 540-1: 1991, proof to be supplied). 20-22mm thickness preferred<br>**Work surface**: to be continuous decorative laminate, at least 0.8mm thick, complying with General Purpose laminates, SANS 4586-2, bonded to the substrate. A high-pressure laminate may be proposed. Pale grey colour preferred, supplier to provide samples. Abrasion test results as per CKS 36:2004 or equivalent, to be supplied. The worktops are to be counter-laminated on the inferior surface<br>**Edging**: Polyurethane or high-impact thermoplastic. No self-adhesive laminate edging. At least 4mm thick. Rectangular cross-section preferred, to minimise gap when the two tops are placed in contiguity. Edging must be securely adhered to the substrate, without visible glue line, gaps or protruding edges. Edging (and the adhesive joint) must be impact-, water-, and heat-resistant, at upper and lower joints with laminate. Colour of edging may be different to colour of top laminate – supplier to provide colour range available<br>**Modesty panels**: desk to have modesty panels for one long and one short length of the small unit (800x600). Supplier to propose material and available colours<br>**Drawer unit**: three- drawer fitted unit, (to mount under the left or right side of the longer top). Carcass of laminated particleboard or MDF, to match colour of worktop laminate. Carcass preferably to be affixed to steel frame<br>**Drawer construction**: epoxy-coated steel sides with integral roller runners and laminated board front preferred, but all laminated board construction with metal roller runners will be considered. Colour of drawer front may differ from colour of carcass. Supplier to provide samples of options. Drawer fronts may be fully recessed (preferred) or overlay. Drawer fronts to have durable edging. Top drawer to be lockable, supplied with two keys. Top drawer to have a pen and pencil storage tray. Top drawer 85mm side, remaining two drawers 150mm sides. Base of drawer minimum 16mm laminated particleboard, not 4mm hardboard. Drawer pulls must be of simple, minimally-protruding type, suitable for operation with gloved hands. Non-corrosive, resistant to disinfectants. Wooden knobs and cheap plastic not acceptable.

Applicable Standards	Compliance with SANS 660:2012 is required, with particular reference to strength and impact requirements.<br>Epoxy coating to comply with Type 2 of SANS 1274.<br>SABS EN 312-5: 2003.<br>SABS 540-1: 1991.<br>General Purpose laminates, SANS 4586-2.<br>CKS 36:2004 or equivalent.<br>Fittings, fasteners, clips, locks, rivets, washers, must comply with SANS 135 or equivalent.
Performance Specifications	**Worktops**: Dimensions: 1x 000mmx600mmx35mm rectangular top and 1x800x600 rectangular top, when fitted together will form an "L" shaped workstation. Overall height (on frame): 720-750mm.<br>Must be cleanable and disinfectable with standard solutions.<br>A minimum 10-year service life is desired.
Accessories	The unit to be supplied with a key and a spare key.<br>Pen and pencil storage tray.
Warranties	Minimum guarantee 5 years on all components. Spares availability guaranteed for minimum of 5 years.
Instructions to Suppliers	**Note**: for infection control purposes, the desk must be fixed in position. Provision must be made for brackets to fix a long edge of the larger worktop to a wall. The brackets may be part of the frame, or may attach under the worktop, depending on design. Brackets must not interfere with positioning of drawer unit. Supplier to supply brackets if not part of the construction, at no additional cost.<br>Supplier to deliver and assemble, but not fix to wall.
Note to Procurement	These specifications are superior to the quality generally available at discount retail outlets. Inferior construction and materials, lead to vastly reduced service life.

Catalogue Number	Item Name	Available on Transversal Contract	Contract Number or Code
PHC-F-008	***Desk, rectangular***	No	

Functional Specifications	Simple rectangular desk (optionally, one short edge may be curved, with radius = one-half of width).<br>**Frame**: Desk to have a self-supporting metal frame – tops must not form part of structural integrity. Frame of mild steel, epoxy powder-coated (comply with Type 2 of SANS 1274). Supplier to specify steel thickness. Preference is for open frame with individual legs at ground level, for easy floor cleaning. Legs to have suitable durable plugs and/or ferrules, with adequate capacity for levelling. Plug/Ferrule material must withstand normal cleaning materials used on flooring, as well as diluted bleach and 10% glutaraldehyde solution. Ferrules and levellers must be non-staining, non-corroding, non-rusting.<br>**Worktops:**<br>**Material**: tops are to be constructed of either moisture resistant particleboard (must comply with SABS EN 312-5: 2003, proof to be supplied) or medium density fibreboard (must comply with SABS 540-1: 1991, proof to be supplied). 20-22mm thickness preferred<br>**Work surface** to be continuous decorative laminate, at least 0.8mm thick, complying with general purpose laminates, SANS 4586-2, bonded to the substrate. A high- pressure laminate may be proposed. Pale grey colour preferred, supplier to provide samples. Abrasion test results as per CKS 36:2004 or equivalent, to be supplied. The worktops are to be counter-laminated on the inferior surface<br>**Edging**: Polyurethane or high-impact thermoplastic. No self-adhesive laminate edging. At least 4mm thick. Rectangular cross-section preferred, to minimise gap when the two tops are placed in contiguity. Edging must be securely adhered to the substrate, without visible glue line, gaps or protruding edges. Edging (and the adhesive joint) must be impact, water, and heat resistant, at upper and lower joints with laminate. Colour of edging may be different to colour of top laminate – supplier to provide colour range available<br>**Modesty panels**: desk to have modesty panels for one long and one short length of the unit (800x600). Supplier to propose material and available colours (may or may not be same as top work surface)<br>**Drawers**: two, approximately 150mm depth, approximately 400mm wide, suspended side-by-side under top or attached to frame. Can be all-steel epoxy-coated construction, on roller or ball-bearing slides. Base of drawer minimum 16mm laminated particleboard, not 4mm hardboard. Drawer pulls must be of simple, minimally-protruding type, suitable for operation with gloved hands. Non-corrosive, resistant to disinfectants. Wooden knobs and cheap plastic not acceptable.

Applicable Standards	Compliance with SANS 660:2012 is required, with particular reference to strength and impact requirements.<br>Epoxy coating to comply with Type 2 of SANS 1274.<br>SABS EN 312-5: 2003.<br>SABS 540-1: 1991.<br>General Purpose laminates, SANS 4586-2.<br>CKS 36:2004 or equivalent.<br>Fittings, fasteners, clips, locks, rivets, washers, must comply with SANS 135 or equivalent.
Performance Specifications	**Worktops**: Dimensions: 1 000x600 rectangular top. Overall height (on frame): 720-750mm.<br>Must be cleanable and disinfectable with standard solutions.<br>A minimum 10-year service life is desired.
Accessories	The unit to be supplied with a key and a spare key.<br>Pen and pencil storage tray.
Warranties	Minimum 5 years on all components. Spares availability guaranteed for minimum of 5 years.
Instructions to Suppliers	**Note**: for infection control purposes, the desk must be fixed in position. Provision must be made for brackets to fix a short edge of the worktop to a wall. The brackets may be part of the frame, or may attach under the worktop, depending on design. Brackets must not interfere with positioning of drawer unit. Supplier to supply brackets if not part of the construction, at no additional cost.<br>Supplier to deliver and assemble, but not fix to wall.
Note to Procurement	These specifications are superior to the quality generally available at "discount" retail outlets. Inferior construction and materials, lead to vastly reduced service life.

Catalogue Number	Item Name	Available on Transversal Contract	Contract Number or Code
PHC-F-009	***Filing cabinet: bulk***	No	

Functional Specifications	All-metal epoxy-coated movable filing cabinets, on levelled steel tracks. Pale grey colour preferred. Can have contrasting darker accents.<br>Each cabinet will be double-sided, except for the last cabinet (single-sided, stationary, usually against an end wall) and the first cabinet (single-sided, lockable)<br>Each cabinet (or bay) to have a maximum of 6 adjustable shelves – floor level not for general use<br>A fully loaded cabinet must be moved easily by hand pressure, otherwise must have a mechanism to multiply the force exerted by a user (e.g. lever or rotary gear)<br>Cabinets must be stable, non-tipping when fully loaded<br>Steel shelves, with rolled/formed front lip, no sharp edges<br>Shelves to be slotted for steel vertical dividers<br>First cabinet must lock securely when in the closed position<br>Access space between cabinets to be a minimum of 1 metre<br>Rubber bumpers on cabinets ensure quiet closing<br>Steel dividers, vertical, adjustable (not wire "toast-rack" type)<br>To have tabs which fit into slots on shelves, alternatively to grip rolled/formed front edge of shelf<br>Floor panels between rails to have heavy-duty synthetic carpeting.
Applicable Standards	POPI Act 4 of 2013. SANS 757:2004.
Performance Specifications	Each fully-loaded cabinet must not flex or distort.<br>Wheels to be securely seated in tracks, so that cabinets cannot derail.<br>Cabinet movement must be free, but with sufficient inertia not to roll without intention.<br>Epoxy coating according to SANS 778 paragraph 2, proof of compliance must be submitted.<br>Expected service life: minimum 15 years.
Accessories	Vertical dividers: steel PHC-F-010.<br>File-out cards PHC-C-005.
Warranties	5 years on cabinets, shelves, roller mechanisms, track, floor panels and locks.<br>!0-year guarantee on spares availability.

Instructions to Suppliers	Supplier to visit site for measurement, define needs, supply sketch of proposed number of cabinets and layout.<br>Specify the minimum floor loading strength required to carry fully-loaded cabinets.<br>Advise on any additional lighting which may be required for efficient storage or retrieval.<br>Deliver and assemble. Lay and level tracks. Commission system before handover. Train end-users in correct use of the system.<br>Provide details and costing of after-sales support.<br>Stipulate frequency of mandatory service.
Note to Procurement	Ensure that the correct metal vertical dividers are in series with this item – they are not necessarily compatible with systems from a different vendor.<br>Ensure that floor has been certified by Provincial engineering personnel to support floor loading, before finalising procurement.

Catalogue Number	Item Name	Available on Transversal Contract	Contract Number or Code
PHC-F-012	***Filing cabinet: 4-drawer***	No	

Functional Specifications	Conventional 4-drawer all-steel filing cabinet.<br>Cabinet manufactured from high quality sheet metal and finished with baked epoxy coating<br>Colour pale beige with darker accents<br>Lockable; single key locks all drawers, with an internal locking mechanism.
Applicable Standards	SANS 757:2004, POPI Act 4 of 2013.
Performance Specifications	No plastic structural components, no flimsy tin components in drawer slides or locking mechanism.<br>Must be cleanable and disinfectable with standard solutions.
Accessories	Must be supplied with 2 keys.
Warranties	Unit to have a minimum 2-year guarantee on carcass. 5-year guarantee on locks, lock bars, drawer slides and handles.
Instructions to Suppliers	Supplier to indicate what spare parts are available, with pricing.
Note to Procurement	Stationery cupboard PHC-F-18 to have same colours as this item.

Catalogue Number	Item Name	Available on Transversal Contract	Contract Number or Code
PHC-F-020	**Floor polisher: electric, single disc**	No	

Functional Specifications	Commercial-duty single disc electric floor polishing machine.<br>Motor at least 1400W.<br>With self-feeding hopper for cleaning and stripping solutions.<br>Power cable at least 5m.
Applicable Standards	SANS 60335-2-10, SANS 60335-2-67.
Performance Specifications	Must scrub and polish floors, and strip old sealer with the appropriate discs.<br>Cleanable with standard cleaning and disinfectant solutions.<br>Chemical resistant.
Accessories	Standard cleaning disc.
Warranties	2 years.
Instructions to Suppliers	Supplier to deliver and train cleaning personnel in its use.<br>Supplier to state service/maintenance requirements, and to quote post-sales support with pricing.
Note to Procurement	A general purpose cleaning disc PHC-E-012 and a stripping disc PHC-E-013 must be in series with this machine.

Catalogue Number	Item Name	Available on Transversal Contract	Contract Number or Code
PHC-F-021	***Microwave oven***	No	

Functional Specifications	Standard manual microwave oven.<br>Controls for power and cooking time.<br>25-30l, 900W.<br>Stainless steel interior preferred.
Applicable Standards	SANS 60335-2-25, SANS 60335-2-90.
Performance Specifications	5-year design life.
Accessories	Oven dish and carousel.
Warranties	2 years.
Instructions to Suppliers	State available after-sales support.
Note to Procurement	None.

Catalogue Number	Item Name	Available on Transversal Contract	Contract Number or Code
PHC-F-014	***Mirror***	No	

Functional Specifications	Mirror, glass, 300x400 mmx3mm thick, in frame (natural anodised aluminium preferred), pre-drilled for wall mounting. Back to be sealed to keep out dust and moisture.
Applicable Standards	None available.
Performance Specifications	Must be cleanable and disinfectable with standard solutions.
Accessories	To be supplied with wall mounting hardware (stainless steel screws).
Warranties	Minimum 1 year on frame.
Instructions to Suppliers	None.
Note to Procurement	

Catalogue Number	Item Name	Available on Transversal Contract	Contract Number or Code
PHC-F-022	***Refrigerator: bar type***	No	

Functional Specifications	Domestic small bar-type refrigerator, 60-90l capacity.<br>For laboratory specimens only.
Applicable Standards	Refrigerant: CFC free.<br>Certificate: ISO9001, ISO14001, ISO13485, DIN 58345, SANS 60335-2-24.
Performance Specifications	At least A+ grading on energy efficiency.
Accessories	
Warranties	2 years.
Instructions to Suppliers	
Note to Procurement	

Catalogue Number	Item Name	Available on Transversal Contract	Contract Number or Code
PHC-F-047	***Refrigerator: double door***	No	

Functional Specifications	Upright type, double- door, min. 600l.<br>Operating temperature: +2℃ to +8℃ at ambient temperature +5℃ to 35℃ and +60% humidity.<br>Optionally the supplier can propose more than one model for use in moderate and hot temperature zones.<br>Automatic defrost.<br>Thermostat control should be graduated in Degrees C, with 1 degree increments. Display accuracy: 0.1C.<br>Ideally fridge and freezer compartments should have separate controls.<br>Glass door not a prerequisite.<br>Self-closing, lockable doors.<br>Forced air circulation system with intelligent control.<br>Audible and visual alarm for High or low temperature.<br>Door ajar alarm, system failure alarm.<br>4 casters mounted under the bottom (can be 2 fixed, 2 rotating).<br>Interior LED or fluorescent lighting.<br>5/6units shelves made of quality plastic- or epoxy- coated steel wire.<br>Exterior made from high quality structural plate, interior made from ABS material.<br>Visual and audible warning device for high and low temperature alarm (Min/Max thermometer).<br>Safety thermostat to avoid minus temperatures.<br>Built-in elements must be resistant to mechanical damage.<br>Good quality compressor (1 unit) and fan motor.<br>Power supply: 220V/50Hz 1 phase.
Applicable Standards	At least A+ grading on energy efficiency.<br>Refrigerant: CFC free.<br>Certificate: ISO9001, ISO14001, ISO13485, DIN 58345, SANS 60335-2-24.
Performance Specifications	Noise emission: < 60 dB(A).<br>Warning device for min. 12h in the event of power failure (power failure alarm).<br>Temperature holdover period 4 hours or better, in case of power failure.<br>Must be cleanable and disinfectable with standard materials.
Accessories	None.
Warranties	2 years on compressor, 5 years on other components.
Instructions to Suppliers	Includes delivery, installation, training.<br>Supplier must specify available after-sales support, with relevant pricing.
Note to Procurement	None.

Catalogue Number	Item Name	Available on Transversal Contract	Contract Number or Code
PHC-F-043	***Refrigerator: single door***	No	

Functional Specifications	Upright type, single door, approximately 260l.<br>Operating temperature: +2°C to +8°C at ambient temperature +5°C to 35°C and +60% humidity.<br>Optionally the supplier can propose more than one model for use in moderate and hot temperature zones.<br>Automatic defrost.<br>Thermostat control should be graduated in degrees C, with 1 degree increments. Display accuracy: 0.1C.<br>Ideally fridge and freezer compartments should have separate controls.<br>Glass door not a prerequisite.<br>Self-closing, lockable door.<br>Forced air circulation system with intelligent control.<br>Audible and visual alarm for high or low temperature.<br>Door ajar alarm, system failure alarm.<br>4 casters mounted under the bottom (can be 2 fixed, 2 rotating).<br>Interior LED or fluorescent lighting.<br>5/6units shelves made of quality plastic- or epoxy- coated steel wire.<br>Exterior made from high quality structural plate, interior made from ABS material.<br>Visual and audible warning device for high and low temperature alarm (Min/Max thermometer).<br>Safety thermostat to avoid minus temperatures.<br>Built-in elements must be resistant to mechanical damage.<br>Good quality compressor (1 unit) and fan motor.<br>Power supply: 220V/50Hz 1 phase.
Applicable Standards	At least A+ grading on energy efficiency.<br>Refrigerant: CFC free.<br>Certificate: ISO9001, ISO14001,ISO13485, DIN 58345, SANS 60335-2-24.
Performance Specifications	Noise emission: < 60 dB(A).<br>Warning device for min. 12h in the event of power failure (power failure alarm).<br>Temperature holdover period 4 hours or better, in case of power failure.<br>Must be cleanable and disinfectable with standard materials.
Accessories	None.
Warranties	2 years on compressor, 5 years on other components.
Instructions to Suppliers	Includes delivery, installation and training.<br>Supplier must specify availability of after-sales support, with relevant pricing.
Note to Procurement	None.

Catalogue Number	Item Name	Available on Transversal Contract	Contract Number or Code
PHC-F-015	***Seating: waiting area, 2-seater***	No	

Functional Specifications	All metal bench style seating for waiting areas – 2-seater.<br>**Arms** should only be present at either end of the unit; no intermediate arms.<br>**Legs and arms** to be chrome-plated (supplier to indicate the base metal or alloy and the thickness of chrome-plating).<br>**Legs** to have built-in non-removable levelling feet, of non-corroding, non-disintegrating material. Must withstand cleaning with dilute bleach or 10% glutaraldehyde solution. Specify method by which levellers are attached to legs.<br>**Horizontal frame** bar of mild steel, chromed or epoxy powder-coated (comply with Type 2 of SANS 1274). Supplier to indicate steel thickness. Supplier to indicate available colours of powder coating. Extruded bar acceptable provided no decrease in tensile strength.<br>**Seat and backrest**: stainless steel preferred. Supplier to provide option for cold rolled steel, powder-coated silver finish. Seat and backrest may be one or two-piece, depending on design. In either case, all edges must be rigidly supported by the frame of the individual seat, such that the edge of the rolled sheet metal is not loose (enclosed edge preferred).<br>**Seat frames** to be individually attached to the horizontal frame bar. Supplier to declare mode of attachment.
Applicable Standards	Epoxy coating to comply with Type 2 of SANS 1274.<br>Fittings, fasteners, clips, locks, rivets, washers, must comply with SANS 135 or equivalent.
Performance Specifications	Each individual seat must be rated for a 200kg user. The unit as a whole must be rated for 400kg. Rating means that neither the individual seat nor the unit as a whole will deform, deflect or rack, at maximum load.<br>Must be cleanable and disinfectable with standard solutions.<br>A minimum 10-year service life is desired.
Accessories	None.
Warranties	Minimum 5 years on all components. Spares availability guaranteed for minimum of 5 years.
Instructions to Suppliers	Supplier to deliver and assemble, install and level.
Note to Procurement	These specifications are superior to the quality generally available at "discount" retail outlets. Inferior construction and materials, lead to vastly reduced service life. Cheap units are susceptible to rust, distortion, and premature failure.

Catalogue Number	Item Name	Available on Transversal Contract	Contract Number or Code
PHC-F-016	***Seating: waiting area, 3-seater***	No	

Functional Specifications	All metal bench style seating for waiting areas – 3-seater.<br>**Arms** should only be present at either end of the unit; no intermediate arms.<br>**Legs and arms** to be chrome-plated (Supplier to indicate the base metal or alloy and the thickness of chrome-plating).<br>**Legs** to have built-in non-removable levelling feet, of non-corroding, non-disintegrating material. Must withstand cleaning with dilute bleach or 10% glutaraldehyde solution. Specify method by which levellers are attached to legs.<br>**Horizontal frame** bar of mild steel, chromed or epoxy powder-coated (comply with Type 2 of SANS 1274). Supplier to indicate steel thickness. Supplier to indicate available colours of powder coating. Extruded bar acceptable provided no decrease in tensile strength.<br>**Seat and backrest**: stainless steel preferred. Supplier to provide option for cold rolled steel, powder-coated silver finish. Seat and backrest may be one or two-piece, depending on design. In either case, all edges must be rigidly supported by the frame of the individual seat, such that the edge of the rolled sheet metal is not loose (enclosed edge preferred).<br>**Seat frames** to be individually attached to the horizontal frame bar. Supplier to declare mode of attachment.
Applicable Standards	Epoxy coating to comply with Type 2 of SANS 1274.<br>Fittings, fasteners, clips, locks, rivets, washers, must comply with SANS 135 or equivalent.
Performance Specifications	Each individual seat must be rated for a 200kg user. The unit as a whole must be rated for 600kg. Rating means that neither the individual seat nor the unit as a whole will deform, deflect or rack, at maximum load.<br>Must be cleanable and disinfectable with standard solutions.<br>A minimum 10-year service life is desired.
Accessories	None.
Warranties	Minimum 5 years on all components. Spares availability guaranteed for minimum of 5 years.
Instructions to Suppliers	Supplier to deliver and assemble, install and level.
Note to Procurement	These specifications are superior to the quality generally available at "discount" retail outlets. Inferior construction and materials, lead to vastly reduced service life. Cheap units are susceptible to rust, distortion, and premature failure.

Catalogue Number	Item Name	Available on Transversal Contract	Contract Number or Code
PHC-F-017	***Seating: waiting area, 4-seater***	No	

Functional Specifications	All metal bench style seating for waiting areas – 4-seater.<br>**Arms** should only be present at either end of the unit; no intermediate arms.<br>**Legs and arms:** to be chrome-plated (Supplier to indicate the base metal or alloy and the thickness of chrome-plating).<br>**Legs** to have built-in non-removable levelling feet, of non-corroding, non-disintegrating material. Must withstand cleaning with dilute bleach or 10% glutaraldehyde solution. Specify method by which levellers are attached to legs.<br>**Horizontal frame** bar of mild steel, chromed or epoxy powder-coated (comply with Type 2 of SANS 1274). Supplier to indicate steel thickness. Supplier to indicate available colours of powder coating. Extruded bar acceptable provided no decrease in tensile strength.<br>**Seat and backrest**: stainless steel preferred. Supplier to provide option for cold rolled steel, powder-coated silver finish. Seat and backrest may be one or two-piece, depending on design. In either case, all edges must be rigidly supported by the frame of the individual seat, such that the edge of the rolled sheet metal is not loose (enclosed edge preferred).<br>**Seat frames** to be individually attached to the horizontal frame bar. Supplier to declare mode of attachment.
Applicable Standards	Epoxy coating to comply with Type 2 of SANS 1274.<br>Fittings, fasteners, clips, locks, rivets, washers, must comply with SANS 135 or equivalent.
Performance Specifications	Each individual seat must be rated for a 200kg user. The unit as a whole, must be rated for 800kg. Rating means that neither the individual seat nor the unit as a whole,will deform, deflect or rack, at maximum load.<br>Must be cleanable and disinfectable with standard solutions.<br>A minimum 10-year service life is desired.
Accessories	None.
Warranties	Minimum 5 years on all components. Spares availability guaranteed for minimum of 5 years.
Instructions to Suppliers	Supplier to deliver and assemble, install and level.
Note to Procurement	These specifications are superior to the quality generally available at "discount" retail outlets. Inferior construction and materials, lead to vastly reduced service life. Cheap units are susceptible to rust, distortion, and premature failure.

Catalogue Number	Item Name	Available on Transversal Contract	Contract Number or Code
PHC-F-018	***Stationery cabinet***	No	

Functional Specifications	Cabinet manufactured from high quality sheet metal and finished with baked epoxy coating.<br>Unit to have 6 adjustable steel shelves.<br>The unit to be supplied with lockable doors with a handle on one or both doors.<br>Colour: pale beige with darker accents.
Applicable Standards	SANS 757:2004.
Performance Specifications	**Dimensions**: Approximately (H) 1800mm x (W) 900mm x (D) 450mm.<br>No plastic hinges, no flimsy tin components in hinges or locking mechanism.<br>Must be cleanable and disinfectable with standard solutions.
Accessories	The unit to be supplied with a key and a spare key.
Warranties	Unit to have a minimum 2-year guarantee on carcass. 5-year guarantee on locks, lock bars, hinges and handles.
Instructions to Suppliers	Supplier to indicate what spare parts are available, with pricing.
Note to Procurement	4-drawer filing cabinet PHC-F-012 to have matching colours to this item.

Catalogue Number	Item Name	Available on Transversal Contract	Contract Number or Code
PHC-F-011	***Sterile stock cabinet***	No	

Functional Specifications	Cabinet manufactured from high quality sheet metal and finished with baked epoxy coating.<br>Colour: white.<br>Unit to have 6 adjustable steel shelves.<br>The unit to be supplied with lockable doors with a handle on one or both doors.<br>Doors to have glazed panels with fitted 3mm glass panes.<br>Interior to have anti-microbial coating.
Applicable Standards	SANS 757:2004.
Performance Specifications	**Dimensions**: Approximately (H) 1 800mm x (W) 900mm x (D) 450mm.<br>No plastic hinges, no flimsy tin components in hinges or locking mechanism.<br>Must be cleanable and disinfectable with standard solutions.
Accessories	The unit to be supplied with a key and a spare key.
Warranties	Unit to have a minimum 2-year guarantee on carcass. 5-year guarantee on locks, lock bars, hinges and handles.
Instructions to Suppliers	Supplier to indicate what spare parts are available, with pricing.<br>Supplier to provide cleaning instructions to preserve antimicrobial coating.
Note to Procurement	4-drawer filing cabinet PHC-F-012 to have matching colours to this item.

Catalogue Number	Item Name	Available on Transversal Contract	Contract Number or Code
PHC-F-067	***Storage rack: exercise ball, wall-mounted***	No	

Functional Specifications	Epoxy-coated wall-mounted mild steel frame, with spacing between bars to accommodate at least 4 balls, between 45cm and 85cm diameter. Side pieces to prevent ball from rolling off. Diagonal stays to wall, to prevent downward deflection if excessive weight is applied.<br>An alternative design could consist of a series of wall-mounted metal rings, with diameters 10cm smaller than each ball size, mounted sufficiently distant from the backing wall to accommodate the matching ball. Rings could be arranged vertically, if space does not permit horizontal mounting.
Applicable Standards	SANS 778 paragraph 5.2.
Performance Specifications	Ball sizes 45cm, 55cm, 65cm, 75cm, 85cm.<br>Expected service life at least 10 years.
Accessories	All wall-mounting hardware.
Warranties	2 years.
Instructions to Suppliers	Must be mounted on the wall at a height to allow easy access by all staff, but maximise wall and floor space below the frame.
Note to Procurement	

Catalogue Number	Item Name	Available on Transversal Contract	Contract Number or Code
PHC-F-068	***Table: folding***	No	

Functional Specifications	Portable folding table, for outreach teams. Minimum size 120 cm X 75cm (larger preferred), height 70-75cm.<br>Impact- and scratch-resistant polyethylene top. Dark colour preferred.<br>Aluminium fame preferred. Legs fold under top and stay in folded position.<br>Top may be single piece or preferably two hinged pieces, for ease of transportation.<br>Carrying handle.
Applicable Standards	
Performance Specifications	Must support at least 100kg total load on top, without appreciable deflection.<br>Expected service life at least 5 years.
Accessories	None.
Warranties	1 year.
Instructions to Suppliers	
Note to Procurement	

Catalogue Number	Item Name	Available on Transversal Contract	Contract Number or Code
PHC-F-019	***Table: trapezoidal***	No	

Functional Specifications	Trapezoidal table.<br>**Frame**: table to have a self-supporting metal frame; tops must not form part of structural integrity. Frame of mild steel, epoxy powder-coated (comply with Type 2 of SANS 1274). Supplier to specify steel thickness. Preference is for open frame with individual legs at ground level, for easy floor cleaning. Legs to have suitable durable plugs and/or ferrules, with adequate capacity for levelling. Plug/Ferrule material must withstand normal cleaning materials used on flooring, as well as diluted bleach and 10% glutaraldehyde solution. Ferrules and levellers must be non-staining, non-corroding, non-rusting.<br>**Worktops**:<br>**Material** : Tops are to be constructed of either moisture resistant particleboard (must comply with SABS EN 312-5: 2003, proof to be supplied) or medium density fibreboard (must comply with SABS 540-1: 1991, proof to be supplied). 11-18mm thickness<br>**Work surface** to be continuous decorative laminate, at least 0.8mm thick, complying with general purpose laminates, SANS 4586-2, bonded to the substrate. A high- pressure laminate may be proposed<br>**Pale grey colour** preferred, supplier to provide samples. Abrasion test results as per CKS 36:2004 or equivalent, to be supplied. The worktops are to be counter-laminated on the inferior surface<br>**Edging**: Polyurethane or high-impact thermoplastic. No self-adhesive laminate edging. At least 4mm thick. Rectangular cross-section preferred, to minimise gap when the two tops are placed in contiguity. Edging must be securely adhered to the substrate, without visible glue line, gaps or protruding edges. Edging (and the adhesive joint) must be impact, water, and heat resistant, at upper and lower joints with laminate. Colour of edging may be different to colour of top laminate – supplier to provide colour range available.
Applicable Standards	Compliance with SANS 660:2012 is required, with particular reference to strength and impact requirements.<br>Epoxy coating to comply with Type 2 of SANS 1274.<br>SABS EN 312-5: 2003.<br>SABS 540-1: 1991.<br>General Purpose laminates, SANS 4586-2.<br>CKS 36:2004 or equivalent.<br>Fittings, fasteners, clips, locks, rivets, washers, must comply with SANS 135 or equivalent.

Performance Specifications	**Worktops**: Dimensions: 1 200x600 trapezoidal top. Overall height (on frame): 720-750mm.<br>Must be cleanable and disinfectable with standard solutions.<br>A minimum 10-year service life is desired.
Accessories	None.
Warranties	Minimum 5 years on all components. Spares availability guaranteed for minimum of 5 years.
Instructions to Suppliers	Supplier to deliver and assemble, if KDU.
Note to Procurement	These specifications are superior to the quality generally available at "discount" retail outlets. Inferior construction and materials, lead to vastly reduced service life.

Catalogue Number	Item Name	Available on Transversal Contract	Contract Number or Code
PHC-F-045	***Tumble dryer***	No	

Functional Specifications	Large capacity electric tumble drier.<br>Front-loading.<br>Vented type – must be mounted against outside wall, with hole through the wall for moisture exhaust hose.<br>Can be mounted on the wall at eye-level to minimise floor space usage.<br>Auto-shutoff at end of drying cycle.<br>Single-phase electricity supply.
Applicable Standards	At least A grading on energy efficiency.<br>SANS 60335-2-11.
Performance Specifications	Must be suitable for humid coastal climate.<br>Minimum 10kg load.<br>Cleanable with standard cleaning and disinfectant solutions.<br>Chemical resistant.<br>Expected service life: 10 years.
Accessories	All wall-mounting hardware (stainless steel screws), venting hose, spare lint filter (if replaceable).
Warranties	3 years.
Instructions to Suppliers	Supplier to deliver and install in co-operation with departmental technical personnel.
Note to Procurement	

Catalogue Number	Item Name	Available on Transversal Contract	Contract Number or Code
PHC-F-046	***Vacuum cleaner***	No	

Functional Specifications	Large domestic canister–type machine.<br>At least 1800 W motor, 2000W preferred.<br>Bagless type preferred.<br>Washable HEPA filters
Applicable Standards	At least A grading on energy efficiency.<br>SANS 60335-2-2.
Performance Specifications	Chemical resistant.<br>Expected service life: 5 years.
Accessories	Complete with flexible hose, extension tubes, brushes and attachments, spare filters, instruction manual.
Warranties	2 years.
Instructions to Suppliers	
Note to Procurement	Spare HEPA filters to be procured in series with this machine.

Catalogue Number	Item Name	Available on Transversal Contract	Contract Number or Code
PHC-F-058	***Washing machine: front loader***	No	

Functional Specifications	Front-loading automatic washing machine.<br>Direct drive.<br>Pre-set washing, rinsing and spinning cycles.<br>Auto-shutoff when cycle complete.<br>Cold water connection only.<br>Stainless steel drum.
Applicable Standards	Minimum A++ electrical efficiency rating.
Performance Specifications	Minimum 8kg dry load.<br>Cleanable with standard cleaning and disinfectant solutions.<br>Chemical resistant.
Accessories	
Warranties	3 years.
Instructions to Suppliers	
Note to Procurement	

Catalogue Number	Item Name	Available on Transversal Contract	Contract Number or Code
PHC-F-048	***Washing machine: top loader***	No	

Functional Specifications	Large capacity electric washing machine.<br>Top-loader.<br>Automatic, with pre-set washing, rinsing and spinning cycles.<br>Auto-shutoff when cycle complete.<br>Cold water supply only.<br>Single-phase electricity supply.
Applicable Standards	At least A grading on energy efficiency.<br>SANS 60335-2-7.
Performance Specifications	Minimum 15kg load.<br>Cleanable with standard cleaning and disinfectant solutions.<br>Chemical resistant.<br>Expected service life: 10 years.
Accessories	
Warranties	3 years.
Instructions to Suppliers	Supplier to deliver and install in co-operation with departmental technical personnel.
Note to Procurement	

appendix 2

List of Ideal Clinic and Ideal Community Health Centre elements pertaining to equipment, instruments, consumables and furniture

Ideal Clinic Checklists

CHECKLIST FOR ELEMENT: STAFF WEAR APPROPRIATE PERSONAL PROTECTIVE CLOTHING

Use the checklist below to check whether protective clothing is available and worn

Item	
Gloves – non sterile	PHC-C-141, 142, 143
Gloves – sterile	PHC-C-138, 139, 140
Disposable gowns OR aprons	PHC-C-076, 137
Protective face shields OR goggles with surgical face masks	PHC-C-080, 079, 077

CHECKLIST FOR ELEMENT: BASIC MEDICAL SUPPLIES (CONSUMABLES) ARE AVAILABLE

Use the checklist below to check availability of medical and dressing supplies

SURGICAL SUPPLIES	
**Item**	
Intravenous administration set 20 drops/ml	PHC-C-095
Intravenous administration set paeds 60 drops/ml	PHC-C-096
Blade stitch cutter sterile/pack	PHC-C-145
Blood collecting vacutainer (holding barrel/bulldog)	
Blood lancets (haemolance)	PHC-C-146
Urinary (Foley's) catheter silicone/latex 14f	PHC-C-127
Urinary (Foley's) catheter silicone/latex 18f	PHC-C-128
Urine drainage bag	PHC-C-129
Simple face mask OR reservoir mask OR nasal cannula (prongs) for oxygen, adults	PHC-C-108,106,111
Simple face mask OR reservoir mask OR nasal cannula (prongs) for oxygen, paediatric	PHC-C-110,105,107
Face mask for nebuliser OR face mask with nebuliser chamber for adult	PHC-C-107
Face mask for nebuliser OR face mask with nebuliser chamber for paediatric	PHC-C-108
Nasogastric feeding tube 600mm fg8	PHC-C-342
Nasogastric feeding tube 1000mm fg10 OR 12	PHC-C-130
Disposable aprons	PHC-C-137
Eye patches (disposable)	PHC-C-165
Disposable razors	PHC-C-166
Gloves exam n/sterile large /box	PHC-C-143
Gloves exam n/sterile medium /box	PHC-C-142
Gloves exam n/sterile small /box	PHC-C-141
Gloves surg sterile latex sz 6 OR 6.5 OR small/box	PHC-C-138
Gloves surg sterile latex sz 7 OR 7.5 OR medium/box	PHC-C-139

Gloves surg sterile latex sz 8 OR large/box	PHC-C-140
Intravenous cannula (Jelco) 18g green/box	PHC-C-097
Intravenous cannula (Jelco) 20g pink/box	PHC-C-098
Intravenous cannula (Jelco) 22g blue/box	PHC-C-099
Intravenous cannula (Jelco) 24g yellow/box	PHC-C-100
Needles: 18 (pink) OR 20 (yellow)/box	PHC-C-122,123
Needles: 21 (green)/box	PHC-C-124
Needles: 23 (blue)/box OR 22 (black)/box	PHC-C-125
* Syringes 3-part 2ml/box	PHC-C-118
* Syringes 3-part 5ml/box	PHC-C-119
* Syringes 3-part 10 or 20ml/box	PHC-C-120, 349
Insulin syringe with needle/box	PHC-C-121
Suture chromic g0/0 or g1/0 1/2 75cm	PHC-C-181
Suture nylon g2/0 or g3/0 3/8 45cm	PHC-C-182
Suture nylon g4/0 3/8 45cm	PHC-C-098
**Only applicable if the facility uses older HB model**	
Haemolysis applicator sticks	PHC-C-167
HB meter clip	PHC-C-168
HB chamber glass-grooved	PHC-C-169
HB cover glass-plain	PHC-C-170
**Only applicable if facility uses an Automatic External Defibrillator (AED)**	
Replacement pads for AED - adult	PHC-C-174
Replacement pads for AED – paediatric	PHC-C-175
**Only applicable if facilities have a permanent doctor**	
Disposable Amnihook	PHC-C-171
Ultrasound gel medium viscosity	PHC-C-173
Dental syringe and needle for LA	PHC-C-180

DRESSINGS SUPPLIES		
**Item**	**Pack size**	
Plaster roll	1	PHC-C-153
Bandage crepe	1	PHC-C-147
Gauze paraffin 100x100 /box	1	PHC-C-152
Gauze swabs plain n/s 100x100x8ply/pack	100	PHC-C-151
Basic disposable dressing pack (should contain a minimum of: cotton-wool balls, swabs, 2 forceps, disposable drape)	1	PHC-C-150
Cotton wool balls 1g 500`s	1	PHC-C-149
Sanitary towels maternity /pack	12	PHC-C-156
Stockinette 100mm OR150mm/roll	1	PHC-C-154, 186
Adhesive micro-porous surgical tape 24/25mm or 48/50mm	1	PHC-C-159, 185
70% isopropyl alcohol prep pads 24x30 1ply OR 2 ply /box	200	PHC-C-155
Gauze abs grade 1 burn /pack		PHC-C-150

* Syringe three part consists of the barrel, the plunger and the rubber piston

CHECKLIST FOR ELEMENT: REQUIRED FUNCTIONAL DIAGNOSTIC EQUIPMENT AND CONCURRENT CONSUMABLES FOR POINT OF CARE TESTING ARE AVAILABLE

Use the checklist below to check the availability of laboratory equipment and consumables in the various areas where they are used

Item	
**Laboratory equipment and consumables**	
Hb meter	PHC-E-152
Blood glucometer	PHC-E-149
Spare batteries for blood glucometer	
Glass slides for cervical smears	
Lancets	PHC-C-146
Blood glucose strips	PHC-C-181
Urine dipsticks	PHC-C-205
Urine specimen jar OR flask	PHC-E-151, 158
Malaria rapid test (where applicable in facilities in KZN, GP, MP and LP)	PHC-C-200
Rapid HIV test	PHC-C-199
Rh ‘D’ (Rhesus factor) test	PHC-C-204

CHECKLIST FOR ELEMENT: DISINFECTANT, CLEANING MATERIALS AND EQUIPMENT ARE AVAILABLE

Use the checklist below to check whether the disinfectant, cleaning materials and equipment are available

**Disinfectant and cleaning Material**	
High-level disinfection for medical equipment (e.g. sodium perborate powder OR phthalaldehyde)	PHC-C-063, 034, 035
Chlorine compounds (e.g. Biocide D or Clorox)	PHC-C-062
Sanitary all-purpose cleaner	PHC-C-136
Detergent-based solutions	PHC-C-065
Wet polymer (floor polish)	PHC-C-066
Protective polymer (strippers)	PHC-C-067
All cleaning materials clearly labelled	
Materials Safety Data Sheets for all cleaning products	
**Cleaning equipment**	
Two-way bucket system for mopping floors (bucket for clean water and bucket for dirty water) OR Janitor trolley	PHC-E-030
Colour labelled mop – Red for toilets and bathrooms	PHC-E-015
Colour labelled mop – Blue for clinical areas and non-clinical service areas	PHC-E-016
Mop labelled for cleaning exterior areas	PHC-E-017
Green bucket and cloths for bathroom and consulting room basins	PHC-E-009, PHC-C-187
Red bucket and cloths for toilet	PHC-E-007
White cloths for kitchen	PHC-C-189
Blue bucket and cloths for clinical areas and non-clinical service areas	PHC-E-008, PHC-C-188
Spray bottle for disinfectant solution	PHC-E-022
Window cleaning squeegee	PHC-E-150
Mop sweeper or soft-platform broom	PHC-E-018
Floor polisher	PHC-F-020

CHECKLIST FOR ELEMENT: FUNCTIONAL FIREFIGHTING EQUIPMENT IS AVAILABLE

Use the checklist below to check whether firefighting equipment is available

Item	
Fire extinguishers	PHC-E-043
Fire hoses and reels unless it is a single-storey building of less than 250 m^2 in floor area OR the facility has no water supply	PHC-E-044
Two 9 kg or equivalent fire extinguishers where the facility has no water supply	PHC-E-043
Firefighting equipment is maintained according to schedule	

CHECKLIST FOR ELEMENT: FURNITURE IS AVAILABLE AND INTACT IN SERVICE AREAS

Use the checklist below to check whether facility service areas are equipped with sufficient functional furniture

Item	
**Waiting areas**	
Seating	
a. Adequate seating for all patients	PHC-F-015,016,017
b. Chairs / benches intact	
Notice boards available	PHC-E-102,104
**Consulting rooms**	
Desk	
a. Available	PHC-F-007
b. Intact (including the drawers)	
Chair (clinician)	
a. Available	PHC-F-002
b. Intact	
At least 1 chair (patient)	
a. Available	PHC-F-005
b. Intact	
Tilting examination couch	
a. Available	PHC-F-030
b. Intact	
Bedside footstool	
a. Available	PHC-F-031
b. Intact	
Wall-mounted or portable anglepoise-style examination lamp	
a. Available	PHC-E-110, 154
b. Intact	
Lockable medicine cupboards	
Available	PHC-F-018
Intact	
Dressing trolley (at bedside for examination equipment)	
Available	PHC-F-036
Intact (including the drawers)	

CHECKLIST FOR ELEMENT: ESSENTIAL EQUIPMENT IS AVAILABLE AND FUNCTIONAL IN CONSULTING AREAS

Use the checklist below to check whether essential equipment is available and functional in consultation/vital signs and child health rooms

Item	
**CONSULTATION ROOMS**	
Stethoscope	PHC-E-126
Non-invasive Baumanometer (wall mounted/ portable)	PHC-E-115,116
Adult, paediatric and large cuffs (3) for Baumanometer	PHC-E-115,116
Diagnostic sets including ophthalmic pieces (wall mounted or portable)	PHC-E-117,118,119
Patella hammer	PHC-E-120
Tuning fork (only required in one consultation room)	PHC-E-132
Tape measure	PHC-E-113
Clinical thermometers	PHC-E-130,131
**VITAL SIGNS ROOM**<br>**(Note if facility is too small to have a vital signs room, check for equipment in consultation rooms)**	
Non-invasive electronic Baumanometer (wall mounted/ portable)	PHC-E-116
Adult, paediatric and large cuffs (3) for Baumanometer	PHC-E-116
Blood glucometer	PHC-E-149
Peak flow meter	PHC-E-267
Adult clinical scale up to 150 kg	PHC-E-122,123
Stethoscope	PHC-E-126
HB meter	PHC-E-152
Clinical thermometer	PHC-E-130,131
Height measure	PHC-E-125
Tape measure	PHC-E-113
Bin (general waste)	PHC-E-003
Urine specimen jars	PHC-E-150,158

CHILD HEALTH ROOM	
Baby scale	PHC-E-124
Bassinet	PHC-E-025
Stethoscope	PHC-E-126
Blood glucometer	PHC-E-149
Non-invasive Baumanometer (wall mounted/ portable)	PHC-E-115,116
Paediatric cuff for Baumanometer	PHC-E-115,116
Diagnostic sets including ophthalmic pieces(wall mounted or portable)	PHC-E-117,118,119
Patella hammer	PHC-E-120
Tape measure	PHC-E-113
Clinical thermometers	PHC-E-130,131

CHECKLIST FOR ELEMENT: RESUSCITATION ROOM IS EQUIPPED WITH FUNCTIONAL, BASIC RESUSCITATION EQUIPMENT

Use the checklist below to check whether the emergency/resuscitation room complies with measures for functional basic equipment

Item	
Emergency trolley with lockable medicine drawer and accessories	PHC-F-040
Examination couch/2-part obstetric delivery bed	PHC-F-029,030
Wall or ceiling mounted angle-poise-style examination lamp	PHC-E-110,111
Nebuliser OR face mask with nebuliser chamber for adult and paediatric	PHC-E-137
Functional electric powered OR manual suction devices and suction catheters	PHC-E-144, 146
Drip stand	PHC-F-028
Dressing trolley	PHC-F-037, 038, 039
Cardiac arrest board	PHC-E-134
Bin (general waste)	PHC-E-003
Suture material	PHC-C-180, 181, 182
Thermal (space) blanket	PHC-C-179
Gloves exam n/sterile gloves: small, medium and large at least one pair of each size	PHC-C-141, 142, 143
Gloves surgical sterile latex: 6 OR 6.5, 7 OR 7.5 and 8, at least one pair of each size	PHC-C-138, 139, 140
Protective face shields OR Goggles with face mask	PHC-C-080, 079
Disposable plastic aprons	PHC-C-137
Disposable non-sterile face masks	PHC-C-077
Resuscitation algorithms	
Resuscitation documentation register	
Wall-mounted liquid hand soap dispenser	PHC-E-014
Wall-mounted hand paper dispenser	PHC-E-020, 021

CHECKLIST FOR ELEMENT: EMERGENCY TROLLEY IS RESTORED DAILY OR AFTER EACH USE

Use the checklist below to check whether the emergency trolley is sufficiently stocked with unex

Item	
Laryngoscope handle with functional batteries	PHC-E-135
Adult curved blades for laryngoscope size 2	PHC-E-135
Adult curved blades for laryngoscope size 3	PHC-E-135
Adult curved blades for laryngoscope size 4	PHC-E-135
Paediatric straight blades for laryngoscope size 1	PHC-E-135
Spare bulbs for laryngoscope	PHC-E-135
Spare batteries for laryngoscope sizes	
Endotracheal tubes – uncuffed size 2mm OR 2.5mm	PHC-C-086,354
Endotracheal tubes – uncuffed size 3mm OR 3.5mm	PHC-C-087,355
Endotracheal tubes – uncuffed size 4.0mm OR 4.5mm	PHC-C-088,356
Endotracheal tubes – cuffed size 5.0mm	PHC-C-089
Endotracheal tubes – cuffed size 6.0mm	PHC-C-090
Endotracheal tubes – cuffed size 7.0mm	PHC-C-091
Endotracheal tubes – cuffed size 8.0mm	PHC-C-092
Water-soluble lubricant/lubricating jelly	PHC-C-172
Tape to hold tie endotracheal tube in place	
Patella hammer	PHC-E-120
Oropharyngeal airways (Guedel) size 0	PHC-C-113
Oropharyngeal airways (Guedel) size 1	PHC-C-114
Oropharyngeal airways (Guedel) size 2	PHC-C-115
Oropharyngeal airways (Guedel) size 3	PHC-C-116
Oropharyngeal airways (Guedel) size 4	PHC-C-117
Adult-size introducer, intubating stylet or bougie for endotracheal tubes	PHC-I-125

Paediatricsize introducer, intubating stylet or bougie for endotracheal tubes	PHC-I-126
Magill's forceps for adults	PHC-I-123
Magill's forceps for paediatric	PHC-I-124
Laryngeal masks (supraglottic airways): adult	PHC-C-103, 104
Manual bag valve mask/ manual resuscitator OR self-inflating bag with compatiblemasks for adults	PHC-E-142,143
Manual bag valve mask/ manual resuscitator OR self-inflating bag with compatible masks for paediatric	PHC-E-140,141
Simple face mask OR reservoir mask OR nasal cannula (prongs) for oxygen, adults	PHC-E-111,108,106
Simple face mask OR reservoir mask OR nasal cannula (prongs) for oxygen, paediatric	PHC-E-110,107,105
Face mask for nebuliser OR face mask with nebuliser chamber for adult OR spacers for MDIs (adults and paediatrics)	PHC-E-108
Face mask for nebuliser OR face mask with nebuliser chamber for paediatric OR spacers for MDIs (adults and paediatrics)	PHC-E-107
Automatic External Defibrillator (AED) OR ECG monitor and defibrillator	PHC-E-133
Intravenous cannula 18g green and appropriate strapping	PHC-C-101,097
Intravenous cannula 20g pink and appropriate strapping	PHC-C-101,098
Intravenous cannula 22g blue and appropriate strapping	PHC-C-101,099
Intravenous cannula 24g yellow and appropriate strapping	PHC-C-101,100
Syringes 3-part: 2ml	PHC-C-118
Syringes 3-part: 5ml	PHC-C-119
Syringes 3-part: 10ml OR 20ml	PHC-C-120,349
Syringes: insulin syringes	PHC-C-121
Needles: 18 (pink) OR 20 (yellow)	PHC-C-122,123
Needles: 21 (green)	PHC-C-124
Needles: 23 (blue) OR 22 (black)	PHC-C-125,126
Sharps container	PHC-E-005
Admin set 20 drops/ml 1.8m /pack	PHC-C-095
Admin set paeds 60 drops/ml 1.8m /pack	PHC-C-096
Stethoscope	PHC-E-126

Haemoglobin meter	PHC-E-152
Blood glucometer with testing strips and spare batteries	PHC-E-149
Diagnostic set and batteries including opthalmic pieces (wall mounted or portable)	PHC-E-117,118,119
Rescue scissors (to cut clothing)	PHC-I-020
Paediatric Broselow tape OR Pawper tape	PHC-E-128, 129
Wound care (gauze, bandages, cotton wools, plasters, alcohol swabs and antiseptic solutions)	PHC-C-147 onwards
Urinary (Foley's) catheters: 14f	PHC-C-127
Urinary (Foley's) catheters: 18f	PHC-C-128
Urinary bag specified in the surgical supply list	PHC-C-129
Nasogastric tubes: 600mmfg8	PHC-C-130
Nasogastric tubes: 1000mmfg10 or 12	PHC-C-131, 132
Medication/vaculitre stickers	
**Present individually or in combined multifunctional diagnostic monitoring set**	
Pulse oximeter with adult & paediatric probes	PHC-E-139
Non invasive electronic blood pressure monitoring device including paediatric, adult & large adult cuff sizes	PHC-E-136, 116
Clinical thermometer (in 0C, non-mercury)	PHC-E-130, 131
**Emergency medicines (also check expiry dates)**	
Activated Charcoal	
Adrenaline injection 1mg/ml (Epinephrine)	
Amlodipine 5mg OR 10mg tablets	
Aspirin tablets	
Atropine 0.5mg OR 1mg injection	
Benzodiazepine e.g. diazepam injection 5mg/ml injection	
Calcium gluconate 10% injection	
Furosemide 20mg ampoule	
Hydrocortisone 100mg/ml injection	
Insulin, short acting (stored in the medicine fridge)	
Ipratropium 0.25mg/2ml OR 0.5mg/2ml unit dose vial for nebulisation OR 40 mcg MDIs	

Isosorbide dinitrate, sublingual, 5mg tablets
Lidocaine/Lignocaine IV 1% OR 2%
Magnesium sulphate 50%, 1g/2ml ampoule (minimum of 14 ampoules required for one treatment course)
Midazolam (1mg/ml OR 5mg/ml) OR Diazepam 5mg/ml
Nifedipine 10mg capsules
Prednisone 5 mg tablets
Promethazine 25mg ampoule
Short-acting sublingual nitrates e.g. glyceryl trinitrate SL OR isosorbide dinitrate
Salbutamol nebulising solution OR nebules OR MDI
Thiamine 100mg injection
Water for injection
**IV Solutions**
50% dextrose (20ml ampoule or 50ml bag) OR 10% dextrose solution
Pediatric solutions e.g. ½ strength Darrows solution AND neonatalyte solution
Sodium Chloride 0.9%

CHECKLIST FOR ELEMENT: THERE IS AN EMERGENCY STERILE OBSTETRIC DELIVERY PACK

Use the checklist below to check whether there is sterile emergency packs available.

**Item**	Quantity	
**NON-NEGOTIABLE**		
Stitch scissor	1	PHC-I-009
Episiotomy scissor	1	PHC-I-019
Cord scissor	1	PHC-I-018
Dissecting forceps non-toothed (plain)	1	PHC-I-002
Dissecting forceps toothed	1	PHC-I-001
Artery forceps, straight, long	2	PHC-I-003
Needle holder	1	PHC-I-008
Small bowl	2	PHC-I-012
Kidney dishes OR receivers (big)	2	PHC-I-013
**EXTRAS (not part of sterilised pack)**		
Basin	1	PHC-I-021
Stainless-steel round bowl, large	1	PHC-I-022
Green towels	4	
Disposable apron	2	PHC-C-137
Gauzes	5	PHC-C-150
Vaginal tampons	1	PHC-C-158
Sanitary towels	2	PHC-C-156
Round cotton wool balls	1 pack	PHC-C-148
Umbilical cord clamps	2	PHC-C-163

CHECKLIST FOR ELEMENT: THERE IS A STERILE PACK FOR MINOR SURGERY

Use the checklist below to check whether equipment for minor surgery is available

Item	Quantity	
**MINOR STITCH / SUTURING TRAY**		
Small stitch tray	1	PHC-I-010
Stitch scissor	1	PHC-I-009
Toothed forceps	1	PHC-I-001
Non-toothed forceps	1	PHC-I-002
Bard-Parker surgical blade handle to fit accompanying blades (blades do not form part of sterilised pack but must be available)	1	PHC-I-007
Mosquito, straight	2	PHC-I-005
Mosquito, curved	2	PHC-I-006
Artery forceps, straight	2	PHC-I-003
Artery forceps, curved	2	PHC-I-004
Needle holder	1	PHC-I-008
Swab holder	1	PHC-I-011

CHECKLIST FOR ELEMENT: COMPLAINTS/COMPLIMENTS /SUGGESTIONS TOOLKIT IS AVAILABLE AT THE MAIN ENTRANCE/EXIT

Use the checklist below to check whether the complaint forms, box and poster is available at the main entrance

Item
Complaints/compliments/suggestions boxes are visibly placed at main entrance/exit
Official complaint/compliment/suggestion forms and pen are at the box at the main entrance/exit
A standardised poster describing the process to follow to lodge a complaint, give a compliment or make a suggestion is visibly displayed at the entrance of the facility in at least two local languages

PHC-E-100

CHECKLIST FOR ELEMENT: SHARPS ARE DISPOSED OF APPROPRIATELY

Use the checklist below to check whether sharps are disposed of appropriately

Item
Waste is properly segregated
Sharps are disposed of in impenetrable, tamperproof containers
Sharps containers are disposed of when they reach the limit mark
Sharps containers are placed on work surface or in wall mounted brackets

PHC-E-005

CHECKLIST FOR ELEMENT: TEMPERATURE OF THE MEDICINE ROOM/DISPENSARY IS MAINTAINED WITHIN THE SAFETY RANGE

Use the checklist below to check whether the medicine in the medicine room/dispensary is maintained within the safety range

Item	
There is at least one functional, wall-mounted room thermometer	PHC-E-114
The temperature of the pharmacy is recorded daily	PHC-C-134
The temperature of the pharmacy is maintained within the safety range	

CHECKLIST FOR ELEMENT: COLD CHAIN PROCEDURE FOR VACCINES IS MAINTAINED

Use the checklist below to check whether the cold chain for vaccines is maintained

Item	
There is a standard operating procedure for the maintenance of cold chain for vaccines	
Facility has a vaccine or medicine refrigerator with a thermometer	PHC-F-042, 043, 049, 050, PHC-E-153, 042
The temperature of the refrigerator is recorded twice daily, 7 hours apart (check one month's record)	PHC-C-135
The temperature of the refrigerator is maintained between 2-8 ºC (check one month's record)	
There is a cooler box for storage of vaccines if needed	PHC-E-035
Ice packs are available for use as needed	PHC-C-203

CHECKLIST FOR ELEMENT: SPECIMENS ARE COLLECTED, PACKED, STORED AND PREPARED FOR TRANSPORTATION ACCORDING TO THE PRIMARY HEALTH CARE LABORATORY HANDBOOK

Use the checklist below to check whether specimens are handled according to the PHC Laboratory Handbook

Grouping of specimens

Group A	Group B	Group C
Blood<br>Pleural effusion<br>Sputum<br>Stool<br>Urine	Pap smear	MCS (Microscopy, culture band sensitivity)

Item	
**General**	
Specimens are clearly labelled	
Each laboratory request form is correctly completed	
There is at least one functional wall mounted thermometer in area for lab specimens are stored for courier collection	PHC-E-114
The temperature of the storage area for lab specimens is recorded daily	PHC-C-134
**Group A specimens**	
Samples are kept away from direct sunlight	
Where the room temperature exceeds 25°C, samples are stored in the fridge (at +- 5°C)	PHC-F-022
Length of storage does not exceed 24 hours, stored at room temperature (+- 20-25°C)	
**Group B specimens**	
Stored at room temperature	
Stored inside a slide carrier (envelope)	
**Group C specimens**	
Samples placed into the transport medium provided (where appropriate)	
Samples kept away from direct sunlight	
Where room temperature exceeds 25°C, samples are stored in the fridge (+- 5°C)	PHC-F-022
Length of storage does not exceed 24 hours, stored at room temperature (+-20-25°C)	

CHECKLIST FOR ELEMENT: HEALTH CARE WASTE ARE MANAGED APPROPRIATELY

Use the checklist below to check whether health risk care waste is managed appropriately

Item	
Sanitary disposal bins with functional lids OR health care risk waste box	PHC-E-004
* Sanitary disposal bins/boxes lined with appropriate colour plastic bags	PHCC196
Sanitary disposal bins/boxes are clean and not overflowing	
Health care risk waste disposal bins with functional lids OR health care risk waste box	PHC-E-047
Health care risk waste disposal bins/boxes lined with red colour plastic bags	PHC-C-195
Health care risk waste disposal bins/boxes contain only health care waste	
Health care risk waste disposal bins/boxes are not overflowing	
Bins available for general waste	PHC-E-046, 049
Bins for general waste are lined with appropriate coloured bags	PHC-C-190 onwards

* If disposable boxes for sanitary waste is used where gel granules in the bottom of the box treat the waste, no bag is required and facility can score "Y"

CHECKLIST FOR ELEMENT: THERE IS A STANDARD SECURITY GUARD ROOM OR THE FACILITY HAS AN ALARM SYSTEM LINKED TO ARMED RESPONSE

Use the checklist below to check whether facility security adheres to standard guidelines

Item	
Does the facility have an alarm system linked to armed response (if Yes, checklist for security guardroom and security equipment must not be assessed. If No, assess checklist for security guardroom and security equipment)	
**Security guard room**	
Kitchenette – sink with cupboard underneath	
Table	PHC-F-008
Chair	PHC-F-003
Functioning lights	
**Security equipment for security officer(s) and accompanying stationery**	
Baton	
Handcuffs OR Cable ties	
Incident book	
Metal detector	
Telephone OR two-way radio OR dedicated cellphone	

CHECKLIST FOR ELEMENT: ALL STAFF MEMBERS WEAR AN IDENTIFICATION TAG

Use the checklist below to check that the staff on duty wear official identification tags

Staff member	
Staff member 1	PHC-C-202
Staff member 2	PHC-C-202
Staff member 3	PHC-C-202
Staff member 4	PHC-C-202
Staff member 5	PHC-C-202

Note: Identification tag must include the emblem of the facility/district or provincial department of health, full names/ initials and surname of the staff member

CHECKLIST FOR ELEMENT: HAND HYGIENE AND SANITARY FACILITIES ARE AVAILABLE

Use the checklist below to check whether there is running water, toilet paper, liquid hand wash soap and disposable hand paper towels

**Item**	
**Toilet**	
Functional hand wash basin with taps	
Running water	
Toilet paper	PHC-C-081
Liquid hand wash soap/sanitiser	PHC-C-058
Disposable hand paper towels	PHC-C-072, 073
**Consultation room**	
Functional hand wash basin with taps	
Running water	
Liquid hand wash soap/sanitiser	PHC-C-058
Disposable hand paper towels	PHC-C-072, 073
**Vital signs room**	
Functional hand wash basin with taps	
Running water	
Liquid hand wash soap/sanitiser	PHC-C-058
Disposable hand paper towels	PHC-C-072, 073

Ideal Community Health Centre Checklists

CHECKLIST FOR ELEMENT: STAFF WEAR APPROPRIATE PERSONAL PROTECTIVE CLOTHING AT THE 8 HOUR SERVICE AREA

Use the checklist below to check whether protective clothing is available and worn

Item	
Gloves – non sterile	PHC-C-141, 142, 143
Gloves – sterile	PHC-C-138, 139, 140
Disposable gowns OR aprons	PHC-C-076, 137
Protective face shields OR goggles with surgical face masks	PHC-C-080, 079, 077

CHECKLIST FOR ELEMENT: STAFF WEAR APPROPRIATE PERSONAL PROTECTIVE CLOTHING AT THE 24 HOUR EMERGENCY UNIT

Use the checklist below to check whether protective clothing is available and worn

Item	
Gloves – non sterile	PHC-C-141, 142, 143
Gloves – sterile	PHC-C-138, 139, 140
Disposable gowns OR aprons	PHC-C-076, 137
Protective face shields OR goggles with surgical face masks	PHC-C-080, 079, 077

CHECKLIST FOR ELEMENT: STAFF WEAR APPROPRIATE PERSONAL PROTECTIVE CLOTHING AT THE MOU

Use the checklist below to check whether protective clothing is available and worn

Item	
Gloves – non sterile	PHC-C-141, 142, 143
Gloves – sterile	PHC-C-138, 139, 140
Disposable gowns OR aprons	PHC-C-076, 137
Protective face shields OR goggles with surgical face masks	PHC-C-080, 079, 077

CHECKLIST FOR ELEMENT: COMPLAINTS/COMPLIMENTS/SUGGESTIONS TOOLKIT IS AVAILABLE AT THE MAIN ENTRANCE/EXIT AT THE 8 HOUR SERVICE AREA

Use the checklist below to check whether the complaint forms, box and poster is available

Item
Complaints/compliments/suggestions boxes are visibly placed at main entrance/exit
Official complaint/compliment/suggestion forms and pen are at the box at the main entrance/exit
A standardised poster describing the process to follow to lodge a complaint, give a compliment or make a suggestion is visibly displayed at the entrance of the facility in at least two local languages

PHC-E-100

CHECKLIST FOR ELEMENT: COMPLAINTS/COMPLIMENTS/ SUGGESTIONS TOOLKIT IS AVAILABLE AT THE 24 HOUR EMERGENCY UNIT

Use the checklist below to check whether the complaint forms, box and poster is available

Item	
Complaints/compliments/suggestions boxes are visibly placed at main entrance/exit	PHC-E-100
Official complaint/compliment/suggestion forms and pen are at the box at the main entrance/exit	
A standardised poster describing the process to follow to lodge a complaint, give a compliment or make a suggestion is visibly displayed at the entrance of the facility in at least two local languages	

CHECKLIST FOR ELEMENT: COMPLAINTS/COMPLIMENTS/ SUGGESTIONS TOOLKIT IS AVAILABLE AT THE MAIN ENTRANCE/EXIT AT THE MOU

Use the checklist below to check whether the complaint forms, box and poster is available

Item
Complaints/compliments/suggestions boxes are visibly placed at main entrance/exit
Official complaint/compliment/suggestion forms and pen are at the box at the main entrance/exit
A standardised poster describing the process to follow to lodge a complaint, give a compliment or make a suggestion is visibly displayed at the entrance of the facility in at least two local languages

PHC-E-100

CHECKLIST FOR ELEMENT: BASIC MEDICAL SUPPLIES (CONSUMABLES) ARE AVAILABLE

Use the checklist below to check the availability of medical and dressing supplies

SURGICAL SUPPLIES	
**Item**	
Intravenous administration set 20 drops/ml	PHC-C-095
Intravenous admin set paeds 60 drops/ml	PHC-C-096
Blade stitch cutter sterile/pack	PHC-C-145
Blood collecting vacutainer (holding barrel/bulldog)	
Blood lancets (haemolance)	PHC-C-146
Urinary (Foley's) catheter silicone/latex 8f	PHC-C-343
Urinary (Foley's) catheter silicone/latex 10f	PHC-C-344
Urinary (Foley's) catheter silicone/latex 12f	PHC-C-345
Urinary (Foley's) catheter silicone/latex 14f	PHC-C-127
Urinary (Foley's) catheter silicone/latex 16f	PHC-C-346
Urinary (Foley's) catheter silicone/latex 18f	PHC-C-128
Urinary (Foley's) catheter silicone/latex 20f	PHC-C-347
Urinary (Foley's) catheter silicone/latex 22f	PHC-C-348
Catheter suction resp 500mm 06f	PHC-C-359
Catheter suction resp 500mm 08f	PHC-C-360
Catheter suction resp 500mm 10f	PHC-C-361
Catheter suction resp 500mm 12f	PHC-C-362
Catheter suction resp 500mm 14f	PHC-C-363
Catheter thoracic silicone st20	PHC-C-364
Catheter thoracic silicone st24	PHC-C-365
Catheter thoracic silicone st28	PHC-C-366
Catheter thoracic silicone st30	PHC-C-367

Catheter thoracic silicone st32	PHC-C-368
Drainage sys chest u/water adult	PHC-C-369
Urine drainage bag	PHC-C-129
Simple face mask OR reservoir mask OR nasal cannula (prongs) for oxygen, adults	PHC-C-108,106,111
Simple face mask OR reservoir mask OR nasal cannula (prongs) for oxygen, paediatric	PHC-C-110,105,107
Face mask for nebuliser OR face mask with nebuliser chamber for adult	PHC-C-107
Face mask for nebuliser OR face mask with nebuliser chamber for paediatric	PHC-C-108
Nasogastric feeding tube 600mm fg5	PHC-C-342
Nasogastric feeding tube 600mm fg8	PHC-C-130
Nasogastric feeding tube 1000mm fg10 OR 12	PHC-C-131,132
Disposable aprons	PHC-C-137
Eye patches (disposable)	PHC-C-165
Disposable razors	PHC-C-166
Disposable Amnihook	PHC-C-171
Ultrasound gel medium viscosity	PHC-C-173
Gloves exam n/sterile large /box	PHC-C-143
Gloves exam n/sterile medium /box	PHC-C-142
Gloves exam n/sterile small /box	PHC-C-141
Gloves surg sterile latex sz 6 OR6.5 OR Small/box	PHC-C-138
Gloves surg sterile latex sz 7OR 7.5 OR medium/box	PHC-C-139
Gloves surg sterile latex sz 8 OR large/box	PHC-C-140
Endotracheal tubes – uncuffed size 2mm OR 2.5mm	PHC-C-086,354
Endotracheal tubes – uncuffed size 3mm OR 3.5mm	PHC-C-087,355
Endotracheal tubes – uncuffed size 4.0mm OR 4.5mm	PHC-C-088,356
Endotracheal tubes – cuffed size 5.0mm	PHC-C-089
Endotracheal tubes – cuffed size 6.0mm	PHC-C-090
Tube stomach washout 24fg	PHC-C-369

Tube,stomach washout 26fg	PHC-C-370
Tube,stomach washout 28fg	PHC-C-371
Tube stomach washout 30fg	PHC-C-372
Sheath incontinence 25mm	PHC-C-373
Sheath incontinence 30mm	PHC-C-374
Sheath incontinence 35mm	PHC-C-375
Intravenous cannula(Jelco)18g green/box	PHC-C-097
Intravenous cannula(Jelco)20g pink/box	PHC-C-098
Intravenous cannula (Jelco)22g blue/box	PHC-C-099
Intravenous cannula (Jelco)24g yellow/box	PHC-C-100
Needles: 18 (pink) OR 20 (yellow)/box	PHC-C-122,123
Needles: 21 (green)/box	PHC-C-124
Needles: 22 (black)/box	PHC-C-126
Needles: 23 (blue)/box	PHC-C-125
Needles: 25 /box	PHC-C-386
* Syringes 3-part 2ml/box	PHC-C-118
* Syringes 3-part 5ml/box	PHC-C-119
* Syringes 3-part 10 ml/box	PHC-C-120
* Syringes 3-part 20ml/box	PHC-C-349
Insulin syringe with needle /box	PHC-C-121
Dental syringe and needle for LA	PHC-C-180
Suture chromic g0/0 or g1/0 1/2 75cm	PHC-C-181
Suture nylon g2/0 or g3/0 3/8 45cm	PHC-C-182
Suture nylon g4/0 3/8 45cm	PHC-C-098

Only applicable if the facility uses older HB model		
Haemolysis applicator sticks		PHC-C-167
HB meter clip		PHC-C-168
HB chamber glass-grooved		PHC-C-169
HB cover glass-plain		PHC-C-170
**Only applicable if facility uses an Automatic External Defibrillator (AED)**		
Replacement pads for AED – adult		PHC-C-108
Replacement pads for AED - paediatric		PHC-C-175
**DRESSINGS SUPPLIES**		
**Item**	**Pack size**	
Plaster roll	1	PHC-C-153
Bandage crepe	1	PHC-C-147
Gauze paraffin 100x100 /box	1	PHC-C-152
Gauze swabs plain n/s 100x100x8ply/pack	100	PHC-C-151
Gauze abs grade 1 burn /pack	1	PHC-C-150
Basic disposable dressing pack(should contain as a minimum cotton wool balls, swabs, 2 forceps, disposable drape)	1	PHC-C-149
Cotton wool balls 1g 500`s		PHC-C-148
* Skin traction kit - adult (elast 0468)		PHC-C-376
* Skin traction kit - child (elast 0469)		PHC-C-377
* Sodium carboxymethylcel (intrasite) 15g		
Sanitary towels maternity /pack	12	PHC-C-156
Stockinette 100mm OR150mm/roll	1	PHC-C-154, 186
Adhesive micro-porous surgical tape 24/25mm or 48/50mm	1	PHC-C-159, 185
70% isopropyl alcohol prep Pads 24x30 1ply OR 2 ply /box	200	PHC-C-155

CHECKLIST FOR ELEMENT: BASIC CONSUMABLES ARE AVAILABLE FOR THE REHABILITATION TREATMENT AREA

Use the checklist below to check the availability of consumable for the rehabilitation treatment area

Item	
Batteries: hearing aid	PHC-C-265 - 268
Bicarbonate of soda sachets	PHC-C-269
Breadboard - one-handed	PHC-C-270
Cable ties, nylon, medium	PHC-C-271
Cable ties, nylon, long	PHC-C-272
Cardboard A4 coloured	PHC-C-273
Coloured writing instruments	PHC-C-274
Contact adhesive	PHC-C-217
Cerumen curette: replacement curettes	PHC-C-275
Cerumen curette: replacement globe	PHC-C-276
Cushion: wheelchair, thin, positioned	PHC-C-277
Cushion: wheelchair, thick, pressure care	PHC-C-278
Cushion cover: basic stretchable	PHC-C-279
Cushion cover: waterproof	PHC-C-280
Ear mould impression material and scoop	PHC-C-281
Ear mould impression material catalyst	PHC-C-282
Ear mould impression Otolight: replacement globe	PHC-C-283
Ear mould impression Otolight: replacement tips	PHC-C-284
Ear mould impression Otostops 3 sizes	PHC-C-285 - 287
Exercise band latex	PHC-C-288 - 292
Feeding cup	PHC-C-293
Feeding spoon	PHC-C-294
Ferrule: rubber 4 sizes	PHC-C-295 - 297

EVA foam	PHC-C-298
Foam HD compressed	PHC-C-301 - 302
Foam high-density	PHC-C-299 - 300
Masonite	PHC-C-303
Oil: arnica	PHC-C-304
Paper: print-out, tympanometer	PHC-C-305
Pelvic strap 5cm	PHC-C-335
Pelvic strap 3cm	PHC-C-336
Pressure garment material (Elastonet)	PHC-C-306 - 308
Sewing kit	PHC-C-309
Splinting material 6 sizes	PHC-C-310 - 315
Splinting material: thermoplastic tape	PHC-C-316 - 317
Superglue (cyanoacrylate)	PHC-C-041
Toothbrush, large	PHC-C-258
Towelling	PHC-C-318 - 320
Varnish (wood)	PHC-C-321
Velcro circles (hook and loop)	PHC-C-322 - 323
Velcro hook tape 2 sizes	PHC-C-325
Velcro loop tape 2 sizes	PHC-C-324
Washboard one-handed	PHC-C-326
Waterproof wood glue	PHC-C-042
Wheelchair gloves	PHC-C-337
Wheelchair spares kit	PHC-C-327

CHECKLIST FOR ELEMENT: BASIC MEDICAL SUPPLIES (CONSUMABLES) ARE AVAILABLE FOR THE ORAL HEALTH SERVICES

Use the checklist below to check the availability of consumable for the Oral Health Services

SURGICAL SUPPLIES	
**Item**	
**Exodontia (tooth extraction) and minor oral surgery**	
Apron, dental, plastic Adult	PHC-C-224
Apron, dental, plastic child	PHC-C-225
Surgical blades No.11 and No. 12	PHC-C-333, 334
Chlorhexidine oral rinse 0,2%	
Cotton wool balls	PHC-C-148
Dry socket alveolar paste	PHC-C-235
Ethyl chloride	
Haemostat sponge	PHC-C-249
Hydrogen peroxide	
Hypodermic needles (disposable)	PHC-C-176, 177
Saline solution	
Saliva ejectors (disposable)	PHC-C-256, 257
Sutures surgical	PHC-C-330, 331, 332
Topical anaesthetic	PHC-C-223
Local anaesthetic (without vasoconstrictor)	
**Conservative (preventive) dentistry**	
Fissure sealants	PHC-C-236
Amalgam capsules	PHC-C-222
Composite	PHC-C-231, 232
Fluoride gel	PHC-C-245, 246, 247
Varnish cavity liner	PHC-C-264
Prophylaxis paste	PHC-C-250

Cements /liners (kalzinol, Dycal etc.)	PHC-C-230
Articulating paper	PHC-C-226
Cotton wool pellets	PHC-C-254, 255
Polyester strips (composite)	PHC-C-242, 243, 244
Glass ionomers	PHC-C-248
Polishing strips	PHC-C-252, 253
Polishing kit	PHC-C-251
Dental floss	PHC-C-234
Fluoride trays	PHC-C-242, 243, 244
Acid etch and bonding agent	PHC-C-228
Dentin bonding agents	PHC-C-252, 253
**School outreach**	
Toothpaste, dental, fluoride	PHC-C-261, 262, 263
Toothbrushes	PHC-C-258, 259, 260

CHECKLIST FOR ELEMENT: REQUIRED FUNCTIONAL DIAGNOSTIC EQUIPMENT AND CONCURRENT CONSUMABLES FOR POINT OF CARE TESTING ARE AVAILABLE

Use the checklist below to check the availability of laboratory equipment and consumables in the various areas where they are used

Item	
**Laboratory equipment and consumables**	
Hb meter	PHC-E-152
Blood glucometer	PHC-E-149
Spare batteries for blood glucometer	
Glass slides for cervical smears	
Lancets	PHC-C-146
Blood glucose strips	PHC-C-181
Urine dipsticks	PHC-C-205
Urine specimen jar OR flask	PHC-E-151, 158
Malaria rapid test (where applicable in facilities in KZN, GP, MP and LP)	PHC-C-200
Rapid HIV test	PHC-C-199
Rh ‘D’ (Rhesus factor) test	PHC-C-204

CHECKLIST FOR ELEMENT: DISINFECTANT, CLEANING MATERIALS AND EQUIPMENT ARE AVAILABLE

Use the checklist below to check whether the disinfectant, cleaning materials and equipment are available

Disinfectant and cleaning Material	
High-level disinfection for medical equipment (e.g. sodium perborate powder OR phthalaldehyde)	PHC-C-063, 034, 035
Chlorine compounds (e.g. Biocide D or Clorox)	PHC-C-062
Sanitary all-purpose cleaner	PHC-C-136
Detergent-based solutions	PHC-C-065
Wet polymer (floor polish)	PHC-C-066
Protective polymer (strippers)	PHC-C-067
All cleaning materials clearly labelled	
Materials Safety Data Sheets for all cleaning products	
**Cleaning equipment**	
Two-way bucket system for mopping floors (bucket for clean water and bucket for dirty water) OR Janitor trolley	PHC-E-030
Colour labelled mop – Red for toilets and bathrooms	PHC-E-015
Colour labelled mop – Blue for clinical areas and non-clinical service areas	PHC-E-016
Mop labelled for cleaning exterior areas	PHC-E-017
Green bucket and cloths for bathroom and consulting room basins	PHC-E-009, PHC-C-187
Red bucket and cloths for toilet	PHC-E-007
White cloths for kitchen	PHC-C-189
Blue bucket and cloths for clinical areas and non-clinical service areas	PHC-E-008, PHC-C-188
Spray bottle for disinfectant solution	PHC-E-022
Window cleaning squeegee	PHC-E-150
Mop sweeper or soft-platform broom	PHC-E-018
Floor polisher	PHC-F-020

CHECKLIST FOR ELEMENT: FUNCTIONAL FIREFIGHTING EQUIPMENT IS AVAILABLE

Use the checklist below to check whether firefighting equipment is available

Item	
Fire extinguishers	PHC-E-043
Fire hoses and reels unless it is a single-storey building of less than 250 m^2 in floor area OR the facility has no water supply	PHC-E-044
Two 9 kg or equivalent fire extinguishers where the facility has no water supply	PHC-E-043
Firefighting equipment is maintained according to schedule	

CHECKLIST FOR ELEMENT: FURNITURE IS AVAILABLE AND INTACT AT 8 HOUR SERVICE AREAS

Use the checklist below to check whether facility service areas are equipped with sufficient functional furniture in the 8 hour service area

**Item**	
**Waiting areas**	
Seating	
a. Adequate seating for all patients	PHC-F-015,016,017
b. Chairs / benches intact	
Notice boards available	PHC-E-102,104
**Consulting rooms**	
Desk	
a. Available	PHC-F-007
b. Intact (including the drawers)	
Chair (clinician)	
a. Available	PHC-F-002
b. Intact	
At least 1 chair (patient)	
a. Available	PHC-F-005
b. Intact	
Tilting examination couch	
a. Available	PHC-F-030
b. Intact	
Bedside footstool	
a. Available	PHC-F-031
b. Intact	
Lockable medicine cupboards	
a. Available	PHC-F-018
b. Intact	
Dressing trolley (at bedside for examination equipment)	
a. Available	PHC-F-036
b. Intact (including the drawers)	

CHECKLIST FOR ELEMENT: ESSENTIAL EQUIPMENT IS AVAILABLE AND FUNCTIONAL AT THE 8 HOUR SERVICES

Use the checklist below to check whether essential equipment is available and functional in consultation/vital signs and child health rooms

Item	
**CONSULTATION ROOMS**	
Stethoscope	PHC-E-126
Non-invasive Baumanometer (wall mounted/ portable)	PHC-E-115,116
Adult, paediatric and large cuffs (3) for Baumanometer	PHC-E-115,116
Diagnostic sets including ophthalmic pieces (wall mounted or portable)	PHC-E-117,118,119
Patella hammer	PHC-E-120
Tuning fork (only required in one consultation room)	PHC-E-132
Tape measure	PHC-E-113
Clinical thermometers	PHC-E-130,131
Wall mounted or portable angle poise style examination lamp	PHC-E-110, 154
Blood glucometer	PHC-E-149
Peak flow meter	PHC-E-267
Adult clinical scale up to 150 kg	PHC-E-122,123
HB meter	PHC-E-152
Height measure	PHC-E-125
Urine specimen jars	PHC-E-150,158
Baby scale	PHC-E-124
Bassinet on stand	PHC-E-025

CHECKLIST FOR ELEMENT: FURNITURE IS AVAILABLE AND INTACT IN THE REHABILITATION TREATMENT AREA

Use the checklist below to check whether facility service areas are equipped with sufficient functional furniture in the Rehabilitation treatment area

Item	
**Waiting areas**	
Seating	
c. Adequate seating for all patients	PHC-F-015,016,017
d. Chairs / benches intact	
Notice boards available	PHC-E-102,104
**Treatment area**	
Desk	
a. Available	PHC-F-007
b. Intact (including the drawers)	
Chair (clinician)	
a. Available	PHC-F-002
b. Intact	
At least 1 chair (patient)	
a. Available	PHC-F-005
b. Intact	
Tilting examination couch	
a. Available	PHC-F-030
b. Intact	
Bedside footstool	
a. Available	PHC-F-031
b. Intact	
Instrument trolley	
a. Available	PHC-F-038
b. Intact	
Bar fridge	
a. Available	PHC-F-022
b. Intact	

CHECKLIST FOR ELEMENT: ESSENTIAL EQUIPMENT IS AVAILABLE AND FUNCTIONAL AT THE HEALTH SUPPORT SERVICE TREATMENT AREAS

Use the checklist below to check whether essential equipment is available and functional in rehabilitation treatment area

Item	
**PHYSICAL THERAPY**	
**Equipment – Clinical**	
Aluminium crutches – Adult	PHC-E-212
Aluminium crutches – paediatric	PHC-E-213
Balance pad	PHC-E-214
Basin, portable, large	PHC-E-215
Bobath Ball: 85cm	PHC-E-216
Bobath Ball: 65cm	PHC-E-217
Bobath Ball: 45cm	PHC-E-218
Bobath roller: 50cm	PHC-E-219
Bobath roller: 30cm	PHC-E-220
Construction Play equipment	PHC-E-221
Hot water bottle	PHC-E-222
Mat: gym	PHC-E-223
Mirror: hand-held with cover	PHC-E-224
Mirror: hand-held with handle	PHC-E-225
Nebuliser: ultrasonic	PHC-E-137
Patella hammer	PHC-E-120
Soccer ball	PHC-E-226
Standing frame –large	PHC-E-229
Standing frame –medium	PHC-E-228
Standing frame –small	PHC-E-227

Stethoscope	PHC-E-126
Tape measure: soft, retractable	PHC-E-230
Torch	PHC-E-121
Toys: multisensory	PHC-E-231
Transfer board	PHC-E-232
Walking frame: adult	PHC-E-233
Walking frame: paediatric	PHC-E-126
Walking stick: adult	PHC-E-230
Walking stick: paediatric	PHC-E-234
Wedges: set	PHC-E-235
Weights: ankle and wrist	PHC-E-236
Wheelchair: 25 x30cm	PHC-E-237
Wheelchair: 30x30 cm	PHC-E-238
Wheelchair: 30x40cm	PHC-E-239
Wheelchair: 36x40cm	PHC-E-240
Wheelchair: 41cm wide	PHC-E-241
Wheelchair: 46cm wide	PHC-E-242
Wheelchair: 51cm wide	PHC-E-243
**Equipment – General**	
Allen key set	PHC-E-194
Clamps: adjustable (pair)	PHC-E-195
Claw hammer	PHC-E-072
Cordless Drill	PHC-E-196
Drill bit set	PHC-E-197
Electric foam cutter	PHC-E-198
Electric frying pan	PHC-E-199
Electric jigsaw	PHC-E-202

Extension lead (on reel)	PHC-E-211
Eyelet punch	PHC-E-206
Hacksaw	PHC-E-081
Hacksaw blades	PHC-E-082
Heat gun	PHC-E-201
Measuring rule: folding	PHC-E-204
Multi-plug	PHC-E-210
Pliers set	PHC-E-077
Revolving punch	PHC-E-205
Screwdriver set	PHC-E-073
Shifting spanner	PHC-E-074
Spanner set (swivel-head)	PHC-E-075
Tape measure (industrial)	PHC-E-207
Tool box	PHC-E-157
Utility knife	PHC-E-208
Workbench, portable	PHC-E-209
**Instruments**	
Scissors: splinting	PHC-I-102
Scissors: standard, adult	PHC-I-100
Scissors: standard, child	PHC-I-101
**SPEECH THERAPY**	
**Equipment – Clinical**	
Audiometer portable	PHC-E-247
Cards – themed, speech therapy	PHC-E-248 - 253
HiPro box	PHC-E-254
Noisemakers	PHC-E-255
OAE/AABR screener portable	PHC-E-256

Otolight	PHC-E-257
Otoscope, portable	PHC-E-258
Portable screening tympanometer	PHC-E-259
**Instruments**	
Cerumen management kit	PHC-I-103
cup: ear	PHC-I-104
Curette: cerumen, plastic, adult	PHC-I-105
Curette: cerumen, plastic, paed	PHC-I-106
Curette, cerumen, lighted, magnified	PHC-I-107
Ear loop, Bileau, small	PHC-I-108
Forceps, crocodile	PHC-I-109
Syringe, impression	PHC-I-110

CHECKLIST FOR ELEMENT: FURNITURE IS AVAILABLE AND INTACT AT THE ORAL HEALTH SERVICES

Use the checklist below to check whether facility secvt5rvice areas are equipped with sufficient functional furniture in the Oral Health services

Item	
**Waiting areas**	
**Seating**	
Adequate seating for all patients	PHC-F-015,016,017
Chairs / benches intact	
Notice boards available	PHC-E-102,103
**Treatment area**	
**Desk**	
Available	PHC-F-007
Intact (including the drawers)	
**2x Anaesthetist stool (round stool on wheels)**	
Available	PHC-F-065,066
Intact	
**Fridge**	
Available	PHC-F-022, 043
Functional	

CHECKLIST FOR ELEMENT: ESSENTIAL EQUIPMENT IS AVAILABLE AND FUNCTIONAL AT THE ORAL HEALTH SERVICES

Use the checklist below to check whether essential equipment is available and functional at the Oral Health services

Item	Quantity	
**Equipment**		
Amalgam separator	1	PHC-E-164
Amalgamator: digital	1	PHC-E-166
Autoclave: cassette-type, desktop	1	PHC-E-167
Compressor, 2-4 surgeries, fixed	1	PHC-E-168
Cuspidor/spittoon/water unit	1	PHC-E-169
Dental chair: basic	1	PHC-E-172
Dental chair: portable with portable light (for outreach)	1	PHC-E-173
Dental delivery system with hand-pieces (fixed) PHC-E-187	1	PHC-E-171
Dental delivery system with hand-pieces (Mobile – for outreach)	1	PHC-E-170
Dental light: LED	1	PHC-E-184
Dental curing light (corded or cordless)	1	PHC-E-174, 175
Dental scaler: air	1	PHC-E-185
Stool: dentist	1	PHC-F-065
Stool: dental assistant	1	PHC-F-066
Suction: central, wet (commonest type)	1	PHC-E-188
Suction: central, dry (optional in water-scarce areas)	1	PHC-E-186
Suction: dry, mobile (for outreach and back-up)	1	PHC-E-187
Trolley: emergency (fully equipped)	1	PHC-F-040 and Check-list for Element 166
Ultrasonic cleaner	1	PHC-E-189
Water distillation	1	PHC-E-191
X-ray Aprons	2	PHC-E-181, 182
X-ray: wall-mounted	1	PHC-E-192
X-ray: digital oral imaging plate with computer and cabling.	1	PHC-E-193

Hand-pieces		
3-in-one syringe (included in dental delivery system)	1	PHC-E-164
Air motor (high-speed turbine)	1	PHC-E-176
Contra-angle handpiece	1	PHC-E-179
Slow handpiece	1	PHC-E-180
Slow handpiece motor	1	PHC-E-177
Straight handpiece	1	PHC-E-178
**Dental elevators**		
Cryers elevator left	4	PHC-I-050
Cryers elevator right	4	PHC-I-049
Straight elevator Large	4	PHC-I-047
Straight elevator Medium	4	PHC-I-048
Straight elevator Small	4	PHC-I-046
Warwick-James elevator left	2	PHC-I-052
Warwick-James elevator right	2	PHC-I-113
Warwick-James elevator straight	2	PHC-I-051
**Dental extraction forceps**		
Forceps: tooth extracting Lower bicuspids	6	PHC-I-059
Forceps: tooth extracting Lower molars	6	PHC-I-060
Forceps: tooth extracting Lower roots and crowded incisors	2	PHC-I-061
Forceps: tooth extracting upper Anteriors and canines	6	PHC-I-062
Forceps: tooth extracting Upper bicuspids and roots	6	PHC-I-063
Forceps: tooth extracting Upper molars left	6	PHC-I-064
Forceps: tooth extracting Upper Molars right 90	6	PHC-I-065
Forceps: tooth extracting Upper Molars right 89	6	PHC-I-066
Forceps: tooth extracting Upper roots 29	2	PHC-I-067
Forceps: tooth extracting Upper roots 44 N	2	PHC-I-069
Forceps: tooth extracting Upper roots 29 S	2	PHC-I-068
Forceps: tooth extracting Upper roots 76	2	PHC-I-070
Forceps: tooth extracting Upper roots small	2	PHC-I-071
Forceps: tooth extracting Lower molars, child	6	PHC-I-072

Forceps: tooth extracting Lower teeth and roots, child	6	PHC-I-073
Forceps: tooth extracting Lower canines Adult	6	PHC-I-114
Forceps: tooth extracting Lower Anteriors Adult	6	PHC-I-115
Forceps: tooth extracting Upper incisors and canines, child	6	PHC-I-059
Forceps: tooth extracting Upper Molars, child	6	PHC-I-075
Forceps: tooth extracting Upper teeth and roots, Molars, child	6	PHC-I-076
**Conservative Instruments**		
Amalgam carrier plastic right angle	3	PHC-I-034
Amalgam carrier plastic straight	2	PHC-I-033
Amalgam carver	6	PHC-I-040
Amalgam plugger	4	PHC-I-035, 036
Ball burnisher 2.5-3.0mm	6	PHC-I-037. 038
Bib holders	2	PHC-I-081
Bur Blocks	2	PHC-I-039
Bur brushes	2	PHC-I-111
Cement spatula	2	PHC-I-094
Cotton and Dressing Tweezers	20	PHC-I-098, 099
Cotton pellet holder	2	PHC-I-082
Cotton wool holder (small bowl)	2	PHC-I-012
Dappen dishes	6	PHC-I-042
Dental Explorers/Probes Straight	30	PHC-I-043. 044
Dental syringe Aspirating	30	PHC-I-095
Excavator 125/126	4	PHC-I-055
Excavator 129/130	4	PHC-I-053
Excavator 133/134	4	PHC-I-054
Flat plastic	6	PHC-I-056, 057
Handle Mouth Mirror	30	PHC-I-085
Kidney dishes large	4	PHC-I-013
Kidney dishes small	4	PHC-I-032
Matrix retainer Siqueland Narrow	4	PHC-I-083

Matrix retainer Siqueland Wide	4	PHC-I-084
Mouth Mirrors to it Handle Mouth Mirror	30	PHC-I-086
Needle holder	2	PHC-I-087
Waste receiver	2	PHC-E-002, 005
Periodontal		
Dental probe: periodontal	4	PHC-I-045
Periodontal hoe SG 5F	6	PHC-I-116
Scaler, dental: H6/7	6	PHC-I-117
Other		
Artery forceps	2	PHC-I-003, 004
Handle scalpel	2	PHC-I-007
Mouth gag	4	PHC-I-078, 079, 080
Needle holder	2	PHC-I-087
Protective glasses	4	PHC-C079
Rongeur : dental No.4	4	PHC-I-088
Rongeur: dental No. 5S	4	PHC-I-089
Scissors ligature	2	PHC-I-093
Slab: mixing, glass	2	PHC-I-090
Tongue forceps	2	PHC-I-058
Trimmer: gingival margin U3/U4	4	PHC-I-097
Trimmer: gingival margin Ui/U2	4	PHC-I-096
Wax carver	2	PHC-I-041
Wire ligature forceps	2	PHC-I-077

CHECKLIST FOR ELEMENT: FURNITURE IS AVAILABLE AND INTACT AT THE 24 HOUR EMERGENCY UNIT

Use the checklist below to check whether facility service areas are equipped with sufficient functional furniture in the 24 Hour Emergency Unit

Item	
**Waiting areas**	
Notice boards available	PHC-E-102,103
**Seating**	
a. Adequate seating for family/friends	PHC-F-015,016,017
b. Chairs / benches intact	
**Reception/nurses station**	
**Desk**	
a. Available	PHC-F-007
b. Intact (including the drawers)	
**Chair**	
a. Available	PHC-F-002
b. Intact	
**Clinical areas**	
**1 x Chair per patient**	
a. Available	PHC-F-005
b. Intact	
**Hospital beds**	
a. Available according approved bed capacity	PHC-F-072
b. Intact (check ALL)	
**Bedside footstool**	
a. Available	PHC-F-031
b. Intact	

**Bedside locker**	
a. Available	PHC-F-071
b. Intact	
**Lockable Scheduled Medicine cupboard**	
a. Available	PHC-F-070
b. Intact	
**Dressing trolley**	
a. Available (1 per area)	PHC-F-039
b. Intact (including the drawers)	
**Non-clinical area**	
**Dirty utility/sluice area**	
a. Slop-hopper	
b. In working order	
**Medicine storeroom/cupboard/trolley**	
a. Medicine fridge	PHC-F-043,047
b. In working order	

CHECKLIST FOR ELEMENT: ESSENTIAL EQUIPMENT IS AVAILABLE AND FUNCTIONAL AT THE 24 HOUR EMERGENCY UNIT

Use the checklist below to check whether essential equipment is available and functional in the resuscitation, consultation and observation areas in the 24 Hour Emergency Unit

Item	
**CONSULTATION ROOMS**	
Stethoscope	PHC-E-126
Non-invasive Baumanometer (wall mounted/ portable)	PHC-E-115,116
Adult, paediatric and large cuffs (3) for Baumanometer	PHC-E-115,116
Diagnostic sets including ophthalmic pieces (wall mounted or portable)	PHC-E-117,118,119
Patella hammer	PHC-E-120
Tuning fork (only required in one consultation room)	PHC-E-132
Tape measure	PHC-E-113
Clinical thermometers	PHC-E-130,131
Blood glucometer	PHC-E-149
Peak flow meter	PHC-E-267
Adult clinical scale up to 150 kg	PHC-E-122,123
HB meter	PHC-E-152
Height measure	PHC-E-125
Urine specimen jars	PHC-E-150,158
Baby scale	PHC-E-124
Bassinet	PHC-E-025
Wall mounted or portable angle poise examination lamp	PHC-E-110, 154
Ceiling mounted light	PHC-E-111

CHECKLIST FOR ELEMENT: THERE IS A STERILE PACK FOR MINOR SURGERY

Use the checklist below to check whether equipment for minor surgery is available

Item	Quantity	
**MINOR STITCH / SUTURING TRAY**		
Small stitch tray	1	PHC-I-010
Stitch scissor	1	PHC-I-009
Toothed forceps	1	PHC-I-001
Non-toothed forceps	1	PHC-I-002
Bard-Parker surgical blade handle to fit accompanying blades (blades do not form part of sterilised pack but must be available)	1	PHC-I-007
Mosquito, straight	2	PHC-I-005
Mosquito, curved	2	PHC-I-006
Artery forceps, straight	2	PHC-I-003
Artery forceps, curved	2	PHC-I-004
Needle holder	1	PHC-I-008
Swab holder	1	PHC-I-011

CHECKLIST FOR ELEMENT: RESUSCITATION ROOM IS EQUIPPED WITH FUNCTIONAL BASIC FURNITURE AND RESUSCITATION EQUIPMENT (24 HOUR EMERGENCY UNIT)

Use the checklist below to check whether the emergency/resuscitation room complies with standards for functional basic equipment

Item	
Emergency trolley with lockable medicine drawer and accessories	PHC-F-040
Examination bed	PHC-F-029,030
Wall or ceiling mounted angle-poise-style examination lamp	PHC-E-110,111
Nebuliser OR face mask with nebuliser chamber for adult and paediatric	PHC-E-137
Functional electric powered OR manual suction devices and suction catheters	PHC-E-144, 146
Kick about with bucket	PHC-F-032, 033
Anaesthetist stool (round stool on wheels)	PHC-F-034
Drip stand	PHC-F-028
Dressing trolley	PHC-F-037, 038, 039
Cardiac arrest board	PHC-E-134
Bin (general waste)	PHC-E-003
Suture material	PHC-C-180, 181, 182
Thermal (space) blanket	PHC-C-179
Gloves exam n/sterile gloves: small, medium and large at least one pair of each size	PHC-C-141, 142, 143
Gloves surgical sterile latex: 6 OR 6.5, 7 OR 7.5 and 8, at least one pair of each size	PHC-C-138, 139, 140
Protective face shields OR Goggles with face mask	PHC-C-080, 079
Disposable plastic aprons	PHC-C-137
Disposable non-sterile face masks	PHC-C-077
Resuscitation algorithms	
Resuscitation documentation register	
Wall mounted liquid hand soap dispenser/hand sanitizer	PHC-E-014
Wall-mounted hand paper dispenser	PHC-E-020, 021

CHECKLIST FOR ELEMENT: RESTORE THE EMERGENCY TROLLEY IN THE 24 HOUR EMERGENCY UNIT AFTER EACH USAGE

Use the checklist below to check whether the emergency trolley is sufficiently stocked with unexpired medication

Item	
Laryngoscope handle with functional batteries	PHC-E-135
Adult curved blades for laryngoscope size 2	PHC-E-135
Adult curved blades for laryngoscope size 3	PHC-E-135
Adult curved blades for laryngoscope size 4	PHC-E-135
Paediatric straight blades for laryngoscope sizes 00	PHC-E-135
Paediatric straight blades for laryngoscope sizes 0	PHC-E-135
Paediatric straight blades for laryngoscope sizes 1	PHC-E-135
Spare bulbs for laryngoscope	PHC-E-135
Spare batteries for laryngoscope sizes	
Endotracheal tubes - uncuffed size 2.0mm OR 2.5mm	PHC-C-086,354
Endotracheal tubes - uncuffed size 3.0mm OR 3.5mm	PHC-C-087,355
Endotracheal tubes - uncuffed size 4.0mm OR 4.5mm	PHC-C-088,356
Endotracheal tubes - uncuffed size 5.0mm OR 5.5mm	
Endotracheal tubes - cuffed size 3.0mm	PHC-C-357
Endotracheal tubes - cuffed size 4.0mm	PHC-C-358
Endotracheal tubes - cuffed size 5.0mm	PHC-C-089
Endotracheal tubes - cuffed size 6.0mm	PHC-C-090
Endotracheal tubes - cuffed size 7.0mm	PHC-C-091
Endotracheal tubes - cuffed size 8.0mm	PHC-C-092
Water-soluble lubricant/lubricating jelly	PHC-C-172
Tape to hold tie endotracheal tube in place	
Patella hammer	PHC-E-120
Oropharyngeal airways (Guedel) size 0	PHC-C-113
Oropharyngeal airways (Guedel) size 1	PHC-C-114

Oropharyngeal airways (Guedel) size 2	PHC-C-115
Oropharyngeal airways (Guedel) size 3	PHC-C-116
Oropharyngeal airways (Guedel) size 4	PHC-C-117
Oropharyngeal airways (Guedel) size 5	PHC-C-350
Adult-size introducer, intubating stylet or bougie for endotracheal tubes	PHC-I-125
Paediatricsize introducer, intubating stylet or bougie for endotracheal tubes	PHC-I-126
Magill's forceps for adults	PHC-I-123
Magill's forceps for paediatric	PHC-I-124
Laryngeal masks (supraglottic airways) size 2	PHC-C-351
Laryngeal masks (supraglottic airways) size 2.5	PHC-C-352
Laryngeal masks (supraglottic airways) size 3	PHC-C-353
Laryngeal masks (supraglottic airways) size 4	PHC-C-103
Manual bag valve mask/ manual resuscitator OR self-inflating bag with compatiblemasks for adults	PHC-E-142,143
Manual bag valve mask/ manual resuscitator OR self-inflating bag with compatible masks for paediatric	PHC-E-140,141
Simple face mask OR reservoir mask OR nasal cannula (prongs) for oxygen, adults	PHC-E-111,108,106
Simple face mask OR reservoir mask OR nasal cannula (prongs) for oxygen, paediatric	PHC-E-110,107,105
Face mask for nebuliser OR face mask with nebuliser chamber for adult OR spacers for MDIs (adults and paediatrics)	PHC-E-108
Face mask for nebuliser OR face mask with nebuliser chamber for paediatric OR spacers for MDIs (adults and paediatrics)	PHC-E-107
Intravenous cannula 18g green and appropriate strapping	PHC-C-101,097
Intravenous cannula 20g pink and appropriate strapping	PHC-C-101,098
Intravenous cannula 22g blue and appropriate strapping	PHC-C-101,099
Intravenous cannula 24g yellow and appropriate strapping	PHC-C-101,100
Syringes 3-part: 2ml	PHC-C-118
Syringes 3-part: 5ml	PHC-C-119
Syringes 3-part: 10ml OR 20ml	PHC-C-120,349
Syringes: insulin syringes	PHC-C-121
Needles: 18 (pink) OR 20 (yellow)	PHC-C-122,123
Needles: 21 (green)	PHC-C-124

Needles: 23 (blue) OR 22 (black)	PHC-C-125,126
Sharps container	PHC-E-005
Admin set 20 drops/ml 1.8m /pack	PHC-C-095
Admin set paeds 60 drops/ml 1.8m /pack	PHC-C-096
Automatic External Defibrillator (AED) OR ECG monitor and defibrillator	PHC-E-133
Stethoscope	PHC-E-126
Haemoglobin meter	PHC-E-152
Blood glucometer with testing strips and spare batteries	PHC-E-149
Diagnostic set and batteries including opthalmic pieces (wall mounted or portable)	PHC-E-117,118,119
Rescue scissors (to cut clothing)	PHC-I-020
Paediatric Broselow tape OR Pawper tape	PHC-E-128, 129
Wound care (gauze, bandages, cotton wools, plasters, alcohol swabs and antiseptic solutions)	PHC-C-147 onwards
Urinary (Foley's) catheter silicone/latex 8f	PHC-C-343
Urinary (Foley's) catheter silicone/latex 10f	PHC-C-345
Urinary (Foley's) catheter silicone/latex 12f	PHC-C-345
Urinary (Foley's) catheters: 14f	PHC-C-127
Urinary (Foley's) catheter silicone/latex 16f	PHC-C-346
Urinary (Foley's) catheters: 18f	PHC-C-128
Urinary (Foley's) catheter silicone/latex 20f	PHC-C-347
Urinary (Foley's) catheter silicone/latex 22f	PHC-C-348
Urinary bag specified in the surgical supply list	PHC-C-129
Nasogastric tubes: 600mmfg8	PHC-C-130
Nasogastric tubes: 1000mmfg10 or 12	PHC-C-131, 132
Medication/vaculitre stickers	
**Present individually or in combined multifunctional diagnostic monitoring set**	
Pulse oximeter with adult & paediatric probes	PHC-E-139
Non invasive electronic blood pressure monitoring device including paediatric, adult & large adult cuff sizes	PHC-E-136, 116
Clinical thermometer (in ^{0}C, non-mercury)	PHC-E-130, 131

Emergency medicines (also check expiry dates)
Activated Charcoal
Adrenaline injection 1mg/ml (Epinephrine)
Amlodipine 5mg OR 10mg tablets
Aspirin tablets
Atropine 0.5mg OR 1mg injection
Benzodiazepine e.g. diazepam injection 5mg/ml injection
Calcium gluconate 10% injection
Furosemide 20mg ampoule
Hydrocortisone 100mg/ml injection
Insulin, short acting (stored in the medicine fridge)
Ipratropium 0.25mg/2ml OR 0.5mg/2ml unit dose vial for nebulisation OR 40 mcg MDIs
Isosorbide dinitrate, sublingual, 5mg tablets
Lidocaine/Lignocaine IV 1% OR 2%
Magnesium sulphate 50%, 1g/2ml ampoule (minimum of 14 ampoules required for one treatment course)
Midazolam (1mg/ml OR 5mg/ml) OR Diazepam 5mg/ml
Nifedipine 10mg capsules
Prednisone 5 mg tablets
Promethazine 25mg ampoule
Short-acting sublingual nitrates e.g. glyceryl trinitrate SL OR isosorbide dinitrate
Salbutamol nebulising solution OR nebules OR MDI
Thiamine 100mg injection
Water for injection
**IV Solutions**
50% dextrose (20ml ampoule or 50ml bag) OR 10% dextrose solution
Pediatric solutions e.g. ½ strength Darrows solution AND neonatalyte solution
Sodium Chloride 0.9%

CHECKLIST FOR ELEMENT: FURNITURE IS AVAILABLE AND INTACT IN THE MATERNITY OBSTETRIC UNIT

Use the checklist below to check whether facility service areas are equipped with sufficient functional furniture in the MOU

Item	
**Waiting areas**	
Notice boards available	PHC-E-102,103
**Seating**	
a. Adequate seating for family/friends	PHC-F-015,016,017
b. Chairs / benches intact	
**Reception/nurses station**	
**Desk**	
a. Available	PHC-F-007
b. Intact (including the drawers)	
**Chair**	
a. Available	PHC-F-002
b. Intact	
**Service area**	
**1 x Chair per patient**	
a. Available	PHC-F-005
b. Intact	
**Anaesthetist stool**	
a. Available	PHC-F-034
b. Intact	
**Hospital beds**	
a. Available according approved bed capacity	PHC-F-072, 073, 074
b. Intact (check ALL)	

**Over-bed patient trolley**		
a.	Available	PHC-F-075
b.	Intact	
**Obstetric tilting couch**		
a.	Available	PHC-F-029
b.	Intact	
**Bedside footstool**		
a.	Available	PHC-F-031
b.	Intact	
**Bedside locker**		
a.	Available	PHC-F-071
b.	Intact	
**Lockable Scheduled Medicine cupboard**		
a.	Available	PHC-F-070
b.	Intact	
**Dressing trolley**		
a.	Available (1 per ward)	PHC-F-039
b.	Intact (including the drawers)	
**Double bowl stand with two bowls**		
a.	Available	PHC-F-044
b.	Intact	
**Non-clinical area**		
**Dirty utility/sluice area**		
a.	Freezer (for placenta)	PHC-F-076
b.	In working order	
**Medicine storeroom/cupboard/trolley**		
a.	Medicine fridge	PHC-F-043,047
b.	In working order	

CHECKLIST FOR ELEMENT: ESSENTIAL EQUIPMENT IN THE MATERNITY OBSTETRIC UNIT

Use the checklist below to check whether essential equipment is available and functional in the Maternity Obstetric Unit

Item	
Stethoscope	PHC-E-126
Foetal Stethoscope Plastic	PHC-E-264
Non-invasive Baumanometer (wall mounted/ portable)	PHC-E-115,116
Adult, paediatrics and large cuffs (3) for Baumanometer	PHC-E-115,116
Diagnostic sets -including ophthalmic pieces(wall mounted or portable)	PHC-E-117,118,119
Peak flow meter	PHC-E-267
Patella hammer	PHC-E-120
Tape measure	PHC-E-113
Clinical thermometers	PHC-E-130,131
Ceiling mounted or portable - examination lamp	PHC-E-111, 154
Adult clinical scale upto 150 kg	PHC-E-122,123
Baby scale	PHC-E-124
Bassinet with trolley and mattress	PHC-E-025
Blood glucometer	PHC-E-149
HB meter	PHC-E-152
Height measure	PHC-E-125
Urine specimen jars	PHC-E-150,158
CTG Machine (cardiotocographic)	PHC-E-265
Incubator Transport	
Infant Warmer: radiant	PHC-E-190, 260, 261
Suction Unit Mobile Electrical 2x 2lit Bottle	PHC-E-144
Bowl Lotion S/Steel 150mm	PHC-I-012
Rescue scissors	PHC-I-020
Autoclave Stand alone (mobile) Approx 100 lit	PHC-E-157
Bed Pan washer	
Tray Instruments washing with lid 183mm x 140 x 17 mm	PHC-E-157

CHECKLIST FOR ELEMENT: STERILE OBSTETRIC DELIVERY PACKS ARE AVAILABLE IN THE MOU

Use the checklist below to check whether there are emergency sterile obstetric packs available

Item	Quantity	
**NON-NEGOTIABLE**		
Stitch scissor	1	PHC-I-009
Episiotomy scissor	1	PHC-I-019
Cord scissor	1	PHC-I-018
Dissecting forceps non-toothed (plain)	1	PHC-I-002
Dissecting forceps toothed	1	PHC-I-001
Artery forceps, straight, long	2	PHC-I-003
Needle holder	1	PHC-I-008
Small bowl	2	PHC-I-012
Kidney dishes OR receivers (big)	2	PHC-I-013
**EXTRAS (not part of sterilised pack)**		
Basin	1	PHC-I-021
Stainless-steel round bowl, large	1	PHC-I-022
Green towels	4	
Disposable apron	2	PHC-C-137
Gauzes	5	PHC-C-150
Vaginal tampons	1	PHC-C-158
Sanitary towels	2	PHC-C-156
Round cotton wool balls	1 pack	PHC-C-148
Umbilical cord clamps	2	PHC-C-163

CHECKLIST FOR ELEMENT: RESTORE THE EMERGENCY TROLLEY IN THE MOU DAILY OR AFTER EACH USAGE

Use the checklist below to check whether the emergency trolley is sufficiently stocked with unexpired medication in the MOU

Item	
Laryngoscope handle with functional batteries	PHC-E-135
Adult curved blades for laryngoscope size 2	PHC-E-135
Adult curved blades for laryngoscope size 3	PHC-E-135
Adult curved blades for laryngoscope size 4	PHC-E-135
Paediatric straight blades for laryngoscope sizes 00	PHC-E-135
Paediatric straight blades for laryngoscope sizes 0	PHC-E-135
Paediatric straight blades for laryngoscope sizes 1	PHC-E-135
Spare bulbs for laryngoscope	PHC-E-135
Spare batteries for laryngoscope sizes	
Endotracheal tubes - uncuffed size 2.0mm OR 2.5mm	PHC-C-086,354
Endotracheal tubes - uncuffed size 3.0mm OR 3.5mm	PHC-C-087,355
Endotracheal tubes - uncuffed size 4.0mm OR 4.5mm	PHC-C-088,356
Endotracheal tubes - uncuffed size 5.0mm OR 5.5mm	
Endotracheal tubes - cuffed size 3.0mm	PHC-C-357
Endotracheal tubes - cuffed size 4.0mm	PHC-C-358
Endotracheal tubes - cuffed size 5.0mm	PHC-C-089
Endotracheal tubes - cuffed size 6.0mm	PHC-C-090
Endotracheal tubes - cuffed size 7.0mm	PHC-C-091
Endotracheal tubes - cuffed size 8.0mm	PHC-C-092
Water-soluble lubricant/lubricating jelly	PHC-C-172
Tape to hold tie endotracheal tube in place	
Patella hammer	PHC-E-120
Oropharyngeal airways (Guedel) size 0	PHC-C-113
Oropharyngeal airways (Guedel) size 1	PHC-C-114

Oropharyngeal airways (Guedel) size 2	PHC-C-115
Oropharyngeal airways (Guedel) size 3	PHC-C-116
Oropharyngeal airways (Guedel) size 4	PHC-C-117
Oropharyngeal airways (Guedel) size 5	PHC-C-350
Adult-size introducer, intubating stylet or bougie for endotracheal tubes	PHC-I-125
Paediatricsize introducer, intubating stylet or bougie for endotracheal tubes	PHC-I-126
Magill's forceps for adults	PHC-I-123
Magill's forceps for paediatric	PHC-I-124
Laryngeal masks (supraglottic airways) size 2	PHC-C-351
Laryngeal masks (supraglottic airways) size 2.5	PHC-C-352
Laryngeal masks (supraglottic airways) size 3	PHC-C-353
Laryngeal masks (supraglottic airways) size 4	PHC-C-103
Manual bag valve mask/ manual resuscitator OR self-inflating bag with compatiblemasks for adults	PHC-E-142,143
Manual bag valve mask/ manual resuscitator OR self-inflating bag with compatible masks for paediatric	PHC-E-140,141
Simple face mask OR reservoir mask OR nasal cannula (prongs) for oxygen, adults	PHC-E-111,108,106
Simple face mask OR reservoir mask OR nasal cannula (prongs) for oxygen, paediatric	PHC-E-110,107,105
Face mask for nebuliser OR face mask with nebuliser chamber for adult OR spacers for MDIs (adults and paediatrics)	PHC-E-108
Face mask for nebuliser OR face mask with nebuliser chamber for paediatric OR spacers for MDIs (adults and paediatrics)	PHC-E-107
Intravenous cannula 18g green and appropriate strapping	PHC-C-101,097
Intravenous cannula 20g pink and appropriate strapping	PHC-C-101,098
Intravenous cannula 22g blue and appropriate strapping	PHC-C-101,099
Intravenous cannula 24g yellow and appropriate strapping	PHC-C-101,100
Syringes 3-part: 2ml	PHC-C-118
Syringes 3-part: 5ml	PHC-C-119
Syringes 3-part: 10ml OR 20ml	PHC-C-120,349
Syringes: insulin syringes	PHC-C-121
Needles: 18 (pink) OR 20 (yellow)	PHC-C-122,123
Needles: 21 (green)	PHC-C-124

Needles: 23 (blue) OR 22 (black)	PHC-C-125,126
Sharps container	PHC-E-005
Admin set 20 drops/ml 1.8m /pack	PHC-C-095
Admin set paeds 60 drops/ml 1.8m /pack	PHC-C-096
Automatic External Defibrillator (AED) OR ECG monitor and defibrillator	PHC-E-133
Stethoscope	PHC-E-126
Haemoglobin meter	PHC-E-152
Blood glucometer with testing strips and spare batteries	PHC-E-149
Diagnostic set and batteries including opthalmic pieces (wall mounted or portable)	PHC-E-117,118,119
Rescue scissors (to cut clothing)	PHC-I-020
Paediatric Broselow tape OR Pawper tape	PHC-E-128, 129
Wound care (gauze, bandages, cotton wools, plasters, alcohol swabs and antiseptic solutions)	PHC-C-147 onwards
Urinary (Foley's) catheter silicone/latex 8f	PHC-C-343
Urinary (Foley's) catheter silicone/latex 10f	PHC-C-345
Urinary (Foley's) catheter silicone/latex 12f	PHC-C-345
Urinary (Foley's) catheters: 14f	PHC-C-127
Urinary (Foley's) catheter silicone/latex 16f	PHC-C-346
Urinary (Foley's) catheters: 18f	PHC-C-128
Urinary (Foley's) catheter silicone/latex 20f	PHC-C-347
Urinary (Foley's) catheter silicone/latex 22f	PHC-C-348
Urinary bag specified in the surgical supply list	PHC-C-129
Nasogastric tubes: 600mmfg8	PHC-C-130
Nasogastric tubes: 1000mmfg10 or 12	PHC-C-131, 132
Medication/vaculitre stickers	
**Present individually or in combined multifunctional diagnostic monitoring set**	
Pulse oximeter with adult & paediatric probes	PHC-E-139
Non invasive electronic blood pressure monitoring device including paediatric, adult & large adult cuff sizes	PHC-E-136, 116
Clinical thermometer (in ^{0}C, non-mercury)	PHC-E-130, 131

Emergency medicines (also check expiry dates)
Activated Charcoal
Adrenaline injection 1mg/ml (Epinephrine)
Amlodipine 5mg OR 10mg tablets
Aspirin tablets
Atropine 0.5mg OR 1mg injection
Benzodiazepine e.g. diazepam injection 5mg/ml injection
Calcium gluconate 10% injection
Furosemide 20mg ampoule
Hydrocortisone 100mg/ml injection
Insulin, short acting (stored in the medicine fridge)
Ipratropium 0.25mg/2ml OR 0.5mg/2ml unit dose vial for nebulisation OR 40 mcg MDIs
Isosorbide dinitrate, sublingual, 5mg tablets
Lidocaine/Lignocaine IV 1% OR 2%
Magnesium sulphate 50%, 1g/2ml ampoule (minimum of 14 ampoules required for one treatment course)
Midazolam (1mg/ml OR 5mg/ml) OR Diazepam 5mg/ml
Nifedipine 10mg capsules
Prednisone 5 mg tablets
Promethazine 25mg ampoule
Short-acting sublingual nitrates e.g. glyceryl trinitrate SL OR isosorbide dinitrate
Salbutamol nebulising solution OR nebules OR MDI
Thiamine 100mg injection
Water for injection
Oxytocin 5 OR 10IU/ml AND Ergometrine 0.5mg OR oxytocin/ergometrine 5U/0,5 mg (Combination)
**IV Solutions**
50% dextrose (20ml ampoule or 50ml bag) OR 10% dextrose solution
Pediatric solutions e.g. ½ strength Darrows solution AND neonatalyte solution
Sodium Chloride 0.9%

CHECKLIST FOR ELEMENT: ESSENTIAL EQUIPMENT IS AVAILABLE AND FUNCTIONING IN THE TOP AND MMC PROCEDURE ROOM

Use the checklist below to check whether essential equipment is available and functional in the TOP and MMC procedure room

Item	
Stethoscope	PHC-E-126
Non-invasive Baumanometer (wall mounted/ portable)	PHC-E-115, 116
Adult and large cuffs (2) for Baumanometer	PHC-E-115, 116
Diagnostic sets -including ophthalmic pieces(wall mounted or portable)	PHC-E-119, 119, 117
Tape measure	PHC-E-113
Clinical thermometers	PHC-E-130, 131
Blood glucometer	PHC-E-149
Adult clinical scale up to 150 kg	PHC-E-122, 123
HB meter	PHC-E-152
Height measure	PHC-E-125
Freezer for products of conception and foreskins	PHC-F-076
Gynaecology examination couch with stirrups	PHC-F-029
Tilting examination couch	PHC-F-062
Ceiling mounted, Wall mounted or portable angle poise style examination lamp	PHC-E-111, 110, 154

CHECKLIST FOR ELEMENT: SHARPS ARE DISPOSED OF APPROPRIATELY

Use the checklist below to check whether sharps are disposed of appropriately

Item
Waste is properly segregated
Sharps are disposed of in impenetrable, tamperproof containers
Sharps containers are disposed of when they reach the limit mark
Sharps containers are placed on work surface or in wall mounted brackets

PHC-E-005

CHECKLIST FOR ELEMENT: TEMPERATURE OF THE MEDICINE ROOM/DISPENSARY IS MAINTAINED WITHIN THE SAFETY RANGE

Use the checklist below to check whether the medicine in the medicine room/dispensary is maintained within the safety range

Item	
There is at least one functional, wall-mounted room thermometer	PHC-E-114
The temperature of the pharmacy is recorded daily	PHC-C-134
The temperature of the pharmacy is maintained within the safety range	

CHECKLIST FOR ELEMENT: COLD CHAIN PROCEDURE FOR VACCINES IS MAINTAINED

Use the checklist below to check whether the cold chain for vaccines is maintained

Item	
There is a standard operating procedure for the maintenance of cold chain for vaccines	
Facility has a vaccine or medicine refrigerator with a thermometer	PHC-F-042, 043, 049, 050, PHC-E-153, 042
The temperature of the refrigerator is recorded twice daily, 7 hours apart (check one month's record)	PHC-C-135
The temperature of the refrigerator is maintained between 2-8 ºC (check one month's record)	
There is a cooler box for storage of vaccines if needed	PHC-E-035
Ice packs are available for use as needed	PHC-C-203

CHECKLIST FOR ELEMENT: SPECIMENS ARE COLLECTED, PACKED, STORED AND PREPARED FOR TRANSPORTATION ACCORDING TO THE PRIMARY HEALTH CARE LABORATORY HANDBOOK

Use the checklist below to check whether specimens are handled according to the PHC Laboratory Handbook

Grouping of specimens

Group A	Group B	Group C
Blood<br>Pleural effusion<br>Sputum<br>Stool<br>Urine	Pap smear	MCS (Microscopy, culture band sensitivity)

Item	
**General**	
Specimens are clearly labelled	
Each laboratory request form is correctly completed	
There is at least one functional wall mounted thermometer in area for lab specimens are stored for courier collection	PHC-E-114
The temperature of the storage area for lab specimens is recorded daily	PHC-C-134
**Group A specimens**	
Samples are kept away from direct sunlight	
Where the room temperature exceeds 25°C, samples are stored in the fridge (at +- 5°C)	PHC-F-022
Length of storage does not exceed 24 hours, stored at room temperature (+- 20-25°C)	
**Group B specimens**	
Stored at room temperature	
Stored inside a slide carrier (envelope)	
**Group C specimens**	
Samples placed into the transport medium provided (where appropriate)	
Samples kept away from direct sunlight	
Where room temperature exceeds 25°C, samples are stored in the fridge (+- 5°C)	PHC-F-022
Length of storage does not exceed 24 hours, stored at room temperature (+-20-25°C)	

CHECKLIST FOR ELEMENT: HEALTH CARE WASTE IS MANAGED APPROPRIATELY

Use the checklist below to check whether health risk care waste is managed appropriately

Item	
Sanitary disposal bins with functional lids OR health care risk waste box	PHC-E-004
* Sanitary disposal bins/boxes lined with appropriate colour plastic bags	PHCC196
Sanitary disposal bins/boxes are clean and not overflowing	
Health care risk waste disposal bins with functional lids OR health care risk waste box	PHC-E-047
Health care risk waste disposal bins/boxes lined with red colour plastic bags	PHC-C-195
Health care risk waste disposal bins/boxes contain only health care waste	
Health care risk waste disposal bins/boxes are not overflowing	
Bins available for general waste	PHC-E-046, 049
Bins for general waste are lined with appropriate coloured bags	PHC-C-190 onwards

* If disposable boxes for sanitary waste is used where gel granules in the bottom of the box treat the waste, no bag is required and facility can score “Y”

CHECKLIST FOR ELEMENT: THERE IS A STANDARD SECURITY GUARD-ROOM OR THE FACILITY HAS AN ALARM SYSTEM LINKED TO ARMED RESPONSE

Use the checklist below to check whether facility security adheres to standard guidelines

**Item**	
Does the facility have an alarm system linked to armed response (if Yes, checklist for security guardroom and security equipment must not be assessed. If No, assess checklist for security guardroom and security equipment)	
**Security guard room**	
Kitchenette – sink with cupboard underneath	
Table	PHC-F-008
Chair	PHC-F-003
Functioning lights	
**Security equipment for security officer(s) and accompanying stationery**	
Baton	
Handcuffs OR Cable ties	
Incident book	
Metal detector	
Telephone OR two-way radio OR dedicated cellphone	

CHECKLIST FOR ELEMENT: ALL STAFF MEMBERS WEAR AN IDENTIFICATION TAGS

Use the checklist below to check that the staff on duty wear official identification tags

Staff member	
Staff member 1	PHC-C-202
Staff member 2	PHC-C-202
Staff member 3	PHC-C-202
Staff member 4	PHC-C-202
Staff member 5	PHC-C-202
Staff member 6	PHC-C-202
Staff member 7	PHC-C-202
Staff member 8	PHC-C-202
Staff member 9	PHC-C-202
Staff member 10	PHC-C-202

Note: Identification tag must include the emblem of the facility/district or provincial department of health, full names/initials and surname of the staff member

CHECKLIST FOR ELEMENT: HAND HYGIENE AND SANITARY FACILITIES ARE AVAILABLE AT THE 8 HOUR SERVICE AREA

Use the checklist below to check whether there is running water, toilet paper, liquid hand wash soap and disposable hand paper towels

Item	
**Toilet**	
Functional hand wash basin with taps	
Running water	
Toilet paper	PHC-C-081
Liquid hand wash soap	PHC-C-058
Disposable hand paper towels	PHC-C-072, 073
**8 Hour service area**	
Functional hand wash basin with taps	
Running water	
Liquid hand wash soap/hand sanitiser	PHC-C-058
Disposable hand paper towels	PHC-C-072, 073
**Health Support and Oral Health**	
Functional hand wash basin with taps	
Running water	
Liquid hand wash soap/hand sanitiser	PHC-C-058
Disposable hand paper towels	PHC-C-072, 073

INDEX

ITEM SPECIFICATIONS

A
Acid etch 348
AED replacement Pads 285
Aerosol insecticide 438
Air freshener 439
Aluminium crutches 88
Amalgamator: digital 68
Amalgam capsule 349
Amalgam carrier: plastic 206
Amalgam plugger 208
Amalgam separator: 2 surgeries 67
Amniotomy hook 286
Anaesthetic: topical 350
Antibacterial filter: portable suction machine 287
Antibacterial filter: trolley suction 288
Appointment cards: general 268
Apron: dental, plastic 351
Aprons: disposable 413
Articulating paper: thin, blue 352
Audiometer: portable 109
Autoclave: cassette-type, desktop 69
Autoclave: tabletop 63
Automated external defibrillator 46

B
Bags: waste 440
Balance pad 89
Ball burnisher 209
Bandage: crepe 289
Basic tool kit 118
Basin 200
Basin: portable, large 90
Bassinet 470
Batteries: hearing aid 375
Bed: hospital, 2-section 471
Bed: hospital, mattress 472
Bed: hospital, mattress cover 473
Bed: hospital, obstetric 474
Bib: plastic 353
Bicarbonate of soda: sachets 376
Bin: pedal 4,5 l 123
Bin: pedal 20l 124
Bin: sanitary towel 126
Bins: brazier 172
Bin: sharps 127
Bins: waste, heavy duty 147
Bin: wall-mounted, refuse bag holding unit 125
Blade: scalpel 290
Blade: stitch-cutter 291
Blood pressure measurement: aneroid type 18
Blood pressure monitor: automated non-invasive 19
Bobath ball 91
Bobath roller 92
Bolts and nuts: assorted 422
Bonding agent: self-etch 354
Bowie-Dick test packs 411
Bowl: large 201
Bowl: small 202
Breadboard: one-handed 377
Broom 148
Broom: interior use 128
Brush: applicator 355
Bucket for kick-about 475
Buckets: colour coded 129
Building maintenance 631
Bur Block 210
Bur brush 211

C
Cabinet: medicine, mobile 476
Cabinet: scheduled drugs, wall-mounted 477
Cable ties: nylon, ...378
Cardboard: A4, various colours 379
Cardiac arrest board 48
Cardiotocograph 20
Cards: therapy, speech 110
Carver: Amalgam 212
Carver: wax 213
Catheter: intrathoracic, silicon 292
Catheter: suction 293
Cement: zinc oxide and eugenol 356
Cerumen management kit 261
Chair: office 507
Chair: operator 508
Chair: stackable, metal frame 509
Chair: stackable, netted mid-back 510
Chair: visitor 511
Chlorine compounds (bleach) 441
Cleaning 667
Clinical 724
Clock: wall 173
Cloths: cleaning 442
Cold chain 686
Coloured pencils, pens, crayons 380
Complaints and suggestions box 174
Composites 357
Compressor: 2-4 surgeries 70
Construction play equipment 93
Contact adhesive 423
Cooler box 140

Cord clamp: umbilical ...346
Cotton wool: balls 1g (500s) ...294
Cotton wool pellets ...358
Couch: treatment, hydraulic Bobath (neuro) ...478
Credenza ...512
Cupboard: sterile stock ...513
Curette: cerumen, lighted ...263
Curette: cerumen, lighted ...381
Curette: cerumen, plastic ...262
Cushion covers: wheelchair ...383
Cushion: wheelchair ...382
Cuspidor/Spittoon/Water Unit ...71

D

Dappen dish ...214
Delivery system: fixed, with hand pieces, non-fibre-optic ...73
Delivery system: mobile, with hand pieces, non-fibre-optic ...72
Dental chair: basic ...74
Dental chair: portable ...75
Dental curing light ...76
Dental Explorer ...215
Dental floss: Waxed ...359
Dental handpieces: non-fibre-optic ...77
Dental light: LED ...78
Dental Probe: periodontal ...216
Dental scaler: air ...79
Desk: 3-drawer, L-shaped ...514
Desk: rectangular ...515
Detergent: liquid or powder ...443
Diagnostic ...542
Diagnostic combination set: wall mounted ...23
Diagnostic set: portable ...21
Diagnostic set: wall mounted ...22
Dishwashing liquid ...444
Disposable paper cups ...466
Drainage system: chest, underwater, adult ...295
Drain cleaner ...445
Dressing pack: disposable ...296
Drip stand ...479
Dry socket alveolar paste ...360
Dust pan and brush set ...130

E

Ear loop: Bileau, small ...264
Ear mould impression material and scoop ...384
Ear mould impression Otolight: replacement globe ...385
Ear mould impression Otolight: replacement tips ...386
ECG electrodes: disposable, self-adhesive, for resting ECG ...297
Edge Trimmer/brush cutter: heavy duty ...149
Electrocardiograph: 12 channel ...24
Elevator: root, apical ...217
Elevator: root, Cryers ...218
Elevator: root, Warwick-James ...219
Endotracheal tubes: cuffed ...298
Endotracheal tubes: uncuffed ...299
Essential ...570
Examination couch ...480
Examination couch: portable ...481
Examination couch: rehab ...482
Examination light: ceiling-mounted ...39
Examination light: headlamp ...40
Examination light: mobile, without battery ...41
Examination light: wall-mounted ...42
Excavator: dental ...220
Exercise band: latex ...387
Extension cable: electrical, garden ...150
Extension cable: electrical, general purpose ...120
Eye patch: disposable ...300

F

Face shield ...414
Feeding cup ...388
Feeding spoon ...389
Ferrule: rubber ...390
Files and accessories ...463
Filing cabinet: four-drawer ...518
Filling Cabinet: bulk ...516
Fire extinguisher ...144
Fire hose on reel ...145
Fire safety ...690
Fissure sealants ...361
Flat plastic ...221
Floor polisher: electric, single disc ...519
Floor polisher replacement Cleaning disc ...448
Floor polisher replacement Scouring disc ...449
Floor polymer remover ...446
Floor protective polymer ...447
Fluoride gel ...363
Fluoride trays ...362
Foam: EVA ...391
Foam: HD compressed 1300 ...393
Foam: high-density, 36/20 ...392
Foetoscope ...25
Foot stool ...483
Forceps: artery, curved ...187
Forceps: artery, straight ...188
Forceps: crocodile ...265
Forceps: mosquito, curved ...191
Forceps: mosquito, straight ...192
Forceps: non-toothed ...189
Forceps: tongue ...222
Forceps: toothed ...190
Forceps: tooth extracting, Lower Anteriors Adult ...223
Forceps: tooth extracting, Lower Bicuspids ...224
Forceps: tooth extracting, Lower Canines Adult ...225
Forceps: tooth extracting, Lower Molars ...226
Forceps: tooth extracting, Lower molars, child ...238
Forceps: tooth extracting, Lower roots and Crowded Incisors ...227
Forceps: tooth extracting, Lower teeth and roots, child ...239
Forceps: tooth extracting, Upper Anteriors and Canines ...228
Forceps: tooth extracting, Upper Bicuspids and Roots ...229
Forceps: tooth extracting, Upper Incisors and Canines, child ...240
Forceps: tooth extracting, Upper Molars, child ...241
Forceps: tooth extracting, Upper Molars, Left ...230

Forceps: tooth extracting, Upper Molars, Right 231
Forceps: tooth extracting, Upper Molars, Right 232
Forceps: tooth extracting, Upper roots 233
Forceps: tooth extracting, Upper roots 234
Forceps: tooth extracting, Upper roots 235
Forceps: tooth extracting, Upper roots, Small 237
Forceps: tooth extracting, Upper teeth and roots, Molars, child 242
Forceps: wire ligature 243
Fork 151
Freezer: chest, human tissue 520

G
Gag (mouth prop) 244
Garden 693
Gardening set: 3-piece 152
Gauze: absorbent, Type 1, sterile, peel pack (5 swabs) 301
Gauze: paraffin, 100X100 (box of 10) 302
Gauze: swabs, plain 303
General 763
Glass ionomer 364
Gloves: non-sterile 416
Gloves: sterile 415
Glucometer 61
Glucometer consumables 337
Glue: cyanoacrylate, rapid 424
Glue: wood, waterproof 425
Goggles 417
Gown: disposable 418
Gun safe 175

H
Haemoglobinmeter consumables and accessories (older models) 338
Haemoglobinmeter consumables (newer models) 339
Haemoglobinometer 62
Haemostat sponge 365
Hand rub: alcohol-based 450
Hedge clipper: shears, manual 153
Helix test kit and strips 412
High level disinfectants 451
HiPro box 111
HIV test kit: rapid 340
Hoe 154
Holder: Bib 245
Holder: cotton pellet 246
Hosepipe: garden 155
Hot water bottle: standard 94

I
Identification tag 467
Infant warmer: mattress 50
Infant warmer: radiant 49
Infant warmer: temperature probe 51
Instrument steriliser: desktop 65
Intravenous administration set 304
Intravenous cannula 305
Intravenous cannula: winged 306
Introducer (intubation stylet): adult 184
Introducer (intubation stylet): child 185

K
Kettle: electric 167
Kick-about 484
Kidney dish 203
Kitchen 712

L
Ladder: aluminium, folding 121
Lancets, Multipurpose 341
Laryngoscope set 52
Laundry detergent 452
Lawn mower: Electric, heavy duty 156
Lawn mower: petrol, heavy duty 157
Locker: bedside, steel 485
Lubricant gel, sterile 307
Lubricating/releasing oil: spray can 426

M
Magill forceps: adult 182
Magill forceps: child 183
Malaria Rapid Diagnostic test kit 342
Mask: laryngeal, adult 308
Mask: oxygen, diluter type 309
Mask: oxygen, re-breathing type 310
Masonite 394
Maternity health record 269
Mat: gym 95
Matrix retainer 247
Microfibre cloth for static head mop 453
Microwave oven: 25l, 900W manual 521
Mirror 522
Mirror: handheld 96
Miscellaneous 716
Monitor: multi-function vital signs, including SpO_2 53
Mop: colour-coded 131
Mop: static head, complete 132
Multi-plug 122

N
Nails: assorted 427
Nasal Cannula: oxygen 311
Nasogastric catheter 312
Nebuliser 54
Needle holder 193
Needle holder 249
Needles: hypodermic 313
Needles: Hypodermic, Dental 314
Noise makers 112
Notice board: wall-mounted 176

O
OAE/AABR screener (portable) 113
Oil: 2-stroke for brush cutter 428
Oil: 20W50, for petrol lawnmower 429
Oil: arnica 395

Order forms and books 464
Oropharyngeal airway (Guedel) 318
Otolight....114
Otoscope: portable....115
Otostops 396
Oxygen regulator with flow-meter 55

P
Padding: cast orthopaedic....315
Pamphlet-holding boxes....177
Paper: print-out, tympanometer....397
Paper towel dispenser: folded towels....133
Paper towel dispenser: roll 134
Paper towel: folded....454
Paper towel: roll 455
Particulate mask (N-95 respirator)....419
Paste: prophylaxis 366
Patella hammer 26
Patient information register....270
Patient record for adults....271
Patient record for children 272
Peak flow meter....27
Peak Flow Meter: mouth-piece, disposable....317
Pelvic straps....398
Periodontal Hoe 250
Pick mattock....158
Plaster of Paris bandage 316
Plaster: self-adhesive, roll....319
Polishing kit....367
Polishing strips 368
Polyester strips (composite) 369
Poster display boards....178
Pressure garment material: elastonet 399
Pulse oximeter: stand-alone 28

R
Rack: cutlery....169
Rack: dish....168
Rake: leaf and grass, heavy duty....159
Rake: steel, heavy duty....160
Razor, disposable....320
Referral forms: general....273
Referral forms: WBPHCOT....274
Refrigerator: bar type....523
Refrigerator: double door....524
Refrigerator: single door....525
Refrigerator: vaccine, chest....486
Refrigerator: vaccine, upright model....487
Register: assets....275
Register: Daily reception headcount....276
Register: Monthly tally summary 277
Register : Patient safety incident....278
Register: PHC comprehensive 279
Register: Weekly tally notepad....280
Resuscitators: manual....56
Rhesus test kit 344
Rivets: blind, aluminium, assorted 430
Road to Health Booklet for Boys 281
Road to Health Booklet for Girls 282
Rongeur: dental 251

S
Sanitary all-purpose cleaner 456
Sanitary towels: maternity....347
Sanitary towels: standard....321
Scale: adult, with height board....29
Scale: adult, without height board 30
Scale: baby....31
Scaler, Dental....252
Scalpel handle: long, No. 3 194
Scissors: dissecting....195
Scissors: episiotomy 204
Scissors: ligature....253
Scissors: rescue 186
Scissors: splinting 260
Scissors: standard....259
Scissors: stitch....196
Scissors: umbilical 205
Screen: bedside, replacement curtains....489
Screen: bedside, wheeled, complete....488
Screws: chipboard, assorted....431
Screws: roofing, with sealing washers 432
Screws: self-tapping, assorted 433
Sealant: silicone, marine, clear....434
Seating: 2-seater....526
Seating: 3-seater....527
Seating: 4-seater....528
Sewing kit 400
Sheath: incontinence....322
Shovel: square mouth....161
Sick note 283
Skin traction kit 323
Slab: mixing, glass....254
Slasher 162
Smoke alarm 146
Soap: antimicrobial....457
Soap dispenser....135
Soap: handwashing, liquid non-antimicrobial 458
Soccer ball....97
Spade....163
Spatula: Cement....255
Specialised....577
Splinting material: thermoplastic sheets 401
Splinting material: thermoplastic tape 402
Spray bottle 136
Squeegee....137
Stadiometer....32
Standing frame: prone upper standing frame....98
Steel cabinet: lockable, 6 shelves....529
Stethoscope....33
Stitch tray: small....197
Stockinette: 100mm, roll 25m 324
Stockinette: 150mm, roll 25m 325
Stool: anaethetist....490

Stool: dental assistant.....491
Stool: dentist.....492
Stool: height-adjustable, with backrest.....493
Storage rack: exercise ball, wall-mounted.....530
Suction: dry, 2-3 surgeries.....80
Suction: dry, mobile.....81
Suction machine: portable.....57
Suction machine: trolley type.....58
Suction: wet, 2-4 surgeries.....82
Surgical mask.....420
Surgical scrub brush sponges.....459
Suture material: intra-oral.....370
Suture materials.....326
Swab: 70% isopropyl alcohol.....327
Swab holder.....198
Syringe: dental.....199
Syringe: dental, aspirating.....256
Syringe: ear.....34
Syringe: hypodermic.....328
Syringe: impression.....266

T

Table: folding.....531
Table: over- bed.....494
Table: trapezoidal.....532
Tampon: vaginal.....329
Tape: Broselow.....59
Tape: insulation.....435
Tape measure: retractable, soft.....99
Tape: measuring.....43
Tape: MUAC.....44
Tape: PAWPER and flipper chart.....60
Tape: surgical. Adhesive, microporous, 25mm.....330
Tape: surgical. Adhesive, microporous, 50mm.....331
Temperature recording forms.....284
Thermal blanket.....332
Thermometer: data logging.....141
Thermometer: digital, ear-type.....35
Thermometer: digital, pen type.....36
Thermometer probe covers: disposable.....333
Thermometer: refrigerator.....142
Thermometer: room.....143
Tip: dental suction (saliva ejector).....371
Toilet paper.....460
Toilet roll dispenser.....138
Toothbrush.....372
Toothpaste: dental, fluoride.....373
Torch: pupillary.....37
Towelling.....403
Toys: multisensory.....100
Transfer board.....101
Trimmer: Gingival margin.....257
Trolley: anaesthetic.....495
Trolley: emergency.....496
Trolley: gas cylinder.....497
Trolley: janitor.....139
Trolley: medical dressing, large.....498
Trolley: medical dressing, small.....500
Trolley: medical dressing with bucket and bowl attachment.....501
Trolley: medical instruments, medium.....499
Trolley: patient, with mattress.....502
Trolley: soiled instruments and linen.....503
Trolley: soiled linen, complete.....504
Tube: stomach washout.....334
Tumble dryer.....533
Tuning fork.....38
Tweezers: Cotton and Dressing.....258
Tympanometer: portable, screening.....116

U

Ultrasonic cleaner.....83
Ultrasound gel.....335
Urinary catheters (Foley's).....336
Urine collection.....45
Urine dip sticks.....345
Urn: electric.....170

V

Vacuum cleaner.....534
Vacuum cleaner replacement filter set.....461
Varnish: cavity liner.....374
Varnish: wood.....404
Velcro: circle, self-adhesive.....405
Velcro: loop, 5cm width, 50m roll.....406
Viewing box: X-ray.....505

W

Walking frame.....102
Walking stick.....104
Washboard: one-handed.....407
Washers: locking, assorted.....436
Washers: plain, galvanised.....437
Washing machine: front loader.....535
Washing machine: top loader.....536
Watercooler: free-standing.....171
Water distillation.....84
Watering can.....164
Water packs for cold box.....468
Wedges: set of 4.....105
Weight band: ankle and wrist.....106
Wheelbarrow.....165
Wheelchair.....107
Wheelchair gloves.....409
Wheelchair: porter.....506
Wheelchair spares kit.....408
Whiteboard.....179
Wood.....410
Writing materials.....465

X

X-ray Apron.....85
X-ray: Digital oral imaging plate with a computer.....87

TECHNICAL SPECIFICATIONS

A
Adult manual resuscitator ... 591
Allen key set ... 631
Amalgamator: digital ... 609
Amalgam separator: 2 surgeries ... 608
Autoclave: tabletop ... 602
Automated external defibrillator (AED) ... 577

B
Bassinet ... 724
Bassinet, plastic, spare ... 724
Bed: hospital, 2-section ... 725
Bed: hospital, mattress ... 726
Bed: hospital, mattress cover ... 727
Bed: hospital, obstetric ... 728
Bedpan ... 575
Bin: general Waste, 140l, green, with wheels ... 693
Bin: general Waste, 240l, black, with wheels ... 693
Bin: Healthcare Risk Waste, 140l, red, with wheels ... 693
Bin: pedal, 4,5l ... 667
Bin: pedal, 20l ... 668
Bin: sanitary towel ... 670
Bin: sharps ... 671
Bin: wall-mounted, refuse bag holding unit ... 669
Blood pressure monitor: automated, non-invasive, desktop model ... 544
Brazier bin: 105x135x75 ... 716
Brazier bin: 105x190x75 ... 716
Brazier bin: 140x210x130 ... 716
Brazier bin: 140x280x130 ... 716
Broom: garden ... 694
Broom: interior use ... 672
Broselow tape ... 597
Bucket for kick-about ... 730
Buckets: colour-coded ... 673

C
Cabinet: medicine, mobile ... 731
Cabinet: scheduled drugs, wall-mounted ... 732
Cardiac arrest board ... 580
Cardiotocograph ... 547
Chair: office, mid-back ... 763
Chair: operator, high back ... 764
Chair: stackable, ABS shell ... 765
Chair: stackable, netted, mid-back ... 766
Chair: visitor, mid back ... 767
Child manual resuscitator ... 590
Clamps: adjustable (pair) ... 632
Clock: wall ... 717
Complaints and suggestions box ... 718
Compressor: 2-4 surgeries ... 611
Credenza ... 768
Cuspidor/Spittoon/Water Unit ... 612

D
Data logging thermometer ... 687
Delivery system: fixed, with hand pieces, non-fibre-optic ... 614
Delivery system: mobile, with hand pieces, non-fibre-optic ... 613
Dental chair: basic ... 616
Dental chair: portable ... 617
Dental curing light: corded ... 618
Dental curing light: cordless ... 618
Dental light: LED ... 622
Dental scaler: air ... 623
Desk: L-shape, 3-drawer ... 770
Desk, rectangular ... 772
Desktop Autoclave S-Type for Dental Instruments ... 610
Diagnostic combination set: wall-mounted ... 553
Diagnostic set: portable ... 550
Diagnostic set: wall-mounted ... 551
Disclaimer ... ii
Dispenser: toilet roll ... 684
Drill bit set ... 634
Drill, cordless ... 633
Drip stand: mobile ... 734
Dust pan and brush set ... 674

E
Edge trimmer/brush cutter: petrol, heavy duty ... 695
Electrocardiograph (ECG): 12 channel ... 554
Examination couch ... 735
Examination couch: portable ... 736
Examination couch: rehab ... 737
Examination light: clinical. Ceiling-mounted, directional ... 570
Examination light: clinical, wall-mounted, directional ... 573
Examination light: headlamp ... 571
Examination light: mobile, without battery ... 572
Extension cable: electrical, garden ... 696
Extension cable: electrical, general purpose, 3m ... 635
Extension cable: electrical, general purpose, 5m ... 635
Extension cable: electrical, general purpose, 10m ... 635
Extension cable: on reel, 20m ... 635

F
File, flat, combined ... 636
Filing cabinet: 4-drawer ... 776
Filing cabinet: bulk ... 774
Fire extinguisher ... 690
Fire hose on reel ... 691
Floor polisher: electric, single disc ... 777
Foam cutter, electric ... 637
Foot stool ... 738
Fork: garden, 4-pronged ... 697
Frying pan, electric ... 638
Funnel, plastic, medium ... 639

G
Gardening set: 3-piece....698
Glucometer....599
Gun safe....719

H
Hacksaw, all-metal....640
Hacksaw blades, assorted....641
Haemoglobinmeter....601
Hammer: claw, approximately 450gm....642
Hearing protector (muffs)....643
Heat gun, electric....644
Hedge shears....699
Hoe....700
Hosepipe: garden....701

I
Infant manual resuscitator....589
Infant warmer: radiant, mobile....581
Instrument steriliser: desktop....605

J
Jigsaw blades, set....646
Jigsaw, electric....645

K
Kettle: electric....712
Kick-about....739

L
Ladder: aluminium....647
Laryngoscope set for PHC facilities....583
Lawn mower: electric, heavy duty....702
Lawnmower: petrol, heavy duty....703
Locker: bedside, steel....740

M
Mattress for bassinet, spare....724
Measuring rule, folding....648
Microwave oven....778
Mirror....779
Monitor: multifunction vital signs, including SpO_2....584
Mop: black....675
Mop: blue....675
Mop: red....675
Mop: static head, complete....676
Multi-plug....649

N
Nebuliser....587
Notice board: A2 size....720
Notice board: A3 size....720

O
Oral Health....12
Oxygen regulator with flow-meter....588

P
Pamphlet-holding boxes....721
Paper towel dispenser: folded towels....677
Paper towel dispenser: roll....678
Patella hammer....557
PAWPER tape....598
Peak flow meter....558
PEEP valve....592
Pick mattock....704
Pliers set....650
Poster display boards: A0 size....722
Poster display boards: A1 size....722
Pruning saw, medium....651
Pruning shears, bypass, heavy-duty....652
Pulse oximeter: stand-alone....559
Punch, eyelet....654
Punch, revolving....653

R
Rack: cutlery....713
Rack: dish....713
Rake: leaf and grass, heavy duty....705
Rake: steel, heavy duty....706
Refrigerator: bar type....780
Refrigerator: double door....781
Refrigerator: single door....782
Refrigerator: vaccines, chest model....741
Refrigerator: vaccines, upright model....742
Rehabilitation Services....12
Riveter, hand....655
Room thermometer....689

S
Scale: adult, with height board....560
Scale: adult, without height board....561
Scale: baby....562
Scraper, paint, medium....656
Screen: bedside, replacement curtains....744
Screen: bedside, wheeled. Complete with curtain.....743
Screwdriver set....657
Seating: waiting area, 2-seater....783
Seating: waiting area, 3-seater....784
Seating: waiting area, 4-seater....785
Sharpening stone, medium/fine combined....658
Shifting spanner: 0-40mm....659
Shovel: square mouth....707
Slasher....708
Smoke alarm....692
Soap dispenser....679

Spade, garden 709
Sphygmomanometer: aneroid 542
Spray bottle: 2l 681
Spray bottle: 10l backpack 682
Spray bottle: 750ml 680
Squeegee....683
Stadiometer....563
Stationery cabinet 786
Sterile stock cabinet 787
Stethoscope: dual head 564
Stool: anaesthetist 745
Stool: dental assistant....746
Stool: dentist 747
Stool: height adjustable, with backrest 748
Storage rack: exercise ball, wall-mounted 788
Suction: dry, 2-4 surgeries....624
Suction machine: portable....593
Suction: mobile 625
Suction: trolley-type 595
Suction: wet, 2-4 surgeries....626
Swivel-head spanner set 660
Syringe: Ear....565

T

Table: folding....789
Table: over- bed....749
Table: trapezoidal....790
Tape measure: clinical....574
Tape measure: industrial 661
Thermometer: digital, ear-type 566
Thermometer, digital pen type....567
Thermometer: refrigerator....688
Tool box....662
Torch: pupillary....568
Treatment couch: hydraulic Bobath (neuro) 733
Trolley: anaesthetic....750
Trolley: emergency....751
Trolley: gas cylinder....752
Trolley: janitor....685
Trolley: medical dressing, large 753
Trolley: medical dressing, small....755
Trolley: medical dressing with bucket and bowl attachment....756
Trolley: medical instruments, medium....754
Trolley: patient....757
Trolley: soiled instruments and linen, complete 758
Trolley: soiled linen, complete....760
Tumble dryer....792
Tuning fork set....569
Turbine: full range 619

U

Ultrasonic cleaner....627
Urine jar or flask 576
Urn: electric 714
Utility knife, retractable blade....663

V

Vaccine cooler box....686
Vacuum cleaner 793
Voltage tester, contactless....664

W

Washing machine: front loader 794
Washing machine: top loader....795
Watercooler: free-standing, bottled 715
Water distillation....628
Watering can 710
Water packs for vaccine cool box....686
Water-pump pliers....665
Wheelbarrow 711
Wheelchair: porter....762
Whiteboard: 1200x1000 723
Whiteboard: 1800x1200 723
Whiteboard: 3000x1200 723
Workbench, folding, portable....666

X

X-ray: Digital oral imaging plate with a computer 630
X-Ray viewing box: wall mounted....761
X-ray: wall-mounted 629

National Department of Health
Private Bag X828
Pretoria

Department: Health
REPUBLIC OF SOUTH AFRICA
